# Stereo-Differentiating Asymmetric Rh(I)-Catalyzed Pauson–Khand Reaction: A DFT-Informed Approach to Thapsigargin Stereoisomers

**DOI:** 10.1021/jacs.4c11661

**Published:** 2024-12-20

**Authors:** Fatemeh Haghighi, Luke T. Jesikiewicz, Corrinne E. Stahl, Jordan Nafie, Amanda Ortega-Vega, Peng Liu, Kay M. Brummond

**Affiliations:** † Department of Chemistry, University of Pittsburgh, Pittsburgh, Pennsylvania 15260, United States; ‡ BioTools, Inc., Jupiter, Florida 33478, United States

## Abstract

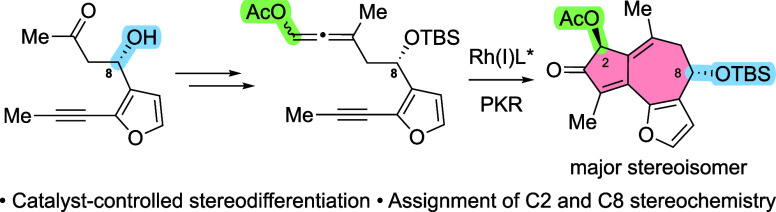

We report a stereo-differentiating dynamic kinetic asymmetric Rh(I)-catalyzed Pauson–Khand reaction, which provides access to an array of thapsigargin stereoisomers. Using catalyst-control, a consistent stereochemical outcome is achieved at C2—for both matched and mismatched cases—regardless of the allene-yne C8 stereochemistry. The stereochemical configuration for all stereoisomers was assigned by comparing experimental vibrational circular dichroism (VCD) and ^13^C NMR to DFT-computed spectra. DFT calculations of the transition-state structures corroborate experimentally observed stereoselectivity and identify key stabilizing and destabilizing interactions between the chiral ligand and allene-yne PKR substrates. The robust nature of our catalyst-ligand system places the total synthesis of thapsigargin and its stereoisomeric analogues within reach.

## Introduction

**Figure 1:**
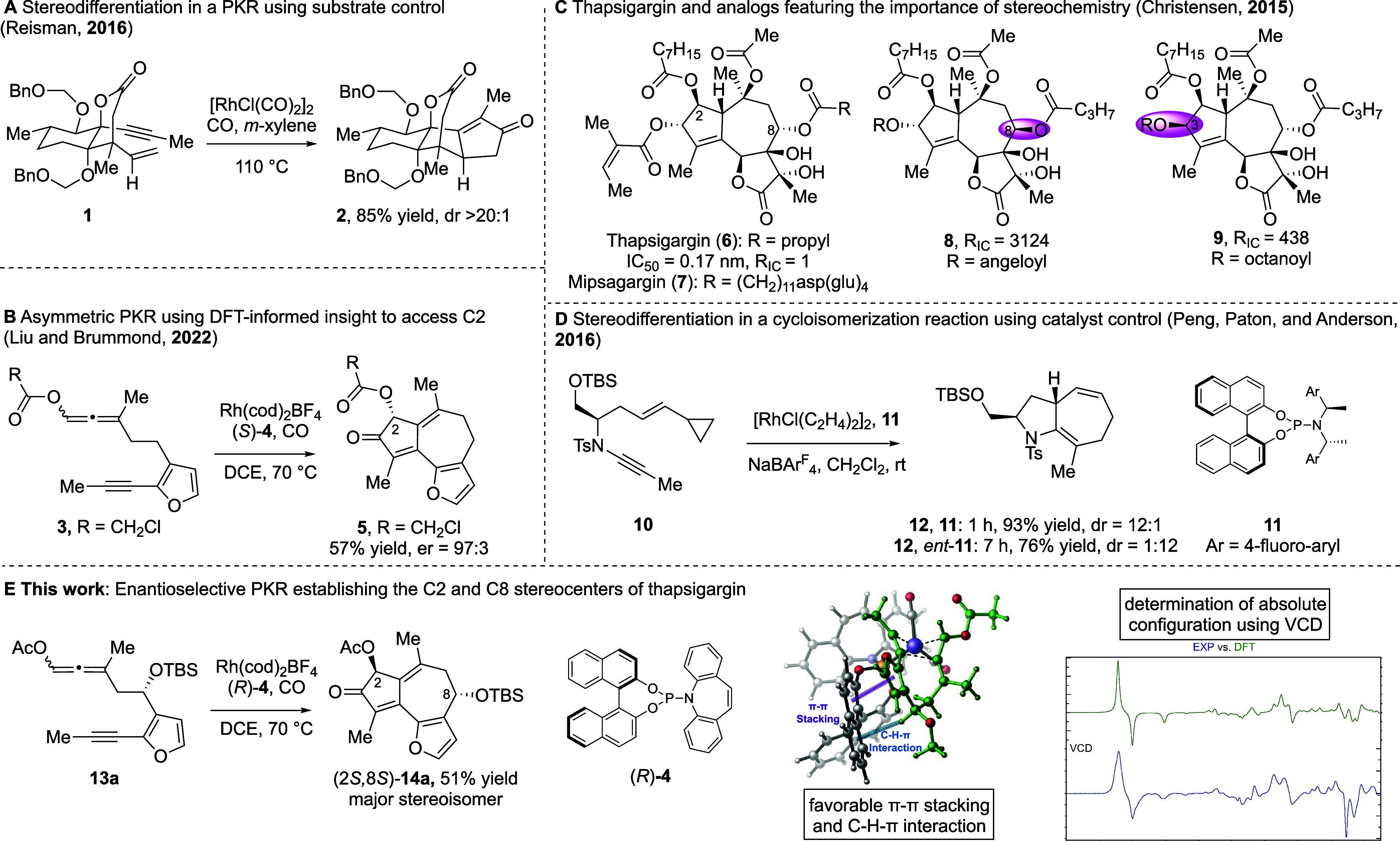
(A) Stereodifferentiation using substrate control; (B) DFT-informed asymmetric PKR; (C) thapsigargin and analogues, highlighting key stereochemical sites; (D) stereodifferentiation using catalyst control; (E) this work: stereodifferentiation in a DyKAT-based asymmetric PKR and access to stereoisomeric analogues of thapsigargin.

The transition metal-catalyzed Pauson–Khand reaction (PKR) is a powerful method for synthesizing ring-fused cyclopentenones—an unsaturated motif found in complex molecular targets and which often bears stereogenic centers.^1−4^ For substrates with at least one preset stereocenter, high diastereoselectivity is often observed in the PKR.^5−8^ Reisman and coworkers recently reported an elegant example of this type of substrate-controlled stereodifferentiation of enyne **1** via a Rh(I)-catalyzed PKR to give cyclopentenone **2** in 20:1 dr; product **2** was then utilized to complete the total synthesis of (+)-ryanodol (Figure 1A).^9^ Yet, asymmetric catalyst-controlled PKRs have rarely been applied to natural product synthesis,^10^ despite the prevalence of chiral nonracemic 5,5-, 5,6-, and 5,7-ring systems in these targets. Further, the PKR is a mechanistically complex reaction, with both precursor structure and the steric and electronic properties of the catalyst impacting reactivity and selectivity. Still, the absence of a catalyst-controlled asymmetric PKR represents a serious omission in the synthetic chemist’s toolbox.

In 2017, we utilized a combination of experiment and computational modeling to establish that racemic allene-ynes could be converted into bicyclo[5.3.0]decadienone products in high yield and good enantiomeric ratios (ers) through an enantioselective Rh(I)-catalyzed PKR.^11^ This stereoconvergent transformation relies on the rapid scrambling of the allenyl carboxy group’s axial chirality under the reaction conditions, facilitating the selective PKR of one enantiomer of the substrate. By utilizing the computed reaction energy profile to establish the oxidative cyclization step as stereodetermining, an effective chiral catalyst was identified in which the corresponding transition state contained a low-energy five-coordinate rhodium complex. The computationally derived mechanistic insights into ligand effects on reactivity and selectivity (e.g., Δ*G*^‡^ and ΔΔ*G*^‡^) were iteratively correlated with experimental data (e.g., yield and enantioselectivity) to inform further reaction design.

Recently, we extended this chiral-catalyzed method to more complex furan-containing allene-ynes to afford 5,7,5-ring systems—an ever-present substructure in natural products (Figure 1B).^12^ Application to the total synthesis of thapsigargin (**6**) offers a high-impact and exciting target for asymmetric PKR technology. Thapsigargin (**6**) is one of the most stereochemically complex members of the 6,12-guaianolide sesquiterpene family. Further, thapsigargin is a potent inhibitor of sarco/endoplasmic reticulum calcium ATPase (SERCA) and has recently shown antiviral activity against the replication of human coronaviruses, including SARS-CoV-2.^13−16^ Mipsagargin (**7**), a prodrug of thapsigargin, has been used in several phase II clinical studies.^17,18^ While over a hundred thapsigargin analogues have been reported, and a pharmacophore model for SERCA inhibition postulated, previous studies utilized only two thapsigargin stereoisomers, the C8-inverted isomer **8** and C3-inverted isomer **9**, each of which is less bioactive than thapsigargin itself (*R*_IC_ = 3124 and 438, respectively) [*R*_IC_ = IC_50_(analogue)/IC_50_(thapsigargin)] (Figure 1C).^19−21^

To date, total syntheses of thapsigargin have used a chiral pool starting material.^22−24^ While chiral pool materials are readily available in known stereochemical configuration, they can be expensive and often require significant modification to reach the desired target.^25−28^ Chiral catalysis offers a more direct synthetic route; yet, there are only three reported asymmetric syntheses of any guaianolide natural product that do not depend on a chiral pool starting material. Of these, two involved asymmetric C–C bond forming processes ([4 + 3]^29^ or [2 + 1]-cycloaddition^30^), and the other utilizes a sharpless asymmetric epoxidation.^31^

Herein, we report our asymmetric PKR approach to thapsigargin, which establishes the absolute stereochemistry of C8 early. While C8 is four bonds away from the stereogenic carbon introduced by the PKR, DFT-computed transition-state (TS) structures place the chiral biaryl backbone of the phosphoramidite ligand in the proximity of the C8 substituent. As such, the absolute configuration of the C8 stereogenic center interacts noncovalently with the phosphoramidite ligand, impacting the overall transition state stability and stereochemical outcome of the PKR (Figure 1E).

Inspired by Peng, Paton, and Anderson who used insights from their DFT calculations to achieve a double stereo-differentiating Rh(I)-catalyzed [5 + 2] carbocyclization for both matched and mismatched cases (Figure 1D),^32^ we asked whether the chiral catalyst, optimized previously for a model system, could be used in a stereoconvergent, stereo-differentiating PKR aimed at thapsigargin. Herein, we report a rare example of a DFT-informed, enantioselective dynamic kinetic asymmetric transformation (DyKAT) that utilizes the existing C8 stereocenter of the allene-yne **13** to initiate an asymmetric catalyst-controlled PKR. This approach provides access to the full stereochemical array of chiral nonracemic thapsigargin isomers, none of which are available from chiral pool starting materials or semisynthesis (Figure 1E).^33^ Access to these thapsigargin stereoisomers will enable important structure–activity relationship (SAR) studies and a broader understanding of thapsigargin’s biological mode of action.

## Results and Discussion

### Synthesis of Racemic and Nonracemic C8-Substituted Chiral Allene-yne Precursors

#### Installation of the C8 Stereogenic Center of Thapsigargin (**6**)

**Scheme 1:**
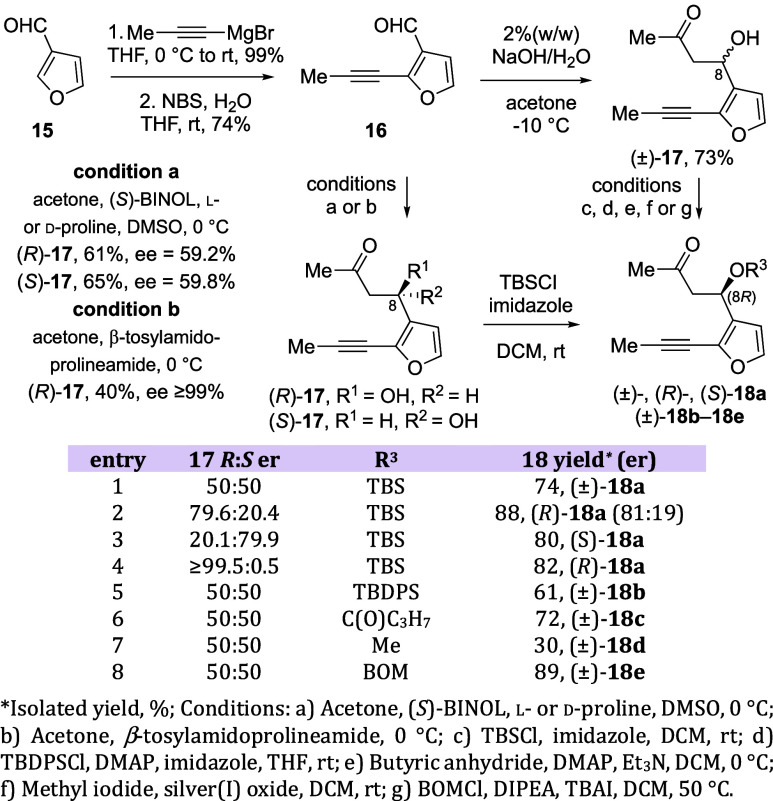
Synthesis of Aldol Adducts **18a**–**18e**

Reacting 3-furfural (**15**) with propynyl magnesium bromide, followed by treatment with *N*-bromosuccinimide and water, yielded oxidative rearrangement product **16** in 74% yield over two steps (Scheme 1).^12^ Subsequent three-carbon homologation of aldehyde **16** with concomitant installation of the C8 stereocenter was then accomplished using either an asymmetric aldol reaction (only a few examples use heteroaryl aldehydes, but there are no reports with 3-furaldehyde^34−47^) or a nonstereoselective reaction (acetone and 2% (w/w) aqueous sodium hydroxide).^48^

To prepare enantioenriched ketone **17**, aldehyde **16** was reacted with acetone in the presence of (*S*)-BINOL and either l- or d-proline (Scheme 1). Reaction with l-proline produced product (*R*)-**17** in 61% yield and 59.2% enantiomeric excess (ee), while d-proline gave ketone (*S*)-**17** in 65% yield and 59.8% ee, as determined by ^1^H NMR using Eu(hfc)*_3_* as a chiral shift reagent (see Supporting Information for asymmetric aldol reaction optimization studies).^49^ Both chiral shift reagents and chiral HPLC analysis showed the same % ee for enantioenriched ketone **17**.

Given the mixture of stereoisomers formed during this process, we wondered whether epimerization of the C8 stereocenter could occurr under the (*S*)-BINOL/proline conditions. As such, aliquots were obtained at two time points (early in the reaction and at completion of the reaction) and analyzed by ^1^H NMR using Eu(hfc)_3_ as a chiral shift reagent. These studies found no significant change in the enantiomeric ratio of product **17** over time (see Supporting Information), suggesting that the observed enantioselectivity resulted from the reaction itself rather than a subsequent epimerization.

Interestingly, when β-tosylamidoprolineamide, a 1,2-diamine-based bifunctional prolineamide catalyst, was used instead of the (*S*)-BINOL/proline system,^50^ ketone (*R*)-**17** was obtained in ≥99% ee. The easy accessibility of the proline catalyst and scalability of the reaction provided adequate material to be tested for stereoselectivity in the PKR in our early investigations.

#### Establishing the Absolute Configuration of Both Enantiomers of Ketone **17**

Vibrational circular dichroism (VCD) was used to determine the absolute configuration of the ketone **17** enantiomers, given that product **17** is an oil and VCD can be performed on solutions.^51−56^ Moreover, VCD has been established as a reliable tool for assigning the absolute configuration of more than 300 natural products.^57^ In the process, we noted that ketone **17** is sensitive to dehydration, and care needed to be taken to remove all acid from the CDCl_3_ solvent before dissolution. In acid-free CDCl_3_, no changes were observed in the IR or VCD spectra over time.

**Figure 2:**
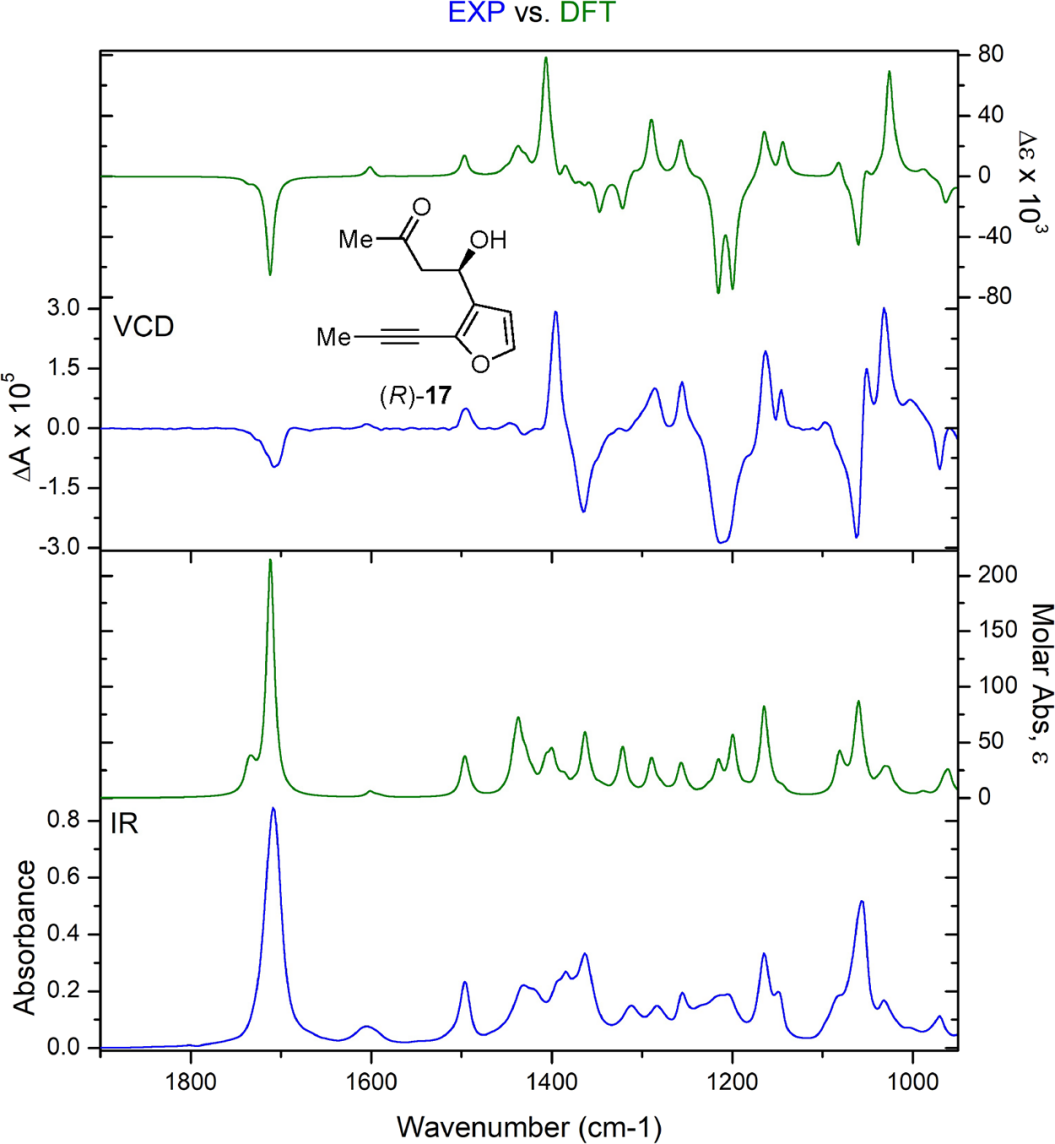
DFT-calculated (green) and experimental (blue) VCD and IR spectra of ketone (*R*)-**17**.

The experimental VCD data for enantiomers (*R*)-**17** and (*S*)-**17** were compared to DFT-calculated IR and VCD spectra using CompareVOA software (BioTools, Jupiter FL) to quantify the results. In both cases, a high *S*_fg_ (overlap) value was observed for IR and VCD (confidence level = 99%, Figures 2 and S9, S10).^58,59^ The analysis included an initial search with MMFF94 yielding 28 conformers (within a 7.0 kcal/mol energy window), which were further optimized using two different basis sets (6-31G(d) and cc-pVTZ) each with two functionals (B3LYP and B3PW91). While the absolute configuration could be determined using any of the calculation methods, the larger cc-pVTZ basis set represented the data most accurately. In that case, the lowest energy conformer (B3LYP/cc-pVTZ—used in plots) represented 73% of the Boltzmann weighted average and showed an intramolecular H-bond between the hydroxyl group and the carbonyl oxygen, suggesting that a H-bond is present in CDCl_3_ solution. Using Goodman’s metric, a Cai•factor (configuration: absolute information) of 61 was measured for (*R*)-**17**, a high score indicating confident assignment of absolute stereochemistry (Figure S11).^60^ Importantly, the assigned absolute configuration aligns well with the predictive model for the organocatalyzed aldol reaction, with the enamine intermediate of l-proline showing a *re*-facial approach of acetone to aldehyde **16** through a half-chair transition state that leads to ketone (*R*)-**17** as the major enantiomer.^61−63^

#### Synthesis of Allene-yne Precursors **13a**–**f**

As we knew that the steric nature of the pre-existing C8 stereocenter of the allene-yne would be important for the DyKAT PKR process, the C8 hydroxyl group was protected as a *tert*-butyldimethylsilyl ether (R^1^ = TBS), a *tert*-butyldiphenylsilyl ether (R^1^ = TBDPS), a butanoyl ester (R^1^ = C(O)C_3_H_7_), a methyl ether, or a benzyloxymethyl ether (Scheme 1). In the racemic case, protected hydroxy ketones (±)-**18a**–**18e** were formed in 30–89% yield. Enantioenriched/pure (*R*)- and (*S*)-**18a** were obtained in 80–88% yield. The enantioenriched (*R*)-**18a** was obtained with 62% ee as determined by ^1^H NMR using Eu(hfc)_3_ as a chiral shift reagent.

**Scheme 2:**
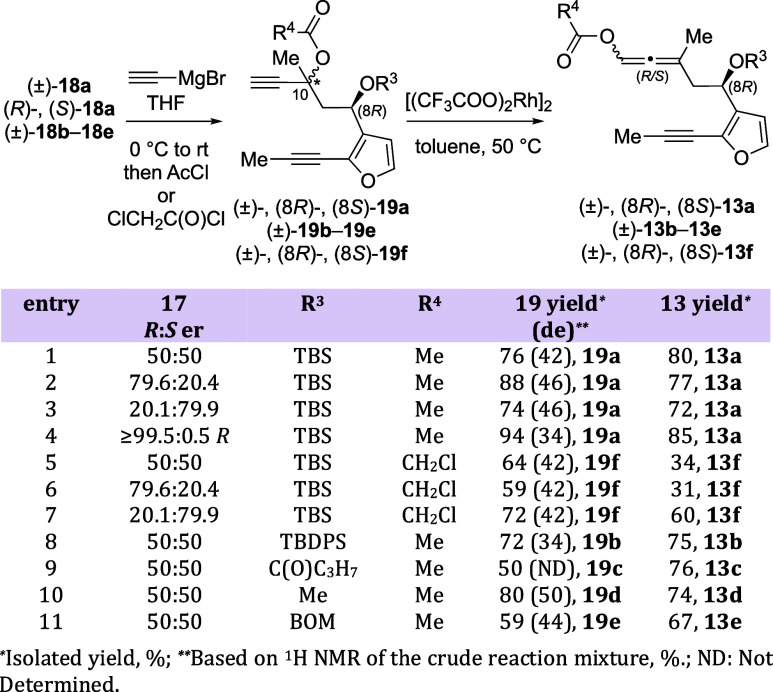
Synthesis of Allene-yne Precursors **13a**–**13f**

Conversion of methyl ketones **18a**–**18e** into the required allene was accomplished using a 3-step/2-pot protocol (Scheme 2). The initial reaction of each ketone **18a**–**18e** with ethynylmagnesium bromide provided propargyl alcohols that were trapped in situ with either acetyl chloride or chloroacetyl chloride to afford diynes (±)-**19a**–**19f** in 50–80% yield. Enantioenriched/pure (*R*)- and (*S*)-**19a** and **19f** were obtained under similar reaction conditions in 59–94% yield and 34–46% diastereomeric excess (de). Finally, treatment of diynes **19a**–**19f** with 5 mol % Rh(II) trifluoroacetate dimer at 50 °C gave racemic allene-ynes (±)-**13a**–**13f** in 34–80% yield and 0% de. Enantioenriched/pure (*R*)- and (*S*)-**13a** and **13f** were obtained in 31–85% yield and 0% de.^12^ The mixture of diastereomers is inconsequential at this point, as the axial chirality of the allenyl carboxy group is rapidly scrambled in the asymmetric PKR.^11^ If an Rh scavenger (SiliaMetS Thiourea) was added to the mixture upon completion of the reaction, improved yields could be achieved (Scheme 2, compare entries 5 and 6 with entry 7). Because the ees for compounds **17a** and **18a** (59.2% vs 62%) are nearly identical, we assume that the ees for **19a**, **19f**, **13a**, and **13f** are unchanged from **17**. This rationalization is further supported by the kinetic resolution studies shown below (Figure 6).

### Rh(I)-Catalyzed Racemic PKR of (±)-**13a**–**13e**

With racemic allene-ynes **13a**–**13e** in hand, we sought to determine the effect of the C8 group on the yield and diastereoselectivity of the PKR in the presence of an achiral or racemic Rh(I)-catalyst.^12^ Reaction of OTBS-substituted (±)-**13a** with cationic [Rh(cod)_2_BF_4_] and triphenyl phosphine in DCE (0.02 M) under a carbon monoxide atmosphere at 70 °C for 112 h afforded the PKR product (±)-**14a** in 42% yield and −26% de in favor of the *cis* diastereomer, as determined by ^13^C NMR (Table 1, entry 1). Attempts to increase the reaction concentration from 0.02 to 0.1 M gave complete consumption of (±)-**13a** in 20 h, but a 26% yield of (±)-**14a** due to the competing formation of aldehyde side product **S8** (entry 2). Subjecting OTBDPS-substituted allene-yne (±)-**13b** to the reaction conditions provided (±)-**14b** in 60% yield and −42% de (entry 3), while butanoyl (±)-**13c** gave (±)-**14c** in 53% yield and −34% de (entry 4).^64^ The methoxy-substituted allene-yne (±)-**13d** gave a 15% yield of (±)-**14d** in −16% de (entry 5). We hypothesized that having a BOM ether at the C8 position would improve the selectivity of the PKR due to π–π interactions between the substrate and the ligand in the oxidative cyclization transition state. However, (±)-**13e** gave (±)-**14e** in 33% yield and −20% de, which was similar to that observed for the OMe group (compare entries 5 and 6). Although the effect of the C8 group on diastereoselectivity is subtle (16–42% de), the larger groups show increasing *cis:trans* ratios (entries 1–6). The diastereoselectivity corresponds to the size of the silyloxy group, rather than its A-value (A values: OTBS = 1.06–1.77; OTBDPS = 0.56–0.62 kcal/mol).^65^ Interestingly, the size of the C8 group had a greater impact on the reaction yield, with (±)-**14b** (*R* = OTBDPS) provided in 60% yield and (±)-**14d** (*R* = OMe) in 15% yield. Lower yields for the products having smaller groups may be due to the C8 oxygen coordinating to the cationic Rh(III) of the catalyst reaction center, facilitating hydrolysis of the allenyl carboxy group and leading to an increase in the level of side product **S10**.

**Table 1:**
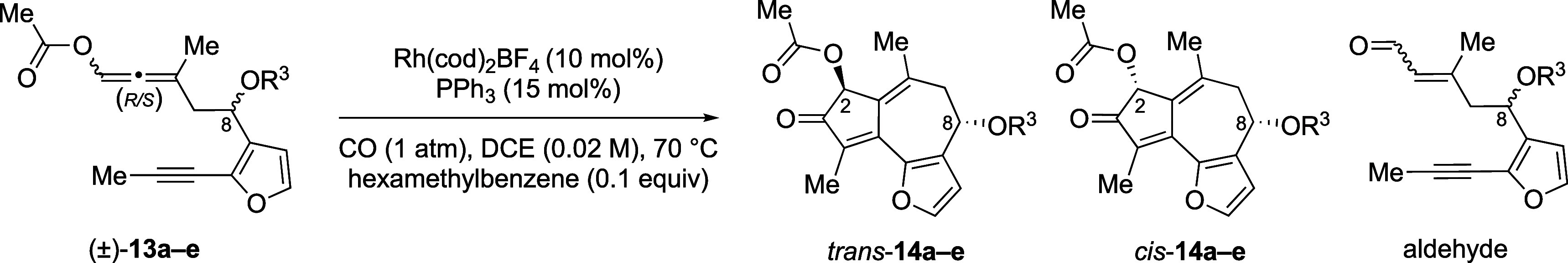
Influence of the C8-Substituent on the Yield and Diastereoselectivity of Rh(I)-Catalyzed PKR Using Achiral or Racemic Catalysts and Allene-ynes (±)-**13a**–**e**

**entry**	**allene**-yne	**R**^**3**^	**deviation fromstandard condition**	**timein h**	**isolated combined yield14 (NMR yield) %**	*trans: cis***14**^a^**(de %)**	**14: aldehyde**^a^
1	(±)-**13a**	TBS	**-**	112	42 (36)	37:63 (−26)	91:9
2	(±)-**13a**	TBS	0.1 M	20	26 (38)	33:66 (−33)	56:44
3	(±)-**13b**	TBDPS	no internal standard	51	60	29:71 (−42)	94:6
4	(±)-**13c**	C(O)C_3_H_7_	no internal standard	168	53	33:67 (−34)	b
5	(±)-**13d**	CH_3_	-	45	15 (22)	42:58 (−16)	67:33
6	(±)-**13e**	BOM	no internal standard	140	33	40:60 (−20)	81:19
7	(±)-**13a**	TBS	(±)-**4** (30 mol %), 0.01 M	165	40 (26)	60:40 (20)	83:17
8	(±)-**13a**	TBS	[Rh(CO)_2_Cl]_2_ (10 mol %), CO:Ar (10:90, 1 atm), toluene (0.02 M) 110 °C, no internal standard	48	c	50:50 (0)	b
9	(±)-**13a**	TBS	[Rh(CO)_2_Cl]_2_ (40 mol %), CO:Ar (10:90, 1 atm), toluene-d_8_ (0.02 M), 110 °C, mesitylene (1 equiv)	73	d(7)	58:42 (16)	b

a Ratio based on ^1^H NMR of crude reaction mixture, all reactions were stopped when no SM remained.

b Trace aldehyde.

c The percent conversion is 9% based on the ratio of **13a**:**14a** (91:9) determined by the integration of the ^1^H NMR peaks at 6.59 and 6.39 ppm.

d The percent conversion is 17% based on the ratio of **13a**:**14a** (83:17).

While these results suggest that both the substrate and catalyst affect the diastereoselectivity of the PKR, the catalyst dictates which diastereomer is formed as the major product. For example, when triphenylphosphine was used as the ligand, the major product was (±)-*cis*-**14a** (entry 1), whereas with (±)-MonoPhos-alkene **4**, the *trans* isomer of product **14a** was favored (entry 7). Interestingly, when allene-yne **13a** is treated with neutral rhodium biscarbonyl chloride dimer [Rh(CO)_2_Cl]_2_ (0.05 equiv) and only CO is available as the ligand, the reaction stalls at 9% conversion, and no diastereoselectivity is observed (entry 8). However, when the catalyst loading was increased to 0.2 equiv under the same conditions, the reaction favored the formation of the *trans* isomer, if only slightly, yet still gave a low % conversion (entry 9). Thus, the dr values for the CO and (±)-MonoPhos-alkene ligands are similar, and the stereoselectivity for triphenylphosphine is the opposite of that of the (±)-MonoPhos-alkene ligand, with neither showing a strong preference for either the *cis* or *trans* product.

### Computed Transition States for the Stereo-Determining Oxidative Cyclization Step of PKR with **13d**

**Figure 3:**
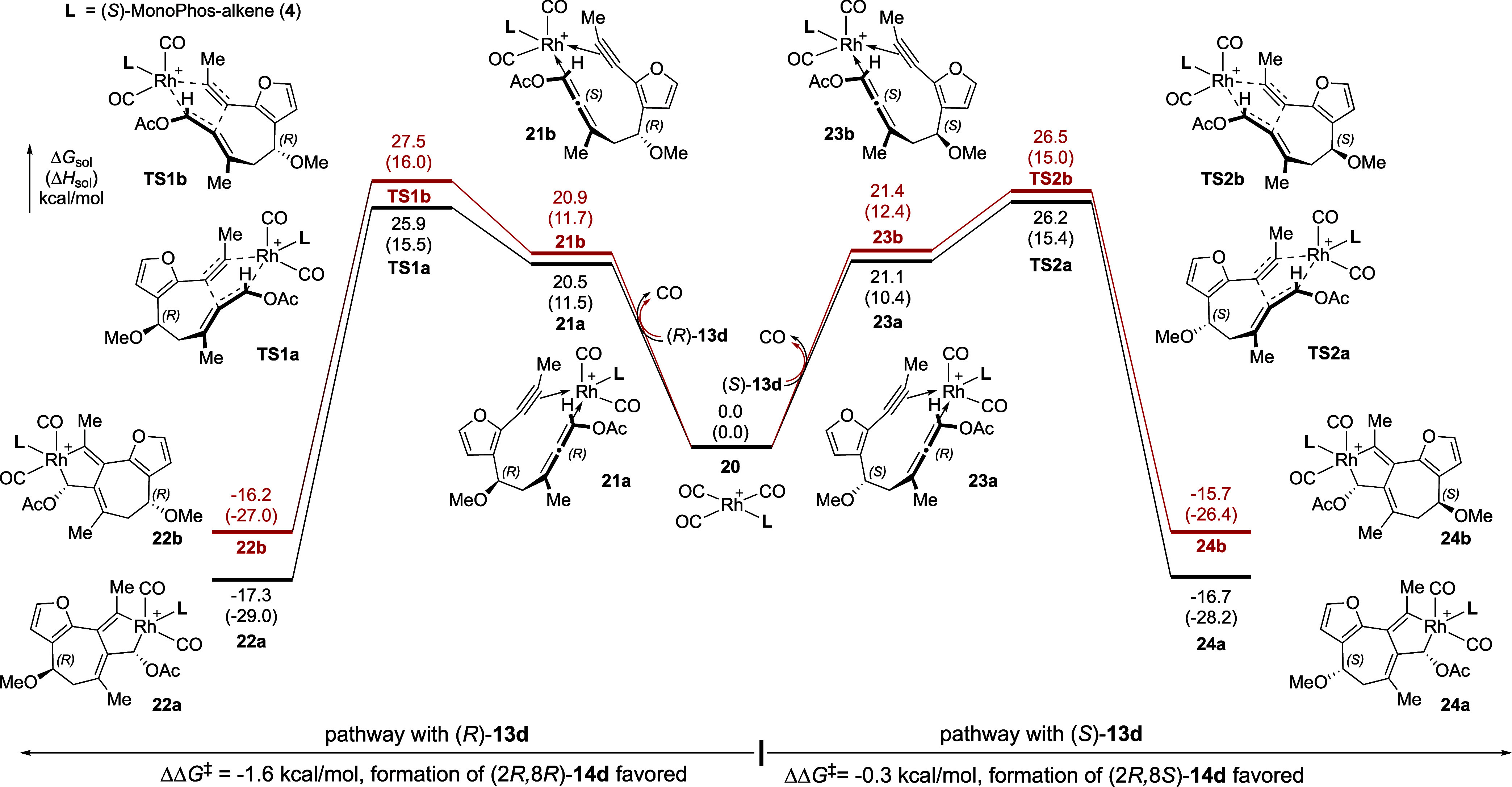
Computed reaction energy profiles for the oxidative cyclization of (*R*)- and (*S*)-**13d** with a (*S*)-MonoPhos-alkene (**4**)-supported Rh catalyst. DFT calculations were performed at the ωB97X-D/def2-TZVP/SMD(DCE)//B3LYP-D3/LANL2DZ-6-31G(d) level of theory.

As we have previously established oxidative cyclization as an irreversible and enantioselectivity-determining step in Rh(I)-catalyzed PKR of allene-ynes,^11,12^ we evaluated this step computationally (see computational SI for details of the DFT calculations) for the methoxy-substituted model compound **13d** to determine how the relative transition-state energies of this step might impact the overall reaction diastereoselectivity. Beginning with the phosphoramidite (*S*)-MonoPhos-alkene (**4**)-supported Rh(I) tricarbonyl complex (**20**), ligand exchange with either allene-yne (8*R*)-**13d** (left pathway, Figure 3) or (8*S*)-**13d** (right pathway) forms four different five-coordinate Rh complexes (**21a**–**b**, **23a**–**b**), each exhibiting a different prochiral π-face of the allene and the alkyne chelating to the Rh center. Although the substrate binding process is endergonic by 20–22 kcal/mol for both (8*R*)- and (8*S*)-**13d**, the equilibration to form allene-yne–rhodium complexes is promoted by the low concentration of dissolved CO.^11,12^ Subsequent oxidative cyclization (**TS1a/b** and **TS2a/b**) occurs via these five-coordinate complexes, as the alternative oxidative cyclization pathways (i.e., those involving four-coordinate allene-yne–rhodium complexes with ligand (*S*)-**4** and one CO ligand) are ∼10 kcal/mol less favorable (see Figure S7).^11,66^ Additionally, the oxidative cyclization of allene-yne **13d** with a rhodium tricarbonyl complex, in the absence of a phosphoramidite ligand, was found to be less favorable by ∼17 kcal/mol computationally (see Figure S8). For reactions with both (8*R*)- and (8*S*)-**13d**, oxidative cyclization favors Rh–C bond formation at the (*Si*)-face of the OAc-substituted allene terminus (**TS1a** and **TS2a**). This suggests that the PKR product **14d** should exhibit catalyst-controlled (*R*)-stereochemistry at C2, as the pathways leading to product (*S*)-**14d** are predicted to be 1.6 and 0.3 kcal/mol higher in energy (**TS1b** and **TS2b**, respectively). Formation of the Rh(III) metallocycles **22a**–**b** and **24a**–**b** are exergonic by 15–18 kcal/mol relative to complex **20**, consistent with previous findings that the oxidative cyclization is irreversible and stereo-determining.

### Rh(I)-Catalyzed Asymmetric PKR

We then sought to extend these predictions to the asymmetric PKR, focusing specifically on the impact of differing C8 functionality.^12^ Reaction of allene-yne (±)-**13a** with Rh(cod)_2_BF_4_ and ligand (*S*)-**4** in DCE under a CO atmosphere (100%)^67^ at 70 °C afforded PKR product **14a** in 33% yield and 20% de. High stereoselectivity was observed at C2 for the (8*R*)*-***14a** isomers (91.5:8.5), and moderate selectivity was observed for the (8*S*)*-***14a** isomers (82.4:17.6), as determined by chiral HPLC (Table 2, entry 1). Reaction of TBDPS-substituted allene-yne (±)-**13b** with ligand (*S*)-**4** mirrored these results, providing product **14b** in 44% yield (de = 24%) and ers of 92:8 and 76:24 (entry 6). The reaction of methoxy allene-yne **14d** with ligand (*S*)-**4** gave **14d** in 28% yield (de = 4%) and er values of 86.2:13.8 and 82.5:17.5. Similarly, the reaction of BOM-substituted allene-yne **14e** gave access to **14e** in 26% yield (de = 8%) and ers of 86:14 and 79:21. While the des for **14d** and **14e** were lower than those for the larger silyloxy groups, the ers were similar across the series (compare entries 1 and 6–8).

**Table 2:**
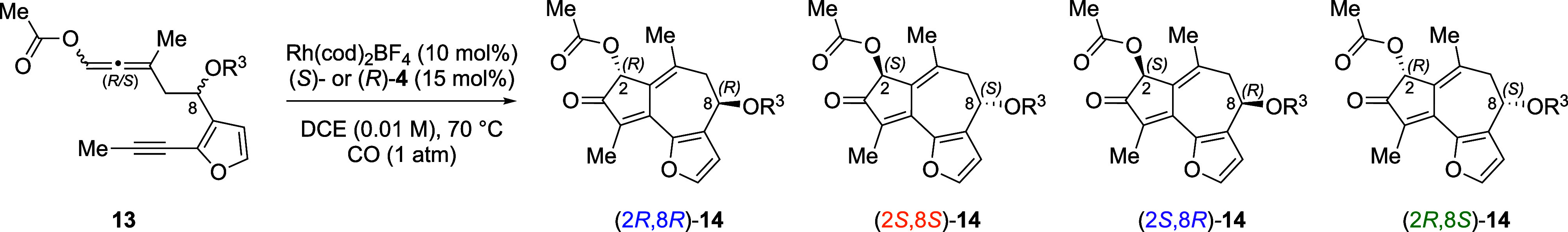

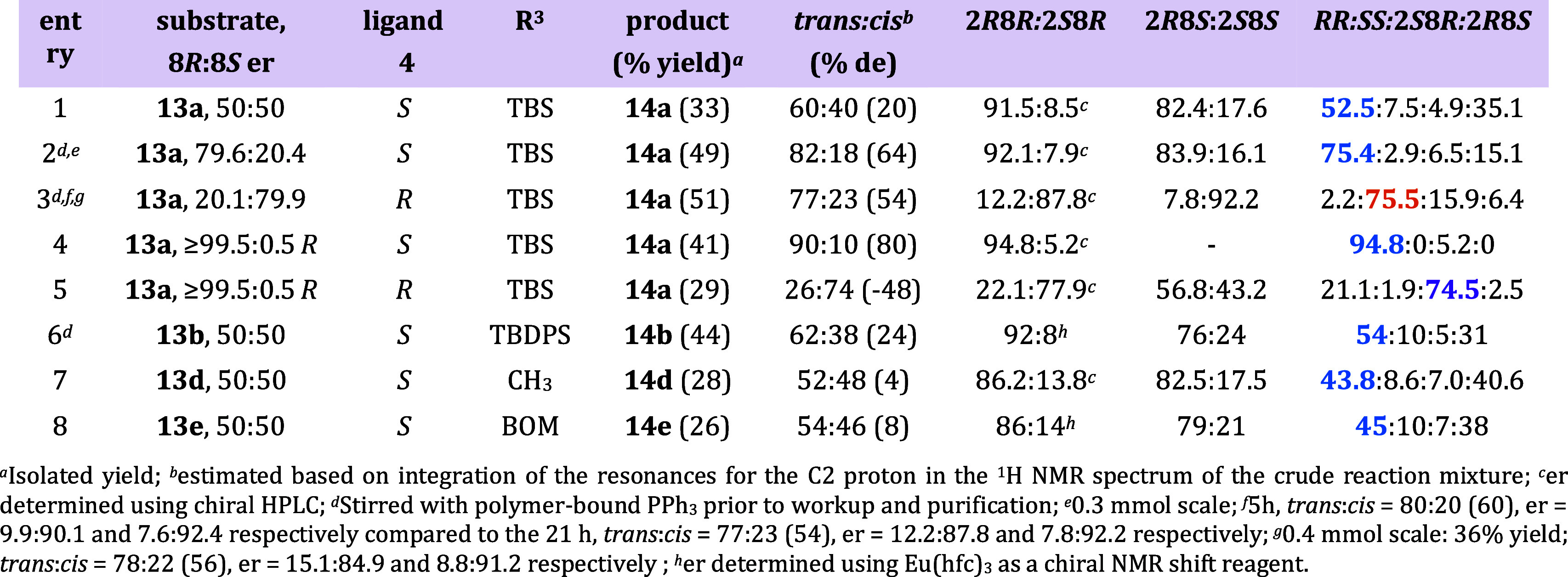
Asymmetric PKR on Allene-ynes **13**: Influence of the C8-Substituent and Chirality on the dr and er

In all cases, the (*R*) configuration was favored at C2 when using (*S*)-**4**, regardless of the C8 configuration, confirming our computational predications. In accord with these studies, (2*R*,8*R*)-**14a**, -**14b**, -**14d**, and -**14e** and (2*R*,8*S*)-**14a**, -**14b**, -**14d**, and -**14e**, arising from **TS1a** and **TS2a**, respectively, were observed as the major stereoisomers.

In order to obtain a reliable isolated yield, we next performed the reaction on a larger scale (0.3 mmol vs 0.05 mmol) using **13a** enriched in the (8*R*) isomer (59.2% ee). By treating the crude reaction mixture with polymer-bound PPh_3_ to remove the Rh catalyst prior to workup and purification, product **14a** could be isolated in 49% yield and 64% de (entry 2). Allene-yne (8*R*)-**13a** provided (8*R*)-**14a** in 92.1:7.9 er, as determined by chiral HPLC, while (8*S*)-**13a** gave the isomer (8*S*)-**14a** in 83.9:16.1 er (entry 2). Similarly, the reaction of enantioenriched (*S*)-**13a** (59.8% ee) with (*R*)-**4** afforded **14a** in 51% yield, 54% de, and ers of 12.2:87.8 and 7.8:92.2, favoring the (2*S*,8*S*) stereoisomer (entry 3). As thapsigargin possesses the same (2*S*,8*S*)-stereochemical pattern at C2 and C8, this stereoisomer is of particular interest. We attribute the differences in stereoselectivity to matched/mismatched interactions between the catalyst and substrate commensurate with the higher computed ΔΔ*G*^‡^ value for (8*R*)-**13d** (1.6 kcal/mol) compared with that for (8*S*)-**13d** (0.3 kcal/mol), see the discussion below.

When enantiopure allene-yne (8*R*)-**13a** was treated with matched ligand (*S*)-**4** under otherwise standard reaction conditions, product **14a** was formed in 41% yield, 80% de, and 94.8:5.2 er (entry 4). In the mismatched case, enantiopure (8*R*)-**13a** was reacted with ligand (*R*)-**4** to afford **14a** in a lower yield and selectivity (29% yield, −48% de, and 22.1:77.9 er, entry 5). Under mismatched conditions, small amounts of the enantiomeric products were observed, possibly due to C8 epimerization under the Rh(I) reaction conditions, along with larger amounts of the aldehyde side product (**14a**:**S8**, 67:33 (entry 4) vs 50:50 (entry 5).

While this asymmetric dynamic kinetic PKR is operating under the predominantly catalyst control, we do observe the weak substrate control as evidenced by increased diastereomeric excess, in favor of the *trans* isomer, as the C8 group increases in size (OMe < OBOM < OTBS < OTBDPS) (Table 2, entries 1 and 6–8). As transition-state structures **TS1b** and **TS2a** place the C8-substituent proximal to the chiral ligand, we surmise that these transition states leading to the *cis-*products would be increasingly destabilized as the substituent at C8 becomes larger.

### Matched and Mismatched Pairs

The higher C2 stereocontrol observed for (8*R*) over (8*S*) in the PKR of **13a**, **13b**, **13e**, and **13f** with (*S*)-**4** suggests the presence of a matched and mismatched case between the substrate and the ligand (Table 2, entries 1, 2, 4, 6, and 8 and Table S3, entries 2 and 4). Similarly, when ligand (*R*)-**4** is utilized, the situation inverts, providing higher C2 stereoselectivity for (8*S*). Again, the more moderate C2 stereocontrol for (8*R*) suggests a mismatched case (entries 3 and 5). Therefore, (8*R*)-**13**/(*S*)-**4** and (8*S*)-**13**/(*R*)-**4** are matched pairs, and (8*S*)-**13**/(*S*)-**4** and (8*R*)-**13**/(*R*)-**4** are mismatched pairs. In the case of methyl-substituted allene-yne **13d**, moderate stereoselectivity was observed for both matched and mismatched pairs, with slightly higher stereoselectivity being observed for the matched pairs (entry 7). In this way, the matched and mismatched cases can provide access to all four stereoisomers of product **14** in synthetically useful selectivity (for additional examples see Table S3, Entries 1–5 and Figure S1).

From the computed energy profiles of the oxidative cyclization with the (*S*)-**4**-supported Rh catalyst (Figure 3), the diastereoselectivity with allene-yne (8*R*)-**13d** (ΔΔ*G*^‡^ = 1.6 kcal/mol) is higher than that with (8*S*)-**13d** (ΔΔ*G*^‡^ = 0.3 kcal/mol), which agrees with the experimentally observed higher stereoselectivity with enantioenriched (8*R*)-allene starting materials (Table 2, entries 4 and 5). To better understand the origins of stereoselectivity and the matched and mismatched pairs in the asymmetric PKR, we calculated distortion and through-space interaction energies and then used NCIPlot to visualize the noncovalent interactions between the phosphoramidite ligand and allene-yne in the oxidative cyclization transition states (Figures S5 and S6, see computational Supporting Information for details). In particular, we sought to reveal what factors favor the formation of the (2*R*) stereocenter with both (8*R*)- and (8*S*)-**13d** and the higher diastereoselectivity with (8*R*)-**13d**. We calculated the overall through-space interactions between the MonoPhos-alkene ligand (*S*)-**4** with the full allene-yne substrate (Δ*E*_int-space_) at the transition states, as well as with the furan and ether fragments of the substrate (Δ*E*_int-space(furan)_ and Δ*E*_int-space(ether)_, see computational Supporting Information for details). In addition, we calculated the distortion energies of the allene-yne substrate (Δ*E*_dist(substrate)_) and the phosphoramidite ligand (Δ*E*_dist(ligand)_) at the transition states with respect to their ground-state geometries (i.e., free substrate and free ligand).

**Figure 4:**
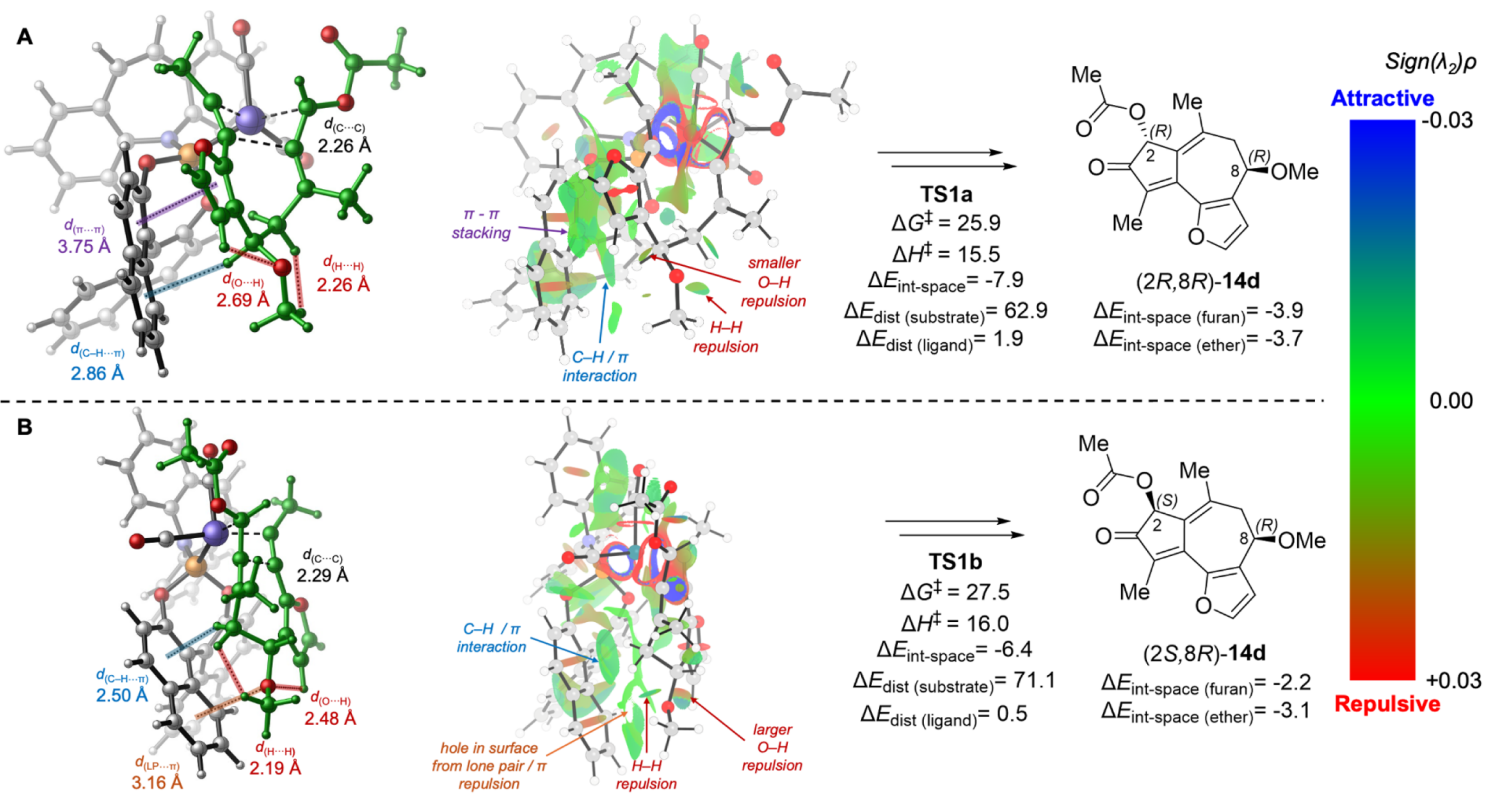
Lowest energy oxidative cyclization transition-state isomers with (*R*)-**13d** (matched pairs). All energies are in kcal/mol with respect to **20**. See Supporting Information for computational details, including distortion and through-space interaction energy calculations.

Between the two oxidative cyclization transition states with (8*R*)-**13d** (**TS1a** and **TS1b**, Figure 4), the major difference is the orientation of the C8-OMe and furanyl substituents of the allene-yne substrate. In the lower energy transition state **TS1a**, the furanyl group points toward the phosphoramidite ligand, whereas C8-OMe is pointing away from the ligand. By contrast, in the less stable **TS1b**, where the Rh catalyst approaches from the opposite face of the allene-yne substrate, C8-OMe is pointing toward the phosphoramidite ligand and the furanyl group is pointing further away. This causes major differences in the ligand–substrate noncovalent interactions as well as the distortion of the allene-yne substrate itself. The overall through-space interaction energies (Δ*E*_int-space_ = −7.9 and −6.4 kcal/mol for **TS1a** and **TS1b**, respectively) indicate that the noncovalent ligand–substrate interaction in **TS1a** is 1.5 kcal/mol more favorable than that in **TS1b**. **TS1a** is stabilized by a π–π stacking interaction between the furanyl group and the biaryl group of the phosphoramidite ligand (Δ*E*_int-space(furan)_ = −3.9 kcal/mol) as well as a C–H/π interaction between the inductively polarized C8–H and the ligand (Δ*E*_int-space(ether)_ = −3.7 kcal/mol). On the other hand, in **TS1b**, the furanyl group has a weaker noncovalent interaction with the ligand due to the longer distance (Δ*E*_int-space(furan)_ = −2.2 kcal/mol). In addition, because the C8-OMe group is pointing toward the biaryl group on the ligand, a lone pair/π repulsion is observed, leading to overall weaker noncovalent interaction between the ligand and the ether fragment on the substrate (Δ*E*_int-space(ether)_ = −3.1 kcal/mol in **TS1b** compared with −3.7 kcal/mol in **TS1a**). This lone pair/π repulsion with C8-OMe also leads to greater substrate distortion (Δ*E*_dist(substrate)_) in **TS1b**, which is evidenced by eclipsing repulsions of C8-OMe with both furanyl C–H (*d*_(O···H)_ = 2.48 Å) and C9–H (*d*_(H···H)_ = 2.19 Å). It should be noted that the relative energies between **TS1a** and **TS1b** are also affected, likely to a lesser degree, by several other factors, including a stronger stabilizing through-bond interaction between the Rh and the allene moiety in **TS1b** and pseudo-1,3-diaxial interactions between C8-OMe and C10-Me groups in the half-chair-like transition state **TS1a**. More detailed analyses of these interactions are provided in Supporting Information.

**Figure 5:**
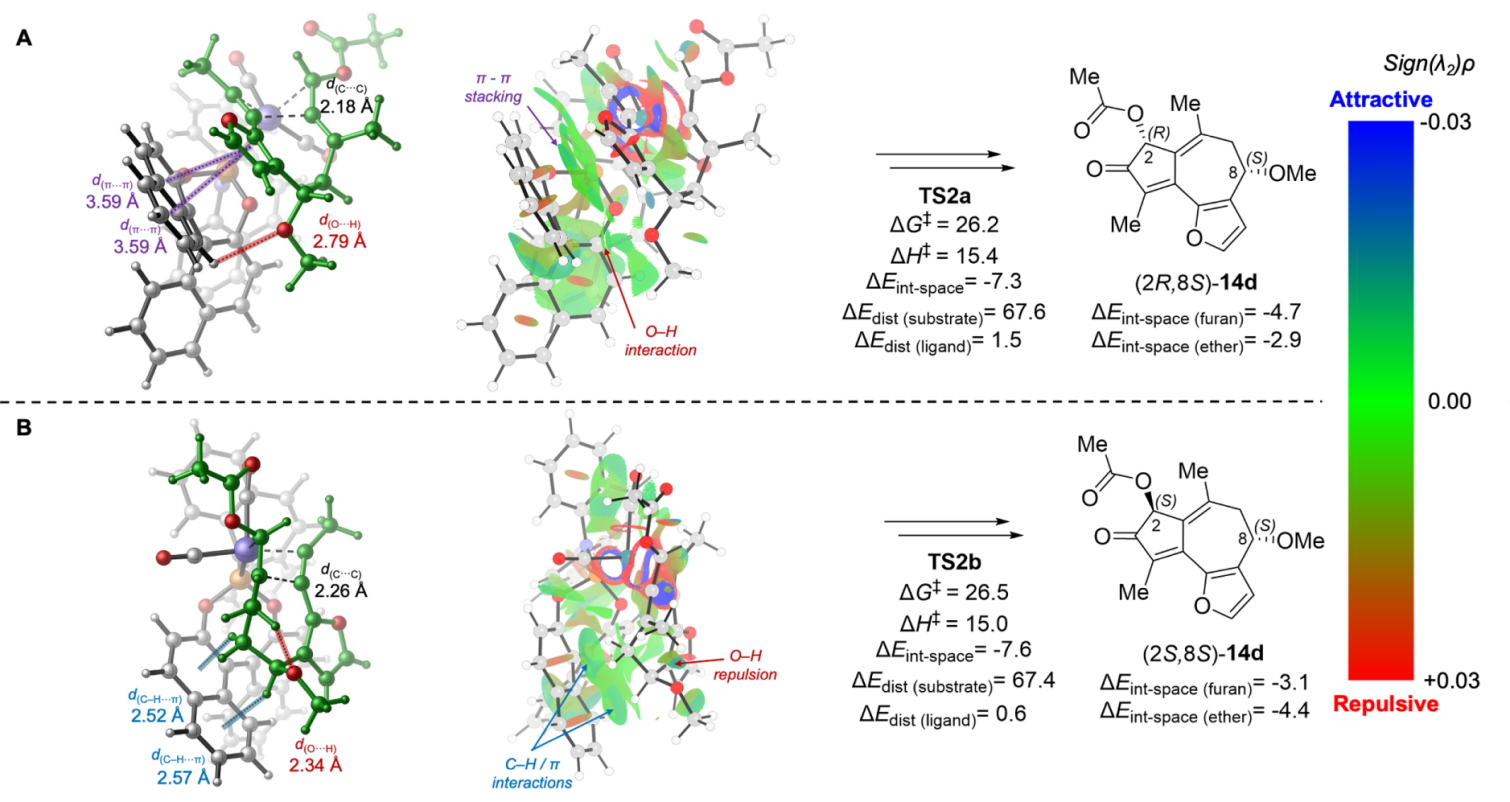
Lowest energy oxidative cyclization transition-state isomers with (*S*)-**13d** (mismatched pairs). All energies are in kcal/mol with respect to **20**. See Supporting Information for computational details, including distortion and through-space interaction energy calculations.

Next, we analyzed the lowest energy transition states in the mismatched reactions of (*S*)-**13d** with the (*S*)-**4** ligand (Figure 5). In **TS2a** that leads to the slightly favored (2*R*) stereocenter, both the furanyl and the C8-OMe groups on (*S*)-**13d** point toward the biaryl group of the phosphoramidite ligand, whereas in **TS2b**, both furanyl and C8-OMe are further away from the ligand. Therefore, **TS2a** is stabilized by π–π stacking and C–H/π interaction of the furanyl group with the ligand (Δ*E*_int-space(furan)_ = −4.7 kcal/mol), while also destabilized by the steric repulsion between the C8-OMe group and the ligand (*d*_(O···H)_ = 2.79 Å), resulting in a weaker through-space interaction with the ether moiety (Δ*E*_int-space(ether)_ = −2.9 kcal/mol). On the other hand, in **TS2b**, although the noncovalent interaction with the furanyl group is weaker (Δ*E*_int-space(furan)_ = −3.1 kcal/mol), the interaction with the ether moiety is stronger (Δ*E*_int-space(ether)_ = −4.4 kcal/mol) due to the favorable C–H/π interaction with C8–H. As a result, the overall through-space ligand–substrate interactions in **TS2a** and **TS2b** are comparable (Δ*E*_int-space_ = −7.3 and −7.6 kcal/mol, respectively). The relatively small activation free energy difference in this mismatched case (ΔΔ*G*^‡^ = 0.3 kcal/mol) can be mostly attributed to the pseudo-1,3-diaxial interaction between C8-OMe and C10-Me in the half-chair-like transition state **TS2b**. This is evidenced by a short O···H distance (2.34 Å) between the methoxy oxygen and C10-Me.

Taken together, the DFT calculations demonstrate that the orientations of the furanyl and C8-OMe groups in the oxidative cyclization transition states both affect the transition-state energies. While the former is affected by the π-facial selectivity of the allene when coordinating to the Rh catalyst, the latter is affected by the absolute configuration of the C8 stereogenic center. The calculations suggest that the dominant factor determining the stereoselectivity (ΔΔ*G*^‡^) is the catalyst-controlled π-facial selectivity that always prefers to produce (2*R*)-PKR products with the (*S*)-**4** ligand, regardless of the absolute configuration of C8 in the allene-yne substrate. On the other hand, the stereochemistry at C8 alters the magnitudes of the product diastereomeric ratio by affecting both noncovalent interactions between the C8-substituent and the chiral phosphoramidite ligand and intramolecular substrate distortions (Figures 4 and 5).

### Absence of Kinetic Resolution

**Figure 6:**
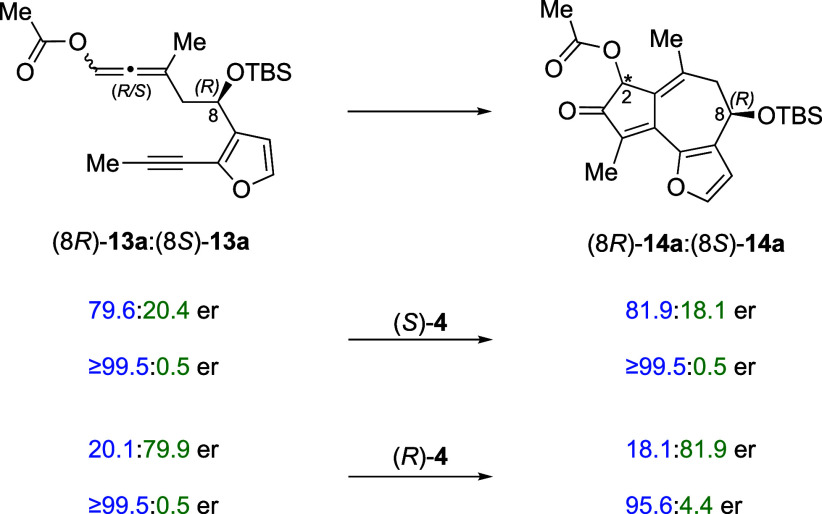
Absence of kinetic resolution in PKR of allene-yne **13a**.

Monitoring the PKR reaction by chiral HPLC shows that there is no significant change in either de or er of the product throughout the reaction (5 h and 21 H). These data demonstrate that (8*R*)- and (8*S*)-**13a** react at the same rate (Table 2, entry 3). Moreover, comparison of the er of allene-yne **13a** to that of **14a** showed negligible or no change in the C8 er, providing further support that the observed asymmetric PKR is not a kinetic resolution process (Figure 6). For example, in the matched cases, enantioenriched **13a** with C8 ers of 79.6:20.4 and ≥99.5:0.5 yielded **14a** with C8 ers of 81.9:18.1 and ≥99.5:0.5. In the mismatched cases, enantioenriched **13a** with C8 ers of 20.1:79.9 and ≥99.5:0.5 yielded **14a** with C8 ers of 18.1:81.9 and 95.6:4.4.

### Assignment of the Relative Configuration in Product **14a**

To assign the relative stereochemistry of product **14a**, the *cis* and *trans* diastereomers were analyzed together by ^13^C NMR, assigning the peaks via peak intensity differences. ^13^C NMR chemical shifts for each were computed using a protocol for calculating Boltzmann-weighted chemical shifts for conformationally flexible natural products with Spartan’24 (ωB97X-D/6-31G*).^68^ Comparison of calculated chemical shifts of the *trans* diastereomer to authentic spectral data for the major isomer showed a good match, with a Boltzmann average DP4 score of 100% and an RMS of 1.72. The minor *cis* diastereomer matched equally well, exhibiting a Boltzmann average DP4 score of 100% and an RMS of 1.35 (see SI for ^13^C NMR analysis).^69^

### Assignment of the Absolute and Relative Configuration of **14g**

To assign the absolute configuration of **14a**, the silyl group was removed upon treatment with TBAF/AcOH at −10 °C (condition a).^70^ The *cis* and *trans* isomers of **14g** were then separated by preparative TLC^71^ to afford (2*S*,8*S*)-**14g** (er = 94.7:5.3) and (2*R*,8*S*)-**14g** (er = 47.7:52.3) (Table 3, entry 1). TBS deprotection of (2*R*,8*R*)-**14a** gave the *cis* and *trans* isomers of **14g**, which could be separated by preparative TLC ((2*R*,8*R*)-**14g**: er = 91.0:9.0; (2*S*,8*R*)-**14g**: er = 70.5:29.5) (entry 2). C2 epimerization was observed when deprotection was conducted with TBAF in the absence of AcOH (condition b),^72^ leading to an er switch for *cis*-**14** (entry 3).

**Figure 7:**
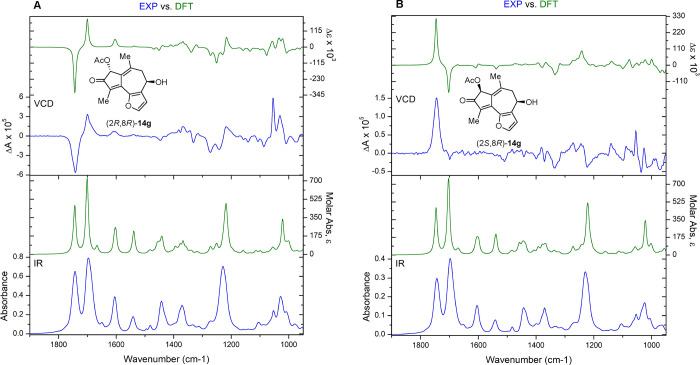
DFT-calculated (green) and experimental (blue) VCD and IR spectra of (A) (2*R,*8*R*)-**14g;** and (B) (2*S,*8*R*)-**14g**.

With three of the four stereoisomers in hand, we turned to VCD to assign the relative and absolute stereochemistry. The experimental IR and VCD data (blue) for (2*S*,8*S*)-**14g** and (2*R*,8*R*)-**14g** were independently measured, compared to DFT (B3LYP/cc-pVTZ)-calculated IR and VCD spectra (green), and quantified using CompareVOA software (BioTools, Jupiter FL). In all cases, high *S*_fg_ (overlap) values were observed, providing configuration assignments with a 100% confidence level (Figures 7A, S12–S15, S18, and S19). The VCD spectra for (2*R,*8*S)*-**14g** exhibit a low signal-to-noise ratio, due to a small amount of material (2 mg); however, computed VCD (B3LYP/cc-pVTZ) showed an excellent correlation to the experiment (Figures 7B, S16, S17, and S20). A Cai•factor of 68 was measured for (2*R,*8*R*)-**14g** showing a very confident assignment (Figure S21).^60^

**Table 3:**
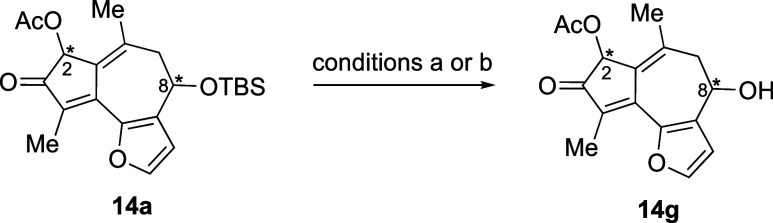
Separation of Product **14a** Diastereomers^a^

**entry**	*SS*:*RRtrans-***14a**	*SR*:*RScis***-14a**	**condition,% yield**	*SS*:*RRtrans-***14f**	*SR*:*RScis-***14f**
1	96.8:3.2	65.9:34.1	a, 44	94.7:5.3	47.7:52.3
2	3.7:96.3	30.1:69.9	a, 56	9.0:91.0	70.5:29.5
3	96.7:3.3	63.4:36.6	b, 29	83.2:16.8	16.4:83.6

a Conditions a: TBAF/AcOH, THF, −10 °C; b: TBAF, THF, 0 °C to rt.

## Conclusion

We report the first example of a catalyst-controlled dynamic kinetic asymmetric PKR, which yields the full stereochemical array of C2 and C8 thapsigargin analogues via a nonchiral pool starting material. This approach utilizes a preset stereocenter to influence catalyst binding in a way that can be predicted by a DFT transition-state analysis. The high stereoselectivity observed for both matched and mismatched cases was critical to our success. Further, VCD and ^13^C NMR strategies, supported by DFT calculations, allowed for the assignment of absolute configuration for all four stereoisomers. Our computational studies show that the catalyst-controlled stereoselectivity results from differences in substrate–ligand noncovalent interactions in the oxidative cyclization transition state, whereas the substrate C8 stereochemistry alters the magnitude of the diastereoselectivity by affecting substrate distortion as well as substrate–ligand noncovalent interactions. Together, these efforts have allowed us to expand the scope of dynamic kinetic asymmetric PKR to allene-ynes with a preset stereocenter. With this advance, we have solidified the robust nature of our catalyst-ligand system and placed the total synthesis of thapsigargin and its stereoisomeric analogs within reach.

## Supporting Information Available

Detailed experimental protocols, characterization data, ^1^H/^13^C/2D NMR spectra, ^1^H NMR with chiral shift reagent spectra, ^13^C NMR data (experimental and computational), HPLC traces, VCD data (experimental and computational), supplementary figures (PDF)
Computational details and optimized geometries (PDF)
SI ZIP file (ZIP)
