# Supplementary material for: Stereo-Differentiating Asymmetric Rh(I)-Catalyzed Pauson–Khand Reaction: A DFT-Informed Approach to Thapsigargin Stereoisomers
Source: J Am Chem Soc. 2024 Dec 20;147(1):498–509. doi: 10.1021/jacs.4c11661 (PMC11726561; doi:10.1021/jacs.4c11661)
Supplement: Supplementary file 1 — ja4c11661_si_001.pdf [file ja4c11661_si_001.pdf]

Supporting Information for Experimental Studies

Stereo-Differentiating Asymmetric Rh(I)-Catalyzed Pauson–Khand Reaction: A DFT-Informed Approach to Thapsigargin Stereoisomers.

Fatemeh Haghighi,[a] Luke T. Jesikiewicz,[a] Corrinne E. Stahl,[a] Jordan Nafie,[b] Amanda Ortega-Vega,[a] Peng Liu,*[a] and Kay M. Brummond*[a]

[a] Department of Chemistry, University of Pittsburgh, Pittsburgh, Pennsylvania 15260, United States

[b] BioTools, Inc., Jupiter, Florida 33478, United States

Corresponding authors: pengliu@pitt.edu; kbrummon@pitt.edu

Table of Contents

General Methods……………………………………………………………………………S-2

Synthesis of **S1** and **16**……………………………………………………………………S-3

Non-stereoselective and asymmetric aldol affording **17** and **S2**……………………………S-5

Synthesis of compounds **18a-e**, **19a-f**, and **13a-f**………………………………………S-17

Racemic and asymmetric PKR affording **14a-f**………………………………………………S-54

Separation of **14a** diastereomers……………………………………………………………S-110

Aldehyde byproduct **S8**………………………………………………………………………S-121

Reaction optimization of asymmetric aldol (proline)…………………………………………S-124

Reaction optimization of asymmetric aldol (β-tosylamidoprolineamide)……………………S-126

Attempts to form derivatives of **17** (**S13** and **S15**) to get crystals for the assignment of absolute configuration……………………………………………………………………………S-128

Asymmetric PKR of **13f** to form **14f**, selective synthesis of stereoisomers.........................S-133

Matched and mismatched cases in PKR of allene-yne **13f**..S-134

COSY, HMBC, and HSQC Spectra of **14a**..S-137

^{1}H and ^{13}C NMR Spectral data..S-143

References...S-210

Table S1. Reaction optimization of asymmetric aldol (proline) S-125

Table S2. Reaction optimization of asymmetric aldol (*β*-tosylamidoprolineamide)................S-126

Table S3. Asymmetric PKR of **13f** to form **14f**, selective synthesis of stereoisomers..........S-134

Figure S1. Matched and mismatched cases in PKR of allene-yne **13f**..........................S-135

Figure S2. COSY and HMBC correlations of **14a**...S-136

General Methods

All commercially available compounds were purchased and used as received unless otherwise noted. Tetrahydrofuran (THF), diethyl ether (Et_2O), and dichloromethane (DCM, CH_2Cl_2) were purified by passing through alumina using a Sol-Tek ST-002 solvent purification system. Toluene and triethylamine (Et_3N) were purified by distilling from calcium hydride. Acetone was purified by refluxing over and distillation from $KMnO_4$ and stored over 4Å molecular sieves. Triphenylphosphine was purified by recrystallization with ethanol. Carbon monoxide (>99.99%) and 10% carbon monoxide in argon (>99.99%) were purchased from Matheson. Purification of the compounds by flash column chromatography was performed using silica gel (40-63 µm particle size, 60 Å pore size). Purification of the compounds by Prep TLC was performed on silica gel

F254 glass plates (250 μm thickness). TLC analyses were performed on silica gel F254 glass plates (250 μm thickness). ^{1}H and ^{13}C NMR spectra were recorded on Bruker Avance 300, 400, 500, or 700 MHz spectrometers. Spectra were referenced to residual chloroform (7.26 ppm, ^{1}H NMR; 77.16 ppm, ^{13}C NMR). Chemical shifts are reported in ppm; multiplicities are indicated by s (singlet), br s (broad singlet), d (doublet), t (triplet), q (quartet), p (pentet), m (multiplet), and dd (doublet of doublets). Coupling constants, *J*, are reported in hertz (Hz). All NMR spectra were obtained at rt. Enantiomeric ratios determined by NMR were performed using $Eu(hfc)_3$ as a chiral shift reagent. IR spectra were obtained using a Perkin Elmer Spectrum Two FT-IR. ESI mass spectrometry was performed on a Waters Micromass GCT high resolution mass spectrometer, while ES mass spectrometry was performed on a Waters Q-TOF Ultima API, Micromass UK Limited high resolution mass spectrometer. Enantiomeric ratios determined by HPLC were performed using a Shimadzu Nexera Series SCL-40 ultra high performance liquid chromatography. Optical rotations were measured using a Jasco P2000 polarimeter. For compound naming in the SI, ChemDraw was used. For labeling of the stereocenters in the manuscript, sesquiterpene lactone numbering was used.

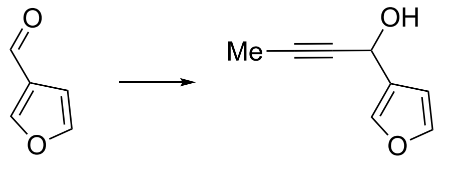

15 S1

1-(furan-3-yl)but-2-yn-1-ol (S1).

This compound was prepared in a manner similar to that previously reported.[1] A flame-dried, 3-necked, 250-mL round-bottomed flask equipped with a magnetic stir bar, addition funnel, nitrogen

inlet adapter, and septum was charged with 3-furfural **15** (1.9 mL, 22 mmol) and THF (22 mL). The flask was placed in an ice-bath. Propynyl magnesium bromide (78 mL of a 0.5 M solution in THF, 40 mmol) was transferred to the addition funnel via cannula and added dropwise to the reaction mixture. After 2 h at 0 °C, TLC showed complete consumption of starting material. Saturated aqueous ammonium chloride (35 mL) was added all at once and stirred for 15 min. The reaction mixture was transferred to a separatory funnel. The aqueous phase was extracted with diethyl ether (2 x 80 mL), dried over magnesium sulfate, filtered, and concentrated *in vacuo*. The crude residue was purified by running through a short plug of silica gel (20% ethyl acetate/hexanes) to afford 3.0 g (99% yield) of the title compound as a light-yellow oil.

^{1}H NMR (400 MHz, $CDCl_3$)

7.51 (s, 1 H), 7.40–7.39 (m, 1 H), 6.51–6.50 (m, 1 H), 5.36 (s, 1 H), 2.06–2.00 (m, 1 H), 1.90 (d, J = 2 Hz, 3 H) ppm

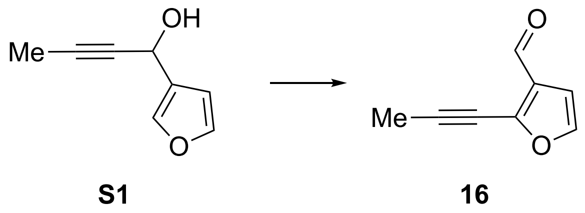

2-(prop-1-yn-1-yl)furan-3-carbaldehyde (16).

This compound was prepared in a manner similar to that previously reported.[1] A flame-dried, 2-necked, 250-mL round-bottomed flask equipped with a magnetic stir bar, nitrogen inlet adapter, and septum was charged with 1-(furan-3-yl)but-2-yn-1-ol **S1** (2.96 g, 21.7 mmol), THF (87 mL, 0.25 M), and water (21.7 mL). *N*-bromosuccinimide (3.86 g, 21.7 mmol) was added in two portions

about 10 min apart by temporary removal of the septum. After 5 h, TLC showed complete consumption of starting material. 1 M HCl (23 mL) was slowly added to the solution and stirred for 10 min. The solution was transferred to a separatory funnel, and the aqueous layer was extracted with diethyl ether (3 x 80 mL). The combined organic layers were washed with brine (1 x 60 mL), dried over magnesium sulfate, filtered, and concentrated *in vacuo*. The crude residue was purified by silica gel flash chromatography (15% ethyl acetate/hexanes) to afford 2.15 g (74% yield) of the title compound as a brown liquid that solidified to a sticky brown solid upon freezing.

^{1}H NMR (400 MHz, $CDCl_3$)

9.98 (s, 1 H), 7.32 (d, J = 2.0 Hz, 1 H), 6.73 (d, J = 2.0 Hz, 1 H), 2.19 (s, 3 H) ppm

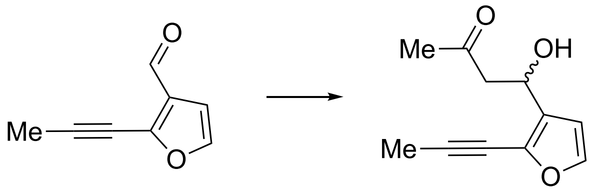

Synthesis of β-hydroxy ketone via a non-stereoselective aldol addition with acetone

(±)-4-hydroxy-4-(2-(prop-1-yn-1-yl)furan-3-yl)butan-2-one ((±)-17).

This compound was prepared in a manner similar to that previously reported, with some modifications.[2] A flame-dried, 2-necked, 5-mL, pear-shaped flask equipped with a magnetic stir bar was charged with 2-(prop-1-yn-1-yl)furan-3-carbaldehyde **16** (1 equiv, 134 mg, 1.0 mmol) and acetone (23 equiv, 1.7 mL, 23.0 mmol). The flask was lowered in a precooled CryoCool bath (-10 °C). A 2% NaOH solution in water w/w (0.2 mL) was added dropwise. After 2 h, TLC showed complete consumption of starting material. Next, 0.5 M HCl was added dropwise until the pH

paper showed 5. The solution was transferred to a 20 mL vial and concentrated in a vacuum using rotary evaporation. Deionized (DI) water (9 mL) was added to the vial, transferred to a separatory funnel, and extracted with EtOAc (5 x 5 mL). The combined organic layers were dried over magnesium sulfate, filtered, and concentrated *in vacuo*. The crude residue was purified by silica gel flash chromatography (gradient 30-50% ethyl acetate/hexanes) to give 146 mg (73% yield) of the title compound as a yellow oil.

^{1}H NMR (500 MHz, $CDCl_3$)

7.26 (s, 1 H), 6.44 (d, *J* = 2.0 Hz, 1 H), 5.20 (dd, *J* = 9.0, 3.0 Hz, 1 H), 3.20 (brs, 1 H), 2.95 (dd, *J*= 17.5, 9.5 Hz, 1 H), 2.81 (dd, *J*= 17.5, 3.0 Hz, 1 H), 2.22 (s, 3 H), 2.11 (s, 3 H) ppm

^{13}C NMR (125 MHz, $CDCl_3$)

209.1, 142.9, 134.1, 130.0, 109.5, 94.0, 69.0, 62.9, 50.2, 30.8, 4.8 ppm

IR (Thin Film)

3414, 2919, 2234, 1706, 1233 cm^{-1}

HRMS HRMS-ESI (m/z): $[M + H]^+$ calcd for $C_{11}H_{11}O_3$, 191.0703; found, 191.0705

TLC R_f = 0.14 (35% ethyl acetate/hexanes); silica gel, UV, *p*-anisaldehyde, $KMnO_4$

Shimadzu Nexera Series SCL-40, UV/PDA detector, 254 nm, CHIRALPAK IB N-3, 150 X 4.6 mm column, 10% *i*PrOH/hexanes, Flow rate: 1 mL/min

Peak	Retention Time (min)	Peak area (%)
Peak 1	5.73	49.93
Peak 2	6.44	50.07

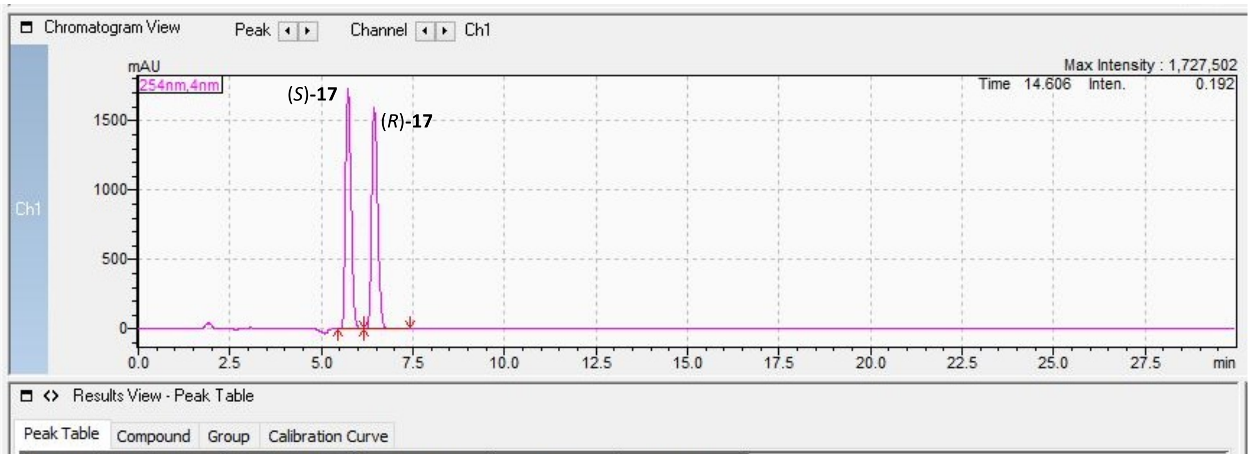

Peak#	Ret. Time	Area	Height	Mark	Area%
1	5.726	18260705	1726749	M	49.930
2	6.443	18311582	1600412	M	50.070
Total		36572286	3327161		100.000

General procedure A: Determination of enantiomeric ratios via NMR using chiral shift reagent

Chiral shift reagent europium tris[3-(heptafluoropropylhydroxymethylene)-(+)-camphorate ($Eu(hfc)_3$, 10 mg) was dissolved in $CDCl_3$ (0.1 mL) and added to an NMR tube containing (±)-**17** in $CDCl_3$. Gradual addition of aliquots of the preprepared $Eu(hfc)_3/CDCl_3$ solution to (±)-**17** in $CDCl_3$ showed the resonance at 6.44 ppm (proton at C4 of furan) splitting into two resonances at 6.80, and 6.77 ppm.

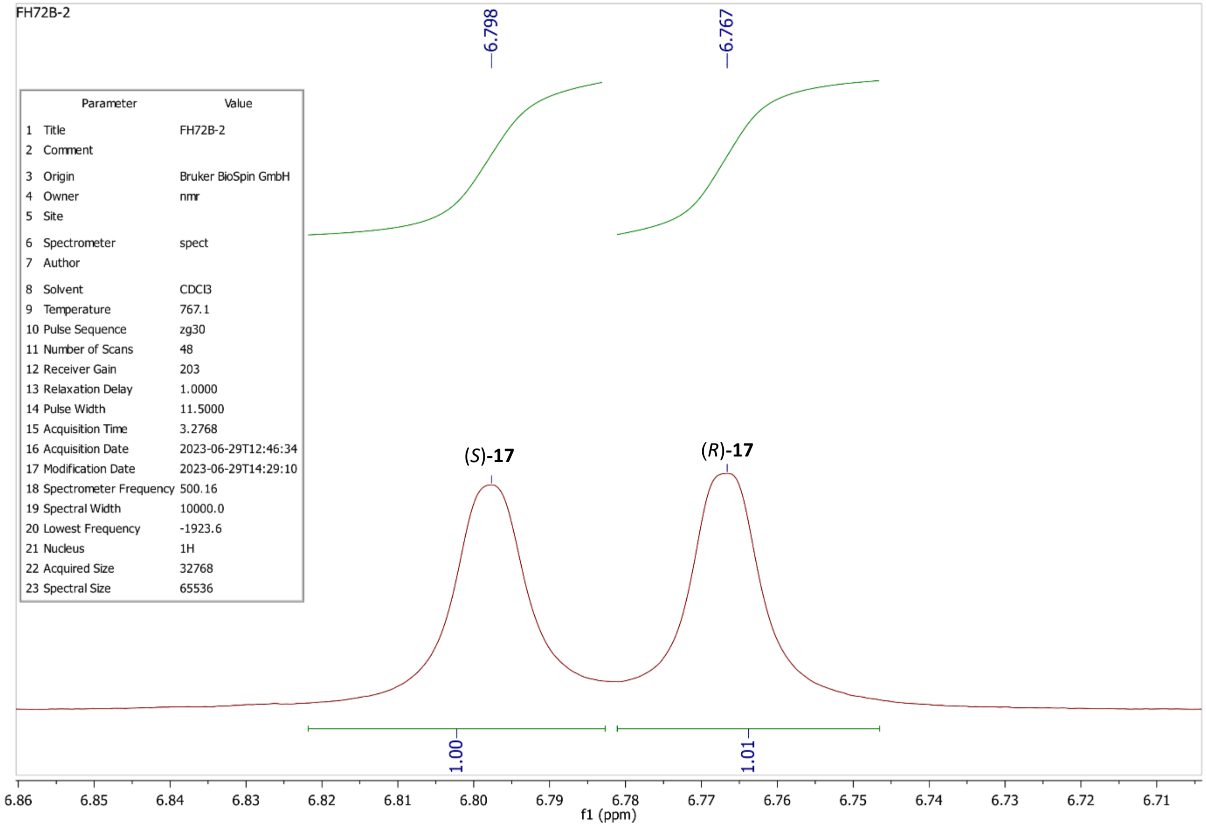

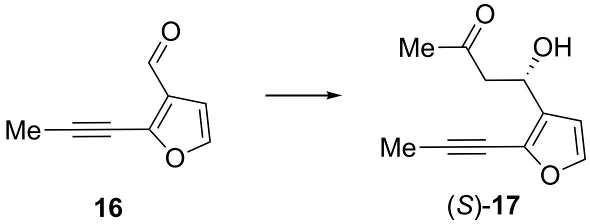

General procedure B: Synthesis of *β*-hydroxy ketone via an asymmetric aldol addition with acetone (proline)

(*S*)-4-hydroxy-4-(2-(prop-1-yn-1-yl)furan-3-yl)butan-2-one ((*S*)-17).

This compound was prepared in a manner similar to that previously reported, with some modifications.[3] A flame-dried, 50-mL, Schlenk tube equipped with a magnetic stir bar was charged

with (*S*)-BINOL (0.02 equiv, 19 mg, 0.07 mmol), D-proline (0.3 equiv, 114 mg, 1.0 mmol), acetone (25 equiv, 6.1 mL, 82.5 mmol), DMSO (6 equiv, 1.3 mL, 18.8 mmol). The Schelenk tube was placed in an ice-bath. After 30 min, 2-(prop-1-yn-1-yl)furan-3-carbaldehyde **16** (1 equiv, 446 mg, 3.3 mmol) was added. The Schelenk tube was placed in a precooled Cryotrol bath (0 °C). After 354 h, TLC showed a trace amount of starting material. Saturated aqueous ammonium chloride (15 mL) was added to the Schelenk tube, transferred to a separatory funnel, and extracted with EtOAc (3 x 30 mL). The combined organic layers were dried over sodium sulfate, filtered, and concentrated *in vacuo*. The crude residue was purified by silica gel flash chromatography (gradient 30-50% ethyl acetate/hexanes) to give 411 mg (65% yield) of the title compound as a yellow oil in a 20.1:79.9 ratio of enantiomers ((*R*)-**17**:(*S*)-**17**). TLC and ^{1}H NMR data match those of (±)-**17**.

Note: This reaction was monitored over time, and er was measured after 23 h and at the end of the reaction.

Aliquot after 23 h:

Determination of enantiomeric ratio: Follows general procedure **A**, gradual addition of aliquots of the preprepared $Eu(hfc)_3/CDCl_3$ solution to (*S*)-**17** in $CDCl_3$ showed the resonance at 6.44 ppm splitting into two resonances at 6.84, and 6.80 ppm (er = 78.7:22.3).

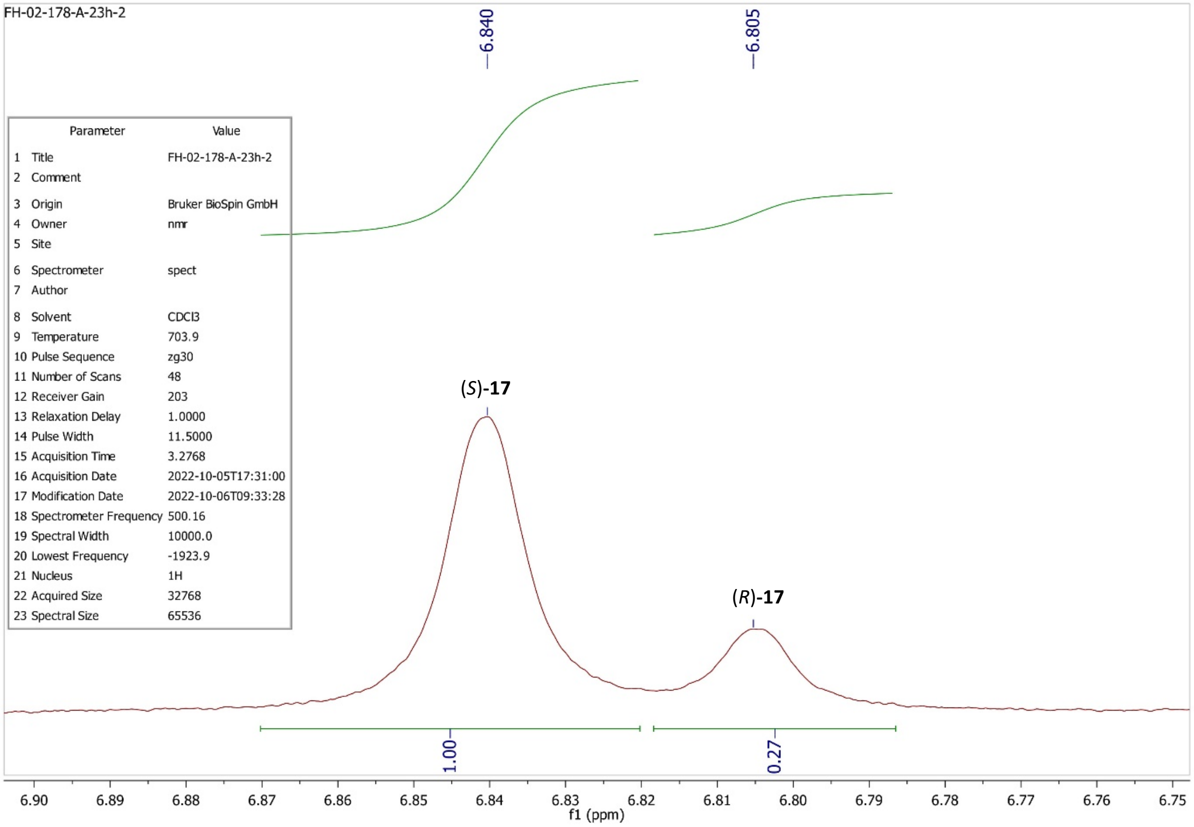

At the end of the reaction:

Shimadzu Nexera Series SCL-40, UV/PDA detector, 254 nm, CHIRALPAK IB N-3, 150 X 4.6 mm column, 10% *i*PrOH/hexanes, Flow rate: 1 mL/min

Peak	Retention Time (min)	Peak area (%)
Peak 1	5.73	79.88
Peak 2	6.46	20.12

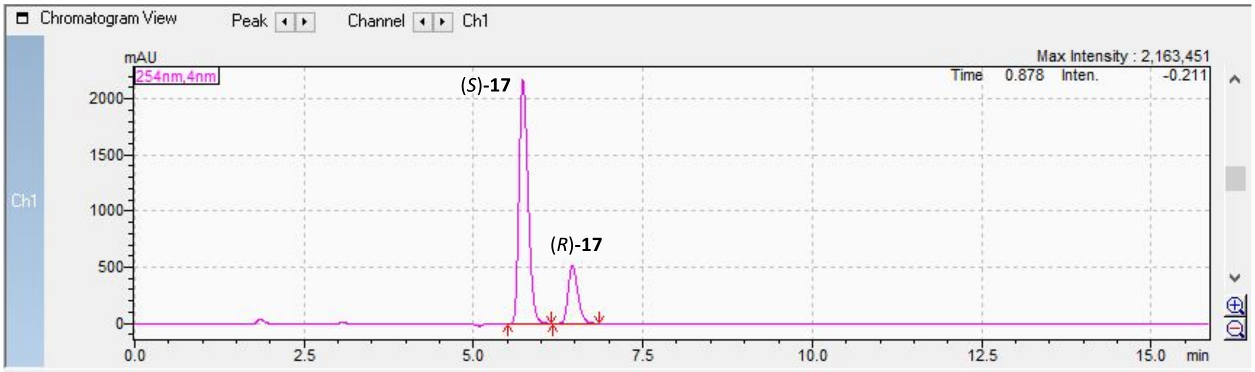

Results View - Peak Table

Peak Table | Compound | Group | Calibration Curve

Peak#	Ret. Time	Area	Height	Mark	Area%
1	5.733	20049518	2162667	M	79.883
2	6.462	5049021	516380	M	20.117
Total		25098539	2679047		100.000

Determination of enantiomeric ratio: Follows general procedure **A**, gradual addition of aliquots of the preprepared Eu(hfc)$_3$/$CDCl_3$ solution to (*S*)-**17** in $CDCl_3$ showed the resonance at 6.44 ppm splitting into two resonances at 6.87, and 6.83 ppm (er = 82:18).

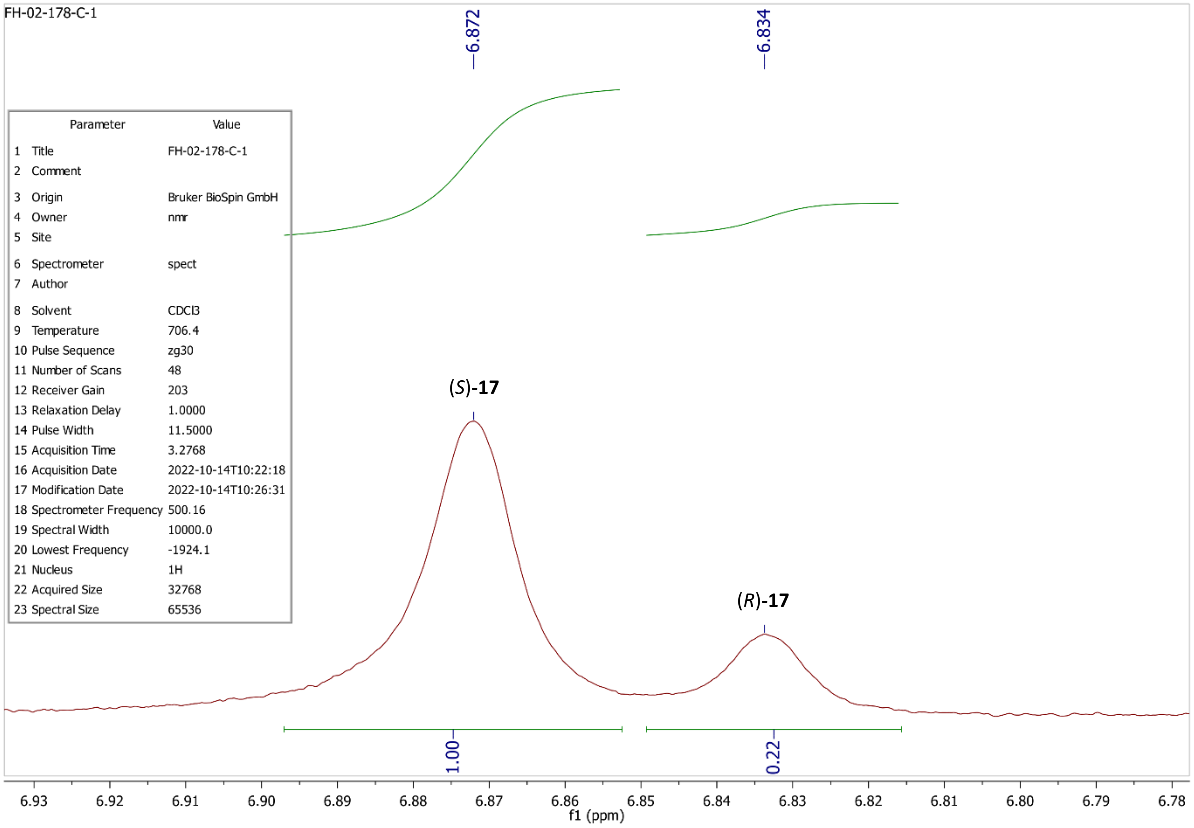

$[\alpha]^{20}_{D}$ = -31.7° (c = 1.0, $CHCl_3$)

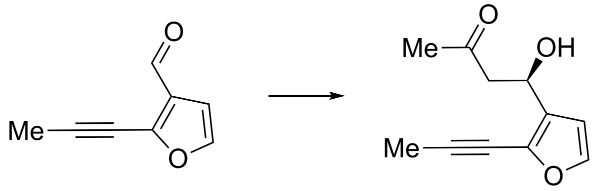

16 (*R*)-**17**

(*R*)-4-hydroxy-4-(2-(prop-1-yn-1-yl)furan-3-yl)butan-2-one ((*R*)-17).

Follows general procedure **B,** (*S*)-BINOL (0.02 equiv, 5 mg, 0.02 mmol), L-proline (0.3 equiv, 31 mg, 0.3 mmol), acetone (25 equiv, 1.7 mL, 22.4 mmol), DMSO (6 equiv, 0.4 mL, 5.1 mmol), 2-(prop-1-yn-1-yl)furan-3-carbaldehyde **16** (1 equiv, 120 mg, 0.9 mmol). After 354 h, saturated aqueous ammonium chloride (4 mL) was added to the Schelenk tube, transferred to a separatory funnel, and extracted with EtOAc (3 x 10 mL). The combined organic layers were dried over sodium sulfate, filtered, and concentrated *in vacuo*. The crude residue was purified by silica gel flash chromatography (gradient 30-50% ethyl acetate/hexanes) to give 105 mg (61% yield) of the title compound as a yellow oil in an 79.6:20.4 ratio of enantiomers ((*R*)-**17**:(*S*)-**17**). TLC and ^{1}H NMR data match those of (±)-**17**.

Shimadzu Nexera Series SCL-40, UV/PDA detector, 254 nm, CHIRALPAK IB N-3, 150 X 4.6 mm column, 10% *i*PrOH/hexanes, Flow rate: 1 mL/min

Peak	Retention Time (min)	Peak area (%)
Peak 1	5.89	20.42
Peak 2	6.64	79.58

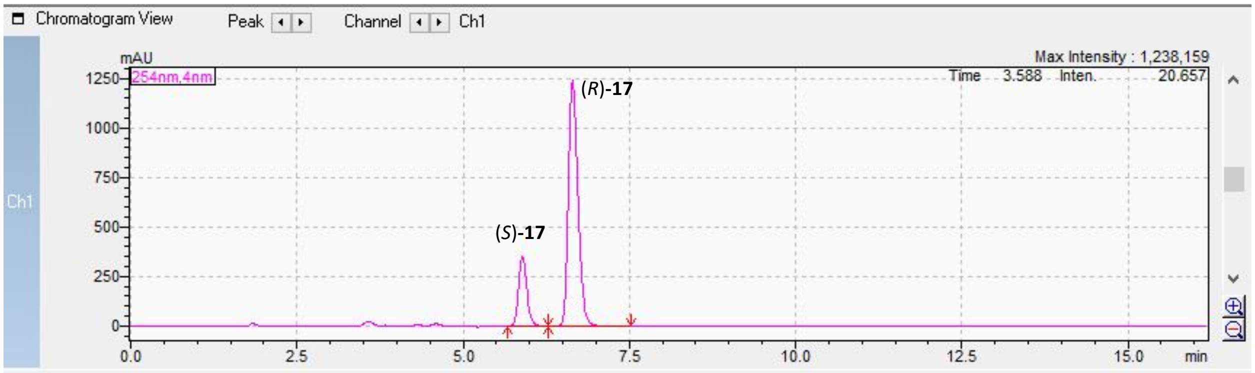

Results View - Peak Table

Peak Table | Compound | Group | Calibration Curve

Peak#	Ret. Time	Area	Height	Mark	Area%
1	5.889	3157257	355294	V	20.419
2	6.645	12304985	1239509	V	79.581
Total		15462242	1594803		100.000

Determination of enantiomeric ratio: Follows general procedure **A**, gradual addition of aliquots of the preprepared Eu(hfc)$_3$/CDCl$_3$ solution to (*R*)-**17** in CDCl$_3$ showed the resonance at 6.44 ppm splitting into two resonances at 6.72, and 6.70 ppm (er = 20:80).

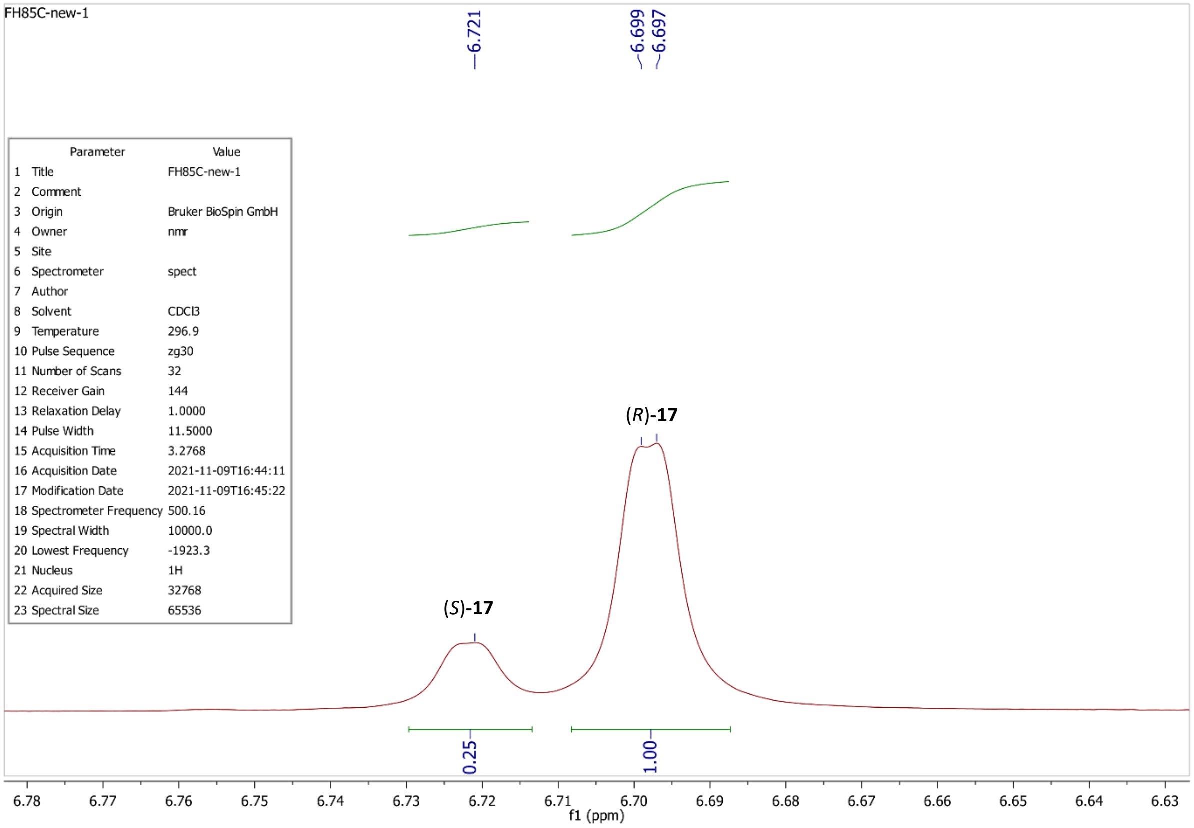

$[\alpha]^{20}_{D}$ = + 23.6° (c = 0.9, $CHCl_3$)

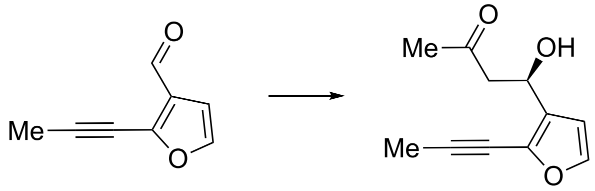

16 (*R*)-**17**

Synthesis of *β*-hydroxy ketone via an asymmetric aldol addition with acetone (*β* tosylamidoprolineamide)

(*R*)-4-hydroxy-4-(2-(prop-1-yn-1-yl)furan-3-yl)butan-2-one ((*R*)-17).

This compound was prepared in a manner similar to that previously reported, with some modifications.[4] A flame-dried, 2-mL Biotage microwave reaction vial equipped with a magnetic stir bar was charged with *β*-tosylamidoprolineamide **S12** (0.05 equiv, 2 mg, 0.004 mmol), followed by 2-(prop-1-yn-1-yl)furan-3-carbaldehyde **16** (1 equiv, 10 mg, 0.075 mmol) in acetone (27 equiv, 0.15 mL, 2.0 mmol). The vial was placed in a precooled Cryotrol bath (0 °C). After 24 h, TLC showed some starting material, therefore a second batch of catalyst was added (*β*-tosylamidoprolineamide (0.05 equiv, 2 mg, 0.004 mmol) in acetone (9 equiv, 0.05 mL, 0.7 mmol)). After 48 h, TLC showed a small amount of starting material, therefore a third batch of catalyst was added (*β*-tosylamidoprolineamide (0.05 equiv, 2 mg, 0.004 mmol) in acetone (9 equiv, 0.05 mL, 0.7 mmol)). After 68 h, TLC showed a trace amount of starting material. Saturated aqueous ammonium chloride (0.3 mL) was added to the vial, and extracted with EtOAc (3 x 0.3 mL). The combined organic layers were dried over sodium sulfate, filtered, and concentrated *in vacuo*. The crude residue was purified by silica gel flash chromatography (30% ethyl acetate/hexanes) to give 6 mg (40% yield) the title compound as a yellow oil in a 100:0 ratio of enantiomers ((*R*)-**17**:(*S*)-**17**) with 89% conversion. TLC and ^{1}H NMR data match those of (±)-**17**.

Note: The percent conversion was calculated based on integrative value of peak at 5.20 ppm **pdt** ^{1}H NMR benchmarked against peak at 9.98 ppm **SM**. The TLC and ^{1}H NMR of the crude reaction mixture showed mainly starting material and product.

Shimadzu Nexera Series SCL-40, UV/PDA detector, 254 nm, CHIRALPAK IB N-3, 150 X 4.6 mm column, 10% *i*PrOH/hexanes, Flow rate: 1 mL/min

Peak	Retention Time (min)	Peak area (%)
Peak 1	6.00	100.00

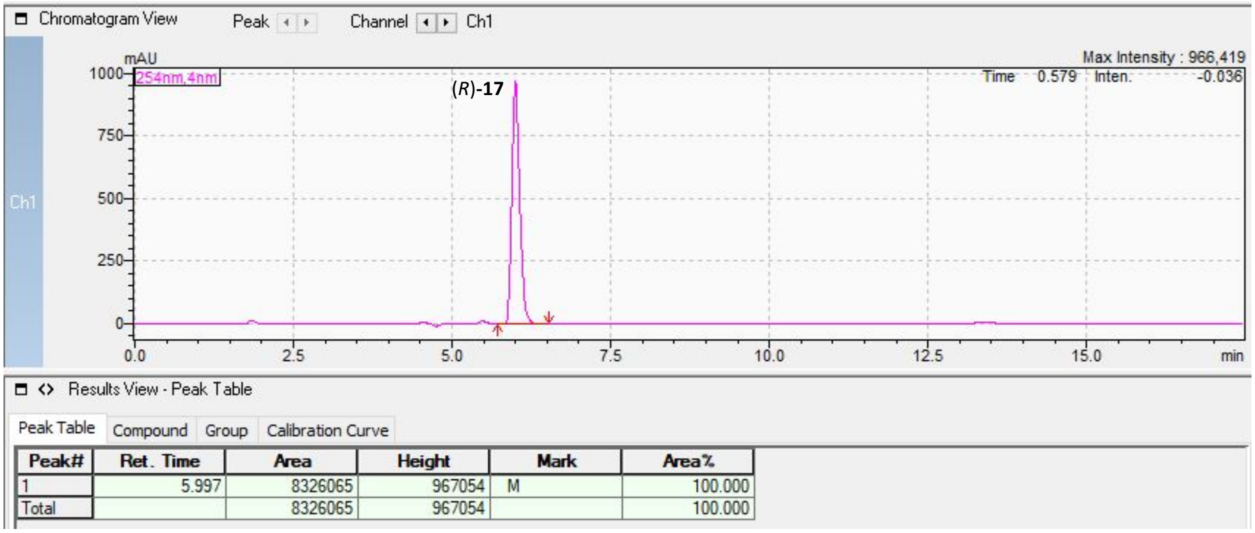

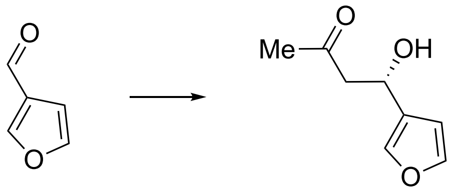

15 (*S*)-**S2**

(*S*)-4-(furan-3-yl)-4-hydroxybutan-2-one ((*S*)-S2).

Follows general procedure **B,** (*S*)-BINOL (0.02 equiv, 3 mg, 0.01 mmol), D-proline (0.3 equiv, 17 mg, 0.15 mmol), acetone (25 equiv, 1.0 mL, 12.5 mmol), DMSO (6 equiv, 0.2 mL, 2.8 mmol), furan-3-carbaldehyde **15** (1 equiv, 48 mg, 0.5 mmol). After 312 h, saturated aqueous ammonium chloride (2 mL) was added to the vial, transferred to a separatory funnel, and extracted with EtOAc (3 x 5 mL). The combined organic layers were dried over sodium sulfate, filtered, and concentrated *in vacuo*. The crude residue was purified by silica gel flash chromatography (30-40% ethyl acetate/hexanes) to give 14 mg (19% yield) of the title compound as a yellow oil in a 32:68 ratio of enantiomers ((*R*)-**S2**:(*S*)-**S2**).

^{1}H NMR (500 MHz, $CDCl_3$)

7.40–7.38 (m, 2 H), 6.39–6.39 (m, 1 H), 5.13 (dd, *J* = 8.8, 3.0 Hz, 1 H), 3.20 (brs, 1 H), 2.90 (dd, *J*= 17.8, 8.5 Hz, 1 H), 2.84 (dd, *J*= 17.5, 3.5 Hz, 1 H), 2.21 (s, 3 H) ppm

^{13}C NMR (125 MHz, $CDCl_3$)

209.0, 143.6, 139.1, 127.6, 108.6, 63.1, 50.7, 30.9 ppm

IR (Thin Film)

3417, 2921, 1708, 1290 cm^{-1}

HRMS HRMS-ESI (m/z): $[M + H]^+$ ($-H_2O$) calcd for $C_8H_9O_2$, 137.0602; found, 137.0598

TLC R_f = 0.18 (35% ethyl acetate/hexanes); silica gel, *p*-anisaldehyde, $KMnO_4$

Determination of enantiomeric ratio: Follows general procedure **A**, gradual addition of aliquots of the preprepared $Eu(hfc)_3/CDCl_3$ solution to (*S*)-**S2** in $CDCl_3$ showed the resonance at 6.39 ppm splitting into two resonances at 6.74, and 6.70 ppm (er = 68:32).

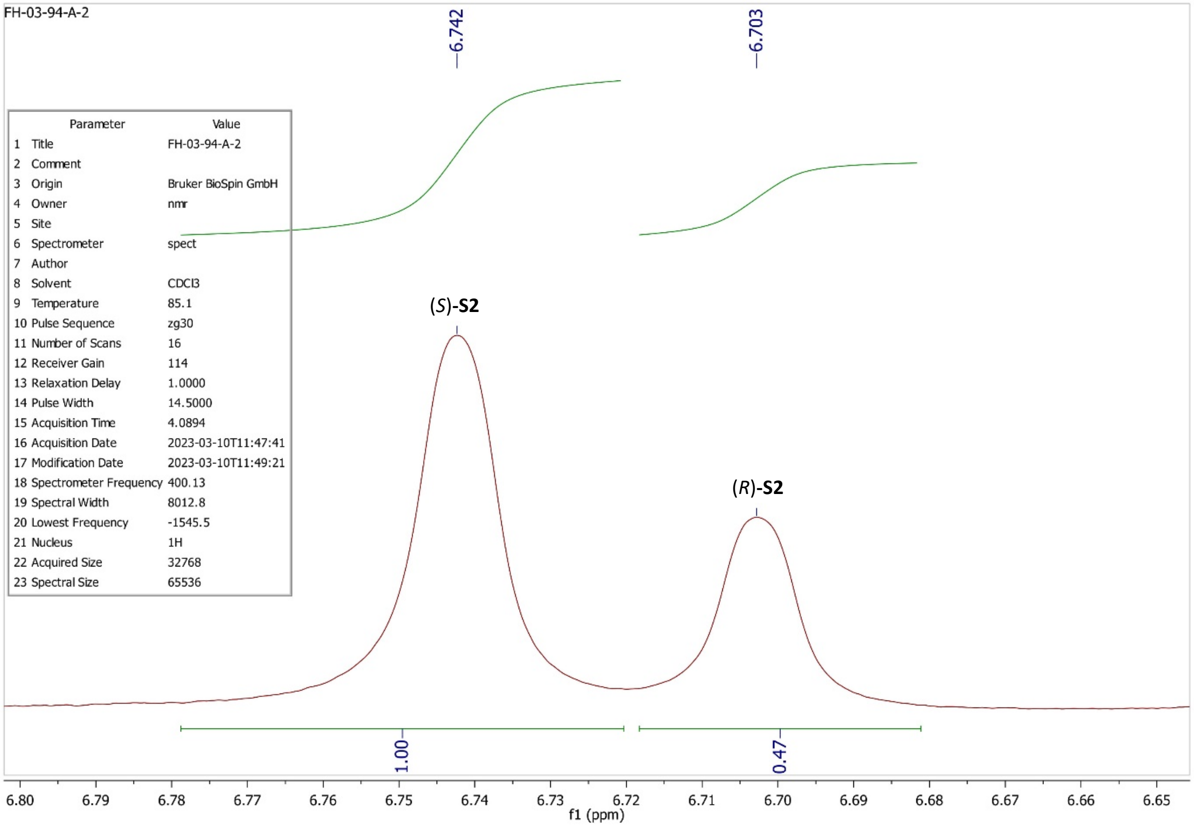

$[\alpha]^{20}_{D}$ = -65.3° (c = 0.5, $CHCl_3$)

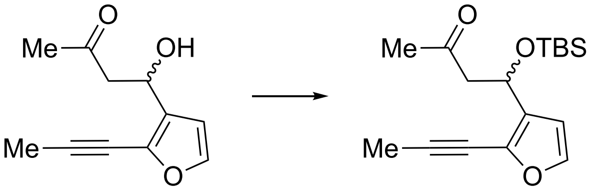

General procedure C: TBS protection of β-hydroxy ketone

(±)-4-((*tert*-butyldimethylsilyl)oxy)-4-(2-(prop-1-yn-1-yl)furan-3-yl)butan-2-one ((±)-18a).

This compound was prepared in a manner similar to that previously reported, with some modifications.[5] A flame-dried, single-necked, 15-mL, round-bottomed flask equipped with a

magnetic stir bar was charged with (±)-4-hydroxy-4-(2-(prop-1-yn-1-yl)furan-3-yl)butan-2-one (±)-**17** (1 equiv, 439 mg, 2.3 mmol) in DCM (3.1 mL). The reaction flask was placed in an ice bath, and imidazole (1.8 equiv, 282 mg, 4.1 mmol) was added. After 15 min, tert-butyldimethylsilyl chloride (TBSCl) (1.8 equiv, 624 mg, 4.1 mmol) was added. The ice-bath was removed, and the reaction was stirred at rt. After 5 h, the consumption of starting material was observed by TLC. Diethyl ether (4 mL) and deionized water (4 mL) were added to the flask, the mixture was transferred to a separatory funnel, and the organic layer was separated. The aqueous layer was extracted with diethyl ether (3 x 5 mL), washed with brine (4 mL), dried over sodium sulfate, filtered, and concentrated *in vacuo*. The crude residue was purified by silica gel flash chromatography (10% ethyl acetate/hexanes) to give 524 mg (74% yield) of the title compound as a yellow oil.

^{1}H NMR (400 MHz, $CDCl_3$)

7.23 (d, *J* = 1.6 Hz, 1 H), 6.40 (d, *J* = 2.0 Hz, 1 H), 5.22 (dd, *J* = 9.2, 4.0 Hz, 1 H), 2.95 (dd, *J*= 14.8, 9.2 Hz, 1 H), 2.50 (dd, *J*= 15.0, 3.6 Hz, 1 H), 2.18 (s, 3 H), 2.12 (s, 3 H), 0.82 (s, 9 H), 0.04 (s, 3 H), -0.11 (s, 3 H) ppm

^{13}C NMR (100 MHz, $CDCl_3$)

206.9, 142.8, 133.7, 131.4, 109.6, 94.0, 69.1, 64.2, 52.4, 31.7, 25.8, 18.2, 4.8, -4.8, -5.2 ppm

IR (Thin Film)

2955, 2930, 2856, 1718, 1252 cm^{-1}

HRMS HRMS-ESI (m/z): $[M + Na]^+$ calcd for $C_{17}H_{26}O_3NaSi$, 329.1543; found, 329.1542

TLC R_f = 0.63 (35% ethyl acetate/hexanes); silica gel, UV, *p*-anisaldehyde

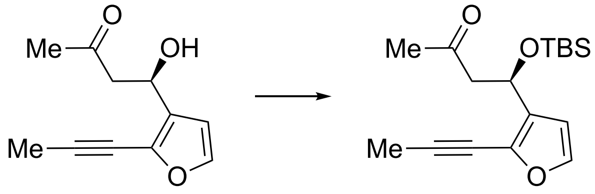

(*R*)-**17** (*R*)-**18a**

(*R*)-4-((*tert*-butyldimethylsilyl)oxy)-4-(2-(prop-1-yn-1-yl)furan-3-yl)butan-2-one ((*R*)-18a).

Follows general procedure **C**: (*R*)-4-hydroxy-4-(2-(prop-1-yn-1-yl)furan-3-yl)butan-2-one (*R*)-**17** (59.2% ee) (103 mg, 0.5 mmol), imidazole (66 mg, 1.0 mmol), TBSCl (145 mg, 1.0 mmol), DCM (1.0 mL). The reaction stirred for 5 h at rt. The crude residue was purified via silica gel flash chromatography (10% ethyl acetate/hexanes) to give 146 mg (88% yield) of the title compound as a yellow oil in an 81:19 ratio of enantiomers ((*R*)-**18a**:(*S*)-**18a**). TLC and ^{1}H NMR data match those of (±)-**18a**.

Determination of enantiomeric ratio: Follows general procedure **A**, gradual addition of aliquots of the preprepared Eu(hfc)$_3$/$CDCl_3$ solution to (*R*)-**18a** in $CDCl_3$ showed the resonance at 6.44 ppm splitting into two resonances at 6.81, and 6.79 ppm (er = 81:19).

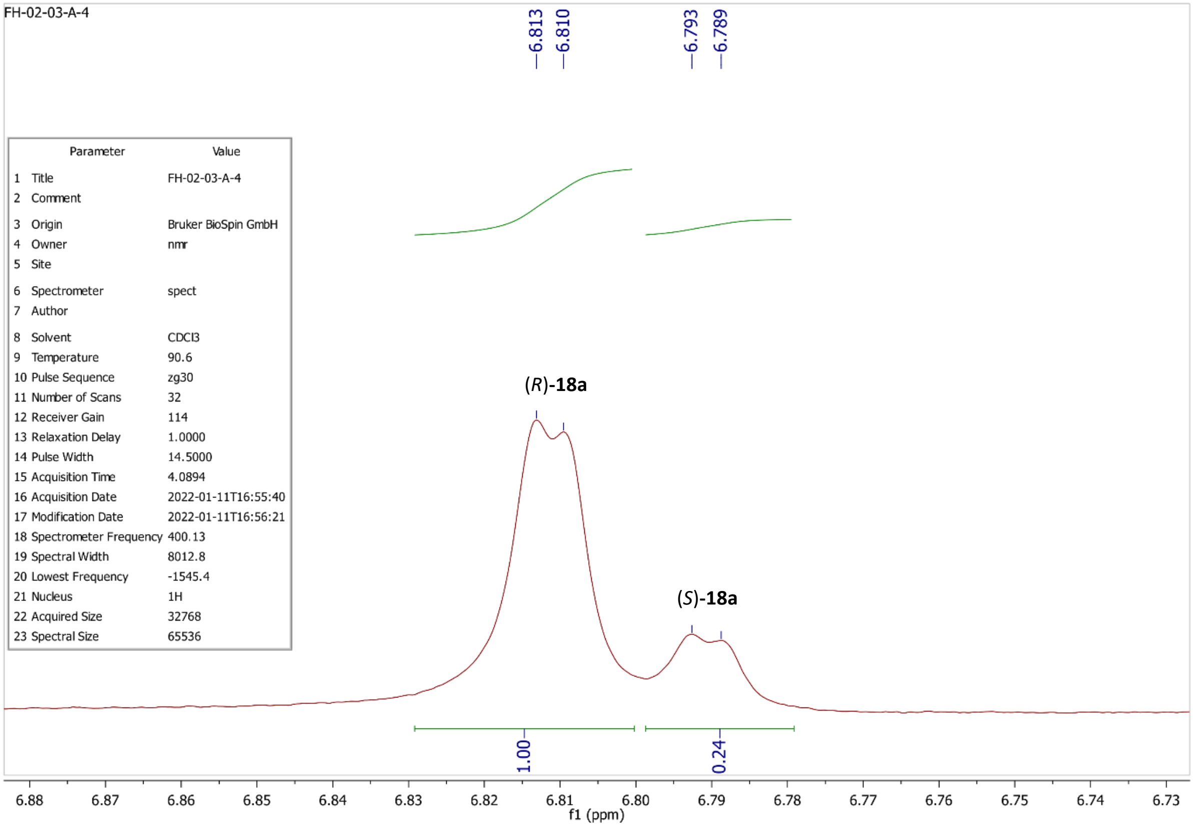

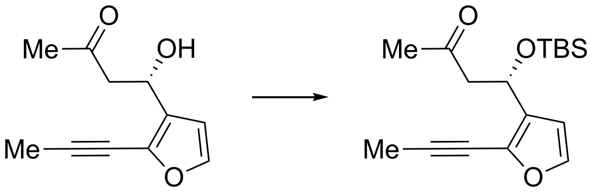

(*S*)-4-((*tert*-butyldimethylsilyl)oxy)-4-(2-(prop-1-yn-1-yl)furan-3-yl)butan-2-one ((*S*)-18a).

Follows general procedure **C**: (*S*)-4-hydroxy-4-(2-(prop-1-yn-1-yl)furan-3-yl)butan-2-one (*S*)-**17** (59.8% ee) (40 mg, 0.2 mmol), imidazole (28 mg, 0.4 mmol), TBSCl (56 mg, 0.4 mmol), DCM (0.3 mL). The reaction stirred for 5 h at rt. The crude residue was purified via silica gel flash

chromatography (10% ethyl acetate/hexanes) to give 51 mg (80% yield) of the title compound as a yellow oil. TLC and ^{1}H NMR data match those of (±)-**18a**.

Determination of enantiomeric ratio: Because there was no erosion in enantiomeric ratio observed in the conversion of (*R*)-4-hydroxy-4-(2-(prop-1-yn-1-yl)furan-3-yl)butan-2-one (*R*)-**17** to (*R*)-4-((*tert*-butyldimethylsilyl)oxy)-4-(2-(prop-1-yn-1-yl)furan-3-yl)butan-2-one (*R*)-**18a**, enantiomeric ratio of (*S*)-4-((*tert*-butyldimethylsilyl)oxy)-4-(2-(prop-1-yn-1-yl)furan-3-yl)butan-2-one (*S*)-**18a** was assumed from (*S*)-4-hydroxy-4-(2-(prop-1-yn-1-yl)furan-3-yl)butan-2-one (*S*)-**17**.

$[\alpha]^{20}_D$ = +23.9° (c = 0.7, $CHCl_3$)

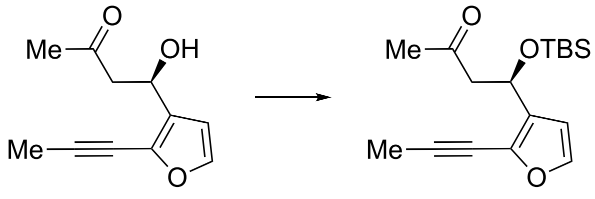

(*R*)-4-((*tert*-butyldimethylsilyl)oxy)-4-(2-(prop-1-yn-1-yl)furan-3-yl)butan-2-one ((*R*)-18a).

Follows general procedure **C**: (*R*)-4-hydroxy-4-(2-(prop-1-yn-1-yl)furan-3-yl)butan-2-one (*R*)-**17** (≥99% ee) (49 mg, 0.3 mmol), imidazole (34 mg, 0.5 mmol), TBSCl (70 mg, 0.5 mmol), DCM (0.4 mL). The reaction stirred for 5 h at rt. The crude residue was purified via silica gel flash chromatography (10% ethyl acetate/hexanes) to give 80 mg (82% yield) of the title compound as a yellow oil. TLC and ^{1}H NMR data match those of (±)-**18a**.

Determination of enantiomeric ratio: Determination of enantiomeric ratio: Because there was no erosion in enantiomeric ratio observed in the conversion of (*R*)-4-hydroxy-4-(2-(prop-1-yn-1-

yl)furan-3-yl)butan-2-one (*R*)-**17** (60% ee) to (*R*)-4-((*tert*-butyldimethylsilyl)oxy)-4-(2-(prop-1-yn-1-yl)furan-3-yl)butan-2-one (*R*)-**18a**, enantiomeric ratio of (*R*)-4-((*tert*-butyldimethylsilyl)oxy)-4-(2-(prop-1-yn-1-yl)furan-3-yl)butan-2-one (*R*)-**18a** was assumed from (*R*)-4-hydroxy-4-(2-(prop-1-yn-1-yl)furan-3-yl)butan-2-one (*R*)-**17** (≥99% ee).

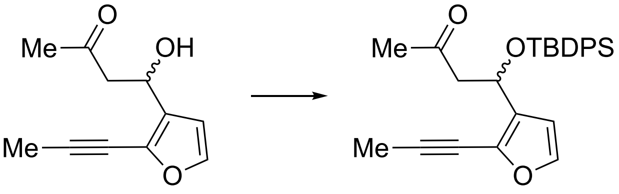

(±)-**17** (±)-**18b**

(±)-4-((*tert*-butyldiphenylsilyl)oxy)-4-(2-(prop-1-yn-1-yl)furan-3-yl)butan-2-one ((±)-18b).

This compound was prepared in a manner similar to that previously reported, with some modifications.[6] A flame-dried, 2-necked, 10-mL, pear-shaped flask equipped with a magnetic stir bar was charged with (±)-4-hydroxy-4-(2-(prop-1-yn-1-yl)furan-3-yl)butan-2-one (±)-**17** (1 equiv, 86 mg, 0.5 mmol), imidazole (4 equiv, 140 mg, 2.1 mmol), 4-Dimethylaminopyridine (DMAP) (0.1 equiv, 9 mg, 0.05 mmol) in THF (0.6 mL). The reaction flask was placed in an ice-bath and *tert*-Butyl(chloro)diphenylsilane (TBDPSCl) (1.1 equiv, 0.2 mL, 0.6 mmol) was added. The solution slowly warmed up to rt. After 5 h, TLC showed a trace amount of starting material. Diethyl ether (1 mL) and deionized water (1 mL) were added to the flask, the mixture was transferred to a separatory funnel, and the organic layer was separated. The aqueous layer was extracted with diethyl ether (3 x 2 mL), washed with brine (1 mL), dried over sodium sulfate, filtered, and concentrated *in vacuo*. The crude residue was purified by silica gel flash chromatography (3-5% ethyl acetate/hexanes) to give 118 mg (61% yield) of the title compound as a colorless oil.

^{1}H NMR (400 MHz, $CDCl_3$)

7.68–7.66 (m, 2 H), 7.55–7.52 (m, 2 H), 7.45–7.35 (m, 4 H), 7.31–7.27 (m, 2 H), 7.13 (d, J = 2.0 Hz, 1 H), 6.36 (d, J = 2.0 Hz, 1 H), 5.25 (t, J = 6.4 Hz, 1 H), 2.94 (dd, J= 14.8, 7.2 Hz, 1 H), 2.68 (dd, J= 14.8, 6.0 Hz, 1 H), 2.00 (s, 3 H), 1.94 (s, 3 H), 1.00 (s, 9 H) ppm

^{13}C NMR (100 MHz, $CDCl_3$)

206.1, 142.5, 136.1, 136.1, 134.2, 133.8, 133.4, 130.5, 129.8, 129.6, 127.6, 127.4, 109.8, 93.6, 69.0, 64.9, 52.7, 30.9, 27.0, 19.4, 4.7 ppm

IR (Thin Film)

2931, 2857, 1716, 1237 cm^{-1}

HRMS HRMS-ESI (m/z): $[M + Na]^+$ calcd for $C_{27}H_{30}O_3NaSi$, 453.1856; found, 453.1861

TLC R_f = 0.56 (35% ethyl acetate/hexanes); silica gel, UV, *p*-anisaldehyde

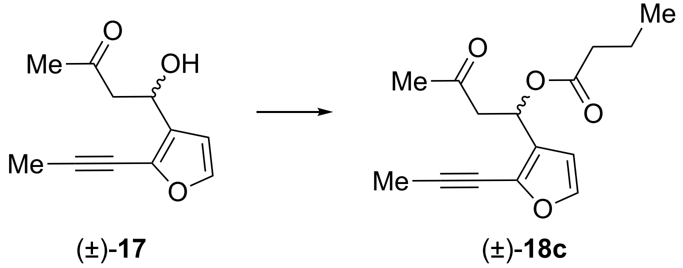

(±)-3-oxo-1-(2-(prop-1-yn-1-yl)furan-3-yl)butyl butyrate ((±)-18c).[7]

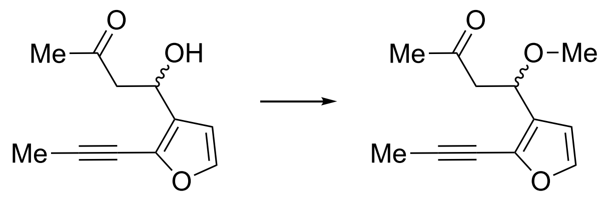

(±)-**17** (±)-**18d**

(±)-4-methoxy-4-(2-(prop-1-yn-1-yl)furan-3-yl)butan-2-one ((±)-18d).

This compound was prepared in a manner similar to that previously reported, with some modifications.[8] A flame-dried 2-mL Biotage microwave reaction vial equipped with a magnetic stir bar was charged with silver oxide (1.6 equiv, 66 mg, 0.3 mmol), iodomethane (9 equiv, 0.9 mL, 1.6 mmol), (±)-4-hydroxy-4-(2-(prop-1-yn-1-yl)furan-3-yl)butan-2-one (±)-**17** (1 equiv, 34 mg, 0.2 mmol) in diethyl ether (1.2 mL) and sealed with a Biotage cap. The vial was lowered into an oil bath preheated to 30 °C. After 70 h, the reaction mixture was filtered through a glass funnel, the reaction vial, the residue, and the filter paper were rinsed with diethyl ether (2 x), and the resulting filtrate was concentrated *in vacuo*. The crude residue was purified by silica gel flash chromatography (15% ethyl acetate/hexanes) to give 11 mg (30% yield) of the title compound as a colorless oil.

^{1}H NMR (500 MHz, $CDCl_3$)

7.28 (d, *J* = 1.5 Hz, 1 H), 6.36 (d, *J* = 1.5 Hz, 1 H), 4.74 (dd, *J* = 9.0, 4.0 Hz, 1 H), 3.20 (s, 3 H), 2.98 (dd, *J*= 15.5, 9.0 Hz, 1 H), 2.57 (dd, *J*= 15.5, 4.0 Hz, 1 H), 2.19 (s, 3 H), 2.12 (s, 3 H) ppm

^{13}C NMR (125 MHz, $CDCl_3$)

206.2, 143.1, 135.9, 127.7, 109.3, 93.7, 71.4, 68.9, 56.6, 49.9, 30.9, 4.8 ppm

IR (Thin Film)

2922, 2853, 2825, 2236, 1715, 1234 cm^{-1}

HRMS HRMS-ESI (m/z): $[M + H]^+$ calcd for $C_{12}H_{15}O_3$, 207.1016; found, 207.1017

TLC $R_f = 0.41$ (35% ethyl acetate/hexanes); silica gel, UV, *p*-anisaldehyde

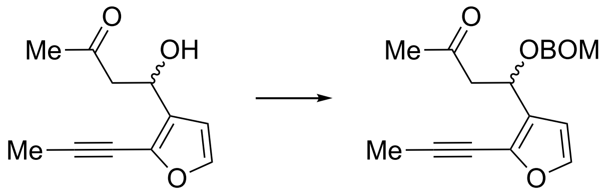

(±)-**17** (±)-**18e**

(±)-4-((benzyloxy)methoxy)-4-(2-(prop-1-yn-1-yl)furan-3-yl)butan-2-one ((±)-18e).

This compound was prepared in a manner similar to that previously reported, with some modifications.[9] A flame-dried 2-mL Biotage microwave reaction vial equipped with a magnetic stir bar was charged with tetrabutylammonium iodide (1.0 equiv, 96 mg, 0.3 mmol), (±)-4-hydroxy-4-(2-(prop-1-yn-1-yl)furan-3-yl)butan-2-one (±)-**17** (1 equiv, 50 mg, 0.3 mmol) in DCM (1.0 mL) and sealed with a Biotage cap. The vial was placed in an ice-bath and ^{i}Pr$_2$NEt (4.0 equiv, 0.2 mL, 1.0 mmol) was added. After 10 min, chloromethyl benzyl ether (2.5 equiv, 0.09 mL, 0.6 mmol) was added. The solution slowly warmed up to rt. After 45 min, The vial was lowered into an oil bath preheated to 50 °C. After 2 h, TLC showed a trace amount of starting material. The reaction mixture was transferred to a separatory funnel, diluted with DCM (1 mL), and washed sequentially with saturated aqueous sodium bicarbonate (1 mL) and saturated aqueous ammonium chloride (3 x 1 mL). The combined aqueous layers were back extracted with DCM (2 x 1 mL). The combined organic layers were washed with 0.2 N NaOH (1 mL), and brine (1 mL), dried over magnesium sulfate, filtered, and concentrated *in vacuo*. Ethyl acetate (1 mL) was added and the suspension was vigorously agitated followed by hexanes (2 mL). The mixture was filtered through Celite® 545, rinsed with 80% ethyl acetate/hexanes (1 mL), and concentrated *in vacuo*. The crude

residue was purified by silica gel flash chromatography (15% ethyl acetate/hexanes) to give 72 mg (89% yield) of the title compound as a yellow oil.

^{1}H NMR (500 MHz, $CDCl_3$)

7.26–7.17 (m, 6 H), 6.30 (d, J = 2.0 Hz, 1 H), 5.19 (dd, J = 9.2, 4.0 Hz, 1 H), 4.62–4.55 (m, 4 H), 2.97 (dd, J= 16.0, 9.5 Hz, 1 H), 2.57 (dd, J= 15.8, 4.0 Hz, 1 H), 2.12 (s, 3 H), 1.98 (s, 3 H) ppm

Inseparable impurities at 7.29–7.26 (m, 3.57), 4.81–4.77 (m, 0.53), 4.38 (d, J = 10.0 Hz, 1.07) ppm

^{13}C NMR (125 MHz, $CDCl_3$)

205.8, 143.1, 138.1, 135.8, 128.5, 128.1, 127.7, 127.6, 109.6, 94.0, 92.4, 69.9, 68.8, 66.5, 49.8, 31.0, 4.8 ppm

IR (Thin Film)

2952, 2921, 2887, 2233, 1715, 1258, 1230, 1158, 1099, 1020 cm^{-1}

HRMS HRMS-ESI (m/z): $[M + H]^+$ calcd for $C_{19}H_{21}O_4$, 313.1434; found, 313.1436

TLC R_f = 0.40 (35% ethyl acetate/hexanes); silica gel, UV, *p*-anisaldehyde

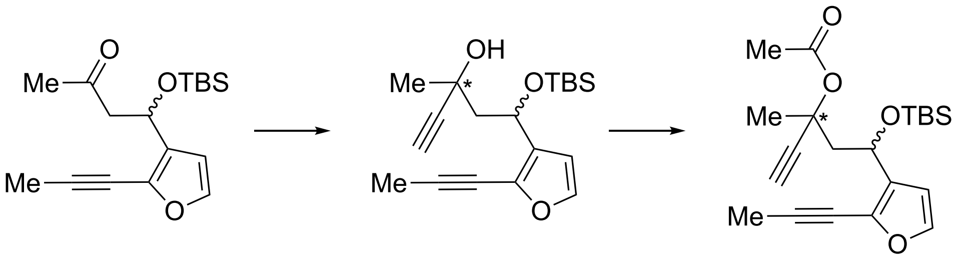

(±)-**18a** (±)-**S3** (±)-**19a**

General procedure D: Synthesis of propargyl carboxyesters

(±)-5-((*tert*-butyldimethylsilyl)oxy)-3-methyl-5-(2-(prop-1-yn-1-yl)furan-3-yl)pent-1-yn-3-yl acetate ((±)-19a).

This compound was prepared in a manner similar to that previously reported, with some modifications.[1] (±)-4-((*tert*-Butyldimethylsilyl)oxy)-4-(2-(prop-1-yn-1-yl)furan-3-yl)butan-2-one (±)-**18a** (333 mg, 1.1 mmol) was diluted with toluene and concentrated using high vacuum (3 x 1.5 mL). A flame-dried, 2-necked, 25-mL, round-bottomed flask equipped with a magnetic stir bar was charged with (±)-4-((*tert*-butyldimethylsilyl)oxy)-4-(2-(prop-1-yn-1-yl)furan-3-yl)butan-2-one (±)-**18a** (1 equiv, 333 mg, 1.1 mmol) in THF (3.8 mL). The reaction flask was placed in an ice-bath and ethynylmagnesium bromide (6.5 mL of a 0.5 M solution in THF, 3.3 mmol, 3 equiv) was added dropwise via syringe over 7 min. The solution slowly warmed up to rt. After 90 min, TLC showed complete consumption of starting material (±)-**18a** (*Rf* = 0.63 in 35% ethyl acetate/hexanes). The flask was placed in an ice-bath and acetyl chloride (0.3 mL, 3.8 mmol, 3.5 equiv) was added dropwise via syringe over 1 min. The solution slowly warmed to rt. After 3.5 h, TLC showed complete consumption of propargyl alcohol intermediate (±)-**S3** (*Rf* = 0.55, 0.65 in 35% ethyl acetate/hexanes). The mixture was transferred to a separatory funnel, diethyl ether (8 mL) and deionized water (8 mL) were added, and the organic layer was separated. The aqueous layer was extracted with diethyl ether (2 x 20 mL) and washed with brine (8 mL); the combined aqueous layers were back extracted with diethyl ether (1 x 15 mL), dried over magnesium sulfate, filtered, and concentrated *in vacuo*. The crude residue was purified by silica gel flash chromatography (12% ether/hexanes) to give 311 mg (76% yield) of the title compound as a yellow oil (diastereomeric ratio (dr) = 2.5:1).

^{1}H NMR (400 MHz, $CDCl_3$)

7.22 (d, J = 2.0 Hz, 2 H), 6.41–6.41 (m, 2 H), 5.16 (t, J = 6.0 Hz, 1 H)*, 5.04 (t, J = 6.0 Hz, 1 H)**, 2.61 (s, 1 H)*, 2.53 (s, 1 H)**, 2.43–2.24 (m, 4 H), 2.11 (s, 3 H)**, 2.10 (s, 3 H)*, 1.94 (s, 3 H)**, 1.87 (s, 3 H)*, 1.74 (s, 3 H)*, 1.70 (s, 3 H)**, 0.84 (s, 18 H), 0.07 (s, 3 H)*, 0.06 (s, 3 H)**, -0.13 (s, 3 H)* -0.14 (s, 3 H)** ppm

Trace impurities at 4.10 (t, J = 6.4 Hz, 0.29), 3.44 (t, J = 6.4 Hz, 0.30) ppm

distinguishable diastereomeric peaks for minor isomer *

distinguishable diastereomeric peaks for major isomer **

* dr = 2.5:1

^{13}C NMR (100 MHz, $CDCl_3$)

169.3*, 169.3**, 142.6**, 142.5*, 134.0*, 134.0**, 132.7*, 132.5**, 110.0*, 109.9**, 93.5*, 93.5**, 84.2**, 83.6*, 74.1*, 74.0*, 73.5**, 73.2**, 69.4, 64.4*, 63.8**, 49.4*, 49.0**, 27.6*, 27.0**, 26.0*, 25.9**, 21.9**, 21.9*, 18.2, 4.6, -4.6, -4.9*, -4.9** ppm

distinguishable diastereomeric peaks for minor isomer *

distinguishable diastereomeric peaks for major isomer **

IR (Thin Film)

3295, 2930, 2857, 1744, 1239 cm^{-1}

HRMS HRMS-ESI (m/z): $[M + Na]^+$ calcd for $C_{21}H_{30}O_4NaSi$, 397.1806; found, 397.1805

TLC R_f = 0.63 (35% ethyl acetate/hexanes); silica gel, UV, *p*-anisaldehyde

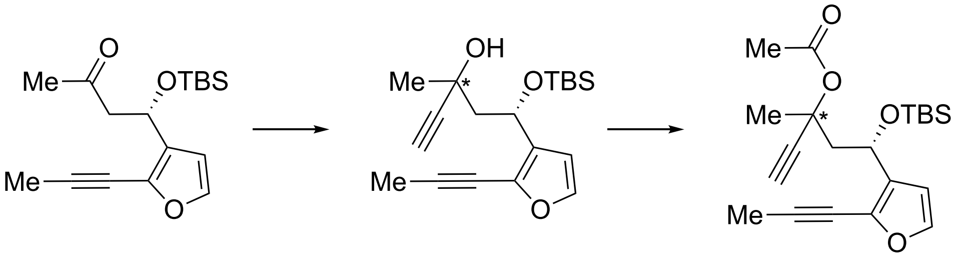

(*S*)-**18a** (*S*)-**S3** (*S*)-**19a**

(5*S*)-5-((*tert*-butyldimethylsilyl)oxy)-3-methyl-5-(2-(prop-1-yn-1-yl)furan-3-yl)pent-1-yn-3-yl acetate ((S)-19a).

Follows general procedure **D**, (*S*)-4-((*tert*-butyldimethylsilyl)oxy)-4-(2-(prop-1-yn-1-yl)furan-3-yl)butan-2-one (*S*)-**18a** (59.8% ee) (87 mg, 0.3 mmol), ethynylmagnesium bromide (1.8 mL of a 0.5 M solution in THF, 0.9 mmol), acetyl chloride (0.08 mL, 1.0 mmol, THF (1 mL). The reaction stirred for 5 h at rt. The crude residue was purified via silica gel flash chromatography (12% ether/hexanes) to give 79 mg (74% yield) of the title compound as a yellow oil (dr = 2.7:1). TLC and ^{1}H NMR data match those of **(±)-19a**.

Enantiomeric ratio of (5*S*)-5-((*tert*-butyldimethylsilyl)oxy)-3-methyl-5-(2-(prop-1-yn-1-yl)furan-3-yl)pent-1-yn-3-yl acetate (*S*)-**19a** was assumed from (*S*)-4-hydroxy-4-(2-(prop-1-yn-1-yl)furan-3-yl)butan-2-one (*S*)-**17**.

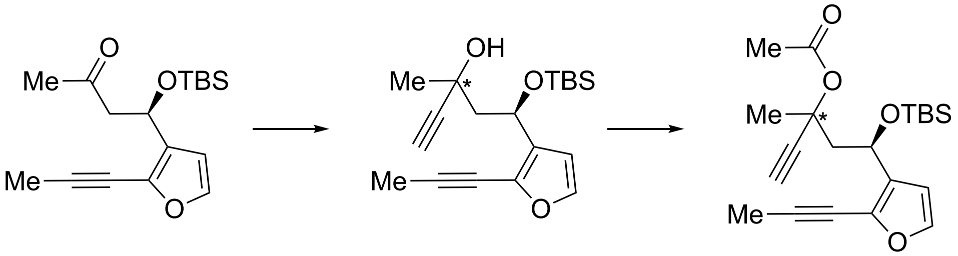

(*R*)-**18a** (*R*)-**S3** (*R*)-**19a**

(5*R*)-5-((*tert*-butyldimethylsilyl)oxy)-3-methyl-5-(2-(prop-1-yn-1-yl)furan-3-yl)pent-1-yn-3-yl acetate ((*R*)-19a).

Follows general procedure **D**, (*R*)-4-((*tert*-butyldimethylsilyl)oxy)-4-(2-(prop-1-yn-1-yl)furan-3-yl)butan-2-one (*R*)-**18a** (59.2% ee) (145 mg, 0.5 mmol), ethynylmagnesium bromide (2.8 mL of a 0.5 M solution in THF, 1.4 mmol), acetyl chloride (0.12 mL, 1.6 mmol), THF (1.6 mL). The reaction stirred for 5 h at rt. The crude residue was purified via silica gel flash chromatography (12% ether/hexanes) to give 154 mg (88% yield) of the title compound as a yellow oil (dr = 2.7:1). TLC and ^{1}H NMR data match those of (±)-**19a**.

Enantiomeric ratio of (5*R*)-5-((*tert*-butyldimethylsilyl)oxy)-3-methyl-5-(2-(prop-1-yn-1-yl)furan-3-yl)pent-1-yn-3-yl acetate (*R*)-**19a** was assumed from (*R*)-4-((*tert*-butyldimethylsilyl)oxy)-4-(2-(prop-1-yn-1-yl)furan-3-yl)butan-2-one (*R*)-**18a**.

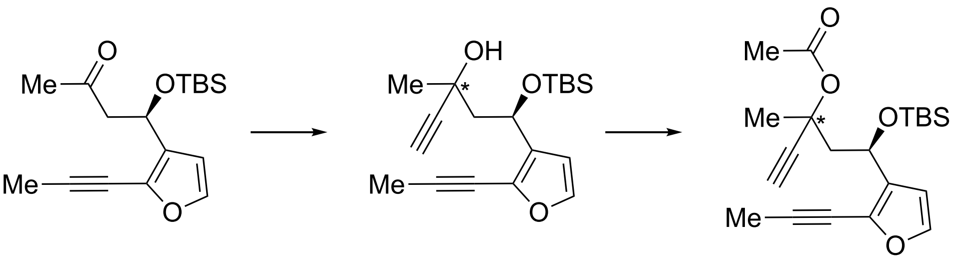

(*R*)-**18a** (*R*)-**S3** (*R*)-**19a**

(5*R*)-5-((*tert*-butyldimethylsilyl)oxy)-3-methyl-5-(2-(prop-1-yn-1-yl)furan-3-yl)pent-1-yn-3-yl acetate ((*R*)-19a).

Follows general procedure **D**, (*R*)-4-((*tert*-butyldimethylsilyl)oxy)-4-(2-(prop-1-yn-1-yl)furan-3-yl)butan-2-one (*R*)-**18a** (≥99% ee) (65 mg, 0.2 mmol), ethynylmagnesium bromide (1.2 mL of a 0.5 M solution in THF, 0.6 mmol), acetyl chloride (0.05 mL, 0.7 mmol), THF (0.7 mL). The

reaction stirred for 5 h at rt. The crude residue was purified via silica gel flash chromatography (12% ether/hexanes) to give 74 mg (94% yield) of the title compound as a yellow oil (dr = 2.7:1). TLC and ^{1}H NMR data match those of (±)-**19a**.

Enantiomeric ratio of (5*R*)-5-((*tert*-butyldimethylsilyl)oxy)-3-methyl-5-(2-(prop-1-yn-1-yl)furan-3-yl)pent-1-yn-3-yl acetate (*R*)-**19a** was assumed from (*R*)-4-((*tert*-butyldimethylsilyl)oxy)-4-(2-(prop-1-yn-1-yl)furan-3-yl)butan-2-one (*R*)-**18a**.

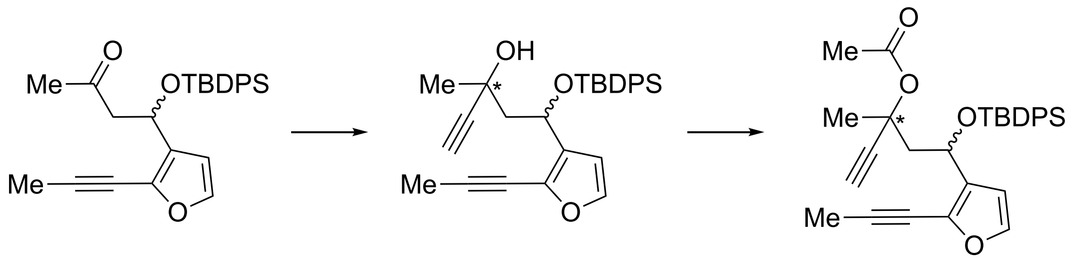

(±)-**18b** (±)-**S4** (±)-**19b**

(±)-5-((*tert*-butyldiphenylsilyl)oxy)-3-methyl-5-(2-(prop-1-yn-1-yl)furan-3-yl)pent-1-yn-3-yl acetate ((±)-19b).

This compound was prepared in a manner similar to that previously reported with some modifications.[1] (±)-4-((*tert*-butyldiphenylsilyl)oxy)-4-(2-(prop-1-yn-1-yl)furan-3-yl)butan-2-one (±)-**18b** (108 mg, 0.2 mmol) was diluted with toluene and concentrated using high vacuum (3 x 0.5 mL). A flame-dried, single-necked, 10-mL, round-bottomed flask equipped with a magnetic stir bar was charged with (±)-4-((*tert*-butyldiphenylsilyl)oxy)-4-(2-(prop-1-yn-1-yl)furan-3-yl)butan-2-one (±)-**18b** (1 equiv, 108 mg, 0.25 mmol) in THF (0.9 mL). The reaction flask was placed in an ice-bath and ethynylmagnesium bromide (1.5 mL of a 0.5 M solution in THF, 0.8 mmol, 3 equiv), was added dropwise via syringe over 1 min. The solution slowly warmed up to rt. After 90 min, TLC showed complete consumption of starting material (±)-**18b** (*Rf* = 0.56 in 35%

ethyl acetate/hexanes). The flask was placed in an ice-bath and acetyl chloride (0.06 mL, 0.9 mmol, 3.5 equiv), was added dropwise via syringe. The solution slowly warmed to rt. After 3 h, TLC showed complete consumption of propargyl alcohol intermediate (±)-**S4** (*Rf* = 0.60, 0.67 in 35% ethyl acetate/hexanes). The mixture was transferred to a separatory funnel, diethyl ether (2 mL) and deionized water (2 mL) were added, and the organic layer was separated. The aqueous layer was extracted with diethyl ether (2 x 5 mL), washed with brine (2 mL), the combined aqueous layers were back extracted with diethyl ether (1 x 5 mL), dried over magnesium sulfate, filtered, and concentrated *in vacuo*. The crude residue was purified by silica gel flash chromatography (5% ethyl acetate /hexanes) to give 90 mg (72% yield) of the title compound as a colorless oil (dr = 2:1).

^{1}H NMR (400 MHz, $CDCl_3$)

7.70–7.67 (m, 4 H), 7.54–7.51 (m, 4 H), 7.44–7.35 (m, 8 H),7.31–7.27 (m, 4 H), 7.18 (d, *J* = 2.0 Hz, 1 H)**, 7.16 (d, *J* = 1.6 Hz, 1 H)*, 6.44 (d, *J* = 1.6 Hz, 1 H)**, 6.43 (d, *J* = 2.0 Hz, 1 H)*, 5.16 (dd, *J* = 9.4, 3.6 Hz, 1 H)*, 4.99 (dd, *J* = 9.6, 4.0 Hz, 1 H)**, 2.70 (dd, *J* = 14.0, 9.6 Hz, 1 H)**, 2.56 (dd, *J* = 15.0, 9.2 Hz, 1 H)*, 2.38 (s, 1 H)**, 2.29 (s, 1 H)*, 2.32–2.24 (m, 2 H), 1.91 (s, 3 H)**, 1.89 (s, 3 H)*, 1.69 (s, 3 H)**, 1.68 (s, 3 H)*, 1.55 (s, 3 H)*, 1.39 (s, 3 H)**, 1.00 (s, 9 H)*, 1.00 (s, 9 H)** ppm

distinguishable diastereomeric peaks for minor isomer *

distinguishable diastereomeric peaks for major isomer **

* dr = 2:1

^{13}C NMR (100 MHz, $CDCl_3$)

169.3*, 169.2**, 142.4**, 142.1*, 136.1*, 136.1*, 136.1**, 136.1**, 134.7*, 134.7**, 134.2*, 134.2**, 133.7*, 133.6**, 131.9*, 131.5**, 129.7**, 129.6*, 129.6**, 129.5*, 127.6**, 127.6*, 127.4**, 127.4*, 110.3*, 110.2**, 93.0, 83.9**, 83.1*, 73.7*, 73.3**, 73.1*, 72.4**, 69.4*, 69.3**, 65.3*, 64.3**, 49.3*, 48.3**, 27.2*, 27.1**, 27.0**, 26.6*, 21.6, 19.4*, 19.4**, 4.6*, 4.6** ppm

distinguishable diastereomeric peaks for minor isomer *

distinguishable diastereomeric peaks for major isomer **

IR (Thin Film)

3295, 2932, 2858, 2239, 2120, 1743, 1239 cm^{-1}

HRMS HRMS-ESI (m/z): $[M + H]^+$ calcd for $C_{31}H_{35}O_4Si$, 499.2299; found, 499.2283

TLC R_f = 0.58 (35% ethyl acetate/hexanes); silica gel, UV, *p*-anisaldehyde

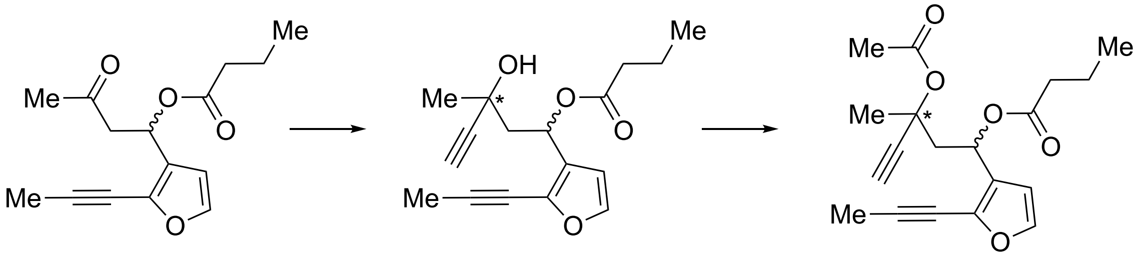

(±)-**18c** (±)-**S5** (±)-**19c**

(±)-3-acetoxy-3-methyl-1-(2-(prop-1-yn-1-yl)furan-3-yl)pent-4-yn-1-yl butyrate ((±)-19c).[7]

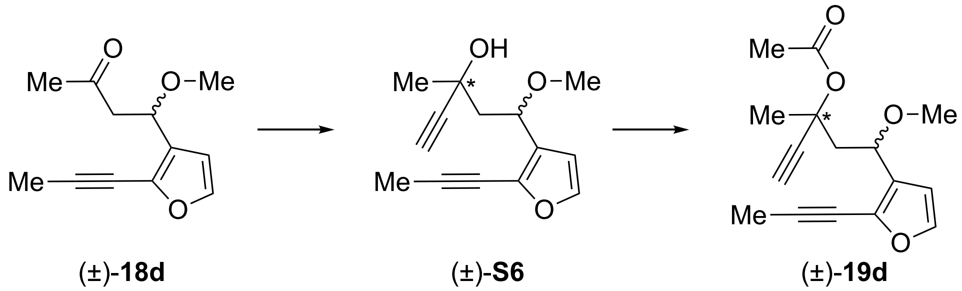

(±)-**18d** (±)-**S6** (±)-**19d**

(±)-5-methoxy-3-methyl-5-(2-(prop-1-yn-1-yl)furan-3-yl)pent-1-yn-3-yl acetate ((±)-19d).

This compound was prepared in a manner similar to that previously reported with some modifications.[1] (±)-4-methoxy-4-(2-(prop-1-yn-1-yl)furan-3-yl)butan-2-one (±)-**18d** (31 mg, 0.15 mmol) was diluted with toluene and concentrated using high vacuum (3 x 0.5 mL). A flame-dried 2-mL Biotage microwave reaction vial equipped with a magnetic stir bar was charged with (±)-4-methoxy-4-(2-(prop-1-yn-1-yl)furan-3-yl)butan-2-one (±)-**18d** (1 equiv, 31 mg, 0.15 mmol) in THF (0.6 mL). The reaction vial was placed in an ice-bath and ethynylmagnesium bromide (1.2 mL of a 0.5 M solution in THF, 0.6 mmol, 4 equiv) was added dropwise via syringe over 1 min. The solution slowly warmed up to rt. After 90 min, TLC showed complete consumption of starting material (±)-**18d** (*Rf* = 0.41 in 35% ethyl acetate/hexanes). The vial was placed in an ice-bath and acetyl chloride (0.04 mL, 0.5 mmol, 3.5 equiv) was added dropwise via syringe. The solution slowly warmed to rt. After 3.5 h, TLC showed complete consumption of propargyl alcohol intermediate (±)-**S6** (*Rf* = 0.34, 0.49 in 35% ethyl acetate/hexanes). The mixture was transferred to a separatory funnel, diethyl ether (1 mL) and deionized water (1 mL) were added, and the organic layer was separated. The aqueous layer was extracted with diethyl ether (2 x 3 mL), washed with brine (1 mL), the combined aqueous layers were back extracted with diethyl ether (1 x 2 mL), dried over magnesium sulfate, filtered, and concentrated *in vacuo*. The crude residue was purified by silica gel flash chromatography (5-8% ethyl acetate/hexanes) to give 33 mg (80% yield) of the title compound as a yellow oil (dr = 3:1).

^{1}H NMR (400 MHz, $CDCl_3$)

7.28 (d, *J* = 1.6 Hz, 2 H), 6.37 (d, *J* = 1.6 Hz, 2 H), 4.63–4.60 (m, 1 H)*, 4.55–4.53 (m, 1 H)**, 3.19 (s, 3 H)*, 3.18 (s, 3 H)**, 2.62 (s, 1 H)*, 2.55 (s, 1 H)**, 2.54–

2.25 (m, 4 H), 2.11 (s, 3 H)**, 2.11 (s, 3 H)*, 1.98 (s, 3 H)**, 1.93 (s, 3 H)*, 1.76 (s, 3 H)*, 1.73 (s, 3 H)** ppm

distinguishable diastereomeric peaks for minor isomer *

distinguishable diastereomeric peaks for major isomer **

* dr = 3:1

^{13}C NMR (100 MHz, $CDCl_3$)

169.4**, 169.4*, 143.0**, 142.9*, 135.8, 129.0*, 128.9**, 109.4*, 109.4**, 93.4*, 93.3**, 84.2**, 83.5*, 73.9*, 73.9*, 73.6**, 73.3**, 72.3*, 71.9**, 69.1, 56.2*, 56.1**, 46.6*, 46.4**, 27.6*, 27.0**, 22.0**, 21.9*, 4.7 ppm

distinguishable diastereomeric peaks for minor isomer *

distinguishable diastereomeric peaks for major isomer **

IR (Thin Film)

3289, 2933, 2823, 1741, 1236 cm^{-1}

HRMS HRMS-ESI (m/z): $[M + Na]^+$ calcd for $C_{16}H_{18}O_4Na$, 297.1097; found, 297.1097

TLC R_f = 0.60 (35% ethyl acetate/hexanes); silica gel, UV, *p*-anisaldehyde

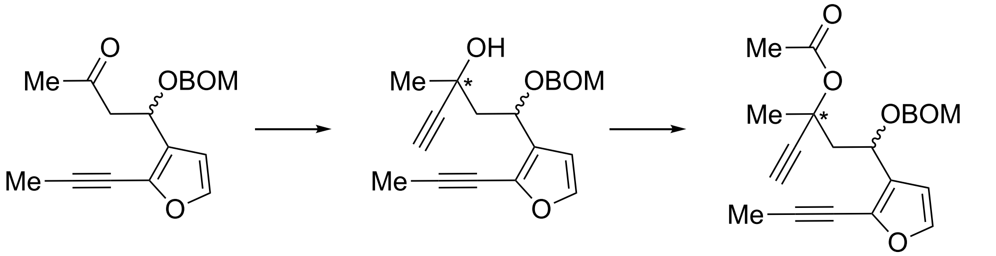

(±)-**18e** (±)-**S7** (±)-**19e**

(±)-5-((benzyloxy)methoxy)-3-methyl-5-(2-(prop-1-yn-1-yl)furan-3-yl)pent-1-yn-3-yl acetate ((±)-19e).

This compound was prepared in a manner similar to that previously reported with some modifications.[1] (±)-4-((benzyloxy)methoxy)-4-(2-(prop-1-yn-1-yl)furan-3-yl)butan-2-one (±)-**18e** (64 mg, 0.2 mmol) was diluted with toluene and concentrated using high vacuum (3 x 0.5 mL). A flame-dried, single-necked, 10-mL, round-bottomed flask equipped with a magnetic stir bar was charged with (±)-4-((benzyloxy)methoxy)-4-(2-(prop-1-yn-1-yl)furan-3-yl)butan-2-one (±)-**18e** (1 equiv, 64 mg, 0.2 mmol) in THF (0.7 mL). The reaction flask was placed in an ice-bath and ethynylmagnesium bromide (1.2 mL of a 0.5 M solution in THF, 0.6 mmol, 3 equiv), was added dropwise via syringe over 1 min. The solution slowly warmed up to rt. After 90 min, TLC showed complete consumption of starting material (±)-**18e** (*Rf* = 0.40 in 35% ethyl acetate/hexanes). The flask was placed in an ice-bath and acetyl chloride (0.05 mL, 0.7 mmol, 3.5 equiv), was added dropwise via syringe. The solution slowly warmed to rt. After 3 h, TLC showed complete consumption of propargyl alcohol intermediate (±)-**S7** (*Rf* = 0.39, 0.50 in 35% ethyl acetate/hexanes). The mixture was transferred to a separatory funnel, diethyl ether (2 mL) and deionized water (2 mL) were added, and the organic layer was separated. The aqueous layer was extracted with diethyl ether (2 x 5 mL), washed with brine (2 mL), the combined aqueous layers were back extracted with diethyl ether (1 x 5 mL), dried over magnesium sulfate, filtered, and concentrated *in vacuo*. The crude residue was purified by silica gel flash chromatography (5–8% ethyl acetate /hexanes) to give 45 mg (59% yield) of the title compound as a yellow oil (dr = 2.6:1).

^{1}H NMR (500 MHz, $CDCl_3$)

7.34–7.27 (m, 12 H), 6.41–6.40 (m, 2 H), 5.19 (t, *J* = 6.0 Hz, 1 H)*, 5.11 (t, *J* = 6.0 Hz, 1 H)**, 4.72–4.64 (m, 6 H), 4.52 (d, *J* = 11.5 Hz, 1 H)**, 4.52 (d, *J* = 12.0 Hz, 1 H)**, 2.60–2.35 (m, 6 H), 2.05 (s, 3 H)**, 2.05 (s, 3 H)*, 1.94 (s, 3 H)**, 1.89 (s, 3 H)*, 1.77 (s, 3 H)*, 1.74 (s, 3 H)** ppm

distinguishable diastereomeric peaks for minor isomer *

distinguishable diastereomeric peaks for major isomer **

* dr = 2.6:1

^{13}C NMR (125 MHz, $CDCl_3$)

169.3, 143.0**, 142.9*, 138.2*, 138.2**, 136.0*, 136.0**, 128.8*, 128.7**, 128.5**, 128.5*, 127.9**, 127.8*, 127.7**, 127.7*, 109.8*, 109.8**, 93.6, 92.2**, 92.2*, 84.0**, 83.5*, 74.1*, 73.7*, 73.6**, 73.5**, 69.9**, 69.9*, 69.0*, 69.0**, 67.1*, 66.8**, 46.6*, 46.1**, 27.6*, 27.1**, 21.9**, 21.8*, 4.7 ppm

distinguishable diastereomeric peaks for minor isomer *

distinguishable diastereomeric peaks for major isomer **

IR (Thin Film)

3289, 2936, 2887, 2237, 1746, 1238, 1096, 1023 cm^{-1}

HRMS HRMS-ESI (m/z): $[M + Na]^+$ calcd for $C_{23}H_{24}O_5Na$, 403.1492; found, 403.1491

TLC R_f = 0.53 (35% ethyl acetate/hexanes); silica gel, UV, *p*-anisaldehyde

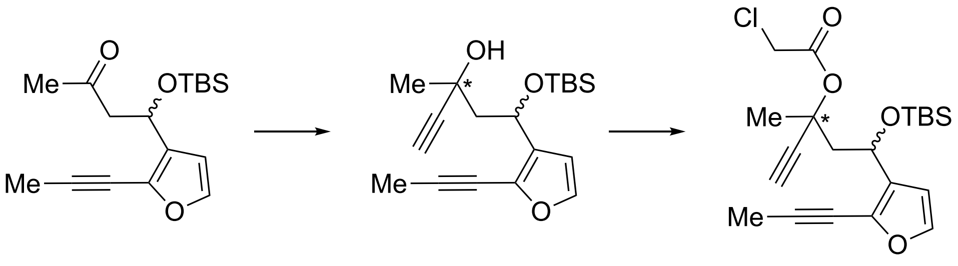

(±)-**18a** (±)-**S3** (±)-**19f**

(±)-5-((*tert*-butyldimethylsilyl)oxy)-3-methyl-5-(2-(prop-1-yn-1-yl)furan-3-yl)pent-1-yn-3-yl 2-chloroacetate ((±)-19f).

This compound was prepared in a manner similar to that previously reported with some modifications.[1] (±)-4-((*tert*-Butyldimethylsilyl)oxy)-4-(2-(prop-1-yn-1-yl)furan-3-yl)butan-2-one (±)-**18a** (191 mg, 0.6 mmol) was diluted with toluene and concentrated using high vacuum (3 x 1.5 mL). A flame-dried, 2-necked, 15-mL, round-bottomed flask equipped with a magnetic stir bar was charged with (±)-4-((*tert*-butyldimethylsilyl)oxy)-4-(2-(prop-1-yn-1-yl)furan-3-yl)butan-2-one (±)-**18a** (1 equiv, 191 mg, 0.6 mmol) in THF (2.2 mL). The reaction flask was placed in an ice-bath and ethynylmagnesium bromide (3.8 mL of a 0.5 M solution in THF, 1.9 mmol, 3 equiv), was added dropwise via syringe over 10 min. The solution slowly warmed up to rt. After 2 h, TLC showed complete consumption of starting material (±)-**18a** (*Rf* = 0.63 in 35% ethyl acetate/hexanes). The flask was placed in an ice-bath and chloroacetyl chloride (0.2 mL, 2.2 mmol, 3.5 equiv), was added dropwise via syringe over 1 min. The solution slowly warmed to rt. After 3 h, TLC showed complete consumption of propargyl alcohol intermediate (±)-**S3** (*Rf* = 0.55, 0.65 in 35% ethyl acetate/hexanes). The mixture was transferred to a separatory funnel, diethyl ether (5 mL) and deionized water (5 mL) were added, and the organic layer was separated. The aqueous layer was extracted with diethyl ether (2 x 15 mL), washed with brine (5 mL), the combined aqueous layers were back extracted with diethyl ether (1 x 10 mL), dried over magnesium sulfate, filtered, and concentrated *in vacuo*. The crude residue was purified by silica gel flash chromatography (6% ether/hexanes) to give 190 mg (64% yield) of the title compound as a yellow oil (dr = 2.5:1).

^{1}H NMR (400 MHz, $CDCl_3$)

7.23 (d, *J* = 1.6 Hz, 2 H), 6.42–6.40 (m, 2 H), 5.17 (t, *J* = 6.0 Hz, 1 H)*, 5.04 (t, *J* = 6.4 Hz, 1 H)**, 3.98–3.77 (m, 4 H), 2.67 (s, 1 H)*, 2.57 (s, 1 H)**, 2.44–2.27

(m, 4 H), 2.12 (s, 3 H)**, 2.10 (s, 3 H)*, 1.78 (s, 3 H)*, 1.74 (s, 3 H)**, 0.84 (s, 18 H), 0.07 (s, 3 H)*, 0.06 (s, 3 H)**, -0.13 (s, 3 H)* -0.14 (s, 3 H)** ppm

Trace less-polar diastereomer of propargyl alcohol intermediate (±)-**S3** at 7.25 (d, *J* = 2.0 Hz, 0.26 H), 5.44 (dd, *J* = 10.8, 2.4 Hz, 0.22 H), 5.13 (s, 0.20 H), 2.53 (s, 0.21 H), 2.12 (s, 0.72 H), 1.49 (s, 0.66 H), 0.87 (s, 1.93 H), 0.17 (s, 0.60 H), -0.16 (s, 0.66 H) ppm

distinguishable diastereomeric peaks for minor isomer *

distinguishable diastereomeric peaks for major isomer **

* dr = 2.5:1

^{13}C NMR (100 MHz, $CDCl_3$)

165.3*, 165.2**, 142.8**, 142.7*, 134.0**, 134.0*, 132.5*, 132.2**, 109.9*, 109.9**, 93.8*, 93.7**, 83.1**, 82.5*, 76.3*, 75.5**, 75.1*, 74.4**, 69.3, 64.4*, 63.6**, 49.3*, 48.7**, 41.4**, 41.4*, 27.6*, 26.9**, 26.0*, 25.9**, 18.2*, 18.1**, 4.7, -4.6, -4.9*, -4.9** ppm

distinguishable diastereomeric peaks for minor isomer *

distinguishable diastereomeric peaks for major isomer **

IR (Thin Film)

2959, 2929, 1767, 1226, 1214 cm^{-1}

HRMS HRMS-ESI (m/z): $[M - H]^+$ calcd for $C_{21}H_{28}ClO_4Si$, 407.15; found, 407.1140

TLC R_f = 0.50 (10% ethyl acetate/hexanes); silica gel, UV, *p*-anisaldehyde

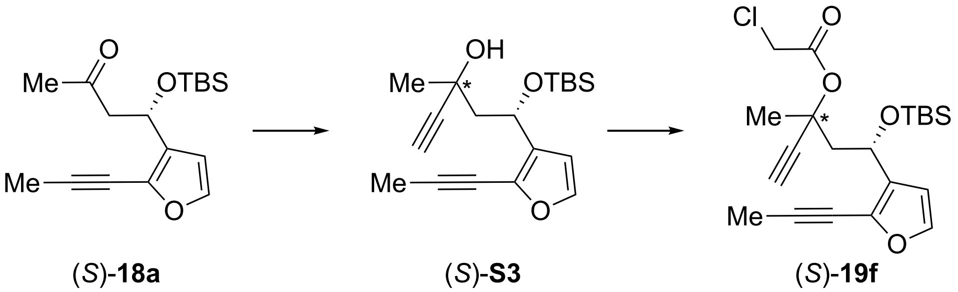

(5*S*)-5-((*tert*-butyldimethylsilyl)oxy)-3-methyl-5-(2-(prop-1-yn-1-yl)furan-3-yl)pent-1-yn-3-yl 2-chloroacetate ((*S*)-19f).

Follows general procedure **D**, (*S*)-4-((*tert*-butyldimethylsilyl)oxy)-4-(2-(prop-1-yn-1-yl)furan-3-yl)butan-2-one (*S*)-**18a** (59.8% ee) (143 mg, 0.5 mmol), ethynylmagnesium bromide (3.2 mL of a 0.5 M solution in THF, 1.5 mmol), chloroacetyl chloride (0.15 mL, 1.9 mmol), THF (1.6 mL). The reaction stirred for 5 h at rt. The crude residue was purified via silica gel flash chromatography (6% ether/hexanes) to give 138 mg (72% yield) of the title compound as a yellow oil (dr = 2.5:1). TLC and ^{1}H NMR data match those of (±)-**19f**.

Enantiomeric ratio of (5*S*)-5-((*tert*-butyldimethylsilyl)oxy)-3-methyl-5-(2-(prop-1-yn-1-yl)furan-3-yl)pent-1-yn-3-yl 2-chloroacetate (*S*)-**19f** was assumed from (*S*)-4-hydroxy-4-(2-(prop-1-yn-1-yl)furan-3-yl)butan-2-one (*S*)-**17**.

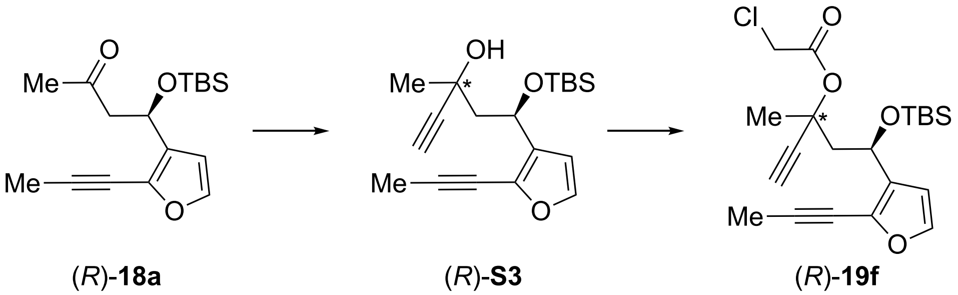

(5*R*)-5-((*tert*-butyldimethylsilyl)oxy)-3-methyl-5-(2-(prop-1-yn-1-yl)furan-3-yl)pent-1-yn-3-yl 2-chloroacetate ((*R*)-19f).

Follows general procedure **D**, (*R*)-4-((*tert*-butyldimethylsilyl)oxy)-4-(2-(prop-1-yn-1-yl)furan-3-yl)butan-2-one (*R*)-**18a** (59.2% ee) (191 mg, 0.6 mmol), ethynylmagnesium bromide (3.8 mL of a 0.5 M solution in THF, 1.9 mmol), chloroacetyl chloride (0.2 mL, 2.2 mmol), THF (2.2 mL). The reaction stirred for 5 h at rt. The crude residue was purified via silica gel flash chromatography (6% ether/hexanes) to give 151 mg (59% yield) of the title compound as a yellow oil (dr=2.5:1). TLC and ^{1}H NMR data match those of (±)-**19f**.

Enantiomeric ratio of (5*R*)-5-((*tert*-butyldimethylsilyl)oxy)-3-methyl-5-(2-(prop-1-yn-1-yl)furan-3-yl)pent-1-yn-3-yl 2-chloroacetate (*R*)-**19f** was assumed from (*R*)-4-((*tert*-butyldimethylsilyl)oxy)-4-(2-(prop-1-yn-1-yl)furan-3-yl)butan-2-one (*R*)-**18a**.

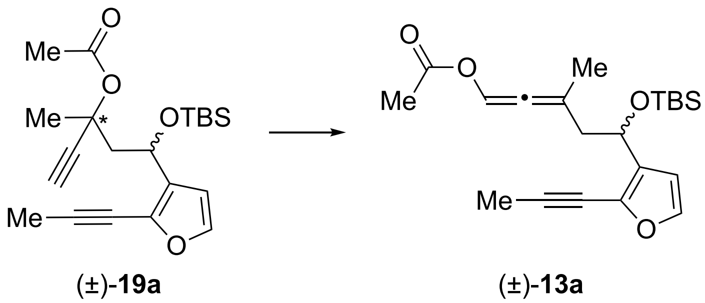

General Procedure E: Synthesis of allenyl carboxyesters via a formal [3,3]-sigmatropic rearrangement[1]

(±)-5-((*tert*-butyldimethylsilyl)oxy)-3-methyl-5-(2-(prop-1-yn-1-yl)furan-3-yl)penta-1,2-dien-1-yl acetate ((±)-13a).

A flame-dried, single-necked, 10-mL, round-bottomed flask equipped with a magnetic stir bar was charged with rhodium(II) trifluoroacetate dimer (0.05 equiv, 27 mg, 0.04 mmol) in a nitrogen-filled glovebox and sealed with a septum before removal. The flask was placed under a nitrogen atmosphere using a Schlenck line and an inlet needle. A solution of (±)-5-((*tert*-butyldimethylsilyl)oxy)-3-methyl-5-(2-(prop-1-yn-1-yl)furan-3-yl)pent-1-yn-3-yl acetate (±)-**19a** (1 equiv, 302 mg, 0.81 mmol) in toluene (4.0 mL, 0.2 M) was added dropwise via syringe over 5 min to the flask. The flask was lowered into an oil bath preheated to 50 °C. After 1 h, consumption of starting material was observed by TLC. The flask was removed from the oil bath allowed to cool to rt. SiliaMetS Thiourea (1.6 equiv, 1000 mg, 1.28 mmol) was added all at once after temporary removal of the septum. After 2.5 h, the reaction mixture was filtered through Celite® 545, and concentrated *in vacuo*. The crude residue was purified by silica gel flash chromatography (5-10% ether/hexanes) to give 242 mg (80% yield) of the title compound as a yellow oil (dr = 1:1).

^{1}H NMR (400 MHz, $CDCl_3$)

7.25–7.21 (m, 4 H), 6.40–6.38 (m, 2 H), 4.89–4.85 (m, 2 H), 2.58–2.49 (m, 2 H), 2.43–2.33 (m, 2 H), 2.12 (s, 3 H)*, 2.11 (s, 3 H)*, 2.10 (s, 3 H)*, 2.10 (s, 3 H)*, 1.85 (d, J = 2.0 Hz, 3 H)*, 1.82 (d, J = 2.0 Hz, 3 H)*, 0.85 (s, 9 H)*, 0.84 (s, 9 H)*, 0.06 (s, 3 H)*, 0.05 (s, 3 H)*, -0.12 (s, 6 H) ppm

distinguishable diastereomeric peaks *

* dr = 1:1

^{13}C NMR (100 MHz, $CDCl_3$)

191.7*, 191.3*, 168.9, 142.6*, 142.5*, 134.3*, 134.0*, 131.9*, 131.8*, 112.2*, 112.0*, 109.6*, 109.6*, 109.3*, 109.2*, 93.5, 69.4*, 69.4*, 66.0*, 65.8*, 44.8*,

44.7*, 25.9*, 25.9*, 21.1, 21.1*, 21.0*, 18.2*, 18.2*, 4.7*, 4.6*, -4.6*, -4.7*, -5.1*, -5.1* ppm

distinguishable diastereomeric peaks *

IR (Thin Film)

2954, 2929, 2857, 1979, 1749, 1251, 1213 cm^{-1}

HRMS HRMS-ESI (m/z): $[M + H]^+$ calcd for $C_{21}H_{31}O_4Si$, 375.1986; found, 375.1991

TLC R_f = 0.39 (10% ethyl acetate/hexanes); silica gel, UV, *p*-anisaldehyde

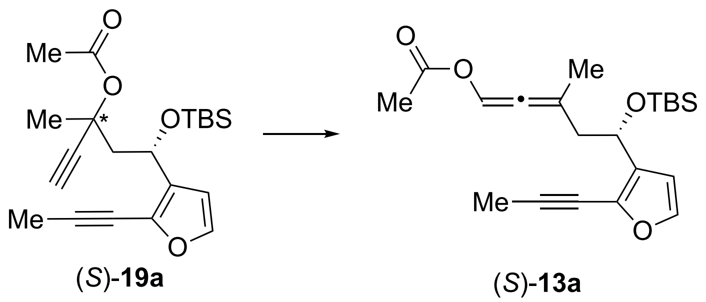

(5*S*)-5-((*tert*-butyldimethylsilyl)oxy)-3-methyl-5-(2-(prop-1-yn-1-yl)furan-3-yl)penta-1,2-dien-1-yl acetate ((*S*)-13a).

Follows general procedure **E**, rhodium(II) trifluoroacetate dimer (27 mg, 0.04 mmol), (5*S*)-5-((*tert*-butyldimethylsilyl)oxy)-3-methyl-5-(2-(prop-1-yn-1-yl)furan-3-yl)pent-1-yn-3-yl acetate (*S*)-**19a** (59.8% ee) (281 mg, 0.8 mmol), toluene (3.8 mL, 0.2 M). The reaction was stirred for 1 h in an oil bath preheated to 50 °C. SiliaMetS Thiourea (1.5 equiv, 879 mg, 1.1 mmol). After 2.5 h at rt, the reaction mixture was filtered through Celite® 545, and concentrated *in vacuo*. The crude residue was purified by silica gel flash chromatography (5-10% ether/hexanes) to give 203 mg (72% yield) of the title compound as a yellow oil (dr = 1:1). TLC and ^{1}H NMR data match those of (±)-**13a**.

Enantiomeric ratio of (5*S*)-5-((*tert*-butyldimethylsilyl)oxy)-3-methyl-5-(2-(prop-1-yn-1-yl)furan-3-yl)penta-1,2-dien-1-yl acetate (*S*)-**13a** was assumed from (*S*)-4-hydroxy-4-(2-(prop-1-yn-1-yl)furan-3-yl)butan-2-one (*S*)-**17**.

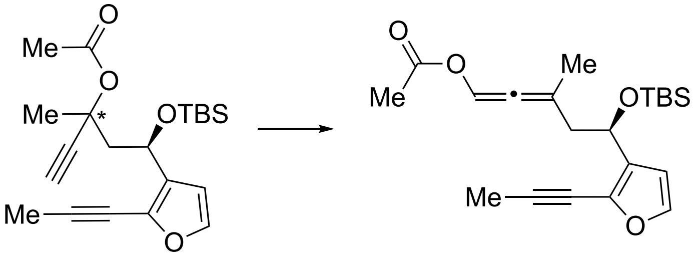

(5*R*)-5-((*tert*-butyldimethylsilyl)oxy)-3-methyl-5-(2-(prop-1-yn-1-yl)furan-3-yl)penta-1,2-dien-1-yl acetate ((*R*)-13a).

Follows general procedure **E**, rhodium(II) trifluoroacetate dimer (13 mg, 0.02 mmol), (5*R*)-5-((*tert*-butyldimethylsilyl)oxy)-3-methyl-5-(2-(prop-1-yn-1-yl)furan-3-yl)pent-1-yn-3-yl acetate (*R*)-**19a** (59.2% ee) (146 mg, 0.4 mmol), toluene (2.0 mL, 0.2 M). The reaction was stirred for 1 h in an oil bath preheated to 50 °C. SiliaMetS Thiourea (1.5 equiv, 457 mg, 0.6 mmol). After 2.5 h at rt, the reaction mixture was filtered through Celite® 545, and concentrated *in vacuo*. The crude residue was purified by silica gel flash chromatography (5-10% ether/hexanes) to give 112 mg (77% yield) of the title compound as a yellow oil (dr = 1:1). TLC and ^{1}H NMR data match those of (±)-**13a**.

Enantiomeric ratio of (5*R*)-5-((tert-butyldimethylsilyl)oxy)-3-methyl-5-(2-(prop-1-yn-1-yl)furan-3-yl)penta-1,2-dien-1-yl acetate (*R*)-**13a** was assumed from (*R*)-4-((tert-butyldimethylsilyl)oxy)-4-(2-(prop-1-yn-1-yl)furan-3-yl)butan-2-one (*R*)-**18a**.

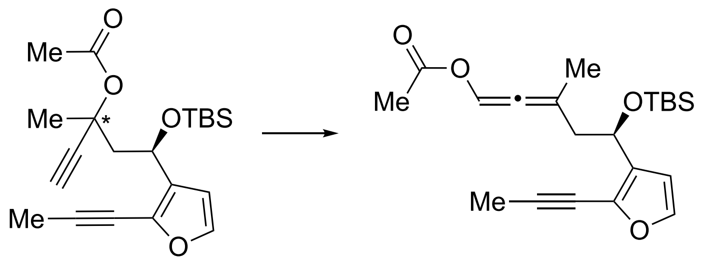

(*R*)-**19a** (*R*)-**13a**

(5*R*)-5-((*tert*-butyldimethylsilyl)oxy)-3-methyl-5-(2-(prop-1-yn-1-yl)furan-3-yl)penta-1,2-dien-1-yl acetate ((*R*)-13a).

Follows general procedure **E**, rhodium(II) trifluoroacetate dimer (4 mg, 0.006 mmol), (5*R*)-5-((*tert*-butyldimethylsilyl)oxy)-3-methyl-5-(2-(prop-1-yn-1-yl)furan-3-yl)pent-1-yn-3-yl acetate (*R*)-**19a** (≥99% ee) (46 mg, 0.1 mmol), toluene (0.6 mL, 0.2 M). The reaction was stirred for 1 h in an oil bath preheated to 50 °C. The reaction mixture was filtered through Celite® 545, and concentrated *in vacuo*. The crude residue was purified by silica gel flash chromatography (5% ether/hexanes) to give 39 mg (85% yield) of the title compound as a yellow oil (dr = 1:1). TLC and ^{1}H NMR data match those of (±)-**13a**.

Enantiomeric ratio of (5*R*)-5-((tert-butyldimethylsilyl)oxy)-3-methyl-5-(2-(prop-1-yn-1-yl)furan-3-yl)penta-1,2-dien-1-yl acetate (*R*)-**13a** was assumed from (*R*)-4-((tert-butyldimethylsilyl)oxy)-4-(2-(prop-1-yn-1-yl)furan-3-yl)butan-2-one (*R*)-**18a**.

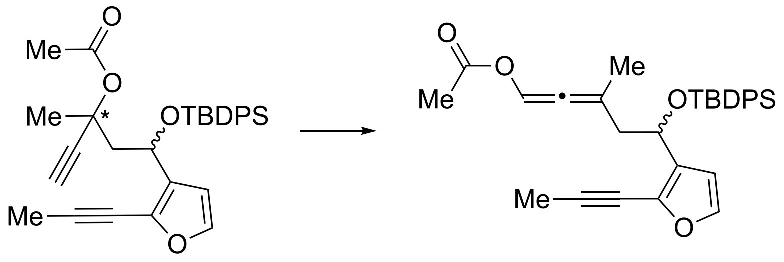

(±)-**19b** (±)-**13b**

(±)-5-((*tert*-butyldiphenylsilyl)oxy)-3-methyl-5-(2-(prop-1-yn-1-yl)furan-3-yl)penta-1,2-dien-1-yl acetate ((±)-13b).

Follows general procedure **E**, rhodium(II) trifluoroacetate dimer (6 mg, 0.009 mmol), (±)-5-((*tert*-butyldimethylsilyl)oxy)-3-methyl-5-(2-(prop-1-yn-1-yl)furan-3-yl)pent-1-yn-3-yl acetate (±)-**19b** (79 mg, 0.2 mmol), toluene (0.8 mL, 0.2 M). The reaction was stirred for 3 h in an oil bath preheated to 50 °C. SiliaMetS Thiourea (1.5 equiv, 188 mg, 0.2 mmol). After 2.5 h at rt, the reaction mixture was filtered through Celite® 545, and concentrated *in vacuo*. The crude residue was purified by silica gel flash chromatography (5% ethyl acetate /hexanes) to give 59 mg (75% yield) of the title compound as a yellow oil (dr = 1:1).

^{1}H NMR (400 MHz, $CDCl_3$)

7.70–7.67 (m, 4 H), 7.54–7.52 (m, 4 H), 7.45–7.36 (m, 8 H), 7.31–7.27 (m, 4 H), 7.18 (t, *J* = 2.0 Hz, 2 H), 7.09–7.09 (m, 1 H)*, 7.04–7.03 (m, 1 H)*, 6.42 (d, *J* = 1.6 Hz, 1 H)*, 6.41 (d, *J* = 2.0 Hz, 1 H)*, 4.91–4.88 (m, 2 H), 2.58–2.37 (m, 4 H), 2.08 (s, 3 H)*, 2.05 (s, 3 H)*, 1.93 (s, 3 H)*, 1.91 (s, 3 H)*, 1.59 (d, *J* = 2.0 Hz, 3 H)*, 1.54 (d, *J* = 2.0 Hz, 3 H)*, 1.02 (s, 9 H)*, 1.01 (s, 9 H)* ppm

distinguishable diastereomeric peaks *

* dr = 1:1

^{13}C NMR (100 MHz, $CDCl_3$)

192.0*, 191.4*, 168.8*, 168.7*, 142.4*, 142.3*, 136.1, 136.0, 134.8*, 134.6*, 134.2*, 134.2*, 133.6*, 133.5*, 131.0*, 130.9*, 129.7*, 129.7*, 129.6, 127.6, 127.4*, 127.4*, 93.0, 69.3, 66.7*, 66.5*, 44.7*, 44.6*, 27.0*, 27.0*, 21.0, 20.7*, 20.6*, 19.4, 4.6*, 4.6* ppm

distinguishable diastereomeric peaks *

IR (Thin Film)

2930, 2896, 2857, 2238, 1978, 1747, 1214 cm^{-1}

HRMS HRMS-ESI (m/z): $[M + Na]^+$ calcd for $C_{31}H_{34}O_4NaSi$, 521.2119; found, 521.2109

TLC R_f = 0.29 (10% ethyl acetate/hexanes); silica gel, UV, *p*-anisaldehyde

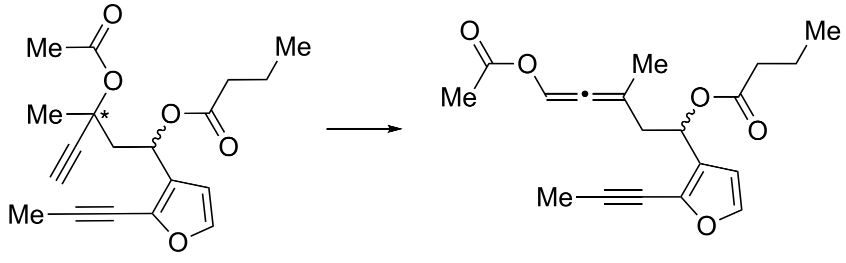

(±)-**19c** (±)-**13c**

(±)-5-acetoxy-3-methyl-1-(2-(prop-1-yn-1-yl)furan-3-yl)penta-3,4-dien-1-yl butyrate ((±)-13c).[7]

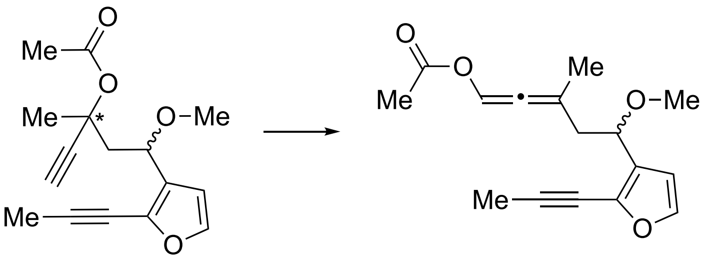

(±)-**19d** (±)-**13d**

(±)-5-methoxy-3-methyl-5-(2-(prop-1-yn-1-yl)furan-3-yl)penta-1,2-dien-1-yl acetate ((±)-13d).

Follows general procedure **E**, rhodium(II) trifluoroacetate dimer (5 mg, 0.008 mmol), 5-methoxy-(±)-3-methyl-5-(2-(prop-1-yn-1-yl)furan-3-yl)pent-1-yn-3-yl acetate (±)-**19d** (32 mg, 0.1 mmol), toluene (0.6 mL, 0.2 M). The reaction was stirred for 1.5 h in an oil bath preheated to 50 °C. SiliaMetS Thiourea (1.5 equiv, 193 mg, 0.2 mmol). After 2.5 h at rt, the reaction mixture was filtered through Celite® 545, and concentrated *in vacuo*. The crude residue was purified by silica gel flash chromatography (12% ethyl acetate /hexanes) to give 24 mg (74% yield) of the title compound as a yellow oil (dr = 1:1).

^{1}H NMR (400 MHz, $CDCl_3$)

7.31–7.27 (m, 4 H), 6.36 (d, *J* = 1.6 Hz, 2 H), 4.41–4.37 (m, 2 H), 3.20 (s, 3 H)*, 3.19 (s, 3 H)*, 2.67–2.59 (m, 2 H), 2.47–2.32 (m, 2 H), 2.13 (s, 3 H)*, 2.12 (s, 6 H)*, 2.10 (s, 3 H)*, 1.85 (d, *J* = 2.0 Hz, 3 H)*, 1.83 (d, *J* = 2.0 Hz, 3 H)* ppm

distinguishable diastereomeric peaks *

* dr = 1:1

^{13}C NMR (100 MHz, $CDCl_3$)

191.0*, 190.7*, 168.9*, 168.8*, 143.0*, 143.0*, 136.6*, 136.2*, 128.2*, 128.0*, 112.2*, 112.1*, 110.0*, 109.8*, 109.2*, 109.1*, 93.4*, 93.3*, 73.6*, 73.5*, 69.2, 56.4*, 56.4*, 41.6*, 41.5*, 21.1*, 21.1*, 21.0*, 21.0*, 4.8*, 4.7* ppm

distinguishable diastereomeric peaks *

IR (Thin Film)

2921, 1747, 1215 cm^{-1}

HRMS HRMS-ESI (m/z): $[M + Na]^+$ calcd for $C_{16}H_{18}O_4Na$, 297.1097; found, 297.1096

TLC $R_f = 0.55$ (35% ethyl acetate/hexanes); silica gel, UV, *p*-anisaldehyde

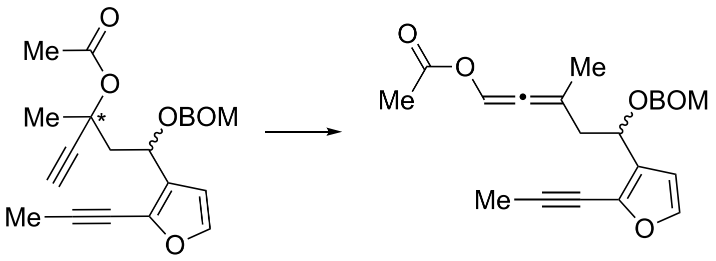

(±)-5-((benzyloxy)methoxy)-3-methyl-5-(2-(prop-1-yn-1-yl)furan-3-yl)penta-1,2-dien-1-yl acetate ((±)-13e).

Follows general procedure **E**, rhodium(II) trifluoroacetate dimer (4 mg, 0.006 mmol), (±)-5-((benzyloxy)methoxy)-3-methyl-5-(2-(prop-1-yn-1-yl)furan-3-yl)pent-1-yn-3-yl acetate (±)-**19e** (45 mg, 0.1 mmol), toluene (0.6 mL, 0.2 M). The reaction was stirred for 2 h in an oil bath preheated to 50 °C. SiliaMetS Thiourea (1.5 equiv, 141 mg, 0.2 mmol). After 2.5 h at rt, the reaction mixture was filtered through Celite® 545, and concentrated *in vacuo*. The crude residue was purified by silica gel flash chromatography (7% ethyl acetate/hexanes) to give 30 mg (67% yield) of the title compound as a white oil (dr = 1:1).

^{1}H NMR (500 MHz, $CDCl_3$)

7.34–7.26 (m, 14 H), 6.38 (s, 2 H), 4.92 (t, J = 7.0 Hz, 2 H), 4.69–4.51 (m, 8 H), 2.70–2.63 (m, 2 H), 2.52–2.41 (m, 2 H), 2.08 (s, 6 H), 2.07 (s, 3 H)*, 2.05 (s, 3 H)*, 1.86 (d, J = 1.5 Hz, 3 H)*, 1.84 (d, J = 2.0 Hz, 3 H)* ppm

distinguishable diastereomeric peaks *

* dr = 1:1

^{13}C NMR (125 MHz, $CDCl_3$)

191.4*, 191.2*, 168.8*, 168.7*, 142.9*, 142.9*, 138.2*, 138.2*, 136.4*, 136.1*, 128.5, 128.2*, 128.0*, 127.9, 127.7, 111.8*, 111.8*, 109.8*, 109.6*, 109.5*, 109.4*, 93.6*, 93.5*, 92.7*, 92.5*, 69.9*, 69.8*, 69.1*, 69.0*, 69.0*, 68.7*, 41.7*, 41.6*, 21.1*, 21.0*, 21.0, 4.7*, 4.7* ppm

distinguishable diastereomeric peaks *

IR (Thin Film)

2980, 2954, 2895, 2237, 1750, 1604, 1258, 1214, 1096, 1043 cm^{-1}

HRMS HRMS-ESI (m/z): $[M + NH_4]^+$ calcd for $C_{23}H_{28}O_5N$, 398.1938; found, 398.1960

TLC R_f = 0.55 (35% ethyl acetate/hexanes); silica gel, UV, *p*-anisaldehyde

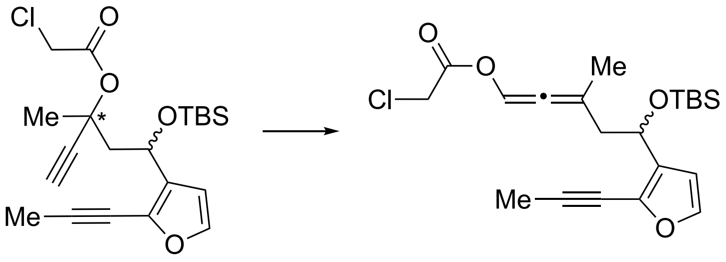

(±)-**19f** (±)-**13f**

(±)-5-((*tert*-butyldimethylsilyl)oxy)-3-methyl-5-(2-(prop-1-yn-1-yl)furan-3-yl)penta-1,2-dien-1-yl 2-chloroacetate ((±)-13f).

Follows general procedure **E**, rhodium(II) trifluoroacetate dimer (14 mg, 0.02 mmol), (±)-5-((*tert*-butyldimethylsilyl)oxy)-3-methyl-5-(2-(prop-1-yn-1-yl)furan-3-yl)pent-1-yn-3-yl 2-chloroacetate (±)-**19f** (174 mg, 0.4 mmol), toluene (2.1 mL, 0.2 M). The reaction was stirred for 4.5 h in an oil bath preheated to 50 °C. The crude residue was purified by silica gel flash chromatography (5%

ether/hexanes). SiliaMetS Thiourea (2.0 equiv, 660 mg, 0.8 mmol). After 4 h at rt, the mixture was filtered through Celite® 545, and concentrated *in vacuo*. Purification by silica gel flash chromatography (5% ether/hexanes) gave 51 mg (34% yield) of the title compound as a yellow oil (dr = 1:1).

^{1}H NMR (400 MHz, $CDCl_3$)

7.28–7.27 (m, 1 H)*, 7.23–7.21 (m, 3 H), 6.40–6.38 (m, 2 H), 4.87 (td, *J* = 6.6, 2.0 Hz, 2 H), 4.11 (s, 2 H)*, 4.11 (s, 1 H)*, 4.10 (s, 1 H)*, 2.58–2.52 (m, 2 H), 2.46–2.35 (m, 2 H), 2.12 (s, 3 H)*, 2.10 (s, 3 H)*, 1.86 (d, *J* = 2.0 Hz, 3 H)*, 1.84 (d, *J* = 2.0 Hz, 3 H)*, 0.85 (s, 18 H), 0.06 (s, 3 H)*, 0.05 (s, 3 H)*, -0.12 (s, 3 H)*, -0.12 (s, 3 H)* ppm

distinguishable diastereomeric peaks *

* dr = 1:1

^{13}C NMR (100 MHz, $CDCl_3$)

191.7*, 191.1*, 165.4*, 165.3*, 142.6*, 142.6*, 134.4*, 134.1*, 131.8*, 131.6*, 113.5*, 113.3*, 109.8*, 109.7*, 109.6*, 109.5*, 93.6*, 93.6*, 69.4*, 69.4*, 65.9*, 65.6*, 44.7*, 44.5*, 40.9*, 40.8*, 25.9*, 25.9*, 21.1, 18.2, 4.7*, 4.7*, -4.6*, -4.7*, -5.1*, -5.1* ppm

distinguishable diastereomeric peaks *

IR (Thin Film)

2955, 2930, 2857, 1771, 1660, 1253 cm^{-1}

HRMS HRMS-ESI (m/z): $[M + H]^+$ calcd for $C_{21}H_{30}O_4ClSi$, 409.1596; found, 409.1576

TLC R_f = 0.38 (10% ethyl acetate/hexanes); silica gel, UV, *p*-anisaldehyde

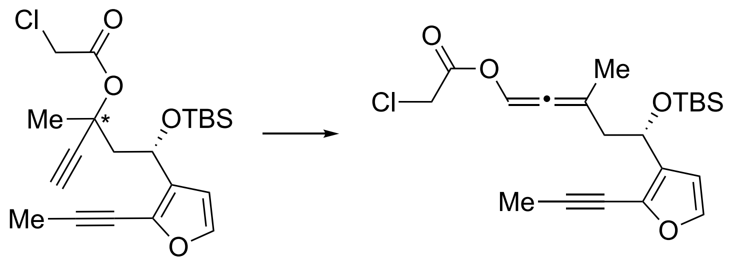

(5*S*)-5-((tert-butyldimethylsilyl)oxy)-3-methyl-5-(2-(prop-1-yn-1-yl)furan-3-yl)penta-1,2-dien-1-yl 2-chloroacetate ((*S*)-13f).

Follows general procedure **E**, rhodium(II) trifluoroacetate dimer (13 mg, 0.02 mmol), (5*S*)-5-((tert-butyldimethylsilyl)oxy)-3-methyl-5-(2-(prop-1-yn-1-yl)furan-3-yl)pent-1-yn-3-yl 2-chloroacetate (*S*)-**19f** (59.8% ee) (138 mg, 0.4 mmol), toluene (1.9 mL, 0.2 M). The reaction was stirred for 2.5 h in an oil bath preheated to 50 °C. SiliaMetS Thiourea (1.5 equiv, 437 mg, 0.6 mmol). After 2 h at rt, the reaction mixture was filtered through Celite® 545, and concentrated *in vacuo*. The crude residue was purified by silica gel flash chromatography (5% ether/hexanes) to give 83 mg (60% yield) of the title compound as a yellow oil (dr = 1:1). TLC and ^{1}H NMR data match those of (±)-**13f**.

Enantiomeric ratio of (5*S*)-5-((tert-butyldimethylsilyl)oxy)-3-methyl-5-(2-(prop-1-yn-1-yl)furan-3-yl)penta-1,2-dien-1-yl 2-chloroacetate (*S*)-**13f** was assumed from (*S*)-4-hydroxy-4-(2-(prop-1-yn-1-yl)furan-3-yl)butan-2-one (*S*)-**17**.

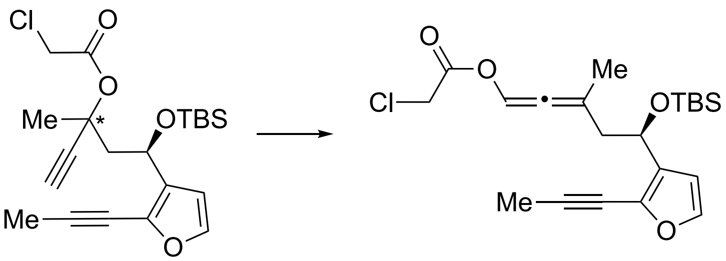

(*R*)-**19f** (*R*)-**13f**

(5*R*)-5-((tert-butyldimethylsilyl)oxy)-3-methyl-5-(2-(prop-1-yn-1-yl)furan-3-yl)penta-1,2-dien-1-yl 2-chloroacetate ((*R*)-13f).

Follows general procedure **E**, rhodium(II) trifluoroacetate dimer (15 mg, 0.02 mmol), (5*R*)-5-((tert-butyldimethylsilyl)oxy)-3-methyl-5-(2-(prop-1-yn-1-yl)furan-3-yl)pent-1-yn-3-yl 2-chloroacetate (*R*)-**19f** (59.2% ee) (155 mg, 0.4 mmol), toluene (2.0 mL, 0.2 M). The reaction was stirred for 4 h in an oil bath preheated to 50 °C. The crude residue was purified by silica gel flash chromatography (5% ether/hexanes). SiliaMetS Thiourea (2.0 equiv, 600 mg, 0.8 mmol). After 4 h at rt, the mixture was filtered through Celite® 545, and concentrated *in vacuo*. Purification by silica gel flash chromatography (5% ether/hexanes) gave 48 mg (31% yield) of the title compound as a yellow oil (dr = 1:1). TLC and ^{1}H NMR data match those of (±)-**13f**.

Enantiomeric ratio of (5*R*)-5-((tert-butyldimethylsilyl)oxy)-3-methyl-5-(2-(prop-1-yn-1-yl)furan-3-yl)penta-1,2-dien-1-yl 2-chloroacetate (*R*)-**13f** was assumed from (*R*)-4-((tert-butyldimethylsilyl)oxy)-4-(2-(prop-1-yn-1-yl)furan-3-yl)butan-2-one (*R*)-**18a**.

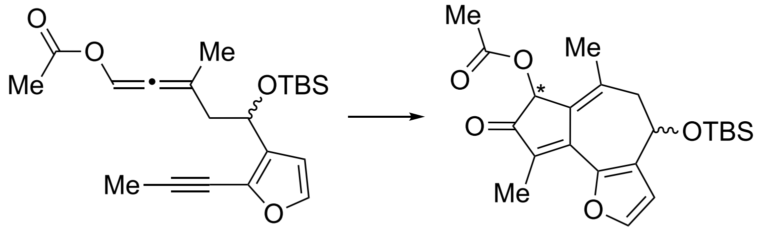

(±)-**13a** **14a**

General Procedure F: Synthesis of 5,7,5-ring system via racemic allenic Pauson-Khand reaction (PKR) using triphenylphosphine[1]

Table 1, entry 1

4-((*tert*-butyldimethylsilyl)oxy)-6,9-dimethyl-8-oxo-4,5,7,8-tetrahydroazuleno[4,5-b]furan-7-yl acetate (14a).

Bis(1,5-cyclooctadiene)rhodium(I) tetrafluoroborate (0.1 equiv, 2.7 mg, 0.0067 mmol) and triphenylphosphine (0.15 equiv, 2.6 mg, 0.01 mmol) were weighed into separate flame-dried 5-mL round-bottomed flasks in a nitrogen-filled glovebox and each sealed with a septum before removing. The flasks were placed under a nitrogen atmosphere via a Schlenk line and an inlet needle. Bis(1,5- cyclooctadiene)rhodium(I) tetrafluoroborate and triphenylphosphine were each dissolved in DCE (0.3 mL each) and the resulting solutions were transferred via syringe to a flame-dried, 25-mL, Schlenk tube and stirred under nitrogen for 30 min at rt. The tube was evacuated via the inlet needle for a few seconds and refilled (3 x) with carbon monoxide (100%) using a separate inlet needle attached to a balloon of CO and stirred for 1 h. Hexamethylbenzene (1 equiv, 10.9 mg, 0.067 mmol in 0.3 mL DCE) was added via syringe to the tube followed by (±)-5-((*tert*-butyldimethylsilyl)oxy)-3-methyl-5-(2-(prop-1-yn-1-yl)furan-3-yl)penta-1,2-dien-1-yl acetate (±)-**13a** (1 equiv, 25 mg, 0.067 mmol) in DCE (0.02 M, 2.4 mL) dropwise via syringe. The tube was lowered into an oil bath preheated to 70 °C and stirred until TLC showed complete

consumption of starting material (112 h). The solution was transferred to a 25-mL round bottomed flask, rinsed with ethyl acetate and concentrated *in vacuo.* The crude residue was purified by silica gel flash chromatography (4-5% ethyl acetate/hexanes) to give 11.4 mg (42% yield) of the title compound as a yellow oil (dr (*trans*:*cis*) = 1:1.7 (37:63)).

Note: The ratio of PKR product **14a** to aldehyde byproduct **S7** was 1:0.1 (91:9) based on crude ^{1}H NMR.

NMR yield: 36%

^{1}H NMR (400 MHz, $CDCl_3$)

7.56–7.56 (m, 2 H), 6.59 (d, J = 1.6 Hz, 1 H)*, 6.58 (d, J = 1.6 Hz, 1 H)**, 5.84 (s, 1 H)**, 5.82 (s, 1 H)*, 4.93 (dd, J = 10.8, 2.8 Hz, 1 H)*, 4.89 (dd, J = 10.6, 2.0 Hz, 1 H)**, 2.94–2.80 (m, 2 H), 2.52 (dd, J = 16.2, 2.8 Hz, 1 H)*, 2.43 (dd, J = 15.2, 2.4 Hz, 1 H)**, 2.20 (s, 3 H)**, 2.18 (s, 3 H)*, 2.16 (s, 3 H)*, 2.14 (s, 3 H)**, 1.94 (s, 3 H)**, 1.92 (s, 3 H)*, 0.95 (s, 9 H)*, 0.94 (s, 9 H)**, 0.14 (s, 6 H), 0.11 (s, 3 H)*, 0.11 (s, 3 H)** ppm

distinguishable diastereomeric peaks for minor isomer (*trans*) *

distinguishable diastereomeric peaks for major isomer (*cis*) **

* dr = 1:1.7 (37:63)

^{13}C NMR (100 MHz, $CDCl_3$)

199.6*, 199.4**, 170.0**, 169.9*, 149.0*, 148.8**, 144.9*, 144.8**, 144.6**, 144.3*, 137.0**, 136.9*, 134.2*, 133.1**, 132.9**, 132.8*, 128.3**, 127.8*, 112.1**, 111.9*, 71.8**, 71.4*, 65.6**, 65.5*, 45.0*, 44.7**, 25.9*, 25.9**, 25.2**, 25.1*, 20.9*, 20.8**, 18.4, 10.3**, 10.1*, -4.6*, -4.6**, -4.7*, -4.7** ppm

distinguishable diastereomeric peaks for minor isomer (*trans*) *

distinguishable diastereomeric peaks for major isomer (*cis*) **

IR (Thin Film)

2929, 2857, 1747, 1700, 1255, 1221 cm-1

HRMS HRMS-ESI (m/z): $[M + Na]^+$ calcd for $C_{22}H_{30}O_5NaSi$, 425.1755; found, 425.1741

TLC R_f = 0.53 (35% ethyl acetate/hexanes); silica gel, UV, *p*-anisaldehyde

Shimadzu Nexera Series SCL-40, UV/PDA detector, 328 nm, CHIRALPAK IA-3, 250 X 4.6 mm column, 0.1% *i*PrOH/hexanes, Flow rate: 1 mL/min, 40 °C

Peak	Retention Time (min)	Peak area (%)
Peak 1	22.56	17.10
Peak 2	25.92	18.40
Peak 3	31.89	31.44
Peak 4	36.78	33.06

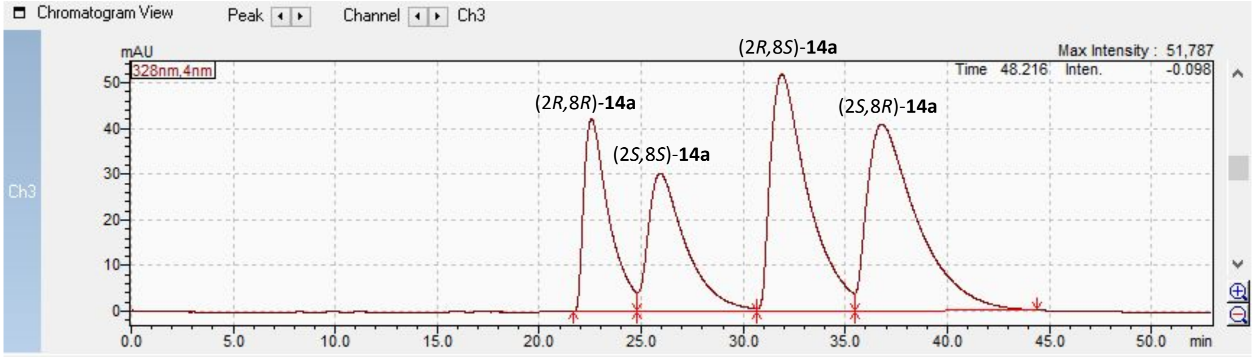

Results View - Peak Table

Peak Table | Compound | Group | Calibration Curve

Peak#	Ret. Time	Area	Height	Mark	Area%
1	22.561	3585244	42074		17.097
2	25.922	3858744	30229	V	18.402
3	31.887	6592186	51700	V	31.437
4	36.784	6933297	40736	V	33.064
Total		20969471	164738		100.000

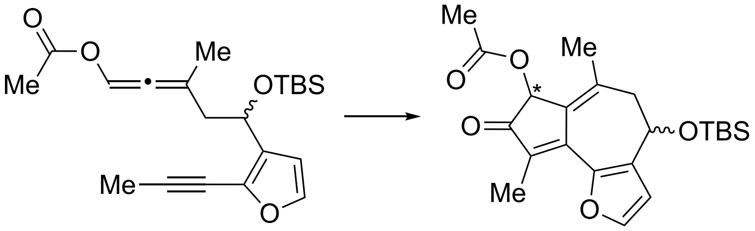

(±)-**13a** **14a**

Table 1, entry 2 (high concentration)

4-((*tert*-butyldimethylsilyl)oxy)-6,9-dimethyl-8-oxo-4,5,7,8-tetrahydroazuleno[4,5-b]furan-7-yl acetate (14a).

Follows general procedure **F**, Bis(1,5-cyclooctadiene)rhodium(I) tetrafluoroborate (2.7 mg, 0.007 mmol), triphenyl phosphine(2.6 mg, 0.01 mmol), carbon monoxide (100%), (±)-5-((*tert*-butyldimethylsilyl)oxy)-3-methyl-5-(2-(prop-1-yn-1-yl)furan-3-yl)penta-1,2-dien-1-yl acetate (±)-**13a** (25 mg, 0.07 mmol), hexamethylbenzene (1.1 mg, 0.007 mmol), DCE (0.7 mL, 0.1 M). The reaction was stirred for 20 h in an oil bath preheated to 70 °C. The crude residue was purified via silica gel flash chromatography (3-5% ethyl acetate/hexanes) to give 7 mg (26% yield) of the

title compound as a yellow oil (dr (*trans*:*cis*) = 1:2 (33:66)). TLC and ^{1}H NMR data match those of **14a**.

Note: The ratio of PKR product **14a** to aldehyde byproduct **S7** was 1:0.8 (56:44) based on crude ^{1}H NMR.

NMR yield: 38%

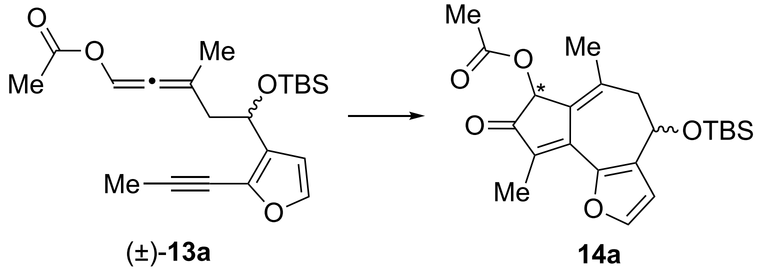

General Procedure G: Synthesis of 5,7,5-ring system via racemic PKR using (±)-MonoPhos-alkene[1]

Table 1, entry 7

4-((tert-butyldimethylsilyl)oxy)-6,9-dimethyl-8-oxo-4,5,7,8-tetrahydroazuleno[4,5-b]furan-7-yl acetate (14a).

(±)-5-((*tert*-butyldimethylsilyl)oxy)-3-methyl-5-(2-(prop-1-yn-1-yl)furan-3-yl)penta-1,2-dien-1-yl acetate (±)-**13a** (15 mg, 0.04 mmol) was diluted with toluene and concentrated using high vacuum (3 x 0.5 mL). Bis(1,5-cyclooctadiene)rhodium(I) tetrafluoroborate (0.1 equiv, 1.6 mg, 0.004 mmol) and (±)-MonoPhos-alkene ((±)-**4** (0.3 equiv, 4.9 mg, 0.01 mmol) were weighed into separate flame-dried 5-mL round-bottomed flasks in a nitrogen-filled glovebox and each sealed with a septum before removing. The flasks were placed under a nitrogen atmosphere via a Schlenk

line and an inlet needle. Bis(1,5-cyclooctadiene)rhodium(I) tetrafluoroborate and (±)-MonoPhos-alkene (±)-**4** were each dissolved in DCE (0.3 mL each) and the resulting solutions transferred via syringe to a flame dried, 25-mL, Schlenk tube and stirred under nitrogen for 30 min at rt. The tube was evacuated via the inlet needle for a few seconds and refilled (3 x) with carbon monoxide (100%) using a separate inlet needle attached to a balloon of CO and stirred for 1 h. Hexamethylbenzene (0.1 equiv, 0.6 mg, 0.004 mmol in 0.3 mL DCE) was added via syringe to the tube followed by (±)-5-((tert-butyldimethylsilyl)oxy)-3-methyl-5-(2-(prop-1-yn-1-yl)furan-3-yl)penta-1,2-dien-1-yl acetate (±)-**13a** (1 equiv, 15 mg, 0.04 mmol) in DCE (0.01 M, 3.1 mL) dropwise via syringe. The tube was lowered into an oil bath preheated to 70 °C and stirred until TLC showed complete consumption of starting material (165 h). The solution was transferred to a 25-mL round-bottomed flask, rinsed with ethyl acetate and concentrated *in vacuo.* The crude residue was purified by silica gel flash chromatography (4-5% ethyl acetate/hexanes) to give 3.5 mg (40% yield) of the title compound as a yellow oil (dr (*trans*:*cis*) = 1.5:1 (60:40)). TLC and ^{1}H NMR data match those of **14a**.

Note: The ratio of PKR product **14a** to aldehyde byproduct **S7** was 1:0.2 (83:17) based on crude ^{1}H NMR.

Note: Three aliquots (2.2 mL) were taken out of the reaction and considered for calculating isolated yield.

NMR yield: 26%

Shimadzu Nexera Series SCL-40, UV/PDA detector, 328 nm, CHIRALPAK IB N-3, 150 X 4.6 mm column,, 0.1% EtOH/hexanes, Flow rate: 1 mL/min

Peak	Retention Time (min)	Peak area (%)
Peak 1	33.78	29.12
Peak 2	36.16	21.21
Peak 3	52.81	30.09
Peak 4	58.49	19.57

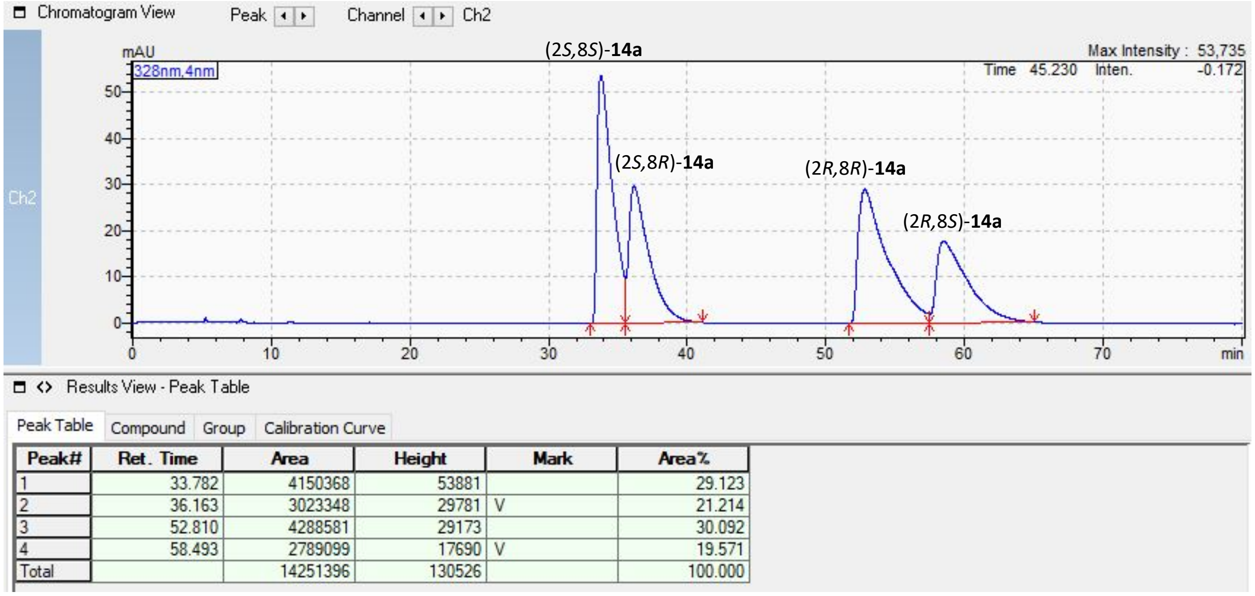

Peak#	Ret. Time	Area	Height	Mark	Area%
1	33.782	4150368	53881		29.123
2	36.163	3023348	29781	V	21.214
3	52.810	4288581	29173		30.092
4	58.493	2789099	17690	V	19.571
Total		14251396	130526		100.000

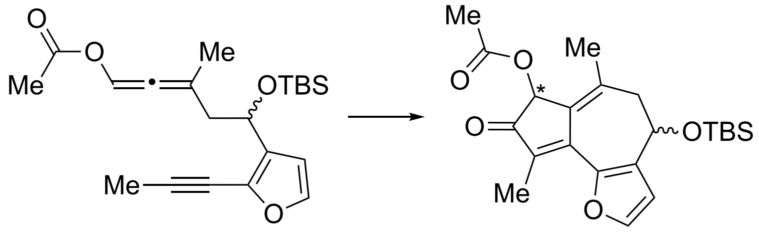

(±)-**13a** **14a**

General Procedure H: Synthesis of 5,7,5-ring system via a ligand-free PKR[10]

Table 1, entry 8

4-((*tert*-butyldimethylsilyl)oxy)-6,9-dimethyl-8-oxo-4,5,7,8-tetrahydroazuleno[4,5-b]furan-7-yl acetate (14a).

Rhodium biscarbonyl chloride dimer (0.05 equiv, 1.1 mg, 0.003 mmol) was weighed into a flame-dried 5-mL round-bottomed flask in a nitrogen-filled glovebox and sealed with a septum before removing. The flask was placed under a nitrogen atmosphere via a Schlenk line and an inlet needle. Rhodium biscarbonyl chloride dimer was dissolved in toluene (0.001 M, 2.2 mL) and the resulting solution was transferred via syringe to a flame-dried, 2-necked, 10-mL round-bottomed flask equipped with a magnetic stir bar, a condenser topped with a septum pierced with a nitrogen inlet needle, and a septum in the side arm. The apparatus was evacuated via the inlet needle for a few seconds and refilled (3 x) with carbon monoxide (10%) using a separate inlet needle attached to a balloon of CO. The flask was lowered into an oil bath preheated to 110 °C. In a separate 5-mL round-bottomed flask, (±)-5-((tert-butyldimethylsilyl)oxy)-3-methyl-5-(2-(prop-1-yn-1-yl)furan-3-yl)penta-1,2-dien-1-yl acetate (±)-**13a** (1 equiv, 21 mg, 0.06 mmol) was dissolved in toluene (0.04 M, 1.4 mL). The resulting solution was added to the reaction flask via syringe, dropwise, over 1.5 h using a syringe pump. After 48 h at 110 °C, a trace amount of product was observed by TLC. ^{1}H NMR showed a ratio of 9:91 for pdt:SM **14a**:**13a** based on peaks at 6.59, 6.39 ppm (dr (*trans:cis*) = 1:1 (50:50)). TLC and ^{1}H NMR data match those of **14a**.

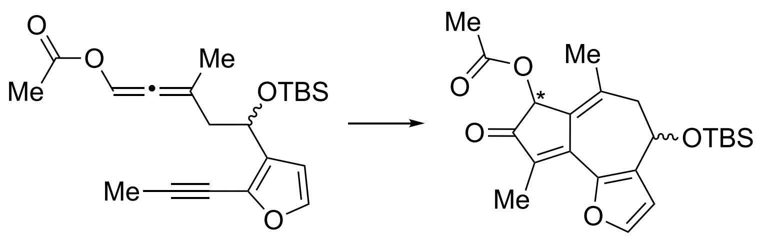

(±)-**13a** **14a**

Table 1, entry 9 (higher catalyst loading)

4-((tert-butyldimethylsilyl)oxy)-6,9-dimethyl-8-oxo-4,5,7,8-tetrahydroazuleno[4,5-b]furan-7-yl acetate (14a).

Follows general procedure **H**, Rhodium biscarbonyl chloride dimer (1.0 mg, 0.003 mmol), carbon monoxide (10%), (±)-5-((tert-butyldimethylsilyl)oxy)-3-methyl-5-(2-(prop-1-yn-1-yl)furan-3-yl)penta-1,2-dien-1-yl acetate (±)-**13a** (5 mg, 0.01 mmol), mesitylene (0.002 mL, 0.01 mmol), toluene-d_8 (0.7 mL, 0.02 M). After 73 h at 110 °C, a trace amount of product was observed by TLC. ^{1}H-NMR showed a ratio of 17:83 for pdt:SM **14a**:**13a** based on peaks at 6.59, 6.39 ppm (dr (*trans:cis*) = 1.4:1 (58:42)). TLC and ^{1}H NMR data match those of **14a**.

NMR yield: 7%

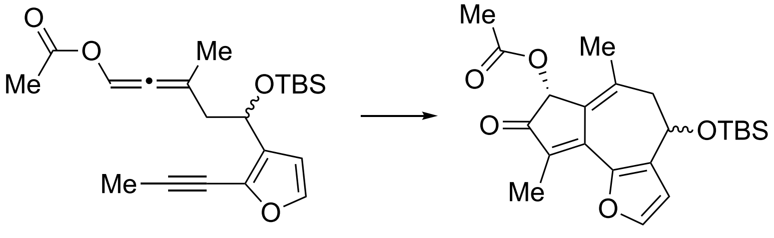

(±)-**13a** **14a**

General Procedure I: Synthesis of 5,7,5-ring system via asymmetric PKR[1]

Table 2, entry 1

(7*R*)-4-((*tert*-butyldimethylsilyl)oxy)-6,9-dimethyl-8-oxo-4,5,7,8-tetrahydroazuleno[4,5-b]furan-7-yl acetate (14a).

(±)-5-((tert-butyldimethylsilyl)oxy)-3-methyl-5-(2-(prop-1-yn-1-yl)furan-3-yl)penta-1,2-dien-1-yl acetate (±)-**13a** (25 mg, 0.067 mmol) was diluted with toluene and concentrated using high

vacuum (1 x 0.5 mL). Bis(1,5-cyclooctadiene)rhodium(I) tetrafluoroborate (0.1 equiv, 2.7 mg, 0.0067 mmol) and (*S*)-MonoPhos-alkene (*S*)-**4** (0.15 equiv, 4.1 mg, 0.01 mmol) were weighed into separate flame-dried 5-mL round-bottomed flasks in a nitrogen-filled glovebox and each sealed with a septum before removing. The flasks were placed under a nitrogen atmosphere via a Schlenk line and an inlet needle. Bis(1,5- cyclooctadiene)rhodium(I) tetrafluoroborate and (*S*)-MonoPhos-alkene (*S*)-**4** were each dissolved in DCE (0.3 mL each) and the resulting solutions transferred via syringe to a flame dried, 25-mL Schlenk tube and stirred under nitrogen for 30 min at rt. The tube was evacuated via the inlet needle for a few seconds and refilled (3 x) with carbon monoxide (100%) using a separate inlet needle attached to a balloon of CO and stirred for 1 h. Hexamethylbenzene (0.1 equiv, 1.1 mg, 0.007 mmol in 0.3 mL DCE) was added via syringe to the tube followed by (±)-5-((tert-butyldimethylsilyl)oxy)-3-methyl-5-(2-(prop-1-yn-1-yl)furan-3-yl)penta-1,2-dien-1-yl acetate (±)-**13a** (1 equiv, 25 mg, 0.067 mmol) in DCE (0.01 M, 5.8 mL) dropwise via syringe. The tube was lowered into an oil bath preheated to 70 °C and stirred until TLC showed complete consumption of starting material (26 h). The solution was transferred to a 25-mL round-bottomed flask, rinsed with ethyl acetate, and concentrated *in vacuo.* The crude residue was purified by silica gel flash chromatography (4-5% ethyl acetate/hexanes) to give 9 mg (33% yield) of the title compound as a yellow oil (dr (*trans*:*cis*) = 1.5:1 (60:40)) [er = 91.5:8.5 (2*R,*8*R*:2*S,*8*R*) and 82.4:17.6 (2*R,*8*S*:2*S,*8*S*) respectively]. TLC and ^{1}H NMR data match those of **14a**.

^{1}H NMR (700 MHz, $CDCl_3$)

7.57–7.56 (m, 2 H), 6.59 (d, *J* = 2.1 Hz, 1 H)**, 6.58 (d, *J* = 1.4 Hz, 1 H)*, 5.84 (s, 1 H)*, 5.82 (s, 1 H)**, 4.93 (dd, *J* = 10.5, 2.8 Hz, 1 H)**, 4.89 (dd, *J* = 10.5, 2.8 Hz, 1 H)*, 2.91 (dd, *J* = 14.4, 10.5 Hz, 1 H)*, 2.84 (dd, *J* = 15.8, 10.5 Hz, 1 H)**,

2.52 (dd, J = 16.4, 2.8 Hz, 1 H)**, 2.42 (dd, J = 14.7, 2.8 Hz, 1 H)*, 2.20 (s, 3 H)*, 2.19 (s, 3 H)**, 2.16 (s, 3 H)**, 2.14 (s, 3 H)*, 1.94 (s, 3 H)*, 1.92 (s, 3 H)**, 0.95 (s, 9 H)**, 0.94 (s, 9 H)*, 0.14 (s, 3 H)*, 0.14 (s, 3 H)**, 0.12 (s, 3 H)**, 0.11 (s, 3 H)* ppm

distinguishable diastereomeric peaks for minor isomer (*cis*) *

distinguishable diastereomeric peaks for major isomer (*trans*) **

* dr = 1.5:1 (60:40)

^{13}C NMR (175 MHz, $CDCl_3$)

199.6**, 199.5*, 170.0*, 170.0**, 149.1**, 148.8*, 145.0**, 144.9*, 144.5*, 144.2**, 137.1*, 137.0**, 134.3**, 133.0*, 133.0*, 132.7**, 128.3*, 127.7**, 112.1*, 111.9**, 71.8*, 71.3**, 65.6*, 65.5**, 45.0**, 44.6*, 25.9**, 25.9*, 25.3*, 25.2**, 21.0**, 20.8*, 18.4*, 18.4**, 10.3*, 10.1**, -4.6**, -4.6*, -4.7**, -4.7* ppm

distinguishable diastereomeric peaks for minor isomer (*cis*)*

distinguishable diastereomeric peaks for major isomer (*trans*) **

Shimadzu Nexera Series SCL-40, UV/PDA detector, 328 nm, CHIRALPAK IA-3, 250 X 4.6 mm column, 0.1% *i*PrOH/hexanes, Flow rate: 1 mL/min, 40 °C

Peak	Retention Time (min)	Peak area (%)
Peak 1	12.49	52.50
Peak 2	15.18	6.03
Peak 3	16.06	36.59
Peak 4	18.85	4.88

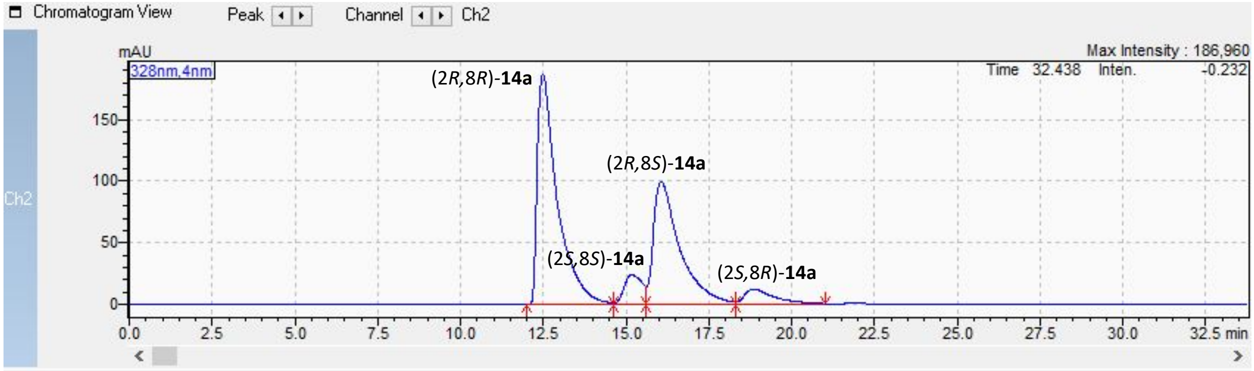

Results View - Peak Table

Peak Table | Compound | Group | Calibration Curve

Peak#	Ret. Time	Area	Height	Mark	Area%
1	12.490	7865023	186977		52.500
2	15.183	903962	23700	V	6.034
3	16.061	5481033	99588	V	36.587
4	18.853	730961	11907	V	4.879
Total		14980979	322172		100.000

Deconvolution for the two middle peaks:

Calculated peak area for peaks 2 and 3 after deconvolution

Peak	Retention Time (min)	Peak area (%)
Peak 2	15.18	7.51
Peak 3	16.07	35.13

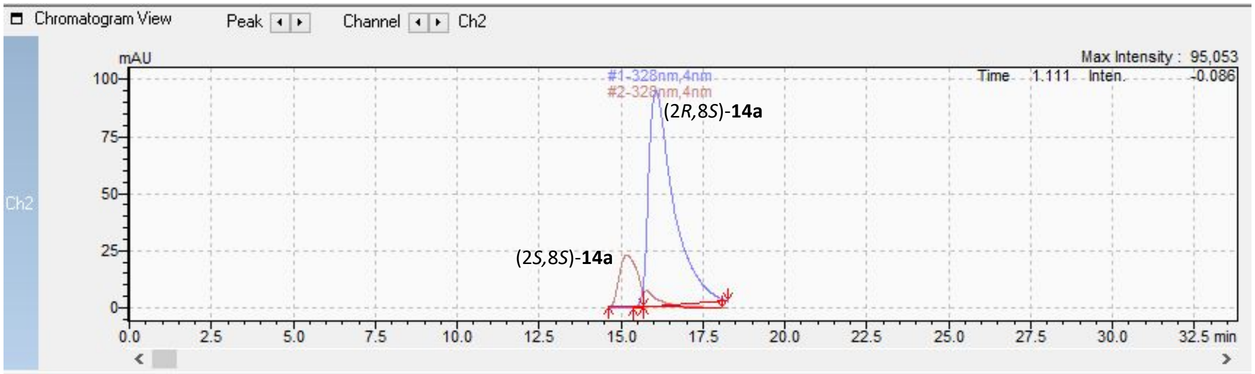

Results View - Peak Table

Peak Table | Compound | Group | Calibration Curve

Peak#	Ret. Time	Area	Height	Mark	Area%
1	15.183	863888	22692	D	14.148
2	15.778	210940	7000	V D	3.455
3	16.069	5031265	94359	D	82.397
Total		6106093	124051		100.000

Asymmetric PKR with low concentration of CO (10% and 1%)

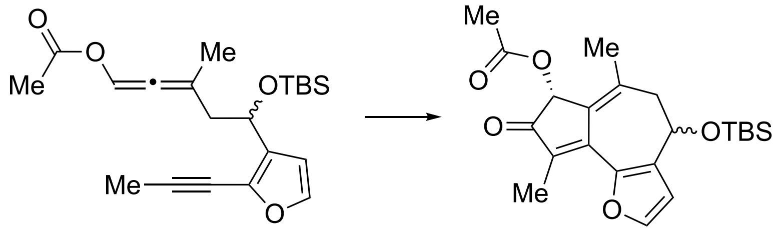

(±)-**13a** **14a**

(7*R*)-4-((*tert*-butyldimethylsilyl)oxy)-6,9-dimethyl-8-oxo-4,5,7,8-tetrahydroazuleno[4,5-b]furan-7-yl acetate (14a).

Follows general procedure **I**, bis(1,5-cyclooctadiene)rhodium(I) tetrafluoroborate (1.7 mg, 0.004 mmol), (*S*)-MonoPhos-alkene (*S*)-**4** (2.6 mg, 0.006 mmol), carbon monoxide (10%), (±)-5-((tert-butyldimethylsilyl)oxy)-3-methyl-5-(2-(prop-1-yn-1-yl)furan-3-yl)penta-1,2-dien-1-yl acetate (±)-**13a** (16 mg, 0.04 mmol), hexamethylbenzene (0.7 mg, 0.004 mmol), DCE (4.3 mL, 0.01 M). The reaction was stirred for 21 h in an oil bath preheated to 70 °C. The crude residue was purified via silica gel flash chromatography (4-5% ethyl acetate/hexanes) to give 6 mg (37% yield) of the

title compound as a yellow oil (dr (*trans*:*cis*) = 1.4:1 (58:42)) [er = 96.3:3.7 (2*R*,8*R*:2*S*,8*R*) and 79.4:20.6 (2*R*,8*S*:2*S*,8*S*) respectively]. TLC and ^{1}H NMR data match those of **14a**.

Note: One aliquot (0.6 mL) was taken out of the reaction and considered for calculating isolated yield.

NMR yield: 37%

Shimadzu Nexera Series SCL-40, UV/PDA detector, 328 nm, CHIRALPAK IA-3, 250 X 4.6 mm column, 0.1% *i*PrOH/hexanes, Flow rate: 1 mL/min, 40 °C

Peak	Retention Time (min)	Peak area (%)
Peak 1	25.88	50.08
Peak 2	29.36	9.92
Peak 3	38.11	38.11
Peak 4	44.95	1.90

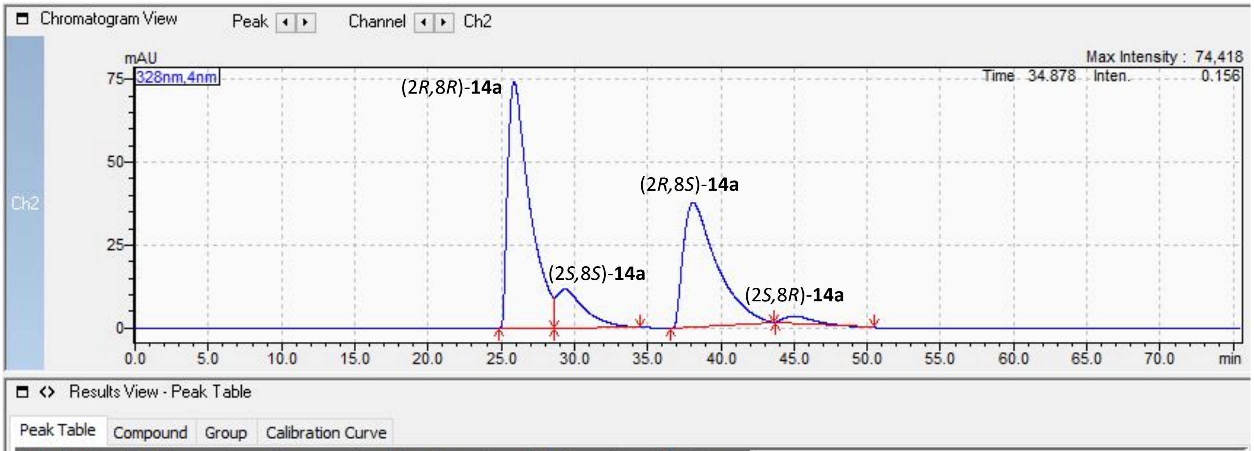

Peak#	Ret. Time	Area	Height	Mark	Area%
1	25.885	7796899	74436		50.075
2	29.361	1543730	11617	V	9.915
3	38.106	5934118	37383	M	38.112
4	44.951	295549	2119	M	1.898
Total		15570296	125555		100.000

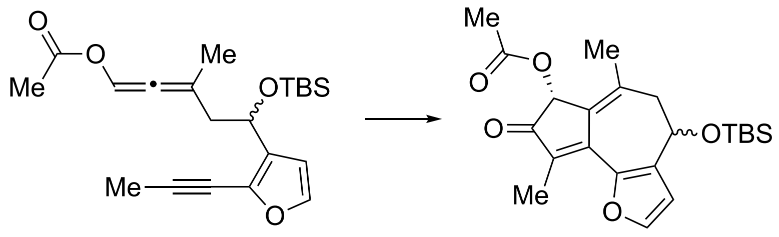

(±)-**13a** **14a**

(7*R*)-4-((*tert*-butyldimethylsilyl)oxy)-6,9-dimethyl-8-oxo-4,5,7,8-tetrahydroazuleno[4,5-b]furan-7-yl acetate (14a).

Follows general procedure **I**, Bis(1,5-cyclooctadiene)rhodium(I) tetrafluoroborate (1.2 mg, 0.003 mmol), (*S*)-MonoPhos-alkene (*S*)-**4** (1.8 mg, 0.004 mmol), carbon monoxide (1%), (±)-5-((tert-butyldimethylsilyl)oxy)-3-methyl-5-(2-(prop-1-yn-1-yl)furan-3-yl)penta-1,2-dien-1-yl acetate (±)-**13a** (11 mg, 0.03 mmol), hexamethylbenzene (0.5 mg, 0.003 mmol), DCE (2.9 mL, 0.01 M). The reaction was stirred for 21 h in an oil bath preheated to 70 °C. The crude residue was purified via silica gel flash chromatography (4% ethyl acetate/hexanes) to give 2 mg (23% yield) of the title compound as a yellow oil (dr (*trans*:*cis*) = 1.3:1 (56:44)) [er = 88.3:12 (2*R,*8*R*:2*S,*8*R*) and 87.3:12.7 (2*R,*8*S*:2*S,*8*S*) respectively]. TLC and ^{1}H NMR data match those of **14a**.

Note: Two aliquots (0.8 mL) were taken out of the reaction and considered for calculating isolated yield.

NMR yield: 31%

Shimadzu Nexera Series SCL-40, UV/PDA detector, 328 nm, CHIRALPAK IA-3, 250 X 4.6 mm column, 0.1% *i*PrOH/hexanes, Flow rate: 1 mL/min, 40 °C

Peak	Retention Time (min)	Peak area (%)

Peak 1	12.61	45.94
Peak 2	15.26	6.09
Peak 3	16.12	41.90
Peak 4	18.82	6.07

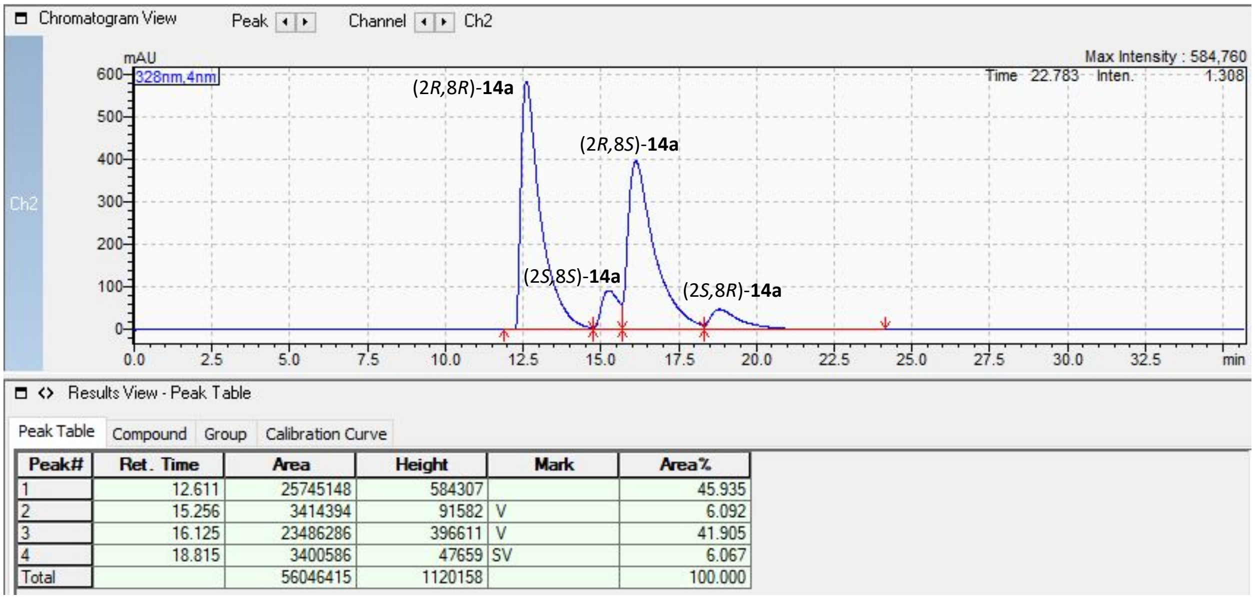

Large scale asymmetric PKR and removal of rhodium catalyst with polymer-bound PPh_3

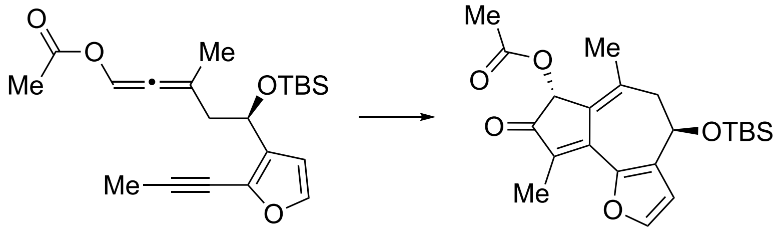

(8*R*)-**13a** (2*R*,8*R*)-**14a**

Table 2, entry 2

(4*R*,7*R*)-4-((*tert*-butyldimethylsilyl)oxy)-6,9-dimethyl-8-oxo-4,5,7,8-tetrahydroazuleno[4,5-b]furan-7-yl acetate ((2*R*,8*R*)-14a).

Follows general procedure **I**, bis(1,5-cyclooctadiene)rhodium(I) tetrafluoroborate (12.1 mg, 0.03 mmol), (*S*)-MonoPhos-alkene (*S*)-**4** (18.5 mg, 0.04 mmol), carbon monoxide (100%), (5*R*)-5-((tert-butyldimethylsilyl)oxy)-3-methyl-5-(2-(prop-1-yn-1-yl)furan-3-yl)penta-1,2-dien-1-yl acetate (*R*)-**13a** (59.2% ee) (112 mg, 0.3 mmol), DCE (30.0 mL, 0.01 M). The reaction was stirred for 22 h in an oil bath preheated to 70 °C. Polymer-bound triphenylphosphine (100 mg, 1.0 eq, 3 mmol/g). After 5 h at rt, the reaction mixture was filtered, and concentrated *in vacuo*. The crude residue was purified via silica gel flash chromatography (5-10% ethyl acetate/hexanes) to give 59 mg (49% yield) of the title compound as a yellow oil (dr (*trans*:*cis*) = 4.7:1 (82:18)) [er = 92.1:7.9 (2*R,*8*R*:2*S,*8*R*) and 83.9:16.1 (2*R,*8*S*:2*S,*8*S*) respectively]. TLC and ^{1}H NMR data match those of **14a**.

Shimadzu Nexera Series SCL-40, UV/PDA detector, 328 nm, CHIRALPAK IA-3, 250 X 4.6 mm column, 0.1% *i*PrOH/hexanes, Flow rate: 1 mL/min, 40 °C

Peak	Retention Time (min)	Peak area (%)
Peak 1	28.25	83.18
Peak 2	41.59	11.31
Peak 3	48.13	5.51

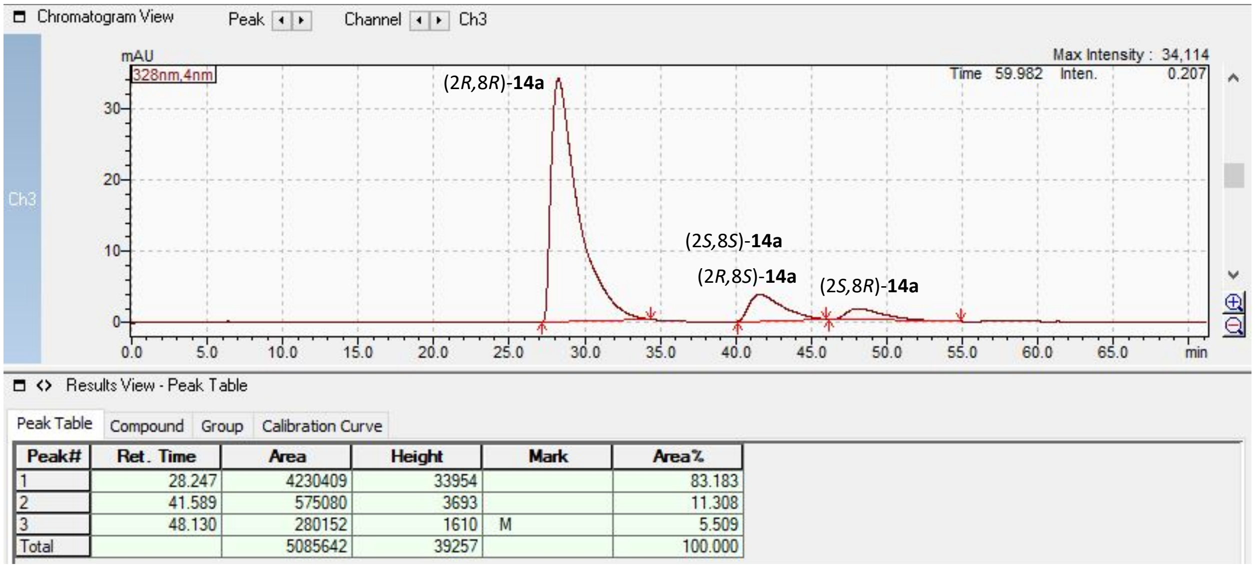

Peak#	Ret. Time	Area	Height	Mark	Area%
1	28.247	4230409	33954		83.183
2	41.589	575080	3693		11.308
3	48.130	280152	1610	M	5.509
Total		5085642	39257		100.000

Shimadzu Nexera Series SCL-40, UV/PDA detector, 328 nm, CHIRALPAK IB N-3, 150 X 4.6 mm column,, 0.1% EtOH/hexanes, Flow rate: 1 mL/min

Peak	Retention Time (min)	Peak area (%)
Peak 1	20.14	2.93
Peak 2	21.63	6.54
Peak 3	28.75	75.40
Peak 4	31.44	15.13

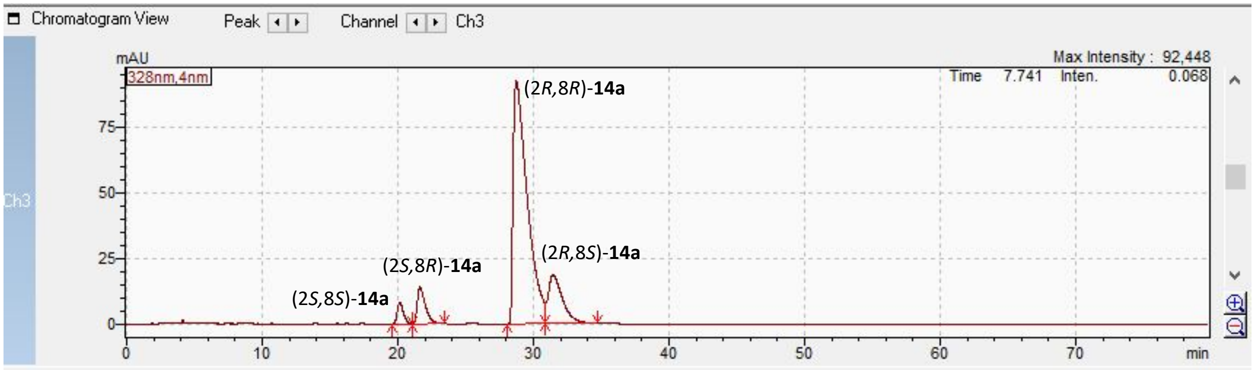

Results View - Peak Table

Peak Table | Compound | Group | Calibration Curve

Peak#	Ret. Time	Area	Height	Mark	Area%
1	20.140	262805	8181		2.933
2	21.633	585865	13986	V	6.538
3	28.747	6756503	92430		75.396
4	31.440	1356171	18736	V	15.134
Total		8961345	133333		100.000

Monitoring the asymmetric PKR for dr and er throughout the reaction

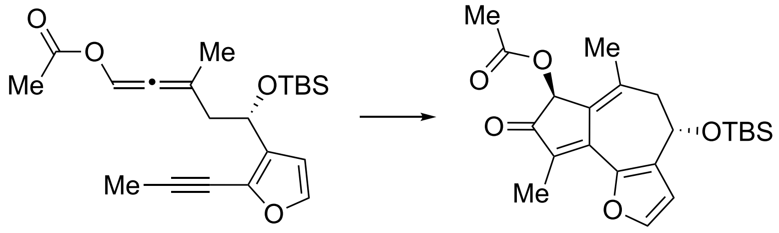

(8*S*)-**13a** (2*S*,8*S*)-**14a**

Table 2, entry 3[f]

(4*S*,7*S*)-4-((*tert*-butyldimethylsilyl)oxy)-6,9-dimethyl-8-oxo-4,5,7,8-tetrahydroazuleno[4,5-b]furan-7-yl acetate ((2*S*,8*S*)-14a).

Follows general procedure **I**, bis(1,5-cyclooctadiene)rhodium(I) tetrafluoroborate (2.2 mg, 0.005 mmol), (*R*)-MonoPhos-alkene (*R*)-**4** (3.3 mg, 0.008 mmol), carbon monoxide (100%), (5*S*)-5-((tert-butyldimethylsilyl)oxy)-3-methyl-5-(2-(prop-1-yn-1-yl)furan-3-yl)penta-1,2-dien-1-yl acetate (*S*)-**13a** (59.8% ee) (20 mg, 0.05 mmol), DCE (5.3 mL, 0.01 M). The reaction was stirred

for 21 h in an oil bath preheated to 70 °C. Polymer-bound triphenylphosphine (18 mg, 1.0 eq, 3 mmol/g). After 4 h at rt, the reaction mixture was filtered, and concentrated *in vacuo*. The crude residue was purified via silica gel flash chromatography (5% ethyl acetate/hexanes) to give 6.4 mg (51% yield) of the title compound as a yellow oil (dr (*trans*:*cis*) = 3.3:1 (77:23)) [er = 12.2:87.8 (2*R*,8*R*:2*S*,8*R*) and 7.8:92.2 (2*R*,8*S*:2*S*,8*S*) respectively]. TLC and ^{1}H NMR data match those of **14a**.

Note: Two aliquots (2.2 mL) were taken out of the reaction and considered for calculating isolated yield.

This reaction was monitored, and after 5 h, dr (*trans:cis*) = 4:1 (80:20), er = 9.9:90.1 (2*R*,8*R*:2*S*,8*R*) and 7.6:92.4 (2*R*,8*S*:2*S*,8*S*) respectively.

Aliquot after 5 h:

Shimadzu Nexera Series SCL-40, UV/PDA detector, 328 nm, CHIRALPAK IB N-3, 150 X 4.6 mm column,, 0.1% EtOH/hexanes, Flow rate: 1 mL/min

Peak	Retention Time (min)	Peak area (%)
Peak 1	39.36	76.54
Peak 2	43.08	15.40
Peak 3	66.24	1.73
Peak 4	69.82	6.33

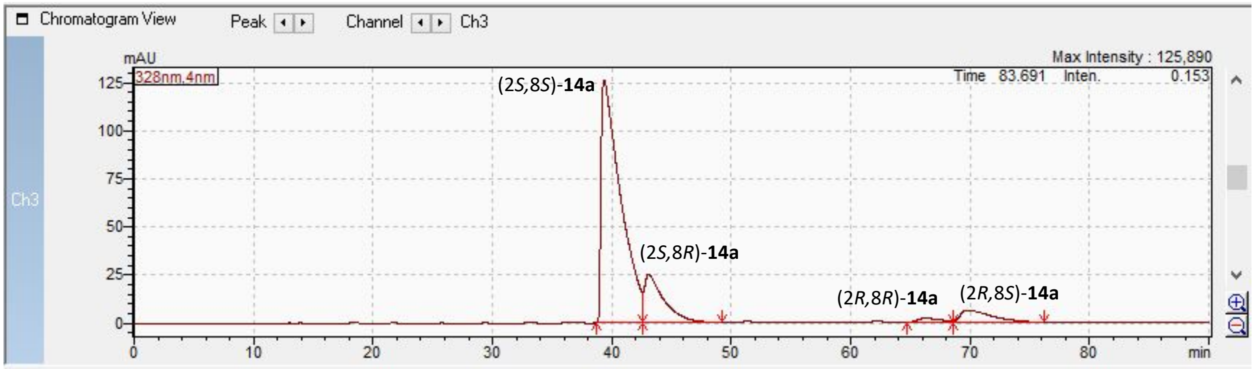

Results View - Peak Table

Peak Table | Compound | Group | Calibration Curve

Peak#	Ret. Time	Area	Height	Mark	Area%
1	39.356	14284498	125786		76.536
2	43.079	2873775	25186	V	15.398
3	66.239	323652	2280		1.734
4	69.818	1181954	6479	V	6.333
Total		18663879	159732		100.000

At the end of the reaction:

Shimadzu Nexera Series SCL-40, UV/PDA detector, 328 nm, CHIRALPAK IB N-3, 150 X 4.6 mm column,, 0.1% EtOH/hexanes, Flow rate: 1 mL/min

Peak	Retention Time (min)	Peak area (%)
Peak 1	37.75	75.52
Peak 2	41.23	15.90
Peak 3	63.69	2.23
Peak 4	67.48	6.35

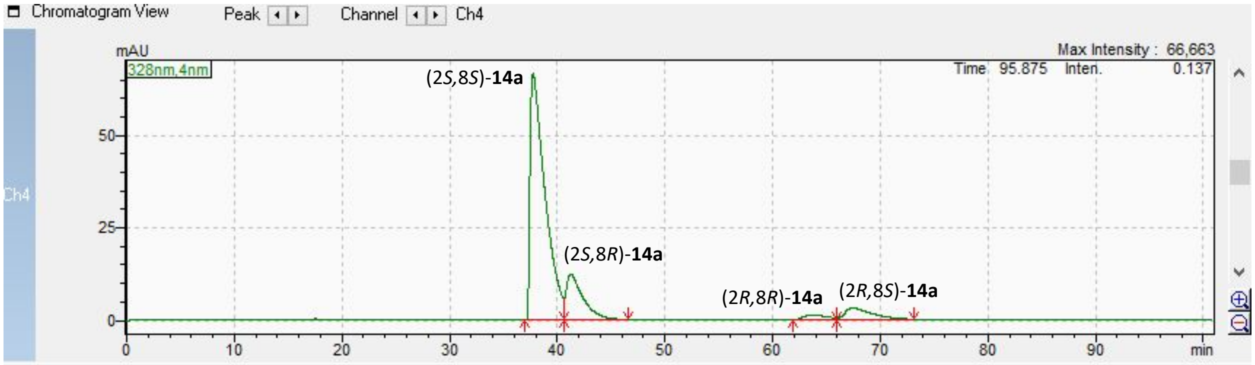

Results View - Peak Table

Peak Table | Compound | Group | Calibration Curve

Peak#	Ret. Time	Area	Height	Mark	Area%
1	37.749	6443978	66556		75.519
2	41.234	1356575	12394	V	15.898
3	63.693	190533	1294		2.233
4	67.479	541788	3115	V	6.349
Total		8532873	83359		100.000

Large scale asymmetric PKR

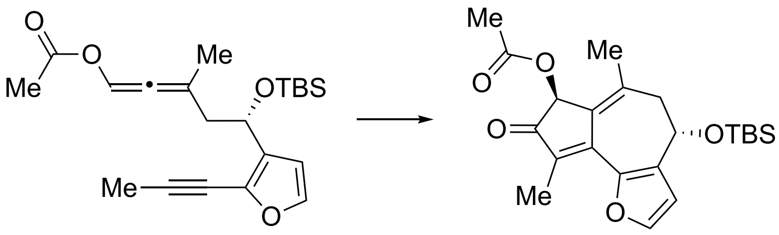

(8*S*)-**13a** (2*S*,8*S*)-**14a**

Table 2, entry 3[g]

(4*S*,7*S*)-4-((*tert*-butyldimethylsilyl)oxy)-6,9-dimethyl-8-oxo-4,5,7,8-tetrahydroazuleno[4,5-b]furan-7-yl acetate ((2*S*,8*S*)-14a).

Follows general procedure **I**, bis(1,5-cyclooctadiene)rhodium(I) tetrafluoroborate (15.2 mg, 0.04 mmol), (*R*)-MonoPhos-alkene (*R*)-**4** (22.8 mg, 0.06 mmol), carbon monoxide (100%), (5*S*)-5-((tert-butyldimethylsilyl)oxy)-3-methyl-5-(2-(prop-1-yn-1-yl)furan-3-yl)penta-1,2-dien-1-yl acetate (*S*)-**13a** (59.8% ee) (140 mg, 0.4 mmol), DCE (37.0 mL, 0.01 M). The reaction was stirred

for 23 h in an oil bath preheated to 70 °C. The crude residue was purified via silica gel flash chromatography (4-6% ethyl acetate/hexanes) to give 53 mg (36% yield) of the title compound as a yellow oil (dr (*trans*:*cis*) = 3.5:1 (78:22)) [er = 15.1:84.9 (2*R*,8*R*:2*S*,8*R*) and 8.8:91.2 (2*R*,8*S*:2*S*,8*S*) respectively]. TLC and ^{1}H NMR data match those of **14a**.

Shimadzu Nexera Series SCL-40, UV/PDA detector, 328 nm, CHIRALPAK IB N-3, 150 X 4.6 mm column,, 0.1% EtOH/hexanes, Flow rate: 1 mL/min

Peak	Retention Time (min)	Peak area (%)
Peak 1	32.06	76.10
Peak 2	35.09	14.09
Peak 3	52.21	2.54
Peak 4	55.40	7.28

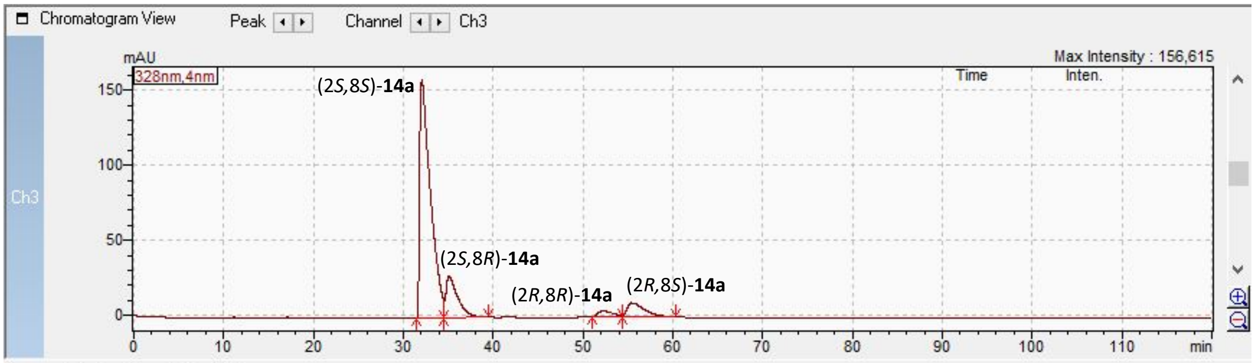

Results View - Peak Table

Peak Table | Compound | Group | Calibration Curve

Peak#	Ret. Time	Area	Height	Mark	Area%
1	32.056	13250913	158017		76.095
2	35.088	2453888	27665	V	14.092
3	52.214	441818	3859		2.537
4	55.404	1266943	9322	V	7.276
Total		17413561	198863		100.000

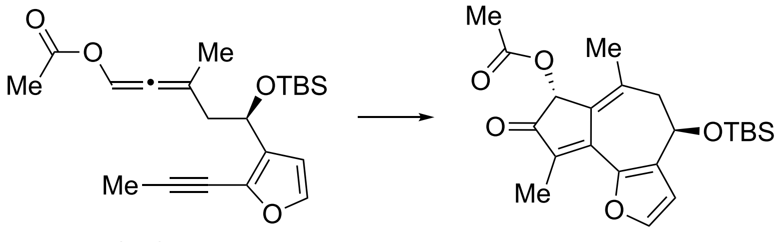

Table 2, entry 4

(4*R*,7*R*)-4-((*tert*-butyldimethylsilyl)oxy)-6,9-dimethyl-8-oxo-4,5,7,8-tetrahydroazuleno[4,5-b]furan-7-yl acetate ((2*R*,8*R*)-14a).

Follows general procedure **I**, bis(1,5-cyclooctadiene)rhodium(I) tetrafluoroborate (1.6 mg, 0.004 mmol), (*S*)-MonoPhos-alkene (*S*)-**4** (2.5 mg, 0.006 mmol), carbon monoxide (100%), (5*R*)-5-((tert-butyldimethylsilyl)oxy)-3-methyl-5-(2-(prop-1-yn-1-yl)furan-3-yl)penta-1,2-dien-1-yl acetate (*R*)-**13a** (≥99% ee) (16 mg, 0.04 mmol), DCE (4.0 mL, 0.01 M). The reaction was stirred for 22 h in an oil bath preheated to 70 °C. Polymer-bound triphenylphosphine (16 mg, 1.0 eq, 3 mmol/g). After 5 h at rt, the reaction mixture was filtered, and concentrated *in vacuo*. The crude residue was purified via silica gel flash chromatography (5-10% ethyl acetate/hexanes) to give 7 mg (41% yield) of the title compound as a yellow oil (dr (*trans*:*cis*) = 8.6:1 (90:10)) [er = 94.8:5.2 (2*R,*8*R*:2*S,*8*R*)]. TLC and ^{1}H NMR data match those of **14a**.

Shimadzu Nexera Series SCL-40, UV/PDA detector, 328 nm, CHIRALPAK IB N-3, 150 X 4.6 mm column,, 0.1% EtOH/hexanes, Flow rate: 1 mL/min

Peak	Retention Time (min)	Peak area (%)
Peak 1	5.76	5.20
Peak 2	6.25	94.80

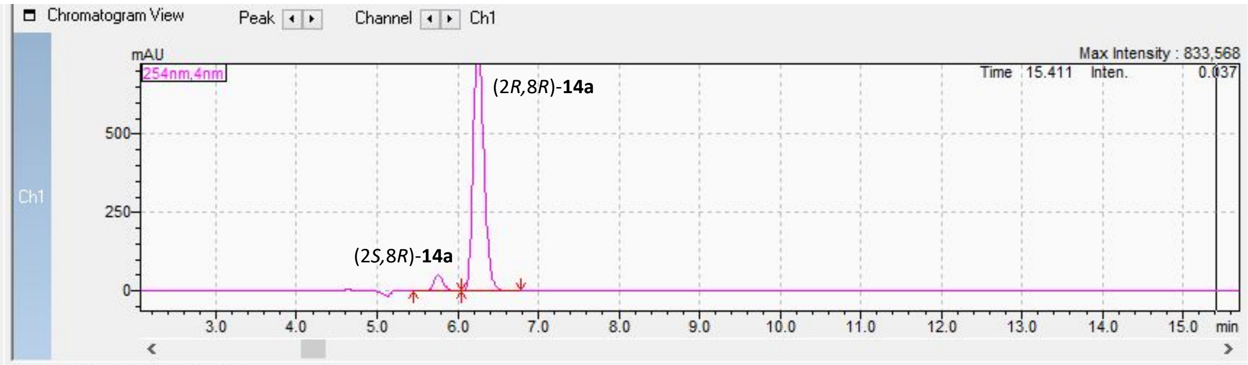

Results View - Peak Table

Peak Table | Compound | Group | Calibration Curve

Peak#	Ret. Time	Area	Height	Mark	Area%
1	5.759	405532	50499	M	5.200
2	6.251	7393715	833377	M	94.800
Total		7799247	883876		100.000

Shimadzu Nexera Series SCL-40, UV/PDA detector, 328 nm, CHIRALPAK IA-3, 250 X 4.6 mm column, 0.1% *i*PrOH/hexanes, Flow rate: 1 mL/min, 40 °C

Peak	Retention Time (min)	Peak area (%)
Peak 1	12.45	92.15
Peak 2	20.28	7.85

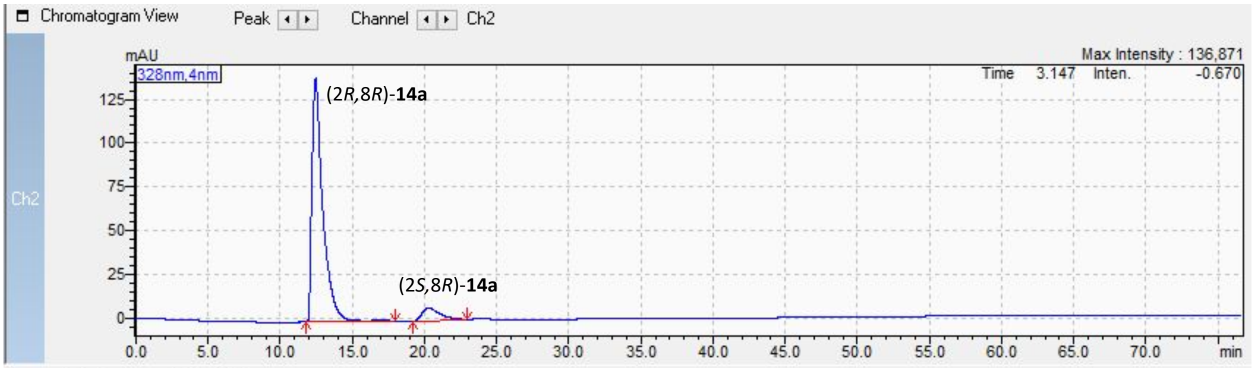

Results View - Peak Table

Peak Table | Compound | Group | Calibration Curve

Peak#	Ret. Time	Area	Height	Mark	Area%
1	12.448	7263289	138745	S	92.154
2	20.281	618378	7226		7.846
Total		7881667	145972		100.000

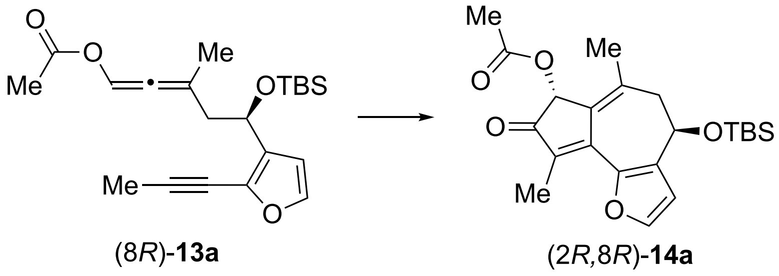

Table 2, entry 5

(4*R*,7*R*)-4-((*tert*-butyldimethylsilyl)oxy)-6,9-dimethyl-8-oxo-4,5,7,8-tetrahydroazuleno[4,5-b]furan-7-yl acetate ((2*R*,8*R*)-14a).

Follows general procedure **I**, bis(1,5-cyclooctadiene)rhodium(I) tetrafluoroborate (1.6 mg, 0.004 mmol), (*R*)-MonoPhos-alkene (*R*)-**4** (2.5 mg, 0.006 mmol), carbon monoxide (100%), (5*R*)-5-((tert-butyldimethylsilyl)oxy)-3-methyl-5-(2-(prop-1-yn-1-yl)furan-3-yl)penta-1,2-dien-1-yl acetate (*R*)-**13a** (≥99% ee) (16 mg, 0.04 mmol), DCE (4.0 mL, 0.01 M). The reaction was stirred for 22 h in an oil bath preheated to 70 °C. Polymer-bound triphenylphosphine (16 mg, 1.0 eq, 3 mmol/g). After 5 h at rt, the reaction mixture was filtered, and concentrated *in vacuo*. The crude residue was purified via silica gel flash chromatography (5-10% ethyl acetate/hexanes) to give 5 mg (29% yield) of the title compound as a yellow oil (dr (*trans*:*cis*) = 1:2.9 (26:74)) [er = 22.1:77.9 (2*R,*8*R*:2*S,*8*R*) and 56.8:43.2 (2*R,*8*S*:2*S,*8*S*) respectively]. TLC and ^{1}H NMR data match those of **14a**.

Shimadzu Nexera Series SCL-40, UV/PDA detector, 328 nm, CHIRALPAK IB N-3, 150 X 4.6 mm column,, 0.1% EtOH/hexanes, Flow rate: 1 mL/min

Peak	Retention Time (min)	Peak area (%)
Peak 1	9.18	1.89

Peak 2	9.70	74.47
Peak 3	11.41	21.11
Peak 4	14.25	2.52

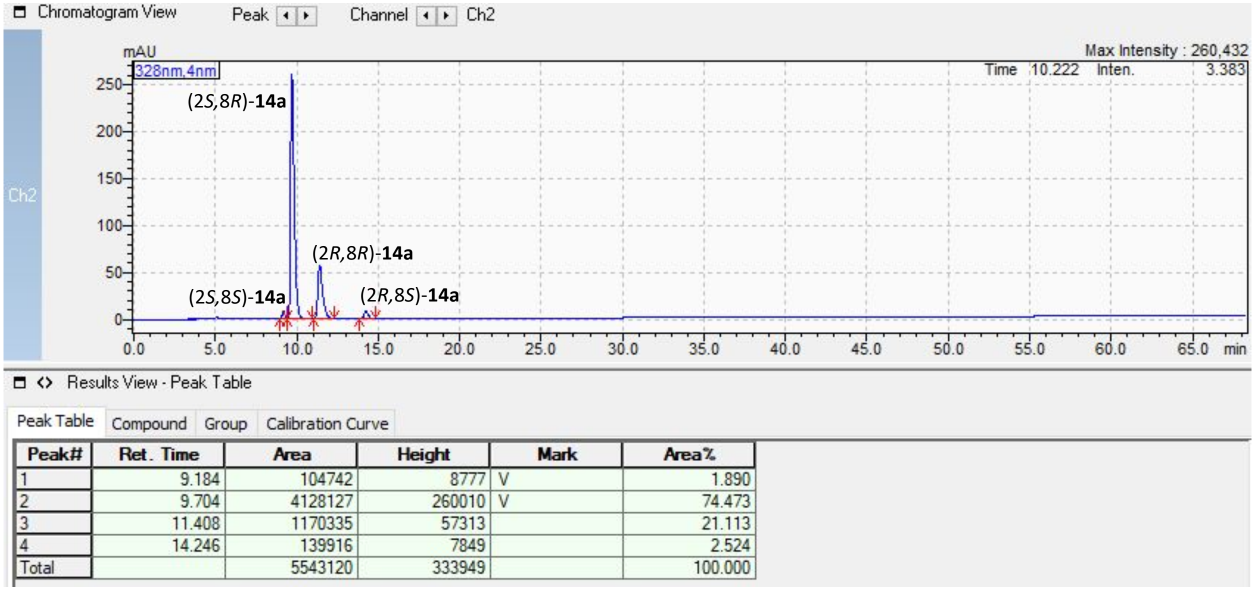

Peak#	Ret. Time	Area	Height	Mark	Area%
1	9.184	104742	8777	V	1.890
2	9.704	4128127	260010	V	74.473
3	11.408	1170335	57313		21.113
4	14.246	139916	7849		2.524
Total		5543120	333949		100.000

Shimadzu Nexera Series SCL-40, UV/PDA detector, 328 nm, CHIRALPAK IA-3, 250 X 4.6 mm column, 0.1% *i*PrOH/hexanes, Flow rate: 1 mL/min, 40 °C

Peak	Retention Time (min)	Peak area (%)
Peak 1	18.57	21.00
Peak 2	31.32	79.00

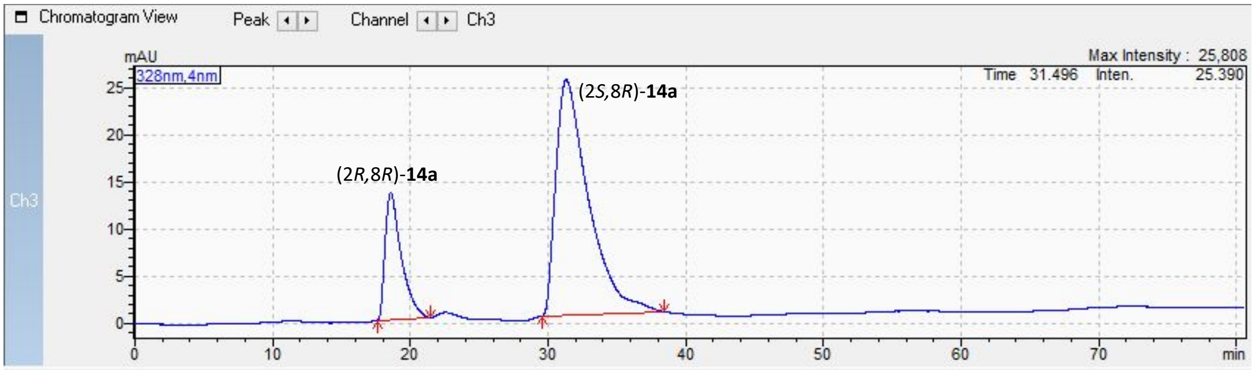

Results View - Peak Table

Peak Table | Compound | Group | Calibration Curve

Peak#	Ret. Time	Area	Height	Mark	Area%
1	18.574	1097268	13464		20.995
2	31.325	4129063	24934		79.005
Total		5226331	38398		100.000

Note: The inconsistency that is observed for the retention times of different samples of **14a** could be the result of a few factors. Temperature can play an important role in the retention of chiral separations, where higher temperature results in faster elution and sharper peaks, and lower temperature results in longer elution and broader peaks. In this case, the column was temperature-controlled via the column oven, so this is likely not the root cause. Another possibility is the effectiveness of HPLC pumps to accurately and consistently mix small percentages of mobile phase components. This is typically more of a problem with low-pressure pumps where air bubbles and issues with gradient proportioning values are more common and can yield slight variations in the actual mobile phase composition delivered to the column. This is the likely cause for the variability in this case as solvents were not premixed, but rather mixed in the system. It is expected that premixing in the future would resolve the issues with drift.[11]

Two different chiral columns (CHIRALPAK IA-3 or IB N-3) and two different mobile phases (0.1% IPA/Hex or 0.1% EtOH/Hex) were used for different samples of **14a**. The chiral column and mobile phase were chosen based on the major peak to get a good separation.

Retention times (min) for different samples of **14a** with CHIRALPAK IA-3 and 0.1% *i*PrOH/hexanes

Peaks	Stereoisomer	FH-02-81-B	FH-02-102-B	FH-02-110-B	FH-02-133-B	FH-03-170-A	FH-04-69-A	FH-04-72-A
Peak 1	(2*R*,8*R*)	22.56	12.49	25.88	12.61	28.25	12.45	18.57
Peak 2	(2*S*,8*S*)	25.92	15.18	29.36	15.26	28.25	-	-
Peak 3	(2*R*,8*S*)	31.89	16.06	38.11	16.12	41.59	-	-
Peak 4	(2*S*,8*R*)	36.78	18.85	44.95	18.82	48.13	20.28	31.32

Retention times (min) for different samples of **14a** with CHIRALPAK IB N-3 and 0.1% EtOH/hexanes

Peaks	Stereoisomer	FH-03-58-A	FH-03-170-A	FH-03-133-C	FH-03-157-A-5 h	FH-03-157-A	FH-04-69-A	FH-04-72-A
Peak 1	(2*S*,8*S*)	33.78	20.14	32.06	39.36	37.75	-	9.18
Peak 2	(2*S*,8*R*)	36.16	21.63	35.09	43.08	41.23	5.76	9.70
Peak 3	(2*R*,8*R*)	52.81	28.75	52.21	66.24	63.69	6.25	11.41
Peak 4	(2*R*,8*S*)	58.49	31.44	55.40	69.82	67.48	-	14.25

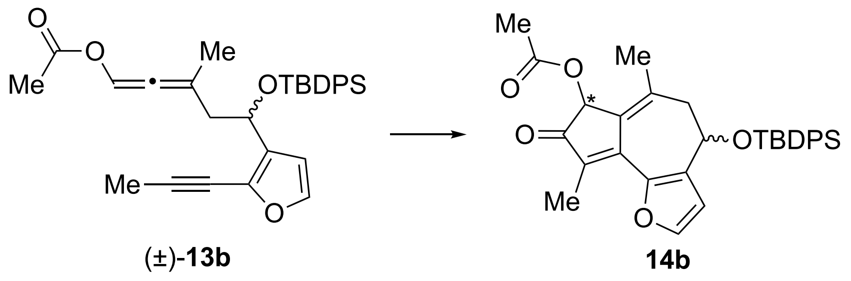

Table 1, entry 3

4-((*tert*-butyldiphenylsilyl)oxy)-6,9-dimethyl-8-oxo-4,5,7,8-tetrahydroazuleno[4,5-b]furan-7-yl acetate (14b).

Follows general procedure **F**, bis(1,5-cyclooctadiene)rhodium(I) tetrafluoroborate (2.0 mg, 0.005 mmol), triphenyl phosphine (2.0 mg, 0.0075 mmol), carbon monoxide (100%), (±)-5-((tert-butyldiphenylsilyl)oxy)-3-methyl-5-(2-(prop-1-yn-1-yl)furan-3-yl)penta-1,2-dien-1-yl acetate (±)-**13b** (28 mg, 0.05 mmol), DCE (2.5 mL, 0.02 M). The reaction was stirred for 51 h in an oil bath preheated to 70 °C. The crude residue was purified via silica gel flash chromatography (4-6% ethyl acetate/hexanes) to give 18 mg (60% yield) of the title compound as a yellow oil (dr (*trans*:*cis*) = 1:2.4 (29:71)).

Note: The ratio of PKR product **14b** to aldehyde byproduct **S8** was 1:0.06 (94:6) based on crude NMR.

^{1}H NMR (400 MHz, $CDCl_3$)

7.73–7.70 (m, 4 H), 7.61–7.57 (m, 4 H), 7.52 (d, *J* = 1.2 Hz, 2 H), 7.47–7.39 (m, 8 H), 7.36–7.33 (m, 4 H), 6.53 (d, *J* = 1.6 Hz, 1 H)**, 6.52 (d, *J* = 2.0 Hz, 1 H)*, 5.76 (s, 2 H), 5.02 (dd, *J* = 9.6, 2.4 Hz, 1 H)*, 4.94 (dd, *J* = 9.4, 1.6 Hz, 1 H)**, 2.75–2.64 (m, 2 H), 2.32–2.22 (m, 2 H), 2.18 (s, 3 H)**, 2.17 (s, 3 H)*, 2.10 (s, 3 H)*, 2.10 (s, 3 H)**, 1.59 (s, 3 H)*, 1.53 (s, 3 H)**, 1.11 (s, 9 H)*, 1.09 (s, 9 H)** ppm

distinguishable diastereomeric peaks for minor isomer (*trans*) *

distinguishable diastereomeric peaks for major isomer (*cis*) **

* dr = 1:2.4 (29:71)

^{13}C NMR (100 MHz, $CDCl_3$)

199.6, 169.9**, 169.8*, 149.1*, 149.0**, 145.3**, 144.9*, 144.8*, 144.7**, 136.2, 136.0*, 136.0**, 135.9**, 135.8*, 134.4*, 134.0*, 133.9**, 133.4**, 133.3*, 133.3**, 132.9**, 132.7*, 130.2*, 130.2**, 130.0**, 130.0*, 128.0, 127.9**,

127.9*, 112.3**, 112.2*, 71.6**, 71.3*, 66.3*, 66.2**, 43.8*, 43.6**, 27.1*, 27.1**, 24.8*, 24.6**, 20.8*, 20.7**, 19.5**, 19.5*, 10.2**, 10.1* ppm

distinguishable diastereomeric peaks for minor isomer (*trans*) *

distinguishable diastereomeric peaks for major isomer (*cis*) **

IR (Thin Film)

3071, 3051, 2931, 2857, 1745, 1697, 1267, 1221 cm^{-1}

HRMS HRMS-ESI (m/z): $[M + H]^+$ calcd for $C_{32}H_{34}O_5Si$, 527.2248; found, 527.2246

TLC R_f = 0.47 (35% ethyl acetate/hexanes); silica gel, UV, *p*-anisaldehyde

Determination of enantiomeric ratio: Follows general procedure **A**, gradual addition of aliquots of the preprepared $Eu(hfc)_3/CDCl_3$ solution to **14b** in $CDCl_3$ showed the resonances for the two diastereomers at 1.59 and 1.53 ppm (methyl at C10) splitting into four resonances at 1.68, 1.65, 1.63, and 1.60 ppm.

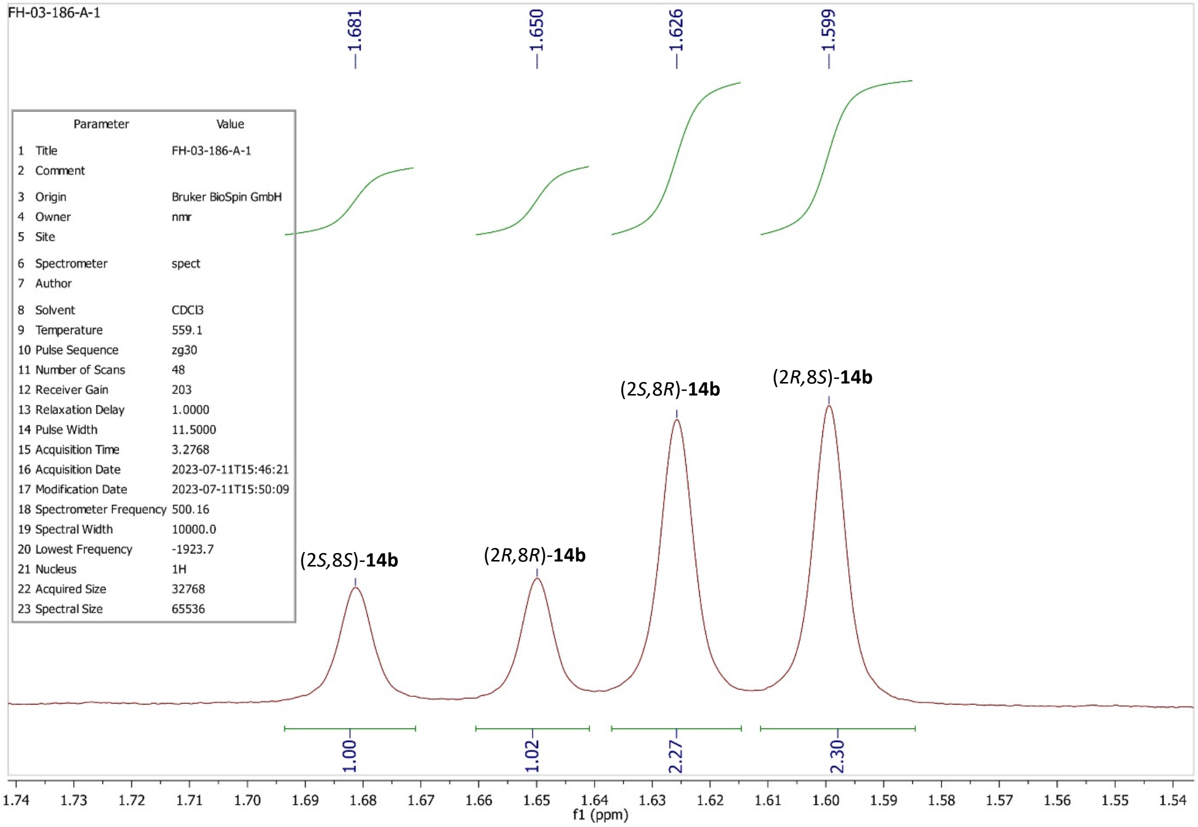

Determination of relative configuration of diastereomers of **14b**: relative configuration of diastereomers of **14b** was determined based on analogy to diastereomers of **14a**. In **14b** peaks at 5.02 (dd, *J* = 9.6, 2.4 Hz, 1 H) and 4.94 (dd, *J* = 9.6, 1.6 Hz, 1 H) ppm (proton at C8) correspond to minor and major diastereomers respectively. In **14a** peaks at 4.93 (dd, *J* = 10.8, 2.8 Hz, 1 H) and 4.89 (dd, *J* = 10.8, 2.0 Hz, 1 H) ppm (proton at C8) correspondsto minor and major diastereomers respectively.

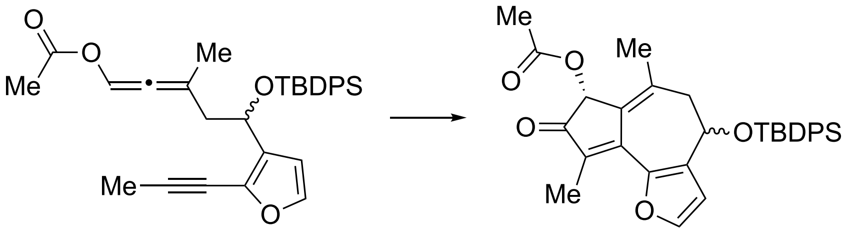

(±)-**13b** **14b**

Table 2, entry 6

(7*R*)-4-((*tert*-butyldiphenylsilyl)oxy)-6,9-dimethyl-8-oxo-4,5,7,8-tetrahydroazuleno[4,5-b]furan-7-yl acetate (14b).

Follows general procedure **I**, bis(1,5-cyclooctadiene)rhodium(I) tetrafluoroborate (2.0 mg, 0.005 mmol), (*S*)-MonoPhos-alkene (*S*)-**4** (3.0 mg, 0.007 mmol), carbon monoxide (100%), (±)-5-((tert-butyldiphenylsilyl)oxy)-3-methyl-5-(2-(prop-1-yn-1-yl)furan-3-yl)penta-1,2-dien-1-yl acetate (±)-**13b** (24 mg, 0.05 mmol), DCE (4.8 mL, 0.01 M). The reaction was stirred for 47 h in an oil bath preheated to 70 °C. Polymer-bound triphenylphosphine (16 mg, 1.0 eq, 3 mmol/g). After 4 h at rt, the reaction mixture was filtered, and concentrated *in vacuo*. The crude residue was purified via silica gel flash chromatography (4-8% ethyl acetate/hexanes) to give 11 mg (44% yield) of the title compound as a yellow oil (dr (*trans*:*cis*) = 1.6:1 (62:38)) [er = 92:8 (2*R*,8*R*:2*S*,8*R*) and 76:24 (2*R*,8*S*:2*S*,8*S*) respectively]. TLC and ^{1}H NMR data match those of **14b**.

Determination of enantiomeric ratio: Follows general procedure **A**, gradual addition of aliquots of the preprepared Eu(hfc)$_3$/CDCl$_3$ solution to **14b** in CDCl$_3$ showed the resonances for the two diastereomers at 1.59 and 1.53 ppm (methyl at C10) splitting into four resonances at 1.64, 1.62, 1.58, and 1.57 ppm.

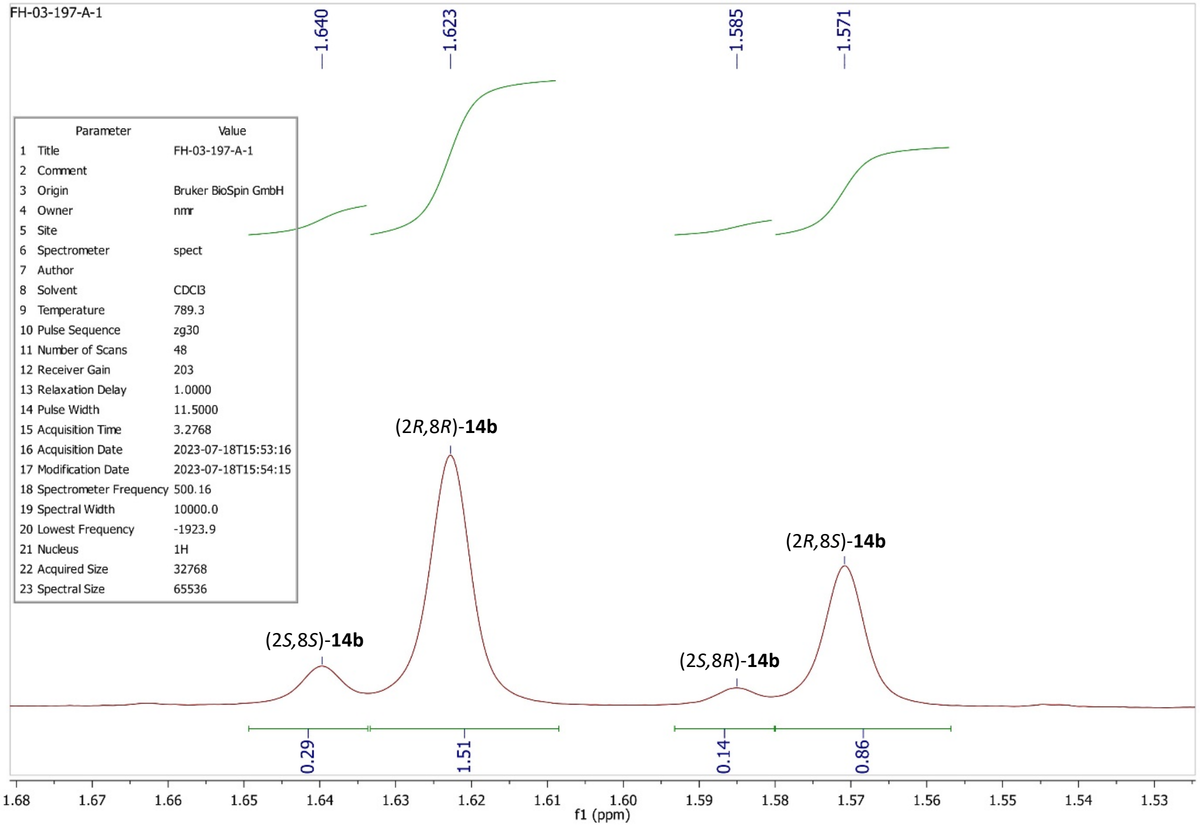

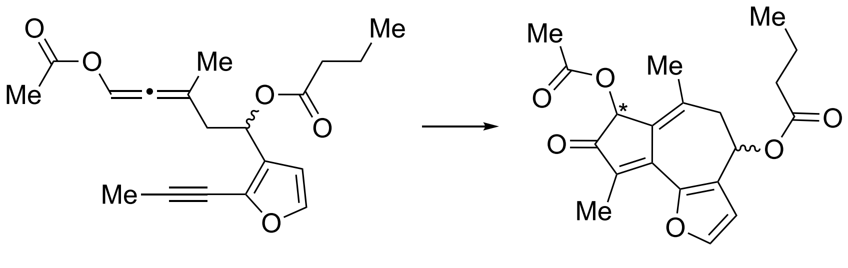

Table 1, entry 4

7-acetoxy-6,9-dimethyl-8-oxo-4,5,7,8-tetrahydroazuleno[4,5-b]furan-4-yl butyrate (14c).[7]

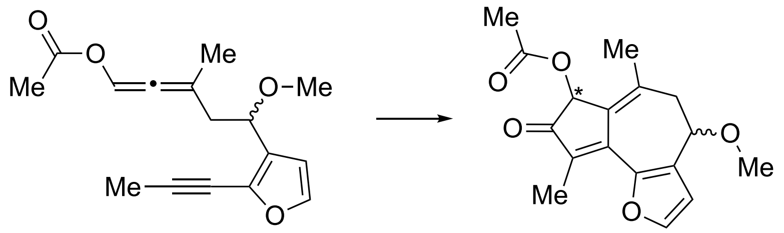

(±)-**13d** **14d**

Table 1, entry 5

4-methoxy-6,9-dimethyl-8-oxo-4,5,7,8-tetrahydroazuleno[4,5-b]furan-7-yl acetate (14d).

Follows general procedure **F**, bis(1,5-cyclooctadiene)rhodium(I) tetrafluoroborate (1.4 mg, 0.003 mmol), triphenyl phosphine (1.4 mg, 0.005 mmol), carbon monoxide (100%), (±)-5-methoxy-3-methyl-5-(2-(prop-1-yn-1-yl)furan-3-yl)penta-1,2-dien-1-yl acetate (±)-**13d** (9 mg, 0.03 mmol), hexamethylbenzene (0.1 equiv, 0.6 mg, 0.003 mmol), DCE (1.7 mL, 0.02 M). The reaction was stirred for 45 h in an oil bath preheated to 70 °C. The crude residue was purified via silica gel flash chromatography (15-25% ethyl acetate/hexanes) to give 1 mg (15% yield) of the title compound as a yellow oil (dr (*trans*:*cis*) = 1:1.4 (42:58)).

Note: The ratio of PKR product **14d** to aldehyde byproduct **S9** was 1:0.5 (67:33) based on crude NMR.

Note: One aliquot (0.5 mL) was taken out of the reaction and considered for calculating isolated yield.

NMR yield: 22%

^{1}H NMR (400 MHz, $CDCl_3$)

7.59 (d, J = 1.2 Hz, 2 H), 6.61 (d, J = 1.6 Hz, 1 H)**, 6.59 (d, J = 1.2 Hz, 1 H)*, 5.87 (s, 1 H)**, 5.80 (s, 1 H)*, 4.44 (dd, J = 7.4, 3.2 Hz, 1 H)*, 4.40 (dd, J = 8.0,

2.4 Hz, 1 H)**, 3.44 (s, 3 H)*, 3.40 (s, 3 H)**, 2.87–2.68 (m, 4 H), 2.21 (s, 6 H), 2.16 (s, 3 H)**, 2.14 (s, 3 H)*, 1.97 (s, 3 H)*, 1.95 (s, 3 H)** ppm

distinguishable diastereomeric peaks for minor isomer (*trans*) *

distinguishable diastereomeric peaks for major isomer (*cis*) **

* dr = 1:1.4 (42:58)

^{13}C NMR (125 MHz, $CDCl_3$)

199.7, 170.1**, 169.8*, 148.8**, 148.7*, 146.2**, 146.1*, 144.9*, 144.9**, 134.0* 133.4**, 133.3*, 133.2**, 132.8**, 132.5*, 128.0**, 127.9*, 112.9**, 112.6*, 73.5*, 73.2**, 71.9*, 71.6**, 57.4*, 56.9**, 40.1**, 40.1*, 25.4*, 25.0**, 20.9**, 20.8*, 10.3**, 10.3* ppm

distinguishable diastereomeric peaks for minor isomer (*trans*) *

distinguishable diastereomeric peaks for major isomer (*cis*) **

IR (Thin Film)

2927, 2858, 1744, 1695, 1221 cm^{-1}

HRMS HRMS-ESI (m/z): $[M + H]^+$ calcd for $C_{17}H_{19}O_5$, 303.1227; found, 303.1236

TLC R_f = 0.20 (35% ethyl acetate/hexanes); silica gel, UV, *p*-anisaldehyde

Shimadzu Nexera Series SCL-40, UV/PDA detector, 328 nm, CHIRALPAK IH-3, 250 X 4.6 mm column, 2% EtOH/hexanes, Flow rate: 1 mL/min

Peak	Retention Time (min)	Peak area (%)
Peak 1	18.85	13.61
Peak 2	19.28	28.14
Peak 3	28.40	29.04

Peak 4	30.78	29.22

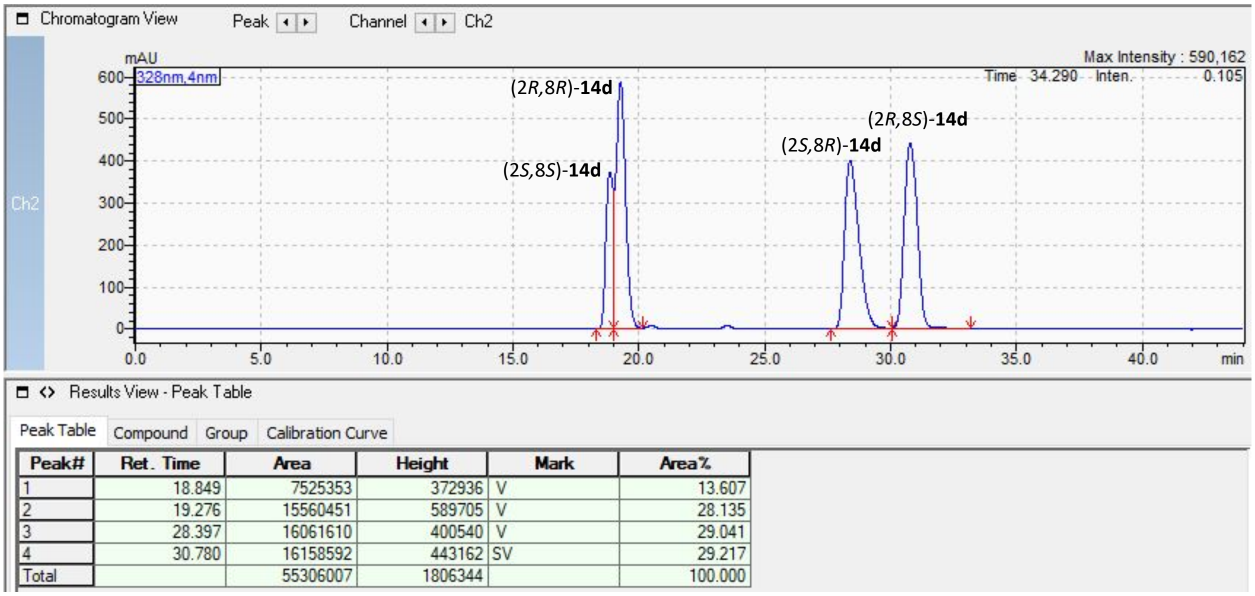

Peak#	Ret. Time	Area	Height	Mark	Area%
1	18.849	7525353	372936	V	13.607
2	19.276	15560451	589705	V	28.135
3	28.397	16061610	400540	V	29.041
4	30.780	16158592	443162	SV	29.217
Total		55306007	1806344		100.000

Determination of relative configuration of diastereomers of **14d**: relative configuration of diastereomers of **14d** was determined based on analogy to diastereomers of **14a**. In **14d** peaks at 4.44 (dd, J = 7.5, 3.0 Hz, 1 H) and 4.40 (dd, J = 8.5, 2.5 Hz, 1 H) ppm (proton at C8) corresponds to minor and major diastereomers respectively. In **14a** peaks at 4.93 (dd, J = 10.8, 2.8 Hz, 1 H) and 4.89 (dd, J = 10.8, 2.0 Hz, 1 H) ppm (proton at C8) correspond to minor and major diastereomers respectively.

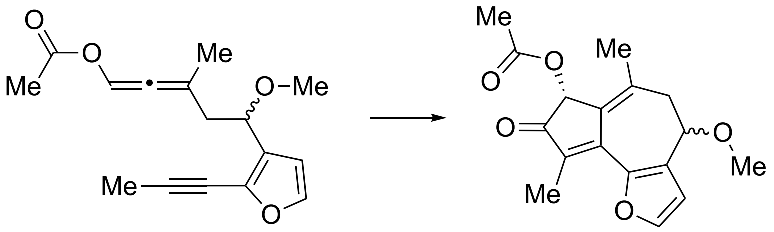

(±)-**13d** **14d**

Table 2, entry 7

(7*R*)-4-methoxy-6,9-dimethyl-8-oxo-4,5,7,8-tetrahydroazuleno[4,5-b]furan-7-yl acetate (14d).

Follows general procedure **I,** bis(1,5-cyclooctadiene)rhodium(I) tetrafluoroborate (3.4 mg, 0.008 mmol), (*S*)-MonoPhos-alkene (*S*)-**4** (5.2 mg, 0.01 mmol), carbon monoxide (100%), (±)-5-methoxy-3-methyl-5-(2-(prop-1-yn-1-yl)furan-3-yl)penta-1,2-dien-1-yl acetate (±)-**13d** (23 mg, 0.08 mmol), hexamethylbenzene (0.1 equiv, 1.4 mg, 0.008 mmol), DCE (8.4 mL, 0.01 M). The reaction was stirred for 24 h in an oil bath preheated to 70 °C. The crude residue was purified via silica gel flash chromatography (15-25% ethyl acetate/hexanes) to give 6.5 mg (28% yield) of the title compound as a yellow oil (dr (*trans*:*cis*) = 1.1:1 (52:48)) [er = 86.2:13.8 (2*R*,8*R*:2*S*,8*R*) and 82.5:17.5 (2*R*,8*S*:2*S*,8*S*) respectively]. TLC and ^{1}H NMR data match those of **14d**.

Note: One aliquot (0.7 mL) was taken out of the reaction and considered for calculating isolated yield.

NMR yield: 22%

Shimadzu Nexera Series SCL-40, UV/PDA detector, 328 nm, CHIRALPAK IH-3, 250 X 4.6 mm column, 2% EtOH/hexanes, Flow rate: 1 mL/min

Peak	Retention Time (min)	Peak area (%)
Peak 1	19.28	42.38
Peak 2	28.51	8.54
Peak 3	30.51	49.08

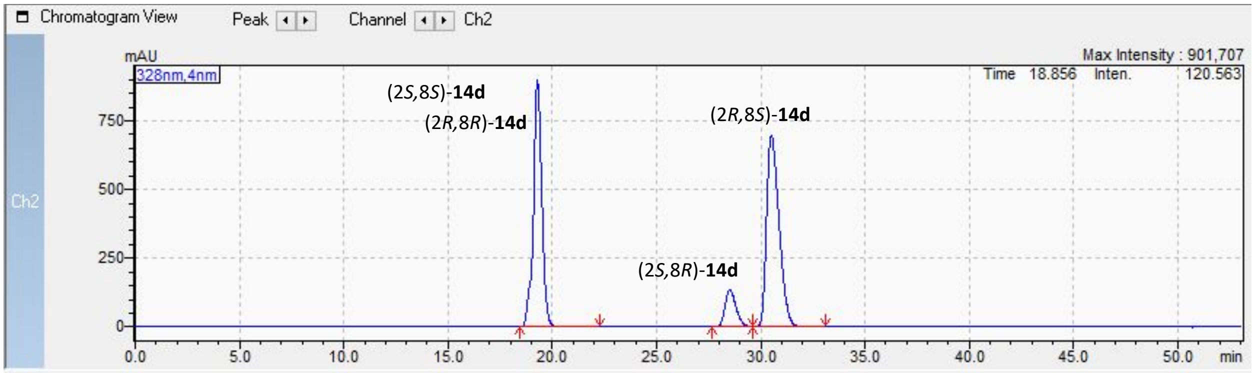

Results View - Peak Table

Peak Table | Compound | Group | Calibration Curve

Peak#	Ret. Time	Area	Height	Mark	Area%
1	19.285	25230722	902157	SV	42.382
2	28.509	5084057	135088		8.540
3	30.511	29217115	697920	V	49.078
Total		59531894	1735165		100.000

Deconvolution for the first peak:

Calculated peak area for peaks 1 and 2 after deconvolution

Peak	Retention Time (min)	Peak area (%)
Peak 1	19.09	6.63
Peak 2	19.29	35.74

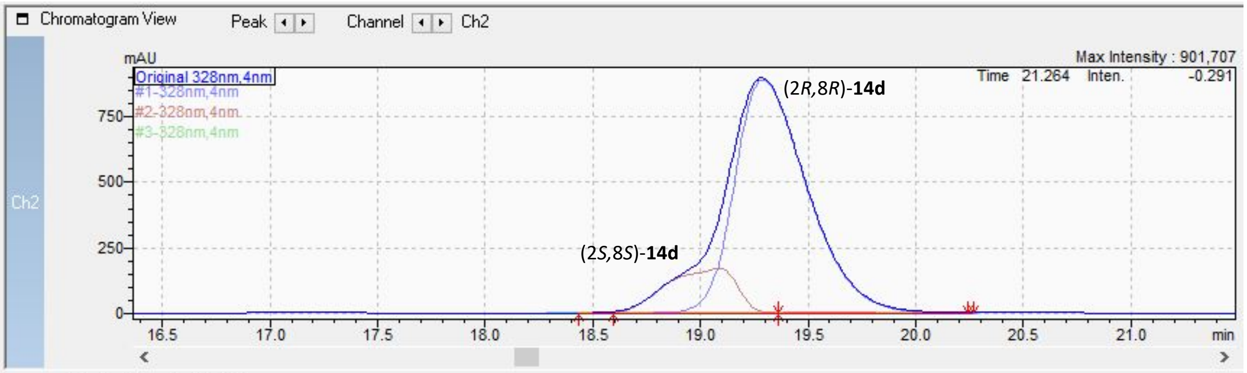

Results View - Peak Table

Peak Table | Compound | Group | Calibration Curve

Peak#	Ret. Time	Area	Height	Mark	Area%
1	19.086	3909142	172725	D	15.640
2	19.290	21034324	893750	D	84.154
3	19.523	51517	1987	V D	0.206
Total		24994984	1068462		100.000

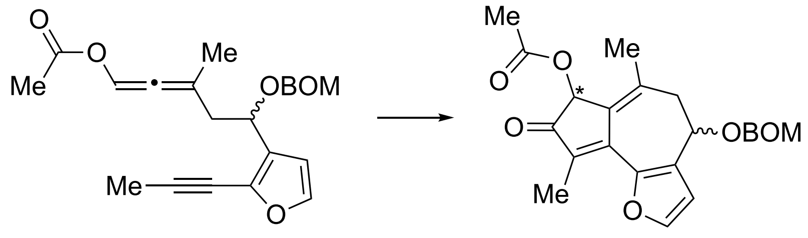

(±)-**13e** **14e**

Table 1, entry 6

4-((benzyloxy)methoxy)-6,9-dimethyl-8-oxo-4,5,7,8-tetrahydroazuleno[4,5-*b*]furan-7-yl acetate (14e).

Follows general procedure **F**, bis(1,5-cyclooctadiene)rhodium(I) tetrafluoroborate (1.2 mg, 0.003 mmol), triphenylphosphine (1.1 mg, 0.004 mmol), carbon monoxide (100%), (±)-5((benzyloxy)methoxy)-3-methyl-5-(2-(prop-1-yn-1-yl)furan-3-yl)penta-1,2-dien-1-yl acetate (±)-**13e** (11 mg, 0.03 mmol), DCE (1.4 mL, 0.02 M). The reaction was stirred for 140 h in an oil bath preheated to 70 °C. The crude residue was purified via silica gel flash chromatography (20% ethyl acetate/hexanes) to give 4 mg (33% yield) of the title compound as a yellow oil (dr (*trans*:*cis*) = 1:1.5 (40:60)). TLC and ^{1}H NMR data match those of **14e**.

Note: The ratio of PKR product **14e** to aldehyde byproduct **S11** was 1:0.2 (81:19) based on crude NMR.

Determination of enantiomeric ratio: Follows general procedure **A**, gradual addition of aliquots of the preprepared Eu(hfc)$_3$/CDCl$_3$ solution to **14e** in CDCl$_3$ showed the resonances for the two diastereomers at 5.86 and 5.81 ppm (proton at C2) splitting into four resonances at 6.10, 6.05, 6.02, and 5.96 ppm.

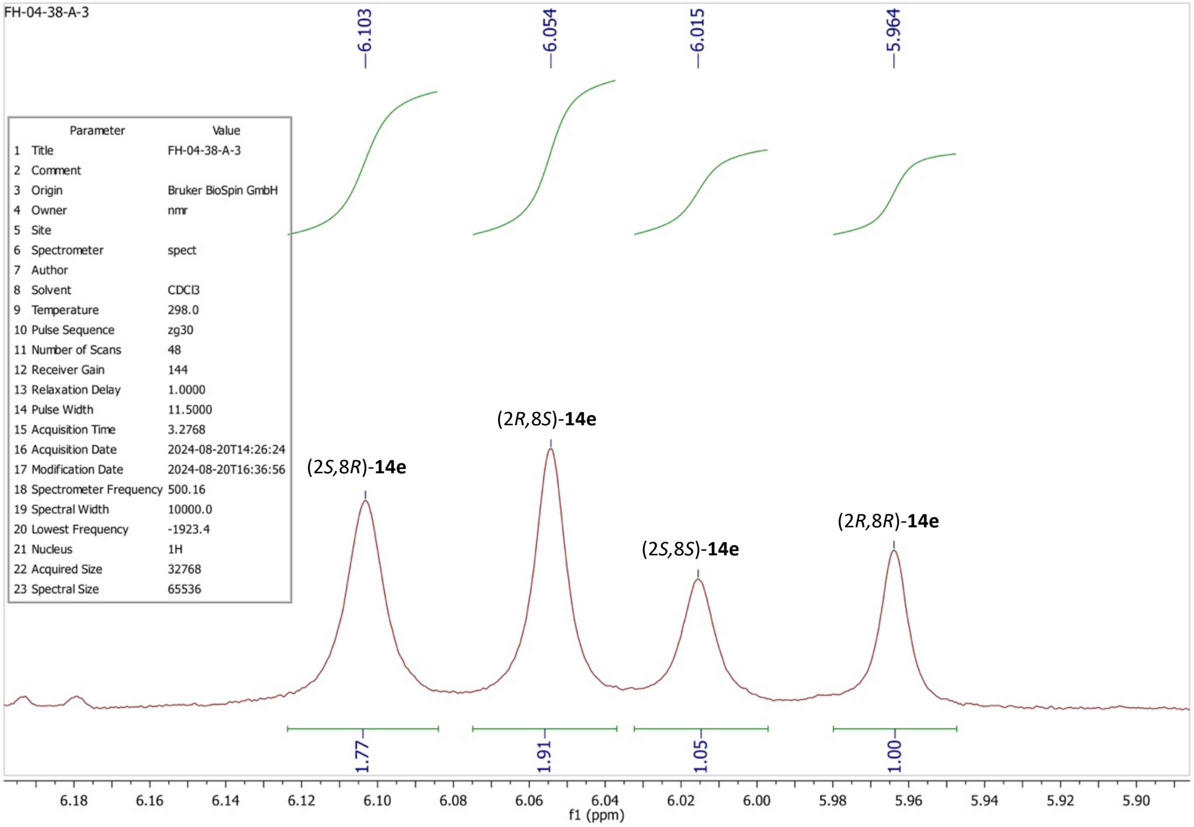

Determination of relative configuration of diastereomers of **14e**: relative configuration of diastereomers of **14e** was determined based on analogy to diastereomers of **14a**. In **14e** peaks at 5.86 (s, 1 H) and 5.81 (s, 1 H) ppm (proton at C2) correspond to major and minor diastereomers respectively. In **14a** peaks at 5.84 (s, 1 H) and 5.82 (s, 1 H) ppm (proton at C2) correspond to major and minor diastereomers respectively.

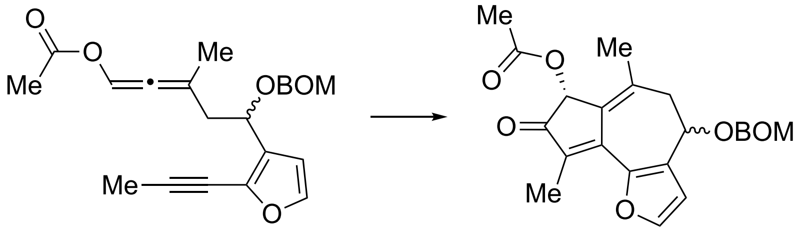

(±)-**13e** **14e**

Table 2, entry 8

(7*R*)-4-((benzyloxy)methoxy)-6,9-dimethyl-8-oxo-4,5,7,8-tetrahydroazuleno[4,5-*b*]furan-7-yl acetate (14e).

Follows general procedure **I**, bis(1,5-cyclooctadiene)rhodium(I) tetrafluoroborate (1.9 mg, 0.005 mmol), (*S*)-MonoPhos-alkene (*S*)-**4** (2.9 mg, 0.007 mmol), carbon monoxide (100%), (±)-5 ((benzyloxy)methoxy)-3-methyl-5-(2-(prop-1-yn-1-yl)furan-3-yl)penta-1,2-dien-1-yl acetate (±)-**13e** (18 mg, 0.05 mmol), DCE (4.7 mL, 0.01 M). The reaction was stirred for 22 h in an oil bath preheated to 70 °C. Polymer-bound triphenylphosphine (16 mg, 1.0 eq, 3 mmol/g). After 4 h at rt, the reaction mixture was filtered, and concentrated *in vacuo*. The crude residue was purified via silica gel flash chromatography (20% ethyl acetate/hexanes) to give 5 mg (26% yield) of the title compound as a yellow oil (dr (*trans*:*cis*) = 1.2:1 (54:46)) [er = 86:14 (2*R,*8*R*:2*S,*8*R*) and 79:21 (2*R,*8*S*:2*S,*8*S*) respectively].

^{1}H NMR (500 MHz, $CDCl_3$)

7.58 (s, 2 H), 7.36–7.30 (m, 10 H), 6.59 (s, 2 H), 5.86 (s, 1 H)*, 5.81 (s, 1 H)**, 4.94–4.82 (m, 6 H), 4.68–4.60 (m, 4 H), 2.88–2.71 (m, 4 H), 2.21 (s, 6 H), 2.14 (s, 6 H), 1.94 (s, 3 H)**, 1.92 (s, 3 H)* ppm

distinguishable diastereomeric peaks for minor isomer (*cis*) *

distinguishable diastereomeric peaks for major isomer (*trans*) **

* dr = 1.2:1 (54:46)

^{13}C NMR (100 MHz, $CDCl_3$)

199.7**, 199.6*, 170.0**, 169.9*, 148.7*, 148.6**, 146.6*, 146.3**, 145.0**, 145.0*, 137.8*, 137.7**, 134.0*, 133.5*, 133.4**, 133.4**, 132.4**, 132.3*,

128.7**, 128.7*, 128.0**, 128.0*, 128.0*, 127.9**, 112.9*, 112.7**, 93.3**, 93.3*, 71.8**, 71.6*, 70.1**, 70.0*, 68.8*, 68.8**, 40.9**, 40.6*, 29.8, 25.4**, 25.1*, 20.9*, 20.8**, 10.3*, 10.3** ppm

distinguishable diastereomeric peaks for minor isomer (*cis*) *

distinguishable diastereomeric peaks for major isomer (*trans*) **

IR	(Thin Film)
	2924, 2855, 1744, 1697, 1605, 1440, 1371, 1222, 1104, 1025 cm^{-1}
HRMS	HRMS-ESI (m/z): $[M + H]^+$ calcd for $C_{24}H_{25}O_6$, 409.1646; found, 409.1654
TLC	R_f = 0.30 (35% ethyl acetate/hexanes); silica gel, UV, *p*-anisaldehyde

Determination of enantiomeric ratio: Follows general procedure **A**, gradual addition of aliquots of the preprepared $Eu(hfc)_3/CDCl_3$ solution to **14e** in $CDCl_3$ showed the resonances for the two diastereomers at 5.86 and 5.81 ppm (proton at C2) splitting into four resonances at 6.20, 6.13, 6.10, and 6.02 ppm.

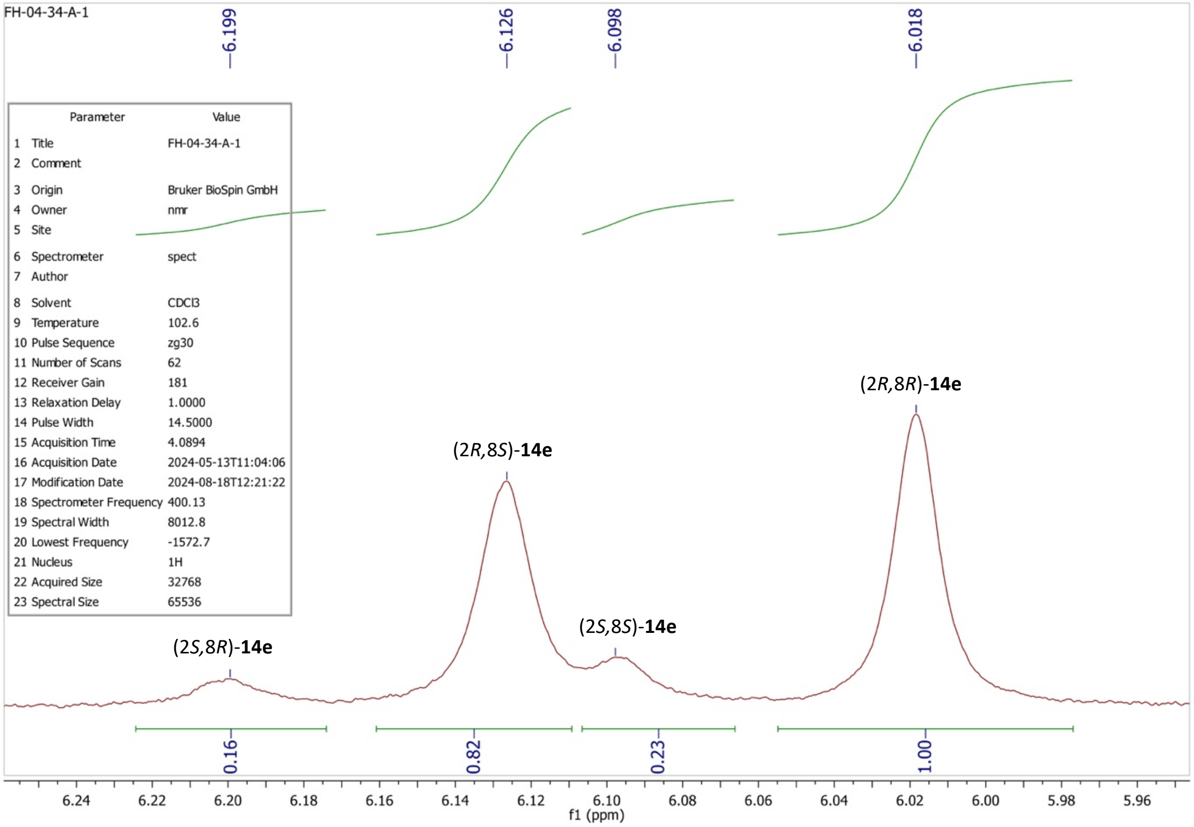

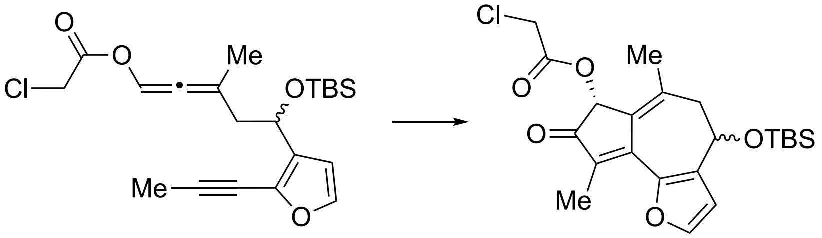

(±)-**13f** **14f**

Table S4, entry 1

(7*R*)-4-((*tert*-butyldimethylsilyl)oxy)-6,9-dimethyl-8-oxo-4,5,7,8-tetrahydroazuleno[4,5-b]furan-7-yl 2-chloroacetate (14f).

Follows general procedure **I**, bis(1,5-cyclooctadiene)rhodium(I) tetrafluoroborate (2.0 mg, 0.005 mmol), (*S*)-MonoPhos-alkene (*S*)-**4** (3.0 mg, 0.007 mmol), carbon monoxide (100%), (±)-5-((tert-butyldimethylsilyl)oxy)-3-methyl-5-(2-(prop-1-yn-1-yl)furan-3-yl)penta-1,2-dien-1-yl 2-chloroacetate (±)-**13f** (20 mg, 0.05 mmol), DCE (4.9 mL, 0.01 M). The reaction was stirred for 44 h in an oil bath preheated to 70 °C. The crude residue was purified via silica gel flash chromatography (3-10% ethyl acetate/hexanes) to give 2 mg (20% yield) of the title compound as a yellow oil (dr (*trans*:*cis*) = 1.4:1 (58:42)) [er = 77:23 (2*R*,8*R*:2*S*,8*R*) and 62:38 (2*R*,8*S*:2*S*,8*S*) respectively].

Note: Three aliquots (2.1 mL) were taken out of the reaction and considered for calculating isolated yield.

^{1}H NMR (400 MHz, $CDCl_3$)

7.58–7.58 (m, 2 H), 6.60 (d, *J* = 1.6 Hz, 1 H)**, 6.59 (d, *J* = 1.6 Hz, 1 H)*, 5.89 (s, 1 H)*, 5.85 (s, 1 H)**, 4.95–4.88 (m, 2 H), 4.15 (s, 2 H)**, 4.13 (s, 1 H)*, 4.13 (s, 1 H)*, 2.94–2.81 (m, 2 H), 2.54 (dd, *J* = 16.4, 2.8 Hz, 1 H)**, 2.46 (dd, *J* = 15.0, 2.4 Hz, 1 H)*, 2.21 (s, 3 H)*, 2.19 (s, 3 H)**, 1.96 (s, 3 H)*, 1.94 (s, 3 H)**, 0.95 (s, 9 H)**, 0.94 (s, 9 H)*, 0.15 (s, 6 H), 0.12 (s, 3 H)**, 0.10 (s, 3 H)* ppm

distinguishable diastereomeric peaks for minor isomer (*cis*) *

distinguishable diastereomeric peaks for major isomer (*trans*) **

* dr = 1.4:1 (58:42)

^{13}C NMR (75 MHz, $CDCl_3$)

198.5*, 198.4**, 166.5*, 166.4**, 149.2*, 149.1**, 145.1*, 145.0**, 144.6*, 144.2**, 137.2**, 137.2*, 135.1**, 133.9*, 133.1**, 132.8*, 127.5**, 127.1*,

112.2**, 112.0*, 73.2**, 72.9*, 65.5, 45.0*, 44.6**, 40.8*, 40.7**, 25.9*, 25.9**, 25.5**, 25.4*, 18.4, 10.3**, 10.1*, -4.6*, -4.6**, -4.7*, -4.7** ppm

distinguishable diastereomeric peaks for minor isomer (*cis*) *

distinguishable diastereomeric peaks for major isomer (*trans*) **

IR (Thin Film)

2954, 2926, 2855, 1749, 1700, 1603, 1254 cm^{-1}

HRMS HRMS-ESI (m/z): $[M + H]^+$ calcd for $C_{22}H_{30}O_5ClSi$, 437.1546; found, 437.1527

TLC R_f = 0.30 (10% ethyl acetate/hexanes); silica gel, UV, *p*-anisaldehyde

Determination of enantiomeric ratio: Follows general procedure **A**, gradual addition of aliquots of the preprepared $Eu(hfc)_3/CDCl_3$ solution to **14f** in $CDCl_3$ showed the resonances for the two diastereomers at 5.89 and 5.85 ppm (proton at C2) splitting into four resonances at 5.98, 5.96, 5.94, and 5.91 ppm.

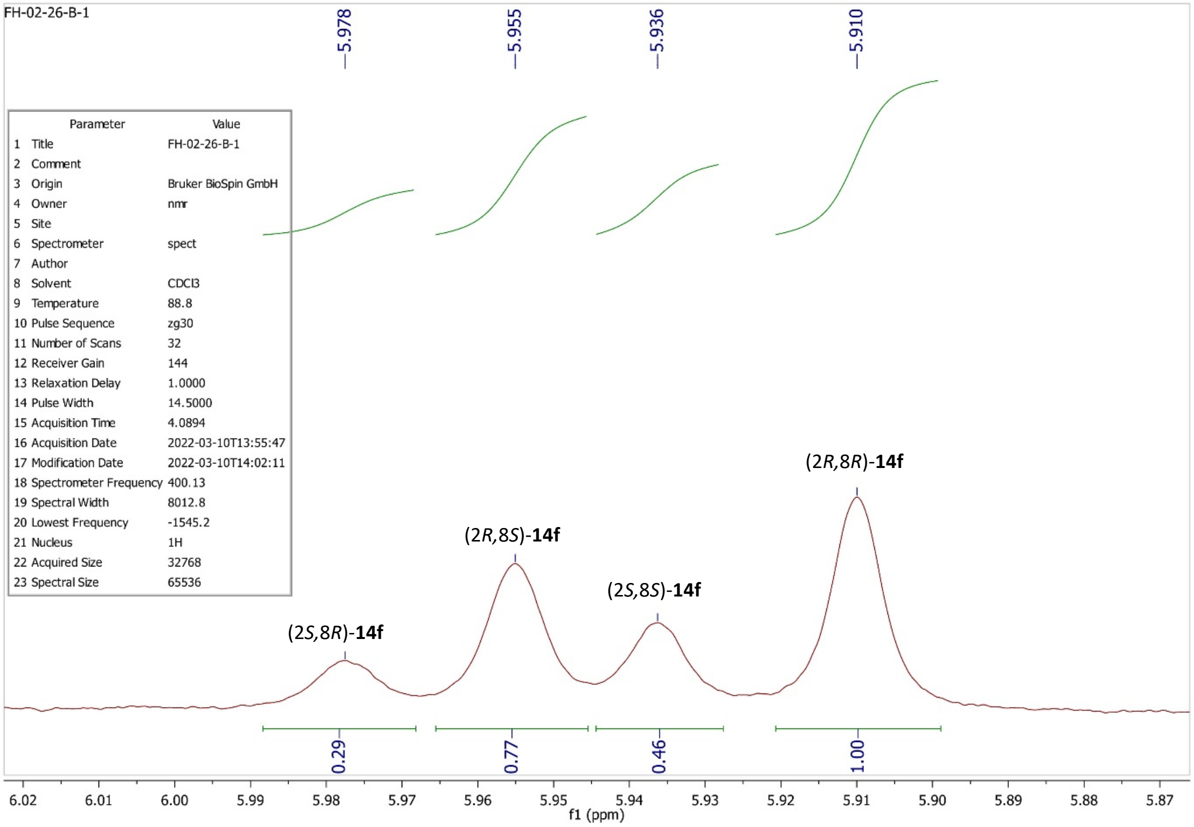

Determination of relative configuration of diastereomers of **14f**: relative configuration of diastereomers of **14f** was determined based on analogy to diastereomers of **14a**. In **14f** peaks at 5.89 (s, 1 H) and 5.85 (s, 1 H) ppm (proton at C2) correspond to minor and major diastereomers respectively. In **14a** peaks at 5.84 (s, 1 H) and 5.82 (s, 1 H) ppm (proton at C2) correspond to minor and major diastereomers respectively.

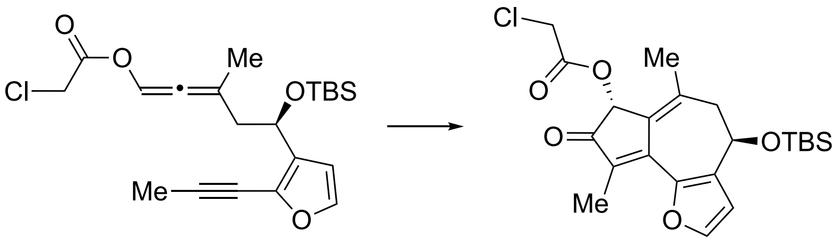

(8*R*)-**13f** (2*R*,8*R*)-**14f**

Table S4, entry 2

(4*R*,7*R*)-4-((*tert*-butyldimethylsilyl)oxy)-6,9-dimethyl-8-oxo-4,5,7,8-tetrahydroazuleno[4,5-b]furan-7-yl 2-chloroacetate ((2*R*,8*R*)-14f).

Follows general procedure **I**, Bis(1,5-cyclooctadiene)rhodium(I) tetrafluoroborate (0.9 mg, 0.002 mmol), (*S*)-MonoPhos-alkene (*S*)-**4** (1.4 mg, 0.003 mmol), carbon monoxide (100%), (5*R*)-5-((tert-butyldimethylsilyl)oxy)-3-methyl-5-(2-(prop-1-yn-1-yl)furan-3-yl)penta-1,2-dien-1-yl 2-chloroacetate (*R*)-**13f** (59.2% ee) (9 mg, 0.02 mmol), DCE (2.2 mL, 0.01 M). The reaction was stirred for 51 h in an oil bath preheated to 70 °C. The crude residue was purified via silica gel flash chromatography (3-10% ethyl acetate/hexanes) to give a trace amount of the title compound as a yellow oil (dr (*trans*:*cis*) = 2.2:1 (69:31)) [er = 78:22 (2*R,*8*R*:2*S,*8*R*) and 61:39 (2*R,*8*S*:2*S,*8*S*) respectively]. TLC and ^{1}H NMR data match those of **14f**.

Note: Due to the small scale a negative number was obtained for the mass, therefore yield calculation wasn't possible.

Determination of enantiomeric ratio: Following General Procedure **A**, gradual addition of aliquots of the preprepared Eu(hfc)$_3$/$CDCl_3$ solution to (2*R*,8*R*)-**14f** in $CDCl_3$ showed the resonances for the two diastereomers at 5.89 and 5.85 ppm (proton at C2) splitting into four resonances at 5.98, 5.96, 5.94, and 5.91 ppm.

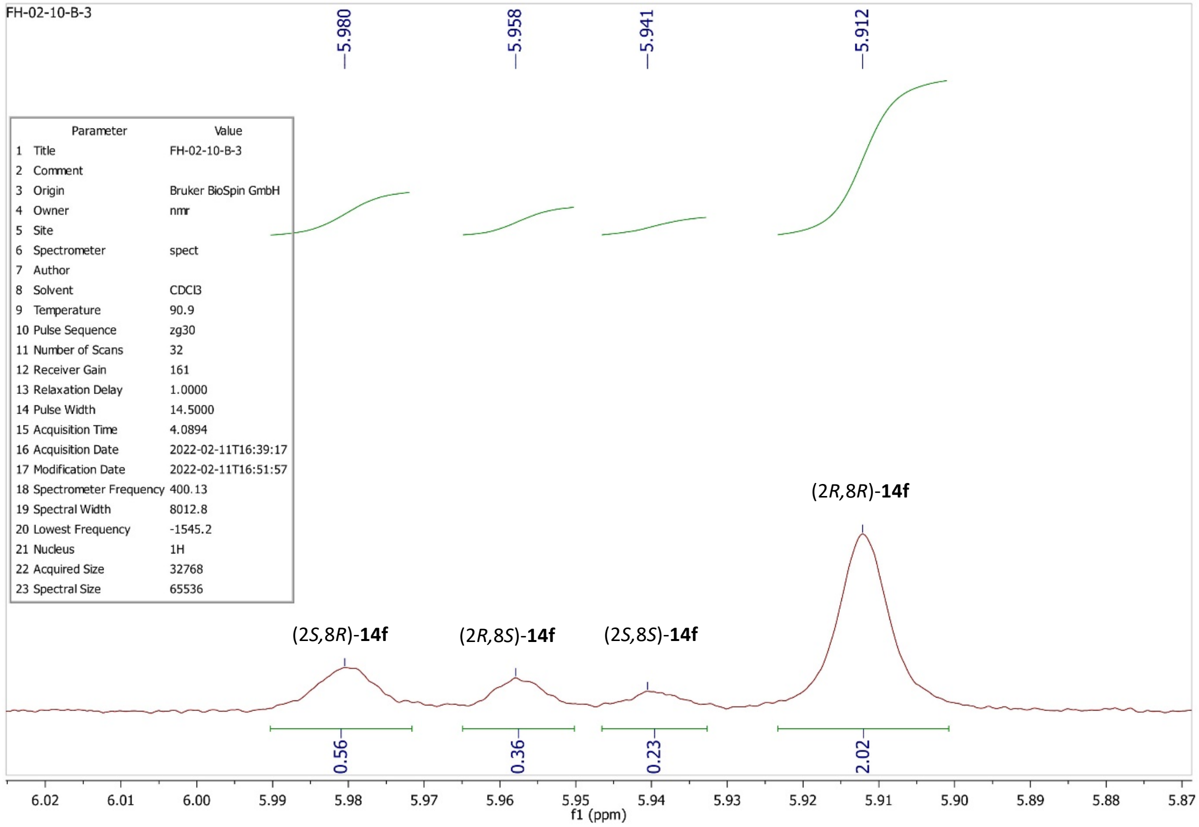

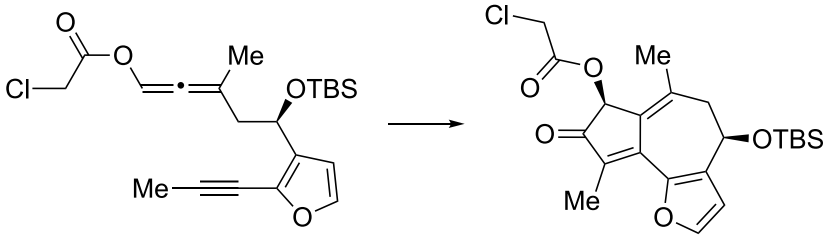

Table S4, entry 3

(4R,7S)-4-((*tert*-butyldimethylsilyl)oxy)-6,9-dimethyl-8-oxo-4,5,7,8-tetrahydroazuleno[4,5-b]furan-7-yl 2-chloroacetate ((2*S*,8*R*)-14f).

Follows General Procedure **I**, Bis(1,5-cyclooctadiene)rhodium(I) tetrafluoroborate (1.7 mg, 0.004 mmol), (*R*)-MonoPhos-alkene (*R*)-**4** (2.6 mg, 0.006 mmol), carbon monoxide (100%), (5*R*)-5-((tert-butyldimethylsilyl)oxy)-3-methyl-5-(2-(prop-1-yn-1-yl)furan-3-yl)penta-1,2-dien-1-yl 2-chloroacetate (*R*)-**13f** (59.2% ee) (17 mg, 0.04 mmol), DCE (4.2 mL, 0.01 M). The reaction was stirred for 44 h in an oil bath preheated to 70 °C. The crude residue was purified via silica gel flash chromatography (3-10% ethyl acetate/hexanes) to give 6 mg (34% yield) of the title compound as a yellow oil (dr (*trans*:*cis*) = 1:1.3 (44:56)) [er = 38:62 (2*R*,8*R*:2*S*,8*R*) and 74:26 (2*R*,8*S*:2*S*,8*S*) respectively]. TLC and ^{1}H NMR data match those of **14f**.

Determination of enantiomeric ratio: Follows general procedure **A**, gradual addition of aliquots of the preprepared $Eu(hfc)_3/CDCl_3$ solution to (2*S*,8*R*)-**14f** in $CDCl_3$ showed the resonances for the two diastereomers at 5.89 and 5.85 ppm (proton at C2) splitting into four resonances at 6.17, 6.14, 6.10, and 6.05 ppm.

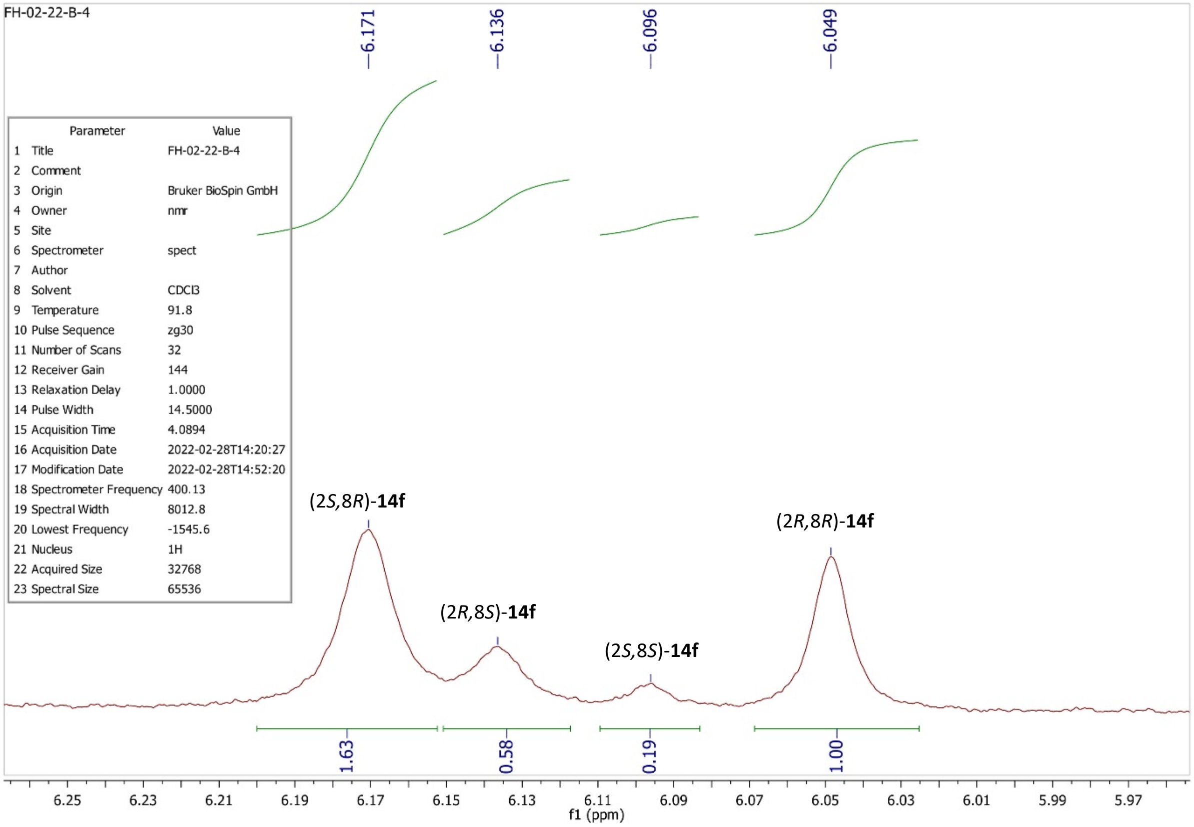

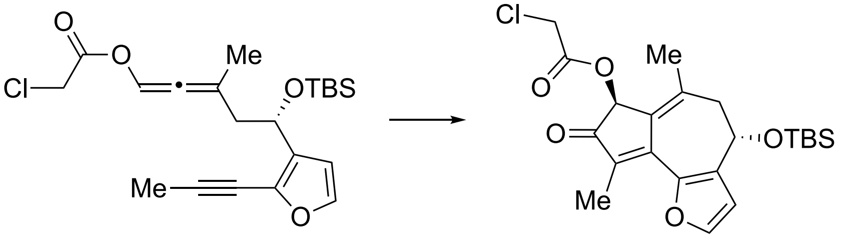

(8*S*)-**13f** (2*S*,8*S*)-**14f**

Table S4, entry 4

(4*S*,7*S*)-4-((*tert*-butyldimethylsilyl)oxy)-6,9-dimethyl-8-oxo-4,5,7,8-tetrahydroazuleno[4,5-b]furan-7-yl 2-chloroacetate ((2*S*,8*S*)-14f).

Follows General Procedure **I**, bis(1,5-cyclooctadiene)rhodium(I) tetrafluoroborate (4.7 mg, 0.01 mmol), (*R*)-MonoPhos-alkene (*R*)-**4** (7.1 mg, 0.02 mmol), carbon monoxide (100%), (5*S*)-5-((tert-butyldimethylsilyl)oxy)-3-methyl-5-(2-(prop-1-yn-1-yl)furan-3-yl)penta-1,2-dien-1-yl 2-chloroacetate (*S*)-**13f** (59.8% ee) (47 mg, 0.1 mmol), mesitylene (1 equiv, 0.02 mL, 0.1 mmol), DCE (11.5 mL, 0.01 M). The reaction was stirred for 68 h in an oil bath preheated to 70 °C. The crude residue was purified via silica gel flash chromatography (3-10% ethyl acetate/hexanes) to give 13 mg (26% yield) of the title compound as a yellow oil 9dr (*trans*:*cis*) = 2.4:1 (71:29)) [er = 40:60 (2*R,*8*R*:2*S,*8*R*) and 24:76 (2*R,*8*S*:2*S,*8*S*) respectively]. TLC and ^{1}H NMR data match those of **14f**.

Determination of enantiomeric ratio: Follows general procedure **A**, gradual addition of aliquots of the preprepared $Eu(hfc)_3/CDCl_3$ solution to (2*S*,8*S*)-**14f** in $CDCl_3$ showed the resonances for the two diastereomers at 5.89 and 5.85 ppm (proton at C2) splitting into four resonances at 6.00, 5.98, 5.96, and 5.93 ppm.

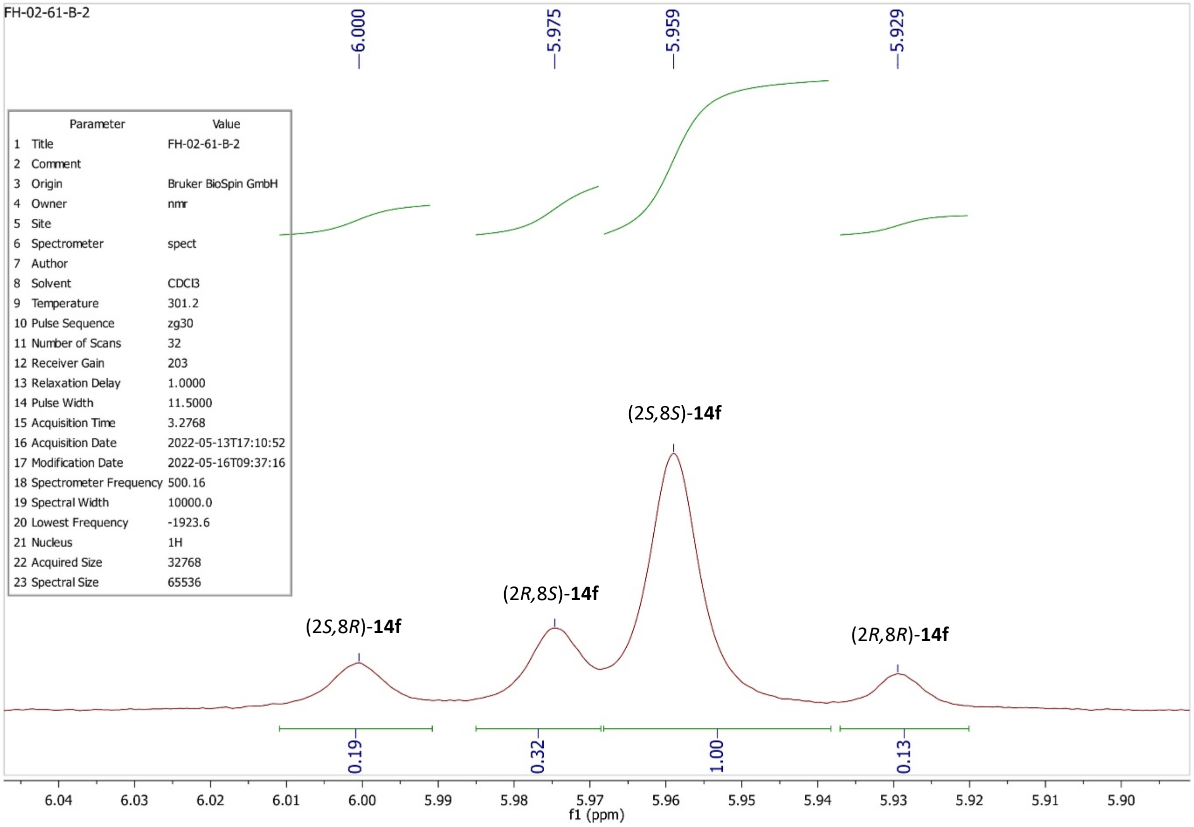

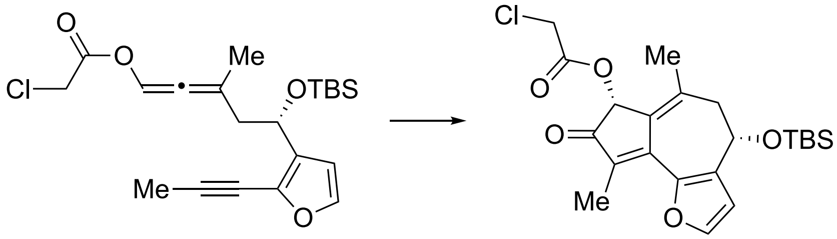

(8*S*)-**13f** (2*R*,8*S*)-**14f**

Table S4, entry 5

(4*S*,7*R*)-4-((*tert*-butyldimethylsilyl)oxy)-6,9-dimethyl-8-oxo-4,5,7,8-tetrahydroazuleno[4,5-b]furan-7-yl 2-chloroacetate ((2*R*,8*S*)-14f).

Follows General Procedure **I**, bis(1,5-cyclooctadiene)rhodium(I) tetrafluoroborate (2.5 mg, 0.006 mmol), (*S*)-MonoPhos-alkene (*S*)-**4** (3.8 mg, 0.009 mmol), carbon monoxide (100%), (5*S*)-5-((tert-butyldimethylsilyl)oxy)-3-methyl-5-(2-(prop-1-yn-1-yl)furan-3-yl)penta-1,2-dien-1-yl 2-chloroacetate (*S*)-**13f** (59.8% ee) (25 mg, 0.06 mmol), DCE (6.1 mL, 0.01 M). The reaction was stirred for 27 h in an oil bath preheated to 70 °C. The crude residue was purified via silica gel flash chromatography (3-10% ethyl acetate/hexanes) to give 3 mg (12% yield) of the title compound as a yellow oil (dr (*trans*:*cis*) = 1:1.1 (48:52)) [er = 83:17 (2*R*,8*R*:2*S*,8*R*) and 60:40 (2*R*,8*S*:2*S*,8*S*) respectively]. TLC and ^{1}H NMR data match those of **14f**.

Determination of enantiomeric ratio: Follows General Procedure **A**, gradual addition of aliquots of the preprepared $Eu(hfc)_3/CDCl_3$ solution to (2*R*,8*S*)-**14f** in $CDCl_3$ showed the resonances for the two diastereomers at 5.89 and 5.85 ppm (proton at C2) splitting into four resonances at 5.96, 5.94, 5.92, and 5.90 ppm.

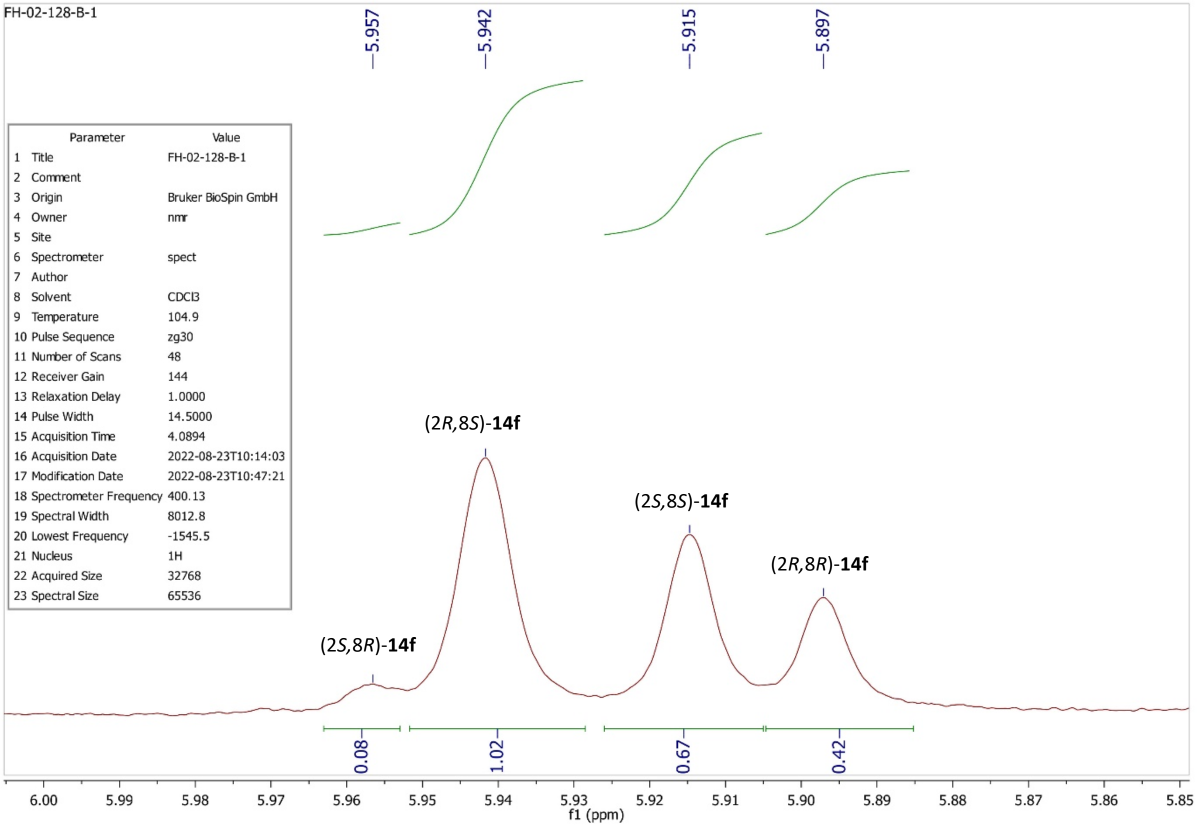

Shimadzu Nexera Series SCL-40, UV/PDA detector, 328 nm, CHIRALPAK IH-3, 250 X 4.6 mm column, 2% EtOH/hexanes, Flow rate: 1 mL/min

Peak	Retention Time (min)	Peak area (%)
Peak 1	6.06	17.89
Peak 2	6.55	29.94
Peak 3	7.89	47.32
Peak 4	9.10	4.86

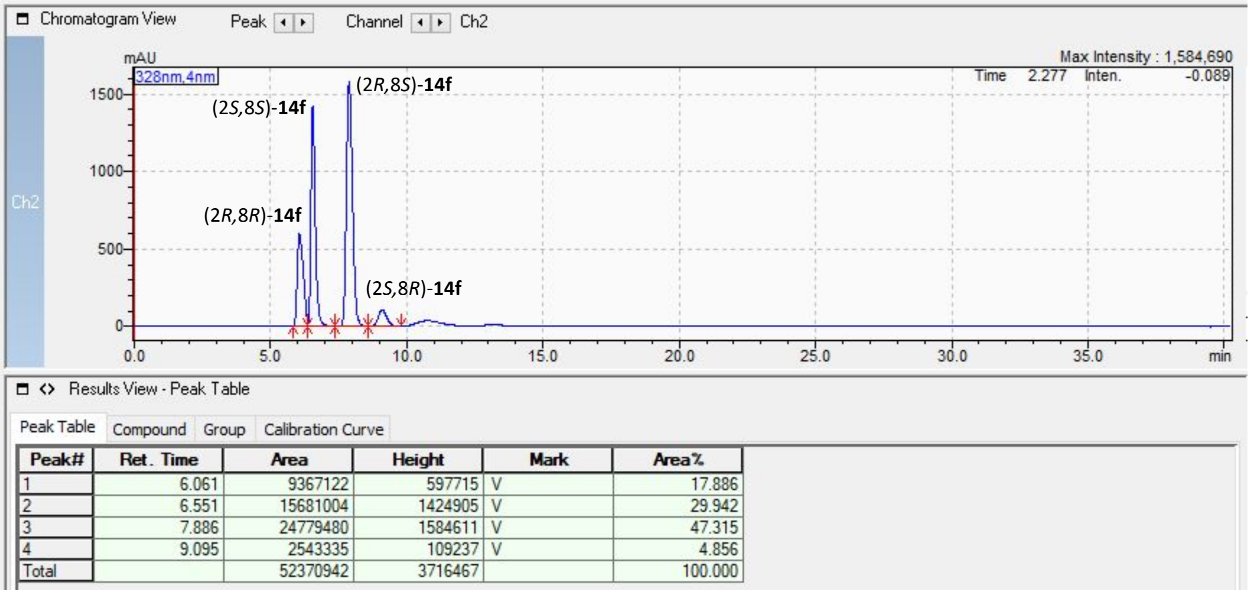

Peak#	Ret. Time	Area	Height	Mark	Area%
1	6.061	9367122	597715	V	17.886
2	6.551	15681004	1424905	V	29.942
3	7.886	24779480	1584611	V	47.315
4	9.095	2543335	109237	V	4.856
Total		52370942	3716467		100.000

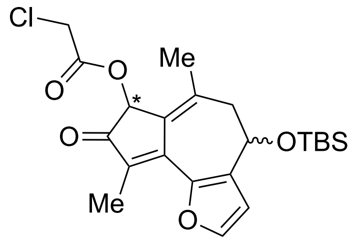

14f

4-((*tert*-butyldimethylsilyl)oxy)-6,9-dimethyl-8-oxo-4,5,7,8-tetrahydroazuleno[4,5-b]furan-7-yl 2-chloroacetate (14f).

(dr (*trans*:*cis*) = 1:1.4 (42:58))

Shimadzu Nexera Series SCL-40, UV/PDA detector, 328 nm, CHIRALPAK IH-3, 250 X 4.6 mm column, 2% EtOH/hexanes, Flow rate: 1 mL/min

Peak	Retention Time (min)	Peak area (%)
Peak 1	6.09	19.71
Peak 2	6.60	22.13
Peak 3	7.95	29.27

Peak 4	9.14	28.89

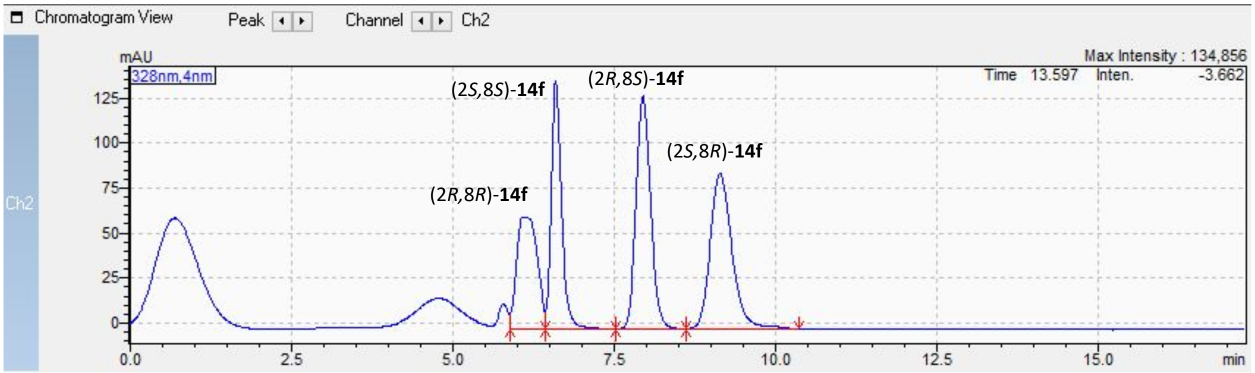

Results View - Peak Table

Peak Table | Compound | Group | Calibration Curve

Peak#	Ret. Time	Area	Height	Mark	Area%
1	6.092	1385654	62365		19.707
2	6.595	1555923	138388	V	22.129
3	7.947	2058012	129611	V	29.270
4	9.144	2031632	86551	M	28.894
Total		7031221	416914		100.000

Note: This HPLC trace is for racemic-**14f**, and was only used to determine the retention times.

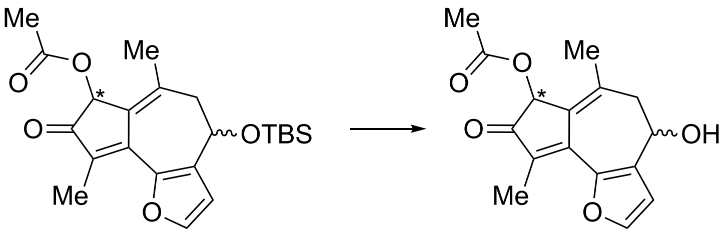

14a **14g**

General Procedure J: TBS deprotection of APKR product to separate diastereomers (TBAF)

4-hydroxy-6,9-dimethyl-8-oxo-4,5,7,8-tetrahydroazuleno[4,5-b]furan-7-yl acetate (14g).

This compound was prepared in a manner similar to that previously reported with some modifications.[12] A flame-dried 0.5-mL Biotage microwave reaction vial equipped with a magnetic

stir bar was charged with 4-((*tert*-butyldimethylsilyl)oxy)-6,9-dimethyl-8-oxo-4,5,7,8-tetrahydroazuleno[4,5-b]furan-7-yl acetate **14a** (1 equiv, 2 mg, 0.005 mmol) in THF (0.2 mL). The reaction vial was placed in an ice-bath and TBAF (2 equiv, 0.01 mL of a 1.0 M solution in THF, 0.01 mmol,) was added dropwise via syringe. The solution slowly warmed up to rt. After 90 min, TLC showed complete consumption of starting material. Saturated aqueous ammonium chloride (0.1 mL) and EtOAc (0.1 mL) were added to the vial. The organic layer was separated, and washed with brine (0.1 mL) and water (0.1 mL). The combined aqueous layers were back extracted with EtOAc (3 x 0.2 mL), dried over sodium sulfate, filtered, and concentrated *in vacuo*. The crude residue was purified by preparative TLC (50% ethyl acetate/hexanes, 1 run) to give 0.8 mg (57% yield) of the title compound as a yellow oil. (dr (*trans*:*cis*) = 1.3:1). Two diastereomers were separated by preparative TLC (50/5/45 chlorofom/ethanol/hexanes, 4 runs).[13]

Minor diastereomer (*cis*)

^{1}H NMR	(500 MHz, $CDCl_3$)
	7.60 (d, *J* = 1.5 Hz, 1 H), 6.64 (d, *J* = 1.5 Hz, 1 H), 5.76 (s, 1 H), 4.90 (dd, *J* = 8.8, 2.0 Hz, 1 H), 2.91 (dd, *J* = 15.8, 8.5 Hz, 1 H), 2.73–2.70 (m, 1 H), 2.22 (s, 3 H), 2.14 (s, 3 H), 1.97 (s, 3 H), 1.64 (brs, 1 H) ppm
^{13}C NMR	(100 MHz, $CDCl_3$)
	199.5, 170.0, 148.3, 145.8, 145.2, 134.8, 133.7, 132.8, 128.4, 112.2, 72.0, 64.7, 43.8, 25.2, 20.8, 10.3 ppm
IR	(Thin Film)
	3428, 2922, 1744, 1697, 1223 cm^{-1}
HRMS	HRMS-ESI (m/z): $[M + H]^+$ calcd for $C_{16}H_{17}O_5$, 289.1070; found, 289.1067
TLC	R_f = 0.24 (50% ethyl acetate/hexanes); silica gel, UV, *p*-anisaldehyde

Shimadzu Nexera Series SCL-40, UV/PDA detector, 328 nm, CHIRALPAK IB N-3, 150 X 4.6 mm column, 2% EtOH/hexanes, Flow rate: 1 mL/min

Peak	Retention Time (min)	Peak area (%)
Peak 1	54.09	49.42
Peak 2	66.61	50.58

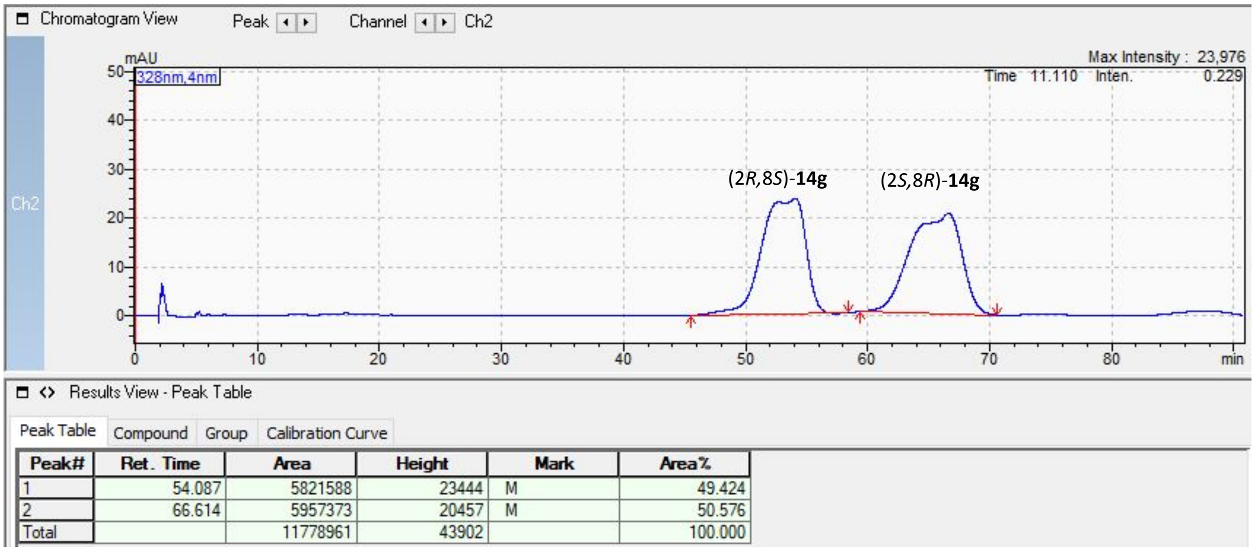

Note: A higher amount of the injection (0.04 mL) caused the shouldering of the peaks.

Major diastereomer (*trans*)

^{1}H NMR (400 MHz, $CDCl_3$)

7.60 (d, J = 1.6 Hz, 1 H), 6.66 (d, J = 1.6 Hz, 1 H), 5.80 (s, 1 H), 4.97 (dd, J = 9.0, 2.8 Hz, 1 H), 2.84 (dd, J = 15.8, 8.8 Hz, 1 H), 2.76 (dd, J = 15.6, 2.8 Hz, 1 H), 2.21 (s, 3 H), 2.14 (s, 3 H), 1.96 (s, 3 H), 1.62 (brs, 1 H) ppm

^{13}C NMR (100 MHz, $CDCl_3$)

199.6, 169.9, 148.6, 145.3, 145.3, 134.9, 133.5, 133.4, 128.2, 111.9, 71.7, 64.8, 43.8, 25.4, 20.9, 10.3 ppm

IR (Thin Film)

3424, 2924, 1741, 1678, 1230 cm^{-1}

HRMS HRMS-ESI (m/z): $[M + H]^+$ calcd for $C_{16}H_{17}O_5$, 289.1070; found, 289.1066

TLC R_f = 0.24 (50% ethyl acetate/hexanes); silica gel, UV, *p*-anisaldehyde

Shimadzu Nexera Series SCL-40, UV/PDA detector, 328 nm, CHIRALPAK IB N-3, 150 X 4.6 mm column, 2% EtOH/hexanes, Flow rate: 1 mL/min

Peak	Retention Time (min)	Peak area (%)
Peak 1	64.54	49.75
Peak 2	69.45	50.25

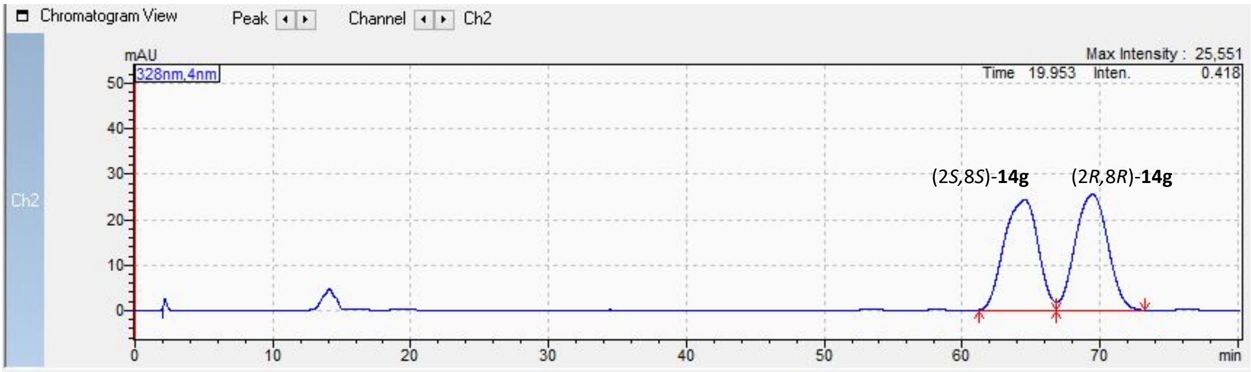

Peak#	Ret. Time	Area	Height	Mark	Area%
1	64.540	4145503	24337		49.753
2	69.449	4186721	25478	V	50.247
Total		8332224	49815		100.000

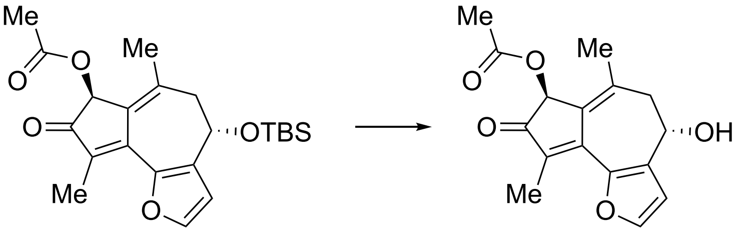

(2*S*,8*S*)-**14a** (2*S*,8*S*)-**14g**

Scheme 2C

(4*S*,7*S*)-4-hydroxy-6,9-dimethyl-8-oxo-4,5,7,8-tetrahydroazuleno[4,5-b]furan-7-yl acetate ((2*S*,8*S*)-14g).

Follows general procedure **J**, (4*S*,7*S*)-4-((*tert*-butyldimethylsilyl)oxy)-6,9-dimethyl-8-oxo-4,5,7,8-tetrahydroazuleno[4,5-b]furan-7-yl acetate (2*S*,8*S*)-**14a** (4 mg, 0.01 mmol), TBAF (0.02 mL of a 1.0 M solution in THF, 0.02 mmol), THF (0.3 mL). The reaction stirred for 1 h at 0 °C to rt. The mixture of diastereomers (dr (*trans*:*cis*) = 1.3:1) was separated by preparative TLC (50/5/45 chlorofom/ethanol/hexanes, 5 runs) to give 0.3 and 0.5 mg (29% yield) of minor (*cis*) and major (*trans*) diastereomers with er = 16.4:83.6 (2*S,*8*R*:2*R,*8*S*) and 83.2:16.8 (2*S,*8*S*:2*R,*8*R*) respectively. TLC and ^{1}H NMR data match those of **14g**.

Minor diastereomer (*cis*)

Shimadzu Nexera Series SCL-40, UV/PDA detector, 328 nm, CHIRALPAK IB N-3, 150 X 4.6 mm column, 2% EtOH/hexanes, Flow rate: 1 mL/min

Peak	Retention Time (min)	Peak area (%)
Peak 1	54.12	83.58
Peak 2	67.35	16.42

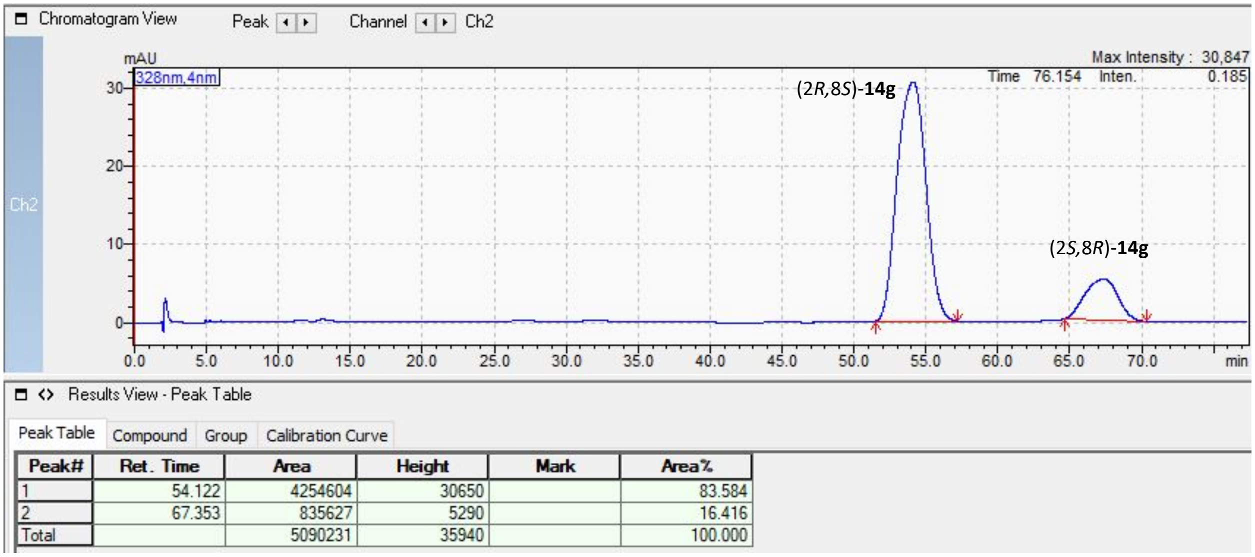

$[\alpha]^{20}_{D}$ = + 62.1° (c = 0.02, $CHCl_3$)

Major diastereomer (*trans*)

Shimadzu Nexera Series SCL-40, UV/PDA detector, 328 nm, CHIRALPAK IB N-3, 150 X 4.6 mm column, 2% EtOH/hexanes, Flow rate: 1 mL/min

Peak	Retention Time (min)	Peak area (%)
Peak 1	62.93	83.23
Peak 2	67.89	16.77

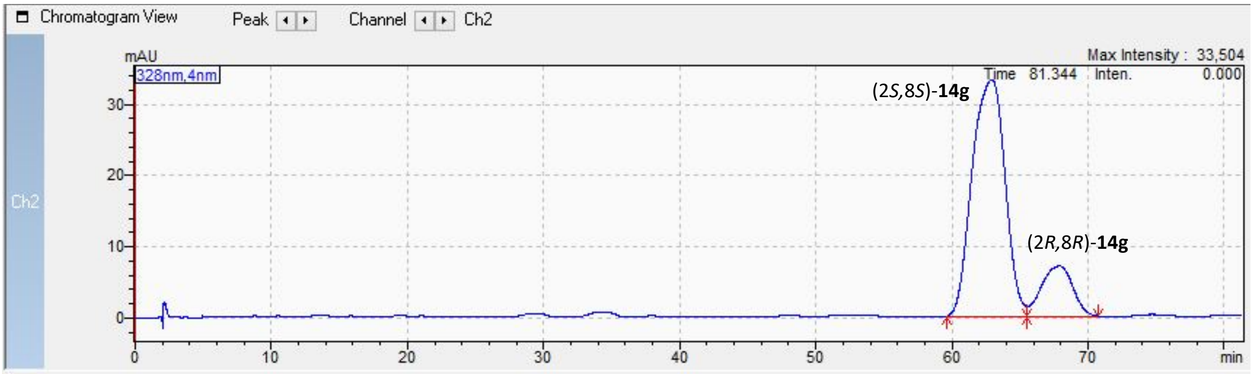

Results View - Peak Table

Peak Table | Compound | Group | Calibration Curve

Peak#	Ret. Time	Area	Height	Mark	Area%
1	62.928	5576815	33221		83.230
2	67.890	1123639	7042	V	16.770
Total		6700454	40263		100.000

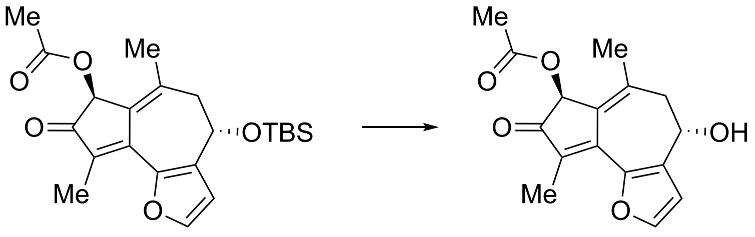

(2*S*,8*S*)-**14a** (2*S*,8*S*)-**14g**

General procedure K: TBS deprotection of APKR product to separate diastereomers (TBAF/AcOH)

Scheme 2A

(4*S*,7*S*)-4-hydroxy-6,9-dimethyl-8-oxo-4,5,7,8-tetrahydroazuleno[4,5-b]furan-7-yl acetate ((2*S*,8*S*)-14g).

This compound was prepared in a manner similar to that previously reported with some modifications.[14] A flame-dried, single-necked, 10-mL round-bottomed flask equipped with a magnetic stir bar was charged with (4*S*,7*S*)-4-((*tert*-butyldimethylsilyl)oxy)-6,9-dimethyl-8-oxo-4,5,7,8-tetrahydroazuleno[4,5-b]furan-7-yl acetate (2*S*,8*S*)-**14a** (1 equiv, 25 mg, 0.06 mmol) in

THF (1.2 mL). The flask was lowered in a precooled CryoCool bath (-10 °C). A solution of TBAF/AcOH (5:1) (1.1 equiv, 0.14 mL, 0.07 mmol) was added dropwise over 10 min. After 2 h, TLC showed complete consumption of starting material. Deionized water (1.0 mL) and EtOAc (2.5 mL) were added to the flask. The mixture was transferred to a separatory funnel, and the organic layer was separated. The aqueous layer was extracted with EtOAc (2 x 2.5 mL), washed with brine (1.5 mL), dried over sodium sulfate, filtered, and concentrated *in vacuo*. The mixture of diastereomers (dr (*trans*:*cis*) = 3.2:1) was separated by preparative TLC (50/5/45 chlorofom/ethanol/hexanes, 5 runs) to give 1 and 7 mg (44% yield) of minor (*cis*) and major (*trans*) diastereomers with er = 47.7:52.3 (2*S*,8*R*:2*R*,8*S*) and 94.7:5.3 (2*S*,8*S*:2*R*,8*R*) respectively. TLC and ^{1}H NMR data match those of **14g**.

Note: Solution of TBAF/AcOH (5:1) was prepared by dissolving AcOH (1 equiv, 0.02 mL, 0.3 mmol) in TBAF (5 equiv, 1.5 mL of a 1.0 M solution in THF, 1.5 mmol).

Minor diastereomer (*cis*)

Shimadzu Nexera Series SCL-40, UV/PDA detector, 328 nm, CHIRALPAK IB N-3, 150 X 4.6 mm column, 2% EtOH/hexanes, Flow rate: 1 mL/min

Peak	Retention Time (min)	Peak area (%)
Peak 1	56.10	52.29
Peak 2	68.90	47.71

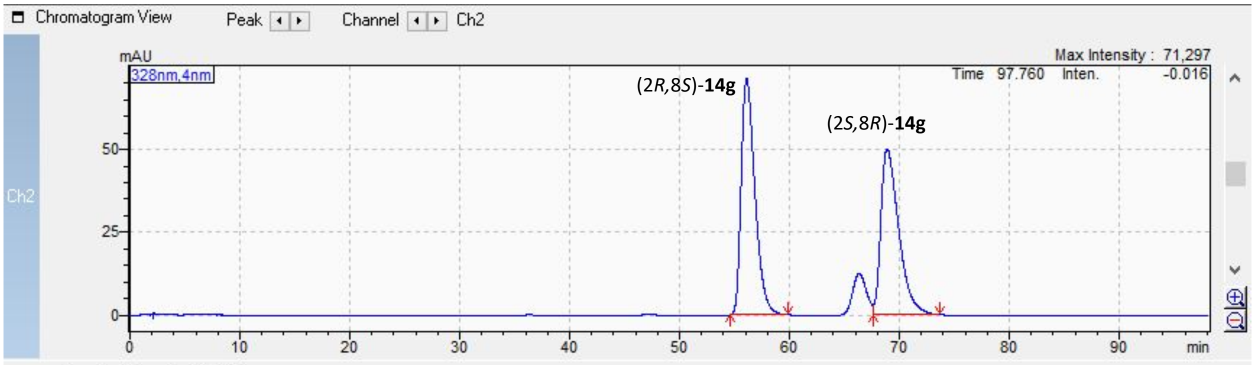

Results View - Peak Table

Peak Table | Compound | Group | Calibration Curve

Peak#	Ret. Time	Area	Height	Mark	Area%
1	56.103	6213315	71218		52.287
2	68.895	5669735	50120	V	47.713
Total		11883050	121338		100.000

Major diastereomer (*trans*)

Shimadzu Nexera Series SCL-40, UV/PDA detector, 328 nm, CHIRALPAK IB N-3, 150 X 4.6 mm column, 2% EtOH/hexanes, Flow rate: 1 mL/min

Peak	Retention Time (min)	Peak area (%)
Peak 1	65.18	94.70
Peak 2	70.82	5.30

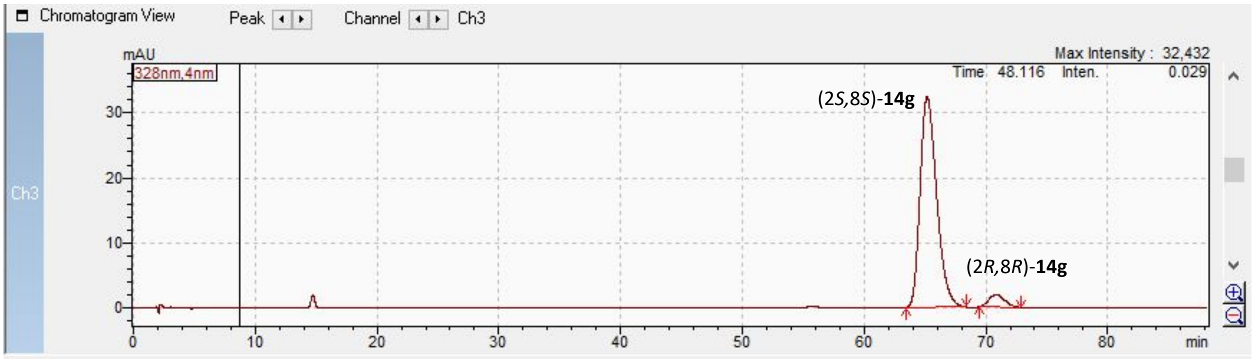

Results View - Peak Table

Peak Table | Compound | Group | Calibration Curve

Peak#	Ret. Time	Area	Height	Mark	Area%
1	65.177	3047777	32307		94.696
2	70.817	170695	1871		5.304
Total		3218472	34178		100.000

$[\alpha]^{20}_D$ = + 69.0° (c = 0.1, $CHCl_3$)

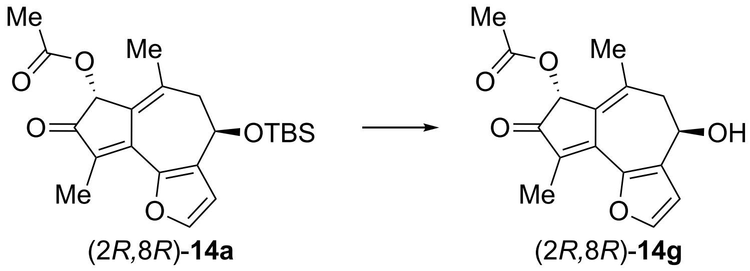

Scheme 2B

(4*R*,7*R*)-4-hydroxy-6,9-dimethyl-8-oxo-4,5,7,8-tetrahydroazuleno[4,5-b]furan-7-yl acetate ((2*R*,8*R*)-14g).

Follows general procedure **K**, (4*R*,7*R*)-4-((*tert*-butyldimethylsilyl)oxy)-6,9-dimethyl-8-oxo-4,5,7,8-tetrahydroazuleno[4,5-b]furan-7-yl acetate (2*R*,8*R*)-**14a** (25 mg, 0.06 mmol), TBAF/AcOH (5:1) (0.14 mL, 0.07 mmol), THF (1.2 mL). The reaction stirred for 30 min at -10 °C. The mixture of diastereomers (dr (*trans*:*cis*) = 2.2:1) was separated by preparative TLC (50/5/45 chlorofom/ethanol/hexanes, 5 runs) to give 2.5 and 7.5 mg (56% yield) of minor (*cis*) and major (*trans*) diastereomers with er = 70.5:29.5 (2*S*,8*R*:2*R*,8*S*) and 9.0:91.0 (2*S*,8*S*:2*R*,8*R*) respectively. TLC and ^{1}H NMR data match those of **14g**.

Minor diastereomer (*cis*)

Shimadzu Nexera Series SCL-40, UV/PDA detector, 328 nm, CHIRALPAK IB N-3, 150 X 4.6 mm column, 2% EtOH/hexanes, Flow rate: 1 mL/min

Peak	Retention Time (min)	Peak area (%)
Peak 1	51.03	29.49

Peak 2	62.17	70.51

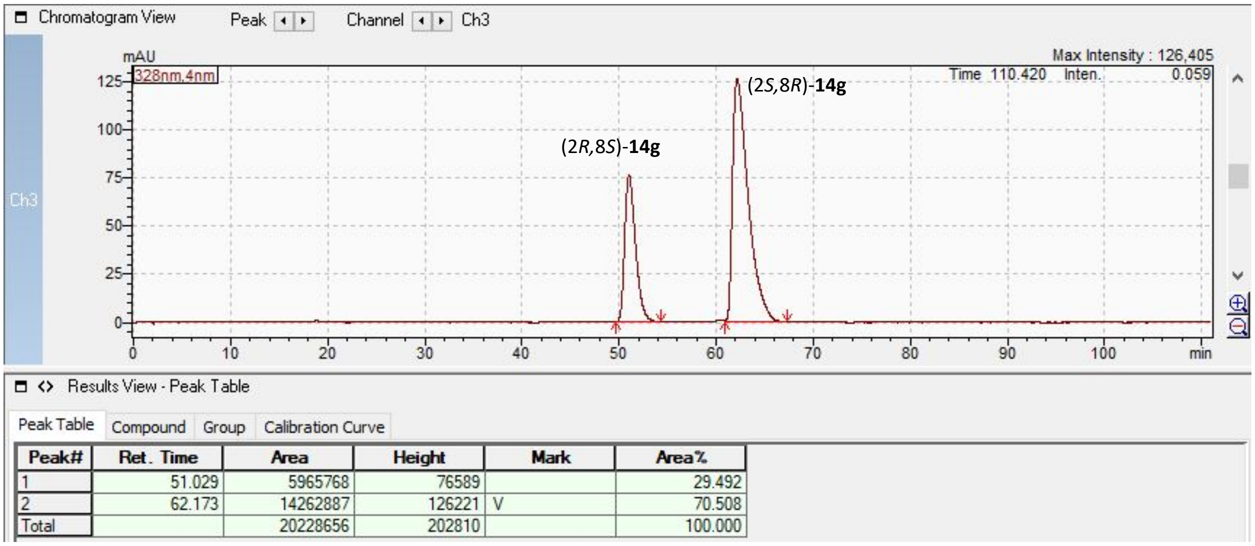

Major diastereomer (*trans*)

Shimadzu Nexera Series SCL-40, UV/PDA detector, 328 nm, CHIRALPAK IB N-3, 150 X 4.6 mm column, 2% EtOH/hexanes, Flow rate: 1 mL/min

Peak	Retention Time (min)	Peak area (%)
Peak 1	60.74	9.04
Peak 2	64.19	90.96

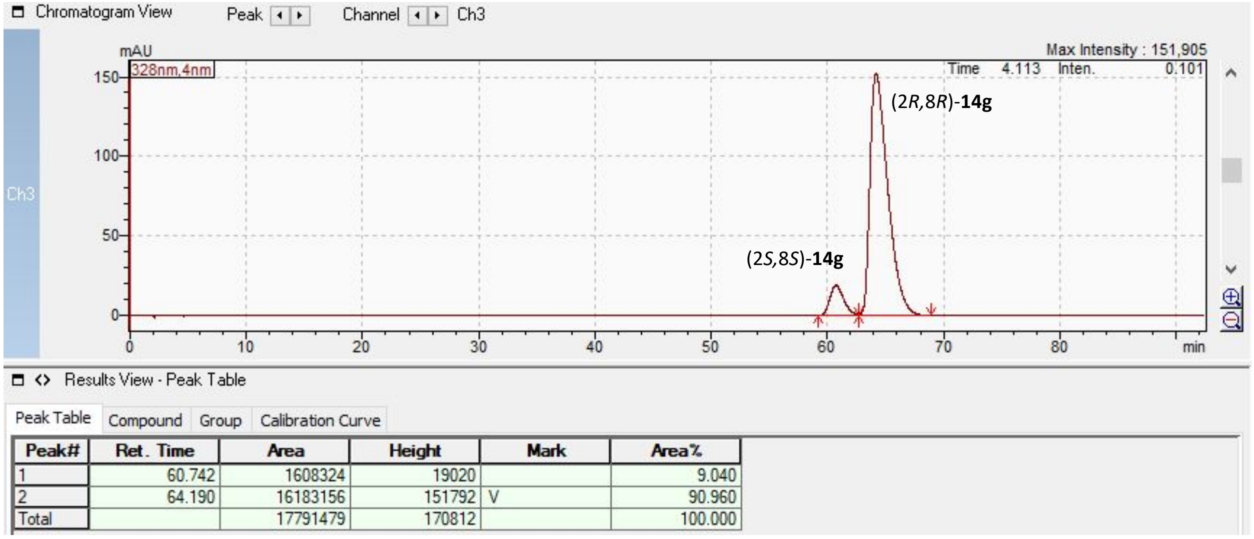

Peak#	Ret. Time	Area	Height	Mark	Area%
1	60.742	1608324	19020		9.040
2	64.190	16183156	151792	V	90.960
Total		17791479	170812		100.000

$[\alpha]^{20}_D$ = - 388.8° (c = 0.1, $CHCl_3$)

Aldehyde byproduct of the asymmetric allenic Pauson–Khand reaction

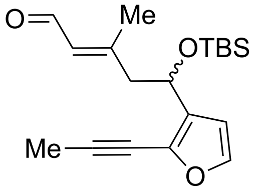

S8

5-((*tert*-butyldimethylsilyl)oxy)-3-methyl-5-(2-(prop-1-yn-1-yl)furan-3-yl)pent-2-enal (S8). Obtained as a 1:1 ratio of isomers as a byproduct of the asymmetric allenic Pauson–Khand reaction that was partially separable by column chromatography.

Single diasteremoer

^{1}H NMR (400 MHz, $CDCl_3$)

9.91 (d, J = 8.4 Hz, 1 H), 7.24 (s, 1 H), 6.41–6.41 (m, 1 H), 5.94 (d, J = 8.0 Hz, 1 H), 4.94 (dd, J = 8.2, 4.4 Hz, 1 H), 3.06 (dd, J = 13.2, 8.4 Hz, 1 H), 2.69 (dd, J =

13.0, 4.4 Hz, 1 H), 2.12 (s, 3 H), 2.01 (s, 3 H), 0.83 (s, 9 H), 0.00 (s, 3 H), 0.12 (s, 3 H) ppm

^{13}C NMR (100 MHz, $CDCl_3$)

191.5, 159.9, 142.9, 133.6, 131.9, 130.6, 109.4, 94.2, 69.0, 66.8, 42.3, 26.3, 25.9, 18.2, 4.7, -4.8, -5.1 ppm

Acetone at 207.1, 31.1 ppm

IR (Thin Film)

2930, 2857, 1678, 1254 cm^{-1}

HRMS HRMS-ESI (m/z): $[M + H]^+$ calcd for $C_{19}H_{29}O_3Si$, 333.1880; found, 333.1881

TLC R_f = 0.70 (35% ethyl acetate/hexanes); silica gel, UV, *p*-anisaldehyde

Synthesis of (±)-MonoPhos-alkene ((±)-4)

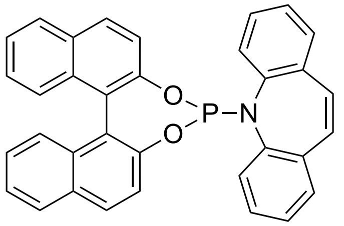

(±)-**4**

5-(dinaphtho[2,1-d:1',2'-f][1,3,2]dioxaphosphepin-4-yl)-5H-dibenzo[b,f]azepine ((±)-4).

This compound was prepared in a manner similar to that previously reported with some modifications.[15]

Note: In the purification step, the pure catalyst was obtained via silica gel flash chromatography (30% toluene/hexanes) instead of solvent trituration.

Synthesis of (*S*)-MonoPhos-alkene ((*S*)-4)

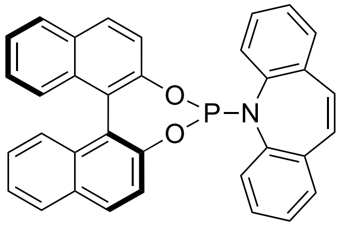

(*S*)-**4**

5-((11bS)-dinaphtho[2,1-d:1',2'-f][1,3,2]dioxaphosphepin-4-yl)-5H-dibenzo[b,f]azepine ((*S*)-4).

This compound was prepared in a manner similar to that previously reported with some modifications.[15]

Note: In the purification step, the pure catalyst was obtained via silica gel flash chromatography (30% toluene/hexanes) instead of solvent trituration.

Synthesis of (*R*)-MonoPhos-alkene ((*R*)-4)

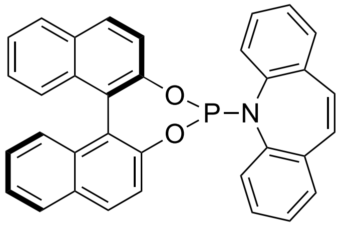

(*R*)-**4**

5-((11bR)-dinaphtho[2,1-d:1',2'-f][1,3,2]dioxaphosphepin-4-yl)-5H-dibenzo[b,f]azepine ((*R*)-4).

This compound was prepared in a manner similar to that previously reported with some modifications.[15]

Note: In the purification step, the pure catalyst was obtained via silica gel flash chromatography (30% toluene/hexanes) instead of solvent trituration.

Reaction optimization of asymmetric aldol (proline)

Reaction of 2-(prop-1-yn-1-yl)furan-3-carbaldehyde **16** with L-proline (30 mol%) and *(S)*-BINOL[3] (1 mol%) in acetone and DMSO at 0 °C for 72 h afforded a ratio of 50:30:20 of starting material (**SM**) **16**:aldol adduct (**AA**) **17**:aldol condensation (**AC**) **S10**. Because there was so much SM, the reaction was allowed to warm to rt and maintained for 24 h, which resulted in the consumption of some **SM** but afforded considerably more **AC** product. The desired product **AA** was obtained in 5% yield and 52% ee (Table **S1**, entry 1). Increasing the amount of acetone from 8 equiv to 25 equiv and *(S)*-BINOL from 1 mol% to 2 mol% led to an increase in the isolated yield of **AA** to 33% with no significant impact on ee (entry 2). To test whether turnover for proline was an issue, we used 100 mol% of L-proline and 7 mol% *(S)*-BINOL which resulted in slight increase in the isolated yield of **AA** (44%) with no significant impact on ee (entry 3). To test whether temperature would impact the ee, the reaction was maintained at -5 °C, which led to a 30% yield brsm with a slightly improved ee (compare entries 2 and 4). D-proline afforded the enantiomeric product in 50% yield and 64% ee. D-proline afforded **AA** in similar yield brsm to L-proline, which indicates there is no matched-mismatched interaction between proline and BINOL (compare entries 2 and 5). Additionally, the er's for this reaction were monitored by $Eu(hfc)_3$ ^{1}H-NMR, which showed no change at two time points (24 and 310 h) (entry 5). Using the original List

Procedure[16], adding 113 equiv of DMSO and eliminating (*S*)-BINOL resulted in the formation of **AC** byproduct as major product (entry 6). Inspired by Wennemers procedure[17], the reaction using D-proline using only acetone at 0 °C afforded **SM** and **AA** with no **AC** as determined by ^{1}H NMR! However, warming the reaction to rt, resulted in the conversion of all the **SM** to both **AA** and **AC** (compare entries 7 and 5). The addition of (*S*)-BINOL to the reaction lowered the isolated yield of **AA** but did not greatly affect the product ratios (compare entries 7 and 8). Keeping the reaction at 0 °C, addition of (*S*)-BINOL does not impact the yield and ee of **AA** (compare entries 9 and 10). Performing reaction with no DMSO slowed down the reaction due to heterogeneity (compare entries 5 and 10). Using (L) or (D)-proline resulted in similar yield and ee of **AA** (compare entries 9 and 11). Increasing the amount of acetone from 25 equiv to 100 equiv slowed the reaction due to a low concentration, and **AA** was obtained in a lower yield (compare entries 5 and 12).[18]

Table S1. Reaction optimization of asymmetric aldol (proline)

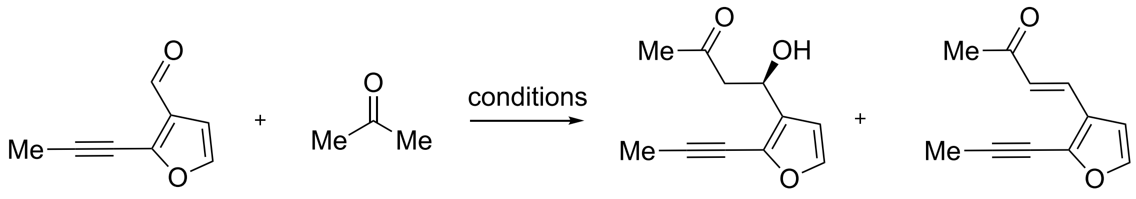

entry	scale (mg **SM**)	acetone equiv	additive	conditions[a]	reaction temperature and time	isolated yield **AA** (%)	er (%ee)[b]	**SM**[c] **16**	**AA:AC**[c] **17:S10**
1	69	8	*(S)*-BINOL 1 mol%	L-proline DMSO (3 equiv)	0 °C, 72 h **rt, 24h**	5<br>8 brsm	76:24 (52)	50<br>26	30:20<br>32:42
2	350	**25**	*(S)*-BINOL 2 mol%	L-proline, DMSO (6 equiv)	0 °C, 120 h	33<br>56 brsm	75:25 (50)	41[d]	38:21[d]
3	35	20	*(S)*-BINOL 7 mol%	**L-proline (100 mol%)**, DMSO (19 equiv)	0 °C, 116 h	44	78:22 (56)	0	62:38
4	67	25	*(S)*-BINOL 2 mol%	L-proline DMSO (6 equiv)	**-5 °C, 164 h**	16<br>30 brsm	79:21 (58)	44	43:13
**5**	**519**	**25**	***(S)*-BINOL 2 mol%**	**D-proline DMSO (6 equiv)**	**0 °C, 310 h**	**50<br>58 brsm**	**18:82 (-64)**	**13**	**67:20**

6	47	33	-	D-proline **DMSO (113 equiv)**	0 °C to rt, 20 h	-	-	0	17:83
7	50	30	-	D-proline	0 °C, 71 h<br><br>rt, 90 h	-<br><br>47	22:78 (-56)<br>23:77 (-54)	77<br><br>0	23:0<br><br>77:23
8	50	30	***(S)*-BINOL 2 mol%**	D-proline	0 °C, 71 h<br><br>rt, 70h	-<br><br>39	22:78 (-56)<br>24:76 (-52)	77<br><br>0	23:0<br><br>71:29
9	50	30	-	D-proline	0 °C, 310 h	33	21:79 (-58)	59	41:0
10	50	30	*(S)*-BINOL 2 mol%	D-proline	**0 °C**, 310 h	35	22:78 (-56)	59	41:0
11	50	30	-	L-proline	0 °C, 310 h	34	19:81 (-62)	59	41:0
12	50	**100**	*(S)*-BINOL 2 mol%	D-proline DMSO (6 equiv)	0 °C, 310 h	33	21:79 (-58)	50	42:8

[a]30 mol% proline is used for all entries except for that of entry 3. [b]ee was determined using Eu(hfc)$_3$ as an NMR shift reagent. [c]ratios are based on integrative values of peaks at 5.20 ppm **AA** and 6.54 ppm **AC** by ^{1}H-NMR benchmarked against peak at 9.98 ppm **SM** [d]ratios are based on isolated **SM**, **AA,** and **AC**.

Reaction optimization of asymmetric aldol (*β*-tosylamidoprolineamide)

Reaction of 2-(prop-1-yn-1-yl)furan-3-carbaldehyde **16** with *β*-tosylamidoprolineamide[4] (5 mol%) in acetone (27 equiv) at -35 °C for 24 h afforded (*R*)-**17** with 12% conversion. After 146 h, only 2% increase in conversion was observed (Table **S2**, entry 1). By increasing the temperature to 0 °C, (*R*)-**17** was obtained with 39% conversion (entry 2). Increasing the catalyst loading to 10 mol% resulted in a slightly lower conversion (entry 3). By addition of the catalyst in portions (3 x 5 mol% every 24 h), conversion increased to 89% (entry 4).

Table S2. Reaction optimization of asymmetric aldol (*β*-tosylamidoprolineamide)

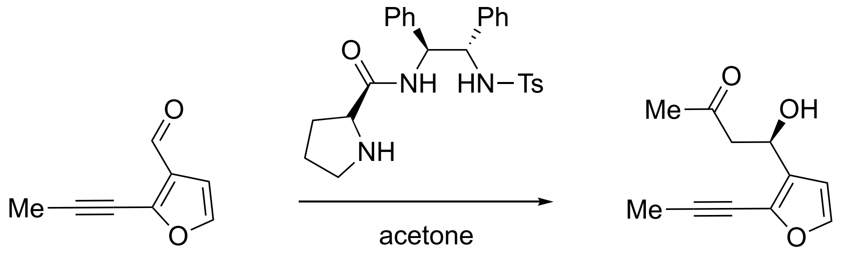

entry	catalyst mol%	t °C	conversion %[a]	ee %[b]
1	5	**-35**	12[c]	≥99
2	5	**0**	39	≥99
3	**10**	0	26	≥99
4	**15**	0	89[d]	≥99

[a]Percent conversion was calculated based on integrative value of peak at 5.20 ppm **pdt** by ^{1}H-NMR benchmarked against peak at 9.98 ppm **SM** after 24 h on a 10 mg scale. [b]Ee was determined using chiral HPLC. [c]14% conversion after 146 h. [d]5 mol% catalyst was added every 24 h.

Synthesis of *β*-tosylamidoprolineamide

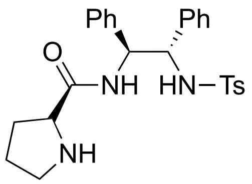

S12

(*R*)-*N*-((1*S*,2*S*)-2-((4-methylphenyl)sulfonamido)-1,2-diphenylethyl)pyrrolidine-2 carboxamide (S12).

This compound was prepared using a procedure entirely analogous to that previously reported.[4]

Attempts to form derivatives of 17 to get crystals for the assignment of absolute configuration

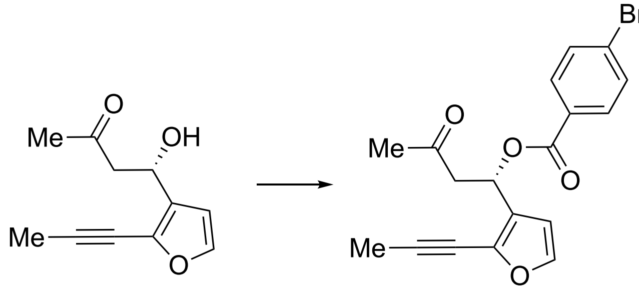

(*S*)-**17** (*S*)-**S13**

(*S*)-3-oxo-1-(2-(prop-1-yn-1-yl)furan-3-yl)butyl 4-bromobenzoate ((*S*)-S13).

This compound was prepared in a manner similar to that previously reported with some modifications.[19] Solution A: a flame-dried, single-necked, 5-mL, round-bottomed flask equipped with a magnetic stir bar was charged with 4-bromobenzoyl chloride (1.1 equiv, 22 mg, 0.1 mmol). The flask was lowered in a precooled CryoCool bath (-40 °C). Et_3N (2 equiv, 0.03 mL, 0.2 mmol), and DCM (0.4 mL) were added. Solution B: a flame-dried 2-mL Biotage microwave reaction vial equipped with a magnetic stir bar was charged with 4,4-dimethylaminopyridine (1.1 equiv, 13 mg, 0.1 mmol). The vial was lowered in a precooled CryoCool bath (-40 °C). (*S*)-4-hydroxy-4-(2-(prop-1-yn-1-yl)furan-3-yl)butan-2-one (*S*)-**17** (59.8% ee) (1 equiv, 18 mg, 0.1 mmol) in DCM (0.5 mL) was added dropwise. Solution A was added dropwise via syringe over 10 min to solution B. After 90 min at -40 °C, TLC showed complete consumption of starting material. The mixture was added to deionized water (1 mL) in a separatory funnel. The organic layer was separated. The aqueous layer was extracted with DCM (3 x 2 mL), dried over sodium sulfate, filtered, and concentrated *in vacuo*. The crude residue was purified by silica gel flash chromatography (10% ethyl acetate/hexanes) to give 7 mg (20%) of the title compound as a yellow oil.

Note: Since (*S*)-**S13** was an oil, X-ray crystallography wasn't possible.

Note: Attempts to eliminate the grease by dissolving (*S*)-**S13** in ethanol and filtering through a cotton plug were unsuccessful.

^{1}H NMR (400 MHz, $CDCl_3$)

7.88 (d, *J* = 8.4 Hz, 2 H), 7.56 (d, *J* = 8.4 Hz, 2 H), 7.26 (s, 1 H), 6.46–6.42 (m, 2 H), 3.25 (dd, *J* = 16.6, 8.0 Hz, 1 H), 3.02 (dd, *J*= 16.4, 5.6 Hz, 1 H), 2.20 (s, 3 H), 2.08 (s, 3 H) ppm

^{13}C NMR (100 MHz, $CDCl_3$)

204.3, 164.8, 142.9, 135.3, 131.8, 131.4, 129.0, 128.4, 126.5, 109.9, 94.4, 69.0, 65.9, 48.5, 30.5, 4.8 ppm

IR (Thin Film)

2958, 2925, 2854, 1720, 1266 cm^{-1}

HRMS HRMS-ESI (m/z): $[M + Na]^+$ calcd for $C_{18}H_{15}O_4BrNa$, 397.0046; found, 397.0046

TLC R_f = 0.44 (35% ethyl acetate/hexanes); silica gel, UV, *p*-anisaldehyde

$[\alpha]^{20}_{D}$ = + 31.0° (*c* = 0.4, $CHCl_3$)

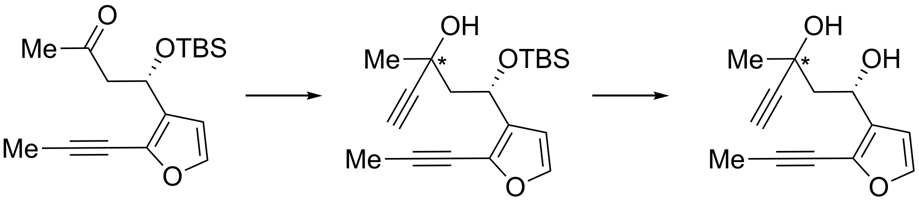

(*S*)-**18a** (*S*)-**S14** (*S*)-**S15**

(5*S*)-5-((*tert*-butyldimethylsilyl)oxy)-3-methyl-5-(2-(prop-1-yn-1-yl)furan-3-yl)pent-1-yn-3-ol ((*S*)-S14).

(*S*)-4-((tert-butyldimethylsilyl)oxy)-4-(2-(prop-1-yn-1-yl)furan-3-yl)butan-2-one (*S*)-**18a** (59.8% ee) (49 mg, 0.2 mmol) was diluted with toluene and concentrated using high vacuum (3 x 0.5 mL). A flame-dried, single-necked, 5-mL, roun-bottomed flask equipped with a magnetic stir bar was charged with (*S*)-4-((tert-butyldimethylsilyl)oxy)-4-(2-(prop-1-yn-1-yl)furan-3-yl)butan-2-one (*S*)-**18a** (1 equiv, 49 mg, 0.2 mmol) in THF (0.6 mL). The reaction flask was placed in an ice-bath and ethynylmagnesium bromide (1.0 mL of a 0.5 M solution in THF, 0.5 mmol, 3 equiv), was added dropwise via syringe over 1 min. The solution slowly warmed up to rt. After 90 min, TLC showed complete consumption of starting material. The mixture was transferred to a separatory funnel, diethyl ether (1 mL) and deionized water (1 mL) were added, and the organic layer was separated. The aqueous layer was extracted with diethyl ether (3 x 3 mL), and washed with brine (5 mL), the combined aqueous layers were back extracted with diethyl ether (1 x 5 mL), dried over magnesium sulfate, filtered, and concentrated *in vacuo*. The crude residue was purified by silica gel flash chromatography (9% ether/hexanes) to give 27 mg (51% yield) of the *major diastereomer*-**S14** as a colorless oil and 10 mg (19% yield) of the *minor diastereomer*-**S14** as a colorless oil.

Major diastereomer

^{1}H NMR (500 MHz, $CDCl_3$)

7.25 (d, J = 2.0 Hz, 1 H), 6.42 (d, J = 2.0 Hz, 1 H), 5.44 (dd, J = 11.2, 2.5 Hz, 1 H), 5.12 (s, 1 H), 2.53 (s, 1 H), 2.12 (s, 3 H), 2.11 (dd, J= 14.2, 11.0 Hz, 1 H), 1.75 (dd, J= 14.2, 2.5 Hz, 1 H), 1.49 (s, 3 H), 0.87 (s, 9 H), 0.17 (s, 3 H), -0.16 (s, 3 H) ppm

^{13}C NMR (125 MHz, $CDCl_3$)

142.9, 134.0, 131.2, 109.9, 94.2, 87.2, 71.9, 68.9, 67.7, 67.4, 49.1, 30.6, 25.9, 18.0, 4.7, -4.3, -5.1 ppm

IR (Thin Film)

3479, 3304, 2954, 2931, 2858, 2220, 1675, 1257 cm^{-1}

HRMS HRMS-ESI (m/z): $[M - H]^+$ calcd for $C_{19}H_{27}O_3Si$, 331.1724; found, 331.1717

TLC R_f = 0.65 (35% ethyl acetate/hexanes); silica gel, UV, *p*-anisaldehyde

Minor diastereomer

^{1}H NMR (500 MHz, $CDCl_3$)

7.25 (d, *J* = 2.0 Hz, 1 H), 6.44 (d, *J* = 2.0 Hz, 1 H), 5.17 (dd, *J* = 9.2, 4.0 Hz, 1 H), 3.93 (s, 1 H), 2.41 (s, 1 H), 2.38 (dd, *J*= 14.5, 9.5 Hz, 1 H), 2.12 (s, 3 H), 1.93 (dd, *J*= 14.5, 4.0 Hz, 1 H), 1.61 (s, 3 H), 0.86 (s, 9 H), 0.11 (s, 3 H), -0.17 (s, 3 H) ppm

^{13}C NMR (125 MHz, $CDCl_3$)

143.0, 133.9, 131.4, 110.0, 94.2, 88.0, 70.8, 69.0, 67.3, 65.5, 49.5, 29.1, 25.9, 18.0, 4.7, -4.4, -5.1 ppm

IR (Thin Film)

3456, 3302, 2958, 2929, 2854, 1254 cm^{-1}

TLC R_f = 0.55 (35% ethyl acetate/hexanes); silica gel, UV, *p*-anisaldehyde

$[\alpha]^{20}_D$ = - 23.6° (*c* = 0.8, $CHCl_3$)

(1*S*)-3-methyl-1-(2-(prop-1-yn-1-yl)furan-3-yl)pent-4-yne-1,3-diol ((*S*)-S15).

Follows general procedure **J**, (5*S*)-5-((*tert*-butyldimethylsilyl)oxy)-3-methyl-5-(2-(prop-1-yn-1-yl)furan-3-yl)pent-1-yn-3-ol *major diastereomer*-**S14** (59.8% ee) (27 mg, 0.08 mmol), TBAF (0.2

mL of a 1.0 M solution in THF, 0.2 mmol), THF (2.8 mL). The reaction stirred for 1 h at 0 °C to rt. The crude residue was purified via silica gel flash chromatography (20-25% ethyl acetate/hexanes) to give the title compound (15 mg, 83%) as a colorless oil with some white crystals in a 20.1:79.9 ratio of enantiomers.

Note: Attempts to recrystallize (*S*)-**S15** from ethyl acetate were unsuccessful.

^{1}H NMR (300 MHz, $CDCl_3$)

7.27 (s, 1 H), 6.44 (d, *J* = 1.8 Hz, 1 H), 5.48 (dd, *J* = 11.1, 2.4 Hz, 1 H), 4.43 (brs, 1 H), 2.70 (brs, 1 H), 2.55 (s, 1 H), 2.14 (dd, *J*= 14.4, 11.1 Hz, 1 H), 2.11 (s, 3H), 1.88 (dd, *J*= 14.4, 2.4 Hz, 1 H), 1.55 (s, 3 H) ppm

^{13}C NMR (125 MHz, $CDCl_3$)

143.0, 134.1, 131.0, 109.3, 94.3, 87.0, 72.1, 68.9, 68.2, 66.3, 48.1, 31.0, 4.8 ppm

IR (Thin Film)

2980, 2926, 1661 cm^{-1}

HRMS HRMS-ESI (m/z): $[M - OH]^+$ calcd for $C_{13}H_{13}O_2$, 201.0910; found, 201.0912

TLC R_f = 0.29 (35% ethyl acetate/hexanes); silica gel, UV, *p*-anisaldehyde

Shimadzu Nexera Series SCL-40, UV/PDA detector, 254 nm, CHIRALPAK IB N-3, 150 X 4.6 mm column, 5% EtOH/hexanes, Flow rate: 1 mL/min

Peak	Retention Time (min)	Peak area (%)
Peak 1	7.05	20.13
Peak 2	8.34	79.87

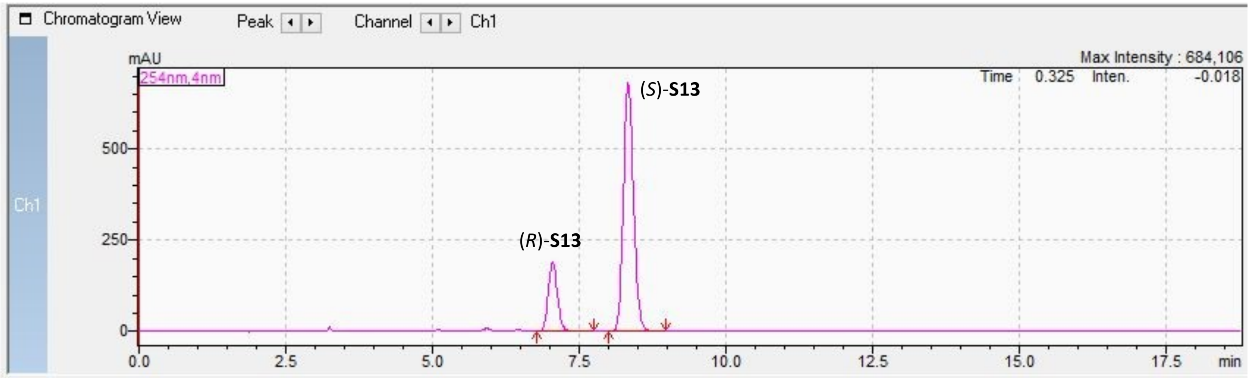

Results View - Peak Table

Peak Table | Compound | Group | Calibration Curve

Peak#	Ret. Time	Area	Height	Mark	Area%
1	7.052	2089739	191200	V	20.128
2	8.339	8292581	684105		79.872
Total		10382320	875305		100.000

$[\alpha]^{20}_D$ = -19.0° (*c* = 0.1, $CHCl_3$)

Asymmetric PKR of 13f to form 14f, selective synthesis of stereoisomers

Reaction of (±)-**13f** with $Rh(cod)_2BF_4$ and (*S*)-**4** in DCE under a CO atmosphere (100%) at 70 °C afforded the PKR product **14f** in low yield (20% yield, de = 16%) with ers = 77:23 and 62:38 for 8*R* and 8*S* respectively as determined by ^{1}H NMR using $Eu(hfc)_3$ as a chiral shift reagent (Table S4, entry 1). It was determined that (2*R*,8*R*)-**14f** was afforded as the major stereoisomer. Reacting enantioenriched (*R*)-**13f** with (*S*)-**4** gave **14f** (de = 38%) with ers = 78:22 and 61:39 (entry 2). Reaction of enantioenriched (*R*)-**13f** with (*R*)-**4** afforded **14f** in 34% yield (de = -12%) with ers = 38:62 and 74:26 (entry 3). Reaction of enantioenriched (*S*)-**13f** with (*R*)-**4**, **14f** was formed in 26% yield with de = 42%, ers = 40:60 and 24:76 with (2*S*,8*S*)-**14f** as the major stereoisomer having the same stereochemistry as thapsigargin at C2 and C8 (entry 4). Reaction of enantioenriched (*S*)-**13f** with (*S*)-**4** afforded **14f** in 12% yield (de = -4%), ers = 83:17 and 60:40 along with significant quantities of aldehyde side product (entry 5). By controlling the chirality of the allene-yne **13f** and the ligand **4**, all 4 stereoisomers of **14f** were formed stereoselectively (entries 2-5). We later

confirmed that the measured ers were the same when using chiral shift reagent or chiral HPLC (entry 5).

Table S3. Asymmetric PKR of **13f** to form **14f**, selective synthesis of stereoisomers

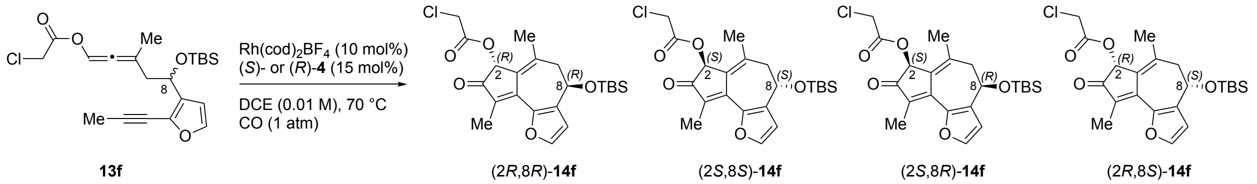

entry	**13f** er *R*:*S*	ligand **4**	isolated yield %	*trans*:*cis*[a] (de %)	2*R*8*R*:2*S*8*R*[b]	2*R*8*S*:2*S*8*S*	*RR*:SS:2*S*8*R*:2*R*8*S* ratio
1	50:50	*S*	20	58:42 (16)	77:23	62:38	**40**:18:12:30
2	75:25	*S*	-[c]	69:31 (38)	78:22	61:39	**64**:7:18:11
3	75:25	*R*	34	44:56 (-12)	38:62	74:26	29:6:**48**:17
4	20:80	*R*	26	71:29 (42)	40:60	24:76	8:**61**:12:19
5[d]	20:80	*S*	12	48:52 (-4)	83:17	60:40	19:31:4:**47**

[a]Based on crude ^{1}H NMR. [b]er was determined using $Eu(hfc)_3$ as NMR shift reagent. [c]Yield was not determined because reaction was performed on 9 mg.[d]er = 37:63 and 9:91 via chiral HPLC.

Matched and mismatched cases in PKR of allene-yne 13f

Investigating the matched and mismatched between allene-yne **13f** and MonoPhos-alkene **4** gave access to the stereoisomers of **14f** selectively. In matched cases, the diastereoselectvity was higher, affording the *trans*-**14f** as the major diastereomer. In mismatched cases, the diastereoselectivity was low with the *cis*-**14f** formed as the major diastereomer (Figure S1). Diastereoselectivity of matched and mismatched cases can be explained by the transition state energies of the oxidative cyclization step. For example, in the matched case—(*R*)-substrate/(*S*)-catalyst—**TS1a** leading to the *trans* diastereomer is lower in energy than **TS1b** leading to *cis* diastereomer (Figure 4). For the mismatched case—(*S*)-substrate/(*S*)-catalyst—**TS2a** leading to the *cis* diastereomer is lower in energy than **TS2b** leading to *trans* diastereomer (Figure 5).

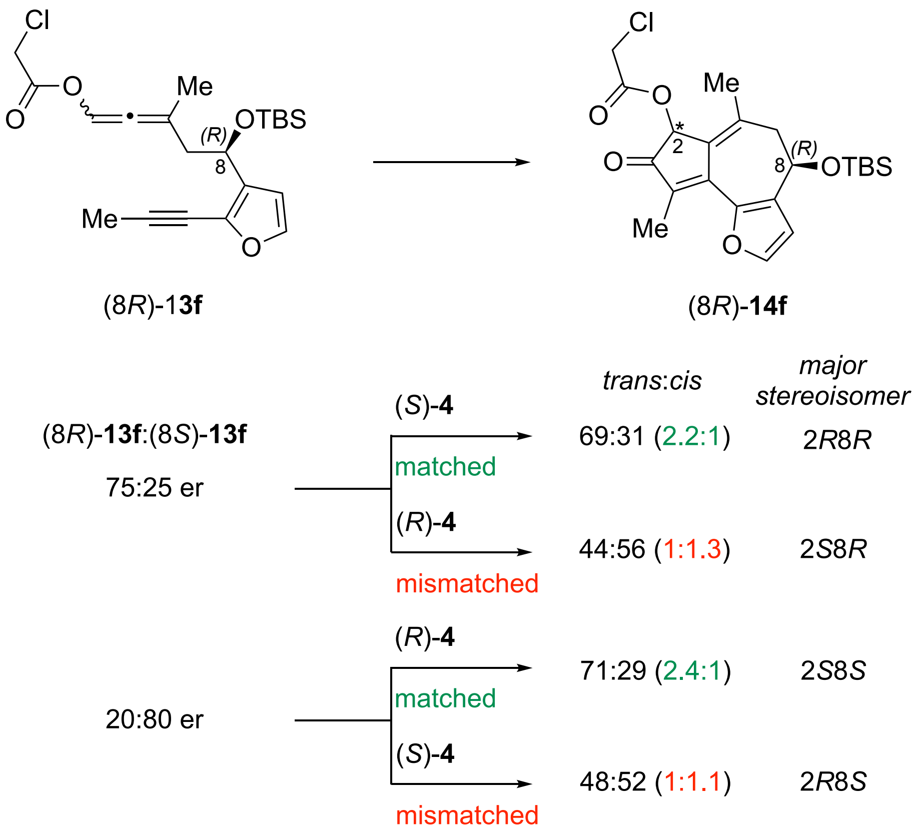

Figure S1. Matched and mismatched cases in PKR of allene-yne **13f**.

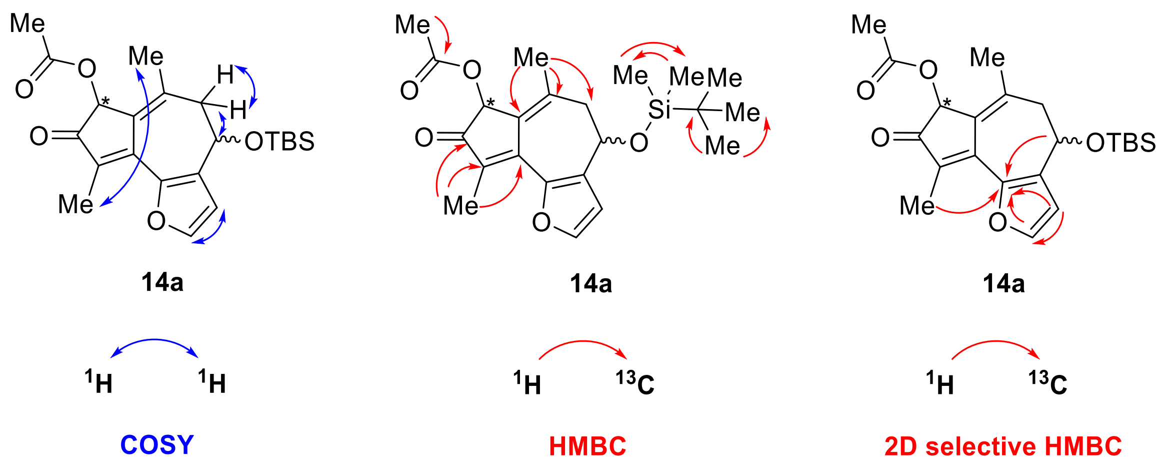

Figure S2. COSY and HMBC correlations of **14a**.

Note: Compound numbering is based on Spartan "set standard labels".

Note: *t*=*trans* and *c*=*cis*.

COSY spectrum of **14a**

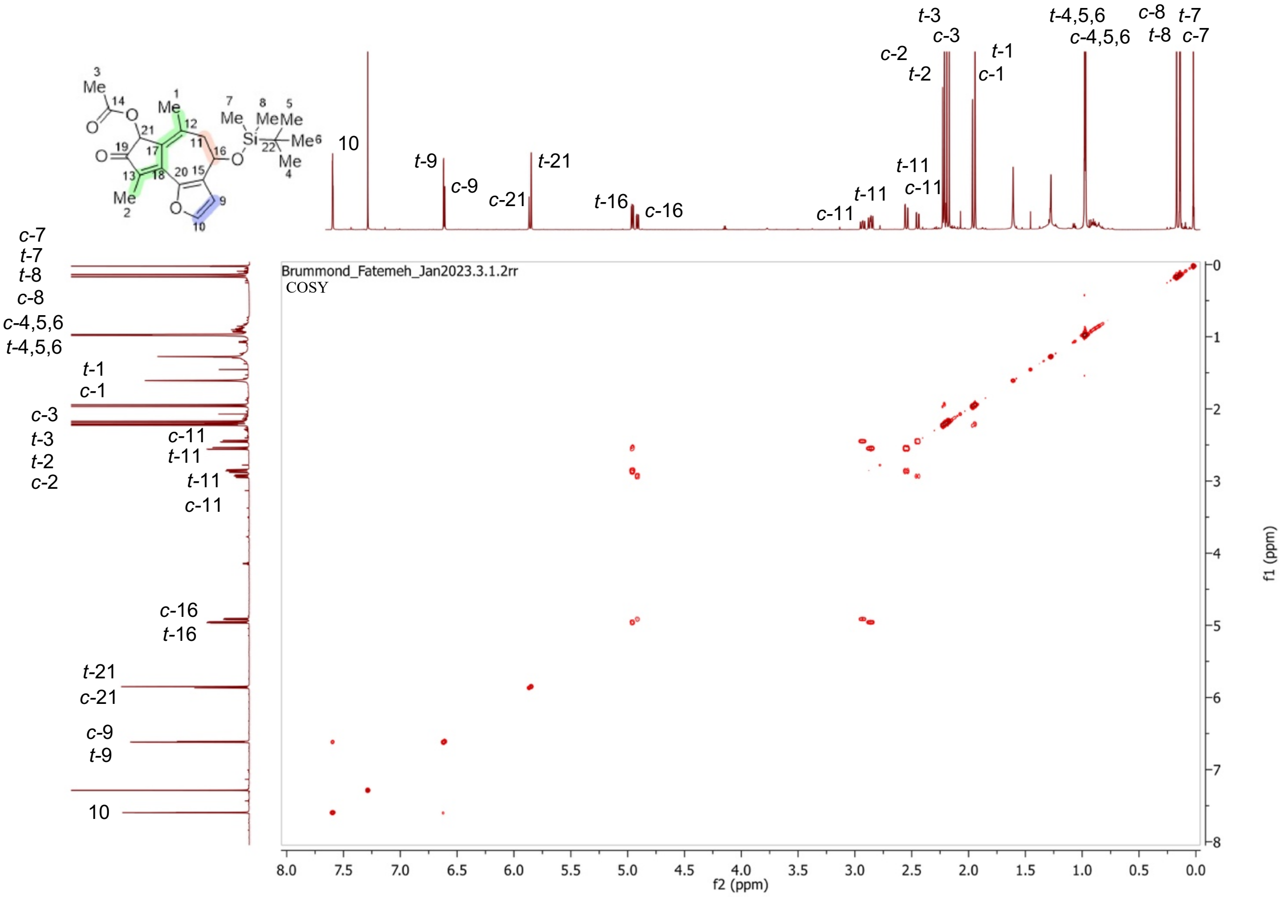

HMBC spectrum of **14a**

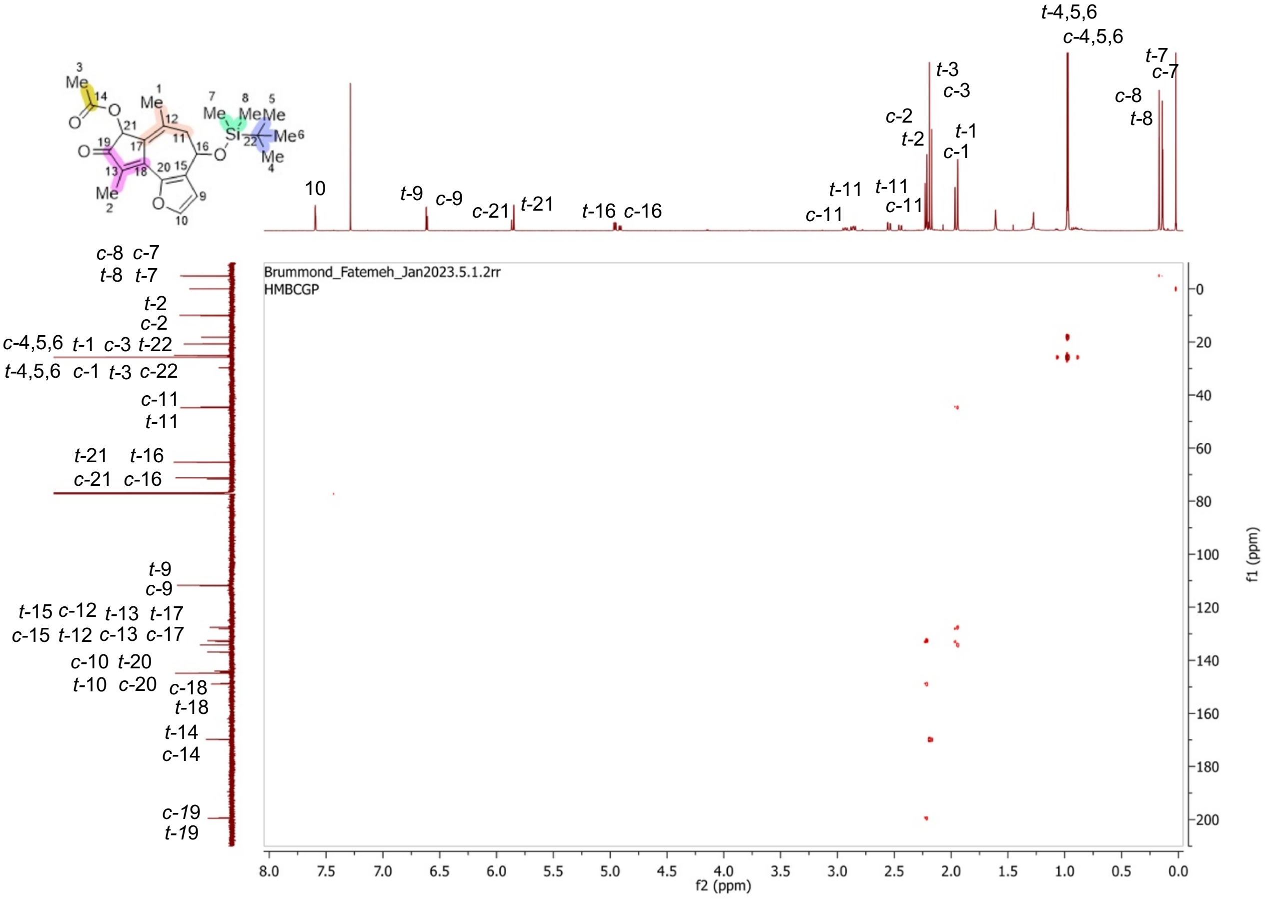

HMBC spectrum of **14a** (-5–45 ppm)

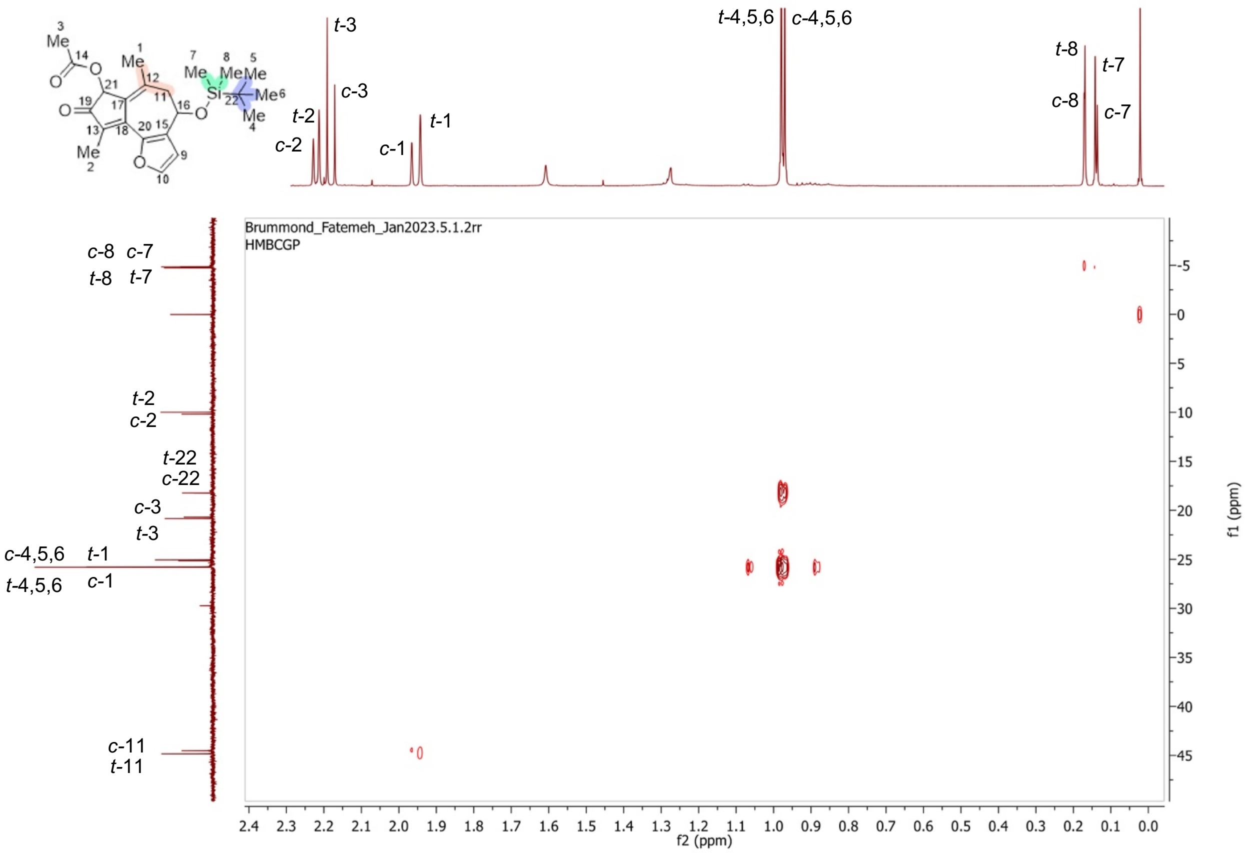

Selective HMBC spectrum of **14a** (142–147 ppm)

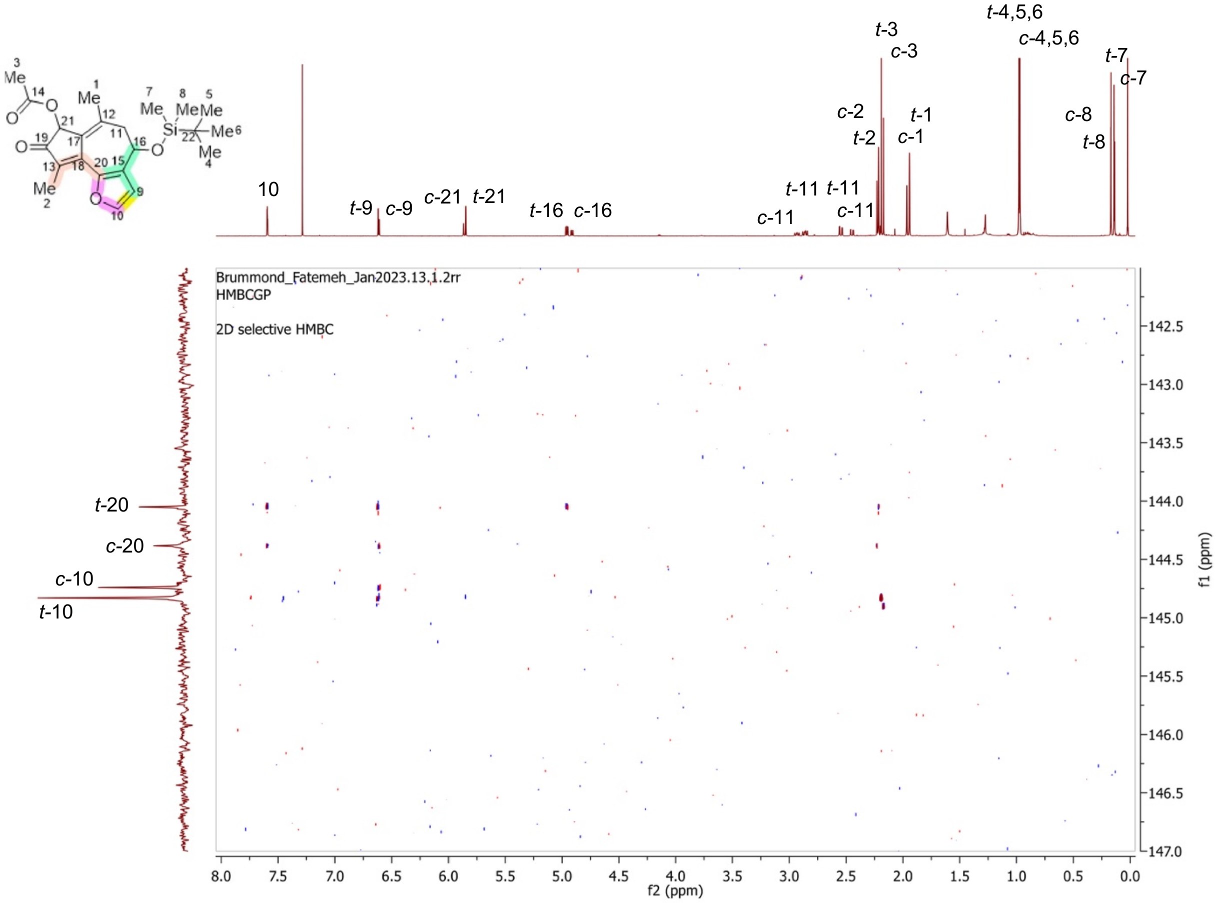

HSQC spectrum of **14a**

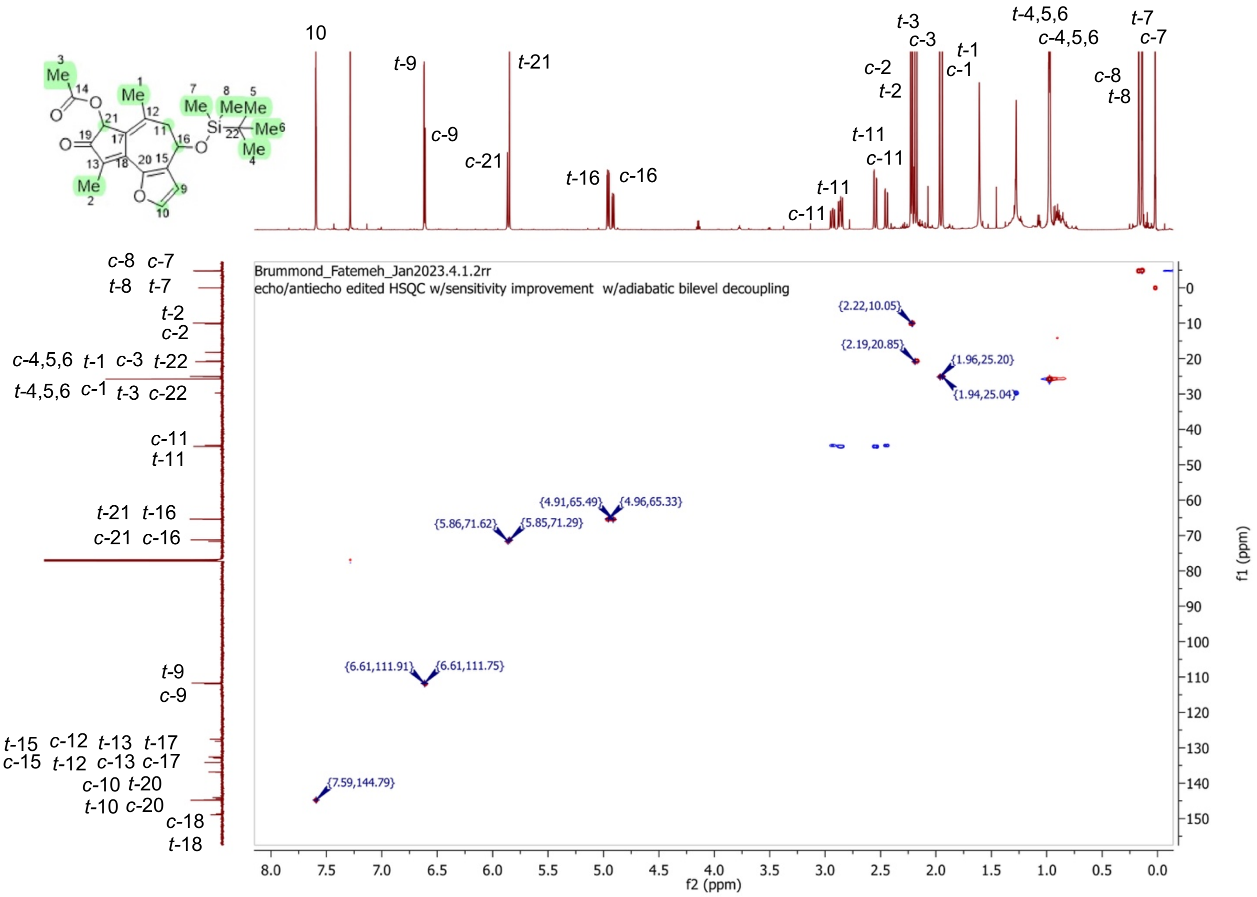

HSQC spectrum of **14a** (-5–40 ppm)

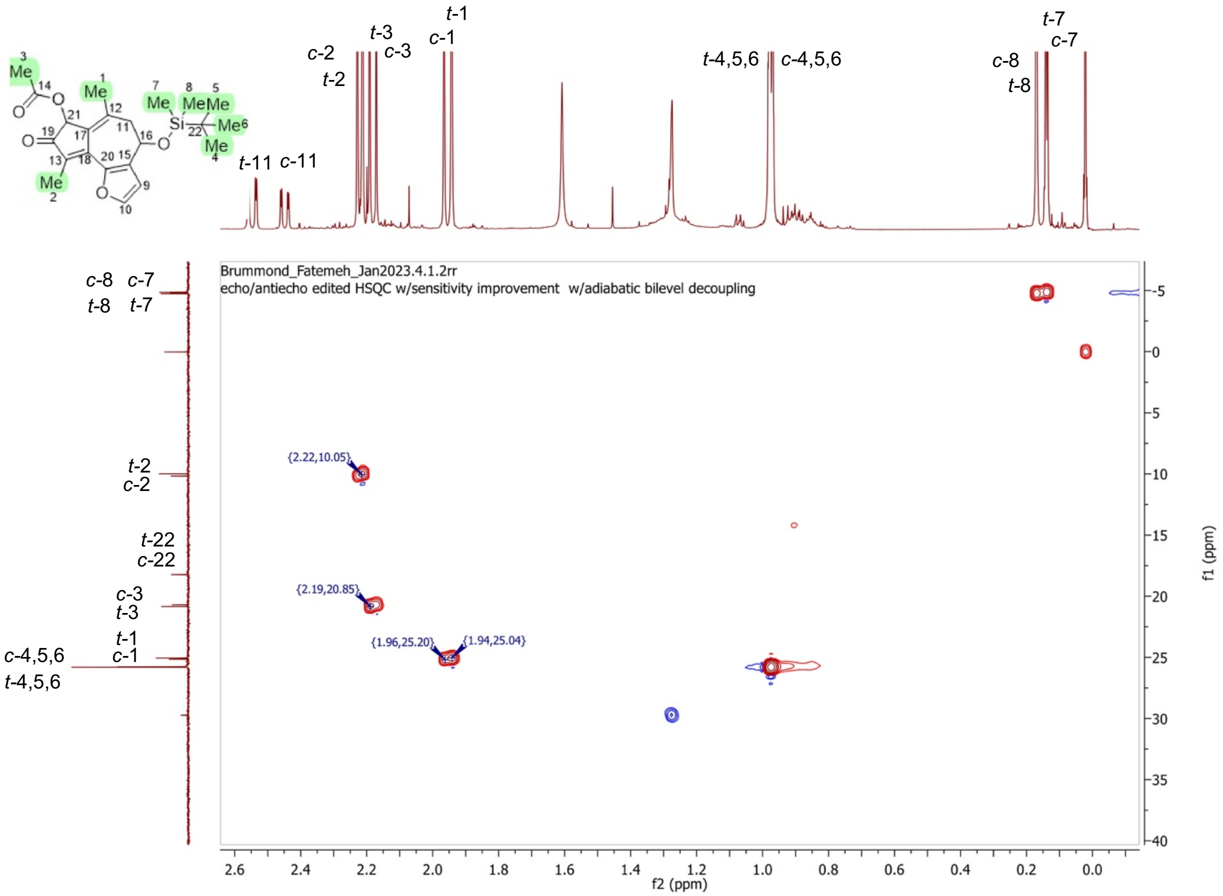

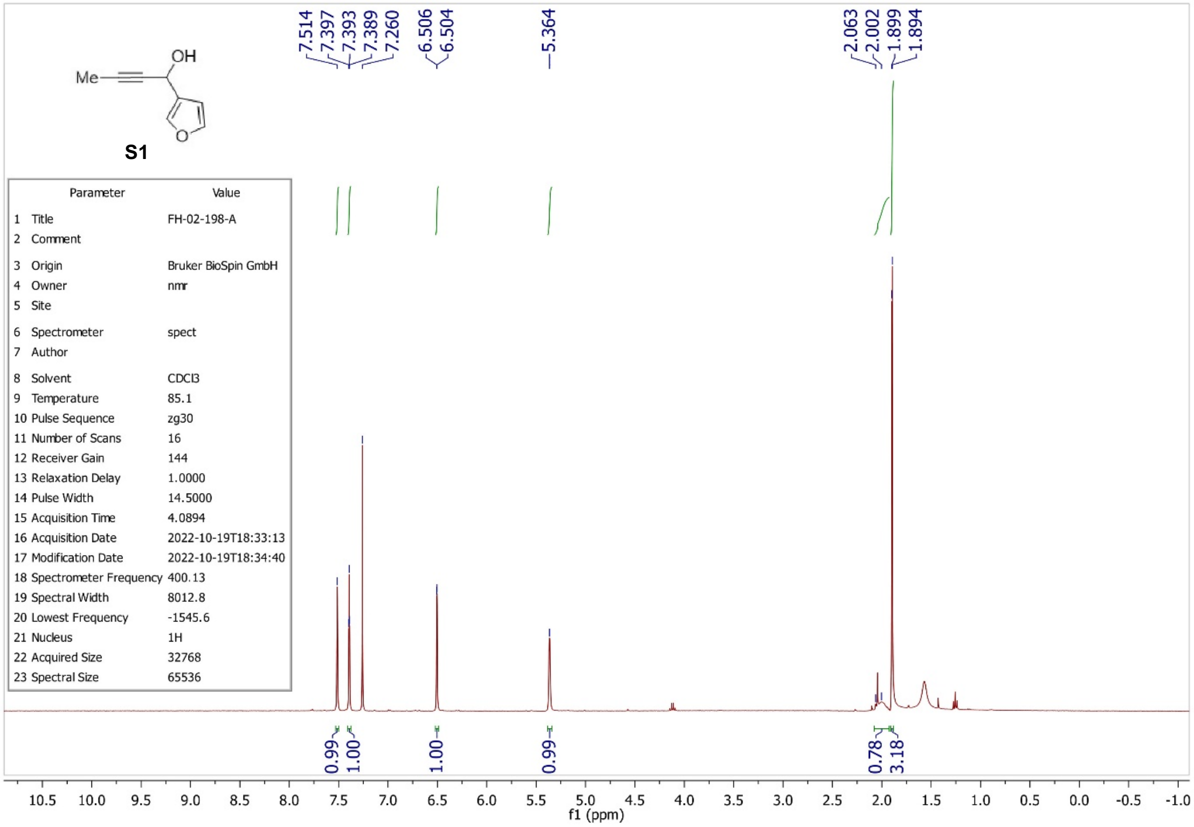

7.514
7.397
7.393
7.389
7.260
6.506
6.504
5.364
2.063
2.002
1.899
1.894
OH
Me
O
S1
Parameter Value
1 Title FH-02-198-A
2 Comment
3 Origin Bruker BioSpin GmbH
4 Owner nmr
5 Site
6 Spectrometer spect
7 Author
8 Solvent CDCl3
9 Temperature 85.1
10 Pulse Sequence zg30
11 Number of Scans 16
12 Receiver Gain 144
13 Relaxation Delay 1.0000
14 Pulse Width 14.5000
15 Acquisition Time 4.0894
16 Acquisition Date 2022-10-19T18:33:13
17 Modification Date 2022-10-19T18:34:40
18 Spectrometer Frequency 400.13
19 Spectral Width 8012.8
20 Lowest Frequency -1545.6
21 Nucleus 1H
22 Acquired Size 32768
23 Spectral Size 65536
0.99
1.00
1.00
0.99
0.78
3.18
10.5 10.0 9.5 9.0 8.5 8.0 7.5 7.0 6.5 6.0 5.5 5.0 4.5 4.0 3.5 3.0 2.5 2.0 1.5 1.0 0.5 0.0 -0.5 -1.0
f1 (ppm)

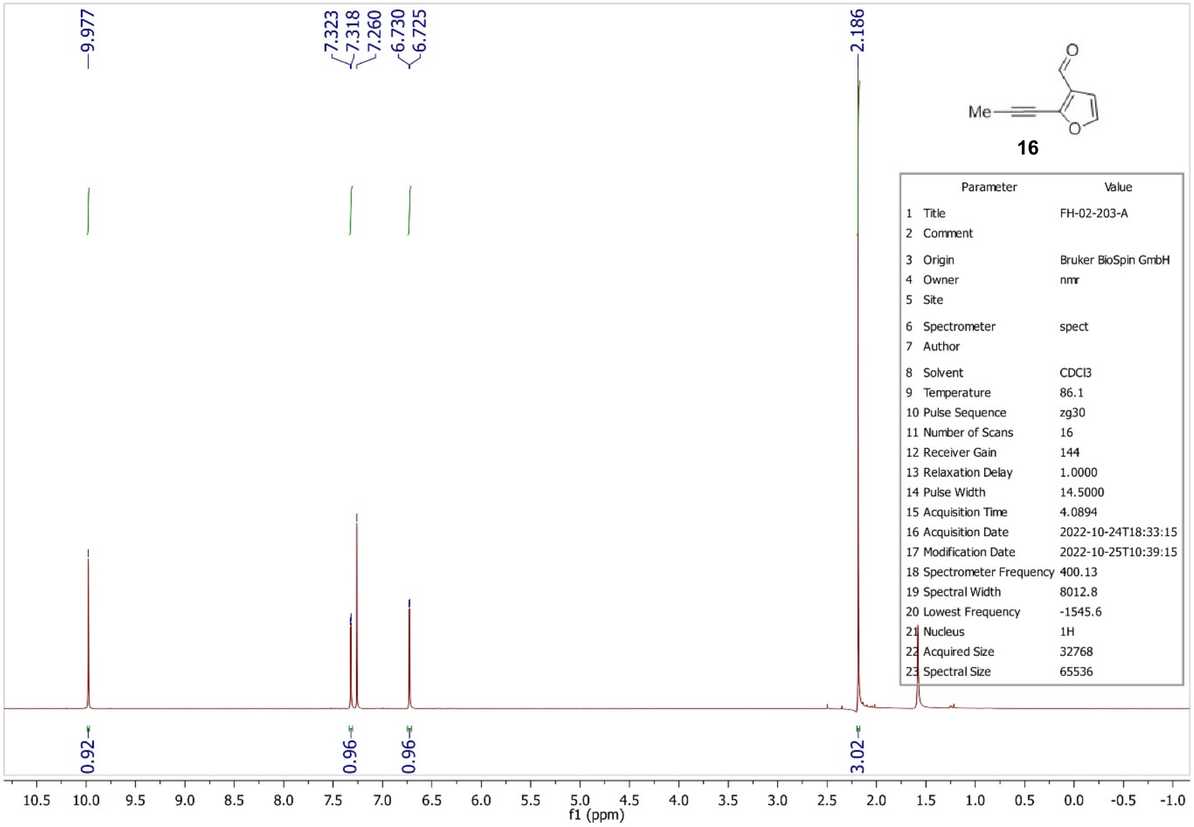

	Parameter	Value
1	Title	FH-02-203-A
2	Comment	
3	Origin	Bruker BioSpin GmbH
4	Owner	nmr
5	Site	
6	Spectrometer	spect
7	Author	
8	Solvent	CDCl3
9	Temperature	86.1
10	Pulse Sequence	zg30
11	Number of Scans	16
12	Receiver Gain	144
13	Relaxation Delay	1.0000
14	Pulse Width	14.5000
15	Acquisition Time	4.0894
16	Acquisition Date	2022-10-24T18:33:15
17	Modification Date	2022-10-25T10:39:15
18	Spectrometer Frequency	400.13
19	Spectral Width	8012.8
20	Lowest Frequency	-1545.6
21	Nucleus	1H
22	Acquired Size	32768
23	Spectral Size	65536

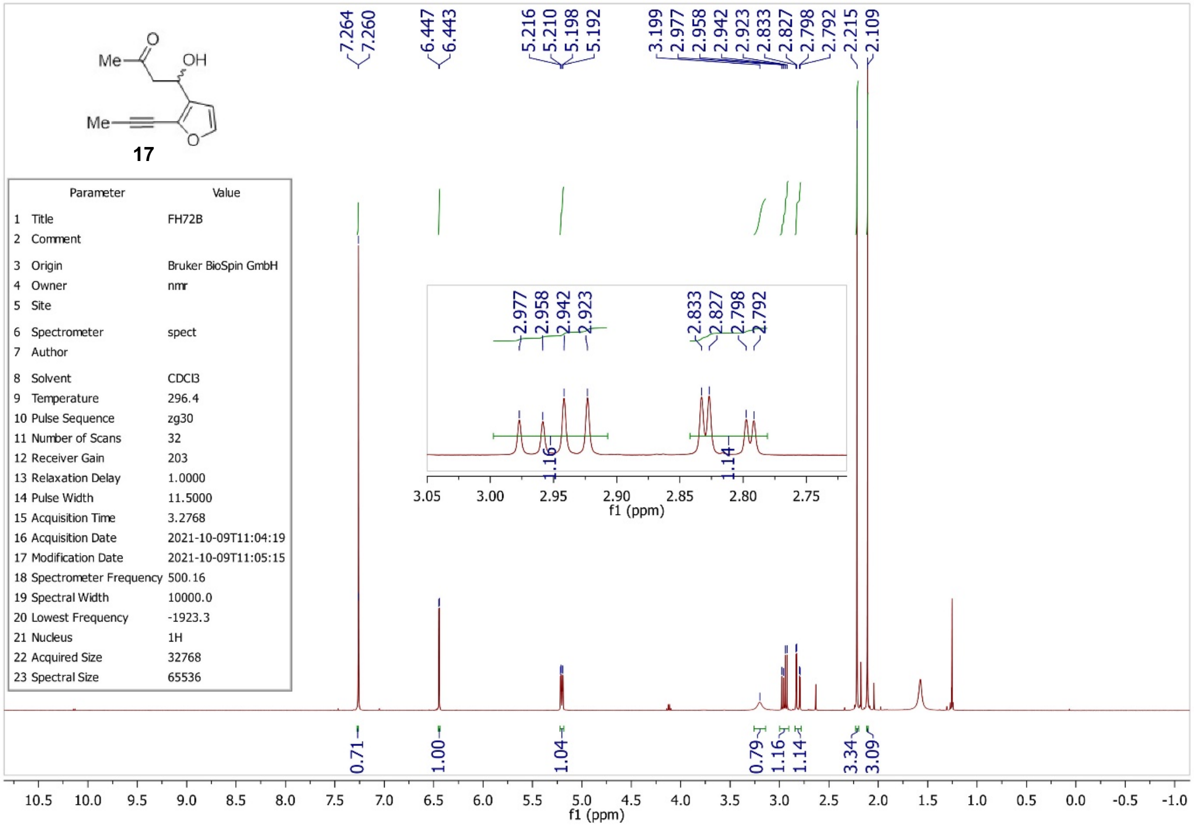
Me
O
OH
Me
O
17
Parameter Value
1 Title FH72B
2 Comment
3 Origin Bruker BioSpin GmbH
4 Owner nmr
5 Site
6 Spectrometer spect
7 Author
8 Solvent CDCl3
9 Temperature 296.4
10 Pulse Sequence zg30
11 Number of Scans 32
12 Receiver Gain 203
13 Relaxation Delay 1.0000
14 Pulse Width 11.5000
15 Acquisition Time 3.2768
16 Acquisition Date 2021-10-09T11:04:19
17 Modification Date 2021-10-09T11:05:15
18 Spectrometer Frequency 500.16
19 Spectral Width 10000.0
20 Lowest Frequency -1923.3
21 Nucleus 1H
22 Acquired Size 32768
23 Spectral Size 65536
7.264
7.260
6.447
6.443
5.216
5.210
5.198
5.192
3.199
2.977
2.958
2.942
2.923
2.833
2.827
2.798
2.792
2.215
2.109
2.977
2.958
2.942
2.923
2.833
2.827
2.798
2.792
1.16
1.14
3.05 3.00 2.95 2.90 2.85 2.80 2.75
f1 (ppm)
0.71
1.00
1.04
0.79
1.16
1.14
3.34
3.09
10.5 10.0 9.5 9.0 8.5 8.0 7.5 7.0 6.5 6.0 5.5 5.0 4.5 4.0 3.5 3.0 2.5 2.0 1.5 1.0 0.5 0.0 -0.5 -1.0
f1 (ppm)

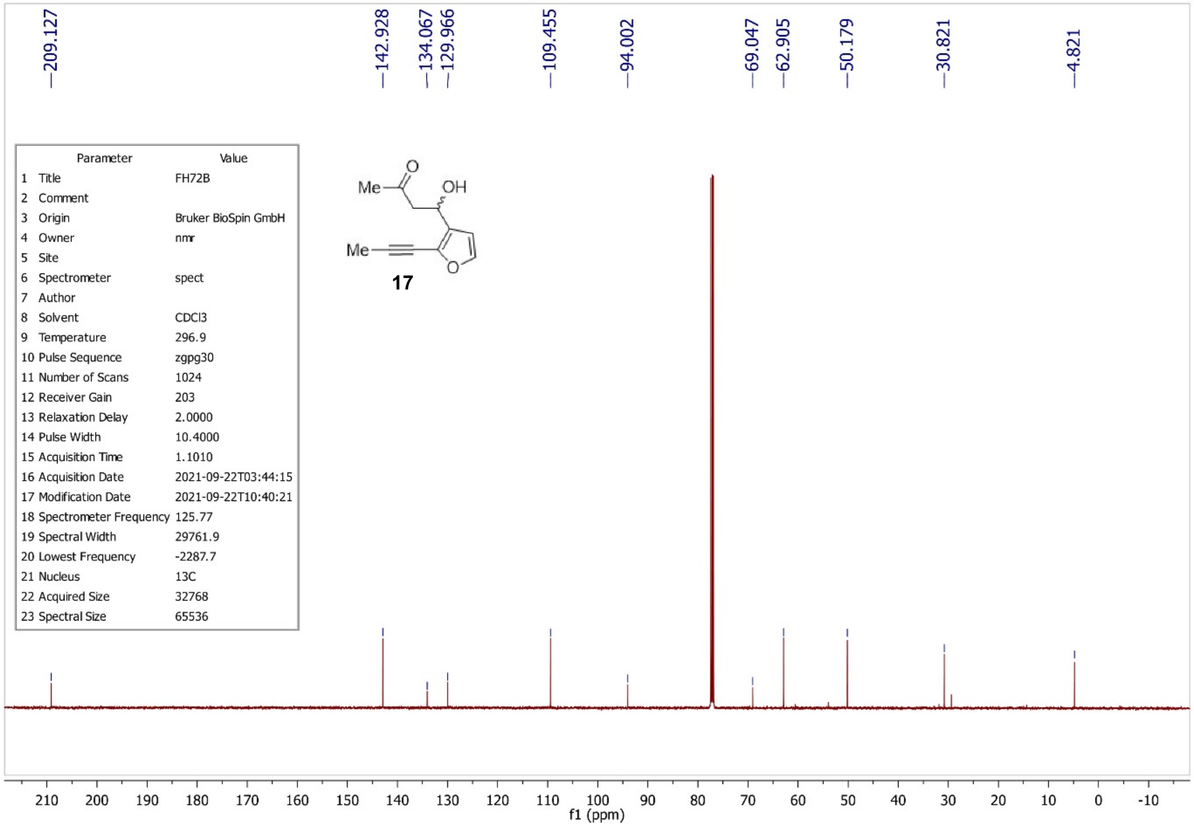
209.127
142.928
134.067
129.966
109.455
94.002
69.047
62.905
50.179
30.821
4.821
Parameter Value
1 Title FH72B
2 Comment
3 Origin Bruker BioSpin GmbH
4 Owner nmr
5 Site
6 Spectrometer spect
7 Author
8 Solvent CDCl3
9 Temperature 296.9
10 Pulse Sequence zgpg30
11 Number of Scans 1024
12 Receiver Gain 203
13 Relaxation Delay 2.0000
14 Pulse Width 10.4000
15 Acquisition Time 1.1010
16 Acquisition Date 2021-09-22T03:44:15
17 Modification Date 2021-09-22T10:40:21
18 Spectrometer Frequency 125.77
19 Spectral Width 29761.9
20 Lowest Frequency -2287.7
21 Nucleus 13C
22 Acquired Size 32768
23 Spectral Size 65536
Me
O
OH
Me
O
17
210 200 190 180 170 160 150 140 130 120 110 100 90 80 70 60 50 40 30 20 10 0 -10
f1 (ppm)

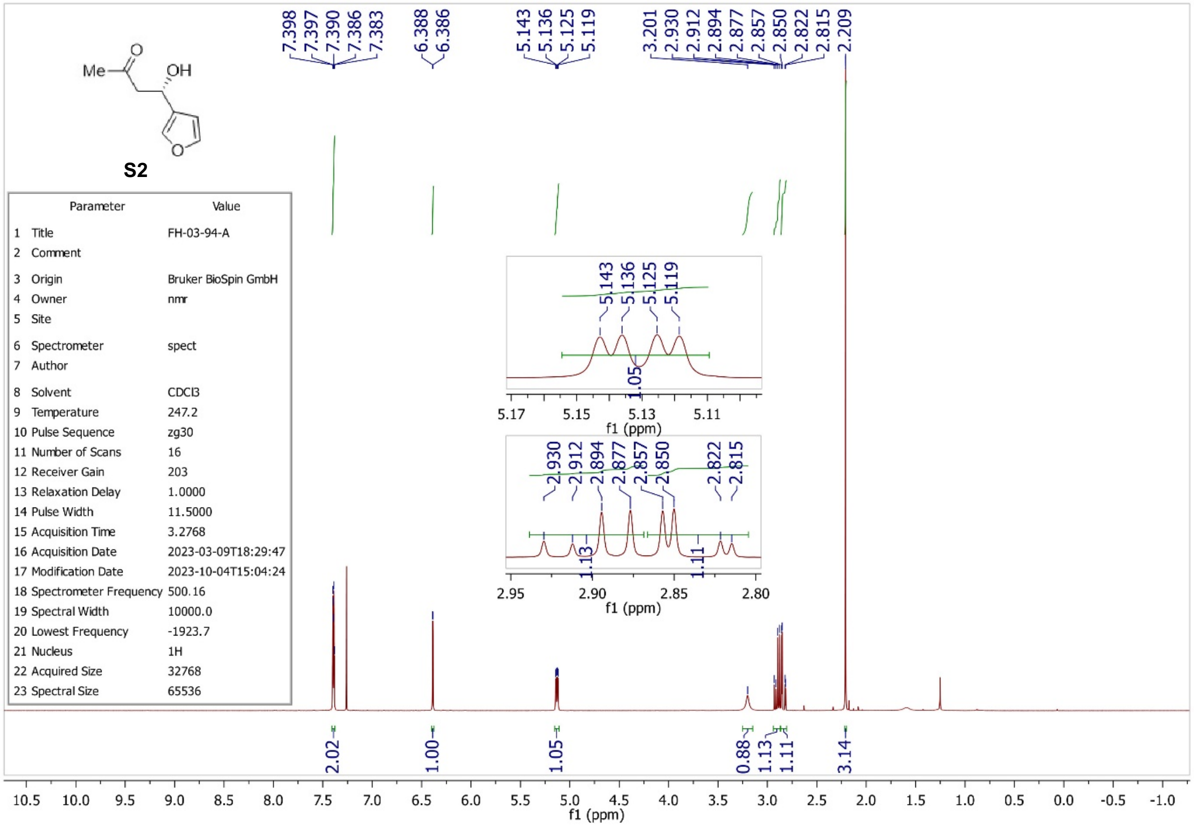

S2
Me
O
OH
O
Parameter	Value
1 Title	FH-03-94-A
2 Comment	
3 Origin	Bruker BioSpin GmbH
4 Owner	nmr
5 Site	
6 Spectrometer	spect
7 Author	
8 Solvent	CDCl3
9 Temperature	247.2
10 Pulse Sequence	zg30
11 Number of Scans	16
12 Receiver Gain	203
13 Relaxation Delay	1.0000
14 Pulse Width	11.5000
15 Acquisition Time	3.2768
16 Acquisition Date	2023-03-09T18:29:47
17 Modification Date	2023-10-04T15:04:24
18 Spectrometer Frequency	500.16
19 Spectral Width	10000.0
20 Lowest Frequency	-1923.7
21 Nucleus	1H
22 Acquired Size	32768
23 Spectral Size	65536
7.398 7.397 7.390 7.386 7.383
6.388 6.386
5.143 5.136 5.125 5.119
3.201 2.930 2.912 2.894 2.877 2.857 2.850 2.822 2.815
2.209
2.02 1.00 1.05 0.88 1.13 1.11 3.14
f1 (ppm)

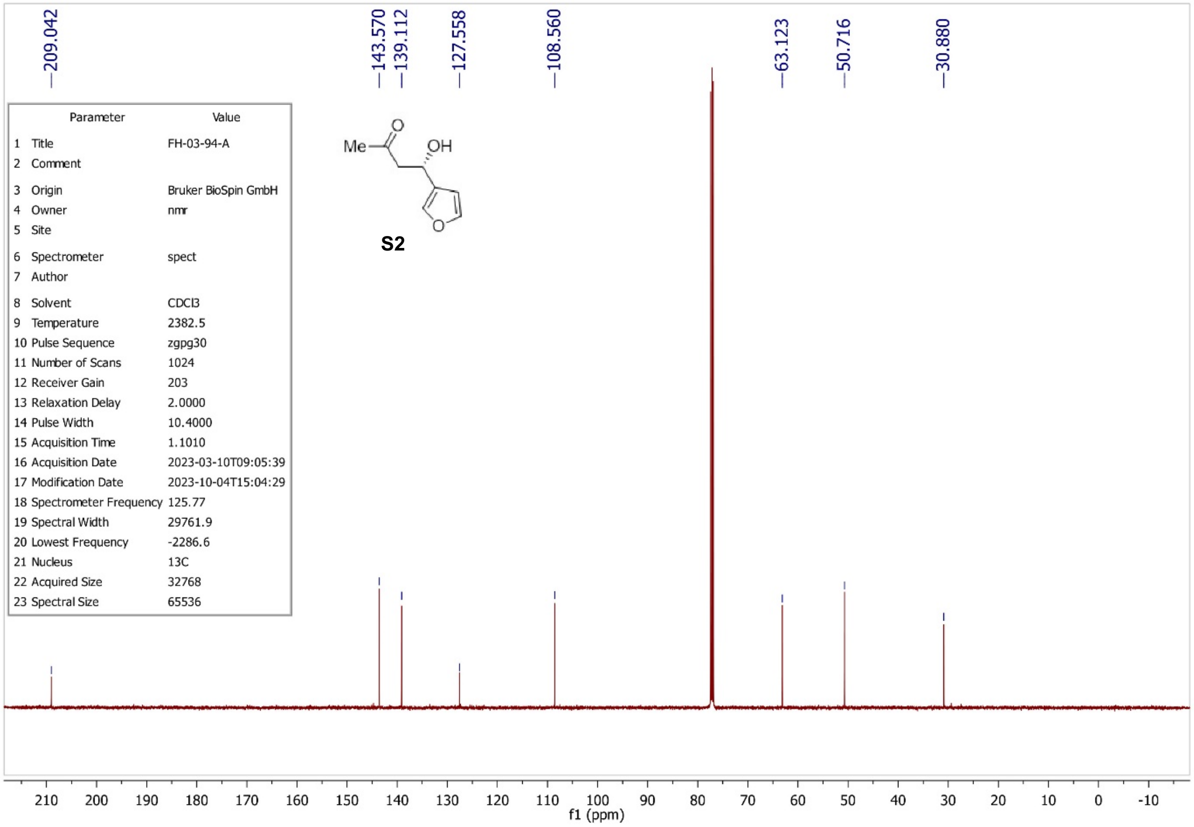
209.042
143.570
139.112
127.558
108.560
63.123
50.716
30.880
Parameter Value
1 Title FH-03-94-A
2 Comment
3 Origin Bruker BioSpin GmbH
4 Owner nmr
5 Site
6 Spectrometer spect
7 Author
8 Solvent CDCl3
9 Temperature 2382.5
10 Pulse Sequence zgpg30
11 Number of Scans 1024
12 Receiver Gain 203
13 Relaxation Delay 2.0000
14 Pulse Width 10.4000
15 Acquisition Time 1.1010
16 Acquisition Date 2023-03-10T09:05:39
17 Modification Date 2023-10-04T15:04:29
18 Spectrometer Frequency 125.77
19 Spectral Width 29761.9
20 Lowest Frequency -2286.6
21 Nucleus 13C
22 Acquired Size 32768
23 Spectral Size 65536
Me
O
OH
O
S2
210 200 190 180 170 160 150 140 130 120 110 100 90 80 70 60 50 40 30 20 10 0 -10
f1 (ppm)

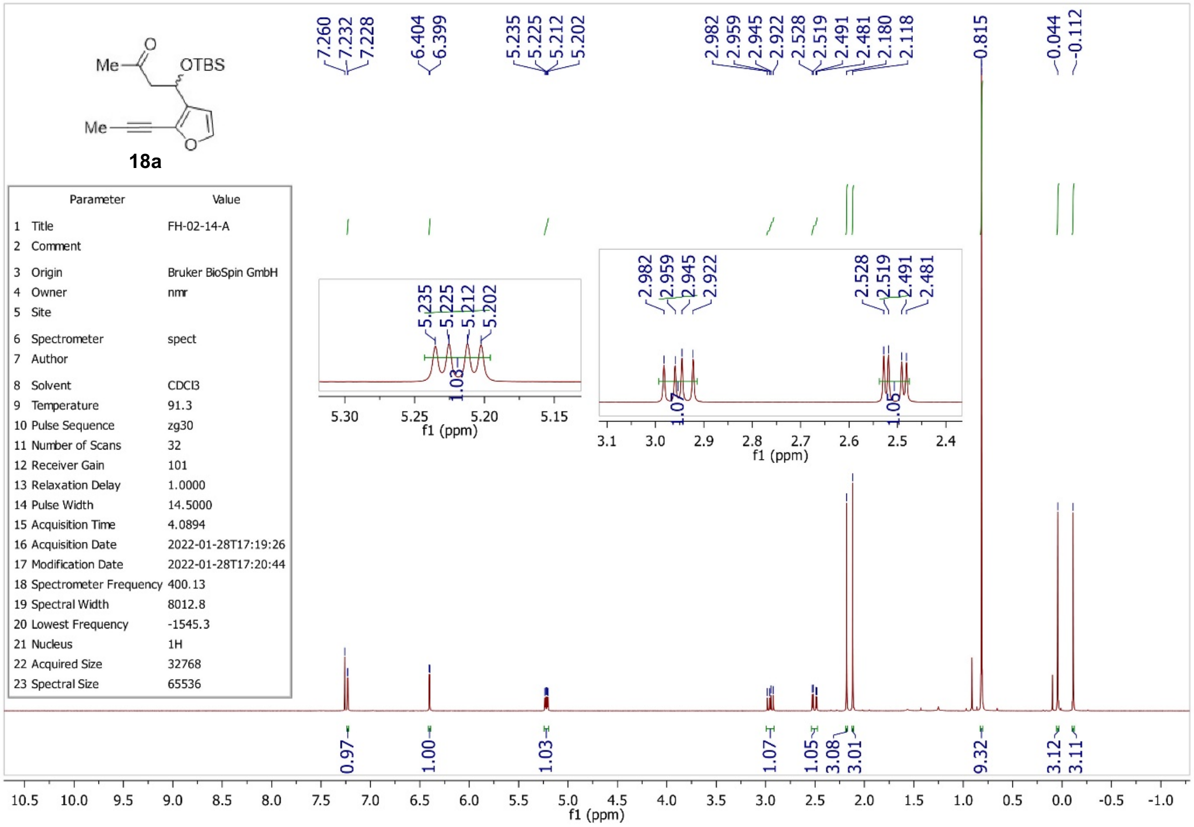

Me
O
OTBS
Me
O
18a
Parameter Value
1 Title FH-02-14-A
2 Comment
3 Origin Bruker BioSpin GmbH
4 Owner nmr
5 Site
6 Spectrometer spect
7 Author
8 Solvent CDCl3
9 Temperature 91.3
10 Pulse Sequence zg30
11 Number of Scans 32
12 Receiver Gain 101
13 Relaxation Delay 1.0000
14 Pulse Width 14.5000
15 Acquisition Time 4.0894
16 Acquisition Date 2022-01-28T17:19:26
17 Modification Date 2022-01-28T17:20:44
18 Spectrometer Frequency 400.13
19 Spectral Width 8012.8
20 Lowest Frequency -1545.3
21 Nucleus 1H
22 Acquired Size 32768
23 Spectral Size 65536
7.260 7.232 7.228 6.404 6.399 5.235 5.225 5.212 5.202 2.982 2.959 2.945 2.922 2.528 2.519 2.491 2.481 2.180 2.118 0.815 0.044 -0.112
0.97 1.00 1.03 1.07 1.05 3.08 3.01 9.32 3.12 3.11
f1 (ppm)

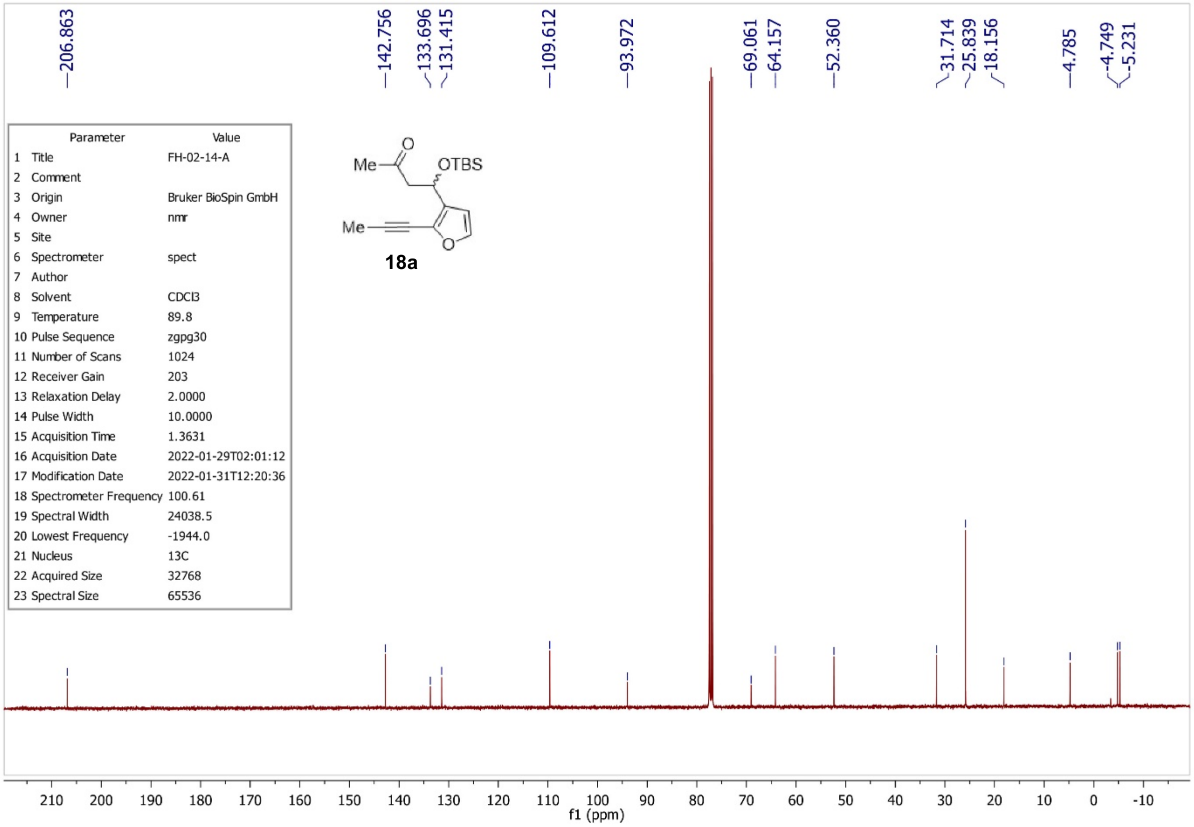
206.863
142.756
133.696
131.415
109.612
93.972
69.061
64.157
52.360
31.714
25.839
18.156
4.785
-4.749
-5.231
Parameter Value
1 Title FH-02-14-A
2 Comment
3 Origin Bruker BioSpin GmbH
4 Owner nmr
5 Site
6 Spectrometer spect
7 Author
8 Solvent CDCl3
9 Temperature 89.8
10 Pulse Sequence zgpg30
11 Number of Scans 1024
12 Receiver Gain 203
13 Relaxation Delay 2.0000
14 Pulse Width 10.0000
15 Acquisition Time 1.3631
16 Acquisition Date 2022-01-29T02:01:12
17 Modification Date 2022-01-31T12:20:36
18 Spectrometer Frequency 100.61
19 Spectral Width 24038.5
20 Lowest Frequency -1944.0
21 Nucleus 13C
22 Acquired Size 32768
23 Spectral Size 65536
Me
O
OTBS
Me
O
18a
f1 (ppm)

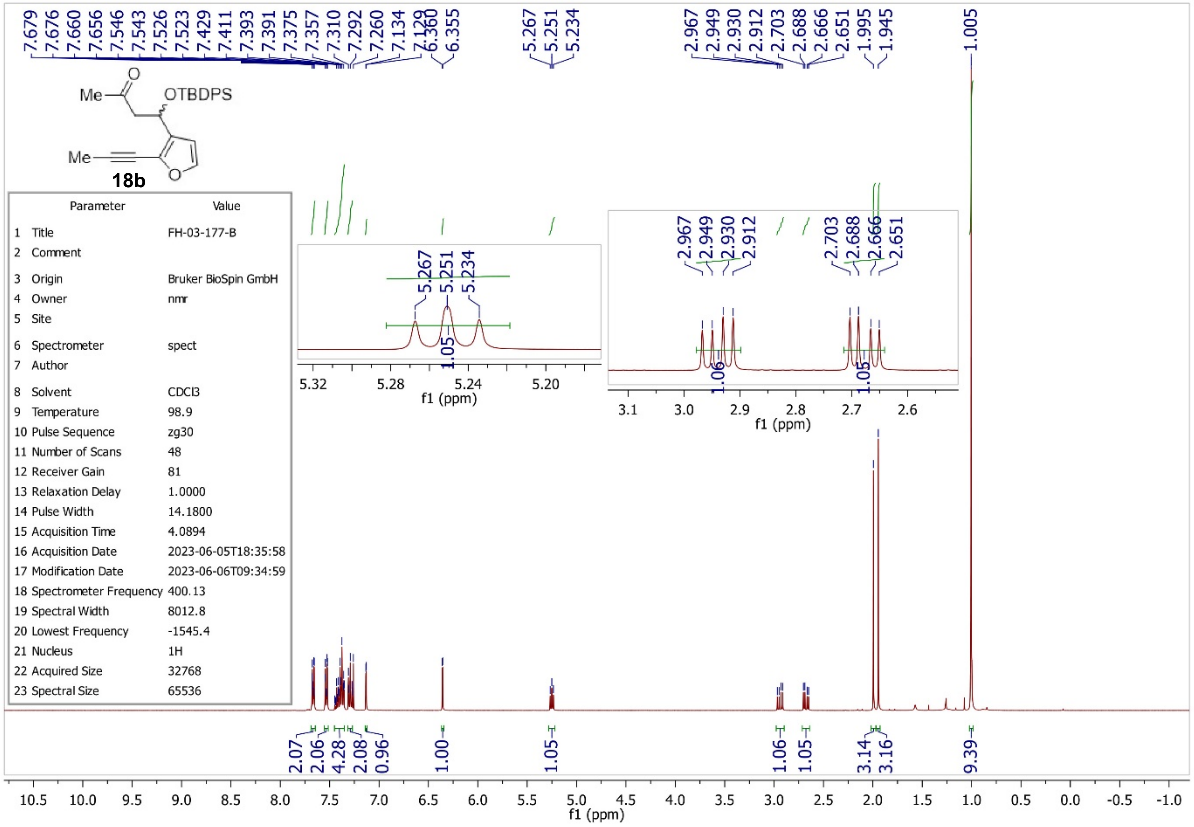

Parameter	Value
1 Title	FH-03-177-B
2 Comment	
3 Origin	Bruker BioSpin GmbH
4 Owner	nmr
5 Site	
6 Spectrometer	spect
7 Author	
8 Solvent	CDCl3
9 Temperature	98.9
10 Pulse Sequence	zg30
11 Number of Scans	48
12 Receiver Gain	81
13 Relaxation Delay	1.0000
14 Pulse Width	14.1800
15 Acquisition Time	4.0894
16 Acquisition Date	2023-06-05T18:35:58
17 Modification Date	2023-06-06T09:34:59
18 Spectrometer Frequency	400.13
19 Spectral Width	8012.8
20 Lowest Frequency	-1545.4
21 Nucleus	1H
22 Acquired Size	32768
23 Spectral Size	65536

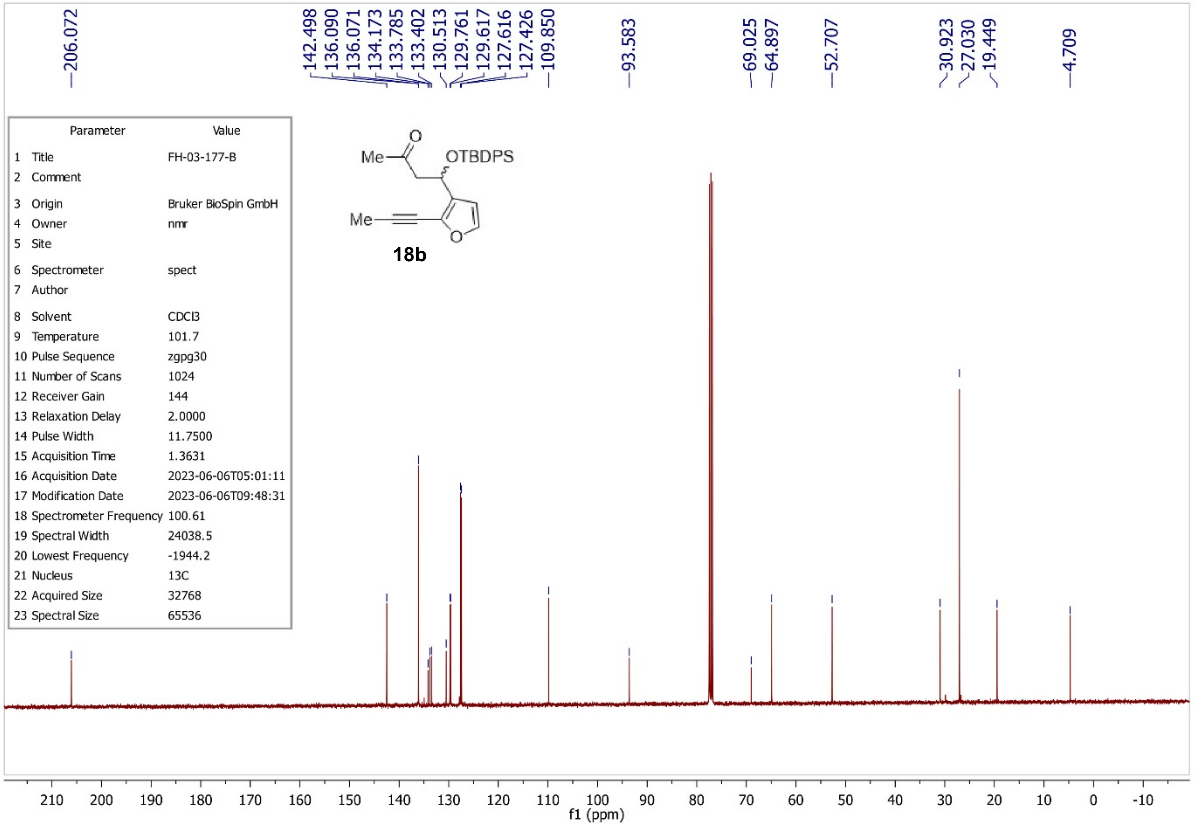
206.072
142.498
136.090
136.071
134.173
133.785
133.402
130.513
129.761
129.617
127.616
127.426
109.850
93.583
69.025
64.897
52.707
30.923
27.030
19.449
4.709
Parameter
Value
1 Title
FH-03-177-B
2 Comment
3 Origin
Bruker BioSpin GmbH
4 Owner
nmr
5 Site
6 Spectrometer
spect
7 Author
8 Solvent
CDCl3
9 Temperature
101.7
10 Pulse Sequence
zgpg30
11 Number of Scans
1024
12 Receiver Gain
144
13 Relaxation Delay
2.0000
14 Pulse Width
11.7500
15 Acquisition Time
1.3631
16 Acquisition Date
2023-06-06T05:01:11
17 Modification Date
2023-06-06T09:48:31
18 Spectrometer Frequency
100.61
19 Spectral Width
24038.5
20 Lowest Frequency
-1944.2
21 Nucleus
13C
22 Acquired Size
32768
23 Spectral Size
65536
Me
O
OTBDPS
Me
O
18b
f1 (ppm)

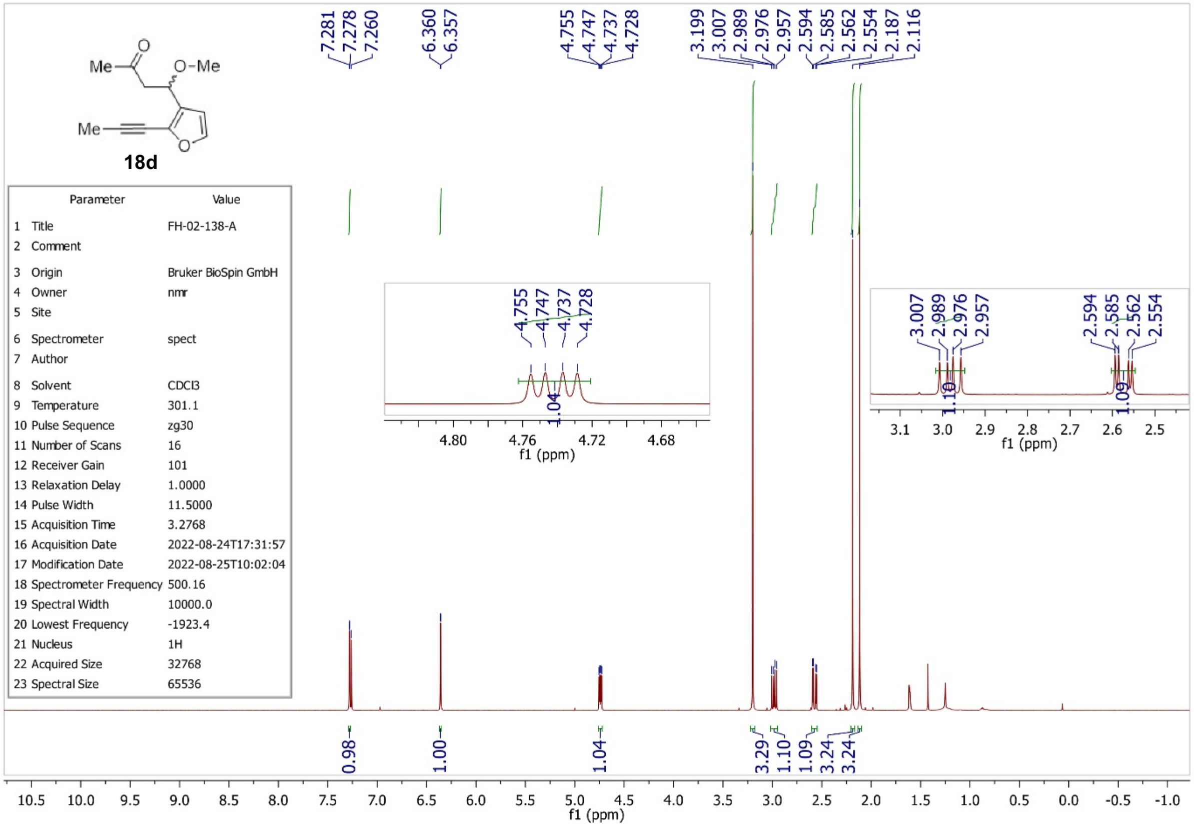

18d
Me
O
O–Me
Me
O
7.281
7.278
7.260
6.360
6.357
4.755
4.747
4.737
4.728
3.199
3.007
2.989
2.976
2.957
2.594
2.585
2.562
2.554
2.187
2.116
Parameter Value
1 Title FH-02-138-A
2 Comment
3 Origin Bruker BioSpin GmbH
4 Owner nmr
5 Site
6 Spectrometer spect
7 Author
8 Solvent CDCl3
9 Temperature 301.1
10 Pulse Sequence zg30
11 Number of Scans 16
12 Receiver Gain 101
13 Relaxation Delay 1.0000
14 Pulse Width 11.5000
15 Acquisition Time 3.2768
16 Acquisition Date 2022-08-24T17:31:57
17 Modification Date 2022-08-25T10:02:04
18 Spectrometer Frequency 500.16
19 Spectral Width 10000.0
20 Lowest Frequency -1923.4
21 Nucleus 1H
22 Acquired Size 32768
23 Spectral Size 65536
0.98
1.00
1.04
3.29
1.10
1.09
3.24
3.24
f1 (ppm)

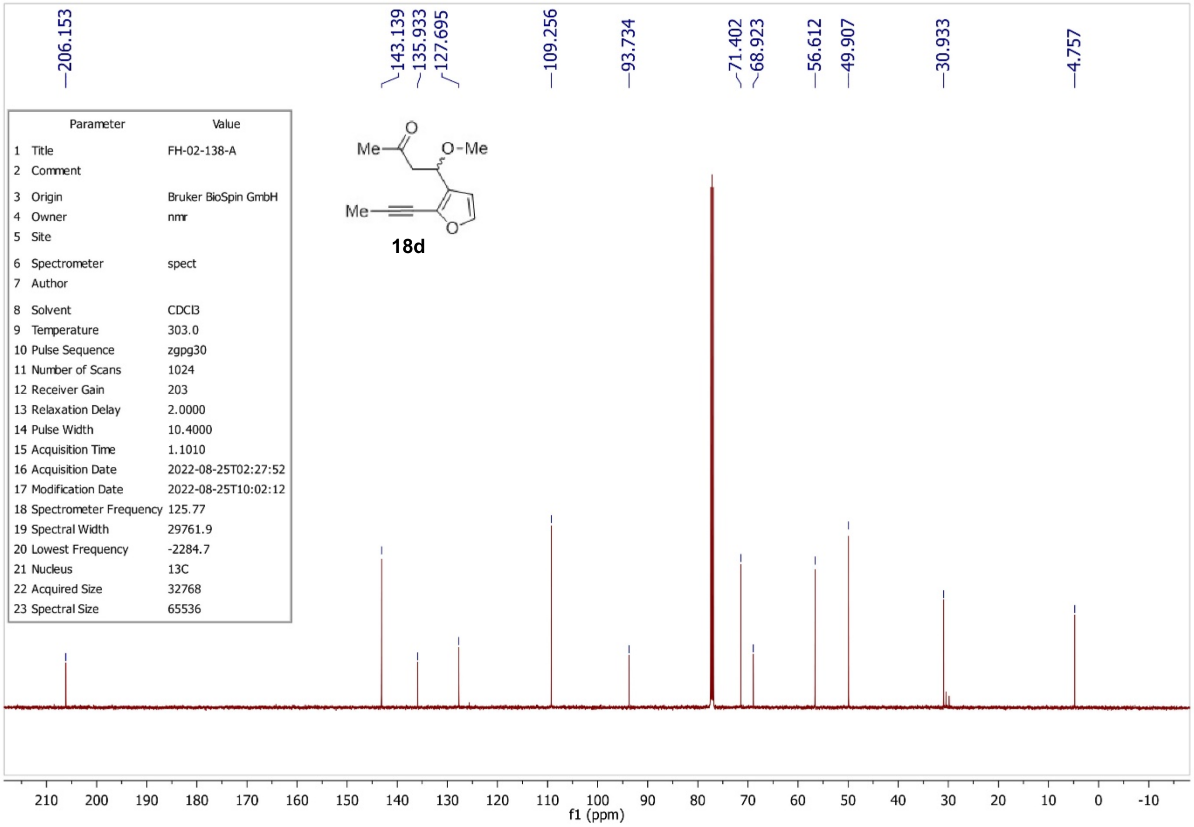
206.153
143.139
135.933
127.695
109.256
93.734
71.402
68.923
56.612
49.907
30.933
4.757
Parameter Value
1 Title FH-02-138-A
2 Comment
3 Origin Bruker BioSpin GmbH
4 Owner nmr
5 Site
6 Spectrometer spect
7 Author
8 Solvent CDCl3
9 Temperature 303.0
10 Pulse Sequence zgpg30
11 Number of Scans 1024
12 Receiver Gain 203
13 Relaxation Delay 2.0000
14 Pulse Width 10.4000
15 Acquisition Time 1.1010
16 Acquisition Date 2022-08-25T02:27:52
17 Modification Date 2022-08-25T10:02:12
18 Spectrometer Frequency 125.77
19 Spectral Width 29761.9
20 Lowest Frequency -2284.7
21 Nucleus 13C
22 Acquired Size 32768
23 Spectral Size 65536
Me
O
O–Me
Me
O
18d
210 200 190 180 170 160 150 140 130 120 110 100 90 80 70 60 50 40 30 20 10 0 -10
f1 (ppm)

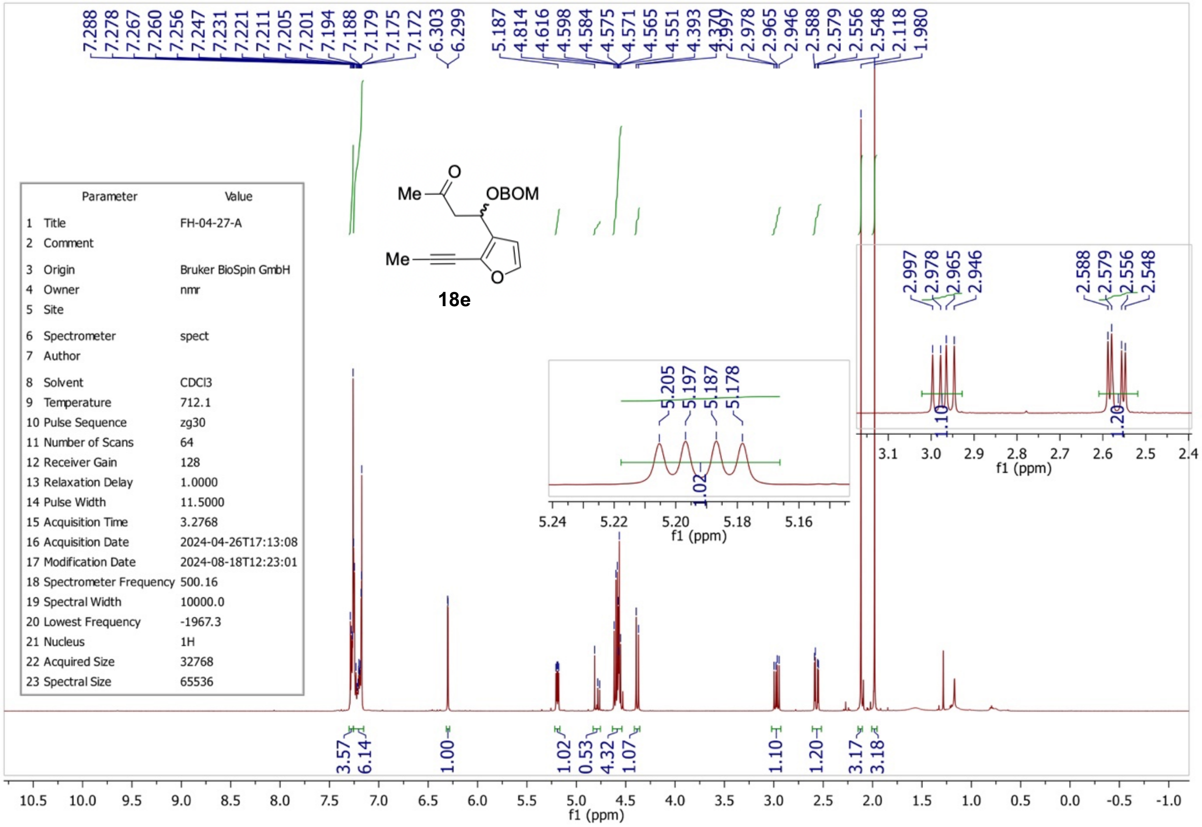

Parameter Value
1 Title FH-04-27-A
2 Comment
3 Origin Bruker BioSpin GmbH
4 Owner nmr
5 Site
6 Spectrometer spect
7 Author
8 Solvent CDCl3
9 Temperature 712.1
10 Pulse Sequence zg30
11 Number of Scans 64
12 Receiver Gain 128
13 Relaxation Delay 1.0000
14 Pulse Width 11.5000
15 Acquisition Time 3.2768
16 Acquisition Date 2024-04-26T17:13:08
17 Modification Date 2024-08-18T12:23:01
18 Spectrometer Frequency 500.16
19 Spectral Width 10000.0
20 Lowest Frequency -1967.3
21 Nucleus 1H
22 Acquired Size 32768
23 Spectral Size 65536
Me
O
OBOM
Me
O
18e
f1 (ppm)

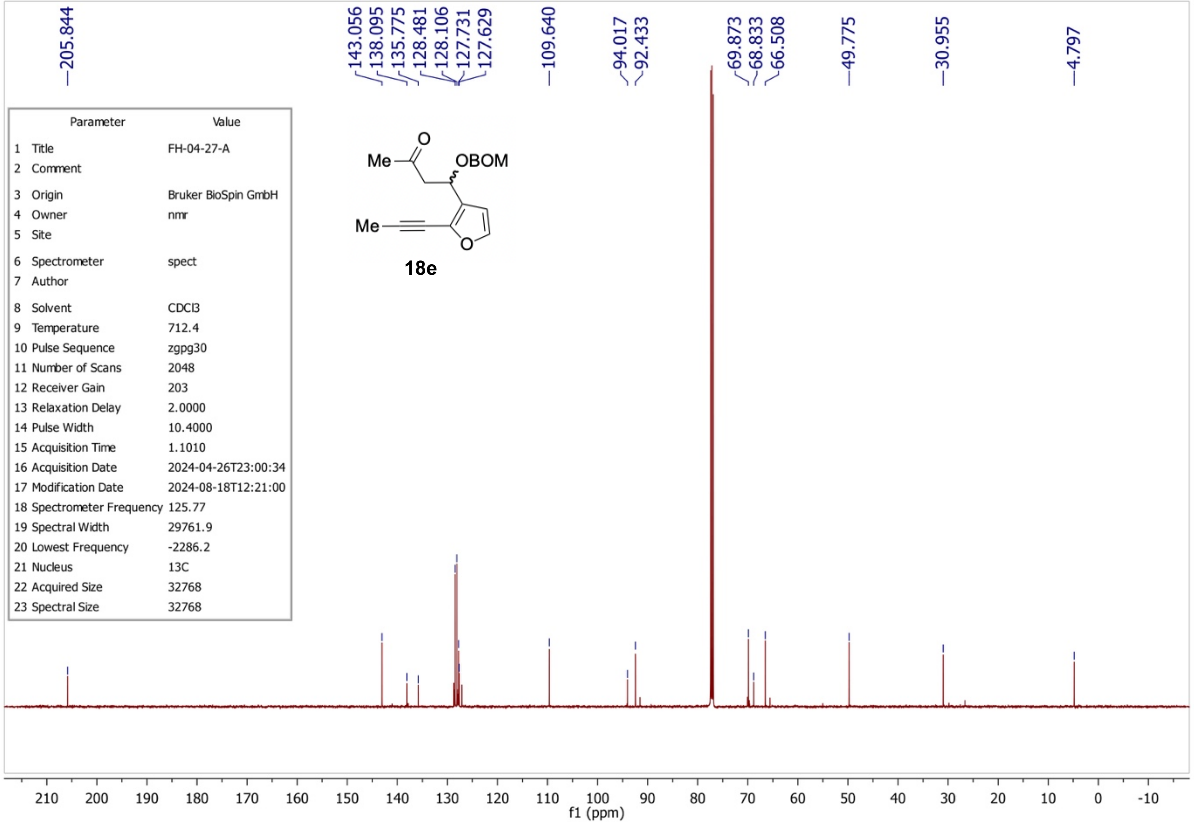
205.844
143.056
138.095
135.775
128.481
128.106
127.731
127.629
109.640
94.017
92.433
69.873
68.833
66.508
49.775
30.955
4.797
Parameter Value
1 Title FH-04-27-A
2 Comment
3 Origin Bruker BioSpin GmbH
4 Owner nmr
5 Site
6 Spectrometer spect
7 Author
8 Solvent CDCl3
9 Temperature 712.4
10 Pulse Sequence zgpg30
11 Number of Scans 2048
12 Receiver Gain 203
13 Relaxation Delay 2.0000
14 Pulse Width 10.4000
15 Acquisition Time 1.1010
16 Acquisition Date 2024-04-26T23:00:34
17 Modification Date 2024-08-18T12:21:00
18 Spectrometer Frequency 125.77
19 Spectral Width 29761.9
20 Lowest Frequency -2286.2
21 Nucleus 13C
22 Acquired Size 32768
23 Spectral Size 32768
Me
O
OBOM
Me
O
18e
210 200 190 180 170 160 150 140 130 120 110 100 90 80 70 60 50 40 30 20 10 0 -10
f1 (ppm)

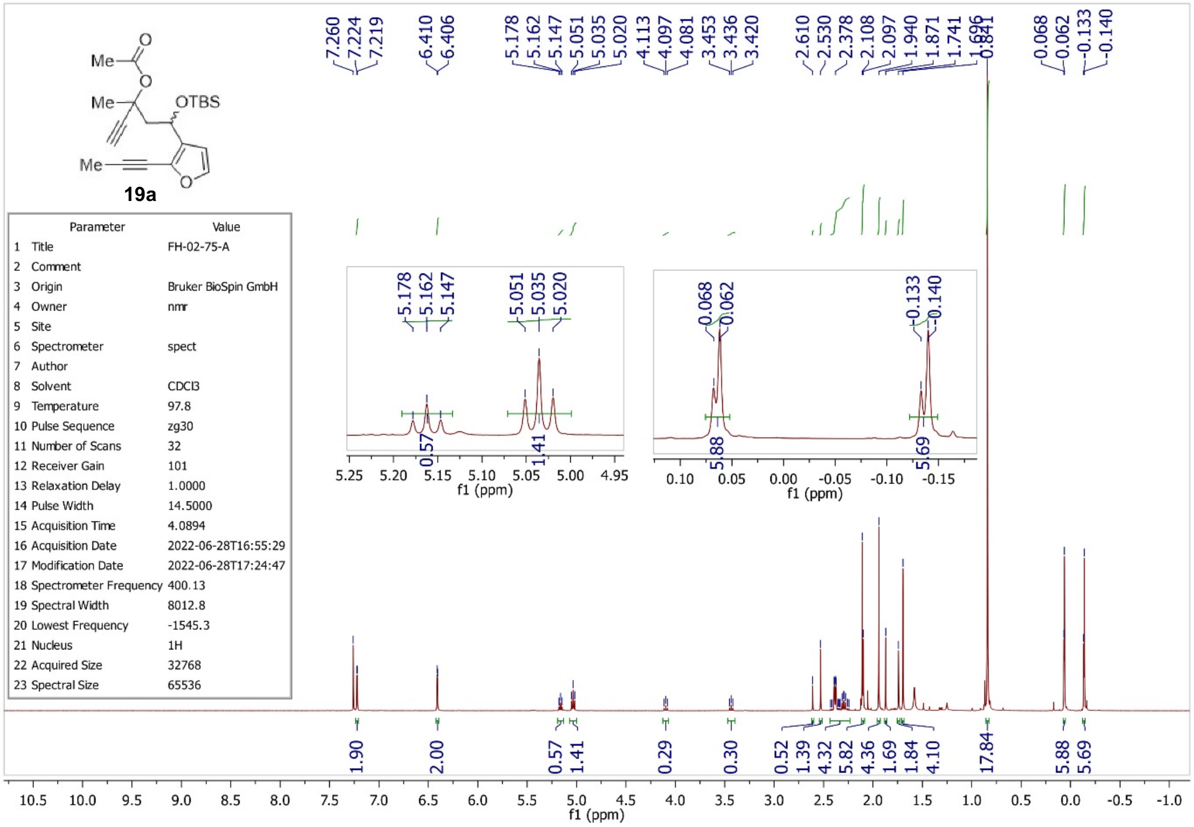

Parameter		Value
1	Title	FH-02-75-A
2	Comment	
3	Origin	Bruker BioSpin GmbH
4	Owner	nmr
5	Site	
6	Spectrometer	spect
7	Author	
8	Solvent	CDCl3
9	Temperature	97.8
10	Pulse Sequence	zg30
11	Number of Scans	32
12	Receiver Gain	101
13	Relaxation Delay	1.0000
14	Pulse Width	14.5000
15	Acquisition Time	4.0894
16	Acquisition Date	2022-06-28T16:55:29
17	Modification Date	2022-06-28T17:24:47
18	Spectrometer Frequency	400.13
19	Spectral Width	8012.8
20	Lowest Frequency	-1545.3
21	Nucleus	1H
22	Acquired Size	32768
23	Spectral Size	65536

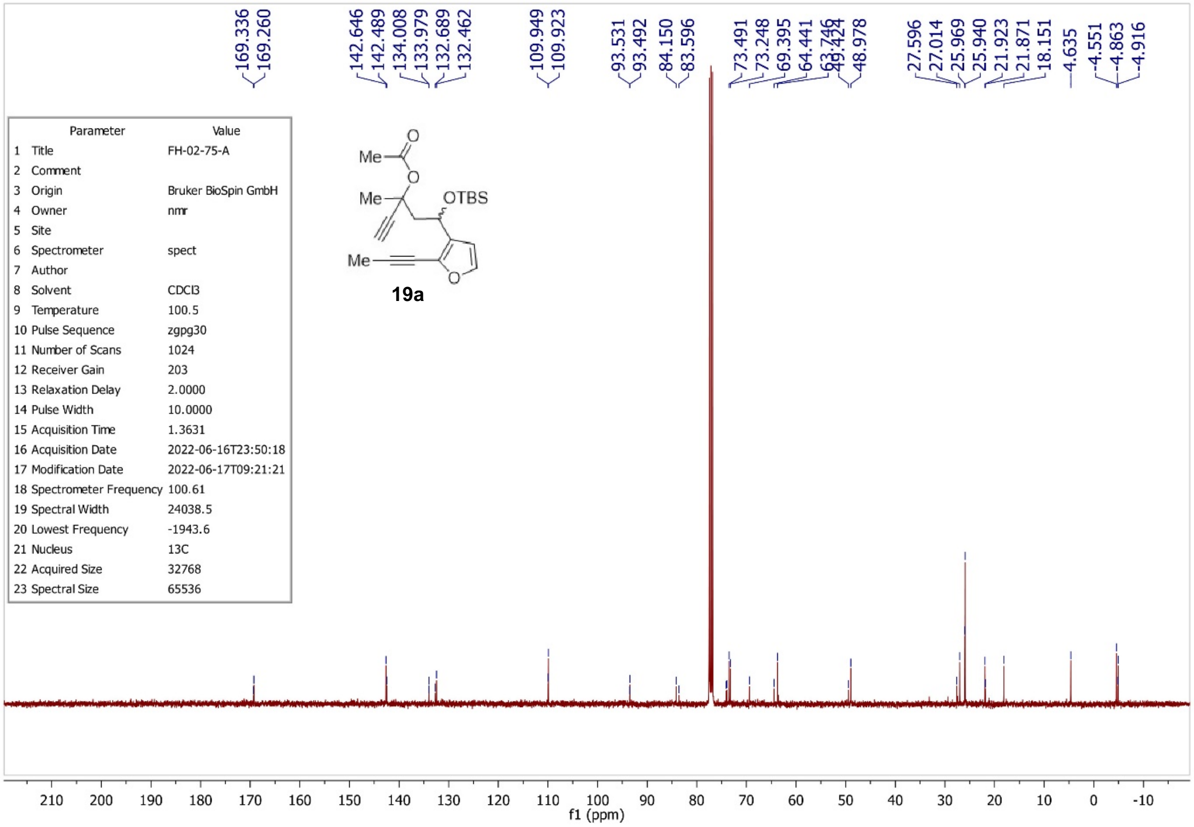
169.336
169.260
142.646
142.489
134.008
133.979
132.689
132.462
109.949
109.923
93.531
93.492
84.150
83.596
73.491
73.248
69.395
64.441
48.978
27.596
27.014
25.969
25.940
21.923
21.871
18.151
4.635
-4.551
-4.863
-4.916
Parameter
Value
1 Title
FH-02-75-A
2 Comment
3 Origin
Bruker BioSpin GmbH
4 Owner
nmr
5 Site
6 Spectrometer
spect
7 Author
8 Solvent
CDCl3
9 Temperature
100.5
10 Pulse Sequence
zgpg30
11 Number of Scans
1024
12 Receiver Gain
203
13 Relaxation Delay
2.0000
14 Pulse Width
10.0000
15 Acquisition Time
1.3631
16 Acquisition Date
2022-06-16T23:50:18
17 Modification Date
2022-06-17T09:21:21
18 Spectrometer Frequency
100.61
19 Spectral Width
24038.5
20 Lowest Frequency
-1943.6
21 Nucleus
13C
22 Acquired Size
32768
23 Spectral Size
65536
Me
O
O
Me
OTBS
Me
O
19a
210 200 190 180 170 160 150 140 130 120 110 100 90 80 70 60 50 40 30 20 10 0 -10
f1 (ppm)

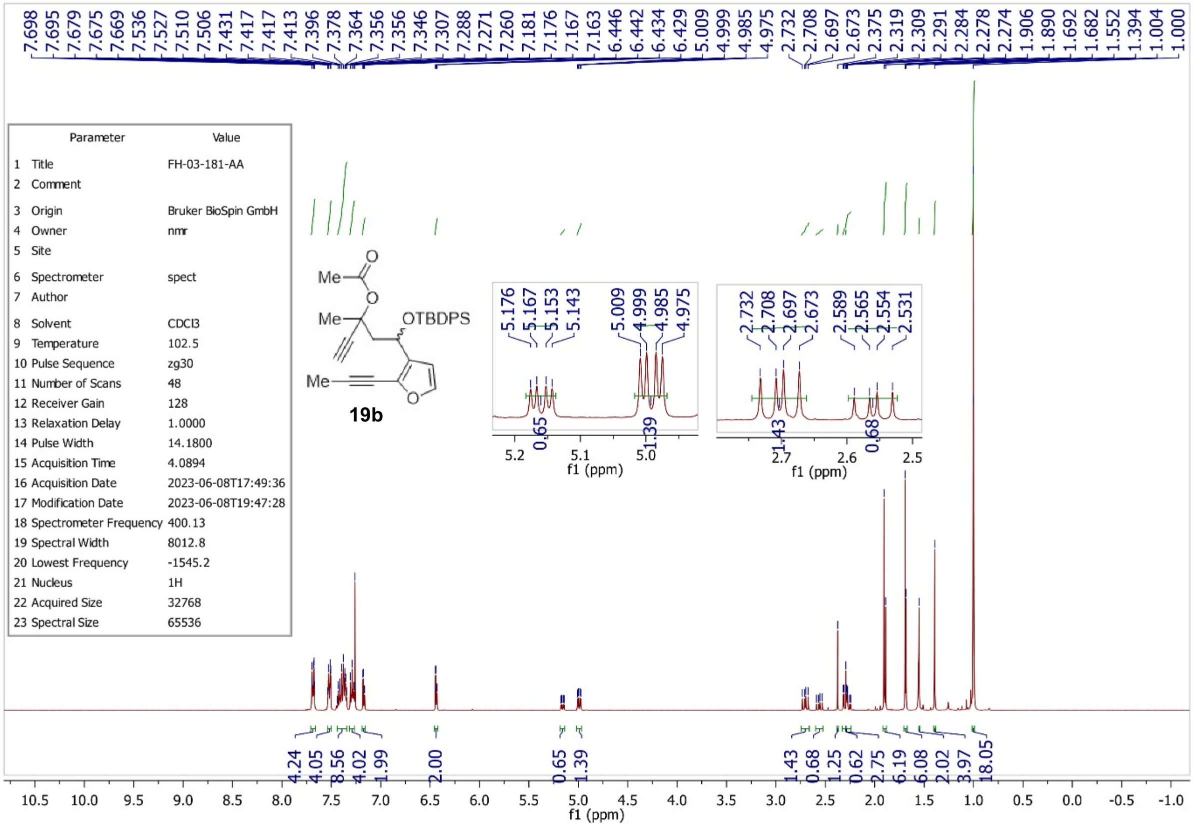
Parameter
Value
1 Title
FH-03-181-AA
2 Comment
3 Origin
Bruker BioSpin GmbH
4 Owner
nmr
5 Site
6 Spectrometer
spect
7 Author
8 Solvent
CDCl3
9 Temperature
102.5
10 Pulse Sequence
zg30
11 Number of Scans
48
12 Receiver Gain
128
13 Relaxation Delay
1.0000
14 Pulse Width
14.1800
15 Acquisition Time
4.0894
16 Acquisition Date
2023-06-08T17:49:36
17 Modification Date
2023-06-08T19:47:28
18 Spectrometer Frequency
400.13
19 Spectral Width
8012.8
20 Lowest Frequency
-1545.2
21 Nucleus
1H
22 Acquired Size
32768
23 Spectral Size
65536
Me
O
O
Me
OTBDPS
Me
O
19b
f1 (ppm)

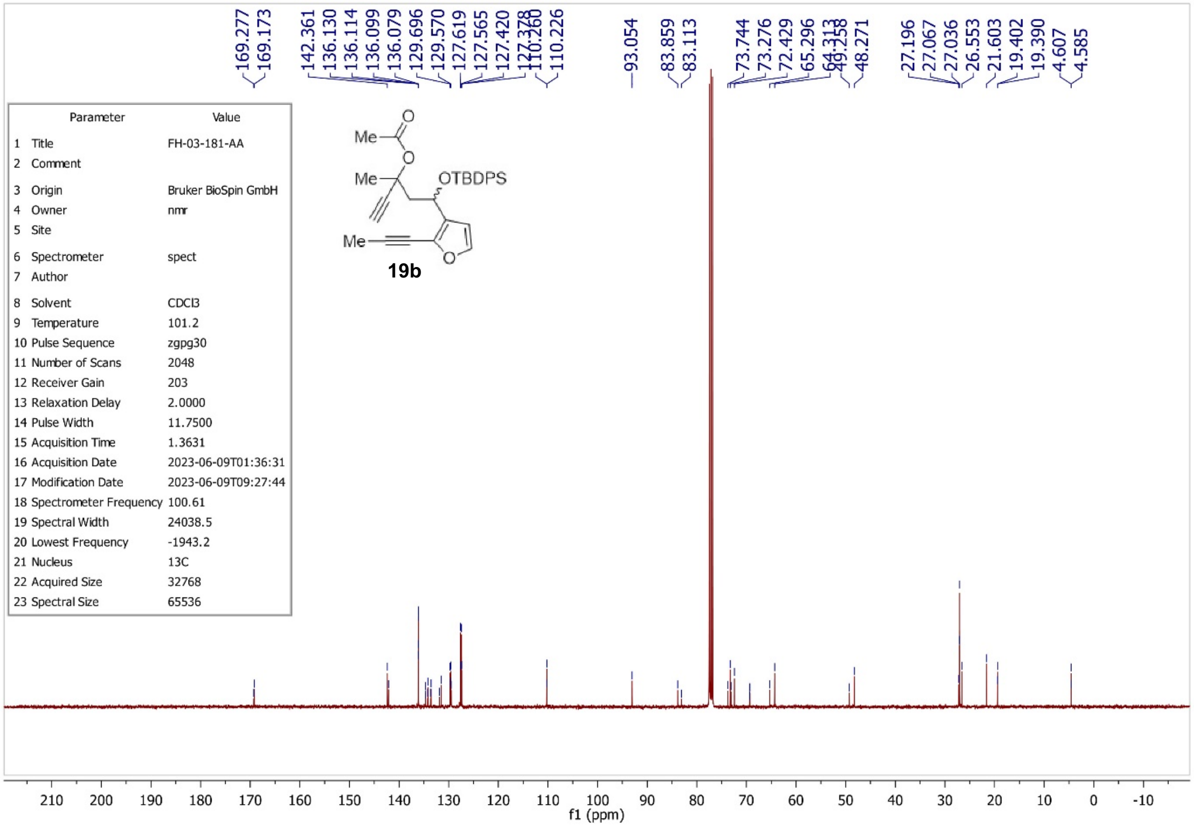
169.277
169.173
142.361
136.130
136.114
136.099
136.079
129.696
129.570
127.619
127.565
127.420
127.378
110.260
110.226
93.054
83.859
83.113
73.744
73.276
72.429
65.296
64.313
49.258
48.271
27.196
27.067
27.036
26.553
21.603
19.402
19.390
4.607
4.585
Parameter Value
1 Title FH-03-181-AA
2 Comment
3 Origin Bruker BioSpin GmbH
4 Owner nmr
5 Site
6 Spectrometer spect
7 Author
8 Solvent CDCl3
9 Temperature 101.2
10 Pulse Sequence zgpg30
11 Number of Scans 2048
12 Receiver Gain 203
13 Relaxation Delay 2.0000
14 Pulse Width 11.7500
15 Acquisition Time 1.3631
16 Acquisition Date 2023-06-09T01:36:31
17 Modification Date 2023-06-09T09:27:44
18 Spectrometer Frequency 100.61
19 Spectral Width 24038.5
20 Lowest Frequency -1943.2
21 Nucleus 13C
22 Acquired Size 32768
23 Spectral Size 65536
Me
O
Me
OTBDPS
Me
O
19b
f1 (ppm)

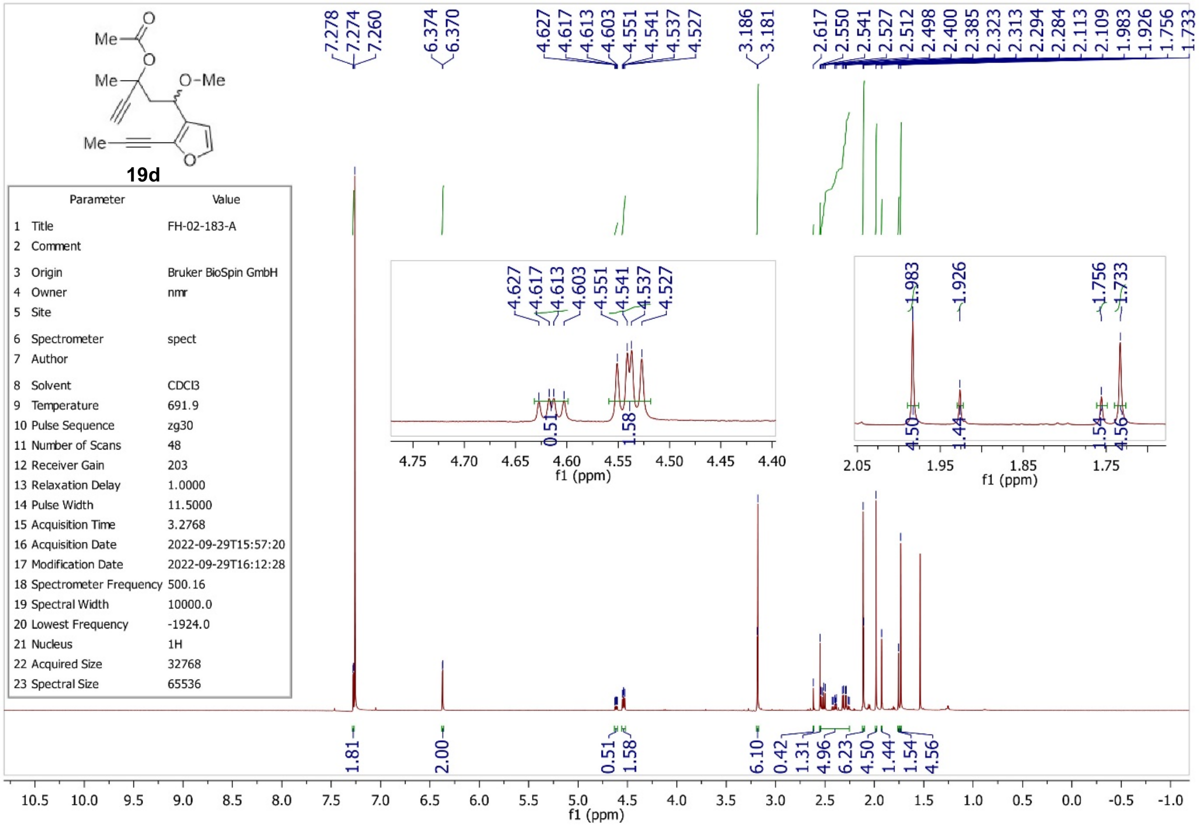
Me
O
O
Me
O–Me
Me
O
19d
Parameter Value
1 Title FH-02-183-A
2 Comment
3 Origin Bruker BioSpin GmbH
4 Owner nmr
5 Site
6 Spectrometer spect
7 Author
8 Solvent CDCl3
9 Temperature 691.9
10 Pulse Sequence zg30
11 Number of Scans 48
12 Receiver Gain 203
13 Relaxation Delay 1.0000
14 Pulse Width 11.5000
15 Acquisition Time 3.2768
16 Acquisition Date 2022-09-29T15:57:20
17 Modification Date 2022-09-29T16:12:28
18 Spectrometer Frequency 500.16
19 Spectral Width 10000.0
20 Lowest Frequency -1924.0
21 Nucleus 1H
22 Acquired Size 32768
23 Spectral Size 65536
7.278
7.274
7.260
6.374
6.370
4.627
4.617
4.613
4.603
4.551
4.541
4.537
4.527
3.186
3.181
2.617
2.550
2.541
2.527
2.512
2.498
2.400
2.385
2.323
2.313
2.294
2.284
2.113
2.109
1.983
1.926
1.756
1.733
1.81
2.00
0.51
1.58
6.10
0.42
1.31
4.96
6.23
4.50
1.44
1.54
4.56
f1 (ppm)

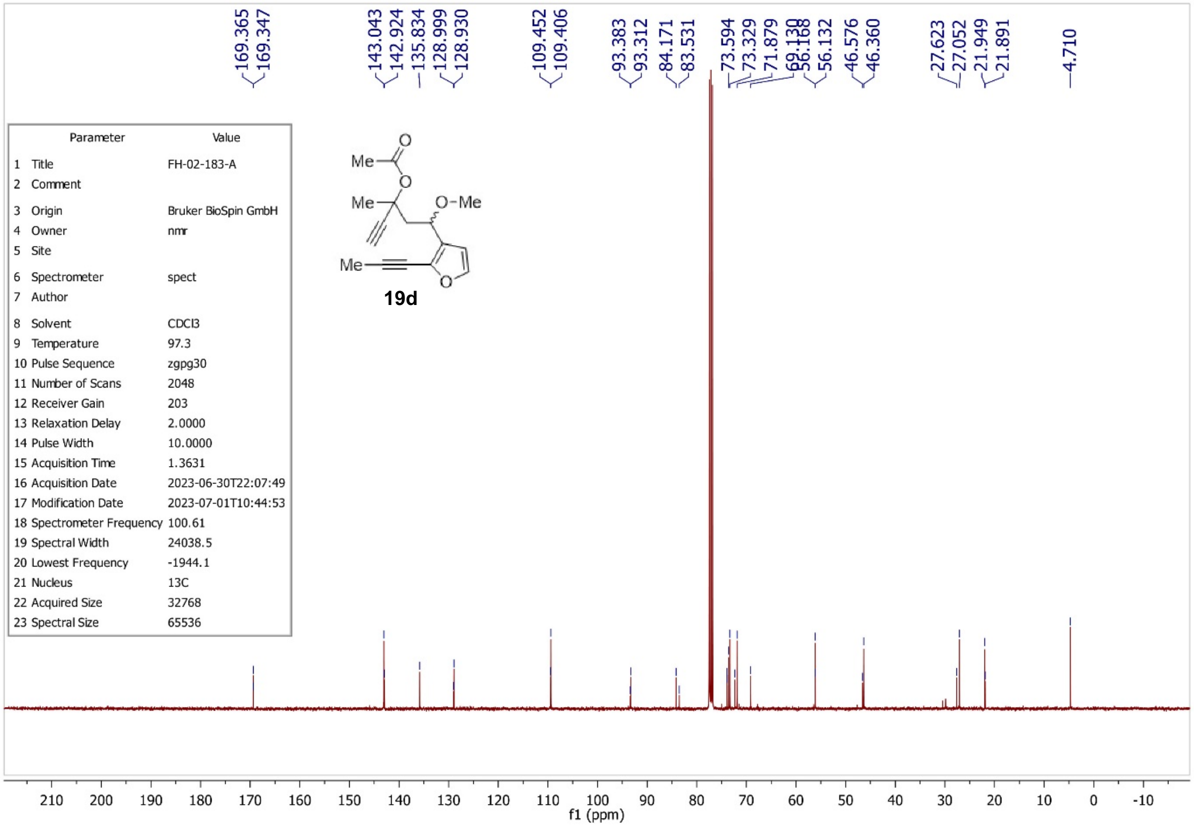
169.365
169.347
143.043
142.924
135.834
128.999
128.930
109.452
109.406
93.383
93.312
84.171
83.531
73.594
73.329
71.879
69.130
56.168
56.132
46.576
46.360
27.623
27.052
21.949
21.891
4.710
Parameter Value
1 Title FH-02-183-A
2 Comment
3 Origin Bruker BioSpin GmbH
4 Owner nmr
5 Site
6 Spectrometer spect
7 Author
8 Solvent CDCl3
9 Temperature 97.3
10 Pulse Sequence zgpg30
11 Number of Scans 2048
12 Receiver Gain 203
13 Relaxation Delay 2.0000
14 Pulse Width 10.0000
15 Acquisition Time 1.3631
16 Acquisition Date 2023-06-30T22:07:49
17 Modification Date 2023-07-01T10:44:53
18 Spectrometer Frequency 100.61
19 Spectral Width 24038.5
20 Lowest Frequency -1944.1
21 Nucleus 13C
22 Acquired Size 32768
23 Spectral Size 65536
Me
O
O
Me
O–Me
Me
O
19d
f1 (ppm)

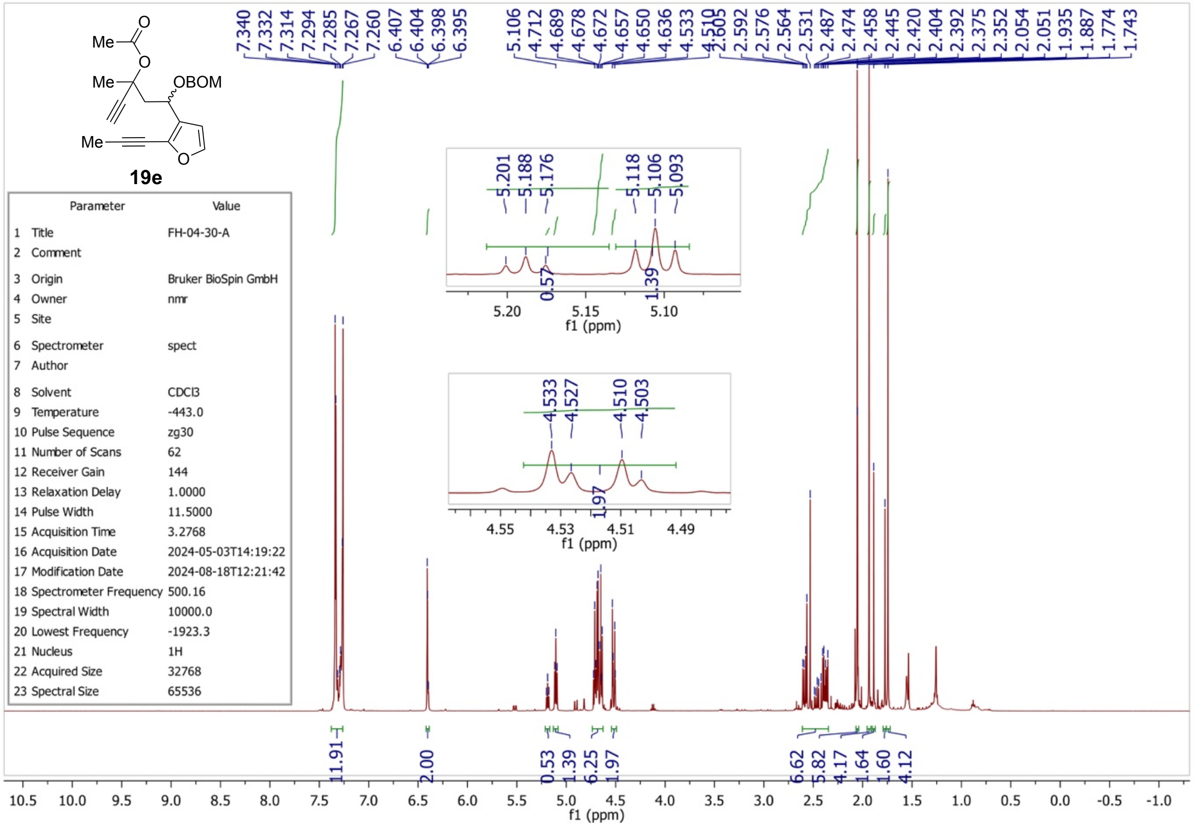
19e
Parameter Value
1 Title FH-04-30-A
2 Comment
3 Origin Bruker BioSpin GmbH
4 Owner nmr
5 Site
6 Spectrometer spect
7 Author
8 Solvent CDCl3
9 Temperature -443.0
10 Pulse Sequence zg30
11 Number of Scans 62
12 Receiver Gain 144
13 Relaxation Delay 1.0000
14 Pulse Width 11.5000
15 Acquisition Time 3.2768
16 Acquisition Date 2024-05-03T14:19:22
17 Modification Date 2024-08-18T12:21:42
18 Spectrometer Frequency 500.16
19 Spectral Width 10000.0
20 Lowest Frequency -1923.3
21 Nucleus 1H
22 Acquired Size 32768
23 Spectral Size 65536
f1 (ppm)

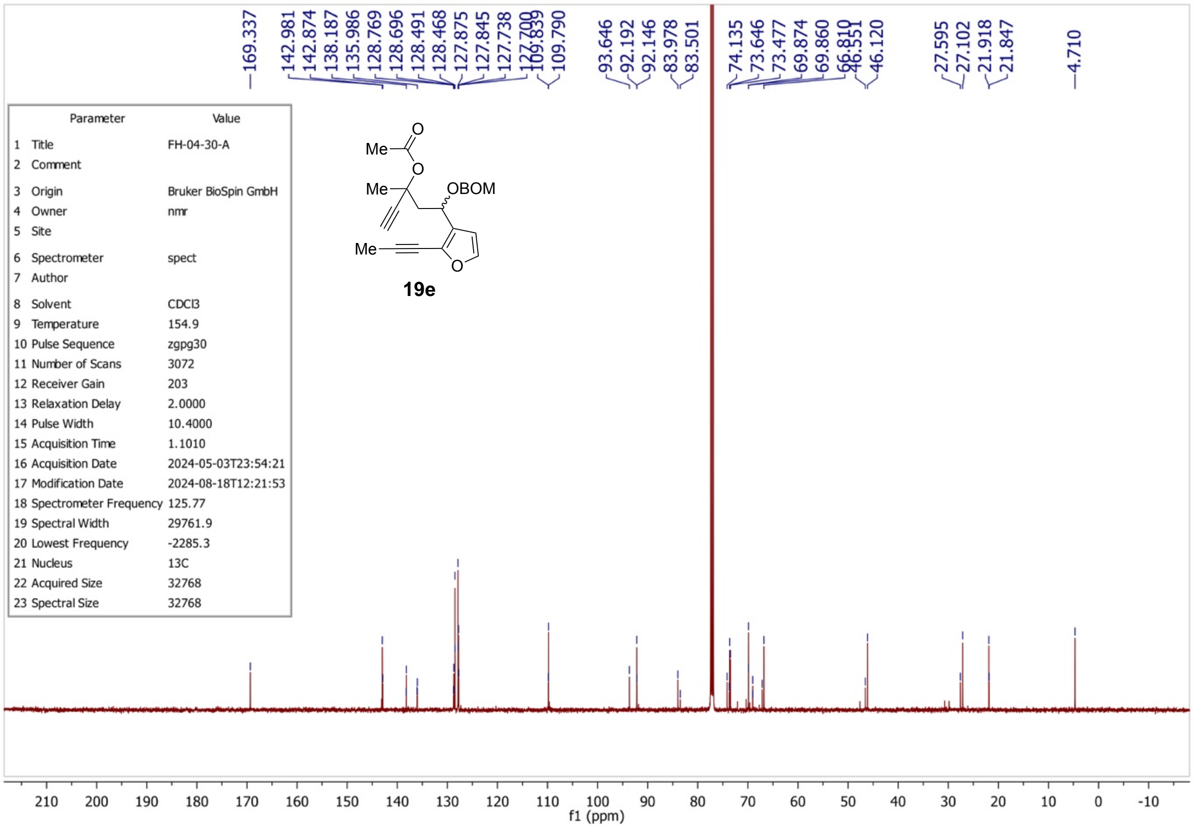

	Parameter	Value
1	Title	FH-04-30-A
2	Comment	
3	Origin	Bruker BioSpin GmbH
4	Owner	nmr
5	Site	
6	Spectrometer	spect
7	Author	
8	Solvent	CDCl3
9	Temperature	154.9
10	Pulse Sequence	zgpg30
11	Number of Scans	3072
12	Receiver Gain	203
13	Relaxation Delay	2.0000
14	Pulse Width	10.4000
15	Acquisition Time	1.1010
16	Acquisition Date	2024-05-03T23:54:21
17	Modification Date	2024-08-18T12:21:53
18	Spectrometer Frequency	125.77
19	Spectral Width	29761.9
20	Lowest Frequency	-2285.3
21	Nucleus	13C
22	Acquired Size	32768
23	Spectral Size	32768

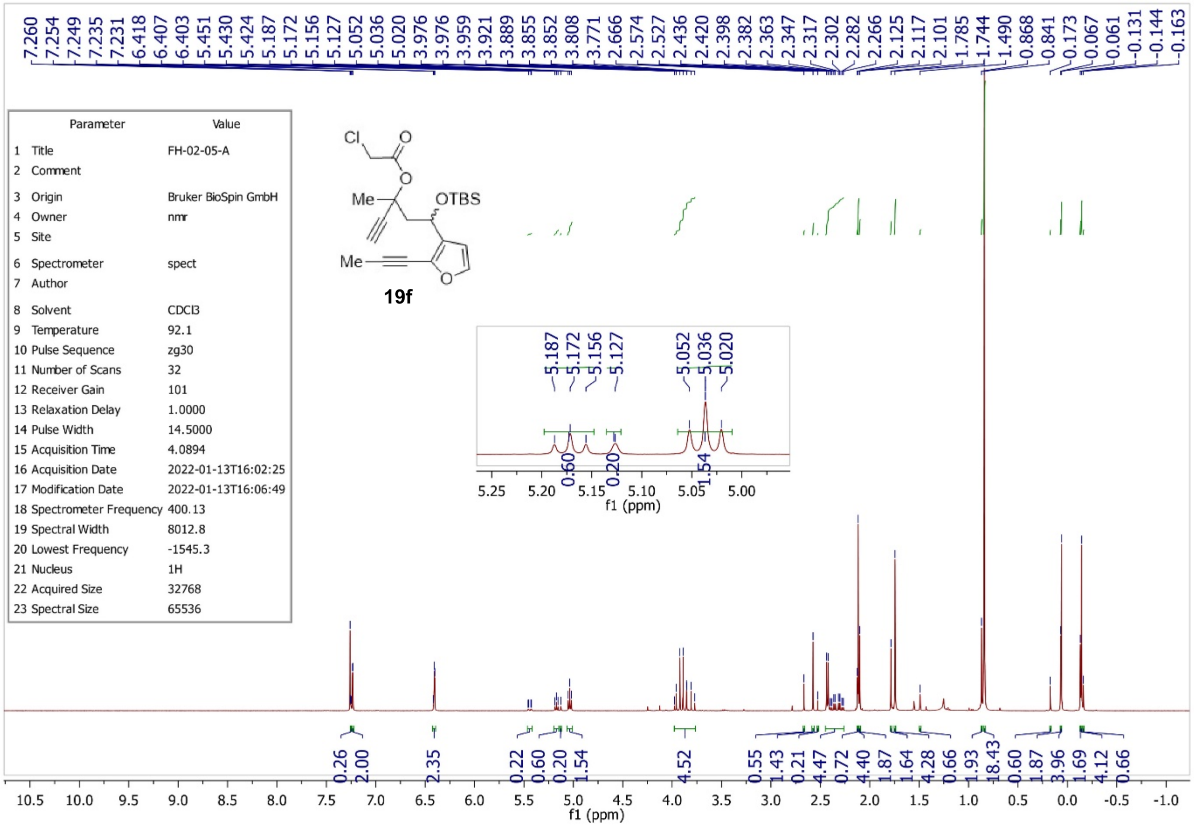

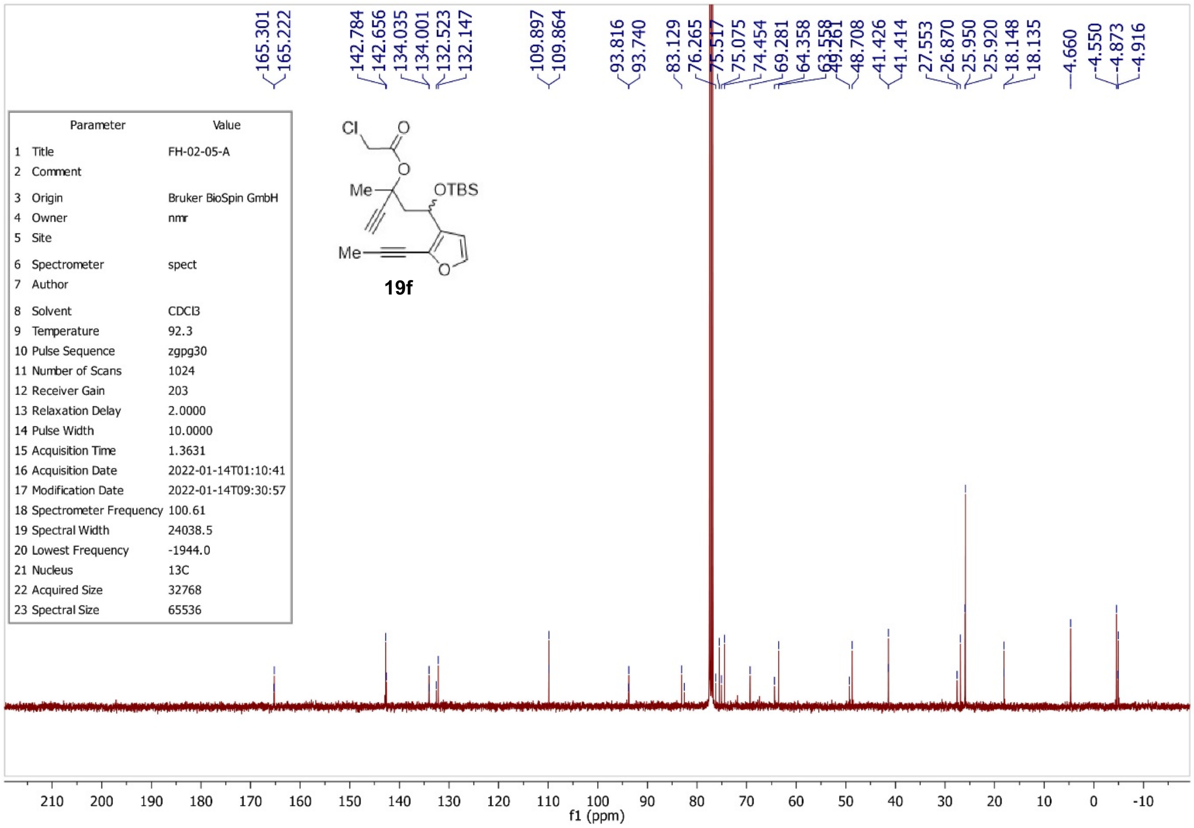

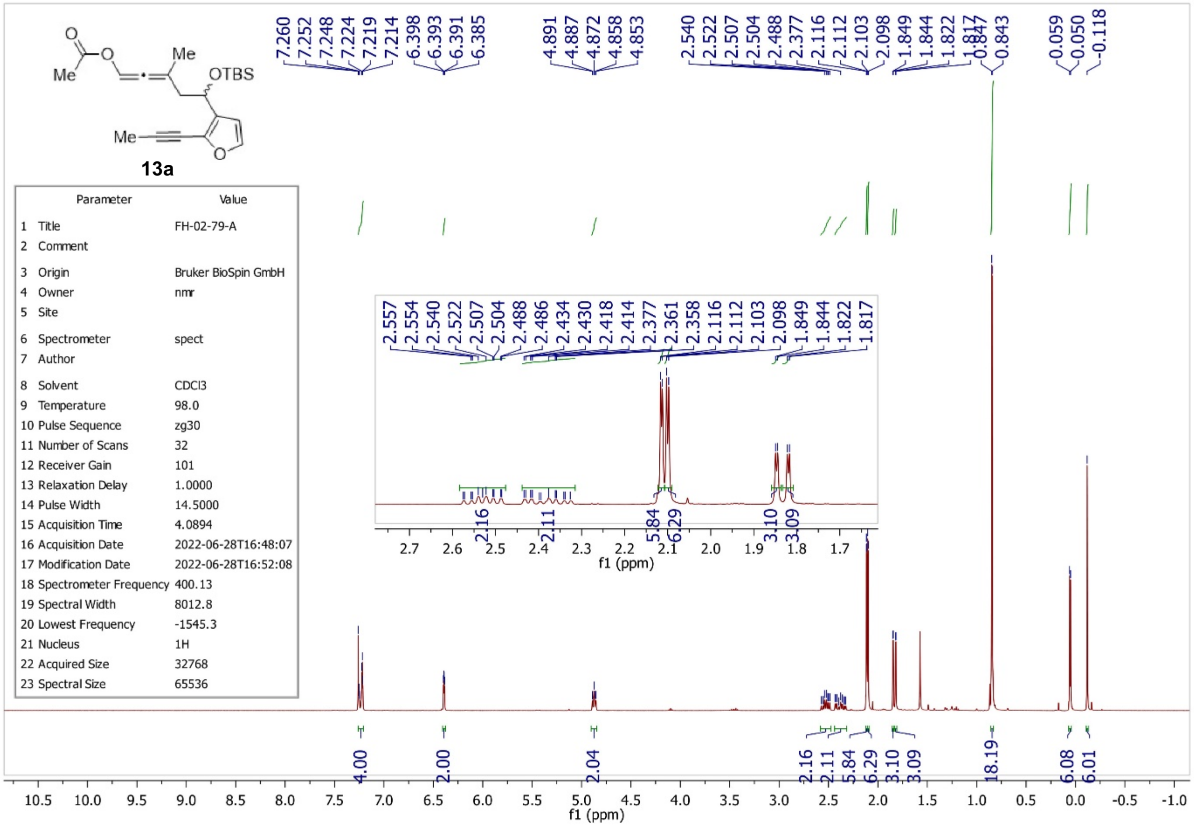

Me
OTBS
O
Me
Me
O
13a
Parameter Value
1 Title FH-02-79-A
2 Comment
3 Origin Bruker BioSpin GmbH
4 Owner nmr
5 Site
6 Spectrometer spect
7 Author
8 Solvent CDCl3
9 Temperature 98.0
10 Pulse Sequence zg30
11 Number of Scans 32
12 Receiver Gain 101
13 Relaxation Delay 1.0000
14 Pulse Width 14.5000
15 Acquisition Time 4.0894
16 Acquisition Date 2022-06-28T16:48:07
17 Modification Date 2022-06-28T16:52:08
18 Spectrometer Frequency 400.13
19 Spectral Width 8012.8
20 Lowest Frequency -1545.3
21 Nucleus 1H
22 Acquired Size 32768
23 Spectral Size 65536
7.260 7.252 7.248 7.224 7.219 7.214 6.398 6.393 6.391 6.385
4.891 4.887 4.872 4.858 4.853
2.540 2.522 2.507 2.504 2.488 2.377 2.116 2.112 2.103 2.098 1.849 1.844 1.822 1.817 0.847 0.843
0.059 0.050 -0.118
2.557 2.554 2.540 2.522 2.507 2.504 2.488 2.486 2.434 2.430 2.418 2.414 2.377 2.361 2.358 2.116 2.112 2.103 2.098 1.849 1.844 1.822 1.817
2.16 2.11 5.84 6.29 3.10 3.09
f1 (ppm)
4.00 2.00 2.04 2.16 2.11 5.84 6.29 3.10 3.09 18.19 6.08 6.01
f1 (ppm)

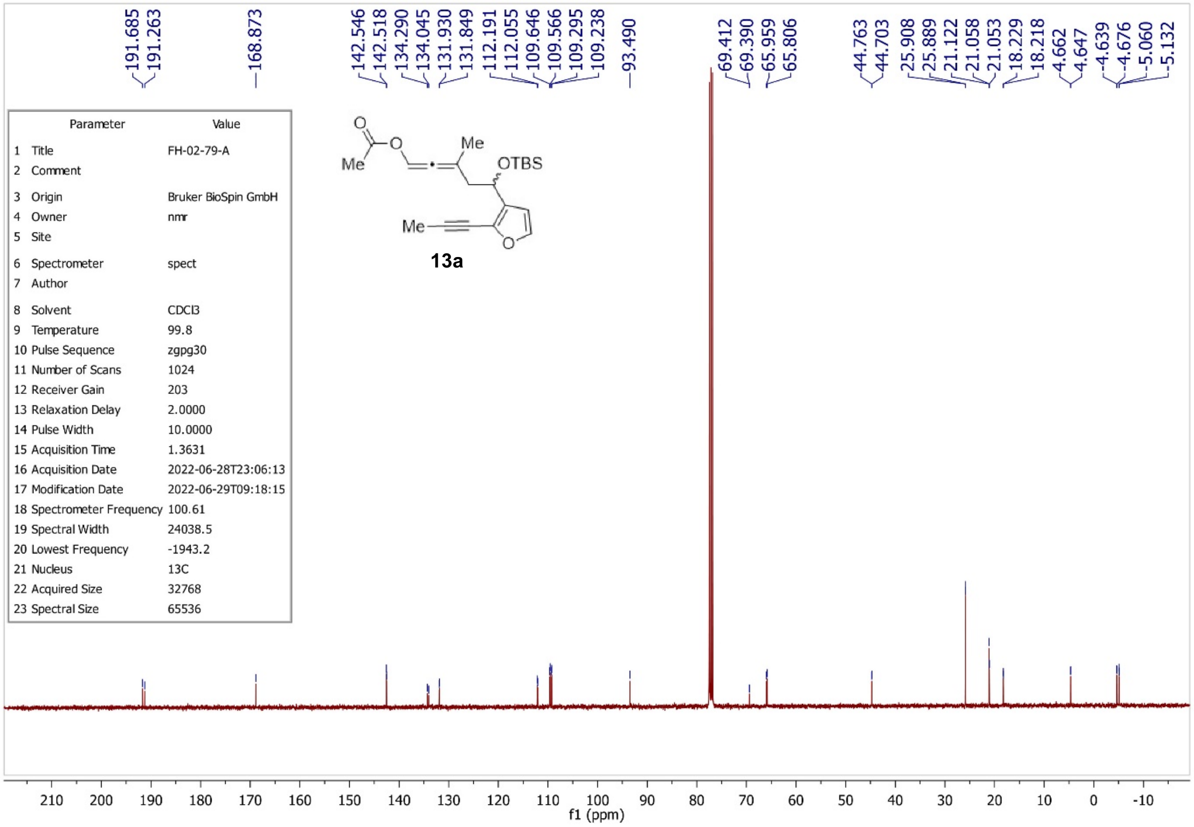
191.685
191.263
168.873
142.546
142.518
134.290
134.045
131.930
131.849
112.191
112.055
109.646
109.566
109.295
109.238
93.490
69.412
69.390
65.959
65.806
44.763
44.703
25.908
25.889
21.122
21.058
21.053
18.229
18.218
4.662
4.647
-4.639
-4.676
-5.060
-5.132
Parameter Value
1 Title FH-02-79-A
2 Comment
3 Origin Bruker BioSpin GmbH
4 Owner nmr
5 Site
6 Spectrometer spect
7 Author
8 Solvent CDCl3
9 Temperature 99.8
10 Pulse Sequence zgpg30
11 Number of Scans 1024
12 Receiver Gain 203
13 Relaxation Delay 2.0000
14 Pulse Width 10.0000
15 Acquisition Time 1.3631
16 Acquisition Date 2022-06-28T23:06:13
17 Modification Date 2022-06-29T09:18:15
18 Spectrometer Frequency 100.61
19 Spectral Width 24038.5
20 Lowest Frequency -1943.2
21 Nucleus 13C
22 Acquired Size 32768
23 Spectral Size 65536
O
O
Me
Me
OTBS
Me
O
13a
210 200 190 180 170 160 150 140 130 120 110 100 90 80 70 60 50 40 30 20 10 0 -10
f1 (ppm)

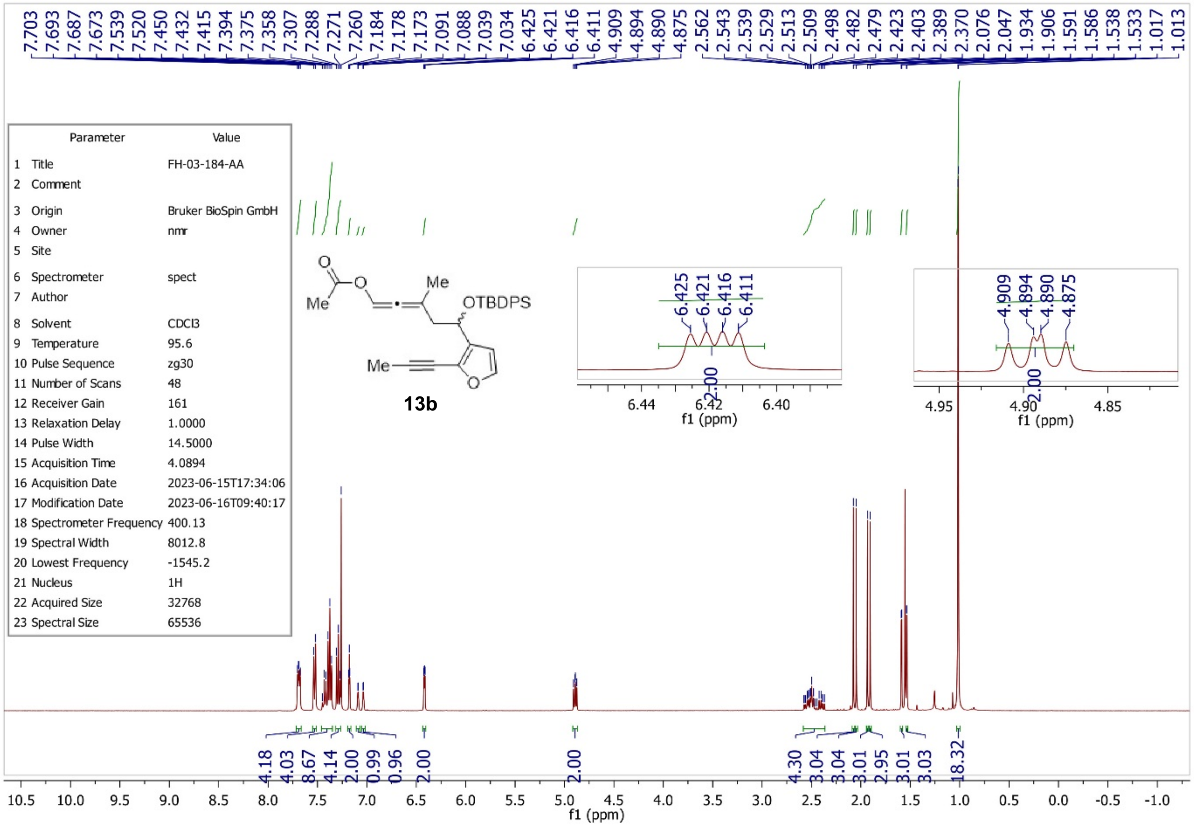

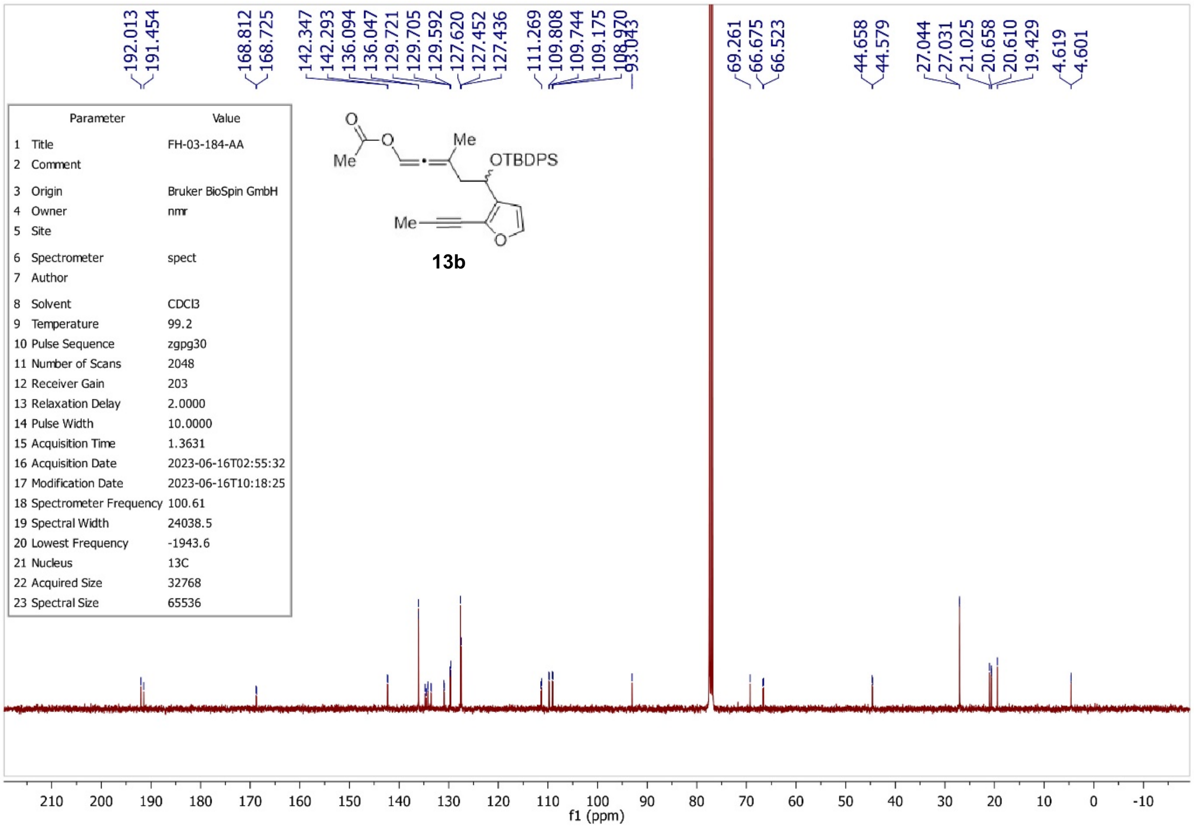

192.013
191.454
168.812
168.725
142.347
142.293
136.094
136.047
129.721
129.705
129.592
127.620
127.452
127.436
111.269
109.808
109.744
109.175
108.970
93.043
69.261
66.675
66.523
44.658
44.579
27.044
27.031
21.025
20.658
20.610
19.429
4.619
4.601
Parameter Value
1 Title FH-03-184-AA
2 Comment
3 Origin Bruker BioSpin GmbH
4 Owner nmr
5 Site
6 Spectrometer spect
7 Author
8 Solvent CDCl3
9 Temperature 99.2
10 Pulse Sequence zgpg30
11 Number of Scans 2048
12 Receiver Gain 203
13 Relaxation Delay 2.0000
14 Pulse Width 10.0000
15 Acquisition Time 1.3631
16 Acquisition Date 2023-06-16T02:55:32
17 Modification Date 2023-06-16T10:18:25
18 Spectrometer Frequency 100.61
19 Spectral Width 24038.5
20 Lowest Frequency -1943.6
21 Nucleus 13C
22 Acquired Size 32768
23 Spectral Size 65536
O
O
Me
Me
OTBDPS
Me
O
13b
210 200 190 180 170 160 150 140 130 120 110 100 90 80 70 60 50 40 30 20 10 0 -10
f1 (ppm)

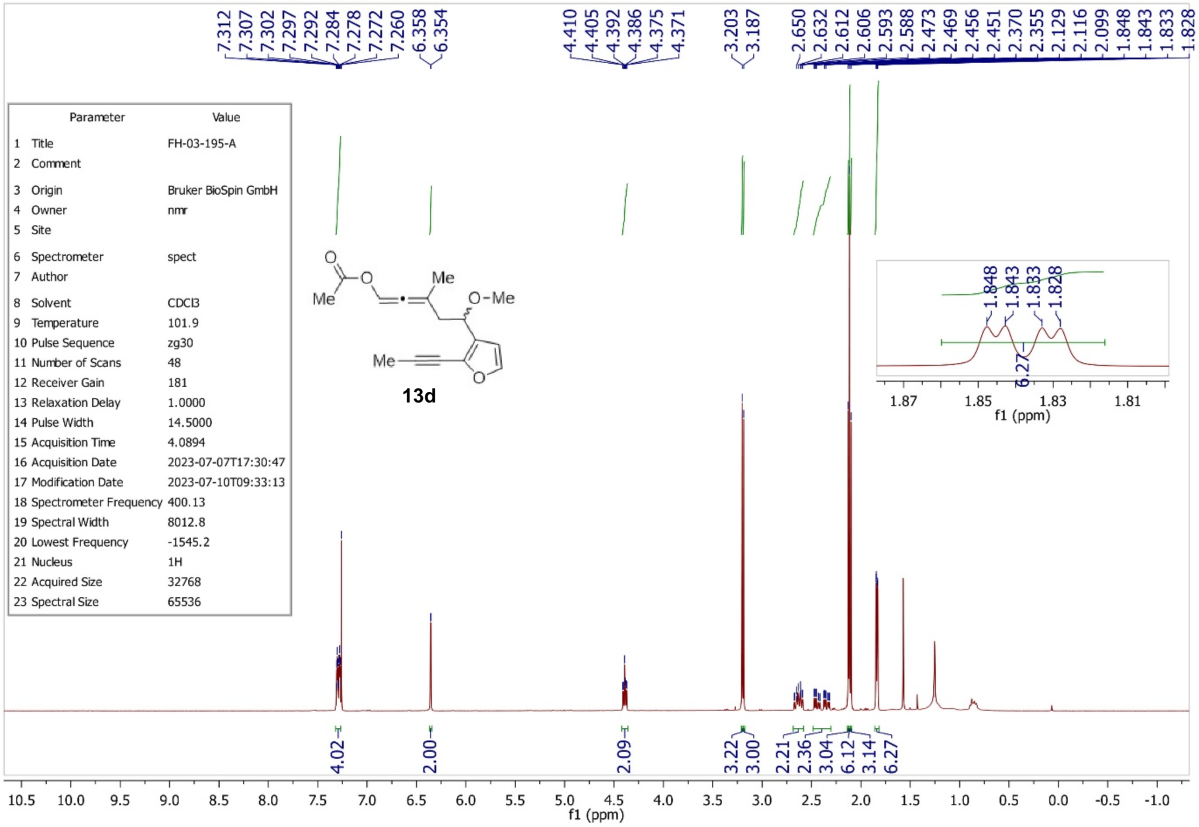

7.312
7.307
7.302
7.297
7.292
7.284
7.278
7.272
7.260
6.358
6.354
4.410
4.405
4.392
4.386
4.375
4.371
3.203
3.187
2.650
2.632
2.612
2.606
2.593
2.588
2.473
2.469
2.456
2.451
2.370
2.355
2.129
2.116
2.099
1.848
1.843
1.833
1.828
Parameter
Value
1 Title
FH-03-195-A
2 Comment
3 Origin
Bruker BioSpin GmbH
4 Owner
nmr
5 Site
6 Spectrometer
spect
7 Author
8 Solvent
CDCl3
9 Temperature
101.9
10 Pulse Sequence
zg30
11 Number of Scans
48
12 Receiver Gain
181
13 Relaxation Delay
1.0000
14 Pulse Width
14.5000
15 Acquisition Time
4.0894
16 Acquisition Date
2023-07-07T17:30:47
17 Modification Date
2023-07-10T09:33:13
18 Spectrometer Frequency
400.13
19 Spectral Width
8012.8
20 Lowest Frequency
-1545.2
21 Nucleus
1H
22 Acquired Size
32768
23 Spectral Size
65536
13d
4.02
2.00
2.09
3.22
3.00
2.21
2.36
3.04
6.12
3.14
6.27
f1 (ppm)

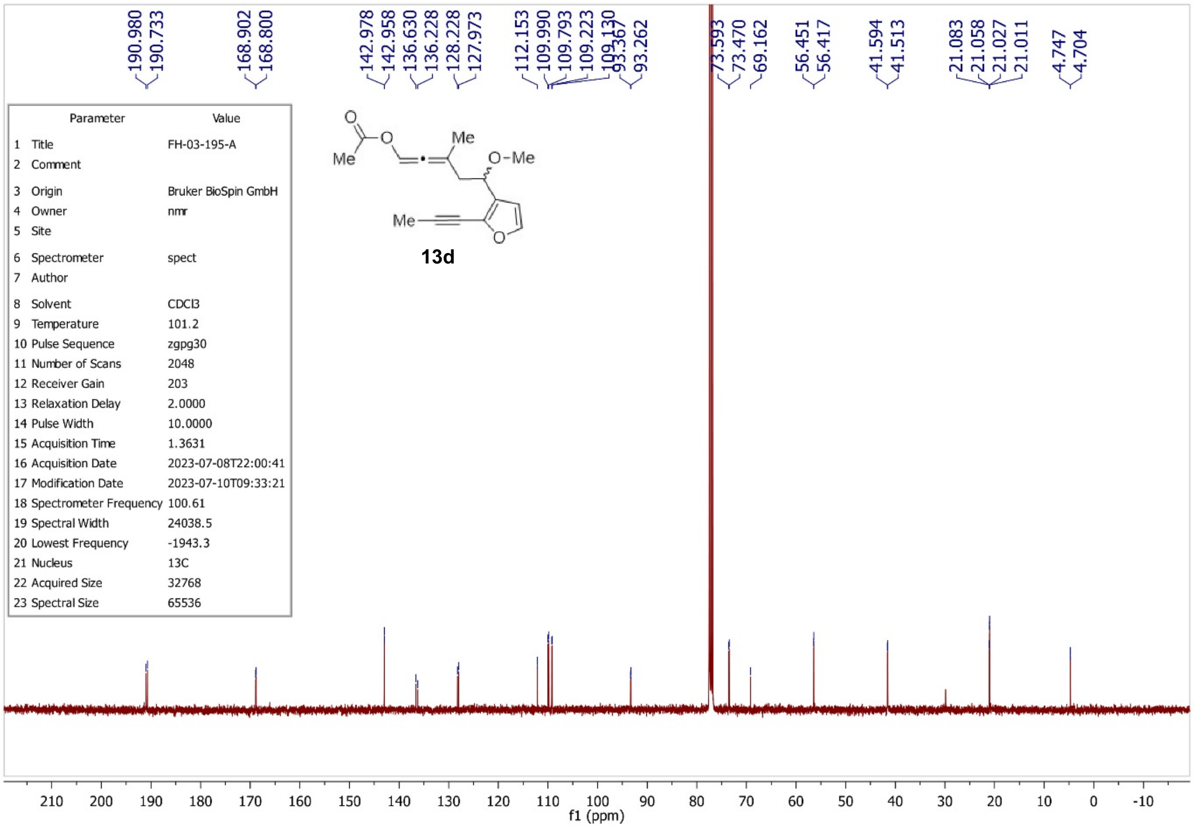
190.980
190.733
168.902
168.800
142.978
142.958
136.630
136.228
128.228
127.973
112.153
109.990
109.793
109.223
109.130
93.367
93.262
73.593
73.470
69.162
56.451
56.417
41.594
41.513
21.083
21.058
21.027
21.011
4.747
4.704
Parameter | Value
1 Title | FH-03-195-A
2 Comment
3 Origin | Bruker BioSpin GmbH
4 Owner | nmr
5 Site
6 Spectrometer | spect
7 Author
8 Solvent | CDCl3
9 Temperature | 101.2
10 Pulse Sequence | zgpg30
11 Number of Scans | 2048
12 Receiver Gain | 203
13 Relaxation Delay | 2.0000
14 Pulse Width | 10.0000
15 Acquisition Time | 1.3631
16 Acquisition Date | 2023-07-08T22:00:41
17 Modification Date | 2023-07-10T09:33:21
18 Spectrometer Frequency | 100.61
19 Spectral Width | 24038.5
20 Lowest Frequency | -1943.3
21 Nucleus | 13C
22 Acquired Size | 32768
23 Spectral Size | 65536
13d
f1 (ppm)

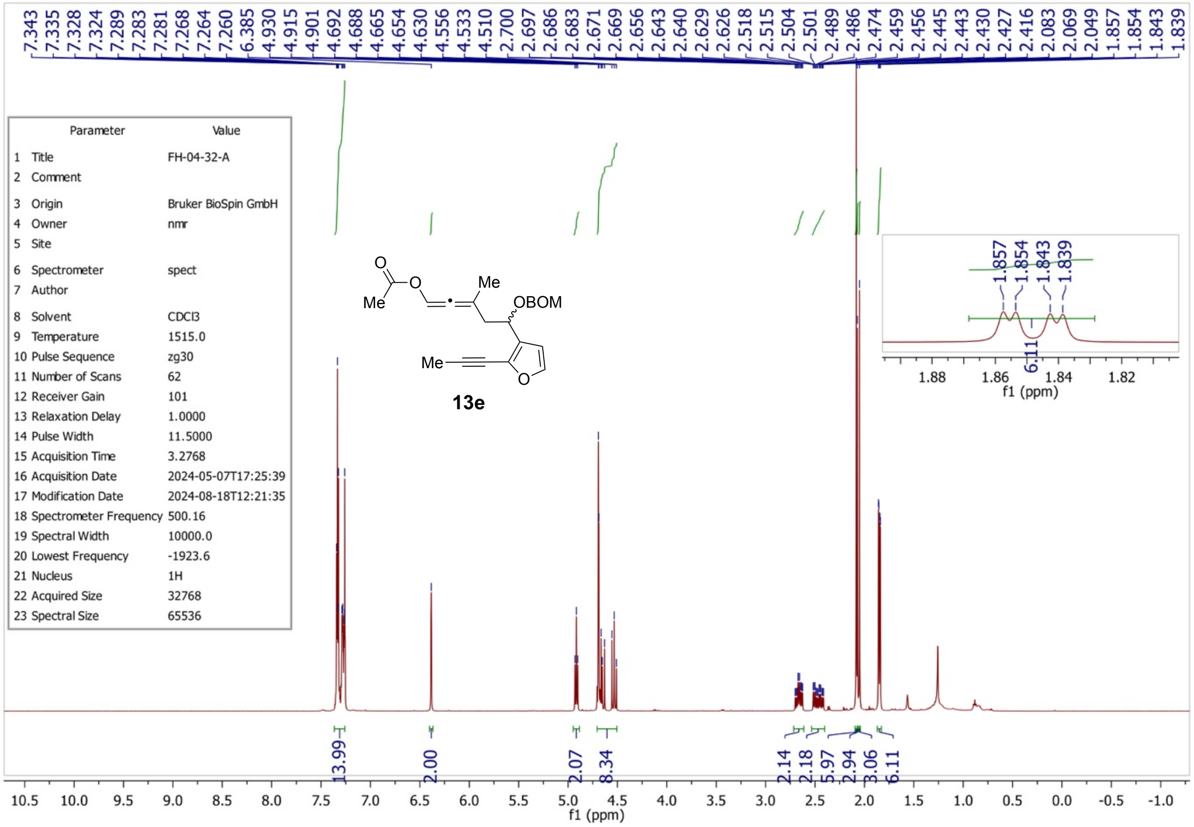
Parameter Value
1 Title FH-04-32-A
2 Comment
3 Origin Bruker BioSpin GmbH
4 Owner nmr
5 Site
6 Spectrometer spect
7 Author
8 Solvent CDCl3
9 Temperature 1515.0
10 Pulse Sequence zg30
11 Number of Scans 62
12 Receiver Gain 101
13 Relaxation Delay 1.0000
14 Pulse Width 11.5000
15 Acquisition Time 3.2768
16 Acquisition Date 2024-05-07T17:25:39
17 Modification Date 2024-08-18T12:21:35
18 Spectrometer Frequency 500.16
19 Spectral Width 10000.0
20 Lowest Frequency -1923.6
21 Nucleus 1H
22 Acquired Size 32768
23 Spectral Size 65536
OBOM
Me
13e
f1 (ppm)

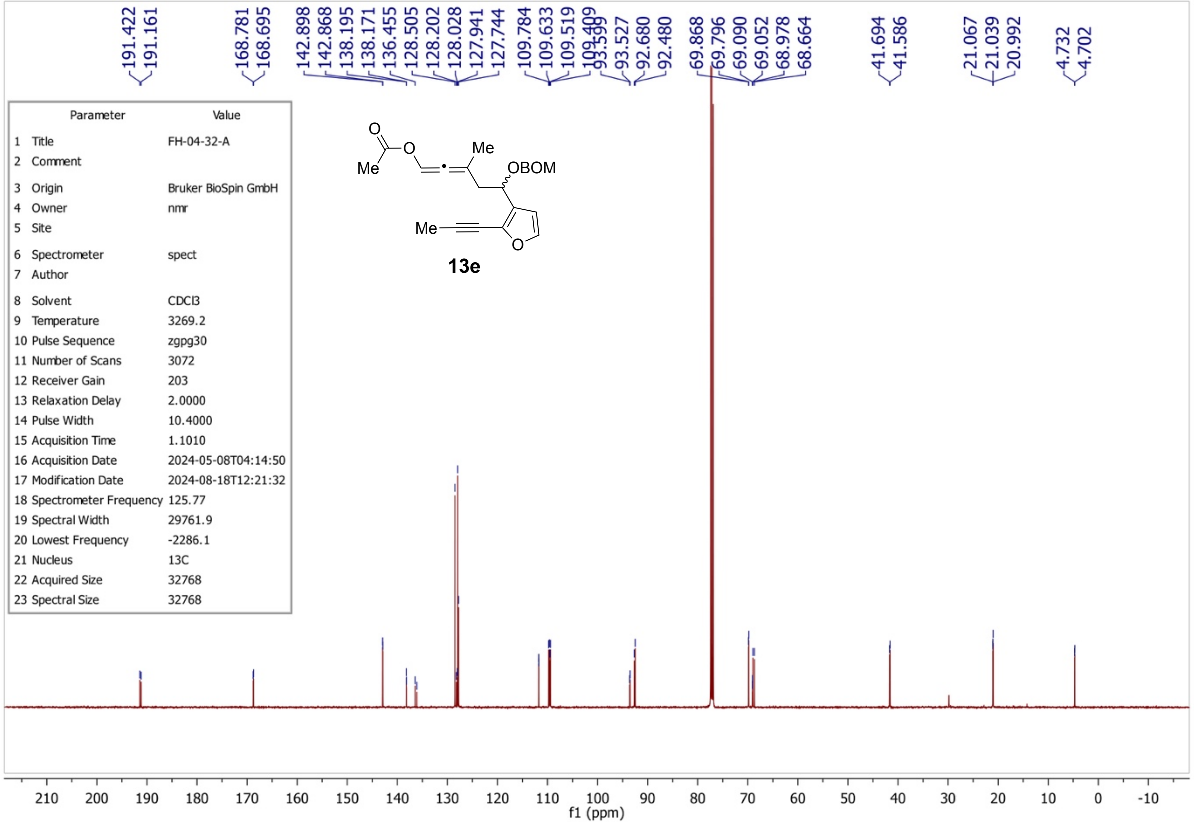

191.422
191.161
168.781
168.695
142.898
142.868
138.195
138.171
136.455
128.505
128.202
128.028
127.941
127.744
109.784
109.633
109.519
109.409
93.599
93.527
92.680
92.480
69.868
69.796
69.090
69.052
68.978
68.664
41.694
41.586
21.067
21.039
20.992
4.732
4.702
Parameter Value
1 Title FH-04-32-A
2 Comment
3 Origin Bruker BioSpin GmbH
4 Owner nmr
5 Site
6 Spectrometer spect
7 Author
8 Solvent CDCl3
9 Temperature 3269.2
10 Pulse Sequence zgpg30
11 Number of Scans 3072
12 Receiver Gain 203
13 Relaxation Delay 2.0000
14 Pulse Width 10.4000
15 Acquisition Time 1.1010
16 Acquisition Date 2024-05-08T04:14:50
17 Modification Date 2024-08-18T12:21:32
18 Spectrometer Frequency 125.77
19 Spectral Width 29761.9
20 Lowest Frequency -2286.1
21 Nucleus 13C
22 Acquired Size 32768
23 Spectral Size 32768
O
O
Me
Me
OBOM
Me
O
13e
f1 (ppm)

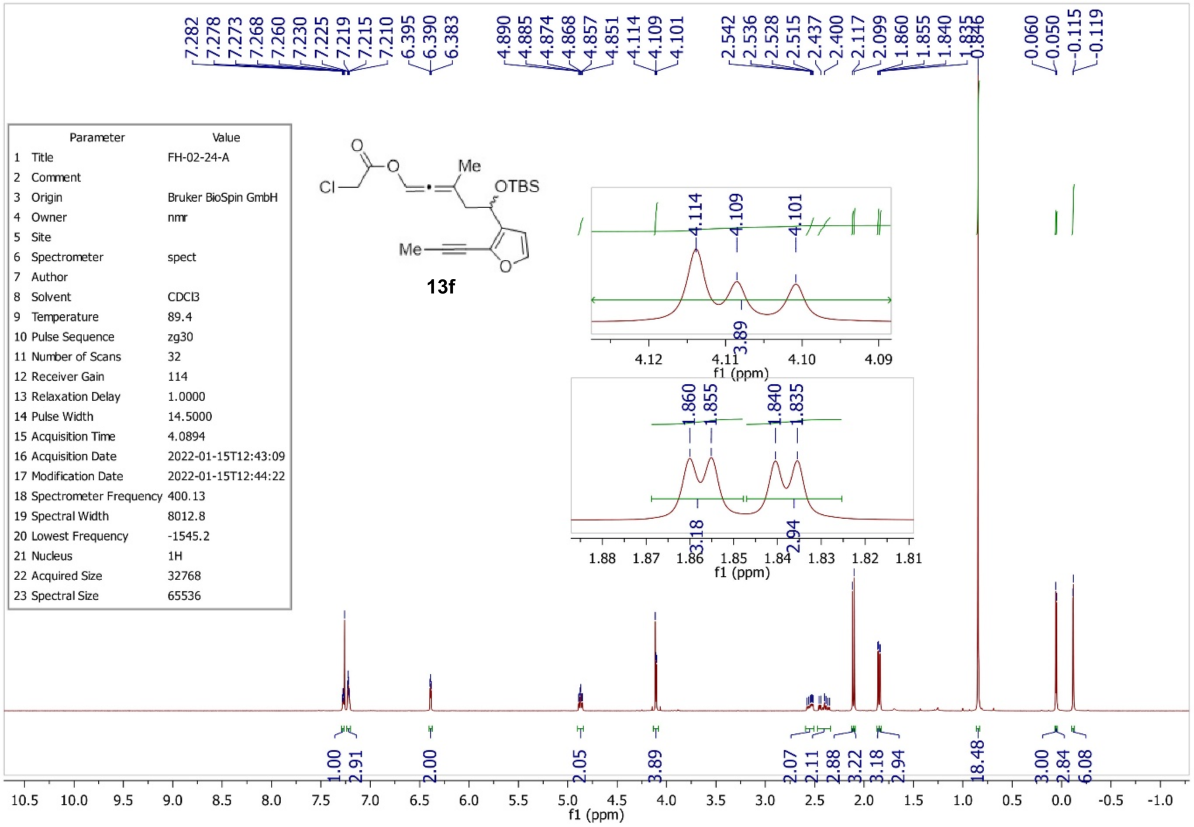

13f
OTBS
Me
Cl
O
Parameter
Value
1 Title
FH-02-24-A
3 Origin
Bruker BioSpin GmbH
8 Solvent
CDCl3
9 Temperature
89.4
10 Pulse Sequence
zg30
11 Number of Scans
32
18 Spectrometer Frequency
400.13
21 Nucleus
1H
f1 (ppm)

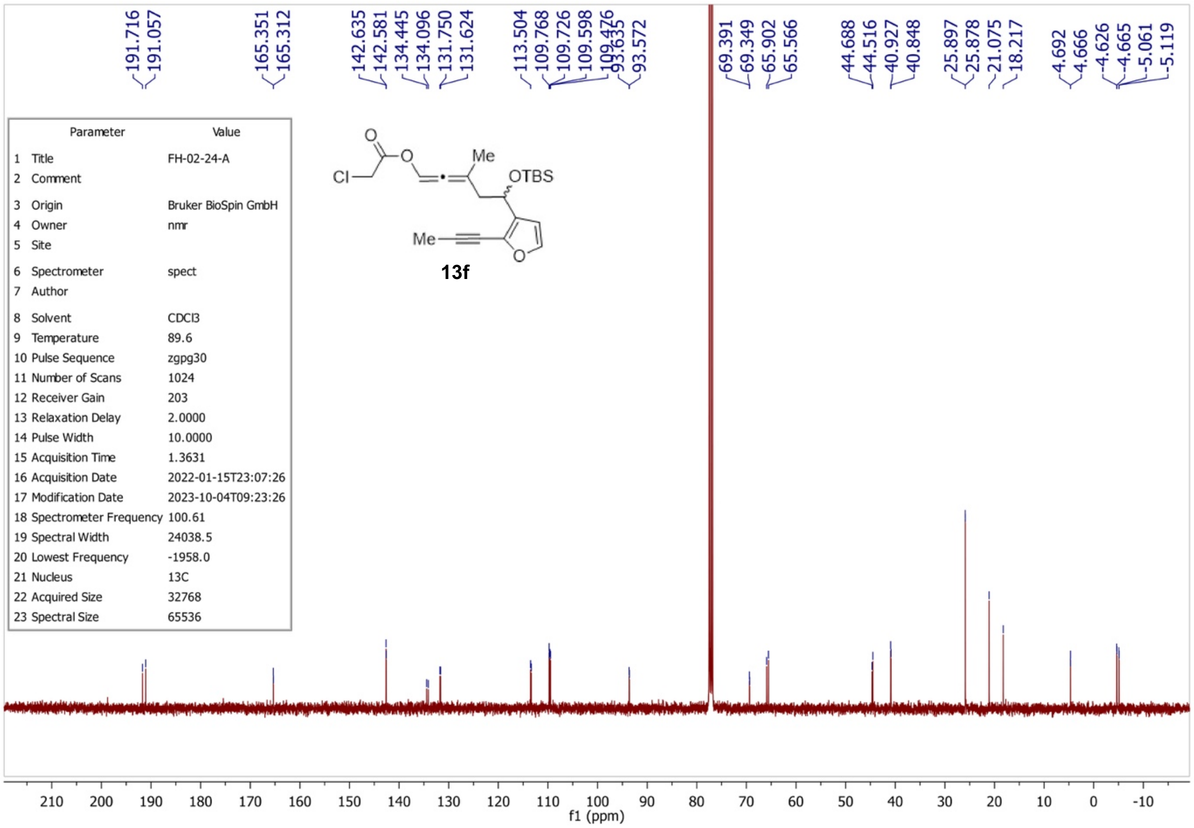
191.716
191.057
165.351
165.312
142.635
142.581
134.445
134.096
131.750
131.624
113.504
109.768
109.726
109.598
109.476
93.635
93.572
69.391
69.349
65.902
65.566
44.688
44.516
40.927
40.848
25.897
25.878
21.075
18.217
4.692
4.666
-4.626
-4.665
-5.061
-5.119
Parameter Value
1 Title FH-02-24-A
2 Comment
3 Origin Bruker BioSpin GmbH
4 Owner nmr
5 Site
6 Spectrometer spect
7 Author
8 Solvent CDCl3
9 Temperature 89.6
10 Pulse Sequence zgpg30
11 Number of Scans 1024
12 Receiver Gain 203
13 Relaxation Delay 2.0000
14 Pulse Width 10.0000
15 Acquisition Time 1.3631
16 Acquisition Date 2022-01-15T23:07:26
17 Modification Date 2023-10-04T09:23:26
18 Spectrometer Frequency 100.61
19 Spectral Width 24038.5
20 Lowest Frequency -1958.0
21 Nucleus 13C
22 Acquired Size 32768
23 Spectral Size 65536
Cl
O
O
Me
OTBS
Me
O
13f
210 200 190 180 170 160 150 140 130 120 110 100 90 80 70 60 50 40 30 20 10 0 -10
f1 (ppm)

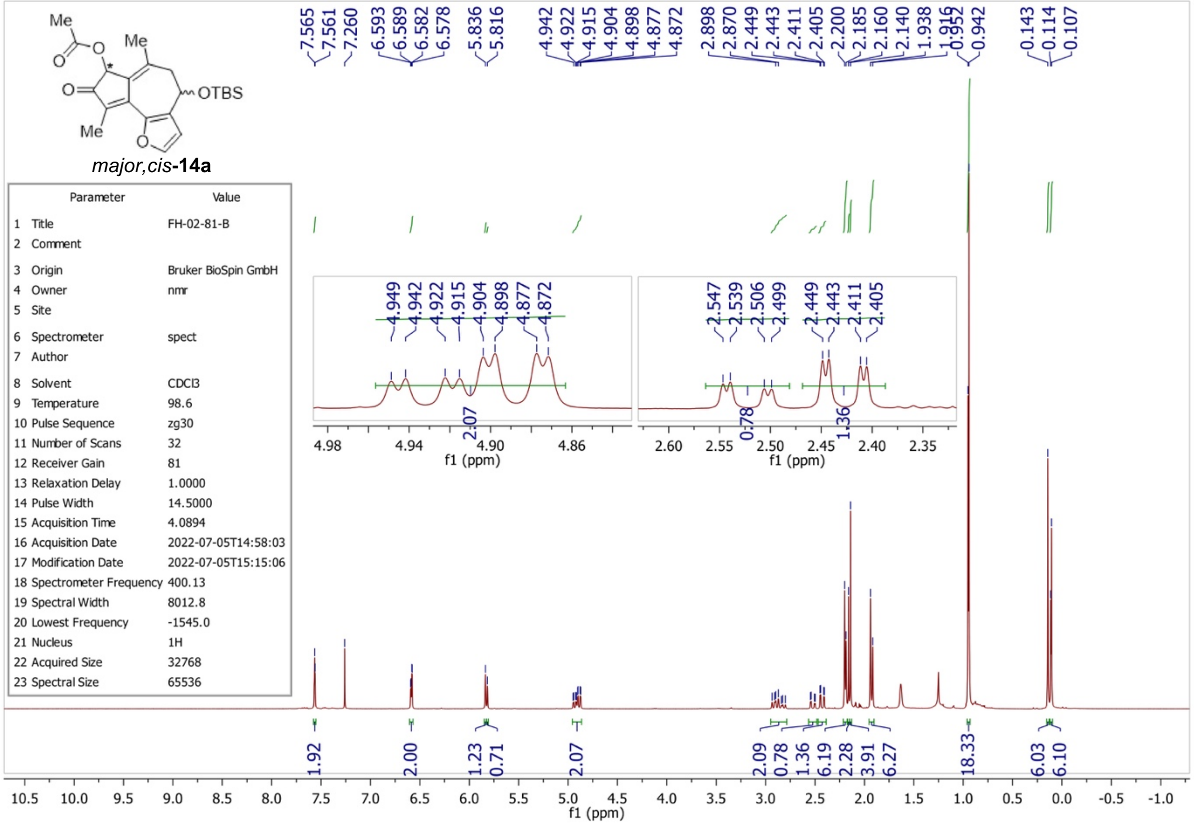
major,cis-14a
Parameter | Value
1 Title FH-02-81-B
2 Comment
3 Origin Bruker BioSpin GmbH
4 Owner nmr
5 Site
6 Spectrometer spect
7 Author
8 Solvent CDCl3
9 Temperature 98.6
10 Pulse Sequence zg30
11 Number of Scans 32
12 Receiver Gain 81
13 Relaxation Delay 1.0000
14 Pulse Width 14.5000
15 Acquisition Time 4.0894
16 Acquisition Date 2022-07-05T14:58:03
17 Modification Date 2022-07-05T15:15:06
18 Spectrometer Frequency 400.13
19 Spectral Width 8012.8
20 Lowest Frequency -1545.0
21 Nucleus 1H
22 Acquired Size 32768
23 Spectral Size 65536
f1 (ppm)

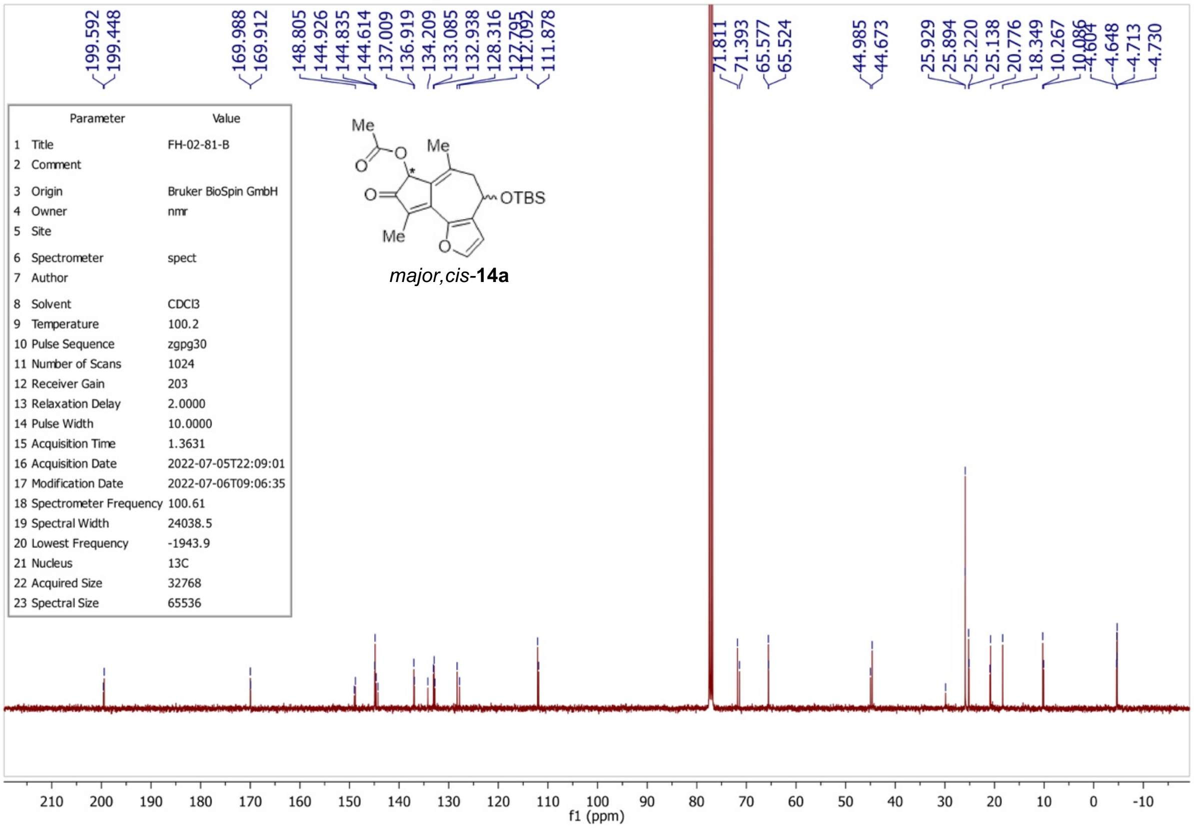
199.592
199.448
169.988
169.912
148.805
144.926
144.835
144.614
137.009
136.919
134.209
133.085
132.938
128.316
127.795
112.092
111.878
71.811
71.393
65.577
65.524
44.985
44.673
25.929
25.894
25.220
25.138
20.776
18.349
10.267
10.086
-4.604
-4.648
-4.713
-4.730
Parameter Value
1 Title FH-02-81-B
2 Comment
3 Origin Bruker BioSpin GmbH
4 Owner nmr
5 Site
6 Spectrometer spect
7 Author
8 Solvent CDCl3
9 Temperature 100.2
10 Pulse Sequence zgpg30
11 Number of Scans 1024
12 Receiver Gain 203
13 Relaxation Delay 2.0000
14 Pulse Width 10.0000
15 Acquisition Time 1.3631
16 Acquisition Date 2022-07-05T22:09:01
17 Modification Date 2022-07-06T09:06:35
18 Spectrometer Frequency 100.61
19 Spectral Width 24038.5
20 Lowest Frequency -1943.9
21 Nucleus 13C
22 Acquired Size 32768
23 Spectral Size 65536
Me
O
O
Me
O
OTBS
Me
O
major,cis-14a
210 200 190 180 170 160 150 140 130 120 110 100 90 80 70 60 50 40 30 20 10 0 -10
f1 (ppm)

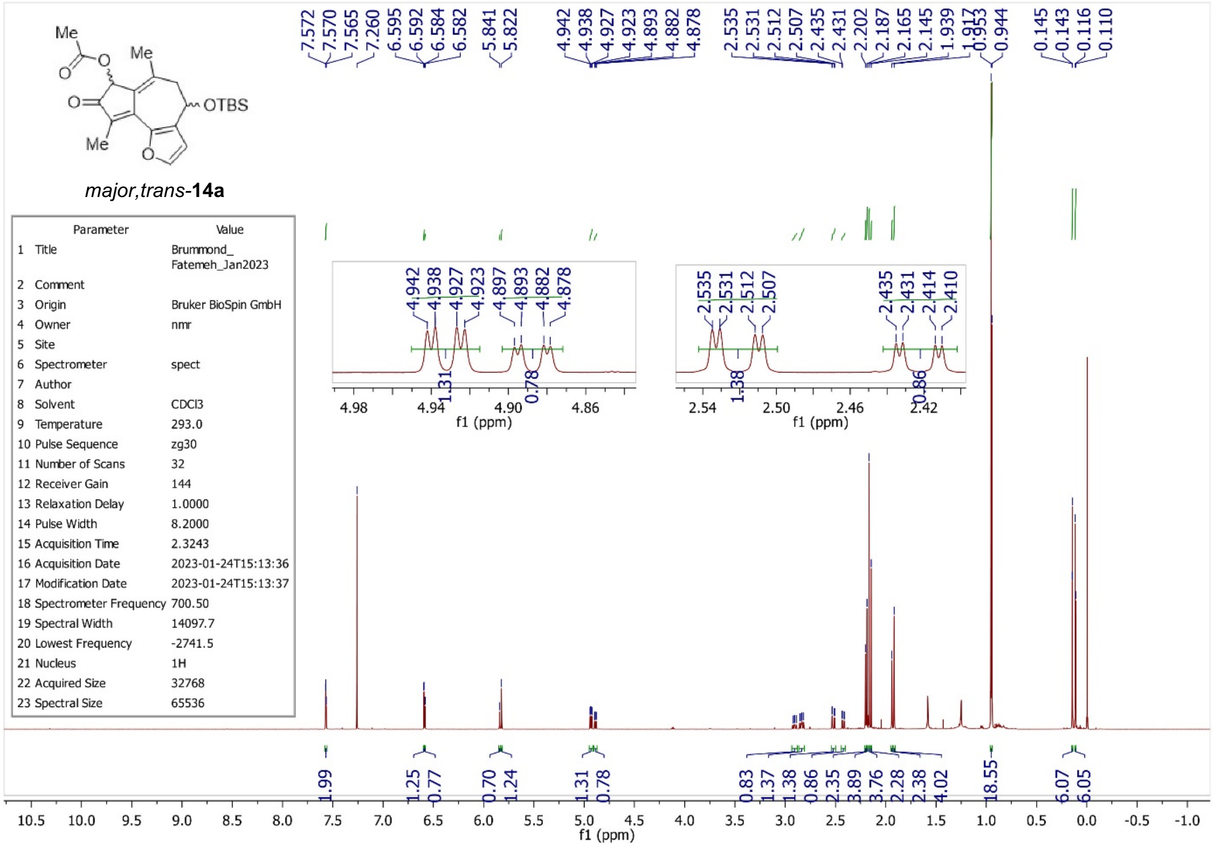

major,trans-**14a**

Parameter	Value
1 Title	Brummond_ Fatemeh_Jan2023
2 Comment	
3 Origin	Bruker BioSpin GmbH
4 Owner	nmr
5 Site	
6 Spectrometer	spect
7 Author	
8 Solvent	CDCl3
9 Temperature	293.0
10 Pulse Sequence	zg30
11 Number of Scans	32
12 Receiver Gain	144
13 Relaxation Delay	1.0000
14 Pulse Width	8.2000
15 Acquisition Time	2.3243
16 Acquisition Date	2023-01-24T15:13:36
17 Modification Date	2023-01-24T15:13:37
18 Spectrometer Frequency	700.50
19 Spectral Width	14097.7
20 Lowest Frequency	-2741.5
21 Nucleus	1H
22 Acquired Size	32768
23 Spectral Size	65536

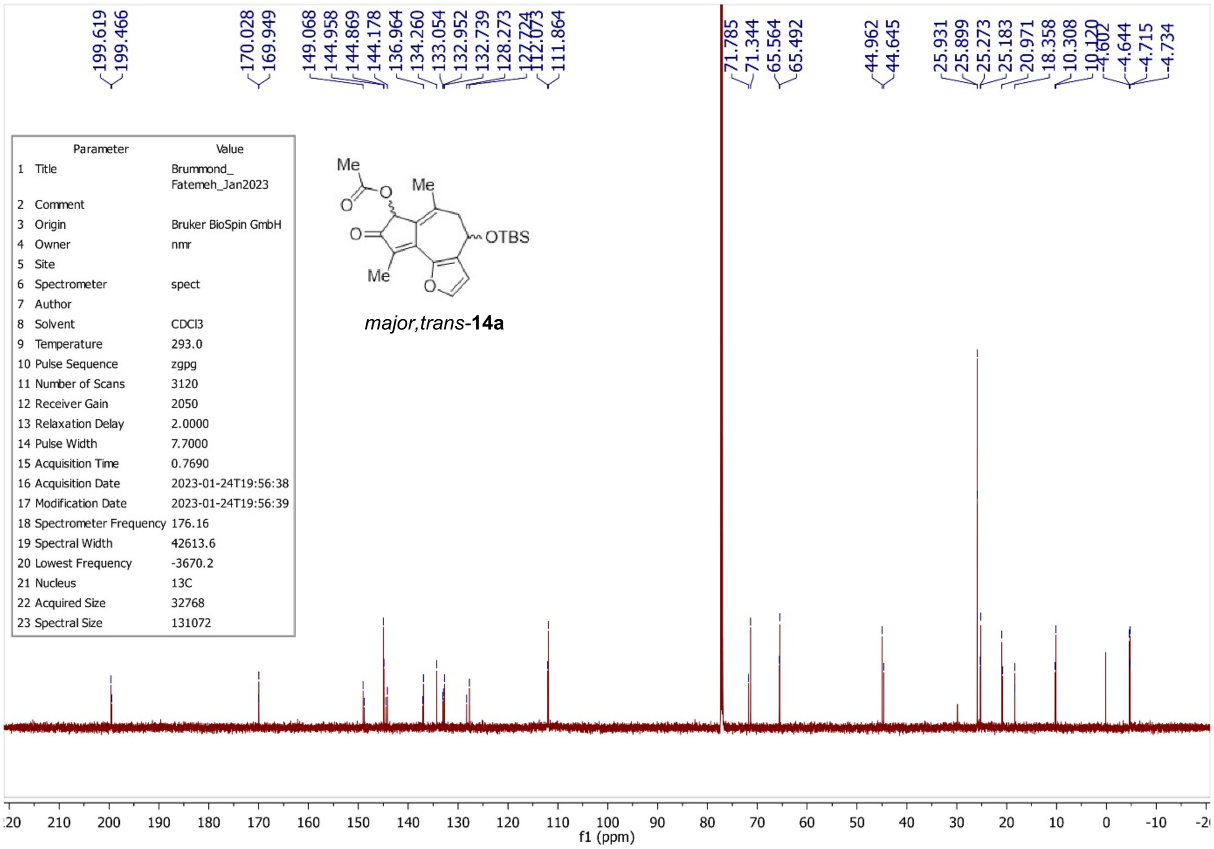

199.619
199.466
170.028
169.949
149.068
144.958
144.869
144.178
136.964
134.260
133.054
132.952
132.739
128.273
127.724
112.073
111.864
71.785
71.344
65.564
65.492
44.962
44.645
25.931
25.899
25.273
25.183
20.971
18.358
10.308
10.120
-4.602
-4.644
-4.715
-4.734
Parameter Value
1 Title Brummond_ Fatemeh_Jan2023
2 Comment
3 Origin Bruker BioSpin GmbH
4 Owner nmr
5 Site
6 Spectrometer spect
7 Author
8 Solvent CDCl3
9 Temperature 293.0
10 Pulse Sequence zgpg
11 Number of Scans 3120
12 Receiver Gain 2050
13 Relaxation Delay 2.0000
14 Pulse Width 7.7000
15 Acquisition Time 0.7690
16 Acquisition Date 2023-01-24T19:56:38
17 Modification Date 2023-01-24T19:56:39
18 Spectrometer Frequency 176.16
19 Spectral Width 42613.6
20 Lowest Frequency -3670.2
21 Nucleus 13C
22 Acquired Size 32768
23 Spectral Size 131072
Me
O
O
Me
O
OTBS
Me
O
major,trans-**14a**
f1 (ppm)

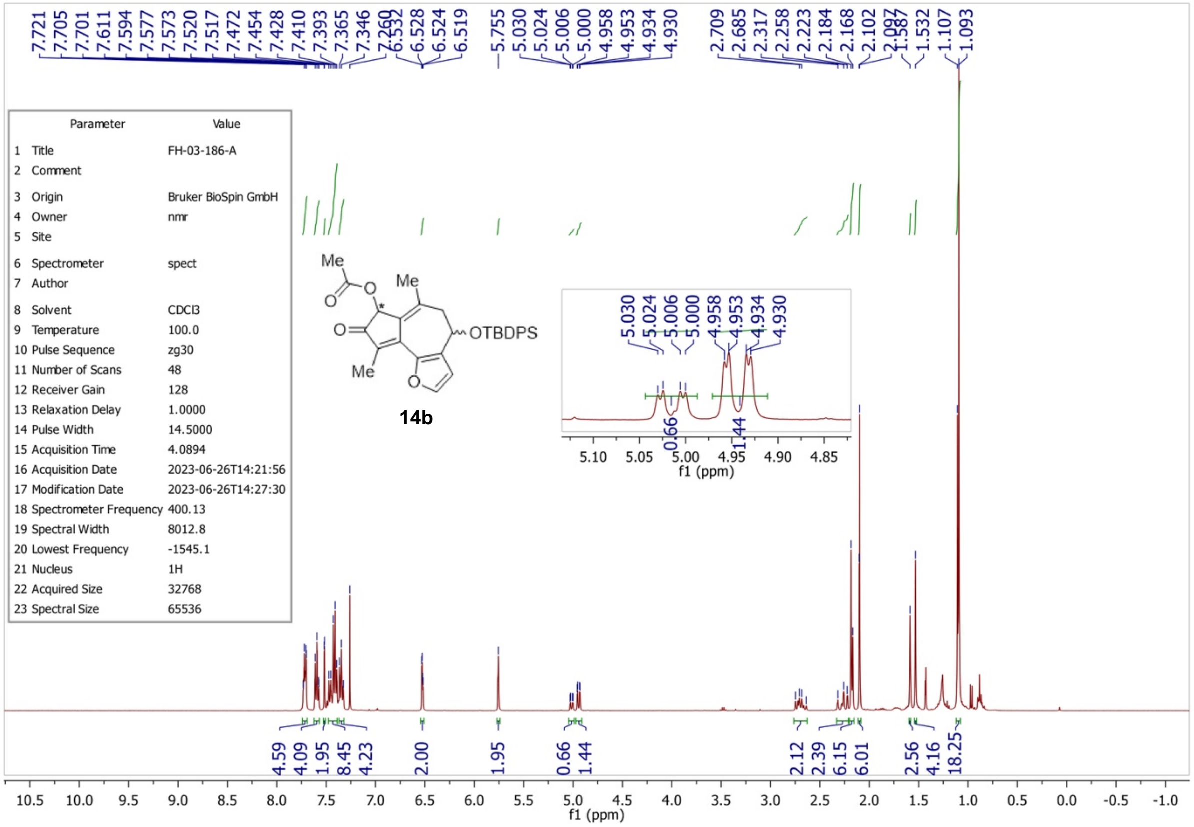
7.721
7.705
7.701
7.611
7.594
7.577
7.573
7.520
7.517
7.472
7.454
7.428
7.410
7.393
7.365
7.346
7.260
6.532
6.528
6.524
6.519
5.755
5.030
5.024
5.006
5.000
4.958
4.953
4.934
4.930
2.709
2.685
2.317
2.258
2.223
2.184
2.168
2.102
2.097
1.587
1.532
1.107
1.093
Parameter
Value
1 Title
FH-03-186-A
2 Comment
3 Origin
Bruker BioSpin GmbH
4 Owner
nmr
5 Site
6 Spectrometer
spect
7 Author
8 Solvent
CDCl3
9 Temperature
100.0
10 Pulse Sequence
zg30
11 Number of Scans
48
12 Receiver Gain
128
13 Relaxation Delay
1.0000
14 Pulse Width
14.5000
15 Acquisition Time
4.0894
16 Acquisition Date
2023-06-26T14:21:56
17 Modification Date
2023-06-26T14:27:30
18 Spectrometer Frequency
400.13
19 Spectral Width
8012.8
20 Lowest Frequency
-1545.1
21 Nucleus
1H
22 Acquired Size
32768
23 Spectral Size
65536
Me
O
O
Me
O
OTBDPS
Me
O
14b
5.030
5.024
5.006
5.000
4.958
4.953
4.934
4.930
0.66
1.44
5.10
5.05
5.00
4.95
4.90
4.85
f1 (ppm)
4.59
4.09
1.95
8.45
4.23
2.00
1.95
0.66
1.44
2.12
2.39
6.15
6.01
2.56
4.16
18.25
10.5
10.0
9.5
9.0
8.5
8.0
7.5
7.0
6.5
6.0
5.5
5.0
4.5
4.0
3.5
3.0
2.5
2.0
1.5
1.0
0.5
0.0
-0.5
-1.0
f1 (ppm)

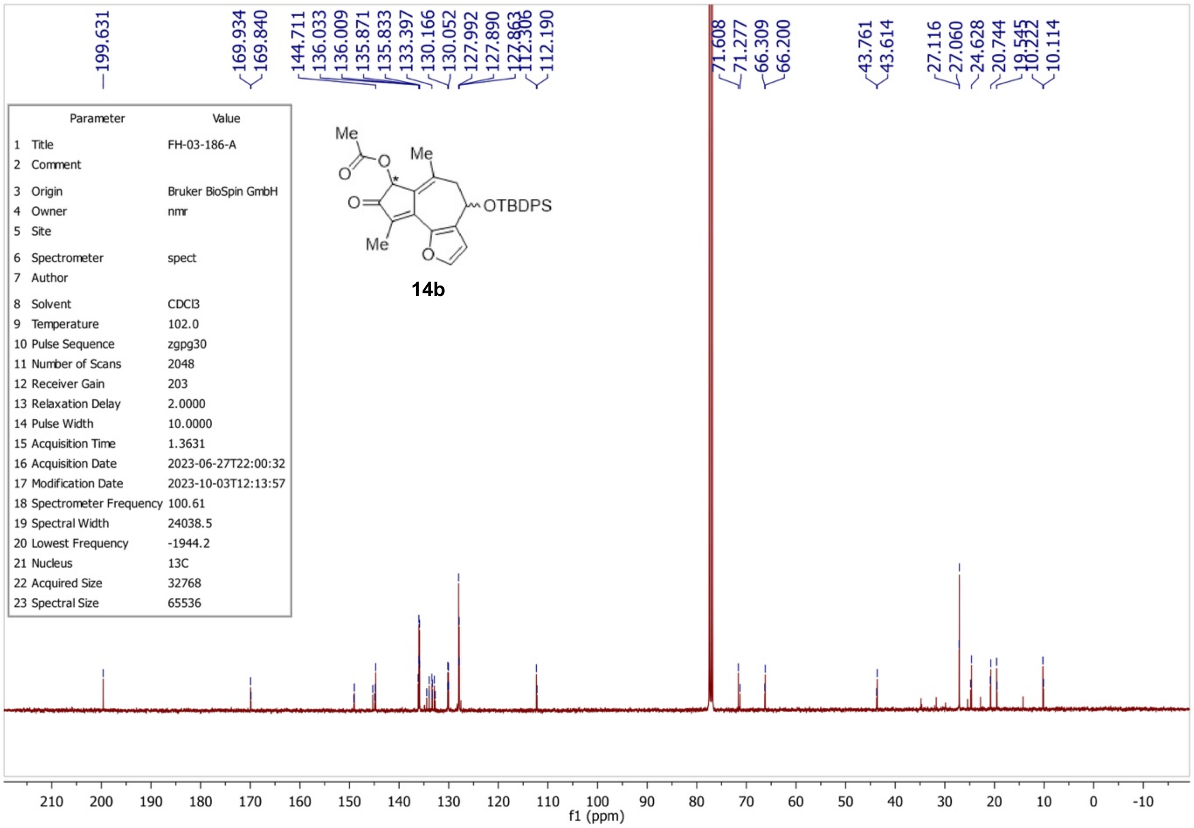

Parameter	Value
1 Title	FH-03-186-A
2 Comment	
3 Origin	Bruker BioSpin GmbH
4 Owner	nmr
5 Site	
6 Spectrometer	spect
7 Author	
8 Solvent	CDCl3
9 Temperature	102.0
10 Pulse Sequence	zgpg30
11 Number of Scans	2048
12 Receiver Gain	203
13 Relaxation Delay	2.0000
14 Pulse Width	10.0000
15 Acquisition Time	1.3631
16 Acquisition Date	2023-06-27T22:00:32
17 Modification Date	2023-10-03T12:13:57
18 Spectrometer Frequency	100.61
19 Spectral Width	24038.5
20 Lowest Frequency	-1944.2
21 Nucleus	13C
22 Acquired Size	32768
23 Spectral Size	65536

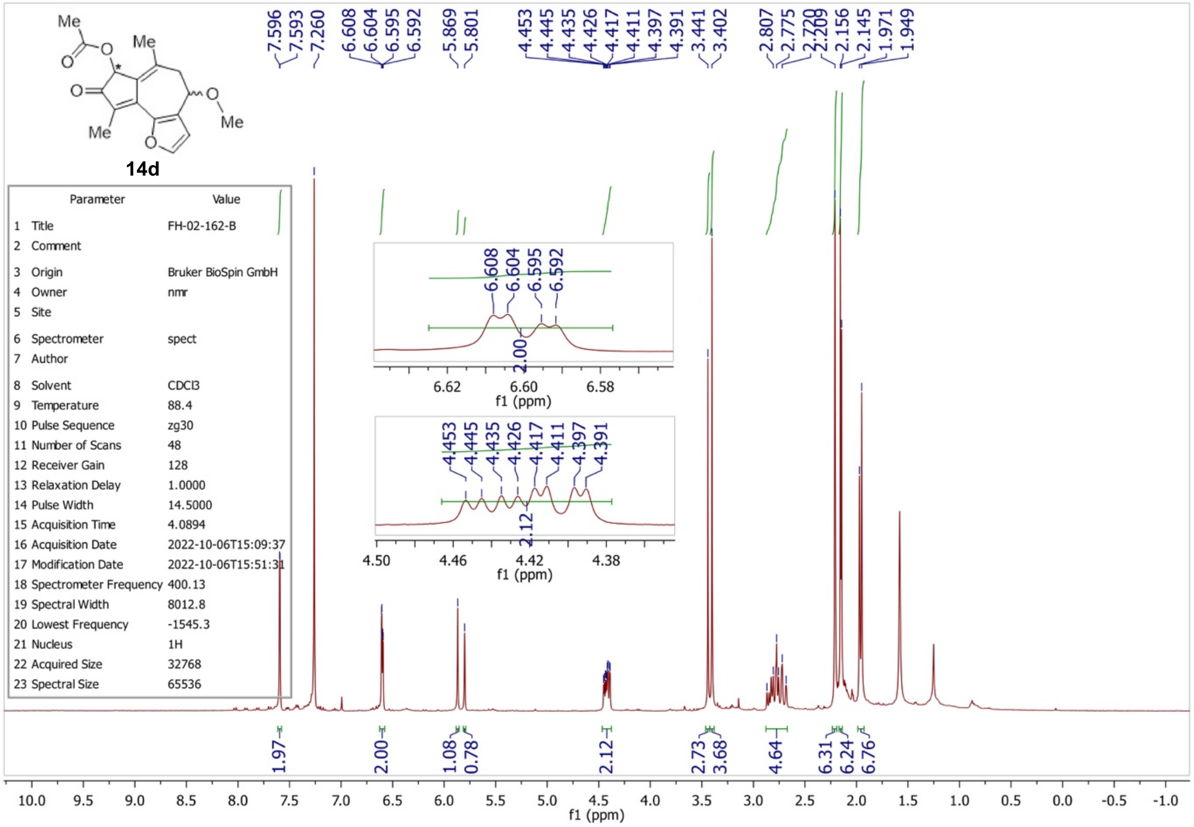

14d
Parameter Value
1 Title FH-02-162-B
2 Comment
3 Origin Bruker BioSpin GmbH
4 Owner nmr
5 Site
6 Spectrometer spect
7 Author
8 Solvent CDCl3
9 Temperature 88.4
10 Pulse Sequence zg30
11 Number of Scans 48
12 Receiver Gain 128
13 Relaxation Delay 1.0000
14 Pulse Width 14.5000
15 Acquisition Time 4.0894
16 Acquisition Date 2022-10-06T15:09:37
17 Modification Date 2022-10-06T15:51:31
18 Spectrometer Frequency 400.13
19 Spectral Width 8012.8
20 Lowest Frequency -1545.3
21 Nucleus 1H
22 Acquired Size 32768
23 Spectral Size 65536
f1 (ppm)

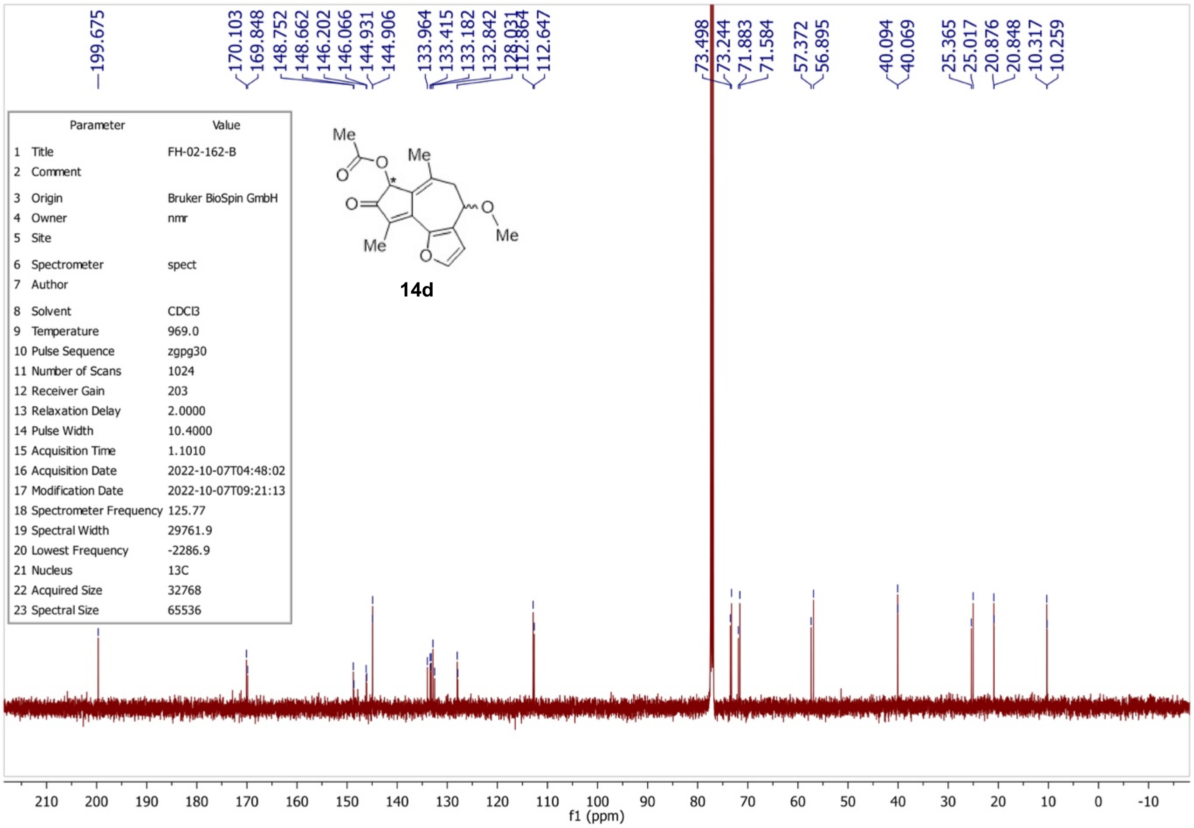
199.675
170.103
169.848
148.752
148.662
146.202
146.066
144.931
144.906
133.964
133.415
133.182
132.842
128.031
112.864
112.647
73.498
73.244
71.883
71.584
57.372
56.895
40.094
40.069
25.365
25.017
20.876
20.848
10.317
10.259
Parameter Value
1 Title FH-02-162-B
2 Comment
3 Origin Bruker BioSpin GmbH
4 Owner nmr
5 Site
6 Spectrometer spect
7 Author
8 Solvent CDCl3
9 Temperature 969.0
10 Pulse Sequence zgpg30
11 Number of Scans 1024
12 Receiver Gain 203
13 Relaxation Delay 2.0000
14 Pulse Width 10.4000
15 Acquisition Time 1.1010
16 Acquisition Date 2022-10-07T04:48:02
17 Modification Date 2022-10-07T09:21:13
18 Spectrometer Frequency 125.77
19 Spectral Width 29761.9
20 Lowest Frequency -2286.9
21 Nucleus 13C
22 Acquired Size 32768
23 Spectral Size 65536
Me
O
O
Me
O
O
Me
Me
O
14d
f1 (ppm)

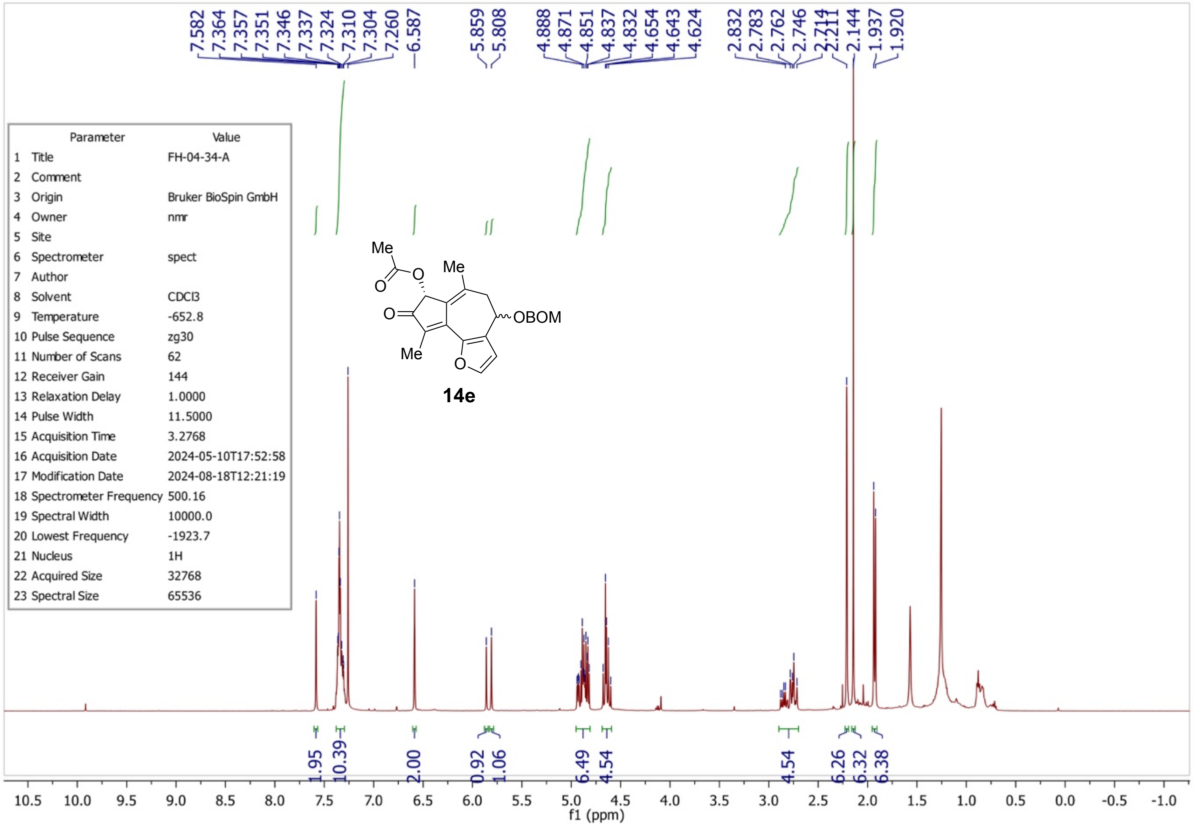

7.582
7.364
7.357
7.351
7.346
7.337
7.324
7.310
7.304
7.260
6.587
5.859
5.808
4.888
4.871
4.851
4.837
4.832
4.654
4.643
4.624
2.832
2.783
2.762
2.746
2.714
2.211
2.144
1.937
1.920
Parameter | Value
1 Title | FH-04-34-A
2 Comment
3 Origin | Bruker BioSpin GmbH
4 Owner | nmr
5 Site
6 Spectrometer | spect
7 Author
8 Solvent | CDCl3
9 Temperature | -652.8
10 Pulse Sequence | zg30
11 Number of Scans | 62
12 Receiver Gain | 144
13 Relaxation Delay | 1.0000
14 Pulse Width | 11.5000
15 Acquisition Time | 3.2768
16 Acquisition Date | 2024-05-10T17:52:58
17 Modification Date | 2024-08-18T12:21:19
18 Spectrometer Frequency | 500.16
19 Spectral Width | 10000.0
20 Lowest Frequency | -1923.7
21 Nucleus | 1H
22 Acquired Size | 32768
23 Spectral Size | 65536
Me
O
O
Me
O
OBOM
Me
O
14e
1.95
10.39
2.00
0.92
1.06
6.49
4.54
4.54
6.26
6.32
6.38
10.5 10.0 9.5 9.0 8.5 8.0 7.5 7.0 6.5 6.0 5.5 5.0 4.5 4.0 3.5 3.0 2.5 2.0 1.5 1.0 0.5 0.0 -0.5 -1.0
f1 (ppm)

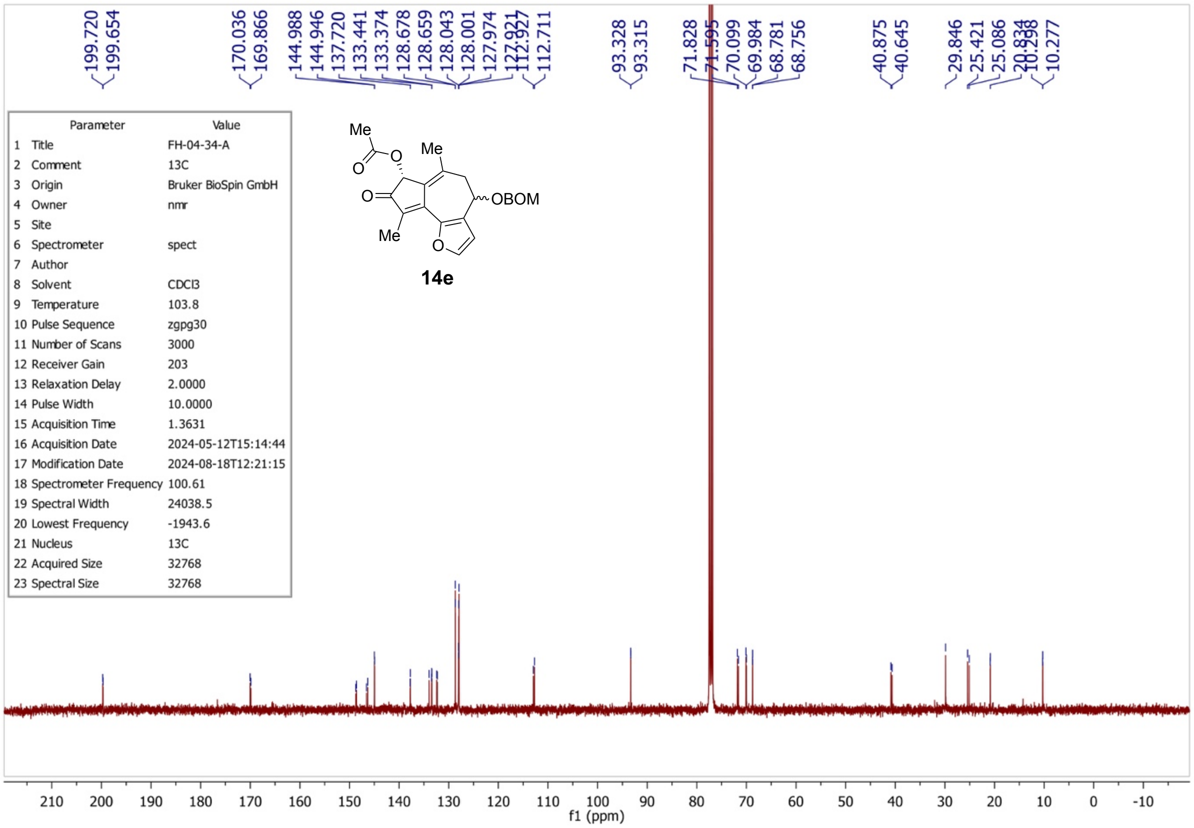

199.720
199.654
170.036
169.866
144.988
144.946
137.720
133.441
133.374
128.678
128.659
128.043
128.001
127.974
127.921
112.927
112.711
93.328
93.315
71.828
71.595
70.099
69.984
68.781
68.756
40.875
40.645
29.846
25.421
25.086
20.834
10.298
10.277
Parameter Value
1 Title FH-04-34-A
2 Comment 13C
3 Origin Bruker BioSpin GmbH
4 Owner nmr
5 Site
6 Spectrometer spect
7 Author
8 Solvent CDCl3
9 Temperature 103.8
10 Pulse Sequence zgpg30
11 Number of Scans 3000
12 Receiver Gain 203
13 Relaxation Delay 2.0000
14 Pulse Width 10.0000
15 Acquisition Time 1.3631
16 Acquisition Date 2024-05-12T15:14:44
17 Modification Date 2024-08-18T12:21:15
18 Spectrometer Frequency 100.61
19 Spectral Width 24038.5
20 Lowest Frequency -1943.6
21 Nucleus 13C
22 Acquired Size 32768
23 Spectral Size 32768
Me
O
O
Me
O
OBOM
Me
O
14e
f1 (ppm)

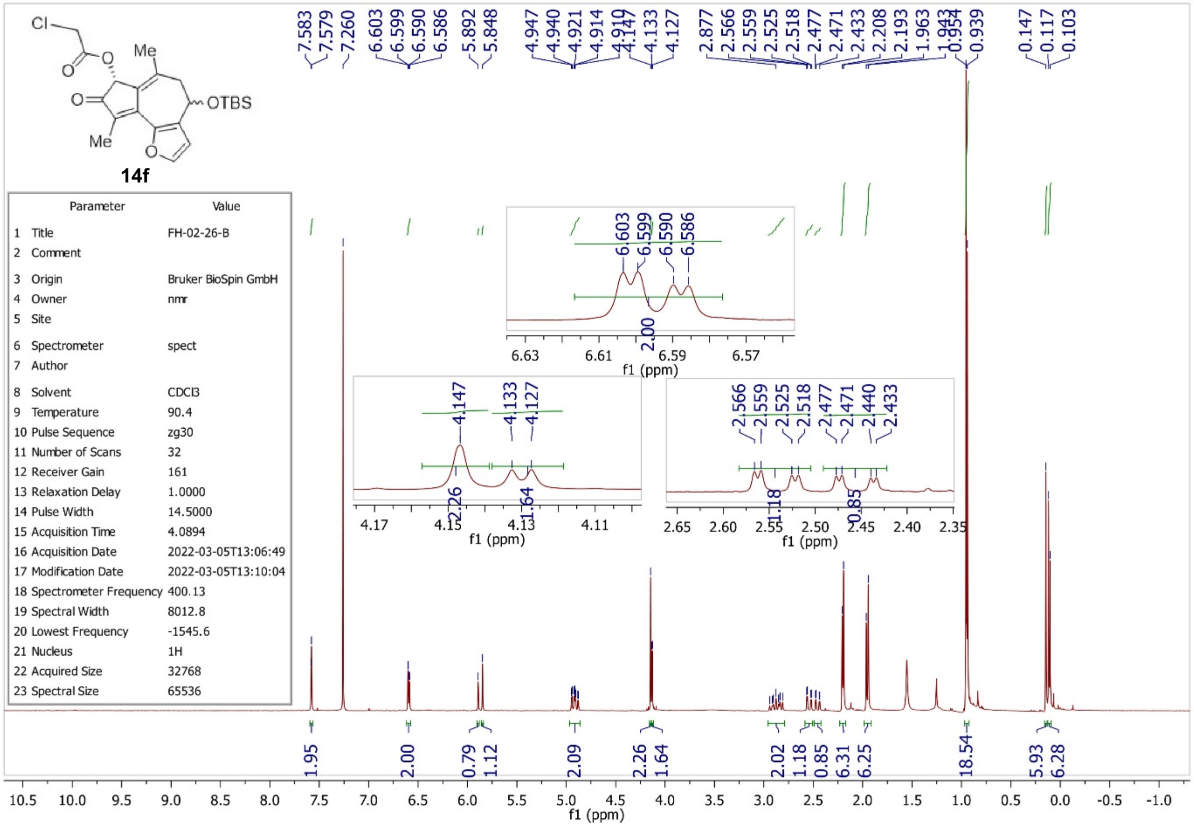

Parameter	Value
1 Title	FH-02-26-B
2 Comment	
3 Origin	Bruker BioSpin GmbH
4 Owner	nmr
5 Site	
6 Spectrometer	spect
7 Author	
8 Solvent	CDCl3
9 Temperature	90.4
10 Pulse Sequence	zg30
11 Number of Scans	32
12 Receiver Gain	161
13 Relaxation Delay	1.0000
14 Pulse Width	14.5000
15 Acquisition Time	4.0894
16 Acquisition Date	2022-03-05T13:06:49
17 Modification Date	2022-03-05T13:10:04
18 Spectrometer Frequency	400.13
19 Spectral Width	8012.8
20 Lowest Frequency	-1545.6
21 Nucleus	1H
22 Acquired Size	32768
23 Spectral Size	65536

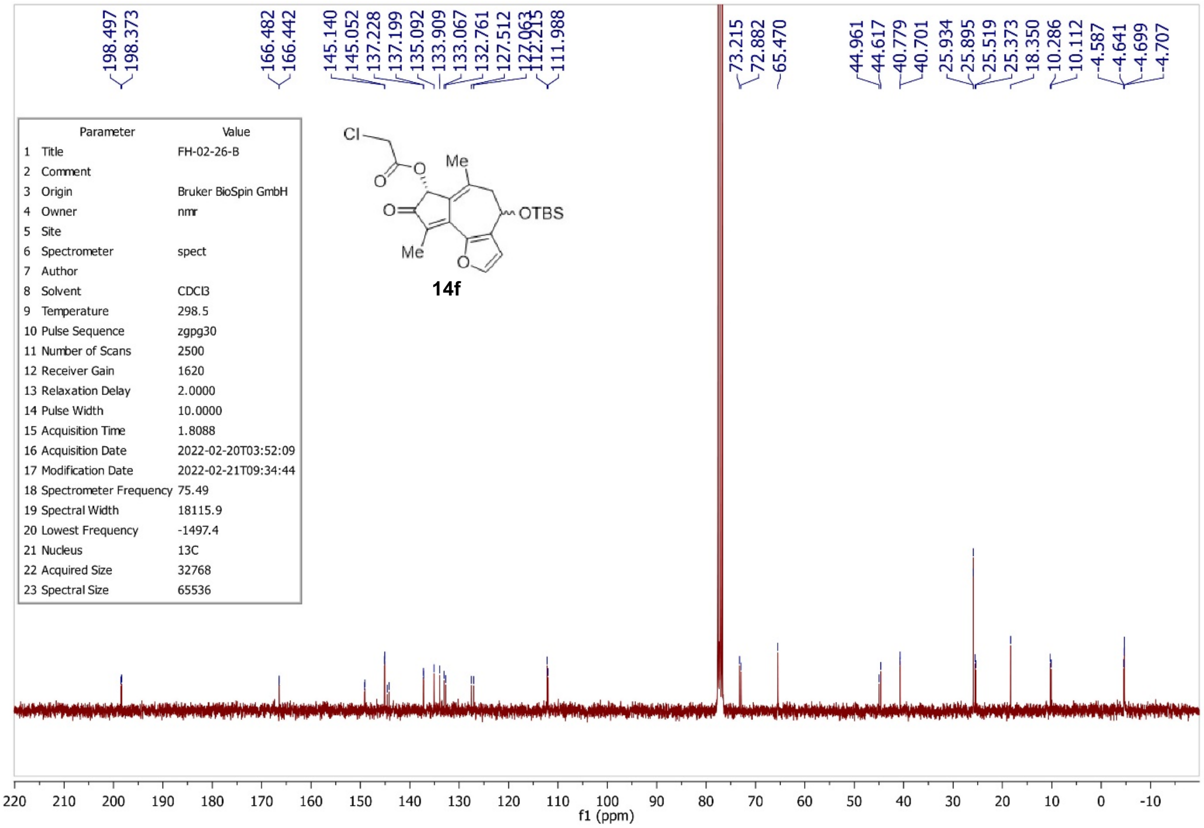

198.497
198.373
166.482
166.442
145.140
145.052
137.228
137.199
135.092
133.909
133.067
132.761
127.512
127.063
112.215
111.988
73.215
72.882
65.470
44.961
44.617
40.779
40.701
25.934
25.895
25.519
25.373
18.350
10.286
10.112
-4.587
-4.641
-4.699
-4.707
Parameter Value
1 Title FH-02-26-B
2 Comment
3 Origin Bruker BioSpin GmbH
4 Owner nmr
5 Site
6 Spectrometer spect
7 Author
8 Solvent CDCl3
9 Temperature 298.5
10 Pulse Sequence zgpg30
11 Number of Scans 2500
12 Receiver Gain 1620
13 Relaxation Delay 2.0000
14 Pulse Width 10.0000
15 Acquisition Time 1.8088
16 Acquisition Date 2022-02-20T03:52:09
17 Modification Date 2022-02-21T09:34:44
18 Spectrometer Frequency 75.49
19 Spectral Width 18115.9
20 Lowest Frequency -1497.4
21 Nucleus 13C
22 Acquired Size 32768
23 Spectral Size 65536
Cl
O
O
Me
OTBS
O
Me
O
14f
220 210 200 190 180 170 160 150 140 130 120 110 100 90 80 70 60 50 40 30 20 10 0 -10
f1 (ppm)

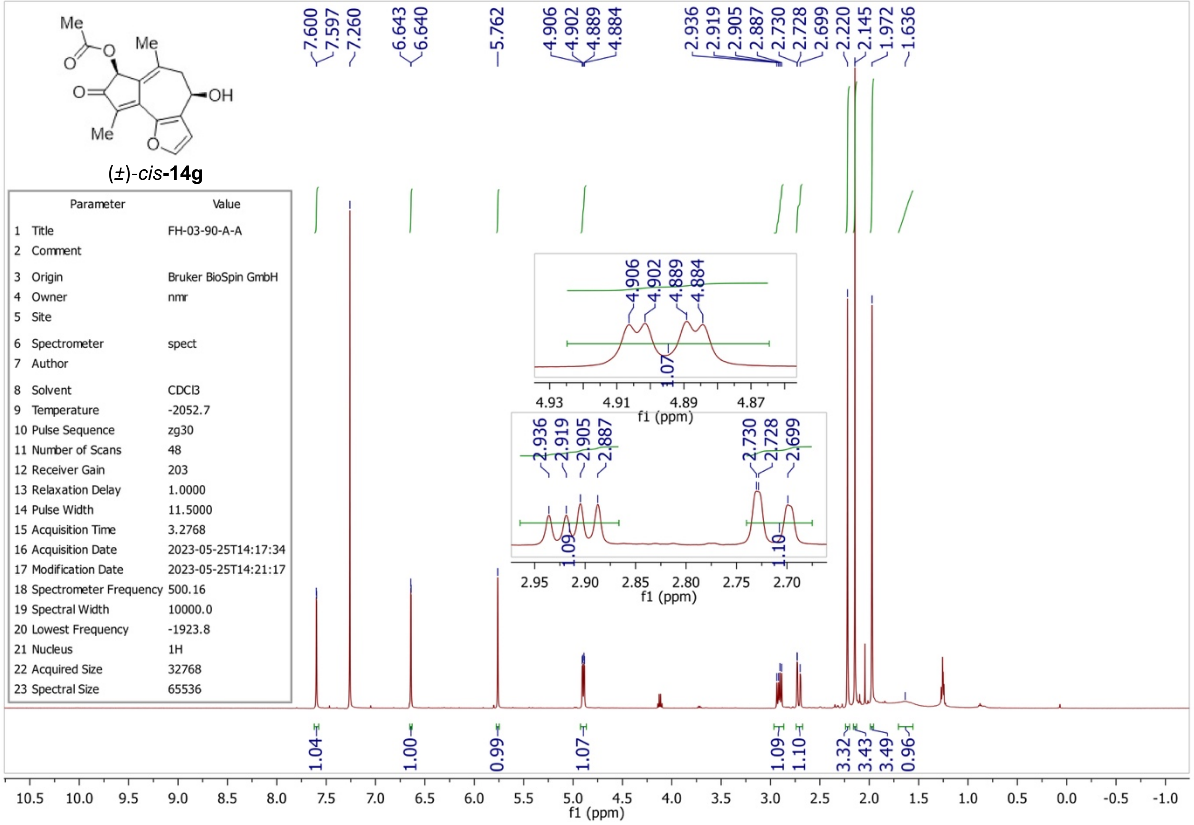
(±)-cis-14g
Me
O
O
Me
O
OH
Me
O
7.600
7.597
7.260
6.643
6.640
5.762
4.906
4.902
4.889
4.884
2.936
2.919
2.905
2.887
2.730
2.728
2.699
2.220
2.145
1.972
1.636
Parameter | Value
1 Title | FH-03-90-A-A
2 Comment
3 Origin | Bruker BioSpin GmbH
4 Owner | nmr
5 Site
6 Spectrometer | spect
7 Author
8 Solvent | CDCl3
9 Temperature | -2052.7
10 Pulse Sequence | zg30
11 Number of Scans | 48
12 Receiver Gain | 203
13 Relaxation Delay | 1.0000
14 Pulse Width | 11.5000
15 Acquisition Time | 3.2768
16 Acquisition Date | 2023-05-25T14:17:34
17 Modification Date | 2023-05-25T14:21:17
18 Spectrometer Frequency | 500.16
19 Spectral Width | 10000.0
20 Lowest Frequency | -1923.8
21 Nucleus | 1H
22 Acquired Size | 32768
23 Spectral Size | 65536
1.07
f1 (ppm)
1.09
1.10
f1 (ppm)
1.04
1.00
0.99
1.07
1.09
1.10
3.32
3.43
3.49
0.96
f1 (ppm)

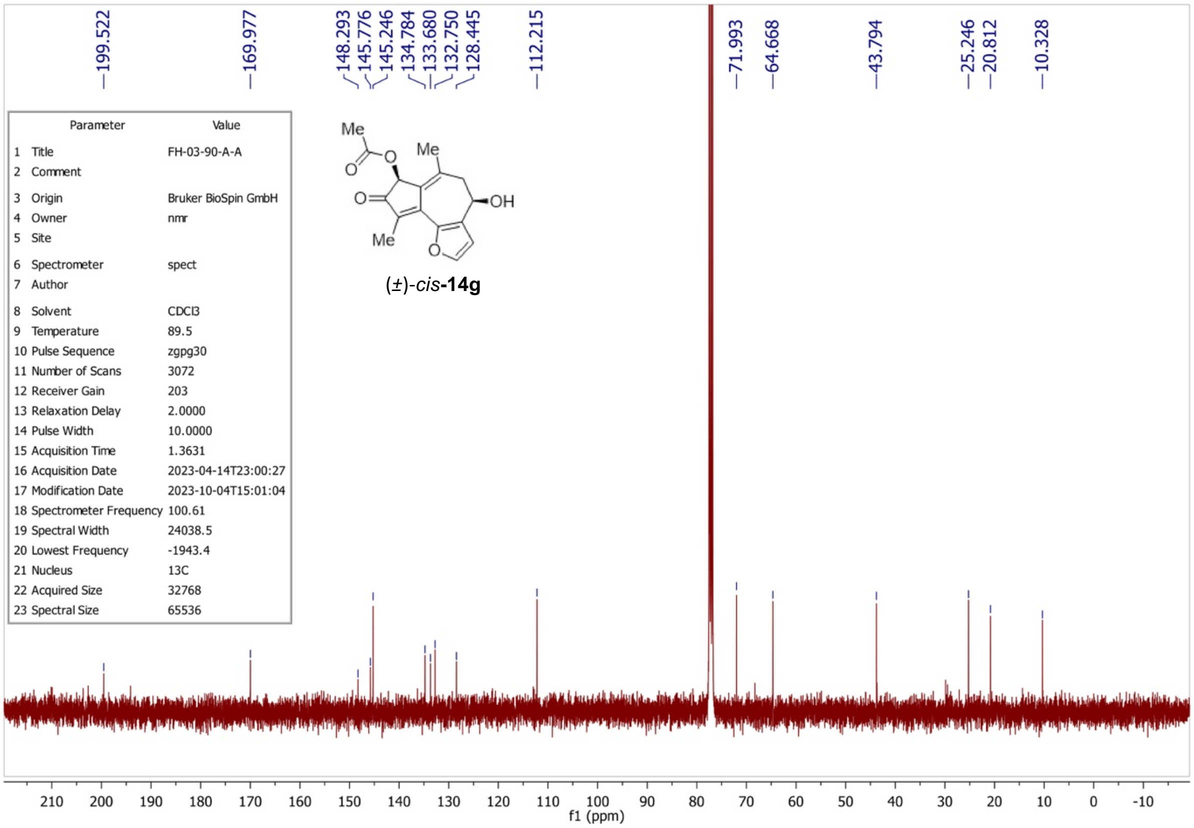

199.522
169.977
148.293
145.776
145.246
134.784
133.680
132.750
128.445
112.215
71.993
64.668
43.794
25.246
20.812
10.328
Parameter Value
1 Title FH-03-90-A-A
2 Comment
3 Origin Bruker BioSpin GmbH
4 Owner nmr
5 Site
6 Spectrometer spect
7 Author
8 Solvent CDCl3
9 Temperature 89.5
10 Pulse Sequence zgpg30
11 Number of Scans 3072
12 Receiver Gain 203
13 Relaxation Delay 2.0000
14 Pulse Width 10.0000
15 Acquisition Time 1.3631
16 Acquisition Date 2023-04-14T23:00:27
17 Modification Date 2023-10-04T15:01:04
18 Spectrometer Frequency 100.61
19 Spectral Width 24038.5
20 Lowest Frequency -1943.4
21 Nucleus 13C
22 Acquired Size 32768
23 Spectral Size 65536
Me
O
O
Me
O
OH
Me
O
(±)-cis-14g
f1 (ppm)

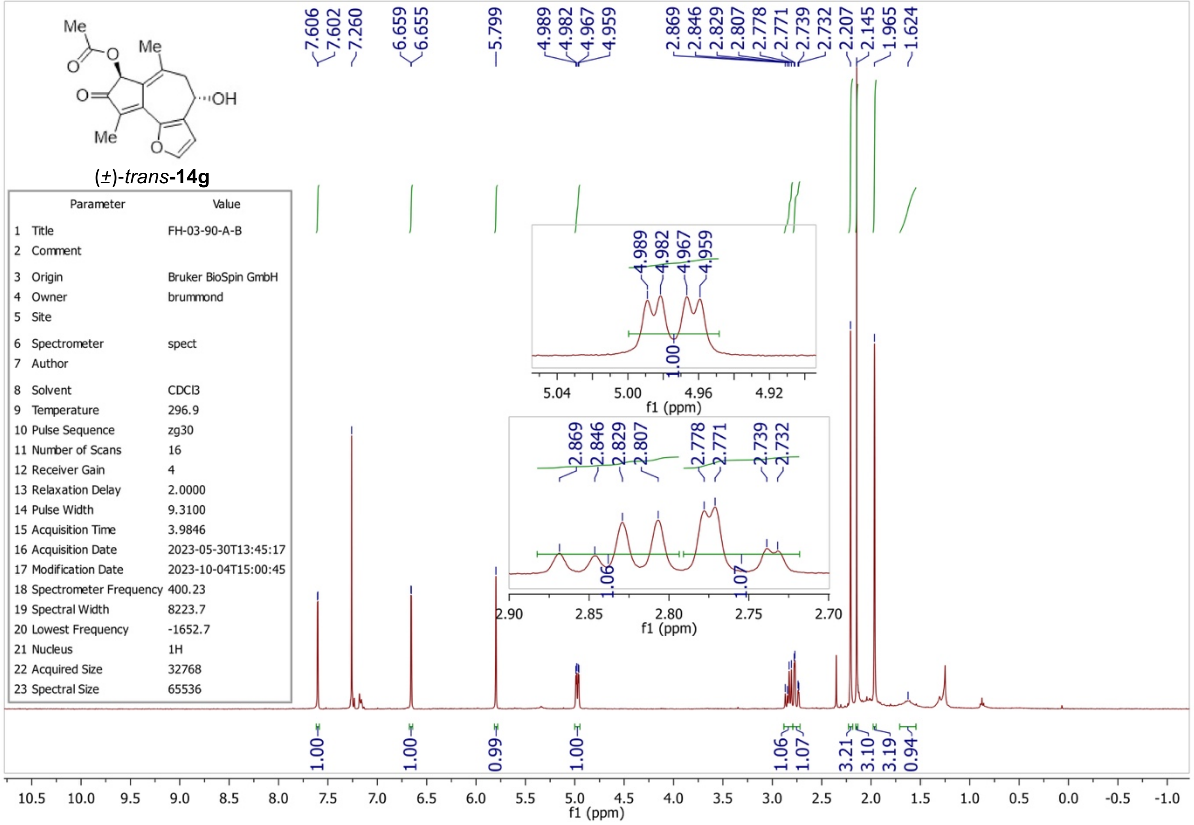

Me
Me
O
O
O
OH
Me
O
(±)-trans-14g
Parameter Value
1 Title FH-03-90-A-B
2 Comment
3 Origin Bruker BioSpin GmbH
4 Owner brummond
5 Site
6 Spectrometer spect
7 Author
8 Solvent CDCl3
9 Temperature 296.9
10 Pulse Sequence zg30
11 Number of Scans 16
12 Receiver Gain 4
13 Relaxation Delay 2.0000
14 Pulse Width 9.3100
15 Acquisition Time 3.9846
16 Acquisition Date 2023-05-30T13:45:17
17 Modification Date 2023-10-04T15:00:45
18 Spectrometer Frequency 400.23
19 Spectral Width 8223.7
20 Lowest Frequency -1652.7
21 Nucleus 1H
22 Acquired Size 32768
23 Spectral Size 65536
7.606 7.602 7.260 6.659 6.655 5.799 4.989 4.982 4.967 4.959 2.869 2.846 2.829 2.807 2.778 2.771 2.739 2.732 2.207 2.145 1.965 1.624
1.00 1.00 0.99 1.00 1.06 1.07 3.21 3.10 3.19 0.94
f1 (ppm)

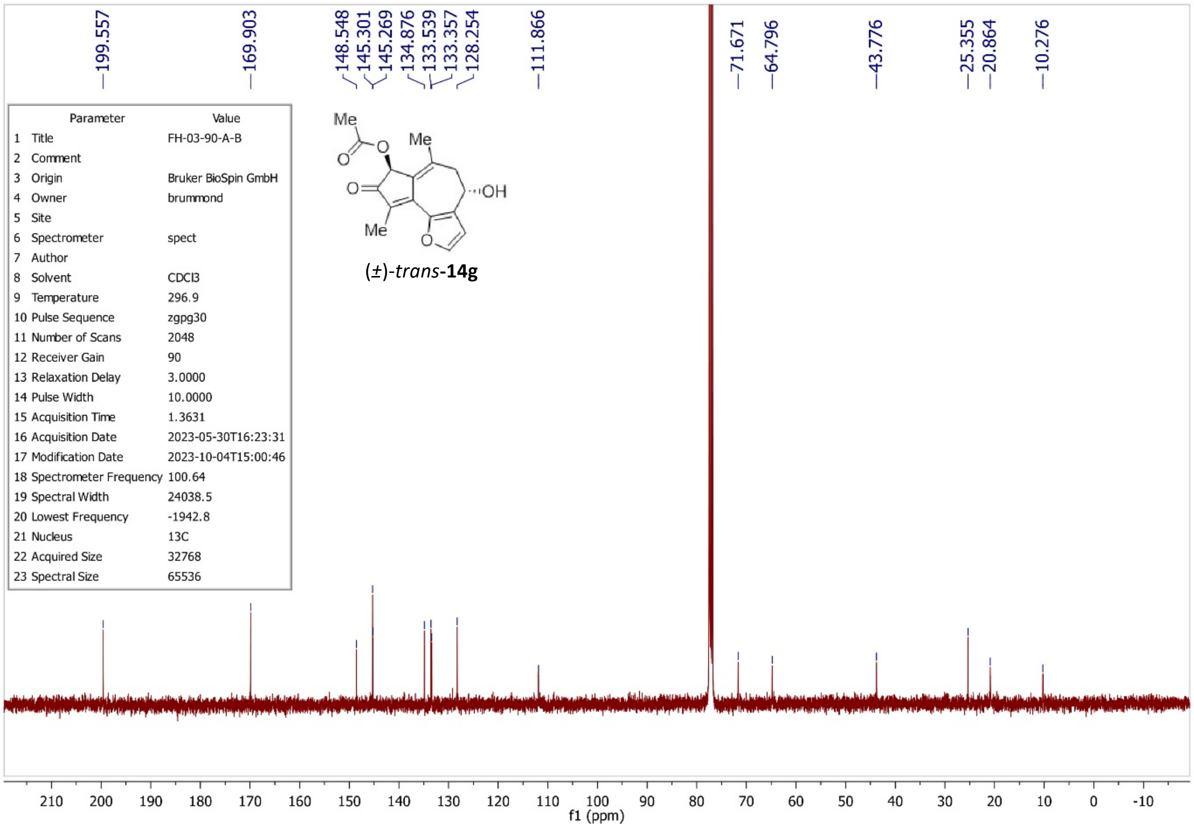
199.557
169.903
148.548
145.301
145.269
134.876
133.539
133.357
128.254
111.866
71.671
64.796
43.776
25.355
20.864
10.276
Parameter Value
1 Title FH-03-90-A-B
2 Comment
3 Origin Bruker BioSpin GmbH
4 Owner brummond
5 Site
6 Spectrometer spect
7 Author
8 Solvent CDCl3
9 Temperature 296.9
10 Pulse Sequence zgpg30
11 Number of Scans 2048
12 Receiver Gain 90
13 Relaxation Delay 3.0000
14 Pulse Width 10.0000
15 Acquisition Time 1.3631
16 Acquisition Date 2023-05-30T16:23:31
17 Modification Date 2023-10-04T15:00:46
18 Spectrometer Frequency 100.64
19 Spectral Width 24038.5
20 Lowest Frequency -1942.8
21 Nucleus 13C
22 Acquired Size 32768
23 Spectral Size 65536
Me
O
O
Me
O
OH
Me
O
(±)-*trans*-**14g**
210 200 190 180 170 160 150 140 130 120 110 100 90 80 70 60 50 40 30 20 10 0 -10
f1 (ppm)

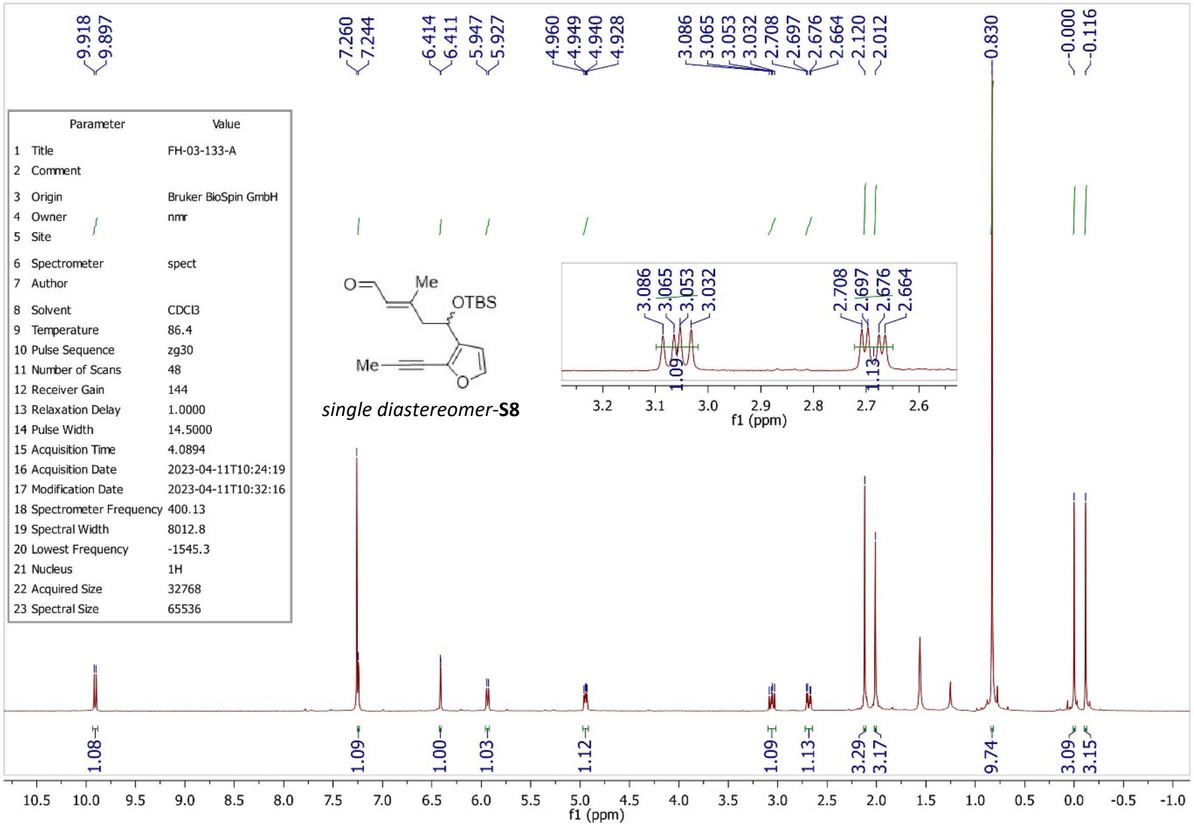
9.918
9.897
7.260
7.244
6.414
6.411
5.947
5.927
4.960
4.949
4.940
4.928
3.086
3.065
3.053
3.032
2.708
2.697
2.676
2.664
2.120
2.012
0.830
-0.000
-0.116
Parameter Value
1 Title FH-03-133-A
2 Comment
3 Origin Bruker BioSpin GmbH
4 Owner nmr
5 Site
6 Spectrometer spect
7 Author
8 Solvent CDCl3
9 Temperature 86.4
10 Pulse Sequence zg30
11 Number of Scans 48
12 Receiver Gain 144
13 Relaxation Delay 1.0000
14 Pulse Width 14.5000
15 Acquisition Time 4.0894
16 Acquisition Date 2023-04-11T10:24:19
17 Modification Date 2023-04-11T10:32:16
18 Spectrometer Frequency 400.13
19 Spectral Width 8012.8
20 Lowest Frequency -1545.3
21 Nucleus 1H
22 Acquired Size 32768
23 Spectral Size 65536
O
Me
OTBS
Me
O
single diastereomer-S8
1.09
1.13
f1 (ppm)
1.08
1.09
1.00
1.03
1.12
1.09
1.13
3.29
3.17
9.74
3.09
3.15
f1 (ppm)

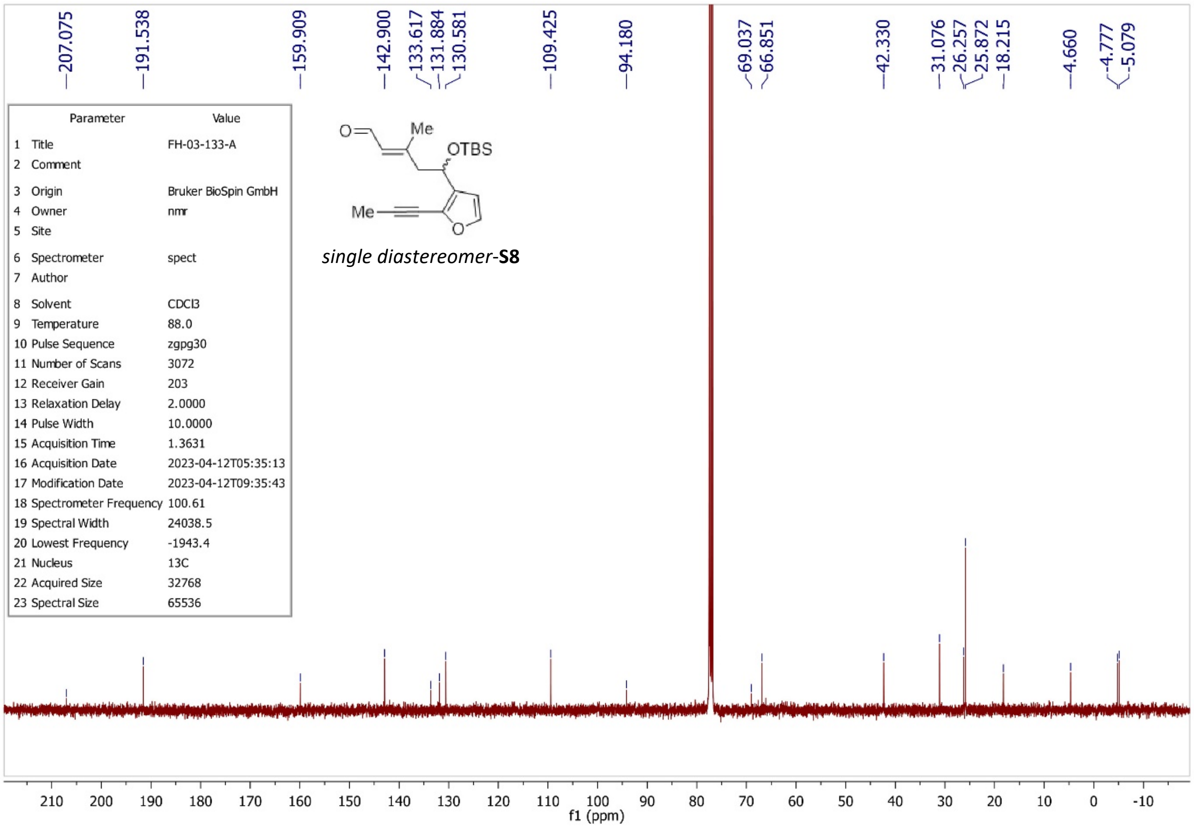

207.075
191.538
159.909
142.900
133.617
131.884
130.581
109.425
94.180
69.037
66.851
42.330
31.076
26.257
25.872
18.215
4.660
-4.777
-5.079
Parameter
Value
1 Title FH-03-133-A
2 Comment
3 Origin Bruker BioSpin GmbH
4 Owner nmr
5 Site
6 Spectrometer spect
7 Author
8 Solvent CDCl3
9 Temperature 88.0
10 Pulse Sequence zgpg30
11 Number of Scans 3072
12 Receiver Gain 203
13 Relaxation Delay 2.0000
14 Pulse Width 10.0000
15 Acquisition Time 1.3631
16 Acquisition Date 2023-04-12T05:35:13
17 Modification Date 2023-04-12T09:35:43
18 Spectrometer Frequency 100.61
19 Spectral Width 24038.5
20 Lowest Frequency -1943.4
21 Nucleus 13C
22 Acquired Size 32768
23 Spectral Size 65536
O
Me
OTBS
Me
O
single diastereomer-**S8**
f1 (ppm)

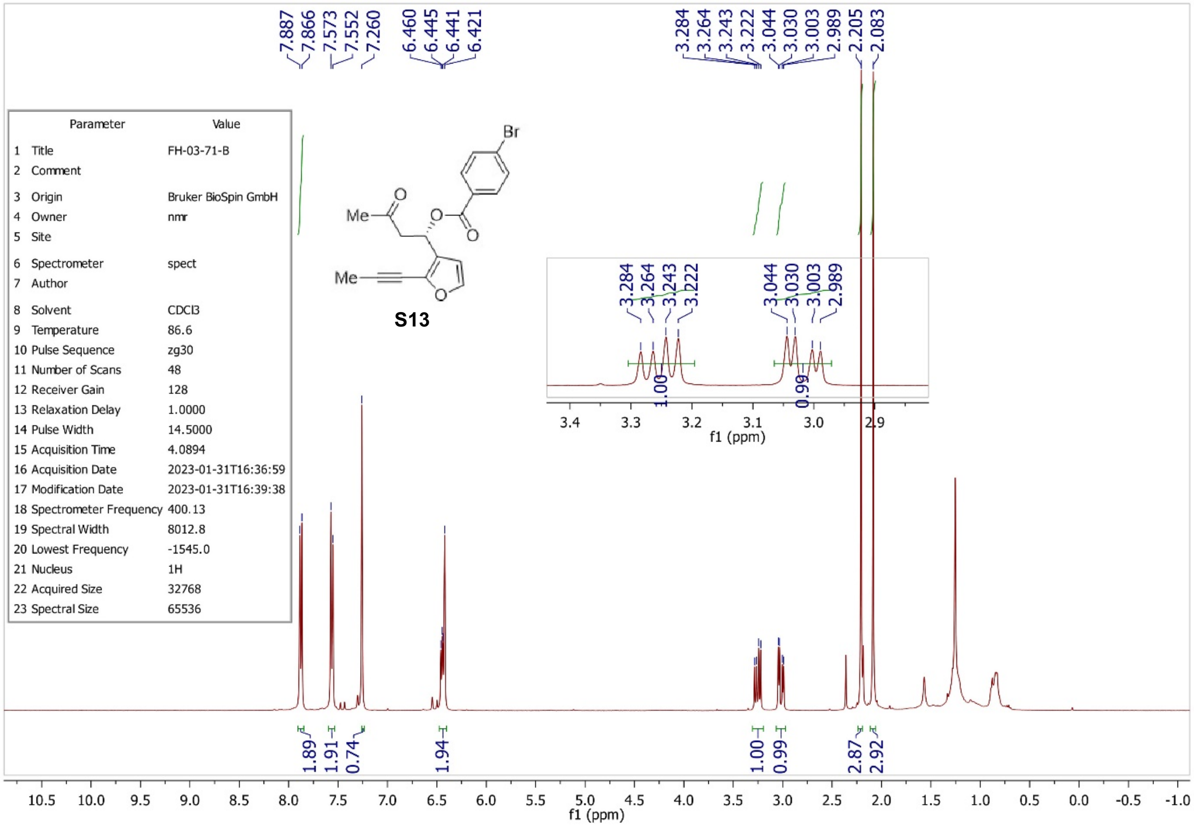
7.887
7.866
7.573
7.552
7.260
6.460
6.445
6.441
6.421
3.284
3.264
3.243
3.222
3.044
3.030
3.003
2.989
2.205
2.083
Parameter
Value
1 Title
FH-03-71-B
2 Comment
3 Origin
Bruker BioSpin GmbH
4 Owner
nmr
5 Site
6 Spectrometer
spect
7 Author
8 Solvent
CDCl3
9 Temperature
86.6
10 Pulse Sequence
zg30
11 Number of Scans
48
12 Receiver Gain
128
13 Relaxation Delay
1.0000
14 Pulse Width
14.5000
15 Acquisition Time
4.0894
16 Acquisition Date
2023-01-31T16:36:59
17 Modification Date
2023-01-31T16:39:38
18 Spectrometer Frequency
400.13
19 Spectral Width
8012.8
20 Lowest Frequency
-1545.0
21 Nucleus
1H
22 Acquired Size
32768
23 Spectral Size
65536
Br
Me
O
O
O
Me
O
S13
1.00
0.99
f1 (ppm)
1.89
1.91
0.74
1.94
1.00
0.99
2.87
2.92
f1 (ppm)

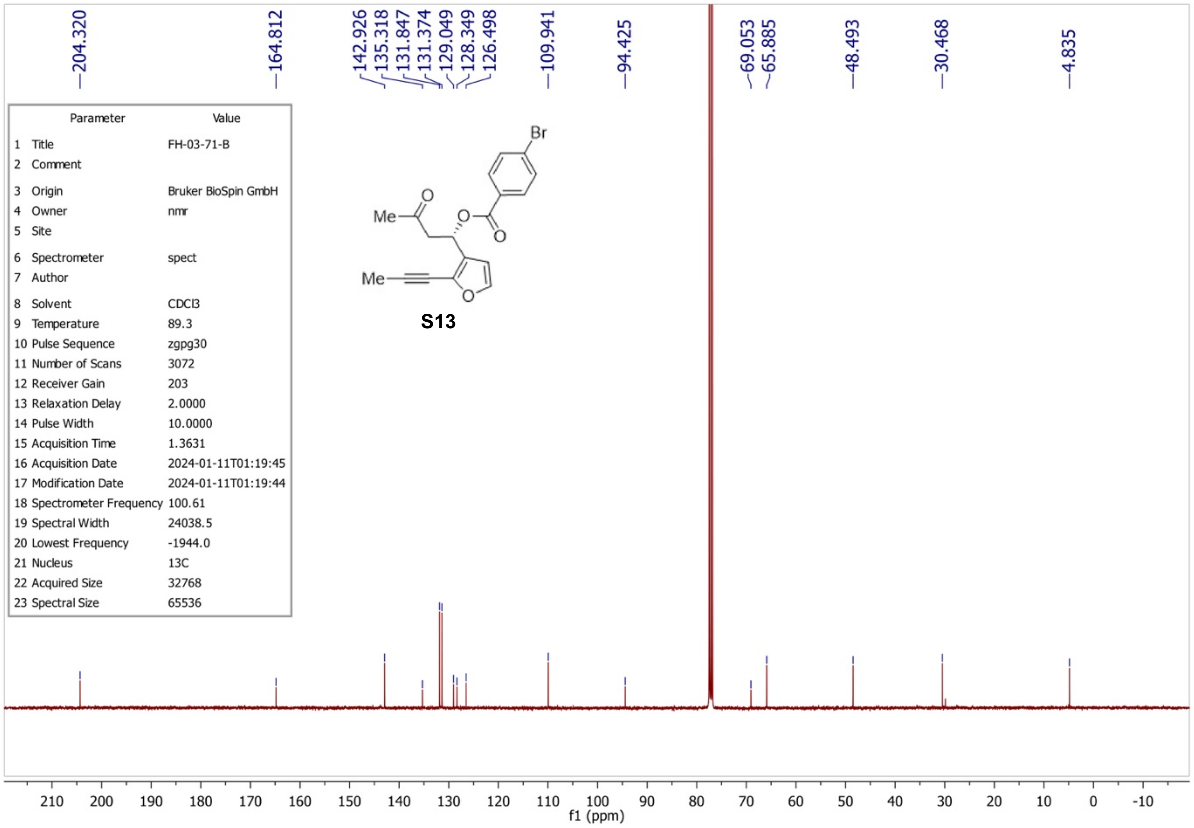

204.320
164.812
142.926
135.318
131.847
131.374
129.049
128.349
126.498
109.941
94.425
69.053
65.885
48.493
30.468
4.835
Parameter
Value
1 Title FH-03-71-B
2 Comment
3 Origin Bruker BioSpin GmbH
4 Owner nmr
5 Site
6 Spectrometer spect
7 Author
8 Solvent CDCl3
9 Temperature 89.3
10 Pulse Sequence zgpg30
11 Number of Scans 3072
12 Receiver Gain 203
13 Relaxation Delay 2.0000
14 Pulse Width 10.0000
15 Acquisition Time 1.3631
16 Acquisition Date 2024-01-11T01:19:45
17 Modification Date 2024-01-11T01:19:44
18 Spectrometer Frequency 100.61
19 Spectral Width 24038.5
20 Lowest Frequency -1944.0
21 Nucleus 13C
22 Acquired Size 32768
23 Spectral Size 65536
Br
Me
O
Me
S13
f1 (ppm)

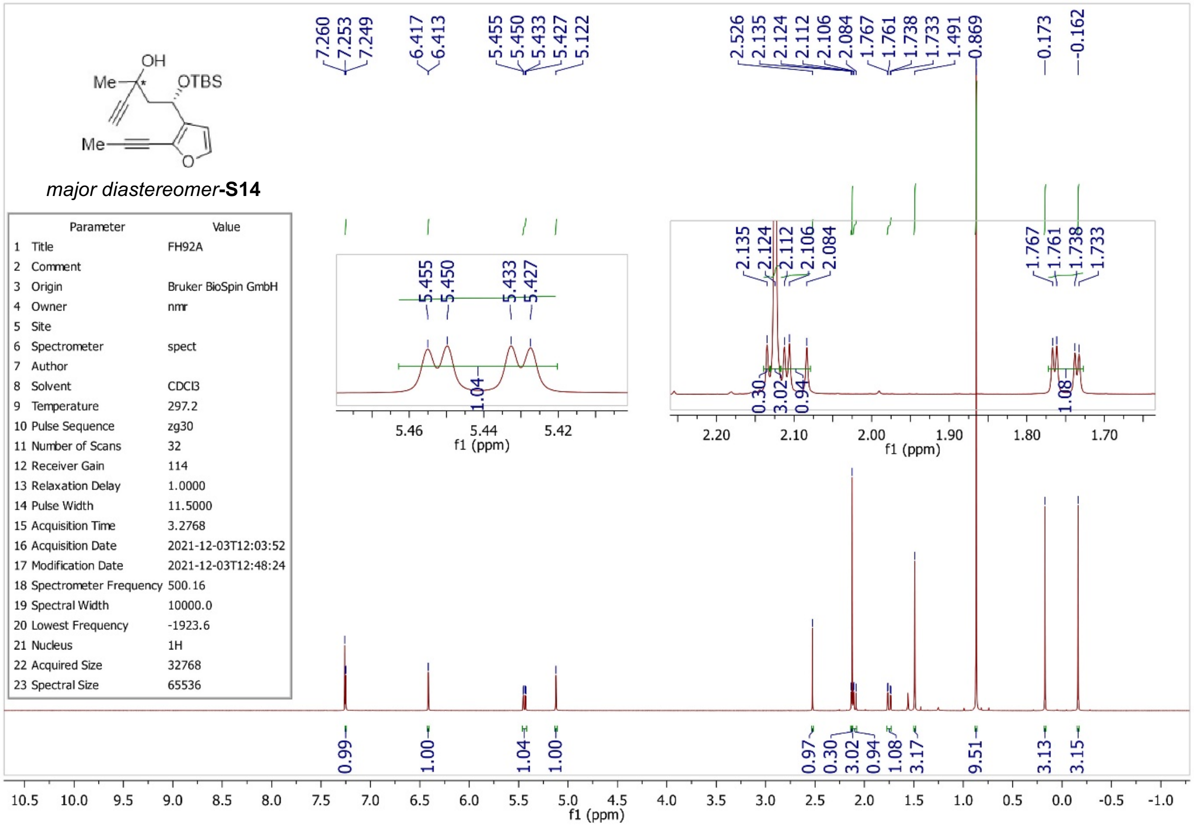

major diastereomer-S14
Parameter Value
1 Title FH92A
2 Comment
3 Origin Bruker BioSpin GmbH
4 Owner nmr
5 Site
6 Spectrometer spect
7 Author
8 Solvent CDCl3
9 Temperature 297.2
10 Pulse Sequence zg30
11 Number of Scans 32
12 Receiver Gain 114
13 Relaxation Delay 1.0000
14 Pulse Width 11.5000
15 Acquisition Time 3.2768
16 Acquisition Date 2021-12-03T12:03:52
17 Modification Date 2021-12-03T12:48:24
18 Spectrometer Frequency 500.16
19 Spectral Width 10000.0
20 Lowest Frequency -1923.6
21 Nucleus 1H
22 Acquired Size 32768
23 Spectral Size 65536
f1 (ppm)

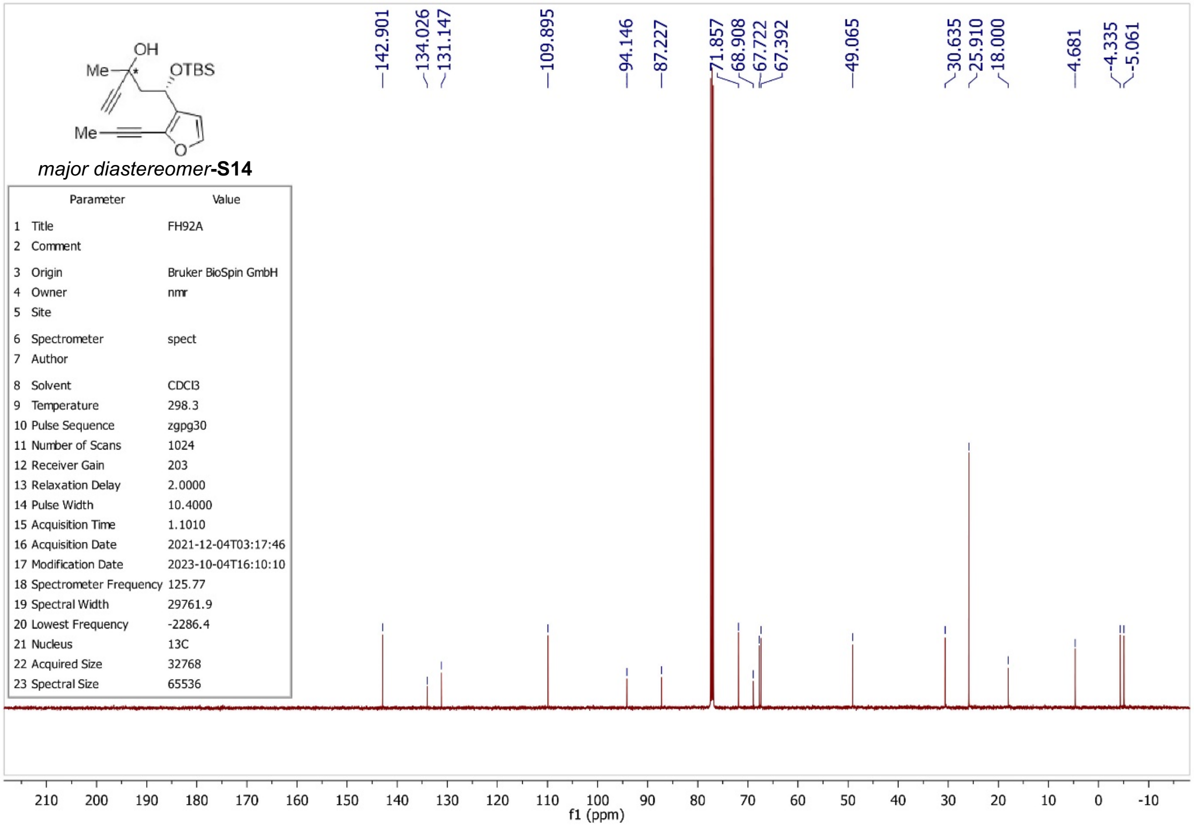

142.901
134.026
131.147
109.895
94.146
87.227
71.857
68.908
67.722
67.392
49.065
30.635
25.910
18.000
4.681
-4.335
-5.061
OH
Me
OTBS
Me
O
major diastereomer-S14
Parameter Value
1 Title FH92A
2 Comment
3 Origin Bruker BioSpin GmbH
4 Owner nmr
5 Site
6 Spectrometer spect
7 Author
8 Solvent CDCl3
9 Temperature 298.3
10 Pulse Sequence zgpg30
11 Number of Scans 1024
12 Receiver Gain 203
13 Relaxation Delay 2.0000
14 Pulse Width 10.4000
15 Acquisition Time 1.1010
16 Acquisition Date 2021-12-04T03:17:46
17 Modification Date 2023-10-04T16:10:10
18 Spectrometer Frequency 125.77
19 Spectral Width 29761.9
20 Lowest Frequency -2286.4
21 Nucleus 13C
22 Acquired Size 32768
23 Spectral Size 65536
210 200 190 180 170 160 150 140 130 120 110 100 90 80 70 60 50 40 30 20 10 0 -10
f1 (ppm)

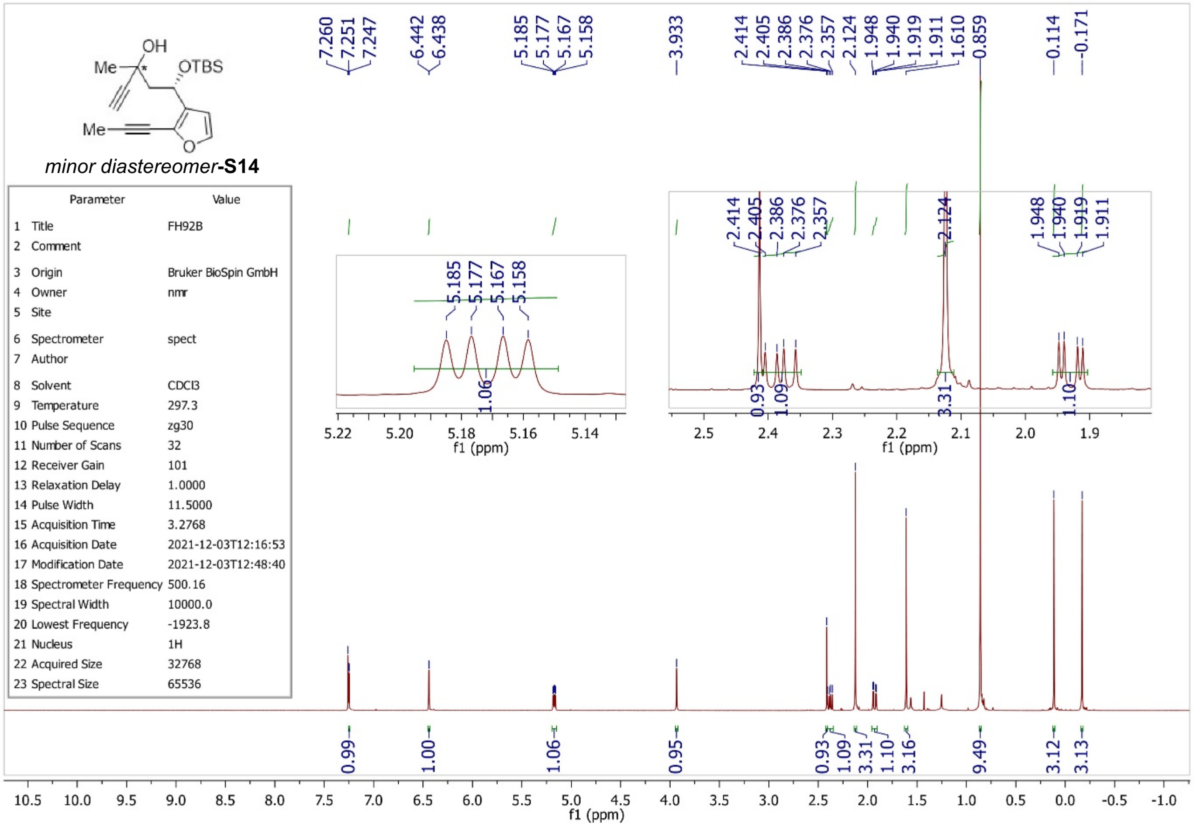
minor diastereomer-S14
Parameter Value
1 Title FH92B
2 Comment
3 Origin Bruker BioSpin GmbH
4 Owner nmr
5 Site
6 Spectrometer spect
7 Author
8 Solvent CDCl3
9 Temperature 297.3
10 Pulse Sequence zg30
11 Number of Scans 32
12 Receiver Gain 101
13 Relaxation Delay 1.0000
14 Pulse Width 11.5000
15 Acquisition Time 3.2768
16 Acquisition Date 2021-12-03T12:16:53
17 Modification Date 2021-12-03T12:48:40
18 Spectrometer Frequency 500.16
19 Spectral Width 10000.0
20 Lowest Frequency -1923.8
21 Nucleus 1H
22 Acquired Size 32768
23 Spectral Size 65536
7.260 7.251 7.247 6.442 6.438 5.185 5.177 5.167 5.158 3.933 2.414 2.405 2.386 2.376 2.357 2.124 1.948 1.940 1.919 1.911 1.610 0.859 0.114 -0.171
0.99 1.00 1.06 0.95 0.93 1.09 3.31 1.10 3.16 9.49 3.12 3.13
f1 (ppm)

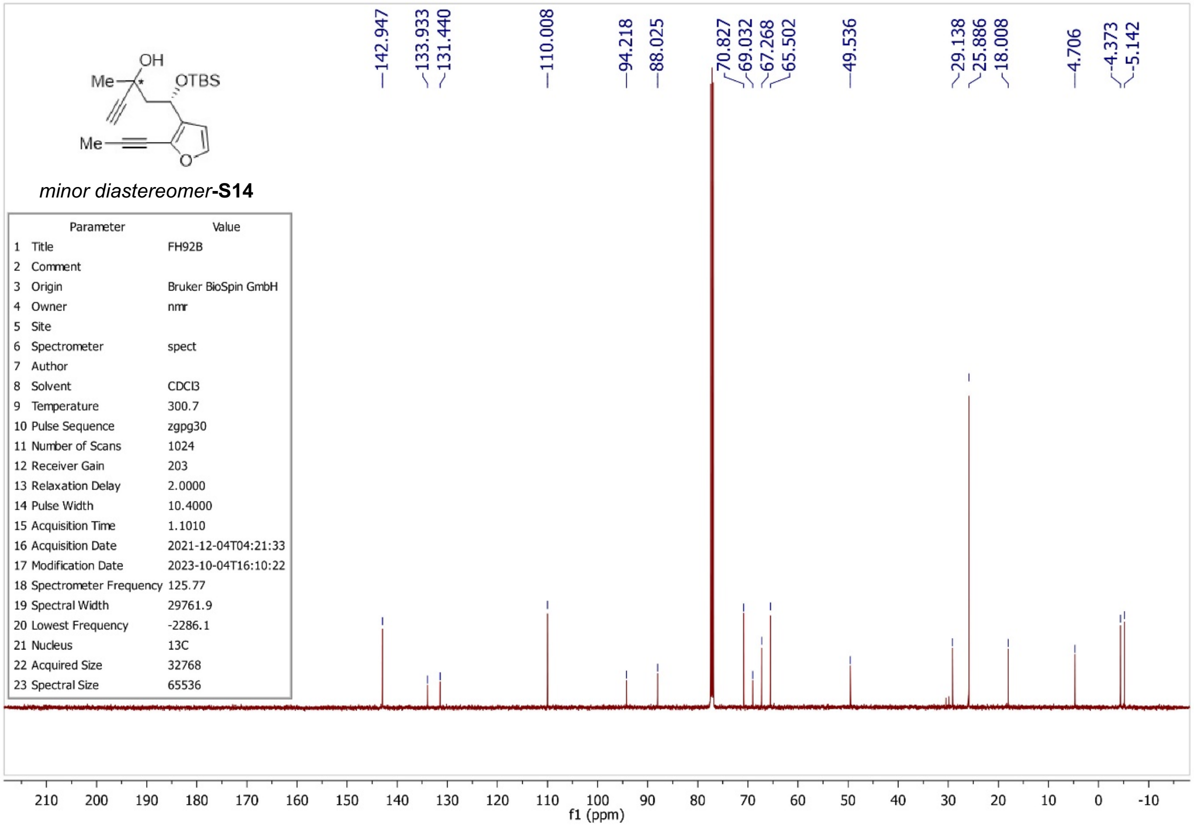
142.947
133.933
131.440
110.008
94.218
88.025
70.827
69.032
67.268
65.502
49.536
29.138
25.886
18.008
4.706
-4.373
-5.142
OH
Me
OTBS
Me
O
minor diastereomer-S14
Parameter Value
1 Title FH92B
2 Comment
3 Origin Bruker BioSpin GmbH
4 Owner nmr
5 Site
6 Spectrometer spect
7 Author
8 Solvent CDCl3
9 Temperature 300.7
10 Pulse Sequence zgpg30
11 Number of Scans 1024
12 Receiver Gain 203
13 Relaxation Delay 2.0000
14 Pulse Width 10.4000
15 Acquisition Time 1.1010
16 Acquisition Date 2021-12-04T04:21:33
17 Modification Date 2023-10-04T16:10:22
18 Spectrometer Frequency 125.77
19 Spectral Width 29761.9
20 Lowest Frequency -2286.1
21 Nucleus 13C
22 Acquired Size 32768
23 Spectral Size 65536
210 200 190 180 170 160 150 140 130 120 110 100 90 80 70 60 50 40 30 20 10 0 -10
f1 (ppm)

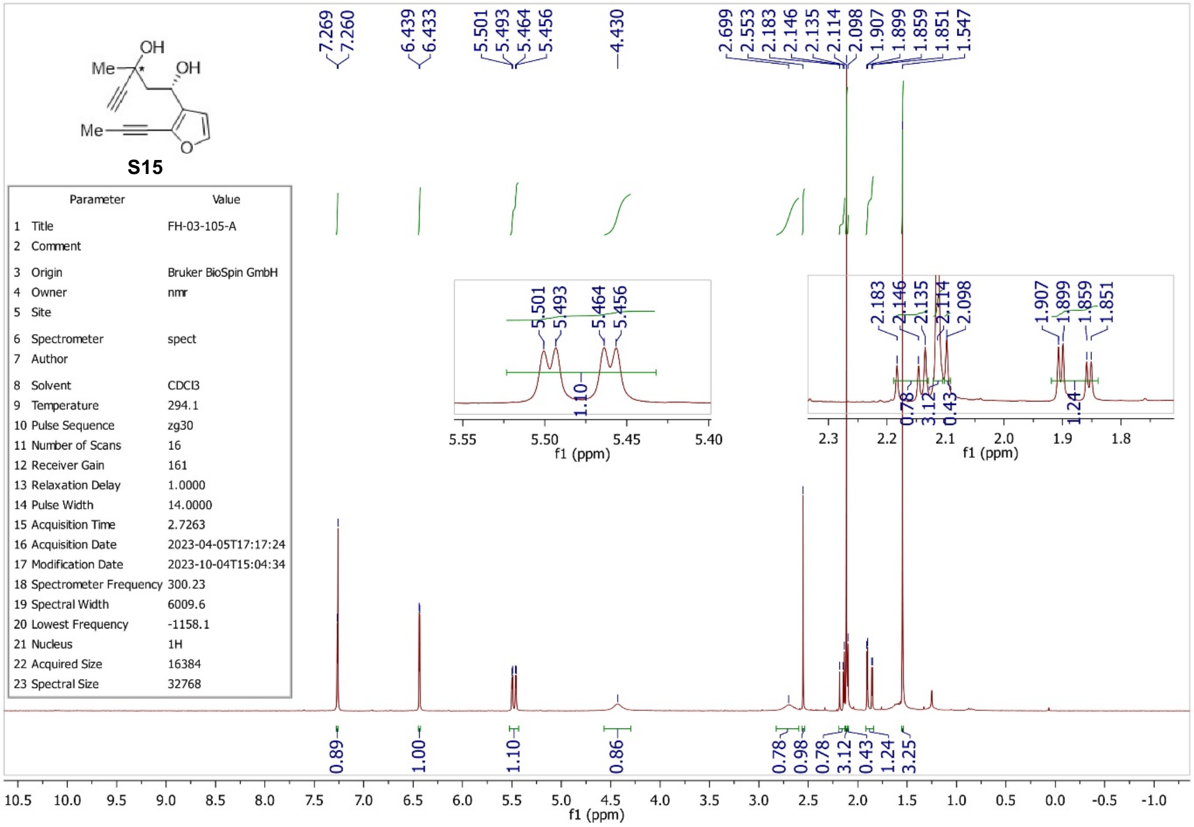
S15
Parameter Value
1 Title FH-03-105-A
2 Comment
3 Origin Bruker BioSpin GmbH
4 Owner nmr
5 Site
6 Spectrometer spect
7 Author
8 Solvent CDCl3
9 Temperature 294.1
10 Pulse Sequence zg30
11 Number of Scans 16
12 Receiver Gain 161
13 Relaxation Delay 1.0000
14 Pulse Width 14.0000
15 Acquisition Time 2.7263
16 Acquisition Date 2023-04-05T17:17:24
17 Modification Date 2023-10-04T15:04:34
18 Spectrometer Frequency 300.23
19 Spectral Width 6009.6
20 Lowest Frequency -1158.1
21 Nucleus 1H
22 Acquired Size 16384
23 Spectral Size 32768
7.269 7.260 6.439 6.433 5.501 5.493 5.464 5.456 4.430 2.699 2.553 2.183 2.146 2.135 2.114 2.098 1.907 1.899 1.859 1.851 1.547
0.89 1.00 1.10 0.86 0.78 0.98 0.78 3.12 0.43 1.24 3.25
f1 (ppm)

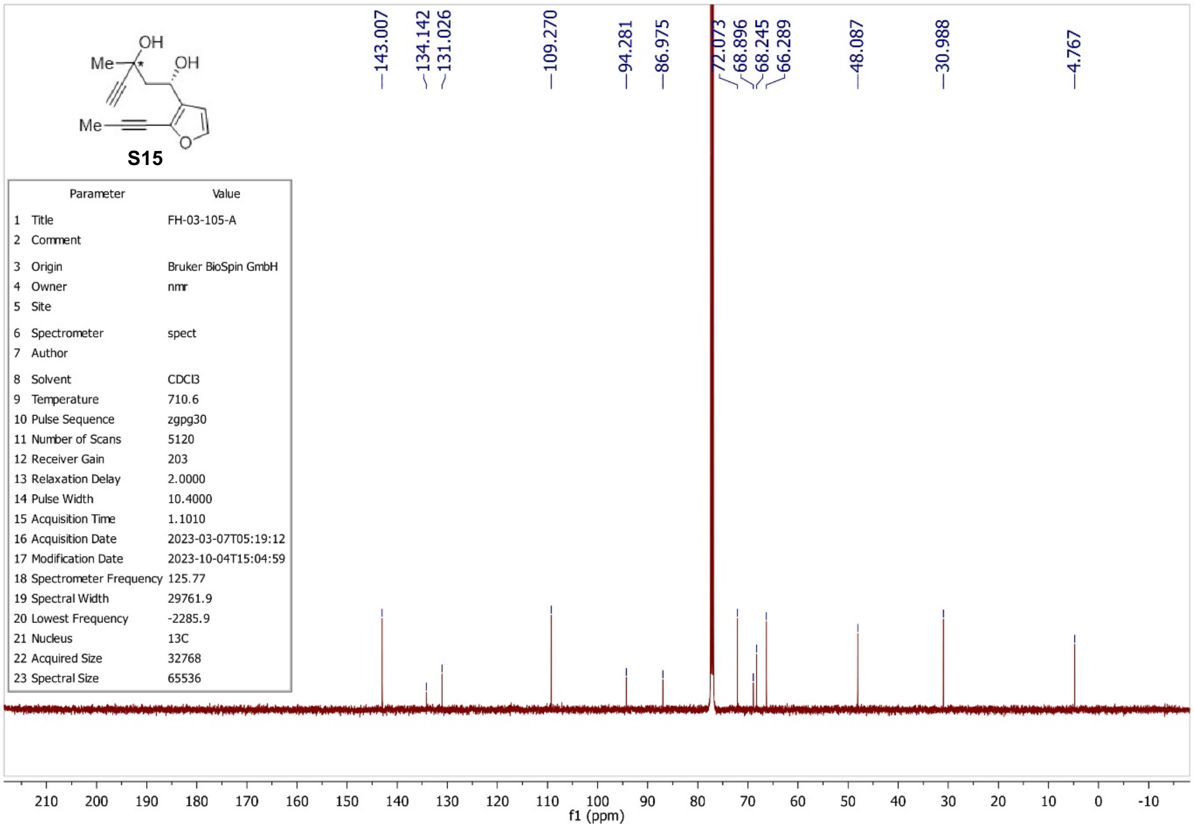
OH
Me
OH
Me
O
S15
Parameter Value
1 Title FH-03-105-A
2 Comment
3 Origin Bruker BioSpin GmbH
4 Owner nmr
5 Site
6 Spectrometer spect
7 Author
8 Solvent CDCl3
9 Temperature 710.6
10 Pulse Sequence zgpg30
11 Number of Scans 5120
12 Receiver Gain 203
13 Relaxation Delay 2.0000
14 Pulse Width 10.4000
15 Acquisition Time 1.1010
16 Acquisition Date 2023-03-07T05:19:12
17 Modification Date 2023-10-04T15:04:59
18 Spectrometer Frequency 125.77
19 Spectral Width 29761.9
20 Lowest Frequency -2285.9
21 Nucleus 13C
22 Acquired Size 32768
23 Spectral Size 65536
143.007
134.142
131.026
109.270
94.281
86.975
72.073
68.896
68.245
66.289
48.087
30.988
4.767
210 200 190 180 170 160 150 140 130 120 110 100 90 80 70 60 50 40 30 20 10 0 -10
f1 (ppm)

Table 1, entry 1: crude ^{1}H NMR, **14a**:**S7** ratio

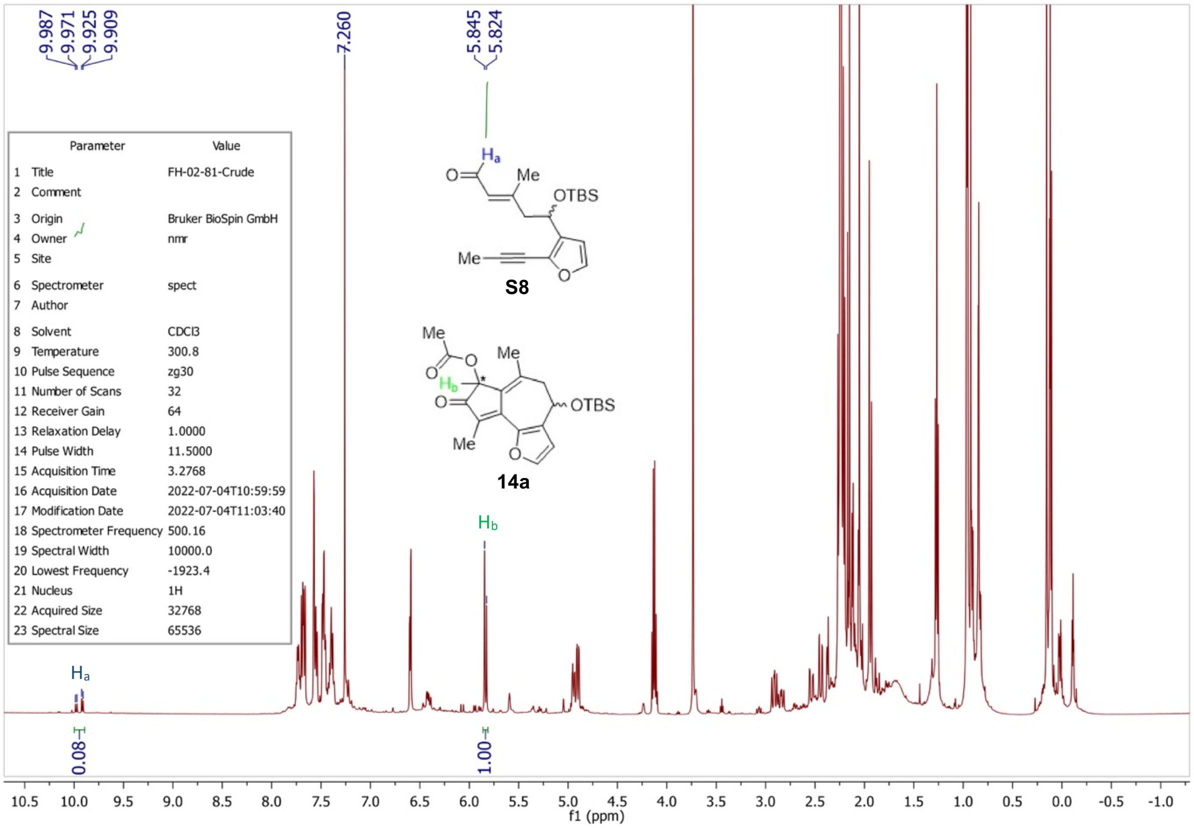

Table 1, entry 2: crude ^{1}H NMR, **14a**:**S7** ratio

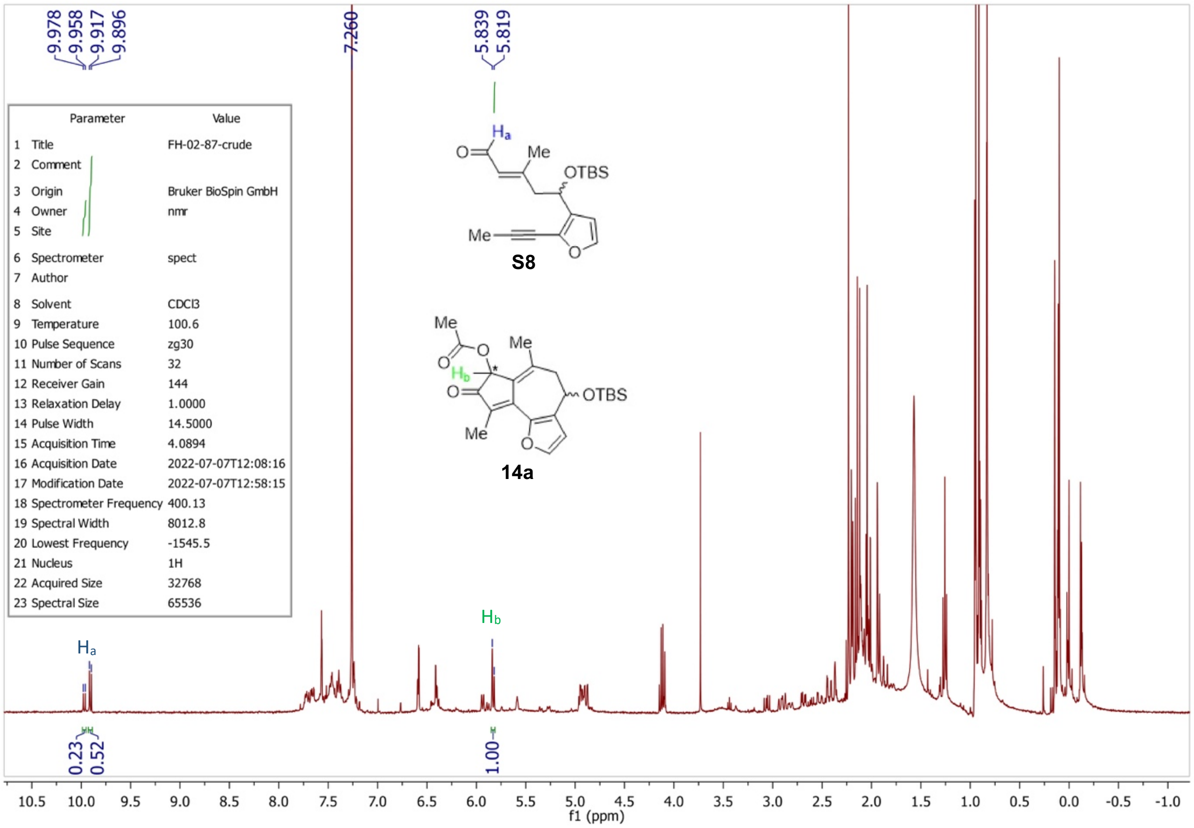

Table 1, entry 7: crude ^{1}H NMR, **14a**:**S7** ratio

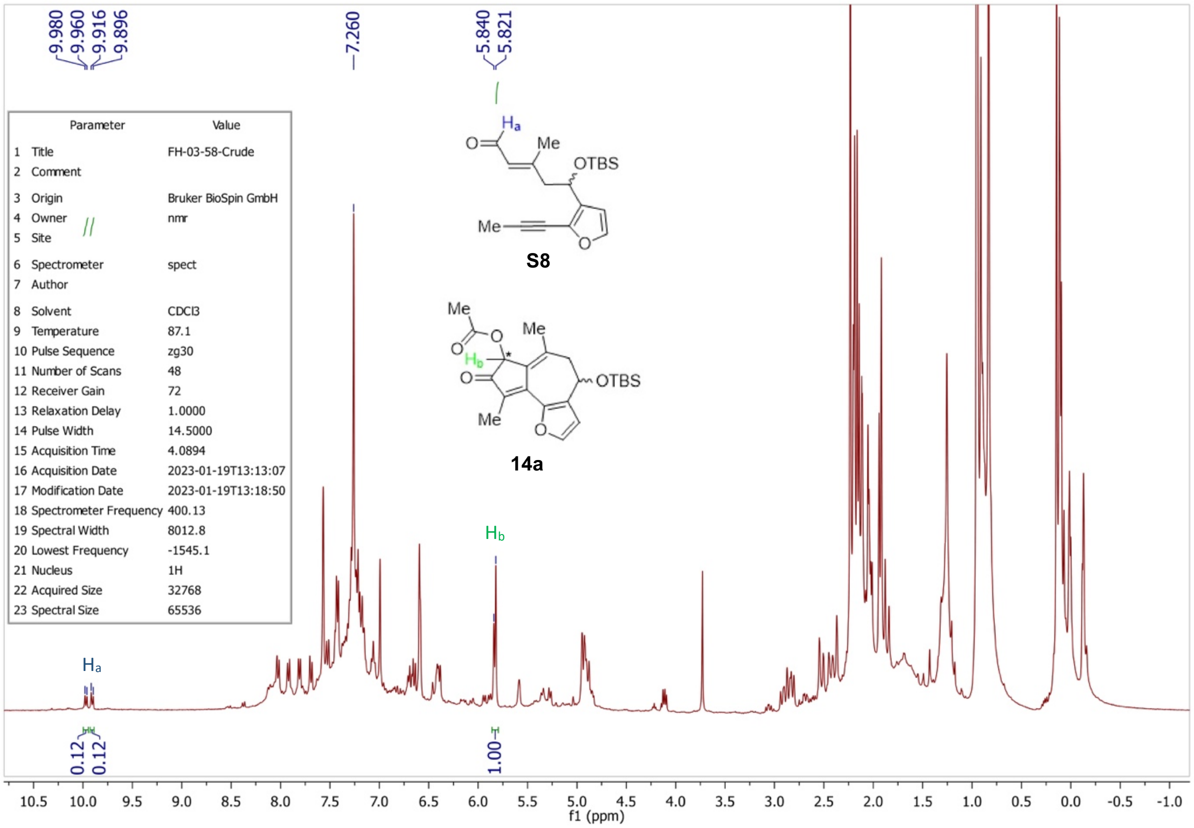

Table 1, entry 8: crude ^{1}H NMR, **14a**:**13a** ratio

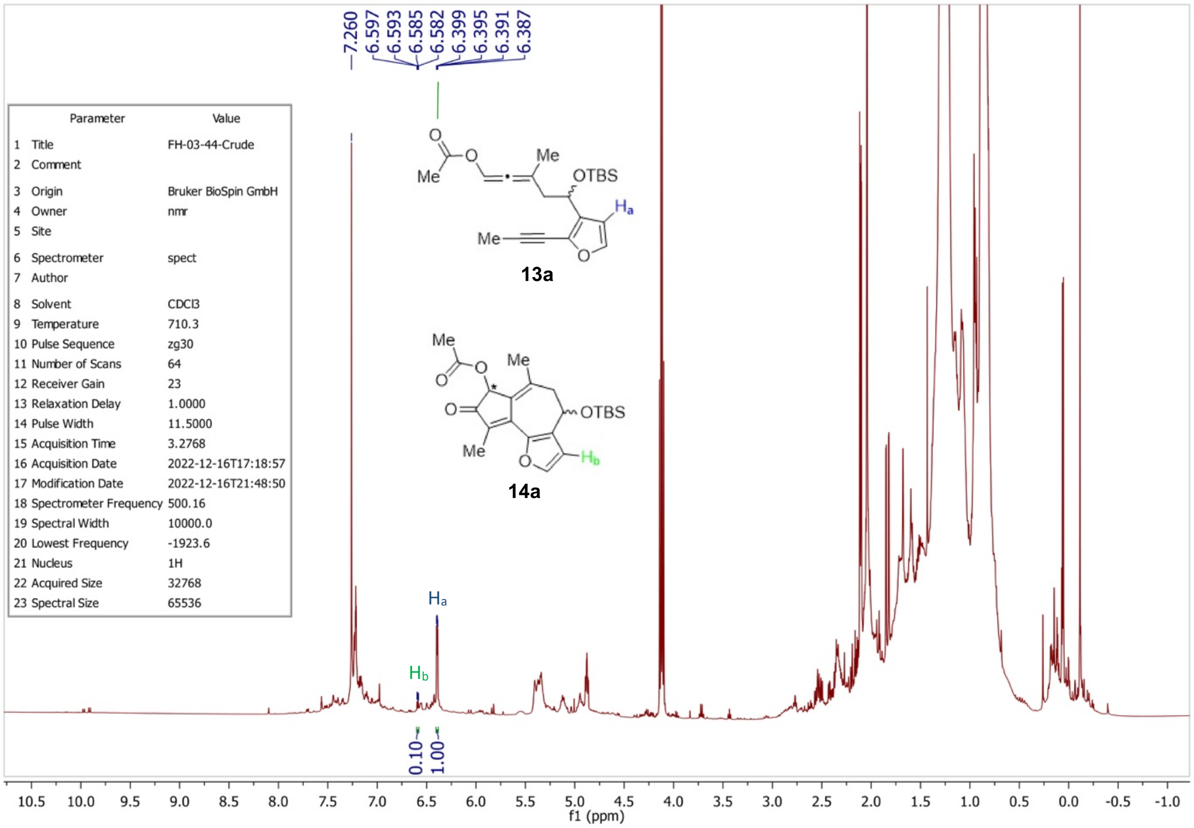

Table1, entry 3: crude ^{1}H NMR, **14b**:**S8** ratio

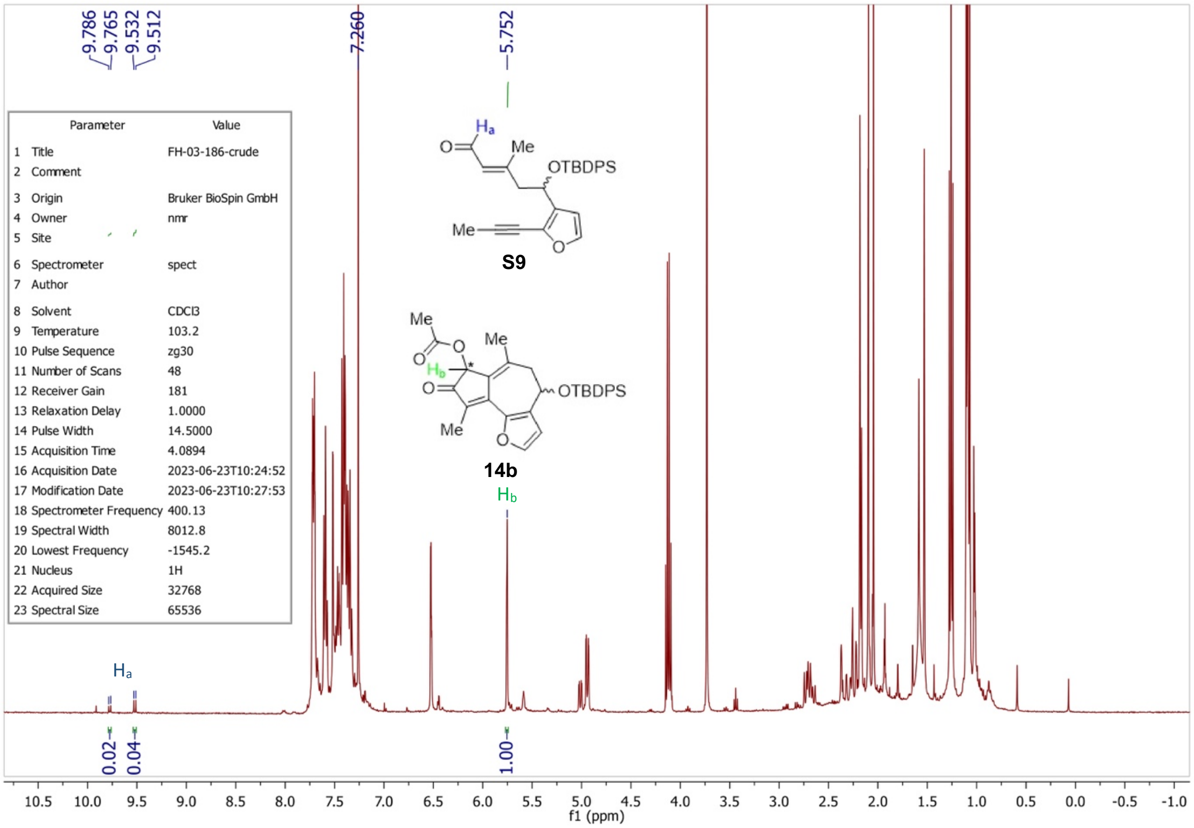

Table1, entry 5: crude ^{1}H NMR, **14d**:**S9** ratio

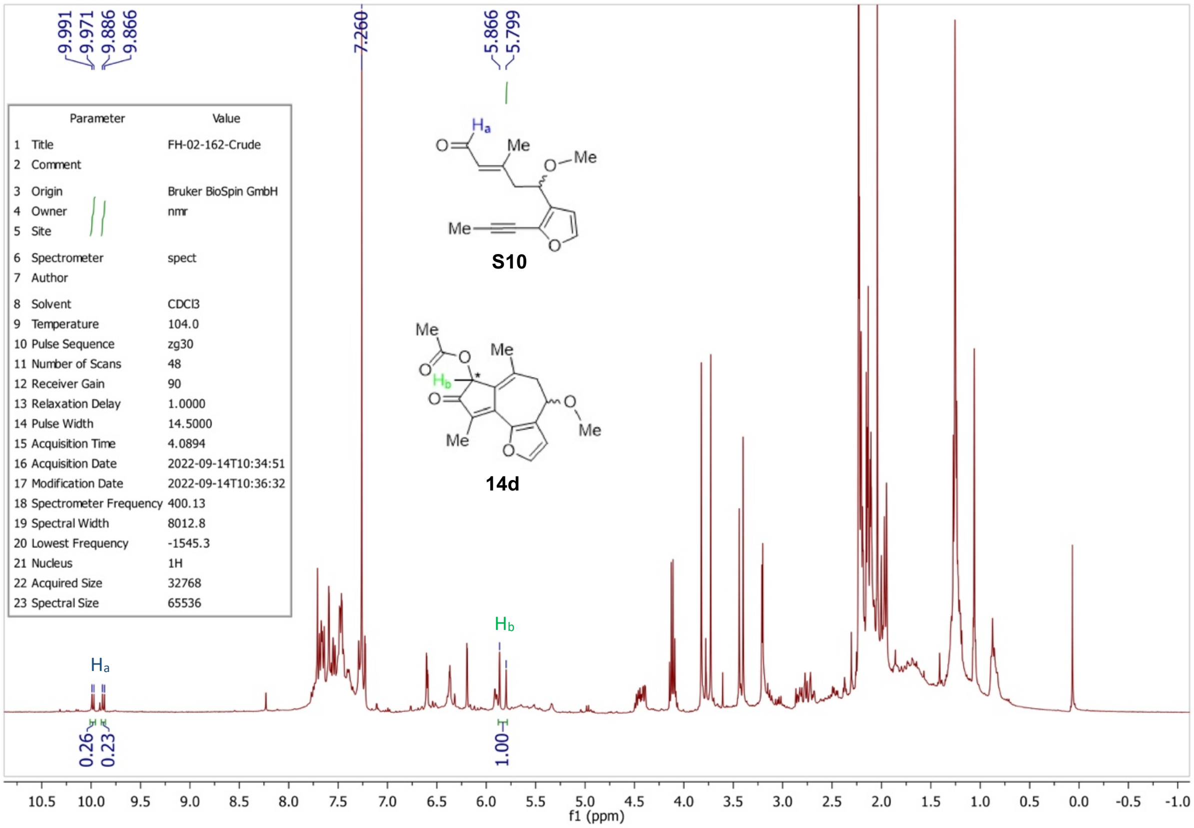

Table1, entry 6: crude ^{1}H NMR, **14e**:**S11** ratio

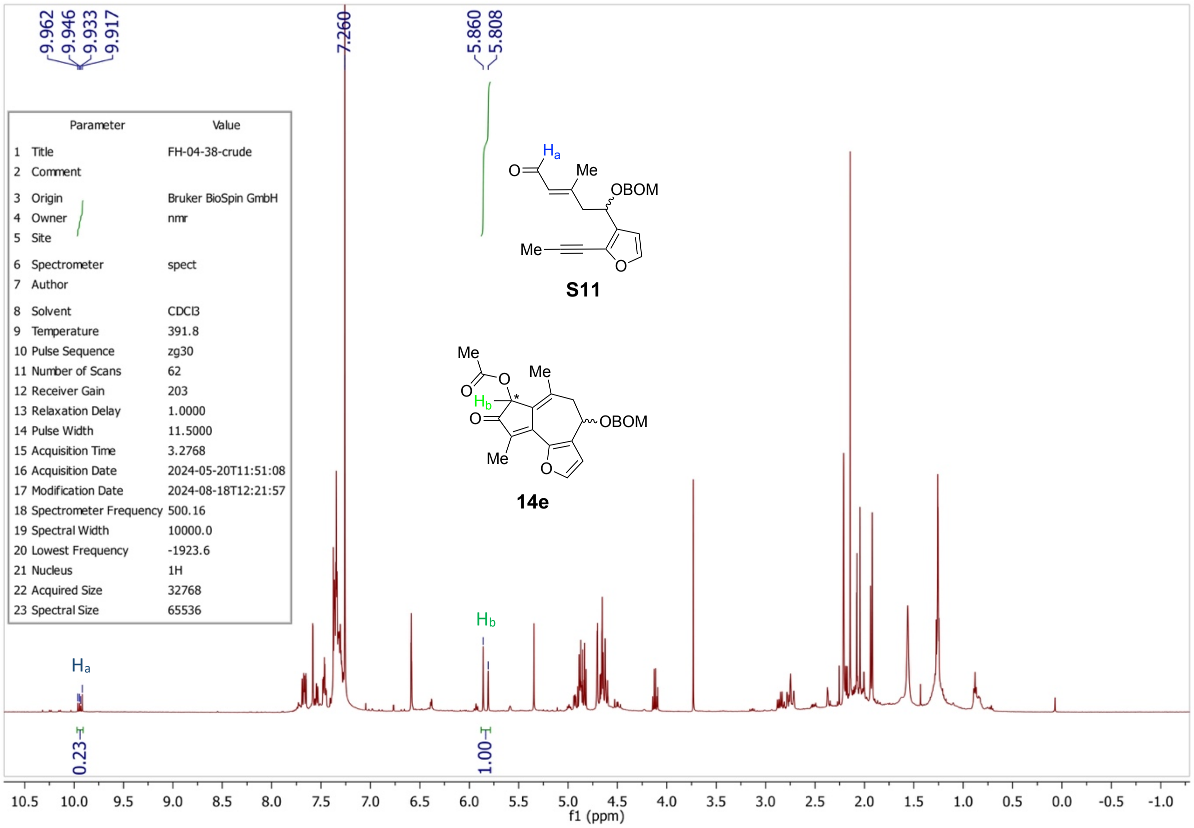

1. Deihl, E. D.; Jesikiewicz, L. T.; Newman, L. J.; Liu, P.; Brummond, K. M., Rh (I)-catalyzed allenic Pauson–Khand reaction to access the thapsigargin core: Influence of furan and allenyl chloroacetate groups on enantioselectivity. *Org. Lett.* **2022,** *24* (4), 995-999.
2. Lingham, A. R.; Hügel, H. M.; Rook, T. J., Studies towards the synthesis of salvinorin A. *Aust. J. Chem.* **2006,** *59* (5), 340-348.
3.Zhou, Y.; Shan, Z., (*R*)-or (*S*)-Bi-2-naphthol assisted, L-proline catalyzed direct aldol reaction. *Tetrahedron: Asymmetry* **2006,** *17* (11), 1671-1677.
4. Ma, K.; Martin, B. S.; Yin, X.; Dai, M., Natural product syntheses via carbonylative cyclizations. *Nat. Prod. Rep.* **2019,** *36* (1), 174-219.
5. Mace, L. H.; Shanmugham, M. S.; White, J. D.; Drew, M. G., A new route to furanoeremophilane sesquiterpenoids. Synthesis of Senecio metabolites (±)-6-hydroxyeuryopsin,(±)-1, 10-epoxy-6-hydroxyeuryopsin,(±)-toluccanolide A and (±)-toluccanolide C. *Org. Biomol. Chem.* **2006,** *4* (6), 1020-1031.
6. Hughes, C. C.; Kennedy-Smith, J. J.; Trauner, D., Synthetic studies toward the guanacastepenes. *Org. Lett.* **2003,** *5* (22), 4113-4115.
7. Deihl, E. D., Expanding the scope of the asymmetric allenic Pauson–Khand reaction towards the synthesis of thapsigargin and analogues, University of Pittsburgh, Pittsburgh, PA, 2022.
8. Matsuki, N.; Inoue, Y.; Mori, T., Orbital control of photochemical rearrangement of 4-Aryl-1, 1-dicyano-1-butenes through the hyperconjugative substitution on the linker chain. *J. Phys. Chem. Lett.* **2016,** *7* (24), 4957-4961.
9. Chuang, K. V.; Xu, C.; Reisman, S. E., A 15-step synthesis of (+)-ryanodol. *Science* **2016,** *353* (6302), 912-915.
10. Wells, S. M.; Brummond, K. M., Conditions for a Rh (I)-catalyzed [2+ 2+ 1] cycloaddition reaction with methyl substituted allenes and alkynes. *Tetrahedron Lett.* **2015,** *56* (23), 3546-3549.
11. Weston J. Umstead Ph.D. Sr. Product Manager; Tracy Hartlage, Sales Manager Daicel Chiral Technologies, PA, USA.
12. Dachavaram, S. S.; Kalyankar, K. B.; Das, S., First stereoselective total synthesis of Neocosmosin A: a facile approach. *Tetrahedron Lett.* **2014,** *55* (41), 5629-5631.
13. Sondack, D. L., Tlc separation and identification of diastereomers of D-ergonovine maleate. *J. Pharm. Sci.* **1974,** *63* (7), 1141-1143.
14. Sit, M. K.; Cao, H. H.; Wu, Y.-D.; Yip, T. C.; Bendel, L. E.; Zhang, W.; Dai, W.-M., Synthesis of the macrolactone cores of maltepolides via a diene–ene ring-closing metathesis strategy. *Org. Lett.* **2023,** *25* (10), 1633-1637.
15. Panda, S.; Ready, J. M., Tandem allylation/1, 2-boronate rearrangement for the asymmetric synthesis of indolines with adjacent quaternary stereocenters. *J. Am. Chem. Soc.* **2018,** *140* (41), 13242-13252.
16. List, B.; Lerner, R. A.; Barbas, C. F., Proline-catalyzed direct asymmetric aldol reactions. *J. Am. Chem. Soc.* **2000,** *122* (10), 2395-2396.
17. Krattiger, P.; Kovasy, R.; Revell, J. D.; Ivan, S.; Wennemers, H., Increased structural complexity leads to higher activity: peptides as efficient and versatile catalysts for asymmetric aldol reactions. *Org. Lett.* **2005,** *7* (6), 1101-1103.
18. Paladhi, S.; Das, J.; Samanta, M.; Dash, J., Asymmetric aldol reaction of thiazole-carbaldehydes: Regio-and stereoselective synthesis of tubuvalin analogues. *Adv. Synth. Catal.* **2014,** *356* (16), 3370-3376.
19. Scott, E.; Stavenger, A., Lewis base-catalyzed, asymmetric aldol additions of methyl ketone enolates. *J. Org. Chem.* **1998,** *63* (4), 918-919.
20. Shevlin, M., Practical high-throughput experimentation for chemists. *ACS Med. Chem. Lett.* **2017,** *8* (6), 601-607.
21. Reichardt, C., Solvatochromic dyes as solvent polarity indicators. *Chem. Rev.* **1994,** *94* (8), 2319-2358.
