# Supplementary material for: Stereo-Differentiating Asymmetric Rh(I)-Catalyzed Pauson–Khand Reaction: A DFT-Informed Approach to Thapsigargin Stereoisomers
Source: J Am Chem Soc. 2024 Dec 20;147(1):498–509. doi: 10.1021/jacs.4c11661 (PMC11726561; doi:10.1021/jacs.4c11661)
Supplement: Supplementary file 2 — ja4c11661_si_002.pdf [file ja4c11661_si_002.pdf]

Supporting Information for Computational Studies

Stereo-Differentiating Asymmetric Rh(I)-Catalyzed Pauson–Khand Reaction: A DFT-Informed Approach to Thapsigargin Stereoisomers.

Fatemeh Haghighi,[a] Luke T. Jesikiewicz,[a] Corrinne E. Stahl,[a] Jordan Nafie,[b] Amanda Ortega-Vega,[a] Peng Liu,*[a] and Kay M. Brummond*[a]

[a] Department of Chemistry, University of Pittsburgh, Pittsburgh, Pennsylvania 15260, United States

[b] BioTools, Inc., Jupiter, Florida 33478, United States

Corresponding authors: pengliu@pitt.edu; kbrummon@pitt.edu

Table of Contents

Computational details of DFT Calculations of the oxidative cyclization reaction energy profiles……………………………………………………………………………..…...…S-5

Computed oxidative cyclization transition state isomers………………..…………..……...…S-5

Distortion-interaction analysis of key transition state isomers…………..………………......….S-8

Non-covalent interaction surfaces of oxidative cyclization TS……………………………...S-11

Comparison of competing four-coordinate and CO-only oxidative cyclization pathways…….S-13

Cartesian coordinates and energies of DFT-computed structures in the oxidative cyclization pathways...……………………………………………………………………….........S-15

Assignment of absolute configuration of **17** using VCD …………………………...…......S-103

VCD measurements………………………………………………………….…….. S-103

VCD calculations…………………………………………………………….………...S-103

Energies and cartesian coordinates used in VCD calculations……………………….…..….S-105

Cai• VCD analysis…………………….………………………..……………..….S-112

Assignment of absolute and relative configuration of **14g** using VCD………..…...…..…….S-116

VCD measurements………………………………………………………….…...….S-116

VCD calculations…………………………………………………….……...….S-117

Energies and cartesian coordinates used in VCD calculations …….…………….……..S-122

Cai• VCD analysis…………………….………………………..……………..….S-143

Assignment of relative configuration of **14a** using ^{13}C NMR……..……………………...……S-146

NMR workflow for computed ^{13}C NMR chemical shifts………………………….…..S-146

DP4 analysis for major isomer……………………..……………………….…..…..S-149

Cartesian coordinates for *trans*-**14a**……………………………………………...….S-149

DP4 analysis for minor isomer……………………...……………………….…..…..S-165

Cartesian coordinates for *cis*-**14a**……………………………………………..…..S-165

Comparing yield and selectivity of proline-catalyzed asymmetric aldol reaction of **15** and **16**……

………………………………………………………………………………………S-179

LUMO values and cartesian coordinates of **15** and **16**…………………………..………..S-179

References……………………………………………………………………..…...…...S-183

Figure S1. Possible oxidative cyclization transition state isomers with (*R*)-**13d** …….…......…S-6

Figure S2. Possible oxidative cyclization transition state isomers with (*S*)-**13d** …….……...…S-7

Figure S3. Summary of the N-aryl rotamers of lowest energy oxidative cyclization TS …...…S-8

Figure S4. Defined fragments for full substrate, furan, and ether through-space interaction calculations……………………………………………………………...……………………...S-9

Figure S5. NCIPlot non-covalent interaction diagrams for **TS1a** and **TS1b** S-12

Figure S6. NCIPlot non-covalent interaction diagrams for **TS2a** and **TS2b**……..………..…S-12

Figure S7. Comparison of four- (**TS3a** and **TS3b**) and five- (**TS1a** and **TS1b**) coordinated oxidative cyclization pathways……………………………..……………………………S-14

Figure S8. Comparison of CO-only (**TS4a** and **TS4b**) and five-coordinated (**TS1a** and **TS1b**) oxidative cyclization pathways……………………………..……………………….....S-15

Figure S9. Experimental VCD (blue) and IR (green) spectra of (*S*)-**17**...................................S-104

Figure S10. DFT-calculated (green) and experimental (blue) VCD and IR spectra of (*R*)-**17** (Boltzmann weighted based Gibbs free energies)..…S-105

Figure S11. Cai.factor for (*R*)-**17**…………………………………………….........…S-112

Figure S12. DFT calculated (green) and experimental (blue) VCD and IR spectra of (2*S*,8*S*)-**14g** (electronic energy weighted)...…S-118

Figure S13. Experimental VCD (blue) and IR (green) spectra of (2*S*,8*S*)-**14g**......................…S-118

Figure S14. Experimental VCD (blue) and IR (green) spectra of (2*R*,8*R*)-**14g**......................…S-119

Figure S15. DFT calculated VCD spectra of (2*R*,8*S*)-**14g** (purple) vs. (2*R*,8*R*)-**14g** (green) and experimental VCD spectrum of (2*R*,8*R*)-**14g** (blue) (electronic energy weighted)……………………………………………………………………………..S-119

Figure S16. Experimental VCD (blue) and IR (green) spectra of (2*S*,8*R*)-**14g**.....................…S-120

Figure S17. DFT calculated VCD spectra of (2*S*,8*S*)-**14g** (purple) vs. (2*S*,8*R*)-**14g** (green) and experimental VCD spectrum of (2*S*,8*R*)-**14g** (blue) (electronic energy weighted)......………..S-120

Figure S18. DFT calculated (green) and experimental (blue) VCD and IR spectra of (2*S*,8*S*)-**14g** (Boltzmann weighted based Gibbs free energies)...…………………………..….........…S-121

Figure S19. DFT calculated (green) and experimental (blue) VCD and IR spectra of (2*R*,8*R*)-**14g** (Boltzmann weighted based Gibbs free energies)……………..…S-121

Figure S20. DFT calculated (green) and experimental (blue) VCD and IR spectra of (2*S*,8*R*)-**14g** (Boltzmann weighted based Gibbs free energies)..…S-122

Figure S21. Cai.factor for for (2*R*,8*R*)-**14g**……………………………………….......…S-142

Figure S22. *Trans*-**14a** with atom labels………………………………………. ………….S-147

Figure S23. Calculated (*trans*-**14a**) minus experimental (major isomer) chemical shifts...…S-147

Figure S24. Calculated (*cis*-**14a**) minus experimental (minor isomer) chemical shifts…...…S-163

Figure S25. LUMO plot of **15**……………………………………………………………..S-179

Figure S26. LUMO plot of **16**……………………………………………………………..S-181

Table S1: Distortion-interaction analysis of key oxidative cyclization transition states……..…S-8

Table S2: Distortion-Interaction calculation (including through-bond) of oxidative cyclization transition states ……………………………………………………………………..…S-10

Table S3. Computed NMR chemical shifts for *trans*-**14a**……………………..……………..S-147

Table S4. Computed NMR chemical shifts for *trans*-**14a**……………………..……………..S-163

Table S5. ^{1}H (700 MHz) and ^{13}C NMR (175 MHz) data (computed and experimental) for *trans*- and *cis*-**14a** ($CDCl_3$) ……….……………………………………………..…...…..…S-177

Computational details of DFT Calculations of the oxidative cyclization reaction energy profiles

DFT calculations of the stereodetermining oxidative cyclization reaction pathways were performed using the Gaussian 16 software package[1]. Molecular geometries were optimized using the B3LYP functional[2,3] with Grimme's DFT-D3 dispersion correction[4] and zero dampening. The 6-31G(d) basis set was used for all non-metal atoms and LANL2DZ for rhodium in the gas phase. Single point energy calculations were performed using the ωB97X-D functional[5] with the def2-TZVP basis set with the SMD solvation model[6] in dichloroethane (DCE). The 3D images of optimized structures were prepared using CYLView.[7]

Non-covalent interactions of transition states were visualized by generating NCIPLOT from .wfn files for two molecular fragments computed at the M06/3-21G level of theory. One fragment contains the (*S*)-MonoPhos-alkene ligand, Rh, and 2 CO ligands and the other fragment contains the allene-yne substate **13d**. The NCIPLOT surface cube files were generated using NCIplot[8] and figures were rendered in PyMOL.[9]

Computed oxidative cyclization transition state isomers

In total, 28 total oxidative cyclization transition state isomers and conformers leading to the four stereoisomers of **14d** were located (Figure S1). These include isomers placing the (*S*)-MonoPhos-alkene ligand at three possible coordination sites on the Rh center, and two rotamers of the (*S*)-MonoPhos-alkene ligand about the phosphorus-rhodium bond (designated "p-rot"). In addition, ligand conformers involving rotations of the *N*-aryl group about the P-N bond were also considered (the higher energy TS conformer of the two is designated as "n-rot"). This leads to

conformers with a T-shaped π/π interaction between the *N*-aryl group and the napthyl portion of the backbone of the ligand, which was observed in our previous studies[10,11], as well as those with a parallel-displaced π/π interaction between the *N*-aryl group and the napthyl part of the ligand backbone. The formation of (2*R*,8*R*)-*trans*-**14d** from (*R*)-**13d** and (2*R*, 8*S*)-*cis*-**14d** from (*S*)-**13d** are favored regardless of the *N*-aryl conformation of the (*S*)-MonoPhos-alkene ligand.

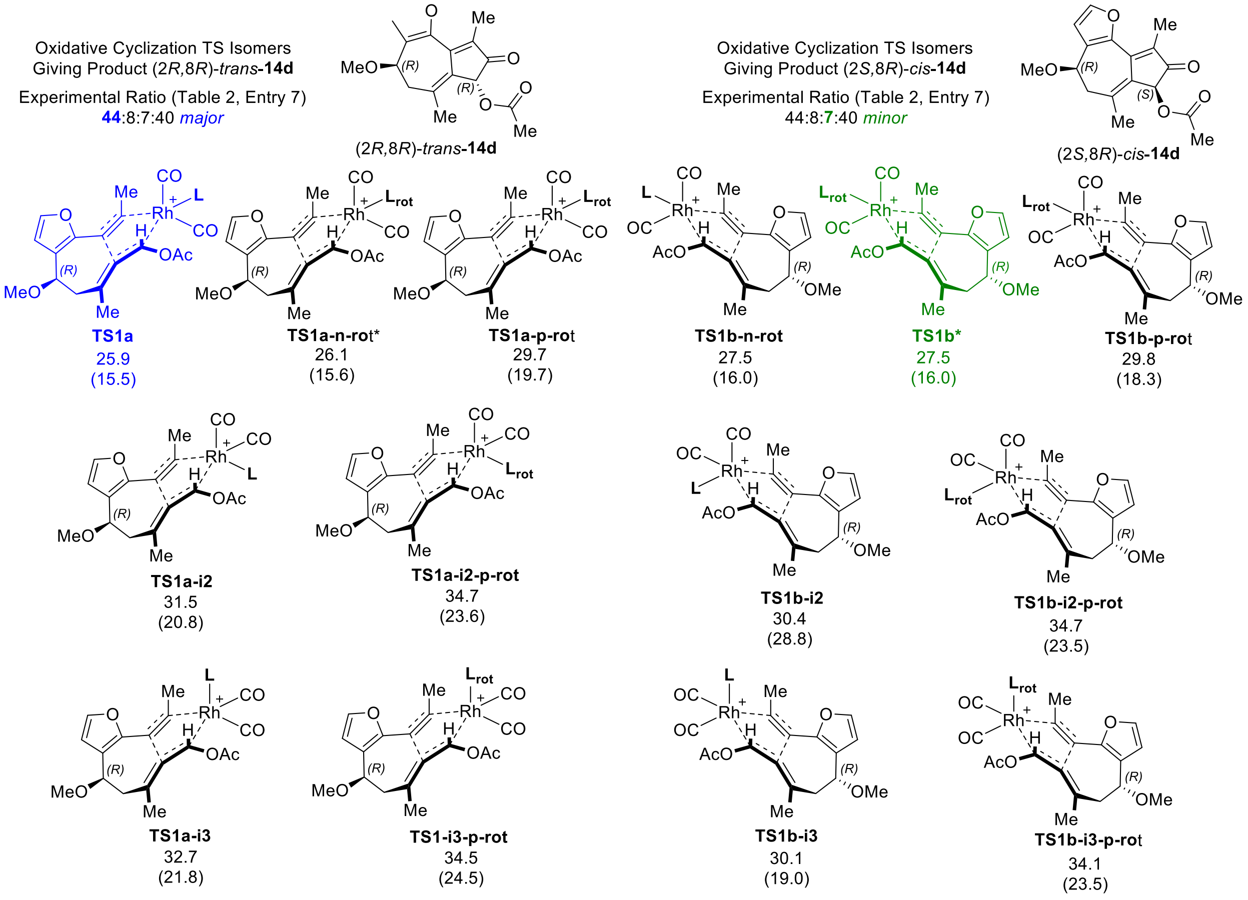

* indicates TS isomer with (*S*)-MonoPhos ligand (**4**) in the paralell displaced π/π conformation

Figure S1. Oxidative cyclization transition state isomers with **(*R*)-13d.** Activation Gibbs free energies and enthalpies (in parentheses) are shown in kcal/mol with respect to **20.**

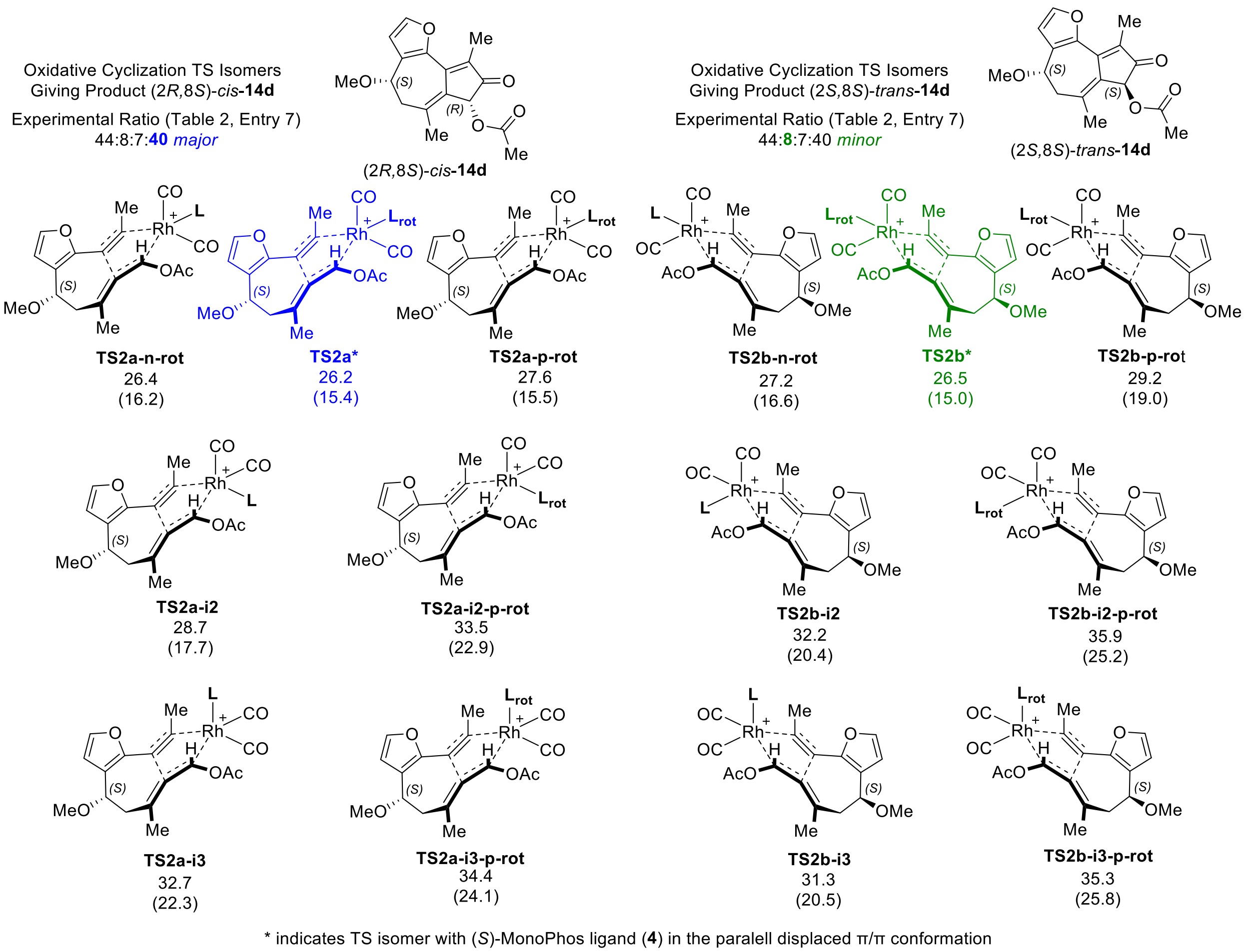

Figure S2. Oxidative cyclization transition state isomers with **(*S*)-13d.** Activation Gibbs free energies and enthalpies (in parentheses) are shown in kcal/mol with respect to **20.**

TS Name	$\Delta H^{\ddagger}$ (kcal/mol)	$\Delta G^{\ddagger}$ (kcal/mol)
**TS1a** (T-shaped π-π)	15.5	**25.9**
**TS1a-n-rot** (parallel displaced π-π)	15.6	26.1
**TS1b-n-rot** (T-shaped π-π)	17.3	28.1
**TS1b** (parallel displaced π-π)	16.0	**27.5**
**TS2a-n-rot** (T-shaped π-π)	16.2	26.4
**TS2a** (parallel displaced π-π)	15.4	**26.2**
**TS2b-n-rot** (T-shaped π-π)	16.6	27.2
**TS2b** (parallel displaced π-π)	15.0	**26.5**
$\Delta\Delta G/H^{\ddagger}$ (**TS1a/b**)	0.5	1.6
$\Delta\Delta G/H^{\ddagger}$ (**TS2a/b**)	-0.4	0.3

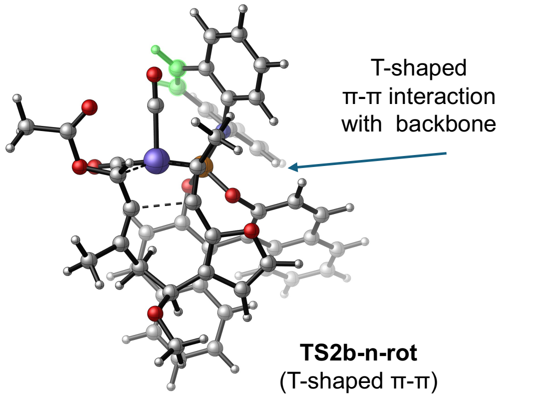

TS2b-n-rot
(T-shaped π-π)

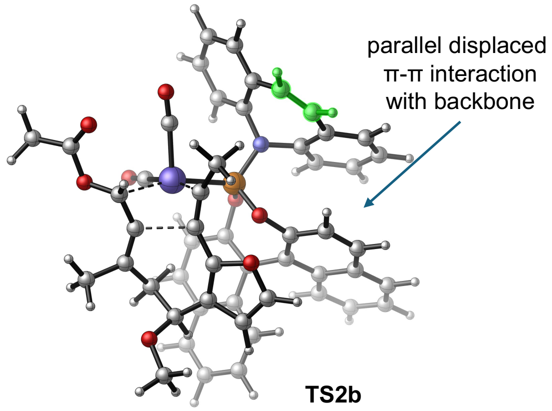

TS2b
(parallel displaced π-π)

Figure S3. Summary of the N-aryl rotomers of lowest energy oxidative cyclization TS

Distortion-interaction analysis of lowest-energy transition state isomers

Table S1. Distortion-Interaction Analysis of Oxidative Cyclization Transition States

	**TS1a** leading to (2*R*,8*R*)-*trans*-**14d**	**TS1b** leading to (2*S*,8*R*)-*cis*-**14d**	**TS2a** leading to (2*R*,8*S*)-*cis*-**14d**	**TS2b** leading to (2*S*,8*S*)-*trans*-**14d**
$\Delta G^{\ddagger}$ (kcal/mol)	25.9	27.5	26.2	26.5
$\Delta H^{\ddagger}$ (kcal/mol)	15.5	16.0	15.4	15.0
$\Delta E_{\text{int-space}}$ (kcal/mol)	-7.9	-6.4	-7.3	-7.6
$\Delta E_{\text{int-space (furan)}}$ (kcal/mol)	-3.9	-2.2	-4.7	-3.1
$\Delta E_{\text{int-space (ether)}}$ (kcal/mol)	-3.7	-3.1	-2.9	-4.4
$\Delta E_{\text{dist (substrate)}}$ (kcal/mol)	62.9	71.1	67.7	67.4

$\Delta E_{\text{dist (ligand)}}$ (kcal/mol)	1.9	0.5	1.5	0.6
$\Delta E_{\text{dist (total)}}$ (kcal/mol)	64.8	71.6	69.2	68.0

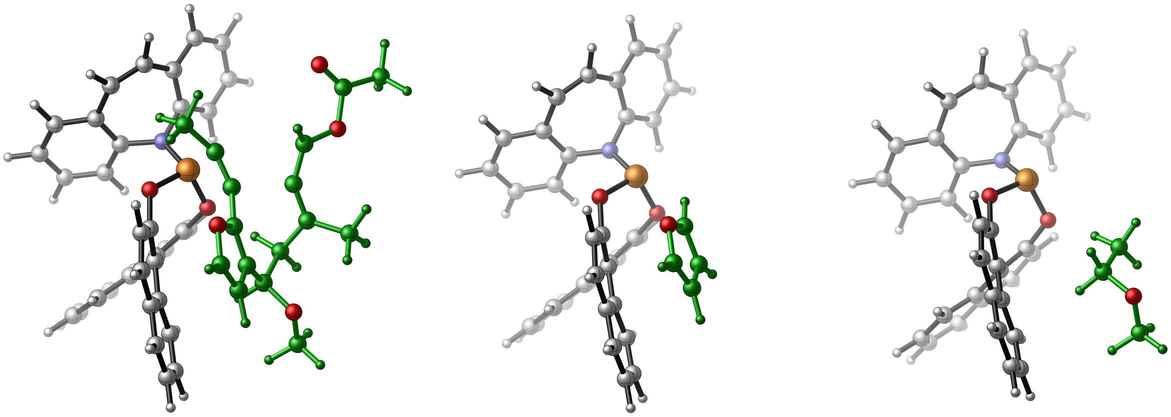

TS1a
(Full Substrate Int – Space)

TS1a
(Furan Fragment Int – Space)

TS1a
(Ether Fragment Int – Space)

Figure S4: Defined fragments for through-space interaction energy calculations between the ligand and the full substrate ($\Delta E_{\text{int-space}}$), furan ($\Delta E_{\text{int-space (furan)}}$), and ether ($\Delta E_{\text{int-space (ether)}}$).

The distortion energies (ΔE_{dist}) were calculated from the energy difference between the phosphoramidite ligand/allene-yne substrate at their transition state geometry and the ground state geometry (*i.e.*, free ligand and the reactant). The through-space interaction energies between the phosphoramidite ligand and the allene-yne substrate ($\Delta E_{\text{int-space}}$) were computed from the interaction energies between the ligand and the substrate in a non-covalent ligand-substrate complex in their TS geometry without Rh and CO moieties. These were calculated from the single point energy of non-covalent ligand-substrate complex and the sum of the single point energies of the ligand and the substrate itself at the TS geometry. We further dissected the overall through-space ligand-substrate interaction energies into the individual contributions of the furan ($\Delta E_{\text{int-space (furan)}}$) and ether portions ($\Delta E_{\text{int-space (ether)}}$) of the substate. The interaction energy between the ligand and the furan ($\Delta E_{\text{int-space (furan)}}$) was calculated by removing all other atoms in the substrate besides

the furan group and adding hydrogens to replace the furan-carbon bonds. The interaction energy between the ligand and the ether ($\Delta E_{\text{int-space (ether)}}$) was calculated by removing all substrate atoms besides the C9 carbon, C8 carbon, and OMe group, and adding hydrogens on the C8 and C9 atoms. Each of the fragments had a single point energy calculated with and without the chiral ligand in the same fashion as the overall through-space energy.

Lastly, in order to model the through-bond effects from the Rh, another variation of distortion interaction energies was calculated. The distortion energies were calculated from the energy difference between the Rh-ligand complex ($\Delta E_{\text{dist (RhCOLig)}}$) and allene-yne substrate ($\Delta E_{\text{dist (subst. rel to Rh-}\pi\text{ complex)}}$) at their transition state geometry and their geometry in the Rh-substrate π complexes (**21a**, **21b**, **23a**, and **23b**). The interaction energies ($\Delta E_{\text{int-space + bond}}$) were calculated from the change of interaction energies between the Rh-ligand complexes and the enyne substrate from the Rh-π complexes (**21a**, **21b**, **23a**, and **23b**) to the transition state structures.

Table S2. Distortion-Interaction calculation (including through-bond) of oxidative cyclization transition states

	**TS1a** leading to (2*R*,8*R*)-*trans*-**14d** (rel. to **21a**)	**TS1b** leading to (2*S*,8*R*)-*cis*-**14d** (rel. to **21b**)	**TS2a** leading to (2*R*,8*S*)-*cis*-**14d** (rel. to **23a**)	**TS2b** leading to (2*S*,8*S*)-*trans*-**14d** (rel. to **23b**)
$\Delta G^{\ddagger}$ (kcal/mol)	25.9	27.5	26.2	26.5
$\Delta H^{\ddagger}$ (kcal/mol)	15.5	16.0	15.4	15.0
$\Delta E_{\text{dist (subst. rel to Rh- }\pi\text{ complex)}}$ (kcal/mol)	27.4	25.7	30.2	28.9
$\Delta E_{\text{dist (RhCOLig)}}$ (kcal/mol)	4.5	1.3	5.3	4.2
$\Delta E_{\text{dist (total rel to to Rh- }\pi\text{ complex)}}$ (kcal/mol)	31.9	27.3	35.5	33.1
TS total $E_{\text{int-space + bond}}$ (kcal/mol)	-111.0	-116.9	-117.6	-115.3
Rh-π-complex total $E_{\text{int-space + bond}}$ (kcal/mol)	-83.9	-95.0	-86.8	-85.7
$\Delta E_{\text{int-space + bond}}$ (kcal/mol)	-27.1	-21.9	-29.3	-29.7

Non-covalent interaction surfaces of oxidative cyclization TS

The computed non-covalent interaction surfaces are plotted below. For **TS1a**, Figure SX A, the darker blue region circled in purple indicates a stabilizing π/π interaction between the furan group on the substrate and the backbone of the ligand. The blue region circled in blue indicates the C-H/π interaction between the C8 C-H and the backbone of the ligand (Figure S5 A). Conversely, **TS1b** indicates a stabilizing C-H/ π interaction with the top face of the binaptyl portion of the ligand backbone, but the hole in the NCI surface around the bottom face indicates the absence of a stabilizing interaction from the OMe group. Additionally, **TS1b** sees more yellow/red regions (indicated in red) around OMe group indicating more adverse intramolecular interactions (Figure S5 B).

The blue regions indicating stabilizing π/π interactions between the furan group of the substrate and the backbone of the ligand in **TS2a** are circled in purple (Figure S6 A). The adverse H-H repulsion between the OMe group and the C8-H is indicated by a yellow region in surface that has been indicated in red. Lastly, **TS2b** showed blue regions at both the bottom and top faces of the binapthyl portion of the ligand indicating the two stabilizing C-H/π interactions with the substrate. The adverse syn-pentane repulsion between the oxygen on the OMe group and methyl group on the allene is shown by the yellow region in the surface circled in red. Additionally, the adverse C-H steric interaction between the OMe H and the carbon on the furan is indicated by a yellow region in the surface between these atoms (Figure S6 B).

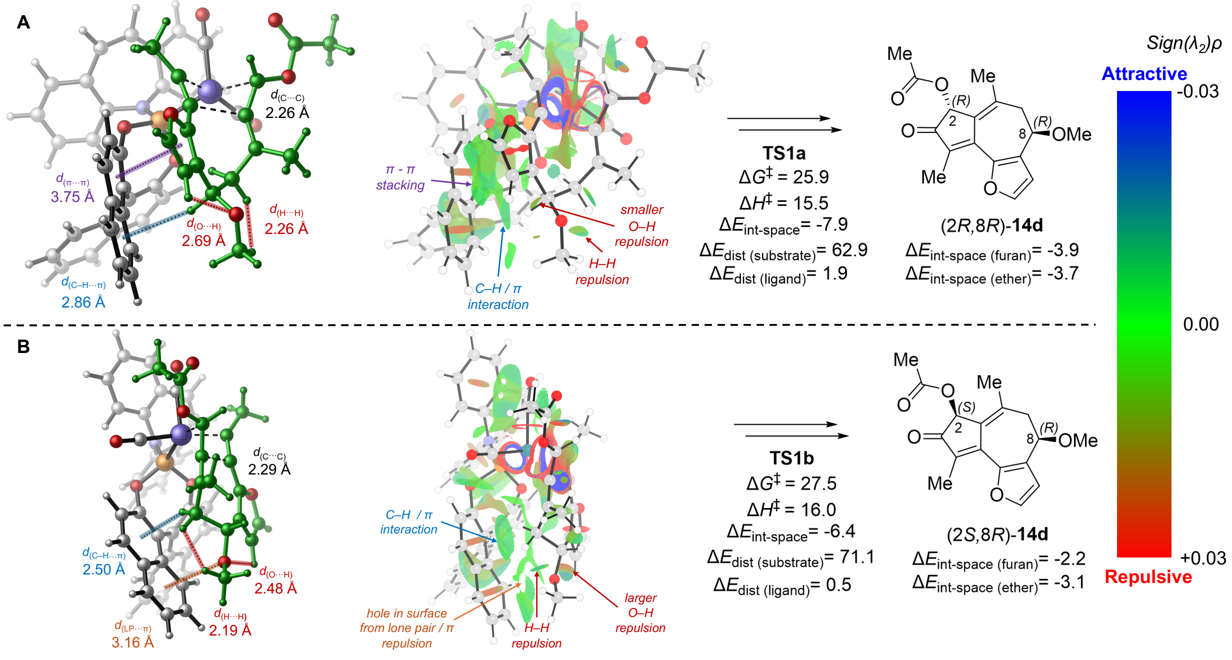

Figure S5. NCIPlot non-covalent interaction diagrams for **TS1a** and **TS1b**

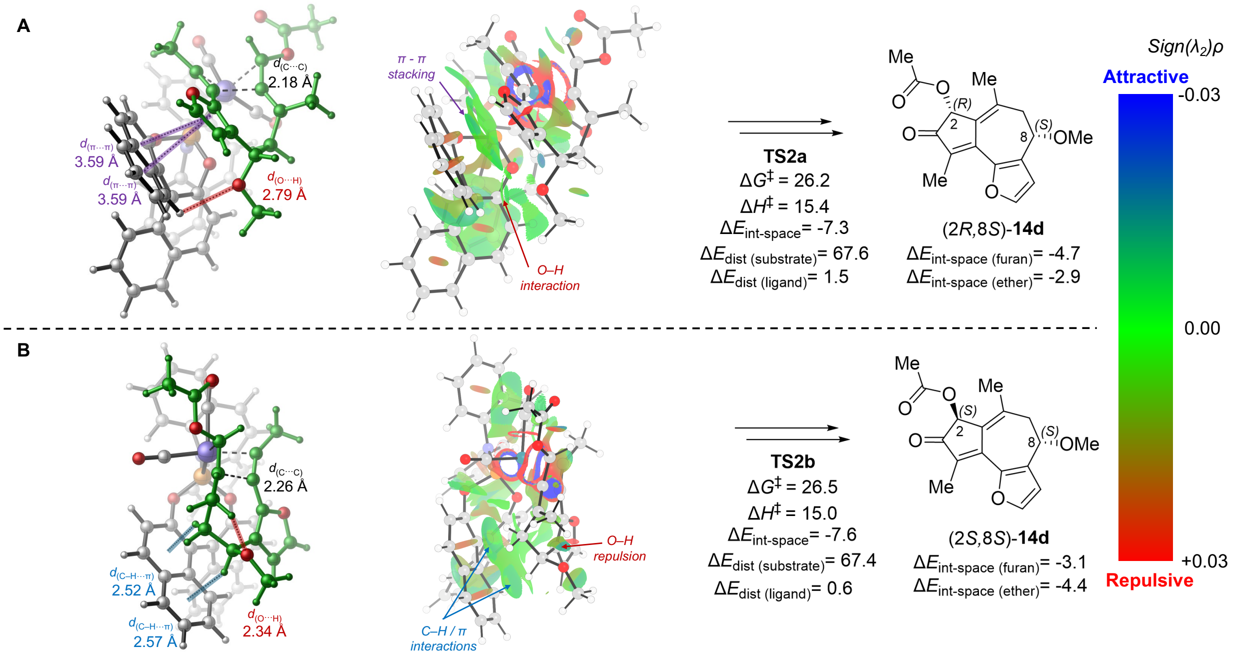

Figure S6. NCIPlot non-covalent intraction diagrams for **TS2a** and **TS2b**

Comparison of competing four-coordinate and CO-only oxidative cyclization pathways

We calculated competing pathways with a Rh tricarbonyl catalyst without the (*S*)-MonoPhos alkene ligand (namely, the "CO-only" pathway) and with a four-coordinated Rh complex involving one (*S*)-MonoPhos alkene and one CO ligand. The four-coordinated pathway (Figure S7) and the CO only pathway (Figure S8) are both disfavored by ca 10 and 17 kcal/mol respectively relative to the five-coordinated pathway with one (*S*)-MonoPhos alkene and two CO ligands on the Rh. In the reaction with (*R*)-**13d**, the CO-only pathway favors the formation of the *cis* diastereomer, (2*S*,8*R*)-*cis*-**14d**, rather than the *trans* diastereomer (2*R*,8*R*)-*trans*-**14d** that is favored in the five-coordinated pathway. This competing pathway could possibly contribute to the formation of the minor diastereomeric product of the reaction. (Note: T-shaped π/π conformer of the (*S*)-MonoPhos-alkene ligand was used for **TS3a** and **TS3b**)

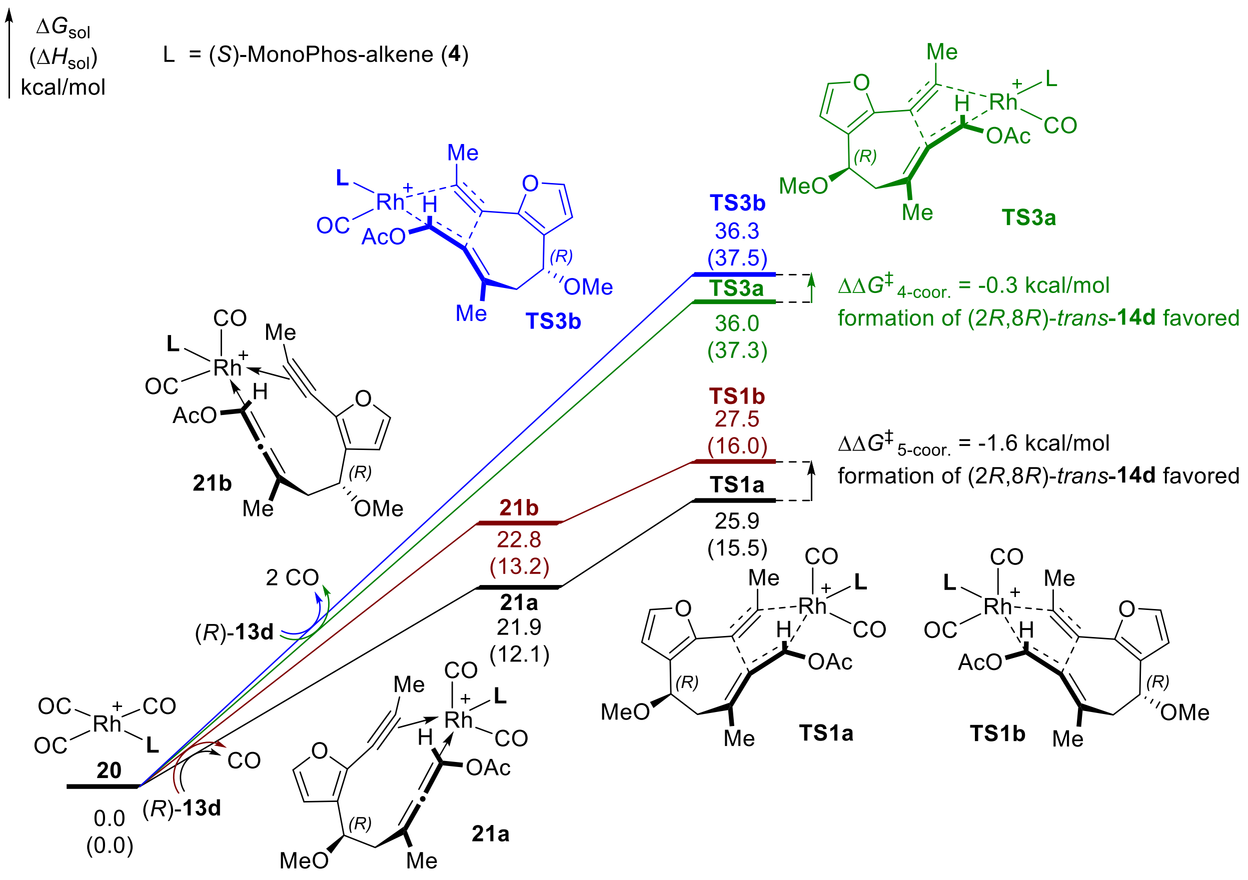

Figure S7. Comparison of four- (**TS3a** and **TS3b**) and five- (**TS1a** and **TS1b**) coordinated oxidative cyclization pathways.

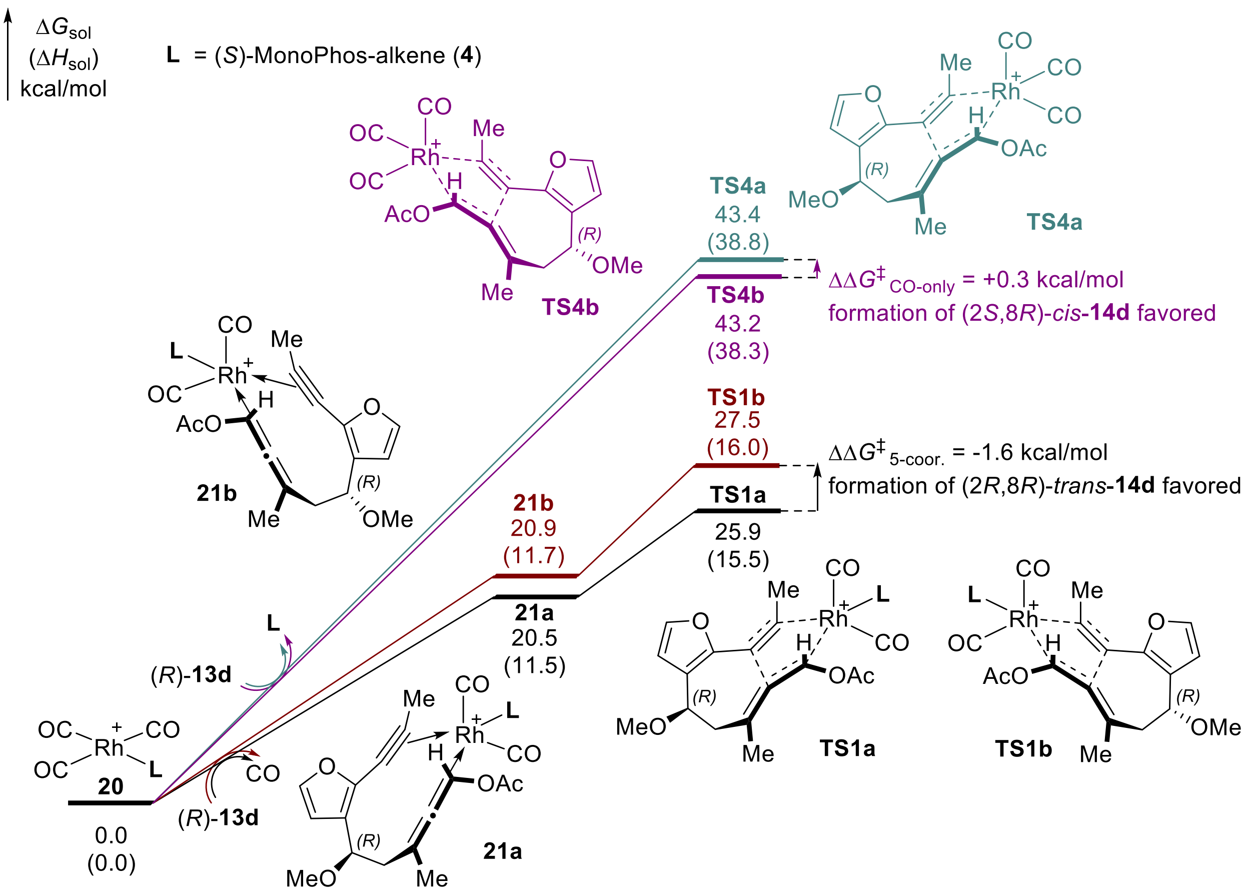

Figure S8. Comparison of CO-only (**TS4a** and **TS4b**) and five-coordinated (**TS1a** and **TS1b**) oxidative cyclization pathways.

Cartesian coordinates and energies of DFT-computed structures in the oxidative cyclization pathways

```
4
B3LYP-D3 SCF energy:                  -1855.51603982 a.u.
B3LYP-D3 enthalpy:                    -1855.021365 a.u.
B3LYP-D3 Gibbs free energy:           -1855.107270 a.u.
wB97X-D SCF energy in solution:       -1855.54644617 a.u.
wB97X-D enthalpy:                     -1855.051771 a.u.
wB97X-D Gibbs free energy:            -1855.137676 a.u.

Cartesian coordinates
ATOM       X           Y           Z
P        1.177354   -1.504381   -0.574487
```

O	0.142016	-1.469745	0.780598
O	0.270192	-0.530245	-1.592703
N	2.403592	-0.492211	0.053004
C	-1.166946	-1.866690	0.570545
C	-0.492603	0.552761	-1.183041
C	3.692879	-1.098822	0.221051
C	2.260726	0.883382	0.433998
C	-1.540355	-3.162892	0.992695
C	-2.065504	-0.987333	-0.016639
C	-1.612317	0.382830	-0.381734
C	-0.091512	1.819585	-1.663780
C	3.835784	-2.163399	1.115926
C	4.786999	-0.670249	-0.562408
C	2.963764	1.877386	-0.284340
C	1.380709	1.241850	1.460968
H	-0.787501	-3.785378	1.465388
C	-2.830587	-3.594576	0.808912
C	-3.398192	-1.457044	-0.283319
C	-2.272275	1.564344	0.108376
H	0.770343	1.866103	-2.319244
C	-0.766106	2.944794	-1.268552
H	2.980028	-2.452529	1.719225
C	5.048626	-2.845722	1.218733
C	5.998027	-1.376313	-0.445017
C	4.708004	0.475108	-1.467036
C	3.925426	1.570214	-1.342546
C	2.715540	3.225114	0.036398
C	1.147554	2.584554	1.752433
H	0.851964	0.459216	1.989561
H	-3.131425	-4.585075	1.140929
C	-3.783867	-2.768454	0.155815
C	-4.354740	-0.686474	-1.002331
C	-1.847314	2.854818	-0.351589
C	-3.309194	1.512710	1.081380
H	-0.453257	3.923673	-1.621846
H	5.142756	-3.682595	1.904815
C	6.131972	-2.450989	0.430772
H	6.844456	-1.066199	-1.053129
H	5.437297	0.476162	-2.275557
H	4.084993	2.372347	-2.061914
H	3.241628	3.998517	-0.518471
C	1.816568	3.580319	1.036515
H	0.431689	2.850314	2.524577
C	-5.106132	-3.226134	-0.092190
C	-5.624619	-1.165417	-1.237222
H	-4.070723	0.290379	-1.375532
C	-2.494706	4.019329	0.139549
C	-3.907872	2.662572	1.548710
H	-3.621663	0.549374	1.467370
H	7.079385	-2.978255	0.499119
H	1.633831	4.628645	1.255325
H	-5.384755	-4.218067	0.256133
C	-6.012977	-2.443214	-0.768852
H	-6.333525	-0.557683	-1.793060
H	-2.163537	4.988651	-0.226285

C	-3.506949	3.931423	1.068329
H	-4.691977	2.593397	2.297946
H	-7.019675	-2.806574	-0.956022
H	-3.990331	4.830466	1.440723

13d

B3LYP-D3 SCF energy: -921.31542498 a.u.
B3LYP-D3 enthalpy: -920.985641 a.u.
B3LYP-D3 Gibbs free energy: -921.062319 a.u.
wB97X-D SCF energy in solution: -921.36238699 a.u.
wB97X-D enthalpy: -921.032603 a.u.
wB97X-D Gibbs free energy: -921.109281 a.u.

Cartesian coordinates

ATOM	X	Y	Z
C	-0.096520	2.641997	0.523473
C	-1.411550	3.128105	0.924978
C	0.998991	2.243270	0.184388
C	2.217378	1.647933	-0.195089
C	0.753105	-1.237230	-0.940530
C	-0.368490	-2.140093	-0.446088
C	-1.476288	-1.588048	-0.011888
C	-2.522703	-0.957340	0.448022
H	-2.628544	-0.589533	1.463249
O	-3.639151	-0.726397	-0.374975
C	-4.444263	0.316598	-0.044518
O	-4.225435	1.083035	0.869214
C	2.589615	0.323176	-0.208145
C	-0.111567	-3.628409	-0.418918
H	0.057776	-4.000220	-1.438319
H	0.802665	-3.824432	0.151903
H	-0.946411	-4.180576	0.020609
C	3.920014	0.283287	-0.729795
O	3.247656	2.425031	-0.675357
C	4.265288	1.577535	-0.987801
H	5.158620	2.044096	-1.375115
H	4.524626	-0.600266	-0.876338
H	-2.141962	2.310969	0.965660
H	-1.379176	3.599673	1.915541
H	-1.788199	3.873910	0.213684
C	1.705152	-0.823549	0.200584
H	1.078533	-0.495026	1.045833
H	1.343761	-1.738976	-1.716733
H	0.333482	-0.323093	-1.368758
O	2.456168	-1.974198	0.572721
C	3.090061	-1.853038	1.835398
H	3.624553	-2.790117	2.012400
H	3.806910	-1.019836	1.855755
H	2.353866	-1.701081	2.640263
C	-5.633241	0.372907	-0.972377
H	-6.219009	-0.548164	-0.884734
H	-6.251629	1.233999	-0.717228
H	-5.293660	0.446301	-2.010658

CO

```
B3LYP-D3 SCF energy:                 -113.30691325 a.u.
B3LYP-D3 enthalpy:                   -113.298571 a.u.
B3LYP-D3 Gibbs free energy:          -113.321014 a.u.
wB97X-D SCF energy in solution:      -113.31556960 a.u.
wB97X-D enthalpy:                    -113.307227 a.u.
wB97X-D Gibbs free energy:           -113.329670 a.u.
```

Cartesian coordinates

ATOM	X	Y	Z
C	0.000000	0.000000	-0.650136
O	0.000000	0.000000	0.487602

20

```
B3LYP-D3 SCF energy:                 -2304.91682235 a.u.
B3LYP-D3 enthalpy:                   -2304.385894 a.u.
B3LYP-D3 Gibbs free energy:          -2304.495842 a.u.
wB97X-D SCF energy in solution:      -2306.11492050 a.u.
wB97X-D enthalpy:                    -2305.583992 a.u.
wB97X-D Gibbs free energy:           -2305.693940 a.u.
```

Cartesian coordinates

ATOM	X	Y	Z
Rh	2.268486	-1.746411	-0.554225
P	0.752928	-0.055725	0.151029
O	-0.271119	-0.394031	1.379994
O	-0.235723	0.275269	-1.096211
N	1.549473	1.305034	0.664950
C	-1.261638	-1.370568	1.124682
C	-1.477245	0.935684	-0.930258
C	2.967521	1.205755	0.910735
C	0.938914	2.609713	0.630090
C	-1.096298	-2.623666	1.748168
C	-2.334243	-1.053247	0.310215
C	-2.494480	0.330538	-0.216285
C	-1.596366	2.188242	-1.559248
C	3.416199	0.598164	2.086370
C	3.869747	1.661311	-0.072664
C	1.317492	3.502554	-0.395112
C	-0.058775	2.930082	1.550340
H	-0.266912	-2.769613	2.430668
C	-2.019289	-3.613348	1.507402
C	-3.258651	-2.109482	-0.009167
C	-3.687736	1.104335	0.013688
H	-0.758794	2.570516	-2.129107
C	-2.762016	2.896307	-1.409172
H	2.686504	0.296969	2.832568
C	4.780771	0.381449	2.281867
C	5.239649	1.416094	0.141339
C	3.435172	2.395246	-1.260315

C	2.349762	3.196164	-1.385715
C	0.637196	4.733294	-0.463032
C	-0.716605	4.155911	1.456941
H	-0.328504	2.204140	2.309865
H	-1.922434	-4.580391	1.992919
C	-3.099182	-3.395053	0.611745
C	-4.312646	-1.944123	-0.949261
C	-3.818607	2.393563	-0.604093
C	-4.734819	0.659972	0.866781
H	-2.874388	3.866713	-1.884009
H	5.129969	-0.096590	3.191904
C	5.689925	0.782650	1.297668
H	5.955209	1.747911	-0.606249
H	4.135297	2.382123	-2.093466
H	2.266626	3.758175	-2.314203
H	0.906584	5.439252	-1.244156
C	-0.363585	5.057712	0.448822
H	-1.506448	4.399803	2.160248
C	-4.019629	-4.434520	0.308803
C	-5.177863	-2.978129	-1.231320
H	-4.429510	-0.992721	-1.454204
C	-4.996449	3.156292	-0.382521
C	-5.857925	1.431366	1.071288
H	-4.641836	-0.295100	1.370029
H	6.753260	0.614569	1.439425
H	-0.872448	6.013792	0.372005
H	-3.894679	-5.398238	0.795512
C	-5.041306	-4.232309	-0.589818
H	-5.972099	-2.830205	-1.957116
H	-5.086353	4.125658	-0.865994
C	-5.999369	2.686666	0.434067
H	-6.641468	1.073351	1.732680
H	-5.737040	-5.034292	-0.817343
H	-6.894067	3.279354	0.599355
C	2.343440	-2.410823	1.284119
O	2.400435	-2.784001	2.358500
C	2.125547	-0.934426	-2.343001
O	2.065170	-0.455339	-3.371895
C	3.491940	-3.142333	-1.194472
O	4.198752	-3.952935	-1.567806

21a

B3LYP-D3 SCF energy: -3112.93405547 a.u.
B3LYP-D3 enthalpy: -3112.082842 a.u.
B3LYP-D3 Gibbs free energy: -3112.232697 a.u.
wB97X-D SCF energy in solution: -3114.14221684 a.u.
wB97X-D enthalpy: -3113.291003 a.u.
wB97X-D Gibbs free energy: -3113.440858 a.u.

Cartesian coordinates

ATOM	X	Y	Z
C	1.220768	-1.734880	1.441620
C	1.902713	-0.940126	2.129692

C	0.421902	-2.913722	1.396685
C	-0.362575	-3.626163	0.510597
C	0.338737	-2.747840	-1.836903
H	0.089854	-3.007261	-2.872641
C	1.739723	-3.196978	-1.519973
C	2.584266	-2.413743	-0.871730
C	3.775201	-2.091128	-0.269983
H	4.013068	-2.357171	0.756229
O	4.870998	-1.799966	-1.074805
C	5.996452	-1.324620	-0.429216
O	6.014507	-1.105323	0.756920
H	0.256446	-1.667471	-1.741556
C	-0.740805	-3.405458	-0.935479
Rh	2.267146	-0.369383	-0.030857
C	3.721788	0.888676	0.744607
O	4.568067	1.495249	1.207653
C	2.115277	-4.615985	-1.876194
H	3.118568	-4.879273	-1.533057
H	1.382759	-5.301447	-1.434673
H	2.065185	-4.752348	-2.963589
C	2.402276	0.047094	-1.910977
O	2.499070	0.287279	-3.025699
P	0.247920	0.956430	0.030920
O	-0.827635	0.806579	1.252582
O	-0.589080	0.401656	-1.288609
N	0.317401	2.636875	0.013075
C	-1.788235	-0.201076	1.341532
C	-1.857930	0.915673	-1.577408
C	-0.603391	3.470084	0.758479
C	1.502620	3.272909	-0.489805
C	-1.741223	-0.986799	2.513169
C	-2.764901	-0.355402	0.371023
C	-2.920787	0.619443	-0.745101
C	-1.970025	1.725105	-2.726189
C	-1.861624	3.763730	0.237510
C	-0.223771	3.919817	2.039497
C	2.463435	3.765689	0.419837
C	1.709501	3.346631	-1.869325
H	-0.980414	-0.764614	3.252135
C	-2.634992	-2.013118	2.676707
C	-3.631812	-1.502432	0.481536
C	-4.162238	1.308441	-0.983549
H	-1.097883	1.860676	-3.356842
C	-3.177763	2.314130	-3.013615
H	-2.113306	3.432406	-0.760614
C	-2.792170	4.459086	1.008584
C	-1.184667	4.606032	2.805394
C	1.127002	3.751100	2.570052
C	2.286656	3.706512	1.870794
C	3.650342	4.302546	-0.115141
C	2.891831	3.886122	-2.374089
H	0.936154	2.973969	-2.533545
H	-2.605946	-2.625717	3.572462
C	-3.564117	-2.330639	1.650147
C	-4.528081	-1.880859	-0.556709

C	-4.285468	2.155074	-2.137369
C	-5.264803	1.229238	-0.089195
H	-3.287770	2.936838	-3.897032
H	-3.779110	4.660704	0.603169
C	-2.455731	4.868266	2.301822
H	-0.914954	4.949026	3.800825
H	1.208588	3.796642	3.654729
H	3.211340	3.716498	2.444594
H	4.410940	4.673183	0.566610
C	3.864612	4.363195	-1.489264
H	3.053436	3.931686	-3.446695
C	-4.403681	-3.471643	1.746740
C	-5.317282	-3.005142	-0.438996
H	-4.579651	-1.281172	-1.457954
C	-5.506400	2.844276	-2.369500
C	-6.427833	1.925584	-0.334593
H	-5.179509	0.624086	0.805417
H	-3.179720	5.398699	2.913111
H	4.788350	4.786491	-1.872329
H	-4.341253	-4.086658	2.640655
C	-5.263413	-3.807045	0.725773
H	-5.988052	-3.277412	-1.248912
H	-5.588533	3.473497	-3.251966
C	-6.558474	2.732780	-1.489765
H	-7.252545	1.857662	0.368890
H	-5.895416	-4.686282	0.807276
H	-7.484648	3.269223	-1.672957
C	2.387101	-0.217398	3.316272
H	3.479629	-0.245993	3.389066
H	2.073919	0.833034	3.285741
H	1.964971	-0.685755	4.213190
O	0.385237	-3.525651	2.636826
C	-0.901999	-4.720864	1.261155
C	-0.422931	-4.609381	2.528759
H	-1.558126	-5.480294	0.867369
H	-0.541967	-5.198506	3.425827
C	7.118197	-1.130378	-1.409279
H	7.998626	-0.762101	-0.882568
H	7.346452	-2.076958	-1.909757
H	6.816059	-0.416675	-2.183366
O	-1.114840	-4.683082	-1.445601
C	-2.137453	-4.635949	-2.429229
H	-1.832826	-4.066537	-3.320583
H	-2.332202	-5.669039	-2.725861
H	-3.058024	-4.191494	-2.023599
H	-1.618187	-2.741220	-0.954560

21b

B3LYP-D3 SCF energy: -3112.93737322 a.u.
B3LYP-D3 enthalpy: -3112.085692 a.u.
B3LYP-D3 Gibbs free energy: -3112.235258 a.u.
wB97X-D SCF energy in solution: -3114.14233598 a.u.
wB97X-D enthalpy: -3113.290655 a.u.

wB97X-D Gibbs free energy: -3113.440221 a.u.

Cartesian coordinates

ATOM	X	Y	Z
C	-1.395473	-1.360943	2.357344
C	-2.555650	-0.948082	2.106848
C	-0.087347	-1.559874	2.740918
C	0.944606	-2.441528	2.404741
C	0.483224	-3.222446	0.013429
H	1.122899	-3.762431	-0.690927
C	-0.957049	-3.579688	-0.227980
C	-1.932024	-2.684371	-0.294226
C	-3.318214	-2.522664	-0.255530
H	-3.882822	-2.733705	0.648390
O	-4.076041	-2.678802	-1.424584
C	-5.373433	-2.228979	-1.351150
O	-5.819247	-1.697554	-0.360726
H	0.654708	-2.158968	-0.150281
C	0.953747	-3.611150	1.452116
H	0.285459	-4.388618	1.859032
Rh	-2.290862	-0.592182	-0.084074
C	-3.882902	0.706364	0.063980
O	-4.891335	1.237795	0.050526
C	-1.276632	-5.053258	-0.373127
H	-2.351789	-5.234291	-0.447512
H	-0.883904	-5.635179	0.470303
H	-0.799327	-5.447281	-1.280137
C	-2.241036	-0.508979	-2.001632
O	-2.279305	-0.457470	-3.143563
P	-0.341107	0.940346	-0.106620
O	0.570640	0.956588	-1.485120
O	0.766682	0.460049	1.024226
N	-0.594613	2.601622	0.015105
C	1.412613	-0.126876	-1.752162
C	2.058102	0.987780	1.053150
C	0.068848	3.597181	-0.799953
C	-1.636937	3.025416	0.908186
C	1.060684	-0.957269	-2.838195
C	2.551543	-0.336095	-0.988793
C	2.951729	0.646131	0.056068
C	2.389285	1.839804	2.128048
C	1.420681	3.880065	-0.608587
C	-0.656881	4.225632	-1.832753
C	-2.805169	3.628854	0.391634
C	-1.522833	2.743266	2.273875
H	0.202494	-0.690550	-3.442317
C	1.821887	-2.065968	-3.117262
C	3.296038	-1.546630	-1.211810
C	4.238499	1.288275	0.055922
H	1.642607	2.040234	2.887801
C	3.641631	2.403449	2.175823
H	1.953427	3.407184	0.204037
C	2.087796	4.748105	-1.471924
C	0.040672	5.086478	-2.700716
C	-2.100529	4.075836	-2.000674

C	-3.022944	3.854055	-1.036809
C	-3.830672	3.951726	1.301077
C	-2.561711	3.056368	3.150384
H	-0.624339	2.258204	2.639278
H	1.565465	-2.706235	-3.956914
C	2.926460	-2.412475	-2.294950
C	4.354708	-1.957060	-0.356051
C	4.583299	2.167324	1.138073
C	5.177740	1.120558	-0.998123
H	3.914353	3.062567	2.995559
H	3.145597	4.942719	-1.322370
C	1.397639	5.343600	-2.529963
H	-0.504323	5.568483	-3.508129
H	-2.475508	4.296479	-2.998426
H	-4.069157	3.912735	-1.331478
H	-4.739574	4.409305	0.920079
C	-3.716419	3.673509	2.660015
H	-2.466287	2.825017	4.207148
C	3.648284	-3.619245	-2.500337
C	5.022319	-3.142041	-0.571351
H	4.624591	-1.332677	0.487395
C	5.853353	2.804480	1.141095
C	6.395779	1.763641	-0.970137
H	4.923443	0.485635	-1.838613
H	1.912521	6.011303	-3.214261
H	-4.527588	3.931433	3.334349
H	3.366501	-4.258498	-3.333325
C	4.676229	-3.979257	-1.658512
H	5.820720	-3.439053	0.102528
H	6.103761	3.461663	1.969963
C	6.744823	2.606609	0.111776
H	7.094286	1.626246	-1.790428
H	5.219330	-4.905366	-1.822808
H	7.710899	3.102342	0.121187
C	-3.902941	-0.804040	2.730071
H	-4.089213	0.263238	2.901249
H	-4.694027	-1.172982	2.070954
H	-3.948547	-1.326805	3.690482
C	2.090514	-1.996187	3.104341
O	0.417688	-0.596797	3.618160
C	1.706025	-0.895075	3.826340
H	2.237060	-0.254675	4.515594
H	3.068180	-2.448130	3.061151
C	-6.095987	-2.480096	-2.645013
H	-7.129608	-2.145975	-2.554316
H	-5.597441	-1.939747	-3.457340
H	-6.063488	-3.545493	-2.894221
O	2.287106	-4.091875	1.449323
C	2.433478	-5.427693	0.999863
H	2.114473	-5.553603	-0.044306
H	3.498119	-5.659778	1.064721
H	1.867528	-6.127780	1.633481

22a

B3LYP-D3 SCF energy: -3112.99851923 a.u.
B3LYP-D3 enthalpy: -3112.144845 a.u.
B3LYP-D3 Gibbs free energy: -3112.290351 a.u.
wB97X-D SCF energy in solution: -3114.20921827 a.u.
wB97X-D enthalpy: -3113.355544 a.u.
wB97X-D Gibbs free energy: -3113.501050 a.u.

Cartesian coordinates

ATOM	X	Y	Z
C	-2.192870	1.752658	1.190977
C	-2.015224	0.524320	1.715346
C	-1.892821	3.075521	1.687700
C	-1.349678	4.145176	1.012584
C	-0.553127	2.749302	-0.965875
H	0.065025	2.857252	-1.859630
C	-1.796747	1.959469	-1.299879
C	-2.631690	1.588502	-0.250284
C	-3.843082	0.795045	-0.312534
H	-4.472580	0.762953	0.570681
O	-4.545936	0.686618	-1.497048
C	-5.569325	-0.252077	-1.514527
O	-5.776250	-0.984202	-0.581173
H	0.040471	2.236504	-0.210761
C	-0.910473	4.163484	-0.433509
H	0.000281	4.780569	-0.494866
Rh	-2.190361	-0.619599	-0.009611
C	-3.206535	-1.933572	0.951007
O	-3.827484	-2.646916	1.593195
C	-2.108151	1.852042	-2.759012
H	-3.008567	1.288318	-2.986507
H	-2.217131	2.878225	-3.134865
H	-1.250158	1.411592	-3.281294
C	-2.353292	-1.406098	-1.878493
O	-2.507886	-1.772577	-2.947455
P	0.191922	-1.155553	0.098367
O	1.219593	-0.523912	1.216448
O	0.739710	-0.482265	-1.321427
N	0.697695	-2.754104	0.219748
C	1.678773	0.791245	1.137041
C	2.105032	-0.551007	-1.626510
C	1.837102	-3.166102	1.007710
C	-0.260712	-3.767836	-0.127567
C	1.275452	1.657898	2.174773
C	2.511695	1.190228	0.103838
C	3.005635	0.207824	-0.901788
C	2.487200	-1.414801	-2.673142
C	3.119745	-3.101693	0.467560
C	1.625742	-3.558668	2.345673
C	-1.052017	-4.348701	0.886897
C	-0.447771	-4.101354	-1.470552
H	0.680109	1.257911	2.984369
C	1.637675	2.980217	2.131669
C	2.826059	2.592099	0.005952
C	4.406781	-0.002893	-1.143423

H	1.715361	-1.940629	-3.225100
C	3.822242	-1.565955	-2.963383
H	3.245511	-2.803816	-0.565518
C	4.226143	-3.393969	1.264003
C	2.759386	-3.836051	3.132625
C	0.293699	-3.705349	2.929644
C	-0.858366	-4.050483	2.305167
C	-2.069903	-5.237560	0.489956
C	-1.453543	-4.995185	-1.837606
H	0.196782	-3.644021	-2.214997
H	1.315821	3.659775	2.914488
C	2.379819	3.489435	1.033689
C	3.511258	3.143932	-1.112252
C	4.810920	-0.897181	-2.192120
C	5.418355	0.607697	-0.352497
H	4.136753	-2.223936	-3.768746
H	5.224332	-3.321967	0.842661
C	4.043771	-3.754492	2.602160
H	2.619534	-4.130373	4.169502
H	0.257252	-3.630278	4.015076
H	-1.729315	-4.224153	2.934102
H	-2.705061	-5.681386	1.251687
C	-2.270955	-5.556199	-0.850534
H	-1.603992	-5.244967	-2.883450
C	2.657690	4.878290	0.918742
C	3.750796	4.498029	-1.200975
H	3.837222	2.487865	-1.910736
C	6.195702	-1.113694	-2.426859
C	6.751702	0.364401	-0.598116
H	5.130701	1.262467	0.461864
H	4.901906	-3.973809	3.230448
H	-3.061132	-6.247934	-1.126743
H	2.320082	5.540857	1.711647
C	3.328512	5.376883	-0.174897
H	4.267802	4.895767	-2.069402
H	6.488372	-1.786396	-3.228930
C	7.148523	-0.496337	-1.649522
H	7.507228	0.834700	0.024526
H	3.532355	6.440499	-0.255819
H	8.203923	-0.673052	-1.834116
C	-1.709385	0.074462	3.100846
H	-0.848525	-0.603592	3.118036
H	-1.520330	0.930461	3.758505
H	-2.559783	-0.483086	3.514863
C	-1.134670	5.160997	2.003797
O	-2.002486	3.360982	3.019994
C	-1.544400	4.638090	3.193168
H	-1.596033	5.020912	4.200985
H	-0.714683	6.144370	1.842586
O	-1.890598	4.674260	-1.336507
C	-2.204373	6.045590	-1.148181
H	-2.895822	6.321979	-1.947203
H	-2.685764	6.224086	-0.177085
H	-1.303182	6.674596	-1.219483
C	-6.288604	-0.212333	-2.831549

H	-7.130276	-0.904434	-2.804721
H	-6.637321	0.803169	-3.043132
H	-5.599113	-0.498376	-3.634022

22b

B3LYP-D3 SCF energy: -3112.99217676 a.u.
B3LYP-D3 enthalpy: -3112.138837 a.u.
B3LYP-D3 Gibbs free energy: -3112.285718 a.u.
wB97X-D SCF energy in solution: -3114.20574706 a.u.
wB97X-D enthalpy: -3113.352407 a.u.
wB97X-D Gibbs free energy: -3113.499288 a.u.

Cartesian coordinates

ATOM	X	Y	Z
C	-2.066950	-2.001177	1.561398
C	-2.155650	-0.685119	1.819928
C	-1.582919	-3.104805	2.362950
C	-0.723363	-4.118893	2.011446
C	0.004969	-2.979455	-0.147817
H	0.748765	-3.096244	-0.937748
C	-1.326488	-2.637488	-0.757069
C	-2.351680	-2.230584	0.095580
C	-3.665550	-1.735423	-0.248834
H	-4.399094	-1.647112	0.545561
O	-4.216807	-2.007803	-1.486902
C	-5.383240	-1.321235	-1.800020
O	-5.834643	-0.471902	-1.075963
H	0.348613	-2.188626	0.522816
C	-0.036745	-4.309693	0.685793
H	-0.553763	-5.083861	0.091771
Rh	-2.340213	0.030842	-0.129084
C	-3.726493	1.219289	0.472427
O	-4.572041	1.854886	0.902323
C	-1.503215	-3.027983	-2.194177
H	-2.466664	-2.752104	-2.614500
H	-1.396125	-4.121392	-2.263398
H	-0.693201	-2.603998	-2.795419
C	-2.396121	0.405242	-2.122490
O	-2.436309	0.574700	-3.250587
P	-0.180021	1.106570	0.052259
O	0.797481	1.062910	-1.271989
O	0.678318	0.208369	1.147392
N	-0.032242	2.752247	0.366716
C	1.529338	-0.063592	-1.627920
C	2.027484	0.501380	1.376298
C	0.958024	3.571832	-0.301898
C	-1.164917	3.452702	0.912883
C	1.195270	-0.653547	-2.865915
C	2.575913	-0.509987	-0.836593
C	2.967112	0.229886	0.397236
C	2.349943	1.069465	2.625313
C	2.237023	3.698616	0.234039
C	0.620262	4.165494	-1.536081

C	-2.086966	4.065121	0.035613
C	-1.350402	3.490563	2.295868
H	0.399031	-0.207011	-3.452397
C	1.904613	-1.742940	-3.310656
C	3.252991	-1.713785	-1.244505
C	4.306358	0.711083	0.610847
H	1.561029	1.198118	3.358838
C	3.648053	1.444051	2.875484
H	2.460615	3.235482	1.186475
C	3.222955	4.388192	-0.471034
C	1.635438	4.844401	-2.234903
C	-0.730069	4.118756	-2.093857
C	-1.902365	4.080411	-1.415716
C	-3.215886	4.684944	0.603711
C	-2.474011	4.115774	2.835087
H	-0.604539	3.027923	2.934409
H	1.679141	-2.187307	-4.276513
C	2.929426	-2.313647	-2.509060
C	4.224608	-2.357896	-0.429303
C	4.643531	1.314667	1.869713
C	5.306276	0.663494	-0.398932
H	3.918876	1.876999	3.834463
H	4.224774	4.461735	-0.058751
C	2.921468	4.954283	-1.712775
H	1.399509	5.298694	-3.193686
H	-0.789184	4.240603	-3.173925
H	-2.816733	4.183065	-1.997884
H	-3.944292	5.153155	-0.052903
C	-3.409093	4.711189	1.981907
H	-2.619988	4.137779	3.910749
C	3.630336	-3.476227	-2.930995
C	4.875248	-3.492618	-0.861582
H	4.450503	-1.948932	0.548034
C	5.962992	1.798932	2.079597
C	6.570590	1.159500	-0.168750
H	5.061480	0.245944	-1.368374
H	3.687504	5.482880	-2.272287
H	-4.288430	5.197329	2.393328
H	3.388590	-3.906107	-3.899807
C	4.591677	-4.050541	-2.130949
H	5.612513	-3.965375	-0.219309
H	6.206678	2.243414	3.041123
C	6.910025	1.723062	1.084303
H	7.314048	1.122141	-0.959684
H	5.122691	-4.937574	-2.463411
H	7.913371	2.102558	1.252897
C	-1.994643	0.051501	3.102942
H	-1.078975	0.651704	3.081355
H	-2.827851	0.740827	3.280399
H	-1.935836	-0.652437	3.940074
C	-0.510989	-4.888149	3.202430
O	-1.902497	-3.195062	3.689163
C	-1.242276	-4.288166	4.180560
H	-1.402688	-4.497887	5.227006
H	0.131436	-5.750263	3.301595

C	-5.915885	-1.778438	-3.127129
H	-6.852178	-1.261669	-3.337856
H	-5.185109	-1.559119	-3.913691
H	-6.074954	-2.861348	-3.117645
O	1.283175	-4.738151	0.962159
C	1.900110	-5.472000	-0.084697
H	2.022592	-4.876749	-0.999363
H	2.892511	-5.749873	0.275220
H	1.330805	-6.383771	-0.322635

23a

B3LYP-D3 SCF energy:	-3112.93521726 a.u.
B3LYP-D3 enthalpy:	-3112.083538 a.u.
B3LYP-D3 Gibbs free energy:	-3112.230668 a.u.
wB97X-D SCF energy in solution:	-3114.14452758 a.u.
wB97X-D enthalpy:	-3113.292848 a.u.
wB97X-D Gibbs free energy:	-3113.439978 a.u.

Cartesian coordinates

ATOM	X	Y	Z
C	-0.931813	-2.283419	-0.765369
C	-1.440198	-1.910409	-1.846108
C	0.037866	-3.008347	-0.024400
C	0.260117	-3.475638	1.258289
C	-1.605368	-2.322939	2.672411
H	-1.853673	-2.247769	3.738461
C	-2.845035	-2.692939	1.897784
C	-3.194291	-2.069571	0.785965
C	-4.015857	-1.886081	-0.304412
H	-3.928761	-2.486388	-1.204310
O	-5.263265	-1.288200	-0.130667
C	-5.882870	-0.852771	-1.280405
O	-5.356967	-0.929758	-2.366297
H	-1.222027	-1.357517	2.336795
C	-0.496957	-3.393128	2.571188
Rh	-2.269433	-0.432810	-0.345096
C	-2.854970	0.654610	-2.016518
O	-3.170838	1.184346	-2.974449
C	-3.700008	-3.827220	2.421444
H	-4.570665	-4.006348	1.786188
H	-3.123146	-4.757302	2.487270
H	-4.049569	-3.591844	3.434449
C	-3.148620	0.537286	1.081086
O	-3.808970	0.972558	1.907371
P	-0.197331	0.748694	-0.066346
O	0.788758	0.786296	-1.368897
O	0.695029	-0.057361	1.067240
N	-0.188669	2.401935	0.258343
C	1.716021	-0.206318	-1.685242
C	1.986817	0.410441	1.351306
C	0.661859	3.331500	-0.455167
C	-1.262805	2.992798	1.005945
C	1.548282	-0.814822	-2.946320

C	2.770358	-0.501761	-0.838085
C	2.995642	0.271587	0.414707
C	2.177439	1.021806	2.608060
C	1.981390	3.524631	-0.052641
C	0.140012	3.991539	-1.586394
C	-2.325019	3.618121	0.313094
C	-1.245665	2.937578	2.402100
H	0.735024	-0.474559	-3.576955
C	2.419897	-1.794508	-3.346126
C	3.635773	-1.592159	-1.210323
C	4.248592	0.928827	0.686256
H	1.349856	1.041875	3.307990
C	3.402197	1.562159	2.916564
H	2.340397	3.031190	0.840070
C	2.829985	4.330960	-0.809375
C	1.020401	4.786464	-2.344790
C	-1.269751	3.922418	-1.962328
C	-2.336468	3.772898	-1.140246
C	-3.392190	4.126037	1.078454
C	-2.303441	3.468132	3.137093
H	-0.394722	2.477874	2.893255
H	2.307460	-2.265608	-4.318608
C	3.458706	-2.231773	-2.482114
C	4.640546	-2.099927	-0.340926
C	4.450900	1.564662	1.957823
C	5.283881	1.028117	-0.283595
H	3.568344	2.027256	3.884211
H	3.864097	4.456091	-0.502720
C	2.349883	4.951005	-1.966921
H	0.641693	5.291834	-3.229321
H	-1.482745	4.132619	-3.008798
H	-3.323176	3.880895	-1.586746
H	-4.225547	4.600145	0.566555
C	-3.383091	4.055206	2.468479
H	-2.292279	3.416855	4.221439
C	4.303234	-3.315798	-2.841955
C	5.437177	-3.160265	-0.715219
H	4.768173	-1.655784	0.638843
C	5.684328	2.218225	2.224020
C	6.459728	1.687908	-0.000804
H	5.135906	0.590541	-1.263432
H	3.009065	5.569844	-2.568367
H	-4.215278	4.463306	3.034081
H	4.160022	-3.786159	-3.811406
C	5.275771	-3.773246	-1.980478
H	6.191563	-3.533766	-0.028846
H	5.826747	2.684369	3.195521
C	6.671825	2.278313	1.267831
H	7.231250	1.759114	-0.761927
H	5.913751	-4.604484	-2.265650
H	7.607878	2.786829	1.478372
C	-1.879540	-2.016035	-3.248663
H	-2.972829	-2.027721	-3.320399
H	-1.523537	-1.166740	-3.841410
H	-1.479292	-2.938398	-3.684184

O	1.071030	-3.427340	-0.846915
C	1.488343	-4.211246	1.195091
C	1.929436	-4.147140	-0.090597
H	1.963031	-4.701885	2.031695
H	2.802055	-4.523312	-0.603094
C	-7.238741	-0.286570	-0.968111
H	-7.710093	0.055329	-1.889599
H	-7.860799	-1.048863	-0.487798
H	-7.140363	0.545320	-0.262353
O	0.415085	-3.295781	3.661371
C	1.181446	-2.100693	3.726099
H	0.552870	-1.217591	3.904464
H	1.862622	-2.222857	4.571169
H	1.765952	-1.936462	2.812400
H	-0.953920	-4.376544	2.733977

23b

B3LYP-D3 SCF energy: -3112.93514647 a.u.
B3LYP-D3 enthalpy: -3112.083332 a.u.
B3LYP-D3 Gibbs free energy: -3112.233239 a.u.
wB97X-D SCF energy in solution: -3114.14141162 a.u.
wB97X-D enthalpy: -3113.289597 a.u.
wB97X-D Gibbs free energy: -3113.439504 a.u.

Cartesian coordinates

ATOM	X	Y	Z
C	-1.307013	-1.445291	1.991372
C	-2.370358	-0.812927	2.141054
C	0.005406	-1.885038	2.257503
C	0.957421	-2.734396	1.713881
C	0.113511	-3.396949	-0.664302
H	0.502451	-4.011454	-1.483699
C	-1.369789	-3.638708	-0.548205
C	-2.243385	-2.656186	-0.405317
C	-3.547137	-2.266097	-0.165835
H	-3.998804	-2.362703	0.816256
O	-4.472347	-2.242207	-1.210446
C	-5.714102	-1.732396	-0.894710
O	-5.972781	-1.287797	0.198842
H	0.306154	-2.352928	-0.916635
C	0.954501	-3.741609	0.577215
Rh	-2.172070	-0.501343	-0.114840
C	-3.742212	0.884322	0.009341
O	-4.716377	1.436330	-0.205527
C	-1.881397	-5.061516	-0.638383
H	-2.973653	-5.099232	-0.635148
H	-1.486527	-5.649478	0.193311
H	-1.519746	-5.524674	-1.565121
C	-2.081524	-0.518143	-2.023795
O	-2.093433	-0.537983	-3.168717
P	-0.255558	1.016165	-0.122815
O	0.700728	1.060183	-1.464980
O	0.768332	0.466819	1.041845

N	-0.469842	2.675676	0.041775
C	1.514973	-0.035685	-1.758834
C	2.087229	0.903823	1.145960
C	0.270372	3.668238	-0.707865
C	-1.519268	3.135923	0.907218
C	1.206061	-0.761403	-2.929913
C	2.593560	-0.347118	-0.945141
C	2.998315	0.558123	0.166170
C	2.426217	1.660934	2.286445
C	1.619664	3.895458	-0.438528
C	-0.383764	4.354196	-1.751217
C	-2.645407	3.783670	0.353531
C	-1.443548	2.875560	2.279131
H	0.404142	-0.406642	-3.565794
C	1.926261	-1.890270	-3.234940
C	3.286520	-1.580770	-1.202541
C	4.320078	1.119115	0.251183
H	1.666336	1.844389	3.037664
C	3.707135	2.141334	2.413601
H	2.094640	3.375159	0.382420
C	2.355856	4.769522	-1.236759
C	0.383964	5.219554	-2.553151
C	-1.821382	4.252008	-1.992952
C	-2.795254	4.032634	-1.079849
C	-3.684730	4.149610	1.230569
C	-2.487852	3.246951	3.125861
H	-0.565993	2.372939	2.670935
H	1.699088	-2.457249	-4.133391
C	2.940310	-2.358858	-2.357640
C	4.273724	-2.095916	-0.316213
C	4.672342	1.911666	1.396062
C	5.290534	0.954205	-0.774920
H	3.988594	2.725452	3.285507
H	3.410475	4.924423	-1.029730
C	1.737974	5.423976	-2.305472
H	-0.103206	5.746973	-3.369050
H	-2.140769	4.506178	-3.001979
H	-3.824626	4.126077	-1.420787
H	-4.565220	4.637359	0.821380
C	-3.611566	3.889541	2.595898
H	-2.422813	3.039090	4.189817
C	3.596525	-3.599122	-2.584396
C	4.878064	-3.310881	-0.556256
H	4.536000	-1.527549	0.568101
C	5.977069	2.467417	1.484433
C	6.542687	1.517740	-0.663066
H	5.033855	0.386991	-1.661917
H	2.308051	6.097186	-2.938688
H	-4.428761	4.186009	3.246613
H	3.328379	-4.175408	-3.466182
C	4.541766	-4.071504	-1.702446
H	5.622150	-3.688606	0.139382
H	6.232072	3.058878	2.360057
C	6.896885	2.273887	0.479538
H	7.264523	1.384827	-1.463670

H	5.030741	-5.024238	-1.882111
H	7.889678	2.707232	0.554863
C	-3.588076	-0.391976	2.862684
H	-3.613484	0.701112	2.936189
H	-4.494329	-0.712515	2.338049
H	-3.586915	-0.817959	3.872074
C	2.135236	-2.540777	2.502056
O	0.562183	-1.199034	3.327175
C	1.837006	-1.616980	3.458983
H	2.399813	-1.175787	4.267965
H	3.085388	-3.030034	2.343827
C	-6.632144	-1.808854	-2.080980
H	-7.618482	-1.439572	-1.800094
H	-6.226393	-1.206016	-2.900910
H	-6.701162	-2.841256	-2.438062
O	0.570757	-5.036343	1.027426
C	1.529444	-5.709358	1.828485
H	1.651284	-5.236672	2.812512
H	1.158690	-6.727373	1.967240
H	2.509801	-5.747293	1.328187
H	1.997274	-3.781208	0.225956

24a

B3LYP-D3 SCF energy: -3112.99580530 a.u.
B3LYP-D3 enthalpy: -3112.142503 a.u.
B3LYP-D3 Gibbs free energy: -3112.288358 a.u.
wB97X-D SCF energy in solution: -3114.20764034 a.u.
wB97X-D enthalpy: -3113.354338 a.u.
wB97X-D Gibbs free energy: -3113.500193 a.u.

Cartesian coordinates

ATOM	X	Y	Z
C	-1.696134	2.374341	1.157887
C	-1.881959	1.140922	1.668698
C	-1.038707	3.548832	1.679441
C	-0.222987	4.446429	1.029766
C	0.186654	2.914544	-0.955743
H	0.833636	2.847691	-1.832911
C	-1.210991	2.497877	-1.318819
C	-2.139019	2.361987	-0.291104
C	-3.524287	1.940025	-0.382773
H	-4.153666	2.085727	0.489050
O	-4.206965	2.049661	-1.580904
C	-5.455041	1.441611	-1.627371
O	-5.879200	0.790816	-0.707431
H	0.609538	2.271449	-0.185048
C	0.228867	4.388790	-0.406869
Rh	-2.356370	0.110499	-0.069739
C	-3.737393	-0.833133	0.863374
O	-4.558043	-1.322955	1.490335
C	-1.517664	2.499902	-2.787142
H	-2.544988	2.242290	-3.029385
H	-1.302423	3.500399	-3.189911

H	-0.833406	1.810679	-3.297123
C	-2.698760	-0.596352	-1.948065
O	-2.924758	-0.915001	-3.019637
P	-0.245970	-1.100547	0.084065
O	0.897940	-0.760255	1.209159
O	0.494844	-0.663047	-1.339936
N	-0.244420	-2.772248	0.258598
C	1.761264	0.333072	1.096185
C	1.770477	-1.174967	-1.616635
C	0.709632	-3.480426	1.082250
C	-1.455327	-3.465686	-0.085972
C	1.642222	1.327717	2.089191
C	2.703326	0.389203	0.081229
C	2.858063	-0.735690	-0.883852
C	1.864564	-2.141028	-2.639188
C	1.957407	-3.823851	0.567043
C	0.375533	-3.745423	2.426356
C	-2.393850	-3.763994	0.925471
C	-1.717054	-3.761792	-1.425410
H	0.910891	1.195006	2.875453
C	2.449489	2.436846	2.034681
C	3.494764	1.587143	-0.029149
C	4.111831	-1.412059	-1.080264
H	0.972437	-2.394942	-3.201834
C	3.079282	-2.731717	-2.893832
H	2.176032	-3.618729	-0.472648
C	2.918263	-4.401841	1.395662
C	1.366568	-4.317443	3.246052
C	-0.949071	-3.476764	2.982720
C	-2.142031	-3.492962	2.340163
C	-3.621574	-4.328633	0.528062
C	-2.935878	-4.331598	-1.792577
H	-0.957681	-3.532965	-2.166788
H	2.355773	3.215666	2.785134
C	3.373989	2.607369	0.972036
C	4.372635	1.825738	-1.123979
C	4.217064	-2.414142	-2.103714
C	5.248380	-1.164035	-0.262637
H	3.173286	-3.474857	-3.680823
H	3.896730	-4.645482	0.992746
C	2.623099	-4.639947	2.741006
H	1.133727	-4.519385	4.288304
H	-0.978444	-3.359063	4.064569
H	-3.035150	-3.386210	2.952762
H	-4.367260	-4.549066	1.286995
C	-3.891813	-4.607588	-0.808976
H	-3.140800	-4.552299	-2.835730
C	4.161034	3.784897	0.869607
C	5.114548	2.984000	-1.198456
H	4.455750	1.084268	-1.909753
C	5.452645	-3.090879	-2.291304
C	6.426016	-1.850494	-0.460535
H	5.177249	-0.433693	0.534760
H	3.370525	-5.079778	3.394573
H	-4.845890	-5.046471	-1.085165

H	4.054093	4.546287	1.636996
C	5.022079	3.969896	-0.186395
H	5.778063	3.142407	-2.043919
H	5.521307	-3.840538	-3.075397
C	6.536639	-2.816276	-1.489571
H	7.278239	-1.651594	0.182762
H	5.622319	4.872656	-0.253739
H	7.474224	-3.343851	-1.637264
C	-1.720197	0.611821	3.050567
H	-1.072828	-0.272199	3.064575
H	-1.311037	1.380072	3.716355
H	-2.691554	0.300791	3.456652
C	0.249985	5.351288	2.035088
O	-1.082430	3.834676	3.016759
C	-0.295658	4.936800	3.211978
H	-0.244683	5.299699	4.227197
H	0.929673	6.175915	1.882075
O	1.550153	4.889472	-0.430834
C	2.011353	5.331741	-1.696597
H	1.359840	6.116948	-2.109389
H	3.011760	5.735113	-1.532135
H	2.087759	4.511062	-2.423967
H	-0.411305	5.022764	-1.045419
C	-6.109663	1.696334	-2.954155
H	-7.112917	1.270200	-2.949444
H	-6.154359	2.771094	-3.155594
H	-5.515330	1.235070	-3.751129

24b

B3LYP-D3 SCF energy:	-3112.99351312 a.u.
B3LYP-D3 enthalpy:	-3112.139929 a.u.
B3LYP-D3 Gibbs free energy:	-3112.287118 a.u.
wB97X-D SCF energy in solution:	-3114.20500089 a.u.
wB97X-D enthalpy:	-3113.351417 a.u.
wB97X-D Gibbs free energy:	-3113.498606 a.u.

Cartesian coordinates

ATOM	X	Y	Z
C	2.454270	1.314539	1.574125
C	2.138252	0.035158	1.837328
C	2.310386	2.519142	2.363237
C	1.799697	3.738739	1.983763
C	0.806910	2.864283	-0.200436
H	0.171142	3.213038	-1.015148
C	1.979532	2.099407	-0.775570
C	2.819528	1.428226	0.111710
C	3.932670	0.560707	-0.198763
H	4.586442	0.264089	0.614637
O	4.569165	0.640594	-1.421822
C	5.481389	-0.367821	-1.702909
O	5.637334	-1.307802	-0.966674
H	0.205189	2.222477	0.448830
C	1.277463	4.100568	0.614082

Rh	2.131557	-0.720280	-0.107300
C	3.086680	-2.268386	0.524553
O	3.698162	-3.120185	0.976427
C	2.277729	2.362492	-2.218288
H	3.081864	1.756525	-2.626839
H	2.556768	3.423370	-2.282089
H	1.369527	2.239458	-2.814818
C	2.104990	-1.118596	-2.095889
O	2.105117	-1.305708	-3.221756
P	-0.253768	-1.109592	0.042176
O	-1.174457	-0.772856	-1.281283
O	-0.820126	-0.014613	1.150359
N	-0.878026	-2.643870	0.338282
C	-1.539132	0.524273	-1.623723
C	-2.197544	0.091531	1.372480
C	-2.052173	-3.138037	-0.352069
C	-0.010512	-3.644620	0.900794
C	-1.039384	1.007202	-2.851928
C	-2.404384	1.252392	-0.822687
C	-3.008221	0.643394	0.396727
C	-2.683281	-0.382041	2.608157
C	-3.322148	-2.886020	0.160726
C	-1.880388	-3.803592	-1.583778
C	0.705248	-4.503275	0.037408
C	0.132242	-3.732910	2.286713
H	-0.418044	0.349663	-3.451071
C	-1.369991	2.274950	-3.265537
C	-2.669669	2.616896	-1.196520
C	-4.430920	0.580287	0.598876
H	-1.973958	-0.753142	3.340419
C	-4.036532	-0.363322	2.846854
H	-3.418224	-2.378534	1.111913
C	-4.453373	-3.256816	-0.565530
C	-3.036534	-4.155439	-2.304453
C	-0.565440	-4.154499	-2.117103
C	0.552686	-4.463283	-1.417194
C	1.589454	-5.428989	0.622616
C	1.010897	-4.661707	2.842878
H	-0.454666	-3.069600	2.914187
H	-1.009524	2.652363	-4.218746
C	-2.157899	3.121544	-2.440232
C	-3.391045	3.511362	-0.358039
C	-4.941457	0.077127	1.843482
C	-5.363157	0.950653	-0.408735
H	-4.431071	-0.718479	3.794847
H	-5.440383	-3.035124	-0.170901
C	-4.307894	-3.885099	-1.805437
H	-2.925962	-4.657951	-3.261825
H	-0.523734	-4.287132	-3.196709
H	1.407830	-4.829685	-1.982883
H	2.158356	-6.092547	-0.023122
C	1.741827	-5.509346	2.003806
H	1.125352	-4.723929	3.920789
C	-2.422974	4.467755	-2.810010
C	-3.615221	4.816332	-0.738960

H	-3.760930	3.159402	0.597688
C	-6.346835	0.004015	2.041304
C	-6.719507	0.850790	-0.190146
H	-4.996937	1.301016	-1.366531
H	-5.184563	-4.166109	-2.381429
H	2.430212	-6.233625	2.428513
H	-2.038563	4.831316	-3.759567
C	-3.138415	5.300348	-1.980466
H	-4.163297	5.481566	-0.078016
H	-6.719389	-0.369884	2.991520
C	-7.220539	0.383348	1.048281
H	-7.412395	1.129487	-0.978765
H	-3.331291	6.328848	-2.270531
H	-8.292641	0.317086	1.207568
C	1.748399	-0.615177	3.118072
H	0.699749	-0.927580	3.080251
H	2.345675	-1.513949	3.309891
H	1.880285	0.079389	3.954614
C	1.758248	4.531873	3.180510
O	2.570048	2.509016	3.704169
C	2.234465	3.744611	4.183915
H	2.402519	3.898211	5.238815
H	1.405983	5.549988	3.273440
C	6.160254	-0.103633	-3.015587
H	6.903891	-0.878872	-3.200284
H	5.417389	-0.101359	-3.821178
H	6.635213	0.882405	-3.003345
O	2.233865	4.761004	-0.215499
C	2.641480	6.037593	0.252170
H	3.195125	5.968442	1.198036
H	3.294710	6.458262	-0.515587
H	1.777441	6.705692	0.393613
H	0.403946	4.759863	0.748825

TS1a

B3LYP-D3 SCF energy: -3112.92707509 a.u.
B3LYP-D3 enthalpy: -3112.077105 a.u.
B3LYP-D3 Gibbs free energy: -3112.224771 a.u.
wB97X-D SCF energy in solution: -3114.13456230 a.u.
wB97X-D enthalpy: -3113.284592 a.u.
wB97X-D Gibbs free energy: -3113.432258 a.u.
Imaginary frequency: -213.0080 cm-1

Cartesian coordinates

ATOM	X	Y	Z
C	-1.183610	1.947306	1.386198
C	-1.799979	1.036444	2.024624
C	-0.310091	3.073067	1.511607
C	0.514779	3.837045	0.718298
C	-0.088175	2.857250	-1.575829
H	0.157587	3.011177	-2.632893
C	-1.529249	3.208083	-1.325906
C	-2.285344	2.468136	-0.515699

C	-3.593039	2.136422	-0.148377
H	-3.996560	2.327099	0.841862
O	-4.536399	2.103230	-1.172466
C	-5.743666	1.502665	-0.877814
O	-5.956227	0.974165	0.185412
H	0.092117	1.804857	-1.351168
C	0.889384	3.714450	-0.732955
H	1.872187	3.219942	-0.786760
Rh	-2.283503	0.352104	0.057770
C	-3.705941	-0.825391	0.861365
O	-4.512106	-1.385128	1.441561
C	-2.055591	4.498907	-1.899002
H	-3.097557	4.680599	-1.625873
H	-1.428249	5.327376	-1.549565
H	-1.974737	4.477535	-2.993337
C	-2.476292	-0.059879	-1.865189
O	-2.601205	-0.269401	-2.980750
P	-0.295467	-1.070063	0.074903
O	0.838872	-0.943733	1.250659
O	0.492332	-0.519269	-1.279771
N	-0.393698	-2.747808	0.044485
C	1.784859	0.078839	1.305412
C	1.749828	-1.032332	-1.613604
C	0.529680	-3.607610	0.752676
C	-1.622721	-3.336682	-0.410580
C	1.772276	0.863736	2.478130
C	2.718373	0.249074	0.295877
C	2.842112	-0.725933	-0.824309
C	1.822722	-1.849545	-2.759978
C	1.766139	-3.919789	0.191275
C	0.179613	-4.059685	2.041430
C	-2.569940	-3.781940	0.536542
C	-1.888116	-3.395021	-1.780702
H	1.047419	0.628079	3.248868
C	2.660859	1.899491	2.608841
C	3.568264	1.410233	0.370178
C	4.080242	-1.402076	-1.109693
H	0.927498	-1.996714	-3.354646
C	3.022497	-2.432705	-3.089837
H	1.997461	-3.575984	-0.808090
C	2.701435	-4.648769	0.924570
C	1.145348	-4.781731	2.767425
C	-1.142274	-3.843731	2.625970
C	-2.327781	-3.740199	1.978343
C	-3.804677	-4.252836	0.049815
C	-3.116363	-3.871266	-2.237907
H	-1.123922	-3.059190	-2.474692
H	2.659963	2.511163	3.505376
C	3.539410	2.233974	1.543797
C	4.405212	1.809083	-0.709304
C	4.163925	-2.256122	-2.261694
C	5.220511	-1.299809	-0.266371
H	3.102042	-3.062076	-3.971838
H	3.671151	-4.868149	0.487990
C	2.392501	-5.070602	2.220683

H	0.898820	-5.128932	3.767405
H	-1.180326	-3.893270	3.712854
H	-3.225955	-3.710662	2.592019
H	-4.556172	-4.583883	0.761332
C	-4.077064	-4.297489	-1.314738
H	-3.323091	-3.906185	-3.303204
C	4.358290	3.392478	1.602908
C	5.171894	2.952019	-0.628197
H	4.425783	1.210807	-1.612825
C	5.382493	-2.931516	-2.541865
C	6.381241	-1.981842	-0.558344
H	5.167131	-0.687793	0.626117
H	3.120420	-5.628889	2.801778
H	-5.037294	-4.668603	-1.660282
H	4.325600	4.004442	2.500299
C	5.157331	3.750213	0.540679
H	5.795311	3.241400	-1.469398
H	5.434111	-3.567001	-3.422179
C	6.471158	-2.797878	-1.711169
H	7.235878	-1.895860	0.106396
H	5.772504	4.643511	0.593648
H	7.395628	-3.323317	-1.931191
C	-2.040345	0.382480	3.330595
H	-1.650154	-0.642416	3.318184
H	-1.544662	0.949158	4.126315
H	-3.111418	0.324566	3.554419
C	1.088867	4.823318	1.585575
O	-0.262206	3.531638	2.811487
C	0.591505	4.593075	2.829055
H	0.718256	5.074386	3.787084
H	1.777677	5.596687	1.282415
C	-6.679658	1.604739	-2.048958
H	-7.628772	1.132439	-1.795289
H	-6.840130	2.655691	-2.310343
H	-6.236869	1.114431	-2.922676
O	1.013538	5.041634	-1.235191
C	1.951017	5.173291	-2.293940
H	1.654821	4.603874	-3.187703
H	1.984317	6.234273	-2.550945
H	2.951847	4.840633	-1.981012

TS1a-n-rot

B3LYP-D3 SCF energy: -3112.92611114 a.u.
B3LYP-D3 enthalpy: -3112.076325 a.u.
B3LYP-D3 Gibbs free energy: -3112.223814 a.u.
wB97X-D SCF energy in solution: -3114.13425891 a.u.
wB97X-D enthalpy: -3113.284473 a.u.
wB97X-D Gibbs free energy: -3113.431962 a.u.
Imaginary frequency: -209.1058 cm-1

Cartesian coordinates

ATOM	X	Y	Z
C	0.993053	-2.363316	1.089241

C	1.797337	-1.718513	1.834407
C	-0.023592	-3.369742	1.079176
C	-0.995033	-3.857536	0.235549
C	-0.460186	-2.476367	-1.860632
H	-0.812828	-2.356596	-2.891474
C	0.941783	-3.019898	-1.854657
C	1.849786	-2.568622	-0.991106
C	3.216093	-2.476048	-0.706953
H	3.671419	-2.934105	0.166319
O	4.067205	-2.314834	-1.797134
C	5.374651	-1.973749	-1.511690
O	5.751239	-1.756525	-0.386885
H	-0.489316	-1.490937	-1.391544
C	-1.456724	-3.403257	-1.121575
H	-2.384501	-2.826948	-0.984651
Rh	2.175348	-0.659442	0.024511
C	3.814068	0.153920	0.873451
O	4.763611	0.469669	1.417701
C	1.256465	-4.189800	-2.752020
H	2.290989	-4.525173	-2.649027
H	0.571823	-5.013661	-2.519743
H	1.077305	-3.910951	-3.798286
C	2.278577	0.172822	-1.772894
O	2.361725	0.641770	-2.809884
P	0.428763	1.008035	0.456578
O	-0.696992	0.925257	1.673352
O	-0.509307	0.872641	-0.888134
N	0.921586	2.600599	0.586581
C	-1.688186	-0.060296	1.671136
C	-1.767719	1.474221	-1.042435
C	2.190825	2.914471	1.183209
C	0.115789	3.726957	0.185339
C	-1.628399	-1.019703	2.703685
C	-2.702735	-0.026938	0.727379
C	-2.827414	1.100951	-0.236906
C	-1.872991	2.420259	-2.080782
C	2.400206	2.677430	2.544873
C	3.206573	3.458842	0.369737
C	0.550688	4.488145	-0.919927
C	-1.078249	4.020922	0.846275
H	-0.825376	-0.950293	3.429100
C	-2.577615	-2.007827	2.764012
C	-3.632027	-1.128818	0.715393
C	-4.046080	1.862809	-0.357619
H	-0.999635	2.621871	-2.689572
C	-3.060371	3.080371	-2.264239
H	1.578669	2.290684	3.141529
C	3.644623	2.942734	3.114915
C	4.458052	3.702046	0.965748
C	2.982808	3.806857	-1.034045
C	1.831533	4.254671	-1.588482
C	-0.288478	5.525872	-1.367492
C	-1.887661	5.057162	0.384541
H	-1.377552	3.412064	1.691532
H	-2.545102	-2.750984	3.554594

C	-3.573645	-2.113257	1.756719
C	-4.584913	-1.311406	-0.326495
C	-4.158894	2.849636	-1.393287
C	-5.136888	1.710655	0.541738
H	-3.158260	3.819146	-3.054404
H	3.810996	2.750586	4.170451
C	4.675887	3.450192	2.317959
H	5.259941	4.109486	0.355864
H	3.872880	3.813975	-1.660637
H	1.879180	4.585354	-2.624540
H	0.022717	6.120483	-2.222478
C	-1.491490	5.807234	-0.727027
H	-2.830271	5.266627	0.880273
C	-4.486322	-3.201427	1.739670
C	-5.440643	-2.392067	-0.325440
H	-4.627422	-0.593848	-1.137200
C	-5.357722	3.601629	-1.513753
C	-6.280046	2.468557	0.407561
H	-5.060547	0.997531	1.354036
H	5.649737	3.652298	2.753175
H	-2.121580	6.612871	-1.091945
H	-4.431269	-3.935288	2.539016
C	-5.401991	-3.343121	0.722327
H	-6.154508	-2.512440	-1.135380
H	-5.430242	4.338621	-2.309586
C	-6.401021	3.416181	-0.635595
H	-7.094748	2.340228	1.114364
H	-6.089501	-4.183680	0.716155
H	-7.310721	4.001127	-0.733208
C	2.287345	-1.461393	3.206455
H	2.092175	-0.421870	3.494886
H	1.784858	-2.132713	3.911212
H	3.369256	-1.621644	3.277080
C	-1.627925	-4.930303	0.945552
O	-0.038968	-4.075594	2.264518
C	-1.018378	-5.016500	2.156406
H	-1.138218	-5.665943	3.010453
H	-2.432021	-5.541457	0.565860
C	6.187639	-1.914053	-2.774072
H	7.212847	-1.634730	-2.530835
H	6.172522	-2.887705	-3.274696
H	5.751468	-1.184773	-3.465090
O	-1.753232	-4.581129	-1.866733
C	-2.765562	-4.405695	-2.847381
H	-2.468668	-3.694149	-3.632415
H	-2.928362	-5.383544	-3.305807
H	-3.702873	-4.054729	-2.391240

TS1a-p-rot

B3LYP-D3 SCF energy:	-3112.92129384 a.u.
B3LYP-D3 enthalpy:	-3112.070909 a.u.
B3LYP-D3 Gibbs free energy:	-3112.219156 a.u.
wB97X-D SCF energy in solution:	-3114.12831665 a.u.

wB97X-D enthalpy: -3113.277932 a.u.
wB97X-D Gibbs free energy: -3113.426179 a.u.
Imaginary frequency: -190.3194 cm-1

Cartesian coordinates

ATOM	X	Y	Z
C	1.620811	0.974771	1.748296
C	0.977704	1.952911	1.233042
C	1.503851	-0.045013	2.737728
C	2.155476	-1.204507	3.118317
C	3.920569	-1.487096	1.262356
H	4.745733	-2.162076	1.012058
C	4.386060	-0.064665	1.104638
C	3.566487	0.907070	0.704748
C	3.586412	2.164087	0.097292
H	3.096505	-1.700183	0.579346
C	3.467838	-1.827113	2.690979
Rh	1.761207	1.065112	-0.542155
C	1.144386	2.540282	-1.770105
O	0.865437	3.387951	-2.479648
C	5.816915	0.272325	1.449883
H	6.052783	1.319752	1.248798
H	6.001686	0.045450	2.503591
H	6.491726	-0.357938	0.856247
C	2.797109	0.023111	-1.876195
O	3.527561	-0.462344	-2.607949
P	-0.291023	-0.296616	-0.689980
O	-1.537626	0.321168	-1.566721
O	-0.875655	-0.270193	0.853756
N	-0.369027	-1.862319	-1.301727
C	-2.261504	1.416785	-1.090301
C	-2.158885	-0.735599	1.139133
C	-1.412652	-2.324008	-2.190524
C	0.733853	-2.758787	-1.095290
C	-2.157999	2.611226	-1.833240
C	-3.072344	1.293904	0.027011
C	-3.258481	-0.026487	0.690677
C	-2.261991	-1.921531	1.895870
C	-2.683029	-2.615415	-1.695313
C	-1.128700	-2.420145	-3.567883
C	1.610704	-3.034907	-2.167243
C	0.923545	-3.346142	0.159055
H	-1.571449	2.607312	-2.743631
C	-2.814463	3.736996	-1.399630
C	-3.700718	2.486512	0.532299
C	-4.556378	-0.619274	0.868876
H	-1.355757	-2.375550	2.280287
C	-3.504720	-2.455110	2.139980
H	-2.865935	-2.554301	-0.630644
C	-3.710955	-2.958393	-2.571610
C	-2.187271	-2.753065	-4.434465
C	0.213770	-2.239677	-4.116641
C	1.396660	-2.514467	-3.517588
C	2.718220	-3.865917	-1.907503
C	2.003099	-4.200635	0.377975

H	0.215413	-3.128137	0.949855
H	-2.747166	4.660776	-1.967448
C	-3.570182	3.716415	-0.197630
C	-4.422080	2.508963	1.758489
C	-4.672493	-1.846113	1.606515
C	-5.735177	-0.063119	0.300229
H	-3.604647	-3.366509	2.723151
H	-4.702755	-3.163268	-2.179846
C	-3.463722	-3.015577	-3.946432
H	-1.990630	-2.820051	-5.501269
H	0.249579	-1.965089	-5.169362
H	2.292642	-2.440198	-4.130092
H	3.416983	-4.073379	-2.713476
C	2.910510	-4.447747	-0.657571
H	2.139190	-4.664030	1.350639
C	-4.191637	4.893375	0.300355
C	-5.001135	3.670684	2.219246
H	-4.507686	1.598731	2.340059
C	-5.952995	-2.438414	1.776535
C	-6.958949	-0.671786	0.471445
H	-5.662693	0.845536	-0.285785
H	-4.263420	-3.270737	-4.635317
H	3.760772	-5.102994	-0.492869
H	-4.092665	5.813794	-0.269315
C	-4.896251	4.874194	1.481706
H	-5.541982	3.663853	3.161168
H	-6.027519	-3.362160	2.344645
C	-7.075058	-1.864998	1.224044
H	-7.844407	-0.233359	0.020432
H	-5.364849	5.779893	1.854915
H	-8.048105	-2.328834	1.355286
C	0.082351	3.110306	1.427888
H	-0.871700	2.934060	0.918829
H	-0.110563	3.261051	2.495357
H	0.515567	4.026616	1.010685
C	1.318428	-1.807037	4.115941
O	0.332460	0.079027	3.460171
C	0.247006	-0.984310	4.290911
H	-0.615502	-1.008659	4.939976
H	1.512347	-2.737158	4.631851
O	4.571572	2.375407	-0.859960
C	4.475824	3.547232	-1.586775
O	3.555173	4.314107	-1.448985
H	3.217429	3.064918	0.577590
O	4.542959	-1.477090	3.555827
C	4.481298	-2.011300	4.868672
H	5.441714	-1.788421	5.338642
H	3.678352	-1.555763	5.463041
H	4.336364	-3.103139	4.850634
H	3.320006	-2.919517	2.730860
C	5.641919	3.678471	-2.524430
H	6.578998	3.675503	-1.958138
H	5.669818	2.822071	-3.206647
H	5.548775	4.605720	-3.089849

TS1a-i2

B3LYP-D3 SCF energy:	-3112.91826245 a.u.
B3LYP-D3 enthalpy:	-3112.067732 a.u.
B3LYP-D3 Gibbs free energy:	-3112.214878 a.u.
wB97X-D SCF energy in solution:	-3114.12671834 a.u.
wB97X-D enthalpy:	-3113.276188 a.u.
wB97X-D Gibbs free energy:	-3113.423334 a.u.
Imaginary frequency:	-191.4443 cm-1

Cartesian coordinates

ATOM	X	Y	Z
C	3.551524	1.186882	-0.329914
C	4.159935	0.076428	-0.517690
C	3.464436	2.516739	-0.824366
C	2.795909	3.695828	-0.542608
C	0.852282	2.871188	0.895395
H	-0.013762	3.293630	1.413283
C	1.484575	1.837497	1.773212
C	2.127187	0.763981	1.315133
C	2.588451	-0.470911	1.788385
H	0.498967	2.422724	-0.032139
C	1.784854	4.043955	0.521893
Rh	2.157679	-0.703223	-0.327513
C	2.864857	-2.591421	-0.338184
O	3.483992	-3.546482	-0.403617
C	1.368198	2.055245	3.263570
H	2.000920	1.379414	3.842719
H	1.603660	3.094908	3.510516
H	0.323359	1.878434	3.553954
C	5.448907	-0.612311	-0.735512
H	5.415314	-1.210439	-1.653708
H	6.260993	0.118050	-0.818624
H	5.674810	-1.298892	0.089277
C	3.234475	4.639847	-1.521463
O	4.260938	2.704665	-1.943803
C	4.117986	3.991840	-2.332684
H	4.702511	4.293683	-3.189266
H	2.917062	5.670779	-1.594331
C	1.881027	-0.484627	-2.310975
O	1.822399	-0.366401	-3.445037
P	-0.230886	-0.970412	-0.167481
O	-1.070354	-0.209684	-1.363041
O	-0.762918	-0.209757	1.194409
N	-0.975355	-2.468426	-0.346374
C	-1.311506	1.160187	-1.329753
C	-2.137935	-0.052994	1.398685
C	-2.133715	-2.654993	-1.197841
C	-0.273273	-3.664022	0.026542
C	-0.679587	1.934235	-2.327258
C	-2.157971	1.700271	-0.375067
C	-2.859967	0.820287	0.602937
C	-2.716790	-0.815165	2.433928
C	-3.411746	-2.392957	-0.709907

C	-1.929302	-3.035934	-2.539742
C	0.393326	-4.413646	-0.966718
C	-0.249257	-4.058834	1.366057
H	-0.081352	1.431021	-3.077467
C	-0.843223	3.297843	-2.326683
C	-2.274676	3.133933	-0.322990
C	-4.290397	0.825332	0.748365
H	-2.070261	-1.436308	3.044872
C	-4.074601	-0.751384	2.636269
H	-3.537219	-2.118443	0.329195
C	-4.510878	-2.455800	-1.565480
C	-3.051174	-3.076253	-3.388141
C	-0.619873	-3.425631	-3.059166
C	0.380327	-4.037011	-2.380572
C	1.106816	-5.554932	-0.553570
C	0.459096	-5.196501	1.747891
H	-0.801265	-3.467084	2.089526
H	-0.361750	3.906569	-3.087016
C	-1.609180	3.933215	-1.313712
C	-2.969441	3.806288	0.720778
C	-4.895854	0.033625	1.782544
C	-5.142786	1.550223	-0.129066
H	-4.538620	-1.327937	3.431716
H	-5.501095	-2.226428	-1.183431
C	-4.326525	-2.786662	-2.910890
H	-2.912266	-3.354464	-4.429566
H	-0.494061	-3.317609	-4.134946
H	1.234952	-4.383901	-2.959187
H	1.640164	-6.138198	-1.299227
C	1.141701	-5.942757	0.781852
H	0.478787	-5.502141	2.789100
C	-1.696041	5.350176	-1.242469
C	-3.017153	5.182391	0.771385
H	-3.455768	3.224604	1.494695
C	-6.310389	0.030234	1.919017
C	-6.511451	1.511747	0.021204
H	-4.705082	2.127905	-0.934818
H	-5.176644	-2.824477	-3.585525
H	1.699801	-6.827388	1.072450
H	-1.198461	5.939947	-2.008375
C	-2.383634	5.966260	-0.222230
H	-3.544915	5.671366	1.584965
H	-6.755035	-0.566137	2.711553
C	-7.104459	0.753874	1.059292
H	-7.143145	2.065518	-0.667238
H	-2.437017	7.049613	-0.172406
H	-8.184724	0.739153	1.168588
O	1.783782	-1.120048	2.725080
C	2.185216	-2.377912	3.103839
O	3.094937	-2.961716	2.566349
C	1.368600	-2.857385	4.272249
H	1.683323	-2.319515	5.173884
H	0.307701	-2.645496	4.116539
H	1.533174	-3.924934	4.416357
H	3.647539	-0.691538	1.897269

O	2.350120	4.677655	1.672415
H	1.132840	4.809980	0.085619
C	3.667476	4.299137	2.054293
H	3.856256	4.804872	3.004094
H	3.770862	3.215403	2.199285
H	4.415222	4.626211	1.320300

TS1a-i2-p-rot

B3LYP-D3 SCF energy: -3112.91756037 a.u.
B3LYP-D3 enthalpy: -3112.067165 a.u.
B3LYP-D3 Gibbs free energy: -3112.213730 a.u.
wB97X-D SCF energy in solution: -3114.12207989 a.u.
wB97X-D enthalpy: -3113.271685 a.u.
wB97X-D Gibbs free energy: -3113.418250 a.u.
Imaginary frequency: -229.9291 cm-1

Cartesian coordinates

ATOM	X	Y	Z
C	-4.004542	0.649300	-0.887675
C	-3.785543	0.022433	-1.986587
C	-4.991537	0.757218	0.126634
C	-5.150510	1.340908	1.372856
C	-2.736020	1.915378	1.985710
H	-2.194967	2.430476	2.787066
C	-2.200389	2.377325	0.656047
C	-2.135410	1.568916	-0.411100
C	-1.531200	1.578152	-1.691727
H	-2.582197	0.838692	2.111580
C	-4.231813	2.227234	2.182273
Rh	-1.893474	-0.467865	-1.096921
C	-1.245413	-1.390883	-2.748371
O	-0.901111	-1.880002	-3.719582
C	-1.830525	3.834528	0.493161
H	-1.148662	3.985023	-0.346236
H	-2.741773	4.420887	0.337111
H	-1.356776	4.215680	1.402250
C	-4.313407	-0.319646	-3.323990
H	-4.333327	-1.407926	-3.456397
H	-5.328462	0.072040	-3.448805
H	-3.673462	0.091383	-4.114466
C	-6.423703	0.902178	1.850745
O	-6.102561	-0.024421	-0.151188
C	-6.955969	0.096182	0.887822
H	-7.890544	-0.437494	0.797224
H	-6.870045	1.158631	2.801496
C	-2.892007	-1.931932	-0.066323
O	-3.738529	-2.524934	0.419477
P	0.285450	-0.772932	-0.083202
O	0.878311	0.119517	1.150944
O	1.295187	-0.386732	-1.341673
N	0.686512	-2.287209	0.538878
C	1.524424	1.347927	1.018613
C	2.678779	-0.521846	-1.193321

C	1.555585	-2.471437	1.684419
C	-0.026350	-3.451985	0.099551
C	0.949035	2.406165	1.750092
C	2.695720	1.486508	0.291768
C	3.371077	0.318160	-0.338719
C	3.297408	-1.539360	-1.949242
C	2.939554	-2.440836	1.530440
C	0.972709	-2.644930	2.955707
C	-0.995233	-4.030902	0.949062
C	0.215503	-3.968632	-1.176060
H	0.079356	2.191376	2.358504
C	1.507105	3.657075	1.690942
C	3.232952	2.817611	0.146685
C	4.752868	0.005771	-0.079228
H	2.694074	-2.105397	-2.650705
C	4.641105	-1.775165	-1.785565
H	3.360380	-2.367917	0.538113
C	3.770212	-2.489127	2.649157
C	1.828109	-2.671276	4.073028
C	-0.456003	-2.883297	3.141330
C	-1.294211	-3.513206	2.284719
C	-1.727850	-5.125500	0.448198
C	-0.522912	-5.052407	-1.647294
H	0.984905	-3.508922	-1.787583
H	1.078473	4.476976	2.260626
C	2.631640	3.906559	0.861451
C	4.323189	3.113640	-0.718747
C	5.389262	-1.045351	-0.822355
C	5.504655	0.659077	0.935579
H	5.136533	-2.549125	-2.364982
H	4.846726	-2.433954	2.518443
C	3.209655	-2.583269	3.925838
H	1.394497	-2.786992	5.062951
H	-0.844756	-2.650160	4.130971
H	-2.295862	-3.737730	2.646367
H	-2.489468	-5.576632	1.078519
C	-1.499541	-5.630061	-0.828262
H	-0.338868	-5.442784	-2.643571
C	3.159753	5.217664	0.717522
C	4.800660	4.399386	-0.850459
H	4.777263	2.316233	-1.294502
C	6.749368	-1.361762	-0.558595
C	6.815291	0.312811	1.180842
H	5.030465	1.427978	1.533606
H	3.848846	-2.605383	4.803488
H	-2.078850	-6.476197	-1.185474
H	2.697012	6.027602	1.275678
C	4.223958	5.464078	-0.118883
H	5.627832	4.597149	-1.526030
H	7.220903	-2.151914	-1.137091
C	7.451563	-0.697463	0.420692
H	7.365323	0.818243	1.969301
H	4.616100	6.470891	-0.226575
H	8.488073	-0.952609	0.619905
O	-0.236444	2.066441	-1.784567

C	0.380435	1.860690	-3.004370
C	1.745364	2.482537	-3.033366
H	1.998202	2.740463	-4.063171
H	1.804722	3.357227	-2.384024
H	2.471161	1.748397	-2.669753
O	-0.152593	1.243697	-3.894498
H	-2.114102	1.726797	-2.596420
O	-4.411642	3.609072	1.908029
C	-5.658958	4.147509	2.321870
H	-5.836088	3.968051	3.393778
H	-5.605290	5.223732	2.144771
H	-6.497981	3.730824	1.748446
H	-4.472685	2.037791	3.242316

TS1a-i3

B3LYP-D3 SCF energy: -3112.91825991 a.u.
B3LYP-D3 enthalpy: -3112.067733 a.u.
B3LYP-D3 Gibbs free energy: -3112.214489 a.u.
wB97X-D SCF energy in solution: -3114.12520619 a.u.
wB97X-D enthalpy: -3113.274679 a.u.
wB97X-D Gibbs free energy: -3113.421435 a.u.
Imaginary frequency: -233.9711 cm-1

Cartesian coordinates

ATOM	X	Y	Z
C	-2.770396	1.720990	-0.716333
C	-1.499626	1.868522	-0.842254
C	-4.005715	2.341295	-1.083251
C	-5.364008	2.121996	-0.969215
C	-5.339969	-0.238137	0.045777
H	-6.024517	-1.029820	0.370720
C	-4.395626	0.096439	1.165888
C	-3.108723	0.361549	0.953637
C	-1.839006	0.392919	1.534688
H	-4.793022	-0.622168	-0.819716
C	-6.172560	0.972369	-0.426859
Rh	-1.637686	-0.246041	-0.578909
C	-4.950676	0.197529	2.564505
H	-4.184857	0.477304	3.292678
H	-5.756291	0.938568	2.584265
H	-5.374396	-0.771823	2.857902
C	-2.462447	-2.083092	-0.453478
O	-3.092186	-3.027742	-0.558305
C	-0.414216	2.844452	-1.080673
H	0.282136	2.480321	-1.840057
H	-0.852225	3.795424	-1.404039
H	0.167204	3.018312	-0.168599
C	-5.997737	3.254270	-1.578113
O	-3.790356	3.531611	-1.751089
C	-5.006993	4.069531	-2.024831
H	-4.995625	5.023691	-2.529937
H	-7.063222	3.417323	-1.651680
C	-1.529132	-0.418730	-2.564913

O	-1.412739	-0.422243	-3.699801
P	0.713946	-0.660737	-0.269260
O	1.508638	-0.168401	1.077840
O	1.397706	0.267292	-1.466969
N	1.326539	-2.226648	-0.307054
C	1.792152	1.181717	1.294798
C	2.786797	0.412692	-1.545482
C	2.419019	-2.688927	0.523461
C	0.620035	-3.212757	-1.075276
C	1.141078	1.791512	2.388658
C	2.707037	1.844241	0.493961
C	3.462630	1.116944	-0.564613
C	3.429848	-0.183740	-2.649821
C	3.729845	-2.313438	0.234076
C	2.125906	-3.475034	1.657086
C	-0.119940	-4.214242	-0.410371
C	0.622864	-3.127959	-2.471031
H	0.503714	1.178739	3.016143
C	1.349711	3.126412	2.636800
C	2.872802	3.259815	0.700497
C	4.900354	1.095368	-0.597609
H	2.825368	-0.668547	-3.408712
C	4.800603	-0.136189	-2.734627
H	3.933121	-1.738489	-0.658478
C	4.766268	-2.658013	1.100264
C	3.186784	-3.798982	2.524171
C	0.793041	-4.003099	1.936665
C	-0.170601	-4.332624	1.045820
C	-0.860964	-5.111941	-1.202816
C	-0.124855	-4.025010	-3.232666
H	1.216045	-2.353597	-2.946586
H	0.862958	3.608701	3.480095
C	2.188321	3.899081	1.789254
C	3.660956	4.070426	-0.162664
C	5.568657	0.465032	-1.701437
C	5.696393	1.633763	0.450608
H	5.312977	-0.587438	-3.579712
H	5.779356	-2.338217	0.875582
C	4.490475	-3.392037	2.256768
H	2.976437	-4.395306	3.408360
H	0.610801	-4.270872	2.975549
H	-1.062728	-4.817889	1.435274
H	-1.447249	-5.882166	-0.709407
C	-0.867353	-5.021979	-2.591424
H	-0.126285	-3.947736	-4.315681
C	2.352765	5.295389	1.994058
C	3.787737	5.424652	0.056145
H	4.160868	3.614372	-1.008846
C	6.989182	0.434484	-1.730825
C	7.071465	1.572882	0.398295
H	5.209653	2.085440	1.306980
H	5.290489	-3.654723	2.942481
H	-1.450627	-5.727649	-3.175220
H	1.837260	5.762381	2.829359
C	3.138366	6.045381	1.149538

H	4.390549	6.023253	-0.620578
H	7.481669	-0.037369	-2.577142
C	7.728297	0.977666	-0.705307
H	7.658502	1.982759	1.215045
H	3.254653	7.112588	1.312870
H	8.813265	0.943998	-0.734901
O	-7.012289	1.507635	0.590297
C	-8.186175	0.749334	0.848310
H	-8.770123	0.589870	-0.070461
H	-7.963165	-0.228948	1.298443
H	-8.781923	1.331613	1.554527
H	-6.809070	0.608317	-1.252917
O	-1.403974	-0.368992	2.620147
C	-1.818969	-1.644877	2.886141
O	-2.589623	-2.260443	2.189645
H	-1.291022	1.327516	1.562650
C	-1.166104	-2.129596	4.151691
H	-0.078312	-2.073778	4.047060
H	-1.453606	-1.487223	4.990645
H	-1.474856	-3.156541	4.347089

TS1a-i3-p-rot
B3LYP-D3 SCF energy: -3112.91381442 a.u.
B3LYP-D3 enthalpy: -3112.063697 a.u.
B3LYP-D3 Gibbs free energy: -3112.211968 a.u.
wB97X-D SCF energy in solution: -3114.12048888 a.u.
wB97X-D enthalpy: -3113.270371 a.u.
wB97X-D Gibbs free energy: -3113.418642 a.u.
Imaginary frequency: -248.1847 cm-1

Cartesian coordinates

ATOM	X	Y	Z
C	-2.757691	1.106823	1.116604
C	-1.784399	0.397664	1.567529
C	-3.286530	2.419443	1.208445
C	-4.274945	3.180375	0.606335
C	-4.820816	1.819205	-1.495011
H	-5.538665	1.841413	-2.322023
C	-4.712567	0.407558	-0.978922
C	-3.599839	-0.066770	-0.399155
C	-3.066039	-1.351389	-0.082778
H	-3.857262	2.173612	-1.876200
C	-5.319034	2.815718	-0.426239
Rh	-1.471966	0.012795	-0.537054
C	-5.943400	-0.461347	-1.077362
H	-5.811128	-1.426349	-0.584068
H	-6.796574	0.057614	-0.631909
H	-6.181178	-0.639600	-2.135333
C	-1.422293	-0.645856	-2.403120
O	-1.399465	-1.112703	-3.443721
C	-1.209668	-0.188941	2.797349
H	-0.120456	-0.111291	2.815951
H	-1.629734	0.307509	3.679295

H	-1.443527	-1.259450	2.847062
C	-4.139741	4.495859	1.147328
O	-2.554729	3.224896	2.069354
C	-3.099403	4.460589	2.028927
H	-2.652532	5.197020	2.680499
H	-4.748652	5.353529	0.896938
C	-0.712828	1.881394	-1.081787
O	-0.502466	2.922752	-1.493758
P	0.753092	-0.757852	-0.093484
O	1.524311	0.102686	1.078611
O	1.592424	-0.329181	-1.464970
N	1.183085	-2.297402	0.403967
C	1.777105	1.459281	0.835753
C	2.991286	-0.249978	-1.432109
C	2.094418	-2.584394	1.488336
C	0.511499	-3.407583	-0.219793
C	1.002593	2.406854	1.540161
C	2.774401	1.813299	-0.057737
C	3.602903	0.756845	-0.703578
C	3.714498	-1.214301	-2.163993
C	3.457353	-2.323891	1.347325
C	1.568450	-3.073421	2.702863
C	-0.402061	-4.178538	0.527858
C	0.751535	-3.686599	-1.568289
H	0.252709	2.069674	2.243413
C	1.205952	3.745027	1.306665
C	2.944866	3.208955	-0.357671
C	5.034126	0.722562	-0.577275
H	3.172848	-1.936390	-2.763950
C	5.087616	-1.211797	-2.107211
H	3.835212	-1.964370	0.398656
C	4.318339	-2.503863	2.428416
C	2.457571	-3.233557	3.782924
C	0.165476	-3.450327	2.875368
C	-0.687283	-3.929686	1.940340
C	-1.050884	-5.241618	-0.128500
C	0.090458	-4.739514	-2.199253
H	1.463397	-3.076662	-2.111448
H	0.616140	4.484711	1.841085
C	2.155180	4.180770	0.345919
C	3.844196	3.671642	-1.357906
C	5.778430	-0.273896	-1.294708
C	5.748226	1.614423	0.269913
H	5.658752	-1.946097	-2.668441
H	5.375127	-2.281869	2.314760
C	3.814014	-2.951482	3.652592
H	2.069614	-3.600580	4.729578
H	-0.190822	-3.436263	3.904279
H	-1.671285	-4.255905	2.269524
H	-1.761321	-5.841257	0.431989
C	-0.805451	-5.525498	-1.469338
H	0.281425	-4.949140	-3.247340
C	2.325520	5.560846	0.053609
C	3.975539	5.016455	-1.624440
H	4.427300	2.952262	-1.920511

C	7.193360	-0.312864	-1.167686
C	7.119077	1.540420	0.380361
H	5.200831	2.353866	0.842701
H	4.477794	-3.085242	4.501528
H	-1.312668	-6.359510	-1.945454
H	1.728959	6.285819	0.601554
C	3.218625	5.974032	-0.907451
H	4.665135	5.345978	-2.396102
H	7.744792	-1.066463	-1.723993
C	7.853262	0.575713	-0.350607
H	7.642657	2.227378	1.038901
H	3.339337	7.031054	-1.125064
H	8.934376	0.534715	-0.257349
O	-3.350932	-2.357893	-1.014984
C	-3.717606	-3.589953	-0.523788
O	-3.753735	-3.849234	0.655039
H	-2.995125	-1.721179	0.934857
O	-6.488346	2.259271	0.155796
C	-7.253924	3.159211	0.944130
H	-7.536808	4.053885	0.368135
H	-8.158521	2.621996	1.236203
H	-6.715360	3.473868	1.848321
H	-5.584000	3.752891	-0.944456
C	-4.091129	-4.499429	-1.661039
H	-4.256158	-5.507514	-1.280300
H	-5.008824	-4.131235	-2.133207
H	-3.301854	-4.500162	-2.416927

TS1b

B3LYP-D3 SCF energy: -3112.93024390 a.u.
B3LYP-D3 enthalpy: -3112.080296 a.u.
B3LYP-D3 Gibbs free energy: -3112.226193 a.u.
wB97X-D SCF energy in solution: -3114.13379381 a.u.
wB97X-D enthalpy: -3113.283846 a.u.
wB97X-D Gibbs free energy: -3113.429743 a.u.
Imaginary frequency: -178.1304 cm-1

Cartesian coordinates

ATOM	X	Y	Z
C	-1.092111	-1.207850	2.116915
C	-1.969250	-0.277711	2.176708
C	0.233644	-1.551016	2.451758
C	1.152029	-2.560416	2.191418
C	0.225816	-3.578999	0.063266
H	0.532917	-4.338874	-0.661816
C	-1.244681	-3.682412	0.322729
C	-2.017361	-2.626111	0.580437
C	-3.379498	-2.305390	0.714756
H	-3.838461	-2.084567	1.674235
O	-4.247207	-2.881786	-0.216123
C	-5.543102	-2.410309	-0.213218
O	-5.902314	-1.517391	0.514337
H	0.499273	-2.605078	-0.342387

C	1.017691	-3.822132	1.381389
H	0.486754	-4.576513	1.986353
Rh	-2.294719	-0.525721	0.082627
C	-3.878518	0.721531	0.179295
O	-4.833673	1.321414	0.339140
C	-1.826434	-5.074801	0.417412
H	-2.876948	-5.068604	0.714925
H	-1.262306	-5.689038	1.131585
H	-1.747095	-5.570024	-0.559300
C	-2.442092	-1.009602	-1.818462
O	-2.571448	-1.291943	-2.918551
P	-0.454241	1.018788	-0.402880
O	0.454829	0.777553	-1.778827
O	0.659311	0.820800	0.771161
N	-0.778282	2.658557	-0.524777
C	1.249518	-0.368098	-1.874120
C	2.000896	1.207155	0.695234
C	-2.085673	3.115064	-0.901677
C	0.235253	3.668496	-0.334455
C	0.858869	-1.339254	-2.821512
C	2.399041	-0.482788	-1.105619
C	2.850731	0.639591	-0.236565
C	2.417732	2.150943	1.655149
C	-2.604296	2.778762	-2.156484
C	-2.839144	3.875455	0.017442
C	0.126283	4.524702	0.781485
C	1.323158	3.752873	-1.206166
H	-0.017352	-1.149392	-3.430334
C	1.613135	-2.477089	-2.974368
C	3.137380	-1.717458	-1.189882
C	4.175675	1.199711	-0.344353
H	1.692262	2.505711	2.377261
C	3.708280	2.612002	1.624063
H	-1.982072	2.216921	-2.847613
C	-3.896652	3.167753	-2.507621
C	-4.138779	4.257899	-0.362936
C	-2.324511	4.265350	1.330487
C	-1.040375	4.535287	1.663439
C	1.179326	5.427780	1.019211
C	2.350047	4.660015	-0.950026
H	1.372673	3.081565	-2.055043
H	1.336761	-3.220530	-3.717190
C	2.747429	-2.708958	-2.152302
C	4.240025	-2.008485	-0.340425
C	4.605248	2.182150	0.609351
C	5.070406	0.853658	-1.393993
H	4.044654	3.346204	2.350683
H	-4.299414	2.897260	-3.479012
C	-4.663545	3.910793	-1.604947
H	-4.740489	4.834166	0.334638
H	-3.084685	4.458092	2.085751
H	-0.860257	4.919827	2.665829
H	1.117463	6.090491	1.878550
C	2.279001	5.494286	0.169016
H	3.208516	4.702346	-1.612928

C	3.498876	-3.910261	-2.260075
C	4.943499	-3.184697	-0.466883
H	4.521562	-1.293120	0.422260
C	5.910248	2.733100	0.505770
C	6.323835	1.420451	-1.476636
H	4.754279	0.143164	-2.148469
H	-5.670255	4.219019	-1.869969
H	3.079549	6.198415	0.375278
H	3.201959	-4.643524	-3.005925
C	4.582737	-4.141067	-1.444780
H	5.781758	-3.382942	0.194840
H	6.222747	3.469118	1.242360
C	6.757489	2.360333	-0.512666
H	6.983516	1.146111	-2.294795
H	5.156555	-5.058123	-1.543459
H	7.750685	2.793245	-0.586382
C	-2.644146	0.649621	3.116901
H	-2.491845	1.682902	2.780498
H	-3.724753	0.468280	3.146054
H	-2.236662	0.537901	4.126851
C	2.372964	-2.158685	2.805021
O	0.862429	-0.558426	3.195009
C	2.132981	-0.955340	3.403114
H	2.745076	-0.287115	3.990468
H	3.299018	-2.709282	2.771092
C	-6.373641	-3.150760	-1.223963
H	-7.398482	-2.780844	-1.190782
H	-5.955153	-3.006504	-2.226002
H	-6.353609	-4.224794	-1.013032
O	2.330318	-4.280369	1.119391
C	2.425283	-5.667530	0.849478
H	1.874726	-5.954613	-0.058177
H	3.483855	-5.878305	0.689480
H	2.054966	-6.267320	1.695071

TS1b-n-rot

B3LYP-D3 SCF energy: -3112.92816000 a.u.
B3LYP-D3 enthalpy: -3112.078042 a.u.
B3LYP-D3 Gibbs free energy: -3112.225015 a.u.
wB97X-D SCF energy in solution: -3114.13191214 a.u.
wB97X-D enthalpy: -3113.281794 a.u.
wB97X-D Gibbs free energy: -3113.428767 a.u.
Imaginary frequency: -197.1807 cm-1

Cartesian coordinates

ATOM	X	Y	Z
C	-1.403734	-1.567924	2.017814
C	-2.328099	-0.683798	2.118448
C	-0.120925	-1.925197	2.473649
C	0.888819	-2.825014	2.152283
C	0.310663	-3.439168	-0.237970
H	0.740356	-4.044204	-1.041913
C	-1.174716	-3.619545	-0.199237

C	-2.019859	-2.644482	0.148641
C	-3.408569	-2.394148	0.154406
H	-3.993425	-2.370223	1.069523
O	-4.125166	-2.824195	-0.965100
C	-5.413734	-2.346064	-1.076532
O	-5.880790	-1.563727	-0.285237
H	0.592035	-2.401997	-0.418801
C	0.946691	-3.904595	1.104598
H	0.403082	-4.790597	1.473485
Rh	-2.345328	-0.507934	-0.003916
C	-3.917866	0.735750	0.108968
O	-4.882668	1.332069	0.222760
C	-1.698960	-5.016211	-0.441767
H	-2.778364	-5.088214	-0.294409
H	-1.204716	-5.741743	0.218109
H	-1.470190	-5.318657	-1.472202
C	-2.282125	-0.641262	-1.971532
O	-2.304257	-0.742850	-3.109188
P	-0.406617	1.006023	-0.098511
O	0.531879	0.932227	-1.452292
O	0.615349	0.457397	1.075360
N	-0.527732	2.682616	-0.023424
C	1.396411	-0.139713	-1.670719
C	1.911908	0.959221	1.181109
C	0.231591	3.574349	-0.872392
C	-1.628938	3.251511	0.701086
C	1.107969	-0.963500	-2.780451
C	2.508924	-0.327697	-0.863832
C	2.846163	0.649049	0.210143
C	2.197897	1.765017	2.302519
C	1.557959	3.874379	-0.567610
C	-0.376638	4.072716	-2.042570
C	-2.743533	3.750701	-0.007035
C	-1.600413	3.260496	2.098378
H	0.259163	-0.714446	-3.406126
C	1.920072	-2.036404	-3.055562
C	3.308799	-1.502839	-1.089432
C	4.119211	1.315722	0.272434
H	1.424395	1.909413	3.048823
C	3.441535	2.338532	2.415698
H	1.992033	3.493026	0.347201
C	2.321377	4.638001	-1.449556
C	0.417989	4.829022	-2.924259
C	-1.792509	3.873217	-2.346706
C	-2.815684	3.750928	-1.468045
C	-3.829852	4.238011	0.744723
C	-2.687635	3.752869	2.820365
H	-0.718751	2.879860	2.603964
H	1.719082	-2.666389	-3.917909
C	3.015181	-2.352097	-2.208842
C	4.373613	-1.878544	-0.225166
C	4.414689	2.161916	1.394869
C	5.088289	1.211697	-0.762888
H	3.682628	2.961549	3.272678
H	3.360598	4.848144	-1.214966

C	1.751326	5.106660	-2.636426
H	-0.030150	5.211717	-3.837505
H	-2.055432	3.953027	-3.400014
H	-3.824162	3.745863	-1.878075
H	-4.703706	4.613434	0.219381
C	-3.806220	4.241308	2.136106
H	-2.661842	3.757844	3.906087
C	3.819858	-3.499559	-2.443669
C	5.129139	-3.001544	-0.476219
H	4.583365	-1.274088	0.649023
C	5.668703	2.827780	1.454573
C	6.287686	1.884224	-0.681098
H	4.869859	0.604471	-1.633358
H	2.343577	5.692114	-3.333391
H	-4.657728	4.626419	2.688997
H	3.596469	-4.123594	-3.305499
C	4.862270	-3.815280	-1.602708
H	5.936360	-3.268151	0.199933
H	5.882298	3.458159	2.314006
C	6.589315	2.692468	0.440946
H	7.008181	1.797134	-1.489192
H	5.475706	-4.690619	-1.796075
H	7.541920	3.211091	0.493553
C	-3.230763	-0.050503	3.110605
H	-3.186359	1.039723	3.014796
H	-4.270894	-0.351036	2.934427
H	-2.947030	-0.337368	4.128509
C	1.997327	-2.494502	2.982419
O	0.347085	-1.064303	3.460694
C	1.606640	-1.439962	3.756260
H	2.095978	-0.878798	4.538670
H	2.955900	-2.987660	2.969976
C	-6.085704	-2.922408	-2.291405
H	-7.109530	-2.552568	-2.348310
H	-5.532931	-2.635298	-3.192564
H	-6.082178	-4.015922	-2.240278
O	2.312943	-4.234445	0.937245
C	2.545102	-5.535575	0.425938
H	2.135587	-5.664358	-0.586172
H	3.628098	-5.656793	0.374227
H	2.119012	-6.307306	1.085217

TS1b-p-rot

B3LYP-D3 SCF energy: -3112.92083223 a.u.
B3LYP-D3 enthalpy: -3112.070407 a.u.
B3LYP-D3 Gibbs free energy: -3112.216360 a.u.
wB97X-D SCF energy in solution: -3114.13059539 a.u.
wB97X-D enthalpy: -3113.280170 a.u.
wB97X-D Gibbs free energy: -3113.426123 a.u.
Imaginary frequency: -208.1296 cm-1

Cartesian coordinates

ATOM	X	Y	Z

C	-1.635080	2.190588	0.108330
C	-1.064807	2.165097	-1.029372
C	-1.708159	2.914910	1.341137
C	-2.155201	2.718970	2.629157
C	-3.246673	0.409357	2.341620
H	-3.799230	-0.322913	2.940776
C	-4.112965	0.940567	1.229043
C	-3.612377	1.147258	0.012322
C	-3.891108	1.291444	-1.348852
H	-3.640016	2.183106	-1.915080
O	-4.960718	0.556993	-1.852097
C	-4.996063	0.385843	-3.221870
O	-4.120621	0.797179	-3.942963
H	-2.374246	-0.103061	1.932353
C	-2.775940	1.522383	3.304433
Rh	-1.949280	0.216612	-1.058135
C	-1.451044	-0.013592	-3.003818
O	-1.121483	-0.086583	-4.091297
C	-5.529980	1.348999	1.549852
H	-6.070222	1.685451	0.661968
H	-5.536753	2.160737	2.288851
H	-6.072383	0.504520	1.992721
C	-3.002780	-1.462367	-0.896833
O	-3.792155	-2.285406	-0.953348
P	0.167071	-0.697610	-0.255572
O	1.080755	0.137365	0.818653
O	1.092444	-0.665005	-1.633767
N	0.263176	-2.194704	0.513795
C	1.865775	1.240089	0.467149
C	2.442695	-1.034288	-1.579291
C	1.144001	-2.452504	1.635544
C	-0.686700	-3.220163	0.190487
C	1.545585	2.452105	1.111141
C	2.928962	1.113330	-0.411469
C	3.348707	-0.219390	-0.926514
C	2.801555	-2.247642	-2.201290
C	2.509793	-2.633983	1.425319
C	0.604706	-2.452497	2.938488
C	-1.690956	-3.563503	1.124618
C	-0.621400	-3.851259	-1.056727
H	0.749525	2.452336	1.842934
C	2.244783	3.590210	0.798753
C	3.611138	2.318481	-0.810235
C	4.683830	-0.721948	-0.738298
H	2.047475	-2.797182	-2.753725
C	4.096882	-2.695206	-2.100689
H	2.891332	-2.667988	0.414302
C	3.376379	-2.746292	2.511335
C	1.500971	-2.550564	4.019684
C	-0.831043	-2.416177	3.205340
C	-1.818529	-2.918051	2.429465
C	-2.617126	-4.555561	0.751650
C	-1.553948	-4.827367	-1.402237
H	0.173222	-3.573005	-1.740446
H	2.003433	4.528266	1.290255

C	3.264939	3.565315	-0.188937
C	4.601915	2.331542	-1.830702
C	5.055040	-1.971620	-1.340851
C	5.644270	-0.056252	0.072181
H	4.393457	-3.625147	-2.577627
H	4.441689	-2.861179	2.335426
C	2.870627	-2.687476	3.812970
H	1.103563	-2.539434	5.031360
H	-1.118558	-2.043828	4.186099
H	-2.822524	-2.926025	2.849796
H	-3.400034	-4.829485	1.453689
C	-2.552164	-5.182060	-0.489163
H	-1.500128	-5.309730	-2.373421
C	3.940982	4.751874	-0.579784
C	5.228769	3.502803	-2.196028
H	4.858016	1.406699	-2.333994
C	6.369195	-2.476485	-1.146732
C	6.904027	-0.582887	0.254441
H	5.372938	0.871063	0.562418
H	3.540935	-2.760619	4.664151
H	-3.280498	-5.944759	-0.747399
H	3.674458	5.686337	-0.092469
C	4.906011	4.724964	-1.560335
H	5.976417	3.488074	-2.983692
H	6.639333	-3.418018	-1.617811
C	7.277937	-1.798101	-0.367673
H	7.617716	-0.061109	0.885378
H	5.414210	5.638437	-1.854387
H	8.277696	-2.195635	-0.220737
C	-0.145865	2.807737	-1.992998
H	0.712827	2.156644	-2.187539
H	-0.646787	2.999089	-2.948805
H	0.216608	3.753403	-1.577978
C	-1.831211	3.922919	3.342701
O	-1.145058	4.169210	1.231258
C	-1.230432	4.758771	2.455859
H	-0.836163	5.761683	2.520688
H	-2.027561	4.110597	4.389026
C	-6.228405	-0.373917	-3.623025
H	-6.228234	-1.357562	-3.140961
H	-7.124084	0.156627	-3.284356
H	-6.246707	-0.487565	-4.706973
O	-1.929899	0.965971	4.311776
C	-0.553675	0.830755	3.975202
H	-0.394946	0.346869	3.004130
H	-0.041464	1.801731	3.973054
H	-0.110754	0.202320	4.750858
H	-3.645451	1.881040	3.867526

TS1b-i2

B3LYP-D3 SCF energy: -3112.92542685 a.u.
B3LYP-D3 enthalpy: -3112.075206 a.u.
B3LYP-D3 Gibbs free energy: -3112.220937 a.u.

wB97X-D SCF energy in solution: -3114.12955118 a.u.
wB97X-D enthalpy: -3113.279330 a.u.
wB97X-D Gibbs free energy: -3113.425061 a.u.
Imaginary frequency: -214.9076 cm-1

Cartesian coordinates

ATOM	X	Y	Z
C	-3.086777	2.500461	-0.196034
C	-4.044794	1.646437	-0.132183
C	-2.676079	3.650942	-0.920665
C	-1.590744	4.511831	-1.015984
C	0.179275	3.122734	0.146576
H	1.233936	3.165560	0.435711
C	-0.615466	2.636003	1.317542
C	-1.636263	1.780740	1.190712
C	-2.366813	0.867735	1.980919
H	-3.389903	1.041810	2.303995
O	-1.598803	0.131935	2.891176
C	-2.139558	-1.035433	3.371226
O	-3.240551	-1.421017	3.060803
H	0.098678	2.439608	-0.701412
C	-0.267551	4.534386	-0.293079
H	-0.357278	5.178708	0.598816
Rh	-2.450935	0.209040	-0.071565
C	-0.358100	3.274243	2.664791
H	-0.703951	2.641034	3.485400
H	-0.893919	4.231337	2.730528
H	0.706693	3.479376	2.801073
C	-2.369431	0.185156	-2.094320
O	-2.368953	0.254202	-3.233035
C	-5.513547	1.479241	-0.085673
H	-5.848465	0.822634	-0.897333
H	-5.830678	1.014219	0.855682
H	-6.011310	2.449523	-0.186412
C	-1.908612	5.436106	-2.055982
O	-3.618541	4.010209	-1.874280
C	-3.140684	5.093039	-2.526281
H	-1.266680	6.229858	-2.404267
H	-3.785091	5.506211	-3.288264
P	-0.311195	-0.906285	-0.090371
O	0.759184	-0.917739	1.148861
O	0.472381	-0.010272	-1.248180
N	-0.227923	-2.546661	-0.455426
C	1.686157	0.084818	1.428854
C	1.756117	-0.384072	-1.657296
C	0.753016	-3.438499	0.126697
C	-1.347502	-3.173456	-1.101338
C	1.634987	0.601449	2.741274
C	2.646900	0.474151	0.508117
C	2.822718	-0.227142	-0.793393
C	1.880427	-0.918072	-2.956134
C	2.030056	-3.529737	-0.422017
C	0.403367	-4.165908	1.283352
C	-2.273892	-3.900472	-0.323320
C	-1.526873	-3.013509	-2.477420

H	0.883561	0.212724	3.415523
C	2.516420	1.581258	3.120965
C	3.478115	1.600548	0.854508
C	4.088621	-0.787706	-1.189383
H	1.003020	-0.949297	-3.593154
C	3.106523	-1.371724	-3.379050
H	2.255146	-2.990649	-1.331942
C	3.009609	-4.293308	0.211540
C	1.413225	-4.916314	1.914769
C	-0.958336	-4.202223	1.811976
C	-2.115651	-4.097017	1.117327
C	-3.402229	-4.429077	-0.979958
C	-2.650496	-3.549486	-3.104699
H	-0.776788	-2.468245	-3.041238
H	2.495254	1.972448	4.134523
C	3.420469	2.144070	2.181043
C	4.331907	2.234416	-0.090557
C	4.224812	-1.354878	-2.501909
C	5.204000	-0.858112	-0.310313
H	3.225101	-1.780051	-4.378784
H	4.009705	-4.336745	-0.209109
C	2.702339	-4.975343	1.392289
H	1.167389	-5.473189	2.815332
H	-1.041528	-4.459220	2.866070
H	-3.040678	-4.257654	1.666485
H	-4.137474	-4.977197	-0.397393
C	-3.591168	-4.257196	-2.348017
H	-2.792098	-3.415807	-4.172835
C	4.237888	3.256154	2.518263
C	5.095937	3.326158	0.262023
H	4.367488	1.860818	-1.106999
C	5.469075	-1.919377	-2.892538
C	6.390381	-1.430178	-0.714416
H	5.110933	-0.470500	0.697083
H	3.463462	-5.561812	1.898312
H	-4.469710	-4.678123	-2.827710
H	4.190990	3.647497	3.531426
C	5.060634	3.838514	1.580841
H	5.730687	3.799105	-0.481696
H	5.559973	-2.335310	-3.892601
C	6.532782	-1.956147	-2.020553
H	7.224763	-1.481822	-0.020986
H	5.676307	4.692610	1.846733
H	7.477024	-2.396369	-2.326703
C	-3.781789	-1.260955	0.260380
O	-4.707027	-1.900336	0.450929
C	-1.156509	-1.727927	4.273952
H	-0.798117	-1.042723	5.048315
H	-1.632210	-2.595154	4.731991
H	-0.294268	-2.047107	3.678220
O	0.669334	5.112758	-1.186970
C	1.835901	5.633027	-0.565947
H	2.460438	4.848461	-0.115869
H	2.410290	6.124380	-1.354393
H	1.581834	6.373179	0.208610

TS1b-i2-p-rot

B3LYP-D3 SCF energy: -3112.91573932 a.u.
B3LYP-D3 enthalpy: -3112.065716 a.u.
B3LYP-D3 Gibbs free energy: -3112.213071 a.u.
wB97X-D SCF energy in solution: -3114.12195451 a.u.
wB97X-D enthalpy: -3113.271931 a.u.
wB97X-D Gibbs free energy: -3113.419286 a.u.
Imaginary frequency: -233.1917 cm-1

Cartesian coordinates

ATOM	X	Y	Z
C	-3.522804	-2.234274	0.133171
C	-2.544025	-3.067842	0.195210
C	-4.699521	-1.983510	-0.596932
C	-5.714806	-1.037415	-0.631668
C	-4.516979	0.970492	0.305853
C	-3.681827	0.419948	1.419746
C	-2.759262	-0.540506	1.264894
C	-1.791436	-1.169511	2.090229
H	-1.959544	-2.155005	2.517429
O	-1.029721	-0.334351	2.908553
C	0.036782	-0.936824	3.535819
O	0.311978	-2.100600	3.373410
H	-3.996744	0.930231	-0.653798
C	-5.872402	0.210685	0.186614
H	-6.226889	-0.052921	1.196706
Rh	-1.191152	-1.431194	0.058877
C	-4.031592	0.904274	2.807697
H	-3.454610	0.400310	3.584882
H	-5.101210	0.755428	3.011437
H	-3.837281	1.980773	2.874826
C	-1.356817	-1.351248	-1.971978
O	-1.537034	-1.306286	-3.098274
C	-2.266465	-4.514475	0.358611
H	-1.611909	-4.867575	-0.446609
H	-1.745194	-4.706636	1.304408
H	-3.196327	-5.092415	0.342521
C	-6.571924	-1.403966	-1.706144
O	-4.904245	-2.894829	-1.628298
C	-6.040396	-2.535010	-2.258710
H	-7.455906	-0.869408	-2.015708
H	-6.352892	-3.175894	-3.070397
P	0.391026	0.396662	-0.072684
O	1.398620	0.133522	-1.345273
O	1.381752	0.184574	1.239942
N	0.159019	2.035227	-0.360768
C	2.279437	-0.949051	-1.325276
C	2.664844	0.745451	1.244222
C	0.890446	2.752973	-1.387275
C	-0.908539	2.760242	0.269021
C	2.043101	-1.980060	-2.259899
C	3.350090	-0.956437	-0.446553

C	3.635703	0.237570	0.398470
C	2.900315	1.816083	2.131723
C	2.211460	3.141800	-1.171781
C	0.255954	2.986517	-2.623409
C	-2.016303	3.165564	-0.505230
C	-0.823521	3.081404	1.626131
H	1.226972	-1.868829	-2.964587
C	2.859230	-3.084625	-2.261666
C	4.156812	-2.144970	-0.385294
C	4.895831	0.928433	0.334072
H	2.104477	2.133815	2.794566
C	4.128508	2.432750	2.133199
H	2.667943	2.966703	-0.206435
C	2.943644	3.723099	-2.205705
C	1.020587	3.556403	-3.658071
C	-1.166168	2.724934	-2.840690
C	-2.155653	2.829610	-1.923575
C	-3.015430	3.925577	0.132688
C	-1.829274	3.827904	2.236735
H	0.044258	2.752759	2.186341
H	2.697650	-3.882758	-2.981051
C	3.907174	-3.213289	-1.311770
C	5.181130	-2.322633	0.584834
C	5.140489	2.030668	1.221823
C	5.903313	0.594631	-0.612933
H	4.326570	3.256314	2.813599
H	3.978975	4.003554	-2.036910
C	2.349743	3.917116	-3.456112
H	0.551241	3.731695	-4.622552
H	-1.461305	2.561745	-3.875545
H	-3.180641	2.753267	-2.282736
H	-3.868605	4.260882	-0.451581
C	-2.922767	4.262633	1.480141
H	-1.755396	4.079127	3.290583
C	4.708877	-4.384791	-1.256592
C	5.930524	-3.477754	0.619575
H	5.364691	-1.539676	1.311346
C	6.383340	2.716965	1.157290
C	7.090806	1.290828	-0.659049
H	5.725941	-0.212040	-1.313997
H	-3.700489	4.864335	1.941710
H	4.515292	-5.181473	-1.970265
C	5.702494	-4.516552	-0.314218
H	6.702433	-3.594635	1.374635
H	6.557722	3.542365	1.842706
C	7.342002	2.355444	0.239394
H	7.841860	1.022203	-1.396216
H	6.306446	-5.418245	-0.277660
H	8.286533	2.889334	0.194733
C	0.339646	-2.676435	0.416832
O	1.095053	-3.486408	0.686801
H	2.919827	4.355945	-4.269543
H	-4.744209	2.017986	0.516156
C	0.761766	0.027170	4.433632
H	0.367319	1.040471	4.342751

H	0.665690	-0.311298	5.470408
H	1.823840	0.011451	4.175964
O	-6.846610	1.009446	-0.461100
C	-7.514619	1.922841	0.394056
H	-6.825047	2.651779	0.846029
H	-8.235370	2.460404	-0.225523
H	-8.050219	1.399570	1.200555

TS1b-i3

B3LYP-D3 SCF energy: -3112.91857194 a.u.
B3LYP-D3 enthalpy: -3112.068072 a.u.
B3LYP-D3 Gibbs free energy: -3112.214679 a.u.
wB97X-D SCF energy in solution: -3114.12951438 a.u.
wB97X-D enthalpy: -3113.279014 a.u.
wB97X-D Gibbs free energy: -3113.425621 a.u.
Imaginary frequency: -207.1053 cm-1

Cartesian coordinates

ATOM	X	Y	Z
C	-3.335030	0.263820	1.235201
C	-2.268591	-0.309243	1.651805
C	-4.748218	0.281726	1.405549
C	-5.868684	0.877021	0.854937
C	-4.889749	1.823151	-1.319729
H	-5.234087	2.362044	-2.210047
C	-3.678737	2.505763	-0.750554
C	-2.672884	1.832435	-0.195269
C	-1.310465	1.914727	0.138632
H	-0.984068	1.979180	1.170952
O	-0.439448	2.549689	-0.769991
C	-0.027332	3.814653	-0.414746
O	-0.456711	4.385107	0.559317
H	-4.647260	0.797717	-1.612219
C	-6.060785	1.823550	-0.311919
Rh	-1.743575	-0.132761	-0.410095
C	-1.442499	0.268126	-2.336316
O	-1.260378	0.511890	-3.434894
C	-3.655018	4.017358	-0.753473
H	-2.821351	4.417788	-0.173504
H	-4.590892	4.419377	-0.345746
H	-3.578743	4.379771	-1.787623
C	-2.726510	-1.851621	-0.817789
O	-3.427917	-2.732062	-0.999217
P	0.545166	-0.835505	-0.103777
O	1.397845	-0.334982	1.206660
O	1.319053	-0.077578	-1.360606
N	0.959477	-2.466060	-0.017809
C	1.889099	0.970681	1.309944
C	2.711796	-0.135525	-1.472611
C	2.014865	-2.994842	0.822857
C	0.139457	-3.415871	-0.716185
C	1.345715	1.764042	2.342896
C	2.893780	1.416260	0.468212

C	3.507245	0.510997	-0.542884
C	3.234848	-0.878053	-2.551773
C	3.351743	-2.793732	0.485087
C	1.665914	-3.667918	2.012144
C	-0.674874	-4.310100	0.014652
C	0.119742	-3.409318	-2.114923
H	0.604155	1.329929	3.003986
C	1.764295	3.062297	2.491556
C	3.292472	2.796990	0.574736
C	4.922911	0.261279	-0.588012
H	2.548737	-1.302365	-3.276705
C	4.594932	-1.044835	-2.656295
H	3.596392	-2.317592	-0.453082
C	4.365055	-3.186785	1.358214
C	2.704836	-4.042059	2.885577
C	0.296884	-4.057568	2.337809
C	-0.703731	-4.355109	1.475547
C	-1.505583	-5.181397	-0.715819
C	-0.716661	-4.278875	-2.813849
H	0.770196	-2.719378	-2.642709
H	1.342334	3.687282	3.272584
C	2.718616	3.619804	1.600818
C	4.203460	3.402851	-0.335205
C	5.465593	-0.520998	-1.662991
C	5.809641	0.715556	0.426698
H	5.015452	-1.611032	-3.482651
H	5.401009	-2.998041	1.093062
C	4.038450	-3.798066	2.571123
H	2.451448	-4.549330	3.812819
H	0.108106	-4.246867	3.393169
H	-1.617906	-4.764595	1.902110
H	-2.143975	-5.872379	-0.171899
C	-1.529455	-5.170438	-2.107294
H	-0.730421	-4.263247	-3.899451
C	3.087811	4.988570	1.689625
C	4.537161	4.735045	-0.225099
H	4.630276	2.809747	-1.134859
C	6.862521	-0.779609	-1.700638
C	7.156253	0.431518	0.369243
H	5.412996	1.280222	1.262318
H	4.820467	-4.095141	3.263433
H	-2.181853	-5.854516	-2.641360
H	2.638820	5.597194	2.469737
C	3.980963	5.538806	0.798504
H	5.229618	5.174843	-0.937012
H	7.261033	-1.364912	-2.525269
C	7.693473	-0.313774	-0.707768
H	7.812847	0.779871	1.161192
H	4.254295	6.587063	0.873039
H	8.758469	-0.522895	-0.742380
C	-1.584030	-0.909837	2.814961
H	-1.473022	-1.988938	2.661176
H	-0.572772	-0.512781	2.940438
H	-2.164988	-0.731641	3.726045
C	-6.983578	0.383082	1.605095

O	-5.138691	-0.564411	2.432467
C	-6.485565	-0.470527	2.540469
H	-8.017342	0.640655	1.429582
H	-6.936939	-1.060828	3.324116
C	0.963560	4.351197	-1.409466
H	1.564793	3.548135	-1.837891
H	0.417135	4.848883	-2.219548
H	1.603334	5.082600	-0.913974
O	-7.311883	1.590930	-0.944118
C	-7.431781	0.370533	-1.667035
H	-7.169219	-0.501456	-1.051753
H	-8.480898	0.294842	-1.960381
H	-6.813125	0.368296	-2.575038
H	-6.170954	2.838613	0.088506

TS1b-i3-p-rot

B3LYP-D3 SCF energy: -3112.90936778 a.u.
B3LYP-D3 enthalpy: -3112.059008 a.u.
B3LYP-D3 Gibbs free energy: -3112.207191 a.u.
wB97X-D SCF energy in solution: -3114.12153524 a.u.
wB97X-D enthalpy: -3113.271175 a.u.
wB97X-D Gibbs free energy: -3113.419358 a.u.
Imaginary frequency: -191.8055 cm-1

Cartesian coordinates

ATOM	X	Y	Z
C	-2.932208	-0.903241	1.545821
C	-1.880345	-0.303018	1.956298
C	-3.738585	-2.051853	1.742764
C	-4.849349	-2.669220	1.189546
C	-4.953704	-1.521941	-1.097738
H	-5.565901	-1.558555	-2.006571
C	-4.772634	-0.093363	-0.670922
C	-3.638733	0.354079	-0.129753
C	-2.904791	1.545530	0.071703
H	-2.796983	2.023111	1.040683
O	-2.945355	2.459028	-1.011301
C	-3.767987	3.542790	-0.842497
O	-4.384682	3.749545	0.175244
H	-3.986141	-1.982118	-1.317959
C	-5.699544	-2.344314	-0.021339
Rh	-1.525769	-0.084642	-0.166855
C	-1.507313	0.209099	-2.117179
O	-1.516767	0.425859	-3.237708
C	-5.962438	0.828476	-0.812219
H	-5.822657	1.771301	-0.280654
H	-6.872594	0.347119	-0.433218
H	-6.138690	1.041382	-1.875738
C	-0.802493	-2.003018	-0.238273
O	-0.518360	-3.103364	-0.160205
P	0.757500	0.738504	0.089673
O	1.731677	0.362815	-1.182738
O	1.351858	-0.241003	1.294240

N	1.281719	2.317803	0.298040
C	2.020297	-0.976564	-1.448489
C	2.731577	-0.352754	1.495753
C	2.429739	2.867530	-0.390589
C	0.386306	3.246653	0.931736
C	1.455375	-1.529536	-2.618236
C	2.854966	-1.687486	-0.601366
C	3.506423	-1.007937	0.554861
C	3.258121	0.211006	2.675828
C	3.717334	2.605296	0.073422
C	2.217711	3.610061	-1.570107
C	-0.384121	4.119957	0.135118
C	0.261343	3.237893	2.321852
H	0.871213	-0.888252	-3.268670
C	1.682948	-2.851712	-2.914152
C	3.035335	-3.090056	-0.863240
C	4.933124	-0.980151	0.728033
H	2.578355	0.668395	3.387178
C	4.615400	0.172399	2.888848
H	3.848255	2.041080	0.987543
C	4.823668	3.044642	-0.652032
C	3.350742	4.031086	-2.291793
C	0.889088	3.984499	-2.047162
C	-0.226743	4.210900	-1.314625
C	-1.325915	4.935033	0.791306
C	-0.677359	4.058306	2.948875
H	0.902182	2.576809	2.897011
H	1.267267	-3.289737	-3.817524
C	2.446440	-3.668716	-2.038886
C	3.749943	-3.946235	0.019570
C	5.484846	-0.387929	1.914710
C	5.831849	-1.479771	-0.254152
H	5.040373	0.597587	3.793878
H	5.823549	2.818483	-0.294107
C	4.638134	3.751808	-1.843155
H	3.207763	4.595795	-3.209357
H	0.830888	4.213778	-3.109709
H	-1.085747	4.609822	-1.845897
H	-1.939430	5.610473	0.202098
C	-1.482007	4.899514	2.174195
H	-0.780044	4.041333	4.029811
C	2.630609	-5.052928	-2.301769
C	3.897900	-5.286999	-0.259038
H	4.176460	-3.535356	0.926970
C	6.895320	-0.355307	2.085484
C	7.194516	-1.418972	-0.062971
H	5.434515	-1.902420	-1.169534
H	5.495510	4.087483	-2.418853
H	-2.223978	5.534645	2.647890
H	2.189374	-5.474356	-3.201383
C	3.343789	-5.847732	-1.434579
H	4.443724	-5.921922	0.432797
H	7.299233	0.087145	2.992471
C	7.735255	-0.861252	1.120336
H	7.862001	-1.799339	-0.830729

H	3.476313	-6.905048	-1.643636
H	8.811745	-0.826806	1.258777
C	-1.196883	0.168937	3.179424
H	-0.142198	-0.114930	3.158679
H	-1.248288	1.260625	3.250553
H	-1.680750	-0.268840	4.059424
C	-5.088877	-3.831389	1.985713
O	-3.284078	-2.809638	2.814056
C	-4.124581	-3.859136	2.948039
H	-5.882313	-4.545615	1.825205
H	-3.914723	-4.529094	3.768861
C	-3.814476	4.395993	-2.084625
H	-4.849907	4.432488	-2.438055
H	-3.526407	5.420320	-1.829351
H	-3.170552	4.009993	-2.875838
O	-6.284547	-3.524595	-0.552060
C	-5.400155	-4.420016	-1.217965
H	-4.538029	-4.692055	-0.592883
H	-5.982955	-5.319097	-1.428707
H	-5.037003	-4.006785	-2.168793
H	-6.567490	-1.764963	0.315875

TS2a

B3LYP-D3 SCF energy: -3112.92495654 a.u.
B3LYP-D3 enthalpy: -3112.075136 a.u.
B3LYP-D3 Gibbs free energy: -3112.222086 a.u.
wB97X-D SCF energy in solution: -3114.13462906 a.u.
wB97X-D enthalpy: -3113.284809 a.u.
wB97X-D Gibbs free energy: -3113.431759 a.u.
Imaginary frequency: -228.8362 cm-1

Cartesian coordinates

ATOM	X	Y	Z
C	-0.812636	-2.472403	-0.513733
C	-1.482918	-2.147788	-1.551965
C	0.338621	-3.195392	-0.071282
C	1.110489	-3.316523	1.062985
C	-0.203766	-1.801745	2.685044
H	-0.201555	-1.527996	3.746529
C	-1.468561	-2.554926	2.378676
C	-2.116468	-2.376098	1.225211
C	-3.377053	-2.455593	0.620646
H	-3.590056	-3.087074	-0.236231
O	-4.471253	-2.192565	1.439631
C	-5.671799	-1.958710	0.797877
O	-5.760449	-1.920985	-0.404424
H	-0.158514	-0.882917	2.102590
C	1.093262	-2.589633	2.379515
H	1.249420	-3.340683	3.172576
Rh	-2.262587	-0.711459	-0.191879
C	-3.654486	-0.148555	-1.529834
O	-4.426852	0.061969	-2.340322
C	-1.954934	-3.583800	3.368919

H	-2.895166	-4.045249	3.058619
H	-1.202116	-4.373960	3.490787
H	-2.098173	-3.120075	4.352972
C	-2.787995	0.399154	1.365407
O	-3.123936	0.951357	2.306083
P	-0.445136	0.890982	-0.587271
O	0.635914	0.794706	-1.836166
O	0.512503	0.641865	0.714984
N	-0.847778	2.514276	-0.632020
C	1.566855	-0.247016	-1.923603
C	1.820990	1.120469	0.866931
C	-2.125876	2.920638	-1.149445
C	0.056427	3.564112	-0.231637
C	1.434260	-1.105423	-3.034769
C	2.599843	-0.349956	-1.005787
C	2.831036	0.701091	0.022969
C	2.020108	1.997714	1.950315
C	-2.419796	2.734703	-2.502117
C	-3.070311	3.486560	-0.266567
C	-0.274235	4.326433	0.908930
C	1.250103	3.776465	-0.924440
H	0.632684	-0.916847	-3.739657
C	2.338563	-2.120582	-3.218252
C	3.469246	-1.494669	-1.119343
C	4.111997	1.351274	0.164001
H	1.172962	2.244456	2.579594
C	3.263575	2.537269	2.155200
H	-1.650309	2.332956	-3.155352
C	-3.686958	3.051172	-2.991456
C	-4.350776	3.770197	-0.779054
C	-2.749145	3.826792	1.118707
C	-1.544956	4.189761	1.620026
C	0.668130	5.271986	1.356441
C	2.164057	4.720445	-0.459510
H	1.467950	3.174278	-1.798517
H	2.266526	-2.769742	-4.086526
C	3.362013	-2.354259	-2.262025
C	4.430892	-1.820882	-0.124894
C	4.326746	2.258850	1.255422
C	5.171605	1.171217	-0.767719
H	3.435125	3.219650	2.982831
H	-3.919585	2.891461	-4.039658
C	-4.657990	3.554266	-2.120536
H	-5.100482	4.190419	-0.113683
H	-3.603931	3.895198	1.788674
H	-1.528083	4.524134	2.655906
H	0.437241	5.864653	2.237876
C	1.872397	5.465314	0.686813
H	3.105411	4.861114	-0.981258
C	4.268703	-3.438863	-2.406179
C	5.283377	-2.891232	-0.285373
H	4.473866	-1.225842	0.777968
C	5.589275	2.893714	1.400628
C	6.378288	1.817309	-0.608983
H	5.021760	0.526363	-1.624936

H	-5.650886	3.790536	-2.490972
H	2.583695	6.198439	1.055280
H	4.191627	-4.065864	-3.291257
C	5.220825	-3.697221	-1.445922
H	6.009294	-3.120159	0.489679
H	5.736528	3.569160	2.239713
C	6.599665	2.676951	0.492228
H	7.166086	1.668563	-1.341840
H	5.911591	-4.526498	-1.568154
H	7.558736	3.172813	0.608958
C	-1.775552	-2.407989	-2.977289
H	-1.724625	-1.484819	-3.563984
H	-1.053169	-3.127829	-3.376152
H	-2.786945	-2.815328	-3.098793
C	2.116949	-4.287095	0.747296
O	0.812918	-4.044540	-1.047927
C	1.886288	-4.697521	-0.526665
H	2.373756	-5.398695	-1.184531
H	2.917710	-4.604553	1.398744
C	-6.772307	-1.758023	1.800944
H	-7.709600	-1.571401	1.276680
H	-6.866957	-2.645231	2.435510
H	-6.531124	-0.912861	2.454553
O	2.216023	-1.710771	2.350005
C	2.594775	-1.165792	3.603787
H	3.533260	-0.632762	3.435450
H	1.857059	-0.447650	3.987905
H	2.752908	-1.954433	4.355705

TS2a-n-rot

B3LYP-D3 SCF energy: -3112.92369297 a.u.
B3LYP-D3 enthalpy: -3112.073758 a.u.
B3LYP-D3 Gibbs free energy: -3112.221678 a.u.
wB97X-D SCF energy in solution: -3114.13344708 a.u.
wB97X-D enthalpy: -3113.283512 a.u.
wB97X-D Gibbs free energy: -3113.431432 a.u.
Imaginary frequency: -213.1645 cm-1

Cartesian coordinates

ATOM	X	Y	Z
C	1.325206	-2.052303	1.280527
C	1.876949	-1.155652	1.996767
C	0.430223	-3.169489	1.328923
C	-0.308993	-3.949148	0.468271
C	0.460508	-2.943792	-1.759624
H	0.310494	-3.061884	-2.839098
C	1.904920	-3.200893	-1.426848
C	2.560449	-2.440480	-0.551938
C	3.821432	-2.018848	-0.128218
H	4.206165	-2.198248	0.871066
O	4.789507	-1.877111	-1.119150
C	5.942542	-1.206136	-0.762827
O	6.088096	-0.713935	0.328386

H	0.169906	-1.926295	-1.494147
C	-0.490831	-3.913107	-1.025057
H	-0.317937	-4.935929	-1.402445
Rh	2.346369	-0.350456	0.075137
C	3.669444	0.886558	0.954715
O	4.423481	1.461726	1.587386
C	2.576730	-4.416001	-2.020235
H	3.632516	-4.475841	-1.746408
H	2.077808	-5.333252	-1.681739
H	2.498313	-4.389762	-3.114206
C	2.535846	0.123074	-1.836614
O	2.660206	0.354079	-2.948014
P	0.281716	0.939006	0.095750
O	-0.825153	0.742013	1.277419
O	-0.488473	0.356144	-1.252397
N	0.309180	2.620986	0.078893
C	-1.787155	-0.272432	1.303554
C	-1.744851	0.872563	-1.595754
C	-0.658332	3.431871	0.788203
C	1.512564	3.278881	-0.346864
C	-1.757439	-1.099724	2.444531
C	-2.749057	-0.384474	0.313061
C	-2.842764	0.601018	-0.800092
C	-1.802360	1.673681	-2.754472
C	-1.886294	3.728221	0.201894
C	-0.354009	3.859986	2.096372
C	2.420874	3.755188	0.623579
C	1.791053	3.384023	-1.711576
H	-0.998910	-0.916892	3.196727
C	-2.683962	-2.101918	2.574929
C	-3.655442	-1.501910	0.392758
C	-4.057479	1.320325	-1.083865
H	-0.905013	1.788584	-3.353361
C	-2.987983	2.281104	-3.091665
H	-2.076051	3.416699	-0.815858
C	-2.865047	4.400948	0.932371
C	-1.362394	4.523339	2.820148
C	0.967289	3.691436	2.696197
C	2.163903	3.668074	2.061128
C	3.634766	4.302293	0.164145
C	2.997293	3.936057	-2.140891
H	1.053352	3.025587	-2.422839
H	-2.681048	-2.736855	3.455280
C	-3.625731	-2.354371	1.543944
C	-4.572977	-1.817892	-0.646610
C	-4.125964	2.156754	-2.249327
C	-5.184048	1.285464	-0.216800
H	-3.055238	2.896395	-3.984546
H	-3.829393	4.602518	0.475830
C	-2.605450	4.785606	2.250641
H	-1.152083	4.848686	3.835634
H	0.989448	3.715425	3.784361
H	3.055302	3.671241	2.685120
H	4.357737	4.658083	0.893114
C	3.921909	4.391970	-1.195067

```
H        3.215002    4.007466   -3.202194
C       -4.520238   -3.454206    1.624769
C       -5.422251   -2.897516   -0.542474
H       -4.594398   -1.205971   -1.540285
C       -5.320741    2.876433   -2.522298
C       -6.319267    2.012566   -0.499633
H       -5.137828    0.689027    0.686413
H       -3.367449    5.297724    2.830603
H        4.864752    4.822516   -1.518953
H       -4.485023   -4.087037    2.507973
C       -5.403978   -3.723973    0.605911
H       -6.112326   -3.117912   -1.352292
H       -5.361757    3.496811   -3.413900
C       -6.397489    2.807137   -1.668439
H       -7.162468    1.978841    0.184152
H       -6.082867   -4.568724    0.676468
H       -7.302696    3.367668   -1.882073
C        2.077423   -0.578754    3.343515
H        1.641436    0.426505    3.390888
H        1.599122   -1.216762    4.094600
H        3.142639   -0.488161    3.584469
C       -0.999332   -4.896629    1.294313
O        0.239536   -3.600469    2.625202
C       -0.630652   -4.646497    2.577151
H       -0.872650   -5.097538    3.527600
H       -1.695275   -5.646709    0.947482
C        6.913853   -1.188157   -1.909154
H        7.821293   -0.666078   -1.605511
H        7.152902   -2.211814   -2.214970
H        6.461838   -0.686153   -2.771453
O       -1.847668   -3.561694   -1.255688
C       -2.353917   -3.923824   -2.530314
H       -3.424209   -3.713220   -2.500227
H       -1.900447   -3.340070   -3.344653
H       -2.200291   -4.994792   -2.735388
```

```
 TS2a-p-rot
B3LYP-D3 SCF energy:                 -3112.92791510 a.u.
B3LYP-D3 enthalpy:                   -3112.077429 a.u.
B3LYP-D3 Gibbs free energy:          -3112.222301 a.u.
wB97X-D SCF energy in solution:      -3114.13510235 a.u.
wB97X-D enthalpy:                    -3113.284616 a.u.
wB97X-D Gibbs free energy:           -3113.429488 a.u.
Imaginary frequency:                  -210.8535 cm-1

Cartesian coordinates
ATOM       X           Y           Z
C       -1.054965   -2.337317   -0.784116
C       -1.395759   -1.729700   -1.858158
C       -0.024961   -3.112163   -0.187573
C        0.299136   -3.662614    1.039863
C       -1.410968   -2.481851    2.547286
H       -1.676833   -2.429432    3.609552
```

C	-2.631722	-2.784125	1.725091
C	-2.816869	-2.250539	0.515975
C	-3.827114	-1.977187	-0.421117
H	-3.852526	-2.420283	-1.411989
O	-5.081825	-1.657165	0.097592
C	-5.955916	-1.033505	-0.766749
O	-5.643624	-0.733427	-1.893562
H	-0.975739	-1.525827	2.247898
C	-0.358207	-3.603534	2.401889
Rh	-2.312306	-0.379298	-0.479988
C	-3.044690	0.704834	-2.005611
O	-3.434401	1.229765	-2.939719
C	-3.622960	-3.787272	2.265066
H	-4.445654	-3.968459	1.570063
H	-3.133597	-4.744298	2.486152
H	-4.041300	-3.417306	3.210081
C	-3.232332	0.512785	1.035554
O	-3.900898	0.867374	1.890267
P	-0.225891	0.835132	-0.154562
O	0.842847	0.911704	-1.393320
O	0.597858	-0.034040	0.992755
N	-0.207457	2.471963	0.247101
C	1.778344	-0.076056	-1.693919
C	1.870580	0.408326	1.375535
C	0.680027	3.419293	-0.390920
C	-1.340304	3.039266	0.924869
C	1.697977	-0.611312	-2.996501
C	2.772286	-0.429373	-0.795842
C	2.933934	0.288517	0.499363
C	1.987021	0.975493	2.661648
C	1.973304	3.601911	0.093276
C	0.224900	4.100690	-1.538615
C	-2.369670	3.641120	0.165381
C	-1.412102	2.979666	2.318747
H	0.941299	-0.220721	-3.666195
C	2.588076	-1.577038	-3.390765
C	3.647426	-1.512127	-1.166223
C	4.176517	0.914993	0.872453
H	1.114915	0.986863	3.305981
C	3.196662	1.486301	3.065549
H	2.281384	3.080424	0.989815
C	2.861271	4.431499	-0.589815
C	1.144627	4.920504	-2.219757
C	-1.153408	4.014071	-2.016640
C	-2.276265	3.820320	-1.282460
C	-3.502116	4.110207	0.858708
C	-2.529699	3.479757	2.984049
H	-0.583108	2.539266	2.862895
H	2.544859	-1.985283	-4.396728
C	3.559033	-2.075374	-2.482750
C	4.582438	-2.081431	-0.257566
C	4.305133	1.503004	2.176489
C	5.274447	1.029680	-0.023968
H	3.306865	1.917366	4.056691
H	3.875318	4.551170	-0.220357

C	2.446995	5.082610	-1.755777
H	0.819018	5.444743	-3.114405
H	-1.292620	4.245450	-3.071115
H	-3.228234	3.915070	-1.800973
H	-4.312891	4.563216	0.294113
C	-3.581637	4.034073	2.246657
H	-2.587484	3.426690	4.066819
C	4.425398	-3.141336	-2.846297
C	5.399074	-3.124737	-0.635847
H	4.640980	-1.695475	0.753155
C	5.528448	2.125136	2.544946
C	6.439478	1.658108	0.357920
H	5.184426	0.629779	-1.026650
H	3.137347	5.721107	-2.298899
H	-4.460937	4.415227	2.757191
H	4.353200	-3.549812	-3.851099
C	5.330767	-3.657205	-1.945442
H	6.098707	-3.546202	0.080070
H	5.614122	2.555163	3.539514
C	6.577378	2.200419	1.657757
H	7.260051	1.741959	-0.348672
H	5.986627	-4.473346	-2.233664
H	7.505386	2.684698	1.946514
C	-1.478022	-1.801206	-3.333663
H	-1.286812	-0.826718	-3.791666
H	-0.749224	-2.525776	-3.711691
H	-2.482987	-2.113041	-3.646246
C	1.540592	-4.346639	0.845478
O	0.970256	-3.418301	-1.101064
C	1.892428	-4.171741	-0.457787
H	2.736836	-4.487690	-1.051281
H	2.090784	-4.872894	1.611094
C	-7.276811	-0.790580	-0.092375
H	-7.958275	-0.308361	-0.793306
H	-7.701705	-1.738088	0.254270
H	-7.130986	-0.155915	0.788477
O	0.622426	-3.556196	3.430502
C	1.410072	-2.373309	3.487168
H	0.816124	-1.497509	3.780306
H	2.170416	-2.551434	4.250765
H	1.903796	-2.157782	2.530911
H	-0.851977	-4.566454	2.580393

TS2a-i2

B3LYP-D3 SCF energy: -3112.92543335 a.u.
B3LYP-D3 enthalpy: -3112.075307 a.u.
B3LYP-D3 Gibbs free energy: -3112.222005 a.u.
wB97X-D SCF energy in solution: -3114.13125270 a.u.
wB97X-D enthalpy: -3113.281126 a.u.
wB97X-D Gibbs free energy: -3113.427824 a.u.
Imaginary frequency: -196.6997 cm-1

Cartesian coordinates

ATOM	X	Y	Z
C	-0.944705	-3.774399	-0.055068
C	-2.210340	-3.645114	-0.219279
C	0.203255	-4.406701	-0.581035
C	1.566346	-4.477049	-0.326507
C	1.973111	-2.319376	0.924753
H	2.816705	-1.775610	1.358801
C	0.791216	-2.206404	1.832627
C	-0.475422	-2.194087	1.412461
C	-1.745646	-1.904089	1.944185
H	-2.479391	-2.681360	2.142264
O	-1.819498	-0.831518	2.835474
C	-3.077692	-0.460930	3.236807
O	-4.081779	-0.952909	2.781288
H	1.770989	-1.889280	-0.053614
C	2.397311	-3.801207	0.733007
Rh	-1.815199	-1.530876	-0.179798
C	-3.788883	-1.141080	-0.165328
O	-4.928888	-1.161898	-0.157869
C	1.085729	-2.176241	3.313454
H	0.184215	-2.240760	3.925439
H	1.764788	-2.992637	3.593140
H	1.598158	-1.234906	3.553772
C	-3.490070	-4.378753	-0.336368
H	-4.014719	-4.086214	-1.253379
H	-3.314860	-5.459564	-0.356018
H	-4.155002	-4.143192	0.503431
C	2.122459	-5.291294	-1.354763
O	-0.074894	-5.131236	-1.734726
C	1.088074	-5.662375	-2.164720
H	1.025645	-6.283476	-3.046415
H	3.166069	-5.541575	-1.458973
C	-1.485023	-1.541716	-2.173835
O	-1.341687	-1.621026	-3.303753
P	-0.803414	0.650405	-0.154235
O	0.288257	0.907209	-1.359057
O	0.118857	0.780339	1.205243
N	-1.702819	2.048740	-0.413408
C	1.588884	0.417176	-1.299072
C	0.967492	1.878968	1.364721
C	-1.237612	3.110004	-1.285535
C	-3.102207	2.066054	-0.092081
C	1.936458	-0.555244	-2.262087
C	2.484372	0.910156	-0.363676
C	2.090365	2.008285	0.565163
C	0.616713	2.810734	2.363226
C	-0.377209	4.090852	-0.798094
C	-1.615716	3.082821	-2.643462
C	-4.050566	1.853337	-1.116662
C	-3.502594	2.269981	1.230283
H	1.195348	-0.849307	-2.996170
C	3.199328	-1.095283	-2.249952
C	3.782949	0.291987	-0.295443
C	2.832408	3.237125	0.656415
H	-0.252201	2.607164	2.980248

C	1.375434	3.945618	2.519869
H	-0.120293	4.096901	0.252831
C	0.169438	5.034564	-1.666951
C	-1.038967	4.034702	-3.504287
C	-2.604856	2.144285	-3.168813
C	-3.669822	1.627233	-2.511212
C	-5.410370	1.837843	-0.752900
C	-4.855165	2.246206	1.564490
H	-2.739309	2.451825	1.980373
H	3.480360	-1.846852	-2.982019
C	4.139405	-0.711709	-1.257930
C	4.719656	0.593349	0.732903
C	2.469930	4.206924	1.651893
C	3.888354	3.558079	-0.240218
H	1.123526	4.673270	3.286403
H	0.859946	5.779490	-1.283040
C	-0.153505	4.996750	-3.026109
H	-1.307212	4.020563	-4.557432
H	-2.537075	1.943787	-4.236475
H	-4.386428	1.054765	-3.097904
H	-6.157014	1.664554	-1.523142
C	-5.809893	2.029791	0.565931
H	-5.165280	2.394458	2.594018
C	5.414804	-1.332294	-1.181884
C	5.944921	-0.035273	0.784102
H	4.460616	1.324916	1.488989
C	3.196475	5.425482	1.734779
C	4.560431	4.756528	-0.143459
H	4.153889	2.853089	-1.019192
H	0.279220	5.720005	-3.710908
H	-6.865458	2.008181	0.818560
H	5.666832	-2.089419	-1.919283
C	6.305482	-0.998870	-0.188199
H	6.641547	0.211555	1.580231
H	2.917439	6.146334	2.499010
C	4.221628	5.697501	0.858167
H	5.356180	4.984207	-0.846753
H	7.278146	-1.479466	-0.139001
H	4.765613	6.634877	0.925807
C	-2.997338	0.584720	4.316041
H	-3.956789	1.095836	4.398182
H	-2.774021	0.096284	5.271689
H	-2.192470	1.295363	4.112000
H	2.276317	-4.340123	1.688431
O	3.746143	-3.899261	0.308451
C	4.699994	-3.773476	1.351021
H	4.539744	-4.532395	2.132051
H	5.679407	-3.925995	0.894255
H	4.686710	-2.775259	1.809484

TS2a-i2-p-rot
B3LYP-D3 SCF energy: -3112.91761093 a.u.
B3LYP-D3 enthalpy: -3112.067514 a.u.

B3LYP-D3 Gibbs free energy: -3112.214776 a.u.
wB97X-D SCF energy in solution: -3114.12301405 a.u.
wB97X-D enthalpy: -3113.272917 a.u.
wB97X-D Gibbs free energy: -3113.420179 a.u.
Imaginary frequency: -228.4155 cm-1

Cartesian coordinates

ATOM	X	Y	Z
C	3.802585	-1.505916	-0.456541
C	3.681780	-1.780865	-1.709324
C	4.771804	-1.011829	0.466258
C	4.851542	-0.610866	1.789127
C	2.375084	-0.574014	2.393813
H	1.716005	-0.373256	3.246523
C	1.967512	-1.865435	1.756328
C	1.967560	-2.013905	0.427958
C	1.355122	-2.776498	-0.577937
H	1.905478	-3.465062	-1.211432
O	0.022245	-3.119963	-0.388541
C	-0.610072	-3.660318	-1.494333
O	-0.039152	-3.800804	-2.548849
H	2.256252	0.253504	1.695587
C	3.829565	-0.569037	2.896710
Rh	1.812651	-0.801684	-1.396403
C	0.965085	-1.158676	-3.187842
O	0.530671	-1.306903	-4.230832
C	1.679302	-3.057250	2.640913
H	1.115048	-3.829580	2.112156
H	2.621455	-3.502700	2.989107
H	1.120005	-2.760009	3.535616
C	4.302634	-2.370511	-2.911530
H	4.378799	-1.619666	-3.707516
H	5.306531	-2.742966	-2.680398
H	3.697713	-3.197560	-3.303458
C	6.191523	-0.147528	1.980959
O	5.994657	-0.797459	-0.145593
C	6.833539	-0.292074	0.789782
H	7.841697	-0.091827	0.458613
H	6.589511	0.260666	2.897225
C	-2.043103	-3.979186	-1.197633
C	2.821009	0.843805	-2.007461
O	3.615692	1.492764	-2.509075
P	-0.141840	0.355217	-0.528638
O	-0.844990	-0.031390	0.897880
O	-1.329937	-0.025524	-1.624114
N	-0.058274	2.021687	-0.258089
C	-1.874510	-0.947417	1.102534
C	-2.584047	0.587714	-1.514126
C	-0.736820	2.672064	0.847596
C	0.907679	2.848418	-0.922311
C	-1.609349	-1.912963	2.095765
C	-3.097571	-0.858869	0.455568
C	-3.426258	0.262780	-0.466261
C	-2.908419	1.539990	-2.502415
C	-2.072273	3.047210	0.727855

C	-0.026787	2.889326	2.046847
C	2.066229	3.257591	-0.219724
C	0.682743	3.255734	-2.241666
H	-0.653704	-1.869761	2.597844
C	-2.555129	-2.852349	2.412500
C	-4.054497	-1.913749	0.699186
C	-4.610179	1.066471	-0.293498
H	-2.218677	1.689371	-3.326269
C	-4.088218	2.237012	-2.403557
H	-2.570920	2.927620	-0.223411
C	-2.757591	3.556451	1.830121
C	-0.743396	3.382845	3.152750
C	1.416715	2.695989	2.150595
C	2.329552	2.871665	1.166889
C	2.992240	4.065385	-0.905238
C	1.612612	4.062469	-2.893652
H	-0.231247	2.939752	-2.734514
H	-2.360402	-3.586962	3.189209
C	-3.784446	-2.902362	1.703082
C	-5.250839	-2.048344	-0.059265
C	-4.944360	2.050148	-1.284611
C	-5.433369	0.976497	0.862565
H	-4.359761	2.966364	-3.161538
H	-3.807313	3.816348	1.733623
C	-2.094667	3.702953	3.051956
H	-0.216989	3.539231	4.090840
H	1.803785	2.495332	3.147535
H	3.378768	2.798609	1.447402
H	3.894299	4.381310	-0.388346
C	2.770431	4.466540	-2.219445
H	1.438218	4.373334	-3.919147
C	-4.730159	-3.934143	1.945159
C	-6.138110	-3.074317	0.184820
H	-5.460970	-1.340164	-0.851566
C	-6.104912	2.851707	-1.110536
C	-6.538850	1.785079	1.011780
H	-5.174450	0.272790	1.644237
H	-2.625660	4.083846	3.919337
H	3.500182	5.094288	-2.721667
H	-4.513405	-4.665978	2.719250
C	-5.887407	-4.020538	1.205547
H	-7.038263	-3.158378	-0.417067
H	-6.351275	3.582626	-1.876305
C	-6.890321	2.722884	0.011801
H	-7.145221	1.704235	1.909279
H	-6.600485	-4.817660	1.393120
H	-7.770489	3.345954	0.139108
H	-2.174584	-4.315009	-0.167784
H	-2.633904	-3.065241	-1.319857
H	-2.399732	-4.727915	-1.905963
O	4.081950	0.628217	3.629642
C	3.697479	0.573011	4.995221
H	4.220747	-0.235184	5.527756
H	3.978487	1.532040	5.436118
H	2.613505	0.431963	5.120121

H	3.994072	-1.436730	3.558314

TS2a-i3

B3LYP-D3 SCF energy: -3112.91797047 a.u.
B3LYP-D3 enthalpy: -3112.067726 a.u.
B3LYP-D3 Gibbs free energy: -3112.215418 a.u.
wB97X-D SCF energy in solution: -3114.12404094 a.u.
wB97X-D enthalpy: -3113.273796 a.u.
wB97X-D Gibbs free energy: -3113.421488 a.u.
Imaginary frequency: -164.2294 cm-1

Cartesian coordinates

ATOM	X	Y	Z
C	-2.819403	1.893089	-0.230033
C	-1.538954	1.922906	-0.163037
C	-3.924417	2.434100	-0.922659
C	-5.308424	2.342611	-0.918932
C	-5.663697	0.055870	0.113230
H	-6.508619	-0.605480	0.332227
C	-4.686746	0.000106	1.251136
C	-3.369703	0.153568	1.115298
C	-2.187992	-0.124042	1.825860
H	-1.558648	0.634619	2.282115
O	-2.236334	-1.344902	2.520202
C	-1.172556	-1.695192	3.310156
O	-0.178535	-1.020576	3.417905
H	-5.215125	-0.276051	-0.827074
C	-6.222300	1.497278	-0.071267
Rh	-1.750435	-0.223053	-0.294899
C	-5.275126	-0.138484	2.637759
H	-4.519068	-0.059097	3.421422
H	-6.042210	0.627410	2.812763
H	-5.769866	-1.114287	2.730698
C	-2.489360	-2.058273	-0.353823
O	-3.075908	-3.037182	-0.339651
C	-0.438717	2.913070	-0.081372
H	0.369342	2.648345	-0.765091
H	-0.827101	3.906161	-0.333540
H	-0.014581	2.948422	0.926467
C	-5.769057	3.182342	-1.973404
O	-3.535179	3.280397	-1.954669
C	-4.659382	3.726720	-2.552820
H	-4.516735	4.424992	-3.364595
H	-6.800063	3.329581	-2.253030
C	-1.663865	-0.055225	-2.313880
O	-1.579253	0.096379	-3.440575
P	0.659252	-0.615126	-0.177231
O	1.619231	-0.245356	1.089418
O	1.201427	0.432230	-1.357735
N	1.269402	-2.164046	-0.440318
C	1.979617	1.065447	1.401650
C	2.572658	0.560637	-1.604740
C	2.449763	-2.675838	0.225202

C	0.495626	-3.100251	-1.201659
C	1.489015	1.577737	2.621571
C	2.818478	1.776749	0.560226
C	3.397681	1.142362	-0.657523
C	3.043659	0.069652	-2.840352
C	3.719584	-2.283975	-0.193388
C	2.285568	-3.522807	1.339453
C	-0.135742	-4.180140	-0.545862
C	0.334437	-2.903177	-2.577148
H	0.888923	0.926182	3.243522
C	1.803746	2.866702	2.978862
C	3.077722	3.155151	0.886164
C	4.817144	1.083764	-0.884373
H	2.326071	-0.314151	-3.557493
C	4.391931	0.092841	-3.104404
H	3.821091	-1.664160	-1.072984
C	4.846206	-2.668892	0.531979
C	3.434587	-3.884827	2.066859
C	0.996090	-4.093600	1.716391
C	-0.035417	-4.402702	0.895798
C	-0.929939	-5.042976	-1.326039
C	-0.466797	-3.764883	-3.324593
H	0.848680	-2.071894	-3.048221
H	1.447522	3.274095	3.921345
C	2.572645	3.694663	2.117227
C	3.788780	4.027138	0.015415
C	5.311209	0.558588	-2.126201
C	5.763358	1.477737	0.101528
H	4.771275	-0.277351	-4.052789
H	5.828168	-2.334100	0.211312
C	4.700352	-3.458256	1.675718
H	3.323672	-4.524803	2.938303
H	0.919727	-4.423042	2.750611
H	-0.861288	-4.966668	1.326517
H	-1.430428	-5.875453	-0.838875
C	-1.096560	-4.842507	-2.692756
H	-0.593580	-3.600854	-4.390433
C	2.836086	5.052005	2.444387
C	4.013740	5.342710	0.356614
H	4.151572	3.649896	-0.933339
C	6.713638	0.489453	-2.345480
C	7.116566	1.380482	-0.136550
H	5.409672	1.844921	1.057640
H	5.571663	-3.749048	2.254895
H	-1.717037	-5.524145	-3.266707
H	2.457686	5.441892	3.385954
C	3.544890	5.861459	1.586863
H	4.554786	5.989617	-0.327913
H	7.072897	0.099065	-3.294188
C	7.600684	0.892549	-1.374015
H	7.820195	1.677890	0.635570
H	3.737824	6.898337	1.845623
H	8.670526	0.829267	-1.549176
C	-1.460587	-2.991803	4.016844
H	-0.578142	-3.300796	4.577033

H	-2.304588	-2.858097	4.702159
H	-1.740535	-3.760238	3.291438
O	-7.480389	1.477515	-0.720228
C	-8.582120	1.249921	0.144568
H	-8.523782	0.273242	0.647677
H	-8.659295	2.036632	0.910389
H	-9.477238	1.267460	-0.480359
H	-6.330436	1.966907	0.921215

TS2a-i3-p-rot
B3LYP-D3 SCF energy: -3112.91286099 a.u.
B3LYP-D3 enthalpy: -3112.062645 a.u.
B3LYP-D3 Gibbs free energy: -3112.210281 a.u.
wB97X-D SCF energy in solution: -3114.12123294 a.u.
wB97X-D enthalpy: -3113.271017 a.u.
wB97X-D Gibbs free energy: -3113.418653 a.u.
Imaginary frequency: -253.9804 cm-1

Cartesian coordinates

ATOM	X	Y	Z
C	-2.800882	0.846131	1.334972
C	-1.779415	0.158176	1.704259
C	-3.433356	2.093443	1.559639
C	-4.487338	2.834083	1.046784
C	-4.863000	1.661387	-1.201936
H	-5.538591	1.734006	-2.062133
C	-4.770979	0.235841	-0.742541
C	-3.645473	-0.266276	-0.210858
C	-3.089299	-1.553590	0.037272
H	-2.956974	-1.957661	1.035111
O	-3.402119	-2.528469	-0.915671
C	-3.624139	-3.810151	-0.461536
O	-3.538072	-4.123315	0.701094
H	-3.880002	2.029972	-1.508610
C	-5.447813	2.577030	-0.096910
Rh	-1.535042	-0.116390	-0.424819
C	-6.028225	-0.595655	-0.806812
H	-5.904623	-1.572239	-0.334426
H	-6.865167	-0.078832	-0.318687
H	-6.320194	-0.748118	-1.854543
C	-1.526493	-0.716183	-2.308059
O	-1.525239	-1.149103	-3.363662
C	-1.138533	-0.478771	2.875258
H	-0.056274	-0.330680	2.877174
H	-1.566949	-0.075574	3.799522
H	-1.300426	-1.563303	2.848819
C	-4.522294	4.043360	1.807419
O	-2.814898	2.819143	2.569537
C	-3.502557	3.974958	2.708874
H	-3.158526	4.637302	3.489722
H	-5.226762	4.849306	1.667674
C	-0.898104	1.803546	-0.917030
O	-0.768586	2.874593	-1.284909

P	0.744935	-0.786822	-0.094201
O	1.513436	0.061054	1.088307
O	1.504818	-0.251702	-1.474317
N	1.271913	-2.322416	0.313386
C	1.677183	1.440376	0.901107
C	2.896981	-0.090046	-1.489438
C	2.242787	-2.611549	1.344197
C	0.632285	-3.433609	-0.341601
C	0.880418	2.306990	1.681148
C	2.613487	1.893607	-0.013340
C	3.476428	0.919501	-0.738835
C	3.645802	-0.976505	-2.290635
C	3.581422	-2.263314	1.164801
C	1.797465	-3.193614	2.549957
C	-0.207567	-4.290464	0.399082
C	0.825169	-3.626324	-1.712705
H	0.185337	1.893807	2.400195
C	0.994719	3.664251	1.503273
C	2.688389	3.309057	-0.253977
C	4.911132	0.967531	-0.668397
H	3.124420	-1.704758	-2.901033
C	5.017513	-0.892446	-2.285535
H	3.897788	-1.831724	0.223752
C	4.496374	-2.448761	2.199674
C	2.739467	-3.357848	3.583625
C	0.426729	-3.660015	2.758333
C	-0.440038	-4.135669	1.834378
C	-0.833635	-5.346980	-0.289346
C	0.187848	-4.675012	-2.374473
H	1.480866	-2.951770	-2.250173
H	0.387552	4.342170	2.096944
C	1.873845	4.198879	0.525610
C	3.515146	3.869277	-1.266824
C	5.683620	0.050514	-1.458400
C	5.604807	1.863946	0.190843
H	5.608171	-1.565944	-2.900258
H	5.532941	-2.158659	2.057018
C	4.070948	-2.989804	3.415982
H	2.413443	-3.796363	4.523177
H	0.116557	-3.722224	3.800296
H	-1.388532	-4.534650	2.186442
H	-1.488180	-6.011824	0.265600
C	-0.636163	-5.543678	-1.653575
H	0.341670	-4.816867	-3.439880
C	1.949965	5.598364	0.291707
C	3.555376	5.230091	-1.475308
H	4.115104	3.212215	-1.885212
C	7.102359	0.093037	-1.387455
C	6.981018	1.869532	0.245289
H	5.038320	2.542926	0.817413
H	4.776652	-3.128848	4.229496
H	-1.123448	-6.375050	-2.154584
H	1.336215	6.260753	0.896875
C	2.774997	6.107151	-0.684320
H	4.190678	5.634805	-2.257735

H	7.674544	-0.600532	-1.998210
C	7.740946	0.984337	-0.556543
H	7.489182	2.558160	0.914070
H	2.824006	7.178179	-0.856713
H	8.825481	1.005863	-0.506648
C	-4.007667	-4.696902	-1.612728
H	-3.242292	-4.645305	-2.391803
H	-4.120001	-5.721916	-1.259146
H	-4.951554	-4.349340	-2.046460
O	-5.943195	3.801775	-0.615432
C	-5.024508	4.579651	-1.377103
H	-5.515163	5.538999	-1.554008
H	-4.082532	4.754235	-0.838322
H	-4.800821	4.115736	-2.347018
H	-6.344469	2.093467	0.309318

TS2b

B3LYP-D3 SCF energy: -3112.93122800 a.u.
B3LYP-D3 enthalpy: -3112.080724 a.u.
B3LYP-D3 Gibbs free energy: -3112.226600 a.u.
wB97X-D SCF energy in solution: -3114.13592776 a.u.
wB97X-D enthalpy: -3113.285424 a.u.
wB97X-D Gibbs free energy: -3113.431300 a.u.
Imaginary frequency: -175.1838 cm-1

Cartesian coordinates

ATOM	X	Y	Z
C	-1.081488	-1.179146	1.918780
C	-1.878299	-0.205275	2.127999
C	0.220513	-1.661817	2.225512
C	1.095593	-2.657351	1.826462
C	-0.051687	-3.567102	-0.279050
H	0.123095	-4.332632	-1.042038
C	-1.486373	-3.632168	0.166830
C	-2.180603	-2.544076	0.493826
C	-3.494834	-2.098985	0.668910
H	-3.904945	-1.825653	1.636647
O	-4.434031	-2.568953	-0.246637
C	-5.694530	-2.010789	-0.172868
O	-5.961210	-1.122405	0.598190
H	0.164229	-2.595598	-0.726082
C	0.959917	-3.806574	0.851143
Rh	-2.248225	-0.401305	0.026781
C	-3.770424	0.928796	0.157585
O	-4.683439	1.585871	0.335295
C	-2.144433	-4.987170	0.287780
H	-3.200996	-4.908335	0.553566
H	-1.615899	-5.585261	1.035009
H	-2.063814	-5.516701	-0.670239
C	-2.408530	-0.871094	-1.877510
O	-2.552656	-1.148736	-2.977308
P	-0.352108	1.090512	-0.426020
O	0.547702	0.878129	-1.815228

O	0.749371	0.795237	0.738329
N	-0.598434	2.746696	-0.466005
C	1.265464	-0.308053	-1.975951
C	2.114752	1.082444	0.675266
C	-1.879394	3.288165	-0.819623
C	0.468025	3.689342	-0.222877
C	0.823537	-1.189968	-2.986742
C	2.388200	-0.552808	-1.198900
C	2.922647	0.486917	-0.276072
C	2.599731	1.950594	1.673447
C	-2.414795	3.035804	-2.086867
C	-2.591224	4.046185	0.134223
C	0.404503	4.488149	0.938173
C	1.560394	3.761352	-1.090097
H	-0.017090	-0.893559	-3.603247
C	1.473347	-2.384768	-3.175284
C	3.010611	-1.846084	-1.322371
C	4.292229	0.933007	-0.350721
H	1.900224	2.335009	2.405801
C	3.924070	2.304197	1.667275
H	-1.823766	2.472182	-2.803436
C	-3.683931	3.509574	-2.418051
C	-3.867715	4.515222	-0.226853
C	-2.060583	4.345454	1.464515
C	-0.765061	4.522683	1.815404
C	1.507393	5.313810	1.226028
C	2.636788	4.592855	-0.784522
H	1.573075	3.138588	-1.976479
H	1.149643	-3.067540	-3.955914
C	2.546883	-2.762766	-2.325194
C	4.051121	-2.276938	-0.452198
C	4.793041	1.837664	0.644485
C	5.168535	0.546644	-1.401906
H	4.313440	2.978809	2.424572
H	-4.100344	3.304277	-3.399577
C	-4.409580	4.252892	-1.482292
H	-4.438001	5.091349	0.496719
H	-2.812559	4.545402	2.226026
H	-0.569837	4.847133	2.836015
H	1.480896	5.930604	2.120627
C	2.611712	5.364152	0.380555
H	3.496751	4.625089	-1.446067
C	3.148798	-4.046329	-2.429893
C	4.605192	-3.532872	-0.572747
H	4.395701	-1.609020	0.327201
C	6.142907	2.274135	0.576156
C	6.468771	1.001184	-1.447977
H	4.802427	-0.104399	-2.186985
H	-5.397765	4.627005	-1.731698
H	3.450815	6.008317	0.625826
H	2.790306	-4.726946	-3.197880
C	4.156468	-4.428201	-1.573679
H	5.394225	-3.839950	0.108023
H	6.508435	2.952138	1.343354
C	6.969264	1.863736	-0.444970

H	7.114027	0.698276	-2.267549
H	4.605780	-5.412910	-1.660807
H	7.997953	2.208589	-0.490666
C	-2.432985	0.760320	3.100724
H	-2.197105	1.783423	2.781780
H	-3.523786	0.677703	3.170772
H	-1.999653	0.586640	4.091398
C	2.318460	-2.401325	2.527194
O	0.862552	-0.825008	3.124286
C	2.116034	-1.298388	3.299748
H	2.735319	-0.750219	3.993687
H	3.231094	-2.971230	2.433054
C	-6.613336	-2.659506	-1.169433
H	-7.605389	-2.214909	-1.089548
H	-6.220281	-2.522395	-2.182611
H	-6.667273	-3.737022	-0.983254
O	0.605242	-5.027496	1.493447
C	1.627406	-5.614072	2.283021
H	1.834195	-5.030688	3.190491
H	1.267827	-6.604093	2.572515
H	2.561290	-5.723410	1.709525
H	1.945256	-3.926917	0.377316

TS2b-n-rot

B3LYP-D3 SCF energy: -3112.92808012 a.u.
B3LYP-D3 enthalpy: -3112.077570 a.u.
B3LYP-D3 Gibbs free energy: -3112.224857 a.u.
wB97X-D SCF energy in solution: -3114.13341324 a.u.
wB97X-D enthalpy: -3113.282903 a.u.
wB97X-D Gibbs free energy: -3113.430190 a.u.
Imaginary frequency: -190.9257 cm-1

Cartesian coordinates

ATOM	X	Y	Z
C	-1.280885	-1.590677	1.792736
C	-2.138346	-0.697797	2.109262
C	0.004584	-2.091599	2.128881
C	0.955744	-2.963589	1.625678
C	0.062602	-3.440487	-0.726408
H	0.334887	-4.037447	-1.602721
C	-1.410436	-3.589569	-0.465083
C	-2.157190	-2.590382	0.005446
C	-3.502309	-2.209326	0.128784
H	-4.018263	-2.166328	1.083596
O	-4.328881	-2.495588	-0.956933
C	-5.590353	-1.938383	-0.924644
O	-5.943750	-1.195978	-0.041472
H	0.307257	-2.400340	-0.943626
C	0.952166	-3.900612	0.437457
Rh	-2.272453	-0.404320	-0.007132
C	-3.789100	0.896371	0.274964
O	-4.704556	1.533621	0.506664
C	-2.048575	-4.938155	-0.703821

H	-3.127787	-4.918343	-0.536777
H	-1.584461	-5.682628	-0.050930
H	-1.861648	-5.250993	-1.739250
C	-2.254553	-0.455562	-1.978306
O	-2.287768	-0.510478	-3.119330
P	-0.321328	1.098941	-0.087886
O	0.630290	1.064693	-1.435133
O	0.671869	0.496669	1.081078
N	-0.417708	2.772924	0.035346
C	1.433709	-0.037145	-1.714022
C	1.999500	0.899629	1.197781
C	0.390099	3.675341	-0.756193
C	-1.506149	3.350477	0.771447
C	1.128093	-0.755947	-2.890959
C	2.508208	-0.353990	-0.896511
C	2.912391	0.544755	0.221765
C	2.341816	1.643043	2.346012
C	1.721629	3.908766	-0.416225
C	-0.178600	4.253220	-1.909364
C	-2.586133	3.931082	0.071947
C	-1.498126	3.296077	2.168296
H	0.321335	-0.399648	-3.521071
C	1.857685	-1.874816	-3.208964
C	3.205749	-1.583453	-1.164602
C	4.238995	1.093744	0.316907
H	1.576491	1.828605	3.091826
C	3.628049	2.105518	2.485501
H	2.126763	3.463770	0.483651
C	2.527197	4.687386	-1.245916
C	0.658428	5.022693	-2.738897
C	-1.595181	4.121783	-2.245457
C	-2.636206	3.999285	-1.388433
C	-3.659306	4.435765	0.830703
C	-2.573288	3.804530	2.897046
H	-0.641741	2.854372	2.668477
H	1.636856	-2.433488	-4.114216
C	2.873439	-2.345912	-2.334541
C	4.189289	-2.106860	-0.279287
C	4.594322	1.874263	1.469315
C	5.210871	0.932429	-0.708593
H	3.912740	2.678227	3.363919
H	3.568822	4.848293	-0.984737
C	1.995386	5.236394	-2.416036
H	0.240607	5.466855	-3.638566
H	-1.836634	4.259537	-3.297875
H	-3.637560	4.052311	-1.812234
H	-4.506659	4.875121	0.311532
C	-3.656930	4.375547	2.220960
H	-2.564296	3.759643	3.982107
C	3.542627	-3.576005	-2.578125
C	4.807704	-3.311129	-0.536856
H	4.436643	-1.554250	0.619046
C	5.903190	2.419080	1.566007
C	6.466399	1.486711	-0.589348
H	4.952092	0.376700	-1.602120

H	2.620586	5.834352	-3.072399
H	-4.498234	4.774484	2.779501
H	3.284579	-4.140075	-3.470765
C	4.487806	-4.054145	-1.699075
H	5.549355	-3.694549	0.158307
H	6.160297	3.000492	2.447743
C	6.823875	2.228860	0.561373
H	7.188429	1.357275	-1.390368
H	4.987553	-4.998587	-1.892449
H	7.819554	2.654406	0.642985
C	-2.905499	-0.067156	3.205528
H	-2.818722	1.023240	3.153862
H	-3.971869	-0.312921	3.128327
H	-2.533363	-0.415684	4.174903
C	2.103921	-2.812641	2.466352
O	0.532554	-1.427100	3.224774
C	1.787675	-1.888299	3.415989
H	2.320767	-1.473908	4.258598
H	3.046497	-3.326330	2.345245
C	-6.391173	-2.382283	-2.116349
H	-7.386403	-1.940168	-2.068536
H	-5.887226	-2.075099	-3.039107
H	-6.464721	-3.474506	-2.132548
O	0.557682	-5.223125	0.786809
C	1.499783	-5.945635	1.563498
H	1.597942	-5.536914	2.578467
H	1.129183	-6.971087	1.628242
H	2.491487	-5.950233	1.084388
H	1.986079	-3.923232	0.059996

TS2b-p-rot

B3LYP-D3 SCF energy: -3112.91998586 a.u.
B3LYP-D3 enthalpy: -3112.069980 a.u.
B3LYP-D3 Gibbs free energy: -3112.217927 a.u.
wB97X-D SCF energy in solution: -3114.12910124 a.u.
wB97X-D enthalpy: -3113.279095 a.u.
wB97X-D Gibbs free energy: -3113.427042 a.u.
Imaginary frequency: -219.4108 cm-1

Cartesian coordinates

ATOM	X	Y	Z
C	-1.682256	2.032777	0.027053
C	-0.984231	2.099192	-1.041050
C	-1.894975	2.674028	1.290044
C	-2.504773	2.408094	2.496661
C	-3.580293	0.152822	1.920806
H	-4.218054	-0.617194	2.370506
C	-4.263116	0.737269	0.714412
C	-3.570061	1.006070	-0.392281
C	-3.654849	1.157077	-1.778005
H	-2.647693	-0.337170	1.632085
C	-3.252800	1.203340	3.003517
Rh	-1.748313	0.121319	-1.237634

C	-1.027661	-0.048943	-3.116234
O	-0.573715	-0.086792	-4.159805
C	-5.718169	1.114069	0.822855
H	-6.107079	1.532289	-0.108328
H	-5.841866	1.839130	1.635449
H	-6.312273	0.229446	1.086550
C	-2.748236	-1.602617	-1.179955
O	-3.490203	-2.463955	-1.288362
P	0.330852	-0.701467	-0.244004
O	1.124295	0.176883	0.885932
O	1.348468	-0.626068	-1.552697
N	0.456937	-2.187771	0.542578
C	1.900833	1.300621	0.598660
C	2.703219	-0.944559	-1.406453
C	1.308076	-2.385723	1.698561
C	-0.484639	-3.230903	0.260105
C	1.496320	2.494556	1.228536
C	3.029785	1.213536	-0.199662
C	3.531142	-0.101337	-0.689347
C	3.145523	-2.140685	-2.007803
C	2.675321	-2.596246	1.533076
C	0.738729	-2.293919	2.985124
C	-1.524339	-3.497475	1.180945
C	-0.371811	-3.958192	-0.929087
H	0.650889	2.459312	1.902407
C	2.180056	3.656787	0.977485
C	3.699651	2.444008	-0.538469
C	4.866936	-0.559146	-0.411820
H	2.445669	-2.714846	-2.605538
C	4.447222	-2.541012	-1.824239
H	3.079763	-2.697008	0.535364
C	3.514195	-2.650871	2.645216
C	1.606853	-2.335040	4.092323
C	-0.702412	-2.228287	3.209630
C	-1.676658	-2.770592	2.440554
C	-2.447035	-4.506673	0.847087
C	-1.296093	-4.956088	-1.232299
H	0.451836	-3.733946	-1.599683
H	1.874996	4.582343	1.457480
C	3.269146	3.673161	0.065734
C	4.760798	2.499105	-1.484083
C	5.323856	-1.789122	-0.996019
C	5.743646	0.131296	0.469590
H	4.809554	-3.456228	-2.284020
H	4.581941	-2.787850	2.503171
C	2.978655	-2.503525	3.927728
H	1.187280	-2.251777	5.091554
H	-1.005856	-1.811254	4.168635
H	-2.689126	-2.754429	2.840001
H	-3.258483	-4.723723	1.536717
C	-2.335811	-5.229092	-0.337354
H	-1.207334	-5.515711	-2.158393
C	3.933026	4.884291	-0.266191
C	5.374239	3.693211	-1.793993
H	5.082146	1.588100	-1.975038

C	6.638233	-2.249537	-0.712965
C	7.005100	-0.353025	0.737359
H	5.405194	1.043361	0.946443
H	3.627843	-2.530453	4.797790
H	-3.060238	-6.004984	-0.565486
H	3.601486	5.804971	0.207299
C	4.966900	4.898049	-1.174169
H	6.176796	3.710014	-2.525622
H	6.974311	-3.176280	-1.170847
C	7.464957	-1.548089	0.134201
H	7.653179	0.186780	1.421697
H	5.464874	5.830228	-1.423702
H	8.465255	-1.912167	0.348795
C	-0.024737	2.852663	-1.875935
H	0.897259	2.276974	-2.010075
H	-0.441046	3.054640	-2.869815
H	0.222133	3.800455	-1.387725
C	-2.277354	3.564243	3.316491
O	-1.308975	3.922277	1.335615
C	-1.559662	4.442026	2.569226
H	-1.177118	5.436151	2.744586
H	-2.640789	3.702169	4.323815
O	-4.416838	1.705233	3.653255
H	-2.619137	0.696028	3.753211
C	-4.975563	0.836594	4.626755
H	-4.233950	0.562936	5.392650
H	-5.792870	1.385199	5.099528
H	-5.381526	-0.085280	4.184904
O	-4.627761	0.404537	-2.427265
C	-4.494832	0.281370	-3.796009
O	-3.556855	0.747319	-4.394727
C	-5.643358	-0.502207	-4.365137
H	-5.524580	-0.585355	-5.445494
H	-5.672450	-1.498597	-3.911076
H	-6.590057	-0.006407	-4.127387
H	-3.345636	2.057631	-2.299504

TS2b-i2

B3LYP-D3 SCF energy: -3112.92360606 a.u.
B3LYP-D3 enthalpy: -3112.073153 a.u.
B3LYP-D3 Gibbs free energy: -3112.218479 a.u.
wB97X-D SCF energy in solution: -3114.12734443 a.u.
wB97X-D enthalpy: -3113.276891 a.u.
wB97X-D Gibbs free energy: -3113.422217 a.u.
Imaginary frequency: -216.5455 cm-1

Cartesian coordinates

ATOM	X	Y	Z
C	-3.799001	0.967804	-0.305754
C	-4.326202	-0.193329	-0.153610
C	-3.924441	2.120156	-1.132532
C	-3.289518	3.331599	-1.350677
C	-1.101970	2.980543	-0.080733

H	-0.198205	3.533795	0.196423
C	-1.615838	2.263415	1.138667
C	-2.182467	1.052615	1.077897
C	-2.452711	-0.038225	1.932881
H	-3.452763	-0.286279	2.279659
O	-1.441653	-0.338280	2.851933
C	-1.462391	-1.585877	3.424831
O	-2.317990	-2.402001	3.180631
H	-0.826010	2.261883	-0.856963
C	-2.109061	3.982964	-0.664047
H	-1.583565	4.570605	-1.436030
Rh	-2.267871	-0.804998	-0.071483
C	-1.600478	2.984004	2.468073
H	-1.637147	2.284906	3.307171
H	-2.466194	3.652533	2.515199
H	-0.706646	3.605237	2.556959
C	-2.191067	-0.933347	-2.091998
O	-2.224524	-0.935001	-3.232475
C	-5.584429	-0.963327	-0.064041
H	-5.608000	-1.747733	-0.829832
H	-5.679206	-1.460219	0.909053
H	-6.445323	-0.301798	-0.208237
C	-3.960993	3.928225	-2.464142
O	-4.930837	1.960312	-2.072223
C	-4.945271	3.067216	-2.847373
H	-3.719217	4.879431	-2.917415
H	-5.704379	3.097324	-3.614943
P	0.143114	-0.928373	-0.086063
O	1.113398	-0.441072	1.136012
O	0.500560	0.151361	-1.296825
N	0.890344	-2.409483	-0.372676
C	1.569272	0.860077	1.349695
C	1.829670	0.296791	-1.710504
C	2.145289	-2.792411	0.240304
C	0.131268	-3.474100	-0.966897
C	1.310907	1.381986	2.634532
C	2.309867	1.543807	0.397243
C	2.747686	0.900786	-0.872566
C	2.155694	-0.207441	-2.986187
C	3.352855	-2.394683	-0.328029
C	2.113704	-3.528237	1.443033
C	-0.420680	-4.473716	-0.137080
C	-0.085052	-3.479185	-2.346987
H	0.758003	0.770712	3.334523
C	1.748300	2.641326	2.956689
C	2.651050	2.915848	0.682586
C	4.133348	0.856061	-1.263850
H	1.361677	-0.611516	-3.605127
C	3.461810	-0.163731	-3.410585
H	3.345161	-1.870046	-1.273302
C	4.554125	-2.655199	0.330165
C	3.336874	-3.763969	2.098543
C	0.881188	-4.087178	1.993538
C	-0.209416	-4.507139	1.309918
C	-1.226667	-5.454377	-0.747534

C	-0.883382	-4.463330	-2.927735
H	0.380383	-2.706362	-2.950318
H	1.568378	3.044380	3.949580
C	2.385627	3.456677	1.984837
C	3.220898	3.779209	-0.294160
C	4.482971	0.327969	-2.552920
C	5.186600	1.257679	-0.397088
H	3.732271	-0.539728	-4.393319
H	5.488375	-2.315976	-0.106970
C	4.542323	-3.327174	1.555740
H	3.332479	-4.316685	3.034507
H	0.899668	-4.294646	3.061552
H	-0.992000	-5.002554	1.880171
H	-1.676793	-6.222990	-0.125404
C	-1.457286	-5.451532	-2.119959
H	-1.057420	-4.459500	-3.999443
C	2.737974	4.803216	2.269734
C	3.533719	5.086579	0.008479
H	3.400567	3.403431	-1.294450
C	5.850032	0.271948	-2.937169
C	6.503304	1.170096	-0.792768
H	4.947421	1.621334	0.594884
H	5.472649	-3.521663	2.081078
H	-2.080787	-6.222206	-2.563187
H	2.539734	5.192428	3.265176
C	3.303613	5.604622	1.304819
H	3.959632	5.727227	-0.758307
H	6.097952	-0.119791	-3.920269
C	6.842403	0.685647	-2.078587
H	7.290394	1.472753	-0.108238
H	3.564088	6.634320	1.530734
H	7.884481	0.631227	-2.379013
C	-2.855477	-2.667160	0.397935
O	-3.431898	-3.619076	0.648782
C	-0.276714	-1.759316	4.332835
H	-0.223327	-0.938429	5.054597
H	-0.356332	-2.712565	4.855493
H	0.637122	-1.739457	3.729037
O	-2.498638	4.852808	0.391309
C	-3.174119	6.033542	-0.012785
H	-4.167027	5.819174	-0.430814
H	-3.290607	6.645984	0.884041
H	-2.588166	6.595342	-0.757087

TS2b-i2-p-rot

B3LYP-D3 SCF energy: -3112.91431697 a.u.
B3LYP-D3 enthalpy: -3112.063994 a.u.
B3LYP-D3 Gibbs free energy: -3112.211211 a.u.
wB97X-D SCF energy in solution: -3114.11946110 a.u.
wB97X-D enthalpy: -3113.269138 a.u.
wB97X-D Gibbs free energy: -3113.416355 a.u.
Imaginary frequency: -227.5821 cm-1

Cartesian coordinates

ATOM	X	Y	Z
C	-3.601219	-1.967447	-0.012385
C	-2.706820	-2.879302	0.108397
C	-4.732199	-1.656781	-0.802928
C	-5.667685	-0.643679	-0.917350
C	-4.467367	1.314875	0.174178
C	-3.703277	0.690193	1.311613
C	-2.824542	-0.311022	1.171797
C	-1.906664	-0.993891	2.009070
H	-2.138761	-1.969277	2.430161
O	-1.109781	-0.208525	2.841569
C	-0.098273	-0.875561	3.492807
O	0.111409	-2.053409	3.332196
H	-3.874113	1.314986	-0.744422
C	-5.818735	0.624397	-0.114367
Rh	-1.278987	-1.291022	-0.008397
C	-4.020420	1.202963	2.697826
H	-3.436116	0.700821	3.470833
H	-5.087896	1.073572	2.897833
H	-3.798891	2.275608	2.740682
C	-1.377149	-1.214075	-2.046110
O	-1.530518	-1.163755	-3.176206
C	-2.497119	-4.332263	0.293361
H	-1.839765	-4.725521	-0.490726
H	-2.012216	-4.538733	1.255214
H	-3.453118	-4.865169	0.257549
C	-6.494072	-0.990370	-2.026997
O	-4.949875	-2.586940	-1.810525
C	-6.022802	-2.174474	-2.517494
H	-7.330126	-0.418177	-2.404836
H	-6.335571	-2.821215	-3.324112
P	0.405477	0.446481	-0.105194
O	1.428235	0.119591	-1.351064
O	1.346198	0.175044	1.233056
N	0.282785	2.095723	-0.398075
C	2.241002	-1.014373	-1.309081
C	2.661450	0.653445	1.273618
C	1.086754	2.767666	-1.401012
C	-0.750699	2.885688	0.210444
C	1.969474	-2.027920	-2.253403
C	3.283685	-1.088894	-0.400274
C	3.620952	0.084592	0.454226
C	2.940167	1.706879	2.169168
C	2.424921	3.066017	-1.149726
C	0.501769	3.049622	-2.651375
C	-1.808890	3.368467	-0.588284
C	-0.681210	3.189639	1.572277
H	1.184129	-1.865300	-2.982588
C	2.714489	-3.181441	-2.233716
C	4.011851	-2.325940	-0.317684
C	4.923923	0.693569	0.425583
H	2.148416	2.074999	2.810396
C	4.204695	2.243874	2.205388
H	2.843393	2.857093	-0.174091

C	3.221337	3.600728	-2.160994
C	1.329710	3.571050	-3.662710
C	-0.928788	2.888524	-2.906273
C	-1.931746	3.054897	-2.013163
C	-2.774481	4.184494	0.031530
C	-1.653107	3.993757	2.163914
H	0.147304	2.799843	2.152203
H	2.525083	-3.967163	-2.959955
C	3.723038	-3.376453	-1.252988
C	4.993409	-2.567779	0.682287
C	5.213760	1.778041	1.321535
C	5.933759	0.296121	-0.493921
H	4.435993	3.053157	2.892391
H	4.268486	3.809712	-1.963865
C	2.674609	3.839717	-3.425028
H	0.898208	3.782397	-4.637598
H	-1.207782	2.754948	-3.949836
H	-2.949384	3.054491	-2.400597
H	-3.589904	4.577808	-0.570126
C	-2.696434	4.503494	1.384093
H	-1.592906	4.228712	3.222285
C	4.446891	-4.596362	-1.175832
C	5.666615	-3.768131	0.737698
H	5.204276	-1.797761	1.415176
C	6.499119	2.383713	1.292329
C	7.164061	0.915133	-0.506171
H	5.724692	-0.497605	-1.200983
H	-3.448214	5.146716	1.832079
H	4.224792	-5.379349	-1.896330
C	5.401304	-4.790634	-0.204181
H	6.406442	-3.933557	1.515448
H	6.706792	3.196395	1.983632
C	7.457746	1.961667	0.400590
H	7.916505	0.599036	-1.222810
H	5.945434	-5.728840	-0.150912
H	8.435271	2.434212	0.382836
C	0.177267	-2.614185	0.394642
O	0.877575	-3.467773	0.677796
H	3.293896	4.242316	-4.221040
H	-4.676745	2.355760	0.432588
C	0.659616	0.040374	4.413376
H	0.328043	1.075499	4.317799
H	0.516471	-0.295475	5.445564
H	1.725284	-0.037428	4.183198
H	-6.417519	1.315551	-0.731833
O	-6.478750	0.418076	1.124484
C	-7.841429	0.033740	1.011689
H	-7.951796	-0.950755	0.536812
H	-8.235855	-0.013213	2.028772
H	-8.418881	0.773008	0.435100

TS2b-i3
B3LYP-D3 SCF energy: -3112.91917827 a.u.

B3LYP-D3 enthalpy: -3112.068763 a.u.
B3LYP-D3 Gibbs free energy: -3112.215642 a.u.
wB97X-D SCF energy in solution: -3114.12715618 a.u.
wB97X-D enthalpy: -3113.276741 a.u.
wB97X-D Gibbs free energy: -3113.423620 a.u.
Imaginary frequency: -203.4626 cm-1

Cartesian coordinates

ATOM	X	Y	Z
C	-3.418742	0.000044	1.036778
C	-2.323698	-0.475890	1.503312
C	-4.824436	-0.210454	1.066375
C	-5.968033	0.263702	0.448167
C	-5.016636	1.540148	-1.564309
H	-5.388009	2.138740	-2.403029
C	-3.824008	2.240363	-0.964775
C	-2.810792	1.596623	-0.387036
C	-1.464502	1.771051	-0.010331
H	-1.180496	1.881352	1.031221
O	-0.609146	2.451016	-0.901662
C	-0.223348	3.716503	-0.521199
O	-0.664446	4.261587	0.461762
H	-4.739410	0.553545	-1.949805
C	-6.180566	1.364189	-0.568501
H	-7.069931	1.074434	-1.153827
Rh	-1.726726	-0.294393	-0.538116
C	-1.376363	0.101521	-2.457571
O	-1.165966	0.344340	-3.551315
C	-3.808523	3.752460	-1.000841
H	-2.990247	4.171276	-0.412334
H	-4.760616	4.134902	-0.624895
H	-3.705521	4.085255	-2.043394
C	-2.588559	-2.085741	-0.961573
O	-3.235183	-3.005245	-1.152000
P	0.584456	-0.850096	-0.133301
O	1.348859	-0.281496	1.203766
O	1.363705	-0.062086	-1.368424
N	1.094626	-2.450900	-0.004112
C	1.765747	1.049409	1.309337
C	2.760673	-0.041051	-1.424538
C	2.144919	-2.901107	0.887034
C	0.362198	-3.459716	-0.716477
C	1.144311	1.821303	2.314098
C	2.777529	1.540298	0.501651
C	3.480140	0.660384	-0.473050
C	3.367967	-0.764516	-2.472164
C	3.479989	-2.625942	0.597948
C	1.789419	-3.573451	2.074819
C	-0.424291	-4.391244	-0.001882
C	0.396745	-3.475521	-2.114804
H	0.399440	1.355618	2.949353
C	1.491960	3.139544	2.468010
C	3.098421	2.941202	0.608523
C	4.908478	0.491132	-0.458990
H	2.736433	-1.235328	-3.217715

C	4.738395	-0.854229	-2.521064
H	3.733306	-2.153364	-0.339660
C	4.478928	-2.940634	1.518164
C	2.812585	-3.867852	2.996004
C	0.434486	-4.041145	2.353155
C	-0.509709	-4.414312	1.457324
C	-1.170143	-5.323765	-0.748145
C	-0.356432	-4.405895	-2.829825
H	1.022904	-2.753875	-2.629406
H	1.008999	3.747572	3.226666
C	2.446942	3.739854	1.606632
C	4.006008	3.587773	-0.276444
C	5.537416	-0.270424	-1.501285
C	5.726632	1.005635	0.584224
H	5.223149	-1.404557	-3.322593
H	5.511519	-2.693797	1.290187
C	4.140739	-3.548394	2.729645
H	2.552744	-4.373059	3.922608
H	0.214497	-4.224250	3.403573
H	-1.414006	-4.872266	1.854708
H	-1.785950	-6.044512	-0.216932
C	-1.139637	-5.335762	-2.139460
H	-0.328891	-4.407185	-3.915277
C	2.738264	5.127269	1.693545
C	4.262845	4.937095	-0.169307
H	4.490665	3.012025	-1.055589
C	6.947131	-0.449330	-1.480299
C	7.088345	0.798135	0.583915
H	5.265475	1.555587	1.396150
H	4.910434	-3.784378	3.458348
H	-1.727116	-6.067136	-2.686064
H	2.229795	5.717249	2.451129
C	3.629425	5.717953	0.826752
H	4.953770	5.408036	-0.862581
H	7.410915	-1.019711	-2.280925
C	7.709836	0.073538	-0.461391
H	7.691671	1.191629	1.396834
H	3.841953	6.780391	0.898808
H	8.785501	-0.074845	-0.451165
C	-1.647114	-1.005178	2.704878
H	-1.457974	-2.076876	2.575514
H	-0.669912	-0.539109	2.858498
H	-2.270962	-0.851923	3.591797
C	-7.041810	-0.541540	0.943858
O	-5.161773	-1.271479	1.892828
C	-6.501308	-1.437421	1.816772
H	-8.082984	-0.454410	0.665782
H	-6.917632	-2.217910	2.436287
C	0.760084	4.288298	-1.503998
H	1.415661	3.510064	-1.898084
H	0.208045	4.732749	-2.340731
H	1.345404	5.064902	-1.010412
O	-6.415853	2.627846	0.035535
C	-7.639786	2.741545	0.746254
H	-7.656026	2.106004	1.641738

H	-7.724958	3.787163	1.049209
H	-8.498496	2.480818	0.108064

TS2b-i3-p-rot

B3LYP-D3 SCF energy: -3112.91017202 a.u.
B3LYP-D3 enthalpy: -3112.059770 a.u.
B3LYP-D3 Gibbs free energy: -3112.208768 a.u.
wB97X-D SCF energy in solution: -3114.11868026 a.u.
wB97X-D enthalpy: -3113.268278 a.u.
wB97X-D Gibbs free energy: -3113.417276 a.u.
Imaginary frequency: -187.2671 cm-1

Cartesian coordinates

ATOM	X	Y	Z
C	-2.927433	-1.143310	1.336131
C	-1.912291	-0.527340	1.814430
C	-3.631303	-2.370773	1.411798
C	-4.699742	-3.003076	0.798254
C	-4.978969	-1.659014	-1.360032
H	-5.646214	-1.638094	-2.228541
C	-4.730808	-0.240694	-0.914279
C	-3.601362	0.166682	-0.335610
C	-2.870495	1.356162	-0.106860
H	-2.819344	1.852273	0.857761
O	-2.847387	2.246006	-1.211105
C	-3.602517	3.383653	-1.101795
O	-4.183759	3.698677	-0.090944
H	-4.046135	-2.145339	-1.664052
C	-5.651601	-2.528421	-0.276936
H	-6.034413	-3.436322	-0.773185
Rh	-1.468818	-0.243939	-0.280388
C	-1.409348	0.028427	-2.235763
O	-1.411179	0.224556	-3.360189
C	-5.852857	0.753655	-1.122054
H	-5.674370	1.696391	-0.601499
H	-6.792875	0.322211	-0.769991
H	-5.961580	0.955649	-2.197365
C	-0.633477	-2.123451	-0.332883
O	-0.273015	-3.201071	-0.256060
P	0.753086	0.700670	0.079304
O	1.787575	0.427514	-1.171802
O	1.355618	-0.299045	1.267346
N	1.194747	2.293058	0.370371
C	2.138487	-0.886872	-1.485736
C	2.731600	-0.366763	1.506229
C	2.322373	2.940031	-0.264265
C	0.289120	3.122981	1.117684
C	1.639438	-1.408625	-2.699005
C	2.969629	-1.603754	-0.640245
C	3.558879	-0.951549	0.563487
C	3.203751	0.166512	2.723192
C	3.621936	2.654138	0.151850
C	2.081128	3.812479	-1.345420

C	-0.511558	4.067993	0.440676
C	0.187557	2.953831	2.499424
H	1.060202	-0.760541	-3.346397
C	1.925353	-2.708342	-3.040603
C	3.211035	-2.986475	-0.952991
C	4.978048	-0.876738	0.779248
H	2.488339	0.562629	3.435882
C	4.555193	0.173722	2.973096
H	3.777304	1.995567	0.995961
C	4.710116	3.194166	-0.531214
C	3.197409	4.334372	-2.027433
C	0.743930	4.235769	-1.751477
C	-0.363721	4.357260	-0.982368
C	-1.471206	4.769000	1.196200
C	-0.758094	3.674694	3.230034
H	0.851543	2.247967	2.987321
H	1.559907	-3.120609	-3.977195
C	2.685134	-3.535436	-2.171689
C	3.926069	-3.853003	-0.080673
C	5.473123	-0.311784	2.003474
C	5.922671	-1.301549	-0.195195
H	4.937378	0.577807	3.906407
H	5.718493	2.948850	-0.211748
C	4.495706	4.028625	-1.631667
H	3.031419	5.000927	-2.869713
H	0.673396	4.612321	-2.770423
H	-1.222109	4.839487	-1.441369
H	-2.124072	5.475190	0.692830
C	-1.601211	4.571921	2.568107
H	-0.838588	3.533480	4.303864
C	2.928490	-4.900104	-2.483791
C	4.132431	-5.174915	-0.407971
H	4.306109	-3.465400	0.857160
C	6.875946	-0.231331	2.216317
C	7.276049	-1.196006	0.037800
H	5.567911	-1.702241	-1.137503
H	5.338637	4.444262	-2.175653
H	-2.352295	5.126487	3.122017
H	2.534369	-5.298618	-3.415158
C	3.639755	-5.705050	-1.624372
H	4.677272	-5.818640	0.276487
H	7.236754	0.190025	3.151085
C	7.761525	-0.665541	1.257104
H	7.978955	-1.519444	-0.724431
H	3.817868	-6.747414	-1.871288
H	8.831591	-0.595026	1.427909
C	-1.284863	-0.123665	3.090563
H	-0.218314	-0.360226	3.079305
H	-1.385139	0.955840	3.243552
H	-1.771733	-0.646972	3.921001
C	-4.759570	-4.312015	1.366369
O	-3.037827	-3.251207	2.305491
C	-3.744815	-4.400688	2.274102
H	-5.473296	-5.083958	1.113837
H	-3.415103	-5.178066	2.947537

C	-3.639506	4.124792	-2.413747
H	-2.763496	3.908290	-3.027537
H	-3.730324	5.196294	-2.223986
H	-4.532948	3.803477	-2.962355
O	-6.743702	-1.789303	0.247604
C	-7.648574	-2.540101	1.044535
H	-7.180575	-2.896052	1.972264
H	-8.471498	-1.867502	1.294636
H	-8.045704	-3.405056	0.490930

TS3a

B3LYP-D3 SCF energy: -2999.58109325 a.u.
B3LYP-D3 enthalpy: -2998.741957 a.u.
B3LYP-D3 Gibbs free energy: -2998.886153 a.u.
wB97X-D SCF energy in solution: -3000.78149674 a.u.
wB97X-D enthalpy: -2999.942360 a.u.
wB97X-D Gibbs free energy: -3000.086556 a.u.
Imaginary frequency: -331.1949 cm-1

Cartesian coordinates

ATOM	X	Y	Z
C	-2.410078	1.111483	1.332074
C	-1.780537	0.109132	1.838177
C	-2.510618	2.532293	1.497807
C	-2.880196	3.636040	0.759062
C	-3.375443	2.470969	-1.476187
H	-3.756284	2.697411	-2.478763
C	-4.229884	1.424083	-0.833206
C	-3.660102	0.452047	-0.072397
C	-3.938047	-0.860601	0.261517
H	-3.799313	-1.277236	1.254527
O	-4.613985	-1.657048	-0.619605
C	-4.739080	-3.008012	-0.268615
O	-4.369640	-3.431637	0.794217
H	-2.344893	2.114930	-1.581497
C	-3.347050	3.792294	-0.663120
H	-2.634199	4.459954	-1.177368
Rh	-1.735481	-0.525829	-0.188653
C	-5.720614	1.562755	-0.911921
H	-6.241857	0.724344	-0.445042
H	-6.023124	2.502196	-0.432387
H	-6.029252	1.636085	-1.963521
C	-1.824401	-0.931558	-2.089728
O	-1.919643	-1.161359	-3.207980
P	0.596340	-0.848342	-0.118910
O	1.460707	-0.109216	1.059539
O	1.285585	-0.275584	-1.506213
N	1.066221	-2.442674	0.126928
C	1.727673	1.263529	0.901791
C	2.688204	-0.255094	-1.590215
C	1.993572	-2.877760	1.136537
C	0.188636	-3.418507	-0.472768
C	1.006635	2.162514	1.712587

C	2.678337	1.666177	-0.019160
C	3.407130	0.646120	-0.823542
C	3.295957	-1.176570	-2.466818
C	3.363989	-2.725795	0.928299
C	1.487813	-3.388242	2.352508
C	-0.841739	-3.999829	0.297017
C	0.307639	-3.690451	-1.837511
H	0.291028	1.779986	2.427526
C	1.224667	3.511215	1.572672
C	2.873939	3.078199	-0.210047
C	4.839577	0.538735	-0.827378
H	2.666327	-1.808954	-3.082191
C	4.667706	-1.239306	-2.529543
H	3.713446	-2.325989	-0.016661
C	4.263060	-3.065448	1.937880
C	2.418798	-3.715366	3.357614
C	0.061186	-3.575843	2.616496
C	-0.946055	-3.815509	1.742698
C	-1.774524	-4.819216	-0.369252
C	-0.620025	-4.513984	-2.474547
H	1.122093	-3.235621	-2.389554
H	0.671807	4.216793	2.186321
C	2.143224	4.004077	0.609023
C	3.736924	3.604696	-1.210879
C	5.470167	-0.414294	-1.696817
C	5.664521	1.315049	0.032524
H	5.152589	-1.940018	-3.203616
H	5.328499	-2.932090	1.776577
C	3.786984	-3.560013	3.155853
H	2.053090	-4.103939	4.304553
H	-0.200847	-3.596749	3.673149
H	-1.935171	-4.001649	2.156148
H	-2.584285	-5.258998	0.204187
C	-1.669062	-5.070083	-1.735144
H	-0.529550	-4.712450	-3.538047
C	2.336671	5.399785	0.425752
C	3.893477	4.964159	-1.368360
H	4.273081	2.923969	-1.861301
C	6.887164	-0.523699	-1.698217
C	7.034447	1.174460	0.014736
H	5.202304	2.018805	0.715094
H	4.482220	-3.821262	3.948040
H	-2.394814	-5.715593	-2.222123
H	1.785088	6.088111	1.061337
C	3.196679	5.873937	-0.537699
H	4.556520	5.342131	-2.141198
H	7.352752	-1.242048	-2.368128
C	7.655923	0.254431	-0.863705
H	7.644880	1.773205	0.684520
H	3.336888	6.942780	-0.669529
H	8.737744	0.160100	-0.870201
C	-1.211578	-0.424223	3.097605
H	-0.143338	-0.629515	2.978895
H	-1.359549	0.294517	3.911470
H	-1.685232	-1.374076	3.367731

C	-2.656877	4.770340	1.609339
O	-2.050105	2.937981	2.732434
C	-2.166803	4.294076	2.783010
H	-1.886329	4.753215	3.719049
H	-2.860369	5.800484	1.354871
C	-5.337314	-3.776341	-1.406956
H	-5.661286	-4.755667	-1.053513
H	-6.171279	-3.229889	-1.854933
H	-4.566529	-3.904738	-2.176784
O	-4.614674	4.433643	-0.608217
C	-4.991176	5.117546	-1.796419
H	-5.182126	4.430199	-2.633321
H	-5.914748	5.652141	-1.565313
H	-4.220892	5.840620	-2.102412

TS3b

B3LYP-D3 SCF energy: -2999.58458655 a.u.
B3LYP-D3 enthalpy: -2998.746086 a.u.
B3LYP-D3 Gibbs free energy: -2998.889544 a.u.
wB97X-D SCF energy in solution: -3000.78113967 a.u.
wB97X-D enthalpy: -2999.942639 a.u.
wB97X-D Gibbs free energy: -3000.086097 a.u.
Imaginary frequency: -311.1956 cm-1

Cartesian coordinates

ATOM	X	Y	Z
C	1.429734	1.792841	1.725548
C	1.661449	0.608520	2.206115
C	0.331407	2.695411	1.713151
C	-0.082179	3.818630	1.004975
C	1.485784	3.700911	-0.994886
H	1.722995	4.277071	-1.896117
C	2.751126	3.316759	-0.303744
C	2.868854	2.185159	0.433757
C	3.903586	1.301044	0.710214
H	4.093502	0.881421	1.696784
O	4.989296	1.285192	-0.156988
C	5.631478	0.074485	-0.307689
O	5.204557	-0.946807	0.176110
H	0.894792	2.831616	-1.288661
C	0.599543	4.601321	-0.082924
H	1.238646	5.376732	0.370764
Rh	2.088205	0.137681	0.204574
C	3.875211	4.319714	-0.302919
H	4.724870	3.994452	0.300337
H	3.532705	5.294911	0.071251
H	4.222721	4.482976	-1.332404
C	2.403288	0.025062	-1.721840
O	2.586041	0.067744	-2.852226
P	0.102514	-1.123240	0.060212
O	-0.852559	-0.902686	-1.255759
O	-0.893280	-0.707680	1.312097
N	0.258029	-2.794671	0.021972

C	-1.560309	0.306344	-1.315915
C	-2.218316	-1.163875	1.292649
C	-0.283876	-3.628754	-1.016918
C	1.399312	-3.278358	0.760165
C	-1.096370	1.282858	-2.220232
C	-2.667610	0.486798	-0.505004
C	-3.112852	-0.621342	0.385811
C	-2.571679	-2.166872	2.217748
C	-1.598071	-4.082279	-0.920445
C	0.512063	-3.907412	-2.149045
C	2.655430	-3.366705	0.120499
C	1.262208	-3.553584	2.121955
H	-0.245202	1.047412	-2.850452
C	-1.735872	2.498280	-2.278671
C	-3.307446	1.773188	-0.520568
C	-4.432077	-1.186621	0.321224
H	-1.826240	-2.513436	2.924947
C	-3.850616	-2.670399	2.206173
H	-2.178682	-3.827717	-0.039730
C	-2.152297	-4.826401	-1.961213
C	-0.076168	-4.651858	-3.189224
C	1.893461	-3.443847	-2.289834
C	2.811227	-3.194322	-1.323222
C	3.776140	-3.668274	0.917289
C	2.382064	-3.883728	2.884946
H	0.274704	-3.493828	2.567365
H	-1.387414	3.269712	-2.958313
C	-2.832600	2.781917	-1.423428
C	-4.368262	2.110029	0.365938
C	-4.800448	-2.215993	1.252495
C	-5.384644	-0.794951	-0.659572
H	-4.142532	-3.438806	2.916797
H	-3.179900	-5.170228	-1.891765
C	-1.387240	-5.110537	-3.096696
H	0.513109	-4.874138	-4.075054
H	2.240623	-3.365332	-3.318696
H	3.817209	-2.933240	-1.645639
H	4.756568	-3.696944	0.453112
C	3.642812	-3.922898	2.279327
H	2.274838	-4.094529	3.944874
C	-3.450454	4.061223	-1.425940
C	-4.942056	3.362202	0.341116
H	-4.724826	1.367990	1.070743
C	-6.105640	-2.774887	1.191933
C	-6.637312	-1.366322	-0.697557
H	-5.112978	-0.042385	-1.390678
H	-1.815899	-5.684978	-3.912490
H	4.522398	-4.160879	2.870168
H	-3.075192	4.814667	-2.112230
C	-4.486513	4.348252	-0.567320
H	-5.751875	3.596287	1.026403
H	-6.374701	-3.546501	1.908778
C	-7.008702	-2.358738	0.241060
H	-7.346615	-1.055607	-1.459202
H	-4.952711	5.329179	-0.578886

H	-8.002194	-2.795366	0.201400
C	1.611305	-0.214119	3.430562
H	2.410590	-0.962167	3.456681
H	1.656383	0.411087	4.328582
H	0.659897	-0.761573	3.427187
C	-1.403281	4.109403	1.456503
O	-0.694505	2.294204	2.551260
C	-1.714099	3.165545	2.389198
H	-2.588834	2.987835	2.995887
H	-2.040237	4.892682	1.079603
C	6.867205	0.234163	-1.146911
H	7.341222	-0.737824	-1.284002
H	6.603940	0.666023	-2.118290
H	7.563056	0.924972	-0.659570
O	-0.417238	5.228419	-0.848162
C	-0.021572	6.440074	-1.472059
H	0.795415	6.291326	-2.193566
H	-0.896822	6.814106	-2.007837
H	0.294219	7.189237	-0.731018

TS4a

B3LYP-D3 SCF energy: -1370.65992609 a.u.
B3LYP-D3 enthalpy: -1370.297425 a.u.
B3LYP-D3 Gibbs free energy: -1370.390783 a.u.
wB97X-D SCF energy in solution: -1371.86550834 a.u.
wB97X-D enthalpy: -1371.503007 a.u.
wB97X-D Gibbs free energy: -1371.596365 a.u.
Imaginary frequency: -213.3705 cm-1

Cartesian coordinates

ATOM	X	Y	Z
C	-0.972928	-0.745704	-0.895769
C	0.014118	-1.423882	-1.362538
C	-2.334253	-0.851868	-0.542617
C	-3.310723	-0.088746	0.082951
C	-1.942008	1.640541	1.375958
H	-2.126157	2.509303	2.015909
C	-0.812917	1.966484	0.434484
C	0.017437	1.052829	-0.079142
H	-1.691127	0.793272	2.022127
C	-3.261712	1.319552	0.633164
Rh	1.073725	-0.802942	0.382453
C	2.791336	-1.702270	-0.263040
O	3.710052	-2.228780	-0.676138
C	-0.666822	3.409429	0.009166
H	-0.475728	4.025811	0.897675
H	0.154003	3.556667	-0.695004
H	-1.604048	3.754246	-0.435701
C	1.810531	0.098745	2.023353
O	2.219419	0.667040	2.920679
C	0.479997	-2.216394	-2.523450
H	0.841609	-3.197551	-2.195006
H	-0.335014	-2.358405	-3.240487

H	1.314166	-1.715425	-3.029504
C	-4.457208	-0.927470	0.185041
O	-2.843924	-2.119224	-0.793301
C	-4.123014	-2.131078	-0.370532
H	-4.668517	-3.049008	-0.534948
H	-5.407096	-0.659946	0.626813
O	-3.442033	2.303091	-0.369856
C	-4.745616	2.341715	-0.939845
H	-4.756856	3.190132	-1.626449
H	-4.974905	1.423675	-1.497341
H	-5.513294	2.492760	-0.166275
H	-4.078509	1.392533	1.369915
C	0.236771	-2.386061	1.449003
O	-0.313538	-3.249980	1.942876
C	1.250780	1.017897	-0.763626
H	1.325018	0.722187	-1.807320
O	2.217417	1.931119	-0.361267
C	3.494779	1.719019	-0.857763
O	3.762966	0.748166	-1.521177
C	4.414556	2.827559	-0.437735
H	5.419386	2.623263	-0.807363
H	4.054864	3.781237	-0.838268
H	4.423042	2.914419	0.653926

TS4b

B3LYP-D3 SCF energy: -1370.66040199 a.u.
B3LYP-D3 enthalpy: -1370.298143 a.u.
B3LYP-D3 Gibbs free energy: -1370.391072 a.u.
wB97X-D SCF energy in solution: -1371.86607446 a.u.
wB97X-D enthalpy: -1371.503815 a.u.
wB97X-D Gibbs free energy: -1371.596744 a.u.
Imaginary frequency: -222.7479 cm-1

Cartesian coordinates

ATOM	X	Y	Z
C	0.815695	-0.938048	-1.028644
C	-0.267620	-1.610361	-1.232314
C	2.191896	-1.074331	-0.809095
C	3.276982	-0.267233	-0.470983
C	2.140579	1.620634	0.770660
H	2.445187	2.530829	1.296722
C	0.909002	1.895571	-0.034736
C	-0.020228	0.974032	-0.323176
C	-1.315396	0.946172	-0.890821
H	-1.506403	0.524253	-1.874386
O	-2.174798	1.982342	-0.542034
C	-3.508930	1.796366	-0.872392
O	-3.903312	0.765529	-1.358153
H	1.985831	0.836512	1.516192
C	3.323409	1.205726	-0.170207
H	3.243226	1.774235	-1.111271
Rh	-1.140745	-0.709330	0.476982
C	-2.938928	-1.596943	0.120280

O	-3.919457	-2.109793	-0.140730
C	0.818918	3.264441	-0.669390
H	-0.038147	3.364339	-1.337427
H	1.733654	3.491175	-1.233305
H	0.735373	4.025418	0.117468
C	-1.704252	0.446097	2.028258
O	-2.011249	1.152054	2.866403
C	-0.869950	-2.535896	-2.223427
H	-1.237253	-3.438726	-1.722082
H	-1.730269	-2.068818	-2.718280
H	-0.135972	-2.822432	-2.983228
C	4.389533	-1.135509	-0.320493
O	2.615884	-2.400726	-0.837032
C	3.933953	-2.402192	-0.568260
H	4.421025	-3.366501	-0.595489
H	5.388484	-0.835606	-0.046545
C	-4.313017	3.014880	-0.524908
H	-5.359809	2.839964	-0.773242
H	-4.210535	3.238533	0.542363
H	-3.936093	3.881530	-1.077991
O	4.570302	1.461457	0.437017
C	5.039758	2.794844	0.285087
H	4.363875	3.528168	0.748504
H	6.006179	2.841813	0.789946
H	5.170814	3.052579	-0.776181
C	-0.242630	-2.158300	1.677100
O	0.327842	-2.965506	2.239378

Assignment of absolute configuration of 17 using VCD

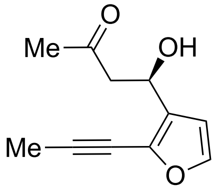

(*R*)-**17**

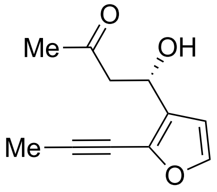

(*S*)-**17**

VCD Measurements

The $CDCl_3$ solvent (Cambridge Isotope Labs Silver Foil) was run through a small plug of activated basic alumina immediately before use in each case.

To a small vial containing ~9 mg of (*R*)-**17** or (*S*)-**17** was added 135 μL of $CDCl_3$. The resulting solution was transferred to a liquid IR cell (BaF_2, 100 μm cell path) and placed in the measurement chamber. The instrument was a BioTools, Inc. (Jupiter, FL) ChiralIR 2X Dual PEM FT-VCD instrument, set to 4 cm^{-1} resolution and 1400 cm^{-1} PEM maximum frequency (both PEMs). The samples were measured overnight in one-hour blocks. The IR data from the first block was solvent and water vapor subtracted, and offset to zero at 2000 cm^{-1}. The VCD data blocks were averaged and then processed by enantiomer subtraction ((E1 – E2)/2) and offset to zero at 2000 cm^{-1}.

VCD Calculations

(*R*)-**17** was constructed using ComputeVOA (BioTools, Jupiter, FL). A thorough conformer search was performed at the MM level using the MMF94 force field and a 7 kcal/mol energy window. All conformers obtained were subjected to DFT geometry optimization and frequency calculation using Gaussian 09 (Wallingford, CT)[12] at the B3LYP/6-31G(d) level. The resulting lowest energy unique conformers were then reoptimized at the B3LYP/cc-pVTZ level starting with the optimized

structures from the first DFT calculations. The computed frequencies (wavenumbers) were scaled using a scaling factor of 0.982 - values obtained from CompareVOA. The resulting spectra were Boltzmann averaged and plotted at 5 cm^{-1} resolution for comparison to the experimental spectra. Relative electronic energies were used to weigh the conformers in the computed VCD spectra shown in Figures 2 and 4 of the main text. The computed VCD spectra using Gibbs free energy-weighted values are shown in Figure S2.

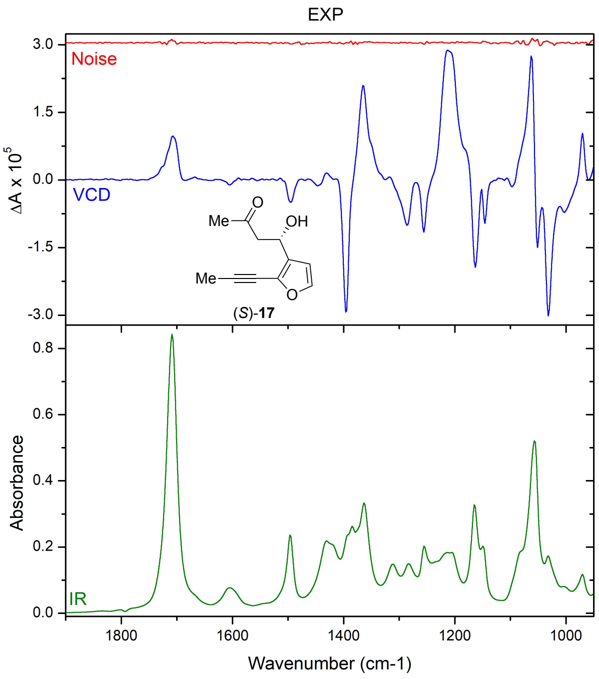

Figure S9. Experimental VCD (blue) and IR (green) spectra of (*S*)-**17**.

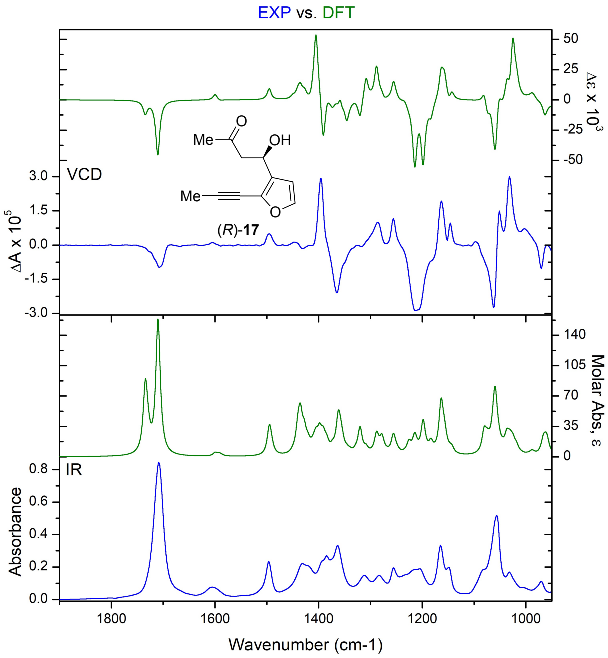

Figure S10. DFT-calculated (green) and experimental (blue) VCD and IR spectra of (*R*)-**17** (Boltzmann weighted based Gibbs free energies).

Energies and cartesian coordinates used in VCD calculations

There were no imaginary frequencies in any of the conformers.

Functional / Basis Set = B3LYP / cc-pVTZ (with CPCM (chloroform)): 6 conformers at 1% or greater Boltzmann weight (Electronic Energy Weighted).

(*R*)-**17** was calculated

Conformer # 1

Electronic Energy: -652.237026 Hartree

Gibbs Free Energy: -652.076802 Hartree

Center Number	Atomic Number	Atomic Type	Coordinates (Angstroms) X	Y	Z
1	8	0	-2.642067	-1.414287	0.514628
2	6	0	-1.970247	-2.595188	0.517981
3	6	0	-0.683328	-2.416642	0.134418
4	6	0	-0.529848	-1.021080	-0.134843
5	6	0	-1.749856	-0.448149	0.106705

6	6	0	0.717173	-0.321213	-0.572617
7	6	0	-2.212210	0.876662	0.013513
8	6	0	-2.596811	2.016299	-0.074251
9	6	0	-3.062780	3.388849	-0.181127
10	6	0	1.701357	-0.136751	0.593366
11	6	0	2.976854	0.588714	0.213042
12	8	0	3.318223	0.718030	-0.950088
13	6	0	3.806479	1.140115	1.340933
14	8	0	1.310341	-1.085498	-1.622764
15	1	0	-2.539931	-3.459592	0.808212
16	1	0	0.068554	-3.179694	0.034737
17	1	0	0.443653	0.672270	-0.944212
18	1	0	-4.011091	3.514492	0.343634
19	1	0	-3.215267	3.675714	-1.223179
20	1	0	-2.343263	4.082219	0.257642
21	1	0	1.221459	0.393092	1.419016
22	1	0	1.992381	-1.115689	0.989194
23	1	0	3.278531	1.983089	1.793630
24	1	0	4.772763	1.475121	0.973339
25	1	0	3.937942	0.392349	2.124570
26	1	0	2.121607	-0.609030	-1.853520

Conformer # 2

Electronic Energy: -652.234824 Hartree

Gibbs Free Energy: -652.074851 Hartree

Center Number	Atomic Number	Atomic Type	Coordinates (Angstroms) X	Y	Z
1	8	0	-1.576210	1.997353	-0.745623
2	6	0	-0.429119	2.712265	-0.615268
3	6	0	0.509759	1.997972	0.051586
4	6	0	-0.085195	0.737159	0.369431
5	6	0	-1.359408	0.783165	-0.133022
6	6	0	0.530180	-0.423223	1.110322
7	6	0	-2.413355	-0.147619	-0.124152
8	6	0	-3.305726	-0.958987	-0.105978

9	6	0	-4.384310	-1.933111	-0.085885
10	6	0	1.278580	-1.395857	0.179400
11	6	0	2.562199	-0.861741	-0.424120
12	8	0	3.194819	0.035274	0.108303
13	6	0	3.037049	-1.508181	-1.695879
14	8	0	1.358850	0.004452	2.186883
15	1	0	-0.438713	3.700088	-1.039633
16	1	0	1.504430	2.324541	0.297994
17	1	0	-0.284654	-0.987046	1.566098
18	1	0	-4.015920	-2.930859	-0.330412
19	1	0	-5.151287	-1.671645	-0.816568
20	1	0	-4.858697	-1.980016	0.896039
21	1	0	1.562696	-2.283672	0.755874
22	1	0	0.615475	-1.747243	-0.612148
23	1	0	2.995817	-2.595507	-1.613038
24	1	0	4.046835	-1.184941	-1.934214
25	1	0	2.362973	-1.228362	-2.509455
26	1	0	2.197449	0.276962	1.784351

Conformer # 3

Electronic Energy: -652.234637 Hartree

Gibbs Free Energy: -652.076066 Hartree

Center Number	Atomic Number	Atomic Type	Coordinates (Angstroms) X	Y	Z
1	8	0	2.598870	-1.518910	-0.451541
2	6	0	1.890270	-2.677291	-0.398308
3	6	0	0.606117	-2.438616	-0.040388
4	6	0	0.491240	-1.024813	0.147690
5	6	0	1.732528	-0.504712	-0.109582
6	6	0	-0.732076	-0.259239	0.553401
7	6	0	2.233975	0.808914	-0.087353
8	6	0	2.649252	1.940802	-0.062798
9	6	0	3.149423	3.305013	-0.034831
10	6	0	-1.806427	-0.248914	-0.528143
11	6	0	-2.918540	0.758467	-0.271989

12	8	0	-2.729736	1.768664	0.372173
13	6	0	-4.255475	0.447087	-0.894411
14	8	0	-1.349061	-0.841544	1.711805
15	1	0	2.435324	-3.573279	-0.635219
16	1	0	-0.169065	-3.173684	0.089546
17	1	0	-0.451310	0.775003	0.763440
18	1	0	2.457725	3.985314	-0.534484
19	1	0	4.111816	3.371875	-0.544498
20	1	0	3.286698	3.656019	0.989523
21	1	0	-1.360922	0.021749	-1.490191
22	1	0	-2.224519	-1.249301	-0.651373
23	1	0	-4.137444	0.171400	-1.944036
24	1	0	-4.926638	1.296995	-0.802289
25	1	0	-4.690966	-0.418067	-0.388182
26	1	0	-0.733008	-0.769883	2.448542

Conformer # 4

Electronic Energy: -652.234530 Hartree

Gibbs Free Energy: -652.074359 Hartree

Center Number	Atomic Number	Atomic Type	Coordinates (Angstroms) X	Y	Z
1	8	0	-2.878720	-0.916125	-0.452661
2	6	0	-2.607303	-2.245504	-0.438869
3	6	0	-1.315720	-2.461578	-0.089677
4	6	0	-0.727108	-1.179179	0.131408
5	6	0	-1.719181	-0.260629	-0.103639
6	6	0	0.694242	-0.919578	0.536405
7	6	0	-1.765971	1.143822	-0.056037
8	6	0	-1.806807	2.348363	-0.021693
9	6	0	-1.852607	3.799891	0.030290
10	6	0	1.480927	-0.182294	-0.556892
11	6	0	2.937387	0.056511	-0.214422
12	8	0	3.356700	-0.058451	0.924584
13	6	0	3.845200	0.446808	-1.349834
14	8	0	0.700675	-0.182204	1.759975

15	1	0	-3.424003	-2.897235	-0.691687
16	1	0	-0.828873	-3.417163	0.007316
17	1	0	1.173159	-1.891851	0.696598
18	1	0	-2.884481	4.151566	0.075126
19	1	0	-1.385044	4.243945	-0.850511
20	1	0	-1.332215	4.174718	0.913291
21	1	0	1.423329	-0.726185	-1.502596
22	1	0	1.025924	0.796073	-0.741921
23	1	0	3.984627	-0.414517	-2.007720
24	1	0	4.810857	0.770308	-0.970450
25	1	0	3.391522	1.234733	-1.952768
26	1	0	1.637610	-0.049948	1.967892

Conformer # 5

Electronic Energy: -652.233539 Hartree

Gibbs Free Energy: -652.073383 Hartree

Center Number	Atomic Number	Atomic Type	Coordinates (Angstroms) X	Y	Z
1	8	0	2.554337	-1.612465	-0.320982
2	6	0	1.790262	-2.735543	-0.270731
3	6	0	0.499399	-2.426588	-0.002884
4	6	0	0.440958	-1.002915	0.127418
5	6	0	1.718639	-0.548552	-0.070776
6	6	0	-0.766068	-0.168773	0.430388
7	6	0	2.280083	0.740804	-0.064464
8	6	0	2.751989	1.850542	-0.055539
9	6	0	3.324331	3.186304	-0.046221
10	6	0	-1.799695	-0.215377	-0.697042
11	6	0	-3.073435	0.568476	-0.421395
12	8	0	-4.158876	0.075923	-0.650223
13	6	0	-2.941333	1.975162	0.107789
14	8	0	-1.444342	-0.632488	1.607546
15	1	0	2.305964	-3.663170	-0.442355
16	1	0	-0.316040	-3.120953	0.101901
17	1	0	-0.438217	0.864148	0.576332

18	1	0	4.388344	3.148396	-0.284446
19	1	0	3.216808	3.654790	0.933648
20	1	0	2.838512	3.828544	-0.782844
21	1	0	-1.344362	0.200155	-1.600462
22	1	0	-2.082594	-1.245765	-0.908181
23	1	0	-2.599233	1.939317	1.144026
24	1	0	-3.905193	2.475618	0.068094
25	1	0	-2.200798	2.541422	-0.459117
26	1	0	-0.822922	-0.611509	2.343413

Conformer # 6

Electronic Energy: -652.233505 Hartree

Gibbs Free Energy: -652.073871 Hartree

Center Number	Atomic Number	Atomic Type	Coordinates (Angstroms) X	Y	Z
1	8	0	-1.610628	-1.815246	1.067091
2	6	0	-0.583198	-2.704822	1.058207
3	6	0	0.430205	-2.258296	0.279435
4	6	0	0.017052	-0.990341	-0.242367
5	6	0	-1.237129	-0.763745	0.261702
6	6	0	0.773020	-0.109268	-1.191151
7	6	0	-2.150590	0.293859	0.105716
8	6	0	-2.927909	1.204818	-0.033552
9	6	0	-3.861147	2.306777	-0.195588
10	6	0	2.160754	0.279162	-0.690255
11	6	0	2.179387	1.275360	0.451733
12	8	0	1.207347	1.940465	0.745294
13	6	0	3.487342	1.409384	1.191531
14	8	0	0.997890	-0.780184	-2.444666
15	1	0	-0.725156	-3.593665	1.646191
16	1	0	1.358024	-2.769159	0.087939
17	1	0	0.193280	0.800947	-1.355117
18	1	0	-4.661301	2.244321	0.543503
19	1	0	-4.317971	2.297500	-1.186847
20	1	0	-3.358107	3.266324	-0.064570

21	1	0	2.737748	-0.606471	-0.416886
22	1	0	2.706846	0.742865	-1.518622
23	1	0	3.652238	0.509951	1.790090
24	1	0	3.463390	2.276396	1.846524
25	1	0	4.323831	1.484585	0.495076
26	1	0	0.143578	-1.071208	-2.781507

CompareVOA results (based on Electronic Energy-weighted Boltzmann distributions)

((*R*)-**17**, (*R*) config):

Uniform Scaling Factor: 0.982

TNS (S_{fg}) IR = 92.0

TNS (S_{fg}) VCD = 80.4

SNS ((*R*) config) VCD = 79.5

SNS ((*S*) config) VCD = 2.3

ESI (Enantiomeric Similarity Index = SNS (*R*) – SNS (*S*) = 77.2

Confidence Level = 99

CompareVOA results (based on Gibbs free energy-weighted Boltzmann distributions)

(*R*)-**17**, (*R*) config):

Uniform Scaling Factor: 0.981

TNS (S_{fg}) IR = 93.0

TNS (S_{fg}) VCD = 79.0

SNS ((*R*) config) VCD = 81.5

SNS ((*S*) config) VCD = 5.4

ESI (Enantiomeric Similarity Index = SNS (*R*) – SNS (*S*) = 76.2

Confidence Level = 99

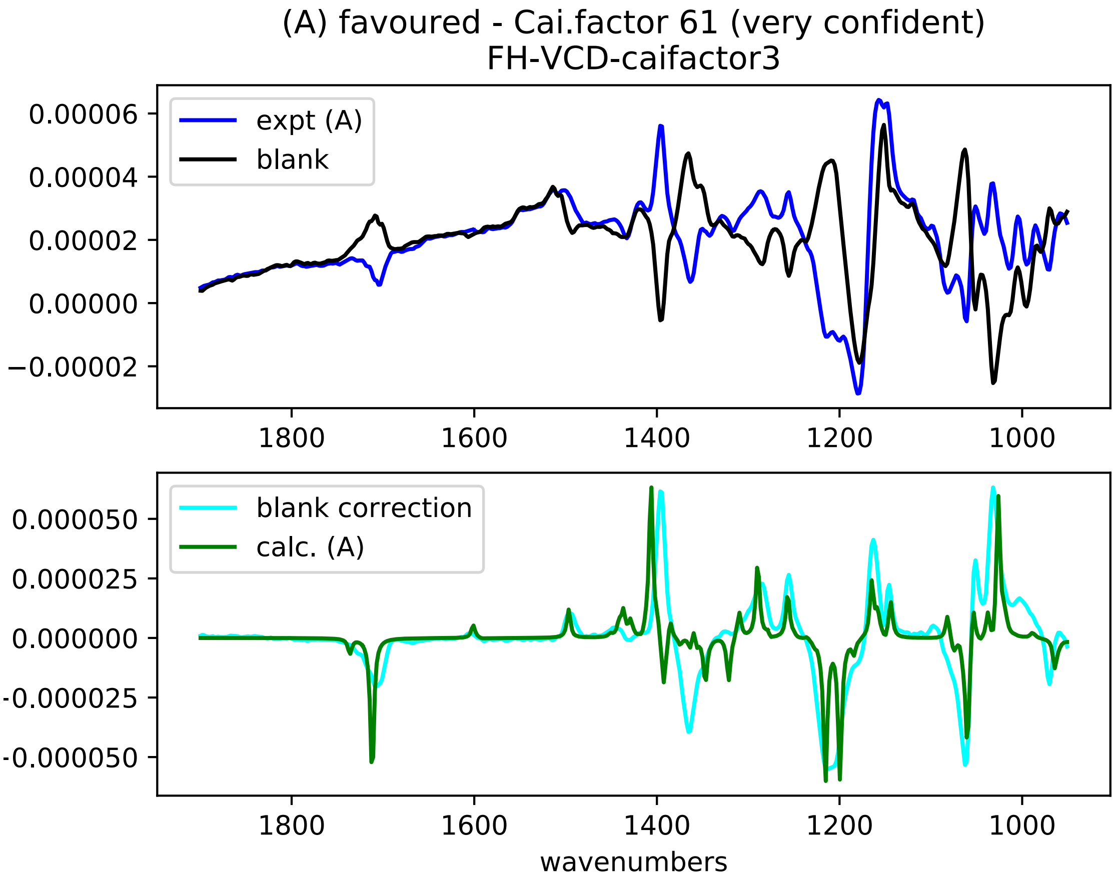

Figure S11. Cai.factor for (*R*)-**17**

##

Cai• VCD analysis

University of Cambridge, 2020

##

FH-VCD-caifactor3.txt (*R*)-**17**

minimum wavenumber: 950.0

maximum wavenumber: 1900.0

Lorentzian broadening: 5.0

Temperature for Boltzmann averaging (Kelvin): 298.0

Defined scaling factor: 0.982

Print out graphs: True

Boltzmann Analysis: True

Boltzmann Analysis Energy cut-off: 10.0

Unique calculated structure criteria: Energy: 0.0001 Frequency: 2.0

Extreme scale factor warning range: 0.01

Insufficient information criterion for Cai.factor: 10

Printing spectra in .csv file

Printing scaling factor analysis in .csv file

Printing two-line summary in .csv file

Experimental data for single enantiomer:

(A) /ihome/pliu/ltj6/bin/caifactor3/experimental/fh-88-cc_VCD-raw-avg.csv

Blank file:

/ihome/pliu/ltj6/bin/caifactor3/experimental/fh-02-178-cc_VCD_raw-avg-USE-AS-SOLVENT-BASELINE.csv

Calculation files:

/ihome/pliu/ltj6/bin/caifactor3/experimental/FHRRS

Only one filename listed for calculations

which is a directory containing the following files:

/ihome/pliu/ltj6/bin/caifactor3/experimental/FHRRS/fatemah1r-a_c4

/ihome/pliu/ltj6/bin/caifactor3/experimental/FHRRS/fatemah1r-a_c12

/ihome/pliu/ltj6/bin/caifactor3/experimental/FHRRS/fatemah1r-a_c3

/ihome/pliu/ltj6/bin/caifactor3/experimental/FHRRS/fatemah1r-a_c21

/ihome/pliu/ltj6/bin/caifactor3/experimental/FHRRS/fatemah1r-a_c8

/ihome/pliu/ltj6/bin/caifactor3/experimental/FHRRS/fatemah1r-a_c19

/ihome/pliu/ltj6/bin/caifactor3/experimental/FHRRS/fatemah1r-a_c15-nf1

/ihome/pliu/ltj6/bin/caifactor3/experimental/FHRRS/fatemah1r-a_c9

/ihome/pliu/ltj6/bin/caifactor3/experimental/FHRRS/fatemah1r-a_c27

/ihome/pliu/ltj6/bin/caifactor3/experimental/FHRRS/fatemah1r-a_c1

/ihome/pliu/ltj6/bin/caifactor3/experimental/FHRRS/fatemah1r-a_c2

/ihome/pliu/ltj6/bin/caifactor3/experimental/FHRRS/fatemah1r-a_c18

/ihome/pliu/ltj6/bin/caifactor3/experimental/FHRRS/fatemah1r-a_c11

/ihome/pliu/ltj6/bin/caifactor3/experimental/FHRRS/fatemah1r-a_c23

/ihome/pliu/ltj6/bin/caifactor3/experimental/FHRRS/fatemah1r-a_c5

/ihome/pliu/ltj6/bin/caifactor3/experimental/FHRRS/fatemah1r-a_c17

/ihome/pliu/ltj6/bin/caifactor3/experimental/FHRRS/fatemah1r-a_c25

/ihome/pliu/ltj6/bin/caifactor3/experimental/FHRRS/fatemah1r-a_c20-nf2

/ihome/pliu/ltj6/bin/caifactor3/experimental/FHRRS/fatemah1r-a_c24

/ihome/pliu/ltj6/bin/caifactor3/experimental/FHRRS/fatemah1r-a_c7

/ihome/pliu/ltj6/bin/caifactor3/experimental/FHRRS/fatemah1r-a_c16

/ihome/pliu/ltj6/bin/caifactor3/experimental/FHRRS/fatemah1r-a_c22

/ihome/pliu/ltj6/bin/caifactor3/experimental/FHRRS/fatemah1r-a_c14

/ihome/pliu/ltj6/bin/caifactor3/experimental/FHRRS/fatemah1r-a_c6

/ihome/pliu/ltj6/bin/caifactor3/experimental/FHRRS/fatemah1r-a_c10

/ihome/pliu/ltj6/bin/caifactor3/experimental/FHRRS/fatemah1r-a_c26

/ihome/pliu/ltj6/bin/caifactor3/experimental/FHRRS/fatemah1r-a_c13

Number of files: 27

Calculated data in Gaussian file

19 files rejected by energy cutoff

0 duplicate files removed

Unique Calculated Structures

Energy: -652.076802 hartrees, 0.000 kJ/mol, Boltzmann Factor: 1.000
/ihome/pliu/ltj6/bin/caifactor3/experimental/FHRRS/fatemah1r-a_c2

Energy: -652.076066 hartrees, 1.933 kJ/mol, Boltzmann Factor: 0.458
/ihome/pliu/ltj6/bin/caifactor3/experimental/FHRRS/fatemah1r-a_c1

Energy: -652.074851 hartrees, 5.123 kJ/mol, Boltzmann Factor: 0.127
/ihome/pliu/ltj6/bin/caifactor3/experimental/FHRRS/fatemah1r-a_c3

Energy: -652.074359 hartrees, 6.415 kJ/mol, Boltzmann Factor: 0.075
/ihome/pliu/ltj6/bin/caifactor3/experimental/FHRRS/fatemah1r-a_c8

Energy: -652.073997 hartrees, 7.365 kJ/mol, Boltzmann Factor: 0.051
/ihome/pliu/ltj6/bin/caifactor3/experimental/FHRRS/fatemah1r-a_c5

Energy: -652.073964 hartrees, 7.452 kJ/mol, Boltzmann Factor: 0.049
/ihome/pliu/ltj6/bin/caifactor3/experimental/FHRRS/fatemah1r-a_c14

Energy: -652.073871 hartrees, 7.696 kJ/mol, Boltzmann Factor: 0.045
/ihome/pliu/ltj6/bin/caifactor3/experimental/FHRRS/fatemah1r-a_c4

Energy: -652.073383 hartrees, 8.978 kJ/mol, Boltzmann Factor: 0.027
/ihome/pliu/ltj6/bin/caifactor3/experimental/FHRRS/fatemah1r-a_c12

Using all 8 unique conformations within energy cut-off

Defined scaling factor: 0.982

Single enantiomer result: Defined SF : 0.982 ; File (A) is assigned to the enantiomer calculated with Cai.factor 24

Single enantiomer blank: Defined SF : 0.982 ; File (A) is assigned to the enantiomer calculated with Cai.factor 62

Single enantiomer result: Opt. SF : 0.978 ; File (A) is assigned to the enantiomer calculated with Cai.factor 26

Single enantiomer blank: Opt. SF : 0.982 ; File (A) is assigned to the enantiomer calculated with Cai.factor 62

Average result over scaling factor range: 0.972 0.992

Experimental data for single enantiomer:

(A) /ihome/pliu/ltj6/bin/caifactor3/experimental/fh-88-cc_VCD-raw-avg.csv

Blank file:

/ihome/pliu/ltj6/bin/caifactor3/experimental/fh-02-178-cc_VCD_raw-avg-USE-AS-SOLVENT-BASELINE.csv

Single enantiomer summary

This is based on File (A) (cautiously confident)

Using the blank data, File (A) is very confident

Optimising the scale factor does not improve overall confidence substantially

Overall Cai.factor is 61 which means very confident assignment

Based on total confidence measure 10

Assignment of absolute and relative configuration of 14g using VCD

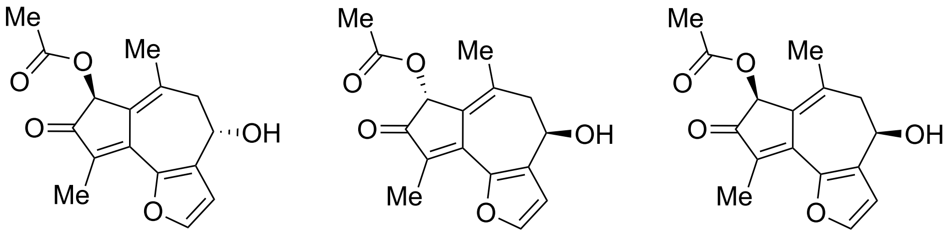

(2*S*,8*S*)-**14g** (2*R*,8*R*)-**14g** (2*S*,8*R*)-**14g**

VCD Measurements

The $CDCl_3$ solvent (Cambridge Isotope Labs Silver Foil) was run through a small plug of activated basic alumina immediately before use in each case.

To a small vial containing 2-6 mg of (2*S*,8*S*)-**14g**, (2*R*,8*R*)-**14g**, or (2*S*,8*R*)-**14g** was added 100 µL of $CDCl_3$. Same instrument, settings and measurement times as above. The VCD data blocks were averaged, and then the *trans* enantiomer pair was processed by enantiomer subtraction ((E1 – E2)/2) and offset to zero at 2000 cm^{-1}. The *cis* isomer was processed by subtraction of a solvent

baseline (12 hours acquisition) and then offset to zero at 2000 cm^{-1}. To determine the enantiomer pair, the *trans* isomers VCD spectra were also processed by solvent subtraction in the same way.

VCD Calculations

(2*R*,8*R*)-**14g** and (2*R*,8*S*)-**14g** were constructed using ComputeVOA (BioTools, Jupiter, FL). A thorough conformer search was performed at the MM level using the MMF94 force field and a 7 kcal/mol energy window. All conformers obtained were subjected to DFT optimization and frequency calculation using Gaussian 09 (Wallingford, CT)[12] at the B3LYP/6-31G(d) level. The resulting lowest energy unique conformers were then reoptimized at the B3LYP/cc-pVTZ level starting with the optimized structures from the first DFT calculations. The computed frequencies (wavenumbers) were scaled using scaling factors in a range of 0.984–0.986 - values obtained from CompareVOA. The resulting spectra were Boltzmann averaged and plotted at 5 cm^{-1} resolution for comparison to the experimental spectra.

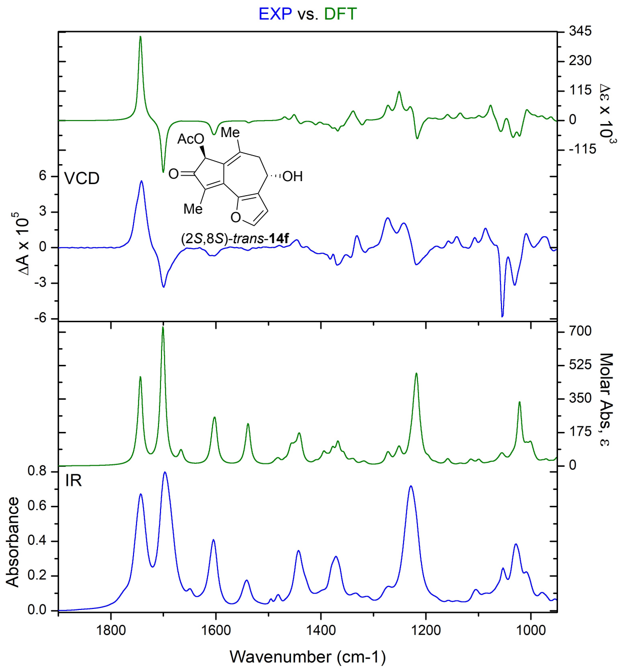

Figure S12. DFT calculated (green) and experimental (blue) VCD and IR spectra of (2*S*,8*S*)-**14g** (electronic energy weighted).

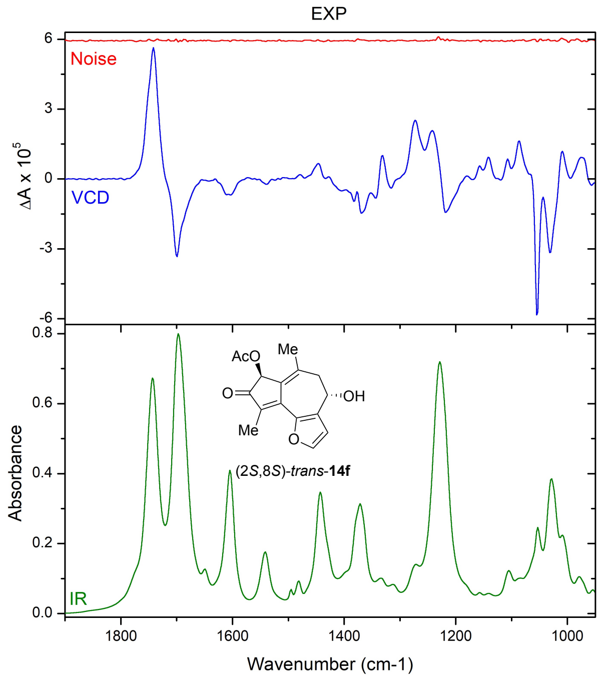

Figure S13. Experimental VCD (blue) and IR (green) spectra of (2*S*,8*S*)-**14g**.

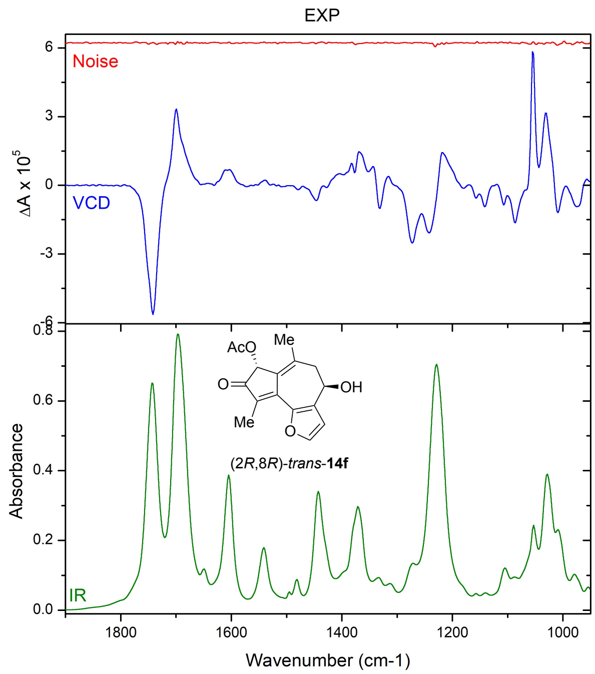

Figure S14. Experimental VCD (blue) and IR (green) spectra of (2*R*,8*R*)-**14g**.

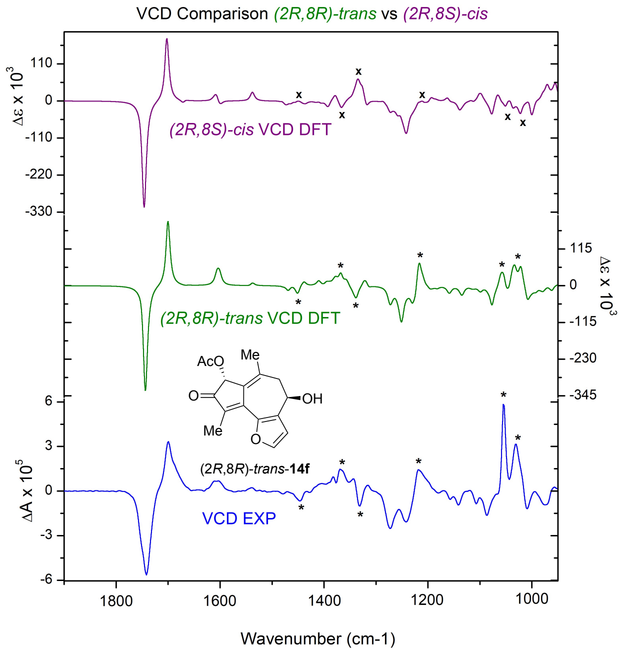

Figure S15. DFT calculated VCD spectra of (2*R*,8*S*)-**14g** (purple) vs. (2*R*,8*R*)-**14g** (green) and experimental VCD spectrum of (2*R*,8*R*)-**14g** (blue) (electronic energy weighted).

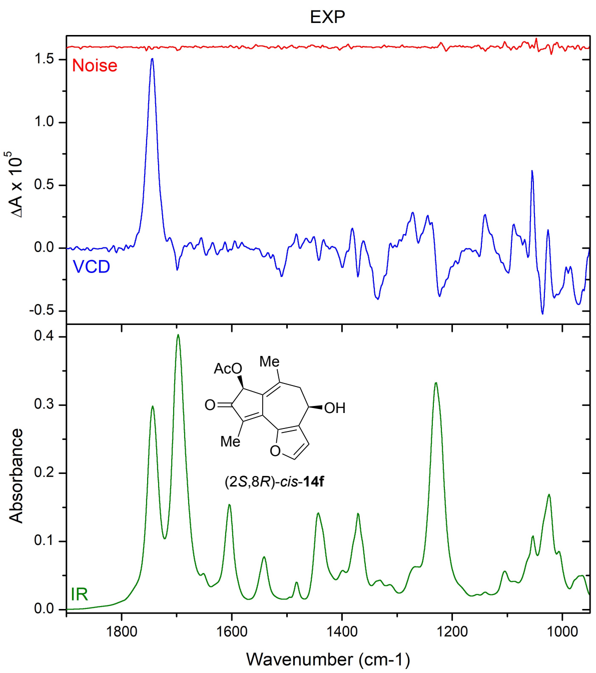

Figure S16. Experimental VCD (blue) and IR (green) spectra of (2*S*,8*R*)-**14g**.

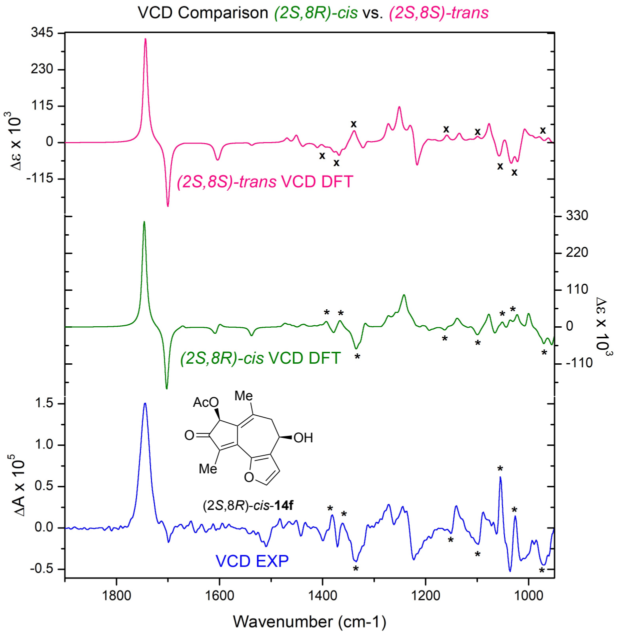

Figure S17. DFT calculated VCD spectra of (2*S*,8*S*)-**14g** (purple) vs. (2*S*,8*R*)-**14g** (green) and experimental VCD spectrum of (2*S*,8*R*)-**14g** (blue) (electronic energy weighted).

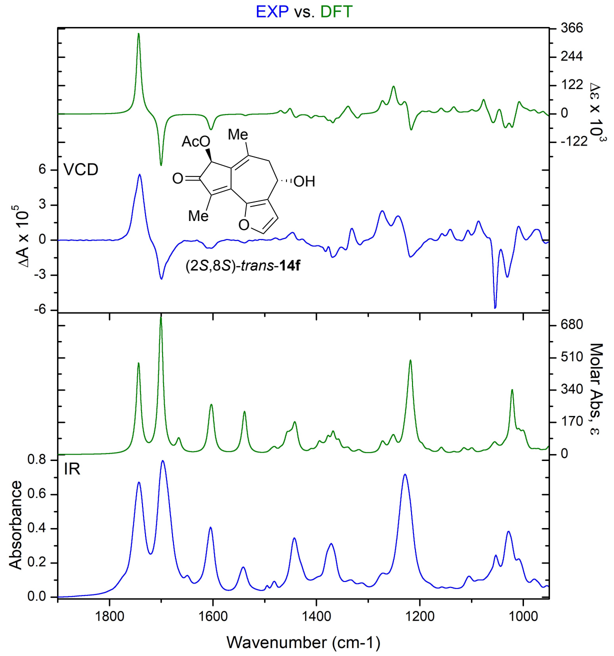

Figure S18. DFT calculated (green) and experimental (blue) VCD and IR spectra of (2*S*,8*S*)-**14g** (Boltzmann weighted based Gibbs free energies).

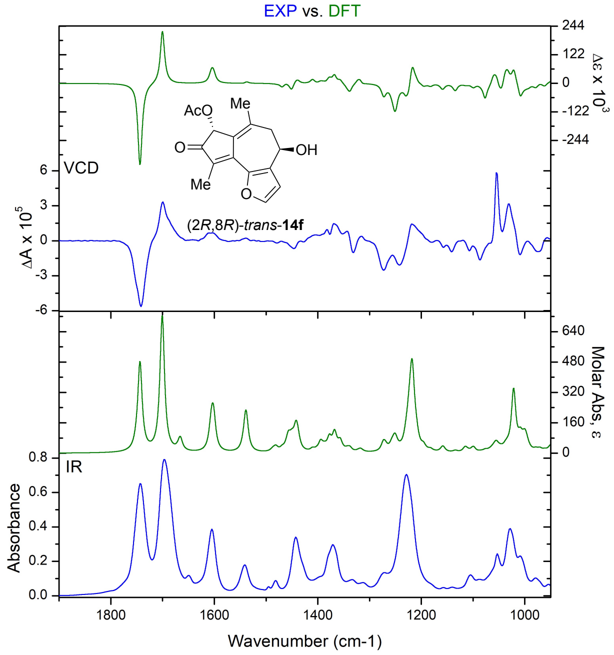

Figure S19. DFT calculated (green) and experimental (blue) VCD and IR spectra of (2*R*,8*R*)-**14g** (Boltzmann weighted based Gibbs free energies).

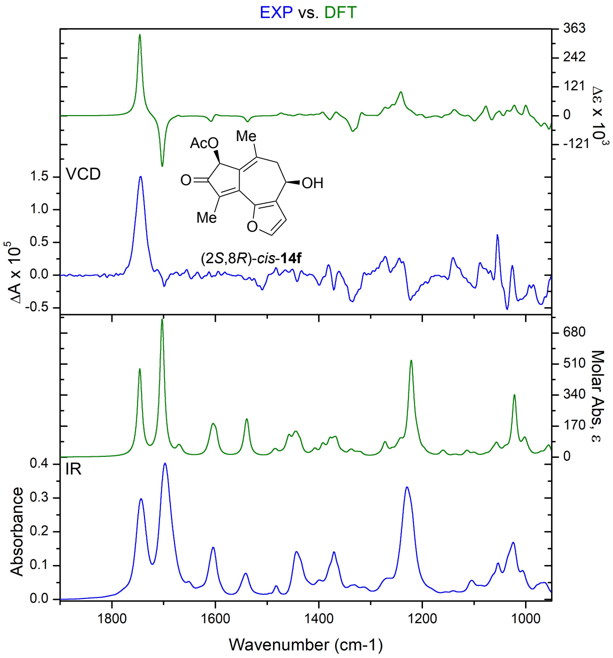

Figure S20. DFT calculated (green) and experimental (blue) VCD and IR spectra of (2*S*,8*R*)-**14g** (Boltzmann weighted based Gibbs free energies).

Energies and cartesian coordinates used in VCD calculations

There were no imaginary frequencies in any of the conformers.

(2*R*,8*R*)-**14g** and (2*R*,8*S*)-**14g** stereoisomers were calculated

(2*R*,8*R*)-**14g**:

Functional / Basis Set = B3LYP / cc-pVTZ (with CPCM (Chloroform)) : 9 conformers at 1% or greater Boltzmann weight (Electronic Energy Weighted).

```
Conformer # 1
Electronic Energy:  -995.802133 Hartree
Gibbs Free Energy: -995.559725 Hartree
Center     Atomic      Atomic             Coordinates (Angstroms)
 Number     Number       Type             X           Y           Z
 ---------------------------------------------------------------------
      1          6           0       -1.727744    0.245903   -0.466760
      2          6           0       -1.539073    1.755523   -0.310603
      3          6           0       -0.116853    2.027472   -0.190942
      4          6           0        0.573345    0.837541   -0.189717
      5          6           0       -0.326424   -0.315213   -0.397560
```

6	6	0	1.987302	0.701207	-0.007049
7	6	0	1.341844	-2.235545	-0.613942
8	6	0	2.327538	-1.798694	0.471643
9	6	0	2.777876	-0.378625	0.302393
10	8	0	-2.457193	2.557381	-0.261662
11	8	0	2.769618	1.833835	-0.042814
12	6	0	4.100669	0.118526	0.463584
13	6	0	-0.036878	-1.611214	-0.616284
14	6	0	-1.111627	-2.611309	-0.948153
15	6	0	4.035566	1.456163	0.235911
16	8	0	-2.551941	-0.228117	0.622442
17	6	0	-3.838552	-0.549607	0.356433
18	8	0	-4.315163	-0.563535	-0.752704
19	6	0	-4.574013	-0.886786	1.622011
20	6	0	0.380391	3.427051	-0.041185
21	8	0	3.437920	-2.692760	0.525850
22	1	0	-2.233204	0.029472	-1.405921
23	1	0	1.805009	-2.076569	-1.595481
24	1	0	1.224630	-3.314117	-0.505813
25	1	0	1.845727	-1.907570	1.446530
26	1	0	4.976662	-0.451376	0.716230
27	1	0	-2.082074	-2.168497	-1.147480
28	1	0	-1.224356	-3.329348	-0.130709
29	1	0	-0.815709	-3.190564	-1.826585
30	1	0	4.774579	2.237433	0.232124
31	1	0	-4.731210	0.027528	2.195706
32	1	0	-3.986584	-1.565096	2.238313
33	1	0	-5.534751	-1.329946	1.379568
34	1	0	0.988195	3.548389	0.855640
35	1	0	0.999688	3.728845	-0.887042
36	1	0	-0.472367	4.101009	0.018975
37	1	0	3.879637	-2.688376	-0.331646

Conformer # 2
Electronic Energy: -995.801970 Hartree
Gibbs Free Energy: -995.559958 Hartree

Center Number	Atomic Number	Atomic Type	Coordinates (Angstroms) X	Y	Z
1	6	0	-1.724480	0.255677	-0.462582
2	6	0	-1.533820	1.762612	-0.283497
3	6	0	-0.111276	2.030861	-0.164491
4	6	0	0.577385	0.840101	-0.178722
5	6	0	-0.324525	-0.309264	-0.397246
6	6	0	1.992368	0.699794	-0.011209
7	6	0	1.340623	-2.230521	-0.617916
8	6	0	2.312962	-1.788753	0.478990
9	6	0	2.781126	-0.383169	0.288654
10	8	0	-2.451363	2.564343	-0.219421

11	8	0	2.781855	1.826084	-0.069092
12	6	0	4.111348	0.101631	0.415509
13	6	0	-0.036950	-1.603793	-0.628773
14	6	0	-1.112955	-2.596438	-0.978701
15	6	0	4.051157	1.438687	0.182920
16	8	0	-2.553669	-0.233478	0.616128
17	6	0	-3.840225	-0.546824	0.341043
18	8	0	-4.312972	-0.543972	-0.769856
19	6	0	-4.581796	-0.898187	1.599257
20	6	0	0.390387	3.427178	-0.000236
21	8	0	3.473636	-2.621220	0.482889
22	1	0	-2.226861	0.054622	-1.406860
23	1	0	1.820665	-2.077084	-1.590184
24	1	0	1.212579	-3.309960	-0.514435
25	1	0	1.806232	-1.865926	1.448685
26	1	0	4.986957	-0.477829	0.643736
27	1	0	-0.809702	-3.171528	-1.857375
28	1	0	-1.239930	-3.319082	-0.167290
29	1	0	-2.078667	-2.147248	-1.186874
30	1	0	4.796251	2.213721	0.156529
31	1	0	-4.742092	0.010000	2.181796
32	1	0	-5.541364	-1.338644	1.347374
33	1	0	-3.997406	-1.582935	2.211275
34	1	0	-0.460008	4.100621	0.091565
35	1	0	0.989448	3.744268	-0.855217
36	1	0	1.019611	3.531686	0.883693
37	1	0	3.216419	-3.496272	0.790403

Conformer # 3
Electronic Energy: -995.801506 Hartree
Gibbs Free Energy: -995.559822 Hartree

Center Number	Atomic Number	Atomic Type	Coordinates (Angstroms) X	Y	Z
1	6	0	-1.718394	0.269317	-0.451021
2	6	0	-1.526891	1.771179	-0.233459
3	6	0	-0.103419	2.035365	-0.116514
4	6	0	0.582427	0.844128	-0.152564
5	6	0	-0.320672	-0.301943	-0.381383
6	6	0	2.000317	0.699719	-0.015205
7	6	0	1.326062	-2.249503	-0.575046
8	6	0	2.295607	-1.786986	0.507566
9	6	0	2.785067	-0.389236	0.269339
10	8	0	-2.443443	2.571569	-0.144670
11	8	0	2.799253	1.815610	-0.125142
12	6	0	4.125967	0.080494	0.327175
13	6	0	-0.038715	-1.597689	-0.615801
14	6	0	-1.117382	-2.576702	-0.995775
15	6	0	4.073043	1.415158	0.077358

16	8	0	-2.565557	-0.245244	0.600355
17	6	0	-3.850292	-0.540579	0.297885
18	8	0	-4.307129	-0.499828	-0.818867
19	6	0	-4.611327	-0.926181	1.534233
20	6	0	0.404620	3.426955	0.066138
21	8	0	3.373186	-2.724168	0.514326
22	1	0	-2.206016	0.095053	-1.408591
23	1	0	1.809437	-2.143482	-1.552515
24	1	0	1.183618	-3.320216	-0.425414
25	1	0	1.776932	-1.822893	1.472312
26	1	0	5.010302	-0.501780	0.514948
27	1	0	-2.066592	-2.113467	-1.245267
28	1	0	-1.286999	-3.289092	-0.183242
29	1	0	-0.790241	-3.165125	-1.856693
30	1	0	4.826874	2.178692	0.004830
31	1	0	-4.756329	-0.040037	2.153399
32	1	0	-4.047547	-1.649030	2.121483
33	1	0	-5.577722	-1.334896	1.256442
34	1	0	1.072079	3.507243	0.923927
35	1	0	0.964982	3.769112	-0.805607
36	1	0	-0.440856	4.097036	0.212437
37	1	0	3.841497	-2.651601	1.351971

Conformer # 4
Electronic Energy: -995.801467 Hartree
Gibbs Free Energy: -995.559069 Hartree

Center Number	Atomic Number	Atomic Type	Coordinates (Angstroms) X	Y	Z
1	6	0	1.603422	0.269058	0.411095
2	6	0	1.343455	1.774061	0.326111
3	6	0	-0.074978	1.978183	0.084934
4	6	0	-0.688545	0.755089	-0.063142
5	6	0	0.278758	-0.355814	0.033811
6	6	0	-2.104344	0.553627	-0.193530
7	6	0	-1.156164	-2.284947	-0.770743
8	6	0	-2.467105	-1.996262	-0.038368
9	6	0	-2.901059	-0.567093	-0.155887
10	8	0	2.210270	2.623833	0.441387
11	8	0	-2.907026	1.667636	-0.284250
12	6	0	-4.247291	-0.109462	-0.223642
13	6	0	0.100027	-1.671424	-0.187060
14	6	0	1.191178	-2.674263	0.069473
15	6	0	-4.190006	1.243470	-0.306040
16	8	0	2.653601	-0.060790	-0.519936
17	6	0	3.884815	-0.324177	-0.024504
18	8	0	4.130985	-0.414102	1.153827
19	6	0	4.879823	-0.493446	-1.136381
20	6	0	-0.645079	3.357082	0.050409

21	8	0	-2.429093	-2.429460	1.328316
22	1	0	1.929244	0.006756	1.417589
23	1	0	-1.269407	-1.962693	-1.810229
24	1	0	-1.029559	-3.366639	-0.795614
25	1	0	-3.239051	-2.617896	-0.492090
26	1	0	-5.136170	-0.716595	-0.219773
27	1	0	2.042659	-2.269587	0.606675
28	1	0	1.549890	-3.105408	-0.869998
29	1	0	0.784097	-3.503220	0.653078
30	1	0	-4.939926	2.009539	-0.390778
31	1	0	5.052214	0.473583	-1.610403
32	1	0	4.491626	-1.168100	-1.897630
33	1	0	5.814787	-0.872265	-0.735927
34	1	0	-1.373119	3.513814	0.847490
35	1	0	0.163016	4.076137	0.172392
36	1	0	-1.156844	3.561248	-0.890073
37	1	0	-1.794794	-1.877714	1.801329

Conformer # 5
Electronic Energy: -995.799931 Hartree
Gibbs Free Energy: -995.557537 Hartree

Center Number	Atomic Number	Atomic Type	Coordinates (Angstroms) X	Y	Z
1	6	0	-1.848682	0.064896	-0.957797
2	6	0	-1.823769	1.564994	-0.658770
3	6	0	-0.446008	1.947600	-0.397371
4	6	0	0.339603	0.821713	-0.359375
5	6	0	-0.428223	-0.394763	-0.693253
6	6	0	1.734174	0.797895	-0.028936
7	6	0	1.407293	-2.168824	-0.734399
8	6	0	2.221241	-1.672690	0.462260
9	6	0	2.572173	-0.219164	0.355844
10	8	0	-2.809510	2.282625	-0.664279
11	8	0	2.418823	1.990679	0.031961
12	6	0	3.824140	0.381011	0.667789
13	6	0	-0.006042	-1.655015	-0.904024
14	6	0	-0.934819	-2.725298	-1.414001
15	6	0	3.674141	1.712531	0.446381
16	8	0	-2.912158	-0.639176	-0.283601
17	6	0	-2.996276	-0.553293	1.061164
18	8	0	-2.215569	0.066422	1.740826
19	6	0	-4.179194	-1.326534	1.572245
20	6	0	-0.091814	3.375315	-0.143477
21	8	0	3.386234	-2.477613	0.638779
22	1	0	-2.103235	-0.060378	-2.010755
23	1	0	1.970304	-1.962370	-1.653154
24	1	0	1.363131	-3.254794	-0.645108
25	1	0	1.640226	-1.835521	1.373376
26	1	0	4.711151	-0.119790	1.012046

27	1	0	-1.914371	-2.358241	-1.700972
28	1	0	-1.073528	-3.500292	-0.654664
29	1	0	-0.481163	-3.220331	-2.276893
30	1	0	4.341616	2.551409	0.531331
31	1	0	-4.098333	-2.372069	1.275750
32	1	0	-5.094965	-0.931277	1.133123
33	1	0	-4.224907	-1.250530	2.653850
34	1	0	0.406092	3.503399	0.817978
35	1	0	0.583003	3.768164	-0.905198
36	1	0	-1.002313	3.972069	-0.148445
37	1	0	3.927106	-2.415185	-0.157413

Conformer # 6
Electronic Energy: -995.799814 Hartree
Gibbs Free Energy: -995.557349 Hartree

Center Number	Atomic Number	Atomic Type	Coordinates (Angstroms) X	Y	Z
1	6	0	-1.842407	0.100408	-0.957406
2	6	0	-1.808836	1.592824	-0.622542
3	6	0	-0.428479	1.960929	-0.356814
4	6	0	0.349791	0.829484	-0.342547
5	6	0	-0.426994	-0.376167	-0.695403
6	6	0	1.747914	0.791196	-0.030813
7	6	0	1.390103	-2.168369	-0.736070
8	6	0	2.189136	-1.672654	0.471909
9	6	0	2.577026	-0.236292	0.341956
10	8	0	-2.791054	2.315394	-0.607622
11	8	0	2.453967	1.972007	0.003401
12	6	0	3.848324	0.340608	0.612864
13	6	0	-0.014781	-1.637343	-0.921877
14	6	0	-0.949291	-2.689620	-1.458595
15	6	0	3.715630	1.673079	0.385544
16	8	0	-2.915485	-0.611311	-0.307200
17	6	0	-3.005673	-0.559260	1.038734
18	8	0	-2.220960	0.032538	1.738496
19	6	0	-4.202126	-1.328737	1.523515
20	6	0	-0.061126	3.380358	-0.076859
21	8	0	3.395650	-2.424376	0.613848
22	1	0	-2.090537	0.002478	-2.014815
23	1	0	1.975235	-1.976631	-1.641916
24	1	0	1.323176	-3.255029	-0.648646
25	1	0	1.575659	-1.795203	1.372655
26	1	0	4.735013	-0.177757	0.928771
27	1	0	-0.485105	-3.185364	-2.315411
28	1	0	-1.116997	-3.467803	-0.708289
29	1	0	-1.916626	-2.305276	-1.764070
30	1	0	4.400468	2.500160	0.443790
31	1	0	-4.151141	-2.360118	1.175810

32	1	0	-4.237579	-1.303057	2.607880
33	1	0	-5.111179	-0.889136	1.113111
34	1	0	-0.969079	3.979506	-0.032809
35	1	0	0.585366	3.792599	-0.853126
36	1	0	0.473501	3.481143	0.867779
37	1	0	3.163619	-3.317181	0.888329

Conformer # 7
Electronic Energy: -995.799729 Hartree
Gibbs Free Energy: -995.557645 Hartree

Center Number	Atomic Number	Atomic Type	Coordinates (Angstroms) X	Y	Z
1	6	0	1.611881	0.261299	0.414998
2	6	0	1.360110	1.768017	0.337254
3	6	0	-0.057414	1.980476	0.099769
4	6	0	-0.676653	0.760616	-0.054275
5	6	0	0.283128	-0.354544	0.039350
6	6	0	-2.093789	0.568872	-0.189751
7	6	0	-1.163591	-2.277359	-0.756788
8	6	0	-2.489595	-1.975836	-0.056849
9	6	0	-2.901089	-0.542999	-0.161044
10	8	0	2.232131	2.612828	0.453786
11	8	0	-2.885123	1.689834	-0.286878
12	6	0	-4.242419	-0.073346	-0.238361
13	6	0	0.092547	-1.668826	-0.170842
14	6	0	1.174114	-2.678375	0.097730
15	6	0	-4.172347	1.278958	-0.317997
16	8	0	2.657378	-0.070571	-0.522011
17	6	0	3.889704	-0.339623	-0.034343
18	8	0	4.143198	-0.433934	1.142246
19	6	0	4.877863	-0.509805	-1.152551
20	6	0	-0.619850	3.362919	0.073704
21	8	0	-2.463932	-2.323956	1.335609
22	1	0	1.939999	-0.006910	1.419084
23	1	0	-1.261820	-1.971482	-1.803215
24	1	0	-1.041492	-3.361336	-0.775153
25	1	0	-3.259493	-2.570779	-0.558171
26	1	0	-5.136777	-0.672393	-0.241569
27	1	0	2.035921	-2.271964	0.616681
28	1	0	1.517979	-3.139054	-0.833260
29	1	0	0.762632	-3.486334	0.707851
30	1	0	-4.914001	2.052563	-0.406164
31	1	0	5.051269	0.457743	-1.625124
32	1	0	4.482700	-1.180989	-1.913311
33	1	0	5.813707	-0.893281	-0.758604
34	1	0	-1.352686	3.516712	0.866852
35	1	0	0.191589	4.076119	0.207616
36	1	0	-1.123415	3.578997	-0.868612

37	1	0	-2.410931	-3.283889	1.403053

Conformer # 8
Electronic Energy: -995.799658 Hartree
Gibbs Free Energy: -995.556343 Hartree

Center Number	Atomic Number	Atomic Type	Coordinates (Angstroms) X	Y	Z
1	6	0	-1.645919	0.069666	-0.986166
2	6	0	-1.557421	1.584973	-0.785332
3	6	0	-0.209833	1.897507	-0.336890
4	6	0	0.478027	0.728053	-0.117085
5	6	0	-0.362768	-0.458149	-0.372601
6	6	0	1.873328	0.642642	0.216767
7	6	0	1.085478	-2.261837	0.656382
8	6	0	2.468584	-1.866688	0.138117
9	6	0	2.758645	-0.406287	0.302432
10	8	0	-2.466439	2.362299	-1.018434
11	8	0	2.557633	1.819429	0.416336
12	6	0	4.038093	0.163094	0.561426
13	6	0	-0.106152	-1.756642	-0.131839
14	6	0	-1.032820	-2.855864	-0.575520
15	6	0	3.855930	1.505441	0.626016
16	8	0	-2.889829	-0.486114	-0.522887
17	6	0	-3.260824	-0.255335	0.755763
18	8	0	-2.587887	0.362843	1.543431
19	6	0	-4.603456	-0.865755	1.041410
20	6	0	0.223068	3.318518	-0.192675
21	8	0	2.685913	-2.304671	-1.210987
22	1	0	-1.669088	-0.123841	-2.059863
23	1	0	1.003385	-1.927468	1.694793
24	1	0	1.045078	-3.350305	0.678299
25	1	0	3.206604	-2.421014	0.718101
26	1	0	4.965891	-0.367252	0.690808
27	1	0	-1.849874	-2.515975	-1.202171
28	1	0	-1.458999	-3.373419	0.289462
29	1	0	-0.456335	-3.604143	-1.125369
30	1	0	4.517989	2.331933	0.812893
31	1	0	-4.588326	-1.931538	0.815765
32	1	0	-5.356794	-0.405663	0.401818
33	1	0	-4.860879	-0.709293	2.083998
34	1	0	0.574438	3.534358	0.816385
35	1	0	1.040281	3.564675	-0.871983
36	1	0	-0.621327	3.968483	-0.415440
37	1	0	2.101056	-1.799853	-1.788411

Conformer # 9
Electronic Energy: -995.799430 Hartree

Gibbs Free Energy: -995.556974 Hartree

Center Number	Atomic Number	Atomic Type	Coordinates (Angstroms) X	Y	Z
1	6	0	-1.835145	0.123317	-0.957242
2	6	0	-1.799254	1.609971	-0.597093
3	6	0	-0.418339	1.969530	-0.322727
4	6	0	0.355196	0.835364	-0.322432
5	6	0	-0.423998	-0.363928	-0.690384
6	6	0	1.756183	0.788764	-0.025358
7	6	0	1.372844	-2.183555	-0.715547
8	6	0	2.165432	-1.681786	0.486955
9	6	0	2.578946	-0.248354	0.332381
10	8	0	-2.779358	2.335060	-0.572955
11	8	0	2.478309	1.960588	-0.021783
12	6	0	3.866344	0.313453	0.557656
13	6	0	-0.018497	-1.626541	-0.922930
14	6	0	-0.954579	-2.663978	-1.485658
15	6	0	3.746028	1.646531	0.324991
16	8	0	-2.916897	-0.594498	-0.329251
17	6	0	-3.018711	-0.563660	1.016427
18	8	0	-2.237360	0.013219	1.732415
19	6	0	-4.223634	-1.333774	1.478450
20	6	0	-0.043250	3.383229	-0.024868
21	8	0	3.298488	-2.542661	0.611784
22	1	0	-2.073264	0.044933	-2.018621
23	1	0	1.966533	-2.023263	-1.622432
24	1	0	1.288446	-3.264821	-0.600190
25	1	0	1.535914	-1.777653	1.378729
26	1	0	4.762423	-0.209295	0.840903
27	1	0	-1.905254	-2.263585	-1.821440
28	1	0	-1.158101	-3.437485	-0.739534
29	1	0	-0.472925	-3.169200	-2.326968
30	1	0	4.444900	2.463283	0.355374
31	1	0	-4.185978	-2.354518	1.099459
32	1	0	-5.127148	-0.870058	1.082293
33	1	0	-4.258947	-1.339793	2.563113
34	1	0	0.512458	3.466685	0.909192
35	1	0	0.585972	3.808961	-0.808231
36	1	0	-0.948969	3.982873	0.049297
37	1	0	3.655088	-2.458202	1.501654

(2*R*,8*S*)-*cis*-**14f**:

Functional / Basis Set = B3LYP / cc-pVTZ (with CPCM (Chloroform)) : 9 conformers at 1% or greater Boltzmann weight (Electronic Energy Weighted).

Conformer # 1
Electronic Energy: -995.801854 Hartree

Gibbs Free Energy: -995.559428 Hartree

Center Number	Atomic Number	Atomic Type	Coordinates (Angstroms) X	Y	Z
1	6	0	-1.698334	0.234722	-0.434398
2	6	0	-1.522266	1.741297	-0.234518
3	6	0	-0.110429	2.010779	-0.021726
4	6	0	0.578004	0.819272	0.004495
5	6	0	-0.327262	-0.336107	-0.152098
6	6	0	2.005343	0.690884	0.072693
7	6	0	1.252308	-2.224638	0.442332
8	6	0	2.504559	-1.809287	-0.334767
9	6	0	2.862533	-0.370085	-0.106158
10	8	0	-2.440490	2.543642	-0.253895
11	8	0	2.748227	1.838184	0.246592
12	6	0	4.182184	0.157918	-0.040649
13	6	0	-0.062497	-1.649907	-0.041631
14	6	0	-1.099158	-2.697947	-0.339956
15	6	0	4.050744	1.490937	0.180660
16	8	0	-2.691613	-0.231601	0.501749
17	6	0	-3.925115	-0.523757	0.029821
18	8	0	-4.214168	-0.529196	-1.142242
19	6	0	-4.863968	-0.842204	1.157974
20	6	0	0.381150	3.414324	0.106907
21	8	0	3.602035	-2.667153	-0.022717
22	1	0	-2.047175	0.033725	-1.447165
23	1	0	1.398213	-1.965996	1.496405
24	1	0	1.197477	-3.311012	0.391259
25	1	0	2.331123	-1.973374	-1.401305
26	1	0	5.099805	-0.393733	-0.138933
27	1	0	-0.668028	-3.452976	-1.001871
28	1	0	-1.395172	-3.221957	0.573727
29	1	0	-1.993149	-2.306659	-0.814311
30	1	0	4.760762	2.287553	0.314289
31	1	0	-5.781948	-1.264642	0.761722
32	1	0	-4.399874	-1.531942	1.860774
33	1	0	-5.092242	0.076574	1.699791
34	1	0	1.079166	3.672980	-0.690344
35	1	0	-0.469064	4.092322	0.058064
36	1	0	0.903904	3.576419	1.049718
37	1	0	3.797504	-2.585492	0.918395

Conformer # 2
Electronic Energy: -995.801640 Hartree
Gibbs Free Energy: -995.559484 Hartree

Center Number	Atomic Number	Atomic Type	Coordinates (Angstroms) X	Y	Z
1	6	0	-1.622682	0.370157	-0.503408

2	6	0	-1.309454	1.850502	-0.281632
3	6	0	0.131952	2.000752	-0.171585
4	6	0	0.722937	0.760541	-0.241178
5	6	0	-0.269799	-0.303718	-0.496238
6	6	0	2.122805	0.501615	-0.073846
7	6	0	1.233675	-2.334784	-0.867991
8	6	0	2.279770	-2.048132	0.207857
9	6	0	2.829935	-0.656676	0.140863
10	8	0	-2.157797	2.720846	-0.180181
11	8	0	2.985958	1.570739	-0.019437
12	6	0	4.186477	-0.272630	0.333971
13	6	0	-0.091296	-1.606106	-0.789405
14	6	0	-1.251611	-2.498305	-1.139065
15	6	0	4.222432	1.079548	0.220359
16	8	0	-2.455381	-0.089218	0.585644
17	6	0	-3.768935	-0.301797	0.339879
18	8	0	-4.270692	-0.226284	-0.755390
19	6	0	-4.496663	-0.646754	1.607686
20	6	0	0.740665	3.347027	0.041003
21	8	0	1.800784	-2.370651	1.520821
22	1	0	-2.166045	0.240006	-1.437166
23	1	0	1.028443	-3.405331	-0.834201
24	1	0	1.683014	-2.146110	-1.849262
25	1	0	3.105333	-2.741582	0.049621
26	1	0	5.021641	-0.923947	0.526675
27	1	0	-2.187558	-1.971250	-1.292445
28	1	0	-1.401761	-3.243061	-0.352095
29	1	0	-1.022619	-3.057103	-2.049990
30	1	0	5.015993	1.803126	0.275727
31	1	0	-5.503108	-0.978165	1.372626
32	1	0	-4.544114	0.237667	2.244135
33	1	0	-3.961503	-1.419159	2.157601
34	1	0	1.339783	3.382142	0.951295
35	1	0	1.398502	3.628479	-0.782154
36	1	0	-0.054606	4.086477	0.116797
37	1	0	1.064391	-1.783091	1.727559

Conformer # 3
Electronic Energy: -995.801497 Hartree
Gibbs Free Energy: -995.559284 Hartree

Center Number	Atomic Number	Atomic Type	Coordinates (Angstroms) X	Y	Z
1	6	0	-1.698738	0.226047	-0.442309
2	6	0	-1.523376	1.736642	-0.274582
3	6	0	-0.113552	2.010753	-0.058603
4	6	0	0.576799	0.820846	-0.010050
5	6	0	-0.325789	-0.338493	-0.156189
6	6	0	2.002498	0.696300	0.082873

7	6	0	1.262421	-2.220954	0.437147
8	6	0	2.502404	-1.787332	-0.351498
9	6	0	2.864736	-0.361606	-0.084488
10	8	0	-2.441949	2.538062	-0.316792
11	8	0	2.739959	1.841149	0.290499
12	6	0	4.181751	0.162090	0.028766
13	6	0	-0.059110	-1.651214	-0.034307
14	6	0	-1.098888	-2.701796	-0.313256
15	6	0	4.043843	1.492760	0.260773
16	8	0	-2.685062	-0.220307	0.511783
17	6	0	-3.920752	-0.527192	0.055941
18	8	0	-4.218207	-0.562733	-1.113613
19	6	0	-4.850657	-0.820605	1.198285
20	6	0	0.376666	3.416467	0.050659
21	8	0	3.635303	-2.579598	0.010879
22	1	0	-2.055420	0.002700	-1.447459
23	1	0	1.424042	-1.973185	1.489913
24	1	0	1.205439	-3.308460	0.380957
25	1	0	2.302489	-1.910158	-1.423092
26	1	0	5.099825	-0.391728	-0.042415
27	1	0	-0.676818	-3.460252	-0.977218
28	1	0	-1.381273	-3.221046	0.607487
29	1	0	-2.000140	-2.314668	-0.776992
30	1	0	4.749881	2.286764	0.427072
31	1	0	-5.772283	-1.250294	0.818634
32	1	0	-4.381221	-1.496265	1.911209
33	1	0	-5.073257	0.109356	1.723036
34	1	0	1.102031	3.654058	-0.728317
35	1	0	-0.469980	4.094318	-0.042538
36	1	0	0.867284	3.601518	1.006605
37	1	0	3.497516	-3.471531	-0.323914

Conformer # 4
Electronic Energy: -995.800810 Hartree
Gibbs Free Energy: -995.558641 Hartree

Center Number	Atomic Number	Atomic Type	Coordinates (Angstroms) X	Y	Z
1	6	0	-1.619739	0.378584	-0.489263
2	6	0	-1.306126	1.850729	-0.219044
3	6	0	0.135217	1.994905	-0.104830
4	6	0	0.721998	0.754797	-0.201434
5	6	0	-0.271002	-0.304334	-0.463584
6	6	0	2.127615	0.497857	-0.083389
7	6	0	1.207422	-2.374658	-0.767098
8	6	0	2.288293	-2.041821	0.251429
9	6	0	2.841426	-0.658346	0.110448
10	8	0	-2.153556	2.719149	-0.093157
11	8	0	2.996011	1.562955	-0.106936

12	6	0	4.210371	-0.278027	0.205647
13	6	0	-0.099929	-1.611289	-0.734564
14	6	0	-1.265115	-2.489900	-1.104844
15	6	0	4.244136	1.071900	0.063043
16	8	0	-2.490749	-0.103523	0.558197
17	6	0	-3.796888	-0.294839	0.265864
18	8	0	-4.264318	-0.183051	-0.841828
19	6	0	-4.567529	-0.667848	1.500176
20	6	0	0.749178	3.334412	0.134046
21	8	0	1.727936	-2.267524	1.553851
22	1	0	-2.134061	0.279569	-1.443638
23	1	0	0.973527	-3.433328	-0.642378
24	1	0	1.640821	-2.284656	-1.768893
25	1	0	3.110291	-2.745969	0.088392
26	1	0	5.054660	-0.929242	0.355344
27	1	0	-2.174669	-1.944870	-1.335665
28	1	0	-1.481300	-3.188989	-0.291655
29	1	0	-1.005454	-3.099285	-1.973960
30	1	0	5.041915	1.792794	0.052629
31	1	0	-5.569607	-0.981192	1.224950
32	1	0	-4.625480	0.198620	2.160012
33	1	0	-4.057410	-1.461329	2.043837
34	1	0	1.412543	3.329452	0.998746
35	1	0	1.342918	3.665150	-0.719718
36	1	0	-0.043323	4.061903	0.301221
37	1	0	2.396082	-2.055419	2.214136

Conformer # 5
Electronic Energy: -995.800714 Hartree
Gibbs Free Energy: -995.558489 Hartree

Center Number	Atomic Number	Atomic Type	Coordinates (Angstroms) X	Y	Z
1	6	0	-1.700917	0.213714	-0.451939
2	6	0	-1.528770	1.728010	-0.316715
3	6	0	-0.120693	2.009405	-0.099928
4	6	0	0.572655	0.822602	-0.030789
5	6	0	-0.323731	-0.342518	-0.168079
6	6	0	1.996712	0.704202	0.088687
7	6	0	1.273908	-2.232437	0.405366
8	6	0	2.502809	-1.776364	-0.377659
9	6	0	2.862921	-0.351416	-0.068602
10	8	0	-2.448535	2.526830	-0.378894
11	8	0	2.724623	1.845811	0.344548
12	6	0	4.174259	0.170932	0.105768
13	6	0	-0.051803	-1.654475	-0.044943
14	6	0	-1.095393	-2.706839	-0.303284
15	6	0	4.028217	1.497606	0.356512
16	8	0	-2.674682	-0.214248	0.524058

17	6	0	-3.915788	-0.531912	0.090766
18	8	0	-4.228958	-0.590310	-1.073744
19	6	0	-4.829064	-0.806065	1.251186
20	6	0	0.366428	3.417567	-0.009603
21	8	0	3.567186	-2.664976	-0.031017
22	1	0	-2.069333	-0.031942	-1.447410
23	1	0	1.435631	-2.013085	1.464894
24	1	0	1.228683	-3.316510	0.316031
25	1	0	2.289192	-1.868739	-1.448455
26	1	0	5.099990	-0.375060	0.069069
27	1	0	-0.686898	-3.462144	-0.979026
28	1	0	-1.352995	-3.229569	0.622730
29	1	0	-2.009049	-2.321531	-0.743248
30	1	0	4.728159	2.286999	0.565062
31	1	0	-5.758483	-1.236189	0.891604
32	1	0	-4.351315	-1.475551	1.964503
33	1	0	-5.038637	0.131071	1.768376
34	1	0	1.118930	3.635687	-0.768104
35	1	0	-0.476081	4.092819	-0.148208
36	1	0	0.822825	3.627062	0.958371
37	1	0	4.231807	-2.640258	-0.726253

Conformer # 6
Electronic Energy: -995.800068 Hartree
Gibbs Free Energy: -995.557489 Hartree

Center Number	Atomic Number	Atomic Type	Coordinates (Angstroms) X	Y	Z
1	6	0	-1.729024	0.376526	-1.000369
2	6	0	-1.482928	1.829510	-0.588875
3	6	0	-0.059301	1.991888	-0.344767
4	6	0	0.552673	0.764061	-0.403870
5	6	0	-0.387500	-0.301487	-0.806986
6	6	0	1.928352	0.510941	-0.088980
7	6	0	1.148322	-2.337371	-0.945024
8	6	0	2.000390	-2.020976	0.283764
9	6	0	2.591317	-0.645374	0.237873
10	8	0	-2.357620	2.673382	-0.493868
11	8	0	2.790474	1.574561	0.030761
12	6	0	3.922706	-0.268388	0.570664
13	6	0	-0.164339	-1.597508	-1.096369
14	6	0	-1.251035	-2.479032	-1.652430
15	6	0	3.987639	1.079416	0.421175
16	8	0	-2.869868	-0.212261	-0.341221
17	6	0	-2.867364	-0.295124	1.005518
18	8	0	-1.945175	0.068526	1.695282
19	6	0	-4.150050	-0.894612	1.507900
20	6	0	0.504194	3.329484	0.003569
21	8	0	1.286414	-2.259480	1.504113

22	1	0	-2.021574	0.369840	-2.050544
23	1	0	0.925299	-3.404917	-0.916526
24	1	0	1.754939	-2.181205	-1.843854
25	1	0	2.819715	-2.738867	0.308513
26	1	0	4.723016	-0.920118	0.876728
27	1	0	-2.152280	-1.944408	-1.933418
28	1	0	-1.525565	-3.245302	-0.921718
29	1	0	-0.874485	-3.012081	-2.529295
30	1	0	4.779013	1.797318	0.542100
31	1	0	-4.117791	-0.970700	2.589963
32	1	0	-4.299114	-1.880909	1.069245
33	1	0	-4.990520	-0.270610	1.204552
34	1	0	1.032870	3.309923	0.956967
35	1	0	1.215064	3.678151	-0.746729
36	1	0	-0.309033	4.050195	0.069237
37	1	0	0.542338	-1.645856	1.547491

Conformer # 7
Electronic Energy: -995.799956 Hartree
Gibbs Free Energy: -995.556706 Hartree

Center Number	Atomic Number	Atomic Type	Coordinates (Angstroms) X	Y	Z
1	6	0	-1.754277	0.074916	-0.997312
2	6	0	-1.744746	1.570741	-0.669709
3	6	0	-0.396536	1.929439	-0.260869
4	6	0	0.371366	0.792421	-0.179901
5	6	0	-0.410270	-0.419074	-0.498519
6	6	0	1.783324	0.766802	0.080600
7	6	0	1.211597	-2.201138	0.274314
8	6	0	2.525776	-1.673954	-0.307947
9	6	0	2.736977	-0.220593	-0.001071
10	8	0	-2.709299	2.305778	-0.791735
11	8	0	2.403292	1.959249	0.383375
12	6	0	3.990418	0.401443	0.259866
13	6	0	-0.060219	-1.712327	-0.388043
14	6	0	-0.943060	-2.830937	-0.871627
15	6	0	3.726340	1.712387	0.491837
16	8	0	-2.935290	-0.597158	-0.521588
17	6	0	-3.249734	-0.498496	0.788556
18	8	0	-2.573777	0.093073	1.593941
19	6	0	-4.536988	-1.214426	1.084817
20	6	0	-0.040907	3.357515	-0.011778
21	8	0	3.630887	-2.460059	0.139127
22	1	0	-1.827643	-0.028504	-2.081108
23	1	0	1.247827	-3.286896	0.197828
24	1	0	1.183911	-1.960428	1.342142
25	1	0	2.516680	-1.818068	-1.391371
26	1	0	4.953057	-0.077117	0.277879

27	1	0	-1.817671	-2.494597	-1.417286
28	1	0	-1.281210	-3.445491	-0.031572
29	1	0	-0.360934	-3.492473	-1.518579
30	1	0	4.346426	2.554786	0.741551
31	1	0	-4.735835	-1.181209	2.151193
32	1	0	-4.481062	-2.248219	0.745899
33	1	0	-5.352586	-0.735097	0.543009
34	1	0	0.735832	3.707629	-0.692814
35	1	0	-0.928622	3.972018	-0.151260
36	1	0	0.333915	3.510105	1.000557
37	1	0	3.687045	-2.391819	1.099682

Conformer # 8
Electronic Energy: -995.799843 Hartree
Gibbs Free Energy: -995.557993 Hartree

Center Number	Atomic Number	Atomic Type	Coordinates (Angstroms) X	Y	Z
1	6	0	-1.623359	0.375913	-0.492777
2	6	0	-1.311577	1.850130	-0.232084
3	6	0	0.129459	1.997257	-0.117684
4	6	0	0.718989	0.758012	-0.210515
5	6	0	-0.272628	-0.302436	-0.472737
6	6	0	2.124351	0.503267	-0.080471
7	6	0	1.219106	-2.353143	-0.798610
8	6	0	2.298518	-2.034582	0.235599
9	6	0	2.842068	-0.648661	0.121037
10	8	0	-2.160640	2.717738	-0.111755
11	8	0	2.987046	1.573115	-0.085780
12	6	0	4.205594	-0.261785	0.245079
13	6	0	-0.096568	-1.606943	-0.750706
14	6	0	-1.258762	-2.492704	-1.113282
15	6	0	4.234738	1.088148	0.106157
16	8	0	-2.484494	-0.102947	0.564809
17	6	0	-3.792214	-0.300849	0.283948
18	8	0	-4.268068	-0.201419	-0.821339
19	6	0	-4.553099	-0.662969	1.527495
20	6	0	0.739353	3.339078	0.118948
21	8	0	1.828384	-2.205208	1.581303
22	1	0	-2.145100	0.269656	-1.442175
23	1	0	0.997172	-3.419751	-0.712955
24	1	0	1.656288	-2.232225	-1.796615
25	1	0	3.125717	-2.727941	0.055374
26	1	0	5.049213	-0.909167	0.412517
27	1	0	-1.007460	-3.089294	-1.993729
28	1	0	-1.455873	-3.203646	-0.305296
29	1	0	-2.177397	-1.954352	-1.322704
30	1	0	5.028418	1.813560	0.113018
31	1	0	-4.619790	0.214317	2.172127

32	1	0	-4.031076	-1.440135	2.083141
33	1	0	-5.552346	-0.993153	1.261755
34	1	0	1.386698	3.342153	0.995798
35	1	0	1.348848	3.661320	-0.726694
36	1	0	-0.056023	4.067838	0.265501
37	1	0	1.611085	-3.135796	1.706645

Conformer # 9
Electronic Energy: -995.799458 Hartree
Gibbs Free Energy: -995.556487 Hartree

Center Number	Atomic Number	Atomic Type	Coordinates (Angstroms) X	Y	Z
1	6	0	-1.755802	0.048152	-0.998732
2	6	0	-1.752154	1.550338	-0.701512
3	6	0	-0.407291	1.922182	-0.295624
4	6	0	0.366640	0.790834	-0.193315
5	6	0	-0.407156	-0.430142	-0.496510
6	6	0	1.774479	0.777168	0.086557
7	6	0	1.234757	-2.194370	0.277683
8	6	0	2.533450	-1.643894	-0.319566
9	6	0	2.736671	-0.201928	0.018029
10	8	0	-2.719568	2.278810	-0.841247
11	8	0	2.379188	1.970667	0.414060
12	6	0	3.978930	0.422482	0.317966
13	6	0	-0.048801	-1.720262	-0.372675
14	6	0	-0.930874	-2.848742	-0.834764
15	6	0	3.700706	1.729773	0.554513
16	8	0	-2.931747	-0.619598	-0.503301
17	6	0	-3.241766	-0.494360	0.805614
18	8	0	-2.566268	0.118148	1.595481
19	6	0	-4.523952	-1.210791	1.122346
20	6	0	-0.059177	3.355765	-0.068167
21	8	0	3.665774	-2.354339	0.187296
22	1	0	-1.834500	-0.078320	-2.079672
23	1	0	1.223503	-1.961403	1.345885
24	1	0	1.275899	-3.281220	0.197533
25	1	0	2.496563	-1.754551	-1.410464
26	1	0	4.941203	-0.053753	0.361623
27	1	0	-0.355035	-3.510008	-1.487806
28	1	0	-1.249407	-3.460589	0.014936
29	1	0	-1.817712	-2.523429	-1.366998
30	1	0	4.308864	2.573019	0.829442
31	1	0	-4.719210	-1.154742	2.188433
32	1	0	-4.463179	-2.251654	0.806652
33	1	0	-5.344221	-0.748482	0.572865
34	1	0	0.735863	3.692082	-0.734700
35	1	0	-0.943301	3.966192	-0.243721
36	1	0	0.287575	3.530397	0.950751

```
    37          1          0       3.646104   -3.246283   -0.174130
 ---------------------------------------------------------------------
```

CompareVOA results (based on Electronic Energy-weighted Boltzmann distributions)

(2*R*,8*R*)-**14g**:

Comparison to (2*R*,8*R*) calculated

Uniform Scaling Factor: 0.985

TNS (S_{fg}) IR = 88.3

TNS (S_{fg}) VCD = 83.7

SNS ((2*R*,8*R*) config) VCD = 85.0

SNS ((2*S*,8*S*) config) VCD = 2.3

ESI (Enantiomeric Similarity Index = SNS (2*R*,8*R*) – SNS (2*S*,8*S*) = 82.8

Confidence Level = 100

Comparison to (2*R*,8*S*) calculated

Uniform scaling factor 0.984

TNS (S_{fg}) IR = 88.2

TNS (S_{fg}) VCD = 61.0

SNS ((2*R*,8*S*) config) VCD = 70.3

SNS ((2*S*,8*R*) config) VCD = 9.1

ESI (Enantiomeric Similarity Index = SNS (2*R*,8*S*) – SNS (2*S*,8*R*) = 61.2

Confidence Level = 98 (Irrelevant as the S_{fg} values are considerably lower than for (2*R*,8*R*))

(2*S*,8*R*)-**14g**:

Comparison to (2*S*,8*R*) calculated

Uniform Scaling Factor: 0.986

TNS (S_{fg}) IR = 88.7

TNS (S_{fg}) VCD = 67.2

SNS ((2*S*,8*R*) config) VCD = 74.9

SNS ((2*R*,8*S*) config) VCD = 8.7

ESI (Enantiomeric Similarity Index = SNS (2*S*,8*R*) – SNS (2*R*,8*S*) = 66.2

Confidence Level = 99

*Comparison to (2*S*,8*S*) calculated

Uniform scaling factor 0.985

TNS (S_{fg}) IR = 88.5

TNS (S_{fg}) VCD = 56.1

SNS ((2*S*,8*S*) config) VCD = 68.5

SNS ((2*R*,8*R*) config) VCD = 13.5

ESI (Enantiomeric Similarity Index = SNS (2*S*,8*S*) – SNS (2*R*,8*R*) = 54.9

Confidence Level = 93 (Irrelevant as the S_{fg} values are lower than for (2*S*,8*R*))

*It should be noted that there was one particularly large VCD couplet band which was dominant in the calculated and experimental spectra and had the same sign in both (2*S*,8*R*) and (2*S*,8*S*) calculated spectra (likely coming from C=O alpha to the chiral center) – this caused relatively high values for comparison to the wrong diastereomer in the case of (2*S*,8*R*)-**14g**, however, the rest of the spectrum was more obviously in disagreement as seen in the plot.

CompareVOA results (based on Gibbs free energy-weighted Boltzmann distributions)

(2*R*,8*R*)-**14g**:

Comparison to (2*R*,8*R*) calculated

Uniform Scaling Factor: 0.985

TNS (S_{fg}) IR = 88.2

TNS (S_{fg}) VCD = 81.9

SNS ((2*R*,8*R*) config) VCD = 83.8

SNS ((2*S*,8*S*) config) VCD = 2.6

ESI (Enantiomeric Similarity Index = SNS (2*R*,8*R*) – SNS (2*S*,8*S*) = 81.2

Confidence Level = 100

Comparison to (2*R*,8*S*) calculated

Uniform scaling factor 0.984

TNS (S_{fg}) IR = 88.2

TNS (S_{fg}) VCD = 61.5

SNS ((2*R*,8*S*) config) VCD = 71.0

SNS ((2*S*,8*R*) config) VCD = 9.1

ESI (Enantiomeric Similarity Index = SNS (2*R*,8*S*) – SNS (2*S*,8*R*) = 61.9

Confidence Level = 99 (Irrelevant as the S_{fg} values are considerably lower than for (2*R*,8*R*))

(2*S*,8*R*)-**14g**:

Comparison to (2*S*,8*R*) calculated

Uniform Scaling Factor: 0.986

TNS (S_{fg}) IR = 88.6

TNS (S_{fg}) VCD = 65.8

SNS ((2*S*,8*R*) config) VCD = 74.8

SNS ((2*R*,8*S*) config) VCD = 9.5

ESI (Enantiomeric Similarity Index = SNS (2*S*,8*R*) – SNS (2*R*,8*S*) = 65.3

Confidence Level = 99

*Comparison to (2*S*,8*S*) calculated

Uniform scaling factor 0.985

TNS (S_{fg}) IR = 88.3

TNS (S_{fg}) VCD = 56.3

SNS ((2*S*,8*S*) config) VCD = 68.7

SNS ((2*R*,8*R*) config) VCD = 13.2

ESI (Enantiomeric Similarity Index = SNS (2*S*,8*S*) – SNS (2*R*,8*R*) = 55.6

Confidence Level = 94 (Irrelevant as the S_{fg} values are lower than for (2*S*,8*R*))

*It should be noted that there was one particularly large VCD couplet band which was dominant in the calculated and experimental spectra and had the same sign in both (2*S*,8*R*) and (2*S*,8*S*) calculated spectra (likely coming from C=O alpha to the chiral center) – this caused relatively high values for comparison to the wrong diastereomer in the case of (2*S*,8*R*)-**14g**, however, the rest of the spectrum was more obviously in disagreement as seen in the plot.

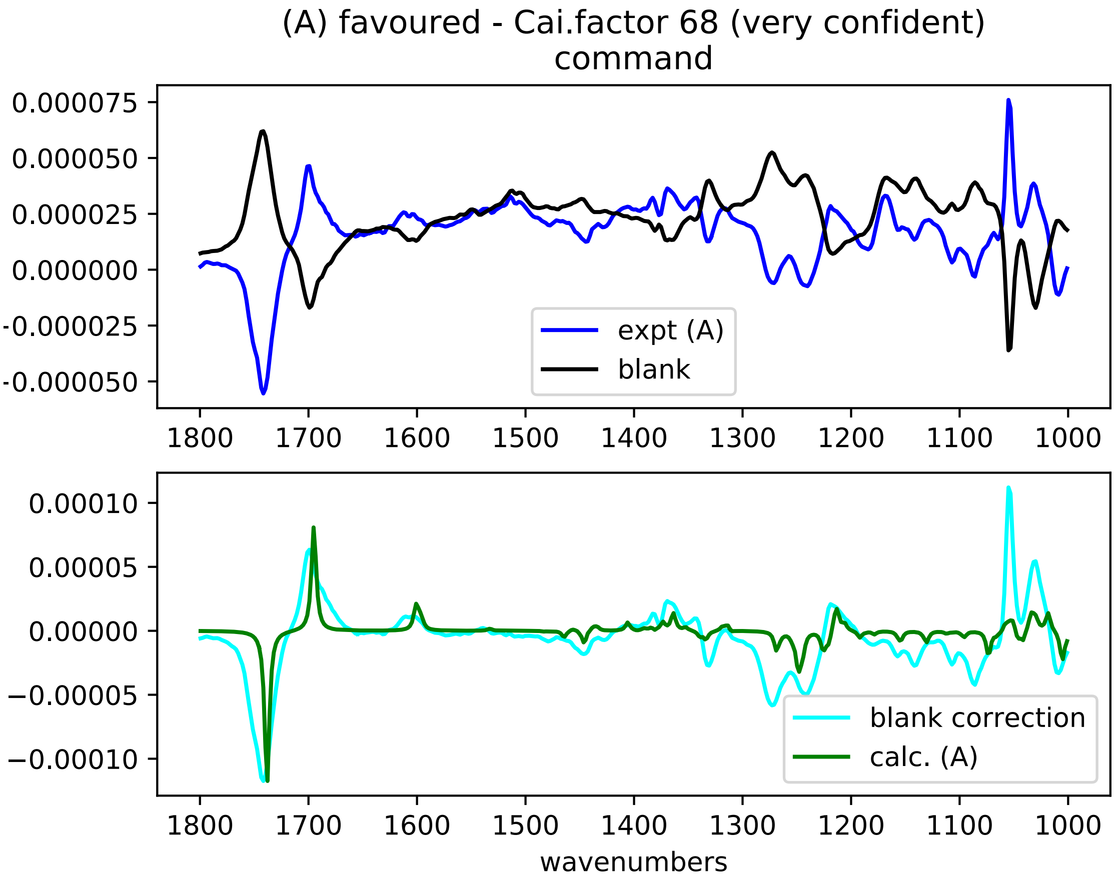

Figure S21. Cai.factor for (2*R*,8*R*)-**14g**

##

Cai• VCD analysis

University of Cambridge, 2020

##

command.txt (2*R*,8*R*)-**14g**

minimum wavenumber: 1000.0

maximum wavenumber: 1800.0

Lorentzian broadening: 5.0

Temperature for Boltzmann averaging (Kelvin): 298.0

Defined scaling factor: 0.982

Print out graphs: True

Boltzmann Analysis: True

Boltzmann Analysis Energy cut-off: 5.0

Unique calculated structure criteria: Energy: 0.0001 Frequency: 2.0

Extreme scale factor warning range: 0.01

Insufficient information criterion for Cai.factor: 10

Printing spectra in .csv file

Printing scaling factor analysis in .csv file

Printing two-line summary in .csv file

Experimental data for single enantiomer:

(A) /ihome/pliu/ltj6/bin/caifactor_RR14g/experimental/fh-03-174-b_VCD_Raw-RR-USE-AS-EXPERIMENT.csv

Blank file:

/ihome/pliu/ltj6/bin/caifactor_RR14g/experimental/fh-03-143-bb_VCD_Raw-SS-USE-AS-BLANK.csv

Calculation files:

/ihome/pliu/ltj6/bin/caifactor_RR14g/experimental/FHRR14g

Only one filename listed for calculations

which is a directory containing the following files:

/ihome/pliu/ltj6/bin/caifactor_RR14g/experimental/FHRR14g/fatemeh2rr-a_c4

/ihome/pliu/ltj6/bin/caifactor_RR14g/experimental/FHRR14g/fatemeh2rr-a_c1

/ihome/pliu/ltj6/bin/caifactor_RR14g/experimental/FHRR14g/fatemeh2rr-a_c5

/ihome/pliu/ltj6/bin/caifactor_RR14g/experimental/FHRR14g/fatemeh2rr-a_c9

/ihome/pliu/ltj6/bin/caifactor_RR14g/experimental/FHRR14g/fatemeh2rr-a_c10

/ihome/pliu/ltj6/bin/caifactor_RR14g/experimental/FHRR14g/fatemeh2rr-a_c2

/ihome/pliu/ltj6/bin/caifactor_RR14g/experimental/FHRR14g/fatemeh2rr-a_c6

/ihome/pliu/ltj6/bin/caifactor_RR14g/experimental/FHRR14g/fatemeh2rr-a_c8

/ihome/pliu/ltj6/bin/caifactor_RR14g/experimental/FHRR14g/fatemeh2rr-a_c3

/ihome/pliu/ltj6/bin/caifactor_RR14g/experimental/FHRR14g/fatemeh2rr-a_c7

Number of files: 10

Calculated data in Gaussian file

6 files rejected by energy cutoff

0 duplicate files removed

Unique Calculated Structures

Energy: -995.559958 hartrees, 0.000 kJ/mol, Boltzmann Factor: 1.000

/ihome/pliu/ltj6/bin/caifactor_RR14g/experimental/FHRR14g/fatemeh2rr-a_c5

Energy: -995.559822 hartrees, 0.357 kJ/mol, Boltzmann Factor: 0.866

/ihome/pliu/ltj6/bin/caifactor_RR14g/experimental/FHRR14g/fatemeh2rr-a_c1

Energy: -995.559725 hartrees, 0.612 kJ/mol, Boltzmann Factor: 0.781

/ihome/pliu/ltj6/bin/caifactor_RR14g/experimental/FHRR14g/fatemeh2rr-a_c3

Energy: -995.559069 hartrees, 2.334 kJ/mol, Boltzmann Factor: 0.390

/ihome/pliu/ltj6/bin/caifactor_RR14g/experimental/FHRR14g/fatemeh2rr-a_c2

Using all 4 unique conformations within energy cut-off

Defined scaling factor: 0.982

Single enantiomer result: Defined SF : 0.982 ; File (A) is assigned to the enantiomer calculated with Cai.factor 36

Single enantiomer blank: Defined SF : 0.982 ; File (A) is assigned to the enantiomer calculated with Cai.factor 65

Single enantiomer result: Opt. SF : 0.984 ; File (A) is assigned to the enantiomer calculated with Cai.factor 39

Single enantiomer blank: Opt. SF : 0.984 ; File (A) is assigned to the enantiomer calculated with Cai.factor 70

Average result over scaling factor range: 0.972 0.992

Experimental data for single enantiomer:

(A) /ihome/pliu/ltj6/bin/caifactor_RR14g/experimental/fh-03-174-b_VCD_Raw-RR-USE-AS-EXPERIMENT.csv

Blank file:

/ihome/pliu/ltj6/bin/caifactor_RR14g/experimental/fh-03-143-bb_VCD_Raw-SS-USE-AS-BLANK.csv

Single enantiomer summary

This is based on File (A) (fairly confident)

Using the blank data, File (A) is very confident

Optimising the scale factor increases the confidence level: very confident

Overall Cai.factor is 68 which means very confident assignment

Based on total confidence measure 10

Assignment of relative configuration of 14a using ^{13}C NMR

NMR workflow for computed ^{13}C NMR chemical shifts

SPARTAN'24 CONFORMATION SEARCH: (x86/macOS) build 1.1.0int23e

Multi-step conformation method:

1) OPT MMFF94x

..prune: window < 40.00 kJ/mol, at most 200

2) quick OPT HF/3-21G(*) TOLE=2.3e-04 TOLG=8.0e-04

..prune: window < 30.00 kJ/mol, at most 100

3) ENERGY WB97X-D/6-31G*

..prune: window < 15.00 kJ/mol, at most 50

4) OPT WB97X-D/6-31G* TOLE=7.6e-05 TOLG=2.0e-01

..prune: window < 10.00 kJ/mol, at most 50

5) OPT WB97X-D/6-31G* REUSERIC

..prune: window < 10.00 kJ/mol, at most 50

6) ENERGY WB97X-V/6-311+G(2DF,2P)[6-311G*]

..prune: window < 10.00 kJ/mol, at most 30

7) ENERGY WB97X-D/6-31G* NMR

Final Energy (Gibbs Free Energy) for *trans*-14a

-1522.113865128 (au/mol) from WB97X-V/6-311+G(2DF,2P)[6-311G*

of -1522.114713000 (au) + 2.226 (kJ/mol) from 10 conformers

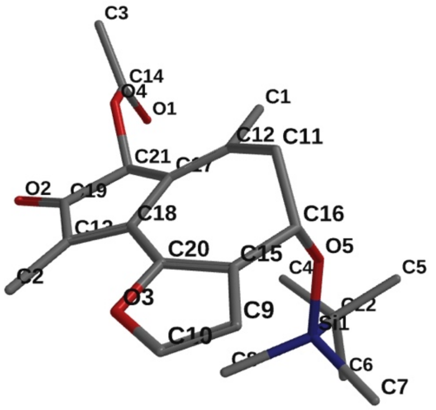

Figure S22. *Trans*-**14a** with atom labels

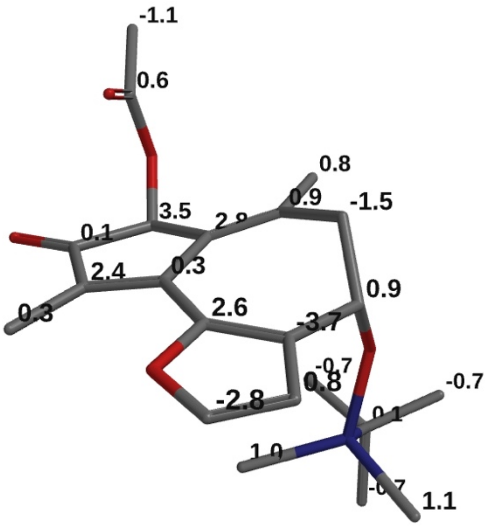

Figure S23. Calculated (*trans*-14a) minus experimental (major isomer) chemical shifts

Table S3. Computed NMR chemical shifts for *trans*-14a

position	δ C	mult	δ H	splitting (Hz)	HMBC (H→C) (2,3-bond)

**1**	26.0	CH_3	2.01	s	11, 12, 17
**2**	10.4	CH_3	2.11	s	13, 18, 19
**3**	19.9	CH_3	1.95	s	14
**4**	25.2	CH_3	0.95	s	5, 6, 22
**5**	25.2	CH_3	0.95	s	4, 6, 22
**6**	25.2	CH_3	0.95	s	4, 5, 22
**7**	-3.6	CH_3	0.00	s	
**8**	-3.6	CH_3	-0.37	s	
**9**	112.7	CH	6.51	d (1.2)	10, 15, 16, 20
**10**	142.2	CH	7.38	d (1.2)	9, 15, 20
**11**	43.5	CH_2	2.64	dd (27.8, 1.1)	1, 12, 15, 16, 17
**.**			2.78	dd (27.8, 7.8)	1, 12, 15, 16, 17
**12**	135.2	C			...
**13**	135.1	C			...
**14**	170.5	C			...
**15**	133.3	C			...
**16**	66.4	CH	5.32	dd (7.8, 1.1)	9, 11, 12, 15, 20

**17**	130.5	C			...
**18**	149.4	C			...
**19**	199.7	C			...
**20**	146.8	C			...
**21**	74.8	CH	5.24	s	12, 13, 14, 17, 18, 19
**22**	18.5	C			...

DP4 Analysis for major isomer

Comparison of computed chemical shifts for *trans*-14a to experimental chemical shifts for major isomer:

DP4 Score 100% using the Boltzmann average

RMS: 1.72

Maximum Absolute: 3.66

Mean absolute: 1.34

Cartesian Coordinates for *trans*-14a

Conformer 1 (-954881.27 kcal/mol)

Atom	x	y	z
C	1.130529	-1.138204	-1.040115
C	1.425915	0.167331	-0.940433
C	1.854424	0.944390	0.244419
C	1.752798	0.434111	1.594627
C	1.165154	-0.688238	2.114600
C	0.515793	-1.801487	1.347589

H	0.510769	-2.687844	1.997059
C	1.307210	-2.141527	0.081889
H	2.367789	-2.246777	0.340585
H	0.966799	-3.118431	-0.275402
C	1.368050	1.159421	-2.085210
H	0.330854	1.425406	-2.314443
C	2.066325	2.405945	-1.528135
O	2.368862	3.378447	-2.183543
C	2.228125	2.213634	-0.080206
O	2.187734	1.230118	2.611882
C	1.252337	-0.564623	3.533987
H	0.897007	-1.272409	4.269991
C	1.881401	0.614553	3.771495
H	2.185305	1.127715	4.670703
C	2.727199	3.329651	0.776493
H	2.957869	4.185322	0.137263
H	3.630866	3.043202	1.322434
H	1.981841	3.630191	1.518710
O	-0.813852	-1.533568	0.961981
Si	-1.978245	-0.463897	1.511843
C	-3.295356	-0.560019	0.149755
C	-2.744767	0.106956	-1.124420
H	-2.562460	1.178210	-0.974266
H	-3.463992	0.008376	-1.949684
H	-1.802291	-0.354183	-1.443269
C	-3.635801	-2.027156	-0.156915
H	-4.401997	-2.084805	-0.943327
H	-4.031368	-2.545630	0.725568
H	-2.753349	-2.577589	-0.500316
C	-4.572594	0.172614	0.594981
H	-5.322446	0.153124	-0.207721
H	-4.382307	1.225691	0.834855
H	-5.025059	-0.297307	1.476489
C	-2.631769	-1.049670	3.174287
H	-3.425044	-0.390458	3.545494
H	-1.831149	-1.051795	3.922302
H	-3.037377	-2.065361	3.110465
C	-1.377497	1.312454	1.674464
H	-0.795627	1.625235	0.801863
H	-0.760382	1.463438	2.565514
H	-2.243577	1.980859	1.753571
C	0.595499	-1.747500	-2.310199
H	1.301452	-2.488824	-2.708421
H	0.392728	-1.021021	-3.094971
H	-0.332471	-2.285005	-2.079240
O	1.897417	0.743580	-3.338898

C	3.218378	0.469770	-3.374872
O	3.942316	0.557430	-2.415451
C	3.632508	0.051026	-4.759434
H	3.497164	0.888622	-5.450844
H	3.003984	-0.771573	-5.109152
H	4.678379	-0.251920	-4.748271

Conformer 2 (-954881.36 kcal/mol)

Atom	x	y	z
C	-1.208437	1.230042	-0.993128
C	-1.540179	-0.067311	-0.891213
C	-2.011625	-0.830319	0.286073
C	-1.975082	-0.299579	1.630778
C	-1.385146	0.816184	2.162233
C	-0.665098	1.897343	1.411325
H	-0.655765	2.790380	2.051906
C	-1.393427	2.251582	0.110935
H	-2.457937	2.399489	0.328492
H	-1.004892	3.211814	-0.241174
C	-1.476660	-1.052399	-2.034223
H	-0.448671	-1.270225	-2.344395
C	-2.124043	-2.319373	-1.471594
O	-2.390575	-3.304464	-2.126095
C	-2.350975	-2.112250	-0.034421
O	-2.484649	-1.066114	2.635953
C	-1.549548	0.720372	3.575776
H	-1.212111	1.430624	4.317624
C	-2.222990	-0.437505	3.799137
H	-2.588682	-0.924912	4.689344
C	-2.848443	-3.228791	0.823813
H	-3.778916	-2.963431	1.334751
H	-2.119310	-3.490362	1.597453
H	-3.026755	-4.103418	0.193495
O	0.665769	1.577820	1.080702
Si	1.786600	0.487066	1.681250
C	3.126293	0.499135	0.340278
C	2.580836	-0.200612	-0.917562
H	2.364466	-1.258657	-0.731517
H	3.316810	-0.154732	-1.731363
H	1.658669	0.272861	-1.274048
C	3.508890	1.944955	-0.013989
H	4.284880	1.953426	-0.792913
H	3.907814	2.484761	0.854355
H	2.644711	2.504958	-0.388025
C	4.375751	-0.247475	0.837471

H	5.140570	-0.277707	0.048299
H	4.154711	-1.286455	1.113800
H	4.823445	0.245014	1.709282
C	2.426165	1.099822	3.339800
H	3.174787	0.412646	3.749768
H	1.608454	1.167407	4.069660
H	2.882754	2.092085	3.255511
C	1.125357	-1.262935	1.892293
H	0.573683	-1.599537	1.009091
H	0.468409	-1.358075	2.762964
H	1.968177	-1.950029	2.037849
C	-0.614930	1.796089	-2.256623
H	-1.326722	2.466842	-2.756401
H	-0.295501	1.037982	-2.972668
H	0.263743	2.397390	-1.994233
O	-2.231835	-0.635419	-3.169889
C	-1.645591	-0.752781	-4.379639
O	-0.484285	-1.037645	-4.543765
C	-2.639749	-0.468594	-5.472345
H	-3.200036	0.444692	-5.253645
H	-3.355896	-1.293317	-5.521372
H	-2.117284	-0.378360	-6.425225

Conformer 3 (-954879.68 kcal/mol)

Atom	x	y	z
C	-1.444267	-0.040008	-2.030593
C	-1.147908	1.227451	-2.368094
C	-0.922924	2.421282	-1.520085
C	-0.942371	2.362078	-0.079126
C	-0.736295	1.317508	0.776134
C	-0.457608	-0.097150	0.366764
H	0.525663	-0.129439	-0.130352
C	-1.493647	-0.615644	-0.630624
H	-1.337966	-1.698349	-0.701596
H	-2.495175	-0.479235	-0.200224
C	-1.099493	1.722764	-3.800462
H	-2.095161	1.697376	-4.254836
C	-0.673817	3.191296	-3.678435
O	-0.379104	3.903176	-4.614871
C	-0.705548	3.545656	-2.255360
O	-1.123669	3.511831	0.633088
C	-0.803333	1.855816	2.093377
H	-0.696587	1.311465	3.019358
C	-1.042336	3.184376	1.938726
H	-1.183319	3.996335	2.635034

C	-0.433039	4.943629	-1.808769
H	0.332987	4.972458	-1.028779
H	-1.331298	5.416681	-1.399415
H	-0.093133	5.527585	-2.667998
O	-0.472602	-0.934638	1.501903
Si	0.926227	-1.633085	2.116027
C	0.352996	-2.530434	3.679890
C	-0.277990	-1.519435	4.652957
H	0.437357	-0.746021	4.957869
H	-0.616669	-2.029420	5.565622
H	-1.146979	-1.024121	4.205084
C	-0.694170	-3.596120	3.314042
H	-1.046708	-4.110181	4.219889
H	-0.279219	-4.359933	2.644987
H	-1.564517	-3.149660	2.819789
C	1.557227	-3.208377	4.356557
H	1.236123	-3.736521	5.265224
H	2.322247	-2.481272	4.654277
H	2.031588	-3.948112	3.699918
C	1.641750	-2.814556	0.839597
H	2.543072	-3.309547	1.218423
H	1.924774	-2.280021	-0.074616
H	0.920730	-3.590898	0.563030
C	2.187211	-0.289855	2.491931
H	1.820110	0.410781	3.249227
H	2.421088	0.293834	1.593409
H	3.126962	-0.720997	2.856197
C	-1.772119	-1.074529	-3.079803
H	-0.952967	-1.801422	-3.164895
H	-1.948011	-0.656788	-4.071679
H	-2.662527	-1.638699	-2.775522
O	-0.293847	0.982955	-4.715274
C	1.028175	0.928731	-4.454798
O	1.539362	1.438441	-3.489693
C	1.746075	0.158346	-5.530042
H	2.797262	0.055870	-5.261832
H	1.657174	0.693736	-6.479890
H	1.290166	-0.826990	-5.660667

Conformer 4 (-954880.76 kcal/mol)

Atom	x	y	z
C	1.335782	-0.817640	-1.560283
C	1.108738	0.493760	-1.383095
C	1.221428	1.312517	-0.156054
C	1.464990	0.739092	1.148913

C	1.411040	-0.546280	1.617032
C	1.132354	-1.779659	0.808546
H	1.482884	-2.641347	1.394817
C	1.907281	-1.754177	-0.511823
H	2.954515	-1.501335	-0.303845
H	1.900703	-2.768827	-0.922791
C	0.599504	1.432717	-2.459411
H	-0.453572	1.230661	-2.682530
C	0.686555	2.821628	-1.815539
O	0.488458	3.866111	-2.397206
C	0.980440	2.632329	-0.388957
O	1.724462	1.589010	2.183031
C	1.669773	-0.471125	3.018409
H	1.720023	-1.296974	3.713825
C	1.849902	0.844477	3.299727
H	2.075164	1.387888	4.203859
C	0.969158	3.798162	0.543613
H	1.955974	3.967906	0.984434
H	0.265129	3.645374	1.368204
H	0.677293	4.691501	-0.015218
O	-0.228914	-1.962335	0.490004
Si	-1.621085	-1.993344	1.420205
C	-2.524297	-0.316206	1.342492
C	-1.935455	0.704152	2.331382
H	-2.003795	0.355237	3.368561
H	-2.487611	1.652657	2.266361
H	-0.886006	0.923542	2.119092
C	-2.427991	0.249140	-0.085741
H	-2.966041	1.204346	-0.155070
H	-2.866148	-0.432324	-0.825656
H	-1.387383	0.423556	-0.372148
C	-4.006186	-0.535561	1.700291
H	-4.541352	0.423747	1.692488
H	-4.127881	-0.966669	2.702797
H	-4.508060	-1.196468	0.985007
C	-2.666146	-3.325245	0.609996
H	-3.596684	-3.501611	1.160128
H	-2.112275	-4.269541	0.571632
H	-2.929018	-3.052486	-0.418487
C	-1.243536	-2.474093	3.200106
H	-0.690951	-1.693188	3.729265
H	-0.658934	-3.399409	3.242916
H	-2.176513	-2.648792	3.748598
C	1.033216	-1.510874	-2.862214
H	1.942425	-1.966574	-3.274848
H	0.609425	-0.856469	-3.623409

H	0.328595	-2.328316	-2.665848
O	1.234987	1.372552	-3.733022
C	2.537238	1.724963	-3.775608
O	3.166255	2.066203	-2.805847
C	3.063769	1.629446	-5.181951
H	2.526694	2.336335	-5.820934
H	2.893668	0.625802	-5.581036
H	4.128010	1.862054	-5.187163

Conformer 5 (-954879.94 kcal/mol)

Atom	x	y	z
C	-1.735907	1.404737	-1.227063
C	-0.687487	2.185600	-1.543096
C	0.408626	2.713873	-0.697390
C	0.515234	2.406196	0.707757
C	0.049876	1.337919	1.419046
C	-0.738996	0.205924	0.837368
H	-0.104727	-0.309202	0.100252
C	-1.979932	0.713767	0.098763
H	-2.613712	-0.160405	-0.090037
H	-2.554076	1.366100	0.770473
C	-0.479974	2.797915	-2.915100
H	-1.243785	3.554603	-3.123002
C	0.873510	3.512121	-2.808505
O	1.478034	3.988623	-3.745823
C	1.265573	3.507268	-1.395186
O	1.233673	3.228458	1.525394
C	0.501076	1.516437	2.758437
H	0.303209	0.865941	3.596870
C	1.205933	2.679093	2.757590
H	1.722573	3.230562	3.527456
C	2.491716	4.224327	-0.936874
H	3.120679	3.583725	-0.311912
H	2.240202	5.110925	-0.345229
H	3.063124	4.542732	-1.812744
O	-1.127940	-0.675119	1.868944
Si	-0.890561	-2.338249	1.796790
C	0.920164	-2.771064	2.186870
C	1.882772	-1.967782	1.294364
H	1.691953	-2.130536	0.226559
H	2.920907	-2.272022	1.488242
H	1.818360	-0.891808	1.491303
C	1.224595	-2.452084	3.659945
H	2.277048	-2.671220	3.888693
H	0.607588	-3.048550	4.342261

H	1.053073	-1.393398	3.886712
C	1.143015	-4.273832	1.939791
H	2.169265	-4.554853	2.212868
H	1.001964	-4.538837	0.885777
H	0.465676	-4.896030	2.538150
C	-2.048365	-3.038200	3.090866
H	-1.898337	-4.115842	3.223803
H	-3.091968	-2.876350	2.803396
H	-1.891340	-2.554739	4.060106
C	-1.356249	-2.960969	0.081231
H	-0.695005	-2.572222	-0.700999
H	-2.382416	-2.669051	-0.170311
H	-1.305182	-4.054699	0.041257
C	-2.824337	1.110094	-2.230460
H	-2.759129	0.067151	-2.566872
H	-2.791950	1.745382	-3.115901
H	-3.804410	1.226306	-1.753357
O	-0.562544	1.919393	-4.033642
C	0.332780	0.910118	-4.082299
O	1.163418	0.711182	-3.230847
C	0.140925	0.096987	-5.333161
H	0.779864	-0.785249	-5.293369
H	0.408519	0.703964	-6.202463
H	-0.906597	-0.195110	-5.441692

Conformer 6 (-954879.87 kcal/mol)

Atom	x	y	z
C	-1.752841	0.106435	-1.445179
C	-1.441234	1.382070	-1.733641
C	-1.238022	2.548534	-0.842127
C	-1.308093	2.443819	0.594959
C	-1.100881	1.371937	1.414160
C	-0.776982	-0.010530	0.935714
H	0.186818	0.029813	0.406361
C	-1.823332	-0.508185	-0.062765
H	-1.675087	-1.589727	-0.161625
H	-2.824616	-0.376085	0.368543
C	-1.327963	1.917984	-3.147097
H	-2.303095	1.921344	-3.644110
C	-0.884532	3.375348	-2.964052
O	-0.528099	4.105952	-3.863857
C	-0.979448	3.691096	-1.534885
O	-1.534623	3.561281	1.345044
C	-1.216042	1.855611	2.748919
H	-1.122989	1.277118	3.655568

C	-1.481657	3.184070	2.639560
H	-1.660135	3.964456	3.362946
C	-0.709585	5.072720	-1.034759
H	-0.027099	5.064484	-0.179910
H	-1.631900	5.568137	-0.711091
H	-0.267225	5.661173	-1.843446
O	-0.715101	-0.904506	2.024624
Si	0.728995	-1.569880	2.569924
C	1.320941	-2.935628	1.384643
C	0.274590	-4.061529	1.329460
H	0.133922	-4.534681	2.308673
H	0.592916	-4.845897	0.628455
H	-0.701592	-3.689927	0.994832
C	1.542757	-2.392002	-0.038383
H	1.961757	-3.178751	-0.680844
H	2.240013	-1.546568	-0.059555
H	0.604436	-2.065120	-0.501335
C	2.652444	-3.506139	1.907131
H	2.992327	-4.327671	1.262158
H	2.557622	-3.909176	2.922490
H	3.444937	-2.749141	1.916217
C	2.021741	-0.212292	2.717996
H	2.942905	-0.597119	3.171038
H	1.649814	0.600813	3.352524
H	2.287389	0.223630	1.747851
C	0.309208	-2.275807	4.253797
H	-0.532041	-2.973976	4.191129
H	0.025635	-1.474656	4.944930
H	1.161022	-2.808183	4.691243
C	-2.048383	-0.897341	-2.532302
H	-1.221317	-1.615404	-2.619588
H	-2.205180	-0.451554	-3.515253
H	-2.940698	-1.476840	-2.265175
O	-0.495425	1.188846	-4.046586
C	0.809173	1.083501	-3.720634
O	1.287985	1.548410	-2.716213
C	1.554278	0.319613	-4.780894
H	2.579670	0.150588	-4.452058
H	1.554610	0.898282	-5.709593
H	1.057311	-0.632752	-4.983928

Conformer 7 (-954880.11 kcal/mol)

Atom	x	y	z
C	-1.327787	-0.084314	-1.858088
C	-1.040657	1.164068	-2.268404

C	-0.784096	2.396681	-1.487078
C	-0.721498	2.405387	-0.046217
C	-0.492217	1.401145	0.851835
C	-0.270125	-0.042544	0.514934
H	0.701953	-0.136065	0.002506
C	-1.342177	-0.586523	-0.429460
H	-1.209444	-1.674511	-0.449230
H	-2.328483	-0.408554	0.020877
C	-1.033122	1.579175	-3.721900
H	-2.002123	1.436664	-4.209721
C	-0.687764	3.069473	-3.691618
O	-0.464057	3.743068	-4.675018
C	-0.632999	3.491458	-2.286893
O	-0.836123	3.594628	0.614821
C	-0.472353	2.009293	2.140085
H	-0.326934	1.511157	3.086341
C	-0.689069	3.333800	1.928653
H	-0.773086	4.184256	2.587902
C	-0.378974	4.918289	-1.924156
H	0.450807	5.014511	-1.216630
H	-1.257104	5.370473	-1.452010
H	-0.141617	5.477102	-2.831663
O	-0.285109	-0.808024	1.698778
Si	1.073601	-1.606479	2.281170
C	0.522760	-2.311456	3.948075
C	-0.003332	-1.171770	4.837922
H	0.767971	-0.416325	5.031858
H	-0.327134	-1.564895	5.811679
H	-0.862337	-0.672566	4.376553
C	-0.599487	-3.340012	3.733110
H	-0.952416	-3.723356	4.700488
H	-0.255702	-4.199635	3.145530
H	-1.456802	-2.897151	3.215786
C	1.713554	-2.993318	4.642611
H	1.399012	-3.414939	5.607646
H	2.528709	-2.287727	4.844173
H	2.118441	-3.816225	4.041761
C	1.570126	-2.949300	1.063500
H	2.456515	-3.493956	1.408094
H	1.811287	-2.521814	0.083066
H	0.762015	-3.674711	0.920682
C	2.487466	-0.378992	2.472004
H	2.249397	0.399755	3.204006
H	2.710520	0.120145	1.522098
H	3.403281	-0.884292	2.799510
C	-1.689847	-1.163218	-2.848360

H	-0.849809	-1.858972	-2.974484
H	-1.969442	-0.786928	-3.833864
H	-2.530453	-1.751510	-2.461442
O	-0.027023	0.901315	-4.476861
C	-0.385821	0.408033	-5.679663
O	-1.522556	0.370553	-6.084623
C	0.823756	-0.088473	-6.423405
H	0.507584	-0.634312	-7.312295
H	1.434623	-0.726982	-5.778994
H	1.435703	0.769418	-6.715975

Conformer 8 (-954880.84 kcal/mol)

Atom	x	y	z
C	1.399381	-1.527551	0.760062
C	1.217706	-1.296468	-0.550245
C	1.410005	-0.050867	-1.323909
C	1.683496	1.224027	-0.699715
C	1.621486	1.647818	0.601169
C	1.304448	0.808471	1.806091
H	1.680873	1.349071	2.686773
C	2.018766	-0.544607	1.735633
H	3.074787	-0.375235	1.492265
H	1.990584	-0.990299	2.734575
C	0.720157	-2.329690	-1.533477
H	-0.320340	-2.619742	-1.353656
C	0.833863	-1.637998	-2.893554
O	0.654867	-2.183906	-3.961258
C	1.191442	-0.232261	-2.657604
O	1.981407	2.280501	-1.509255
C	1.915898	3.043721	0.578534
H	1.969201	3.708587	1.428908
C	2.119215	3.367058	-0.724131
H	2.371542	4.284850	-1.231930
C	1.241592	0.737862	-3.791761
H	2.238637	1.172787	-3.908666
H	0.542285	1.566421	-3.639229
H	0.976227	0.212858	-4.713039
O	-0.068079	0.544132	1.978190
Si	-1.432062	1.516529	2.007518
C	-2.325105	1.482902	0.325184
C	-1.705520	2.464348	-0.683131
H	-1.746475	3.499102	-0.323557
H	-2.256940	2.424383	-1.633119
H	-0.660867	2.224968	-0.902294
C	-2.268474	0.058922	-0.254063

H	-2.820700	0.010119	-1.202953
H	-2.715433	-0.676695	0.425848
H	-1.236776	-0.252427	-0.443462
C	-3.798057	1.879882	0.543428
H	-4.327798	1.902874	-0.418944
H	-3.895543	2.877528	0.991205
H	-4.323395	1.167521	1.189087
C	-2.510771	0.724421	3.323209
H	-3.419365	1.307689	3.507575
H	-1.960704	0.647979	4.267146
H	-2.813112	-0.287600	3.032467
C	-1.003430	3.278363	2.513094
H	-0.449658	3.809884	1.733418
H	-0.403991	3.291909	3.430893
H	-1.921286	3.843701	2.713151
C	0.994483	-2.832538	1.391445
H	1.876073	-3.402665	1.712835
H	0.390493	-3.468049	0.742778
H	0.405440	-2.616782	2.290444
O	1.537062	-3.499091	-1.560771
C	0.911673	-4.693988	-1.544919
O	-0.274868	-4.835785	-1.376142
C	1.908171	-5.803917	-1.741198
H	2.656234	-5.774561	-0.943563
H	2.430494	-5.665566	-2.691489
H	1.390762	-6.762900	-1.731890

Conformer 9 (-954880.27 kcal/mol)

Atom	x	y	z
C	-1.695113	-1.478908	0.576770
C	-1.523199	-2.039666	-0.634001
C	-1.367353	-1.413588	-1.968930
C	-1.288034	0.013978	-2.151212
C	-0.965672	1.018366	-1.281417
C	-0.613792	0.842886	0.164480
H	0.348890	0.312607	0.224610
C	-1.658196	0.000174	0.900226
H	-1.450034	0.107011	1.970722
H	-2.648123	0.446623	0.736868
C	-1.568058	-3.530991	-0.871819
H	-2.518032	-3.981541	-0.570929
C	-1.381447	-3.681192	-2.383573
O	-1.264213	-4.743009	-2.957978
C	-1.328474	-2.337227	-2.970432
O	-1.509436	0.535681	-3.392730

C	-1.000300	2.227725	-2.032273
H	-0.815442	3.221886	-1.656088
C	-1.336618	1.868748	-3.299209
H	-1.494989	2.426733	-4.208970
C	-1.191312	-2.149569	-4.445972
H	-0.397833	-1.438274	-4.690669
H	-2.118336	-1.766285	-4.887263
H	-0.965139	-3.114917	-4.905160
O	-0.521523	2.111217	0.776260
Si	0.751449	2.585578	1.766930
C	2.230467	3.144673	0.709451
C	2.611375	2.055222	-0.307287
H	2.858087	1.104138	0.180884
H	3.495763	2.363250	-0.882302
H	1.805249	1.870876	-1.026661
C	1.872404	4.432938	-0.050316
H	2.714313	4.750208	-0.681207
H	1.643347	5.258760	0.633039
H	1.006688	4.289112	-0.706184
C	3.435737	3.422384	1.624992
H	4.282501	3.795329	1.033132
H	3.773161	2.517296	2.142763
H	3.210650	4.181349	2.384068
C	0.068605	3.998847	2.784909
H	0.847434	4.462741	3.401491
H	-0.720025	3.640505	3.453405
H	-0.362395	4.776794	2.144915
C	1.251539	1.152191	2.883465
H	1.643523	0.296092	2.323082
H	0.398091	0.803321	3.475642
H	2.030277	1.468448	3.586635
C	-1.965224	-2.325822	1.796258
H	-1.073927	-2.358323	2.437650
H	-2.256877	-3.352572	1.569891
H	-2.765255	-1.870234	2.391468
O	-0.498226	-4.217049	-0.218584
C	-0.806203	-5.345285	0.454164
O	-1.932266	-5.729885	0.653347
C	0.446701	-6.032993	0.922861
H	0.183671	-6.856975	1.584401
H	1.099662	-5.323464	1.437666
H	0.991481	-6.415918	0.054721

Conformer 10 (-954880.20 kcal/mol)

Atom	x	y	z

C	-1.503699	0.137462	-1.352097
C	-1.303566	1.438956	-1.625659
C	-1.169137	2.600062	-0.713348
C	-1.113536	2.459758	0.721072
C	-0.815113	1.384677	1.511340
C	-0.496487	0.001379	1.027709
H	0.478038	0.026666	0.515677
C	-1.538090	-0.494899	0.023605
H	-1.375815	-1.572392	-0.090156
H	-2.536289	-0.382957	0.467695
C	-1.285138	2.000200	-3.028323
H	-2.208511	1.803563	-3.580396
C	-1.107816	3.508079	-2.832837
O	-0.951359	4.303051	-3.735400
C	-1.108235	3.782281	-1.391064
O	-1.317269	3.560690	1.502835
C	-0.846948	1.852223	2.856111
H	-0.677159	1.266119	3.746304
C	-1.158760	3.172863	2.783216
H	-1.307892	3.939684	3.527480
C	-0.982254	5.179146	-0.877075
H	-0.175694	5.268078	-0.142474
H	-1.904177	5.509785	-0.387958
H	-0.776322	5.847165	-1.718255
O	-0.463005	-0.886534	2.121835
Si	0.928946	-1.630386	2.698297
C	1.370171	-3.126796	1.610712
C	0.177601	-4.096857	1.568980
H	-0.070235	-4.479911	2.565603
H	0.409493	-4.962251	0.932852
H	-0.720447	-3.615819	1.164884
C	1.709654	-2.688107	0.175622
H	1.984901	-3.560700	-0.431969
H	2.554750	-1.991520	0.147433
H	0.858881	-2.206739	-0.320881
C	2.591070	-3.845940	2.211575
H	2.851594	-4.725111	1.605778
H	2.397587	-4.199749	3.231231
H	3.475104	-3.197396	2.240758
C	2.339745	-0.385673	2.729281
H	3.227325	-0.817507	3.205549
H	2.054835	0.504209	3.301142
H	2.633727	-0.052648	1.728295
C	0.483427	-2.164538	4.437948
H	-0.396903	-2.814894	4.440176
H	0.250653	-1.291147	5.056143

```
H        1.306549     -2.704517      4.920615
C       -1.730049     -0.868093     -2.453096
H       -0.845716     -1.510180     -2.563282
H       -1.951911     -0.424540     -3.424832
H       -2.564790     -1.526961     -2.184319
O       -0.176082      1.517232     -3.789321
C       -0.421341      1.109352     -5.051590
O       -1.525901      0.993343     -5.524189
C        0.869837      0.811677     -5.763674
H        0.656342      0.353411     -6.728287
H        1.493815      0.149564     -5.157108
H        1.421598      1.744085     -5.910163
```

Final Energy (Gibbs Free Energy) for *cis*-14a

-1522.112220448 (au/mol) from WB97X-V/6-311+G(2DF,2P)[6-311G* of -1522.112663000 (au) + 1.162 (kJ/mol) from 9 conformers

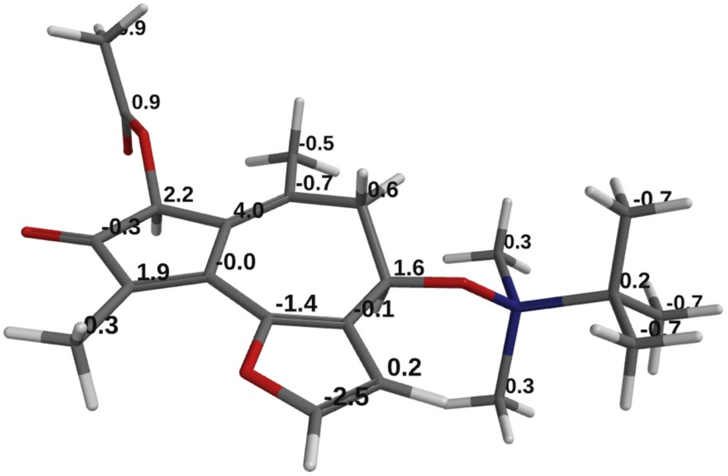

Figure S24. Calculated (*cis*-14a) minus experimental (minor isomer) chemical shifts

Table S4. Computed NMR chemical shifts for *cis*-14a

position	δ C	mult	δ H	splitting (Hz)	HMBC (H→C) (2,3-bond)
**1**	24.8	CH_3	1.93	s	11, 12, 17

**2**	10.6	CH_3	2.12	s	13, 18, 19
**3**	19.9	CH_3	1.96	s	14
**4**	25.2	CH_3	1.11	s	5, 6, 22
**5**	25.2	CH_3	1.11	s	4, 6, 22
**6**	25.2	CH_3	1.11	s	4, 5, 22
**7**	-4.4	CH_3	0.15	s	
**8**	-4.3	CH_3	0.11	s	
**9**	112.3	CH	6.71	d (1.2)	10, 15, 16, 20
**10**	142.4	CH	7.37	d (1.2)	9, 15, 20
**11**	45.2	CH_2	2.34	dd (20.1, 1.5)	1, 12, 15, 16, 17
**.**			3.07	dd (20.1, 11.3)	1, 12, 15, 16, 17
**12**	132.3	C			...
**13**	135.0	C			...
**14**	170.8	C			...
**15**	137.0	C			...
**16**	67.2	CH	5.22	dd (11.3, 1.5)	9, 11, 12, 15, 20
**17**	132.3	C			...
**18**	148.8	C			...

**19**	199.2	C			...
**20**	143.1	C			...
**21**	74.0	CH	5.36	s	12, 13, 14, 17, 18, 19
**22**	18.6	C			...

DP4 Analysis for minor isomer

Comparison of computed chemical shifts for *cis*-14a to experimental chemical shifts for minor isomer:

DP4 Score 100% using the Boltzmann average

RMS: 1.35

Maximum Absolute: 4.02

Mean absolute: 0.96

Cartesian coordinates for *cis*-14a

```
Conformer 1 (-954879.92 kcal/mol)

Atom       x     y          z
C        0.037529     -0.986275      1.666765
C       -0.159937     -0.069722      2.628887
C       -0.766968      1.278374      2.536761
C       -1.024650      1.934639      1.276464
C       -0.694518      1.624075     -0.017201
C        0.001261      0.387565     -0.504473
H        1.083811      0.502769     -0.325739
C       -0.473996     -0.859883      0.247074
H       -1.570593     -0.873790      0.228328
H       -0.140141     -1.727887     -0.329538
C       -0.958247      1.835160      3.767123
C       -0.473070      0.904715      4.793897
C        0.175083     -0.286071      4.085099
```

C	-1.531944	3.157609	4.156633
H	-2.560104	3.272214	3.802236
H	-0.952241	3.986058	3.739231
H	-1.525051	3.232900	5.248110
O	-0.556677	1.021463	5.998435
C	0.763783	-2.277189	1.946295
H	0.059936	-3.118730	1.976365
H	1.327800	-2.268753	2.879496
H	1.468460	-2.485094	1.132311
O	-1.643660	3.151867	1.305530
C	-1.138941	2.713366	-0.820736
H	-1.056239	2.794596	-1.894239
C	-1.706810	3.599879	0.036877
H	-2.190578	4.554025	-0.098980
O	-0.257774	0.233040	-1.883001
Si	0.970356	-0.032702	-2.998495
C	0.075874	-0.262815	-4.647985
C	-0.753393	0.993272	-4.966722
H	-1.498333	1.187186	-4.187534
H	-0.121980	1.883953	-5.066393
C	1.109158	-0.489832	-5.764950
H	0.603266	-0.625725	-6.730645
H	1.712931	-1.386353	-5.583186
H	1.791757	0.361884	-5.872340
C	-0.864584	-1.477039	-4.566474
H	-1.610538	-1.354395	-3.774421
H	-0.315007	-2.405633	-4.372672
H	-1.399920	-1.605366	-5.517033
H	-1.289142	0.864949	-5.917488
C	2.115788	1.460522	-3.013222
H	1.574090	2.374152	-3.280300
H	2.934221	1.328972	-3.731342
H	2.569885	1.622322	-2.027672
C	1.948749	-1.562273	-2.502084
H	1.318010	-2.457522	-2.492221
H	2.379674	-1.446398	-1.500494
H	2.779377	-1.743217	-3.193198
C	0.380783	-2.278013	5.356704
O	1.572234	-2.128425	5.482315
C	-0.434067	-3.374907	5.983861
H	-1.112043	-2.939419	6.723074
H	0.229990	-4.091648	6.466557
H	-1.046640	-3.871073	5.227338
O	-0.407247	-1.478980	4.608403
H	1.251226	-0.296398	4.280607

Conformer 2 (-954879.37 kcal/mol)

Atom	x	y	z
C	0.947278	0.149312	1.726698
C	0.023361	0.001918	2.690214
C	-1.354404	-0.535301	2.592003
C	-2.031019	-0.724514	1.329197
C	-1.706598	-0.391097	0.040276
C	-0.427759	0.225346	-0.441463
H	-0.467414	1.311458	-0.252876
C	0.781464	-0.345244	0.304950
H	0.714924	-1.439275	0.282354
H	1.671302	-0.071599	-0.270720
C	-1.912302	-0.737839	3.819191
C	-0.960971	-0.321667	4.856286
C	0.232813	0.339734	4.153280
C	-3.251946	-1.275632	4.200641
H	-3.405801	-2.283933	3.804619
H	-4.062453	-0.649154	3.815195
H	-3.317140	-1.310509	5.291561
O	-1.085394	-0.414056	6.058475
C	2.285718	0.793981	1.983622
H	3.092207	0.057486	1.867459
H	2.376731	1.238971	2.974543
H	2.464728	1.572946	1.231534
O	-3.285480	-1.264627	1.352881
C	-2.824247	-0.753107	-0.765517
H	-2.902977	-0.647794	-1.837116
C	-3.742379	-1.277893	0.085817
H	-4.726650	-1.697848	-0.053191
O	-0.291596	-0.030162	-1.822338
Si	0.141591	1.153912	-2.930714
C	0.478171	0.196094	-4.526041
C	-0.778695	-0.597184	-4.922670
H	-1.078013	-1.292926	-4.131678
H	-1.627094	0.064597	-5.133953
C	0.847644	1.171841	-5.655494
H	1.049983	0.619955	-6.583582
H	1.748998	1.750886	-5.418118
H	0.037307	1.880163	-5.865822
C	1.639408	-0.786524	-4.296983
H	1.415591	-1.486378	-3.485116
H	2.570716	-0.264105	-4.048013
H	1.826862	-1.373122	-5.206628
H	-0.586769	-1.183690	-5.831588
C	-1.276399	2.375224	-3.113633

H	-2.180711	1.885078	-3.490666
H	-1.017698	3.185919	-3.804517
H	-1.526133	2.832412	-2.149757
C	1.669209	2.069178	-2.319068
H	2.525530	1.396283	-2.204013
H	1.484693	2.545346	-1.348458
H	1.956640	2.861971	-3.018918
C	1.760706	-1.340187	4.800655
O	1.019358	-2.199006	4.394126
C	3.105716	-1.562254	5.437329
H	3.885214	-1.073611	4.844706
H	3.302508	-2.632189	5.501276
H	3.119843	-1.117600	6.436350
O	1.482800	-0.019946	4.732130
H	0.169028	1.419961	4.321280

Conformer 3 (-954879.65 kcal/mol)

Atom	x	y	z
C	-0.282587	-1.411241	1.395045
C	-0.398615	-0.544158	2.414431
C	-0.936581	0.836597	2.416630
C	-1.200469	1.574585	1.203304
C	-0.910786	1.329050	-0.113104
C	-0.252835	0.102868	-0.672011
H	0.826237	0.156221	-0.458191
C	-0.804902	-1.162406	-0.005024
H	-1.900007	-1.109566	-0.006624
H	-0.529773	-2.011683	-0.637715
C	-1.078701	1.329040	3.679163
C	-0.620906	0.322848	4.643549
C	-0.007050	-0.838817	3.849435
C	-1.597548	2.646699	4.153819
H	-2.633667	2.802354	3.839602
H	-1.009777	3.478812	3.753679
H	-1.552229	2.670049	5.245083
O	-0.654822	0.384748	5.853577
C	0.364545	-2.763041	1.559055
H	-0.373808	-3.560278	1.402090
H	0.820386	-2.916410	2.536831
H	1.134421	-2.894027	0.788084
O	-1.794118	2.799500	1.314936
C	-1.363127	2.464619	-0.843650
H	-1.318066	2.597838	-1.913206
C	-1.887576	3.316193	0.075006
H	-2.357188	4.285462	0.003604

O	-0.475375	0.056122	-2.065051
Si	0.708287	-0.356300	-3.184784
C	1.752849	1.175520	-3.610464
C	2.270127	1.845013	-2.326092
H	1.448785	2.211294	-1.699107
H	2.880227	1.160296	-1.723350
C	2.952603	0.756005	-4.477563
H	3.532494	1.639925	-4.776711
H	2.637829	0.247196	-5.397236
H	3.632773	0.086849	-3.937700
C	0.897487	2.186107	-4.392739
H	0.013901	2.496829	-3.824043
H	0.552288	1.774465	-5.347837
H	1.480732	3.090219	-4.614951
H	2.901880	2.709306	-2.574189
C	1.806629	-1.715497	-2.478249
H	2.361081	-1.390368	-1.590390
H	2.541781	-2.039687	-3.223222
H	1.213202	-2.593755	-2.201116
C	-0.229444	-0.979089	-4.679988
H	-0.983069	-0.252953	-5.000961
H	-0.746786	-1.915041	-4.444789
H	0.440407	-1.167127	-5.526816
C	-1.685820	-2.378129	4.476836
O	-2.547279	-1.603778	4.144365
C	-1.904608	-3.751000	5.054515
H	-1.416796	-4.504317	4.429571
H	-2.974381	-3.951294	5.115426
H	-1.457044	-3.806267	6.050452
O	-0.364965	-2.114847	4.372005
H	1.079752	-0.801556	3.976778

Conformer 4 (-954880.09 kcal/mol)

Atom	x	y	z
C	-0.373776	-1.435618	1.300268
C	-0.532590	-0.586461	2.330093
C	-1.119016	0.774032	2.353292
C	-1.448078	1.504705	1.152495
C	-1.161971	1.286722	-0.169556
C	-0.420840	0.115871	-0.743698
H	0.649920	0.238070	-0.518888
C	-0.892407	-1.195264	-0.102702
H	-1.988627	-1.215797	-0.119306
H	-0.555619	-2.012459	-0.746811
C	-1.237062	1.258828	3.623080

C	-0.722904	0.259807	4.566948
C	-0.159685	-0.911892	3.757792
C	-1.766104	2.566519	4.113511
H	-2.820907	2.694729	3.855125
H	-1.223927	3.409487	3.673765
H	-1.663439	2.601245	5.201485
O	-0.733623	0.313466	5.778670
C	0.324081	-2.760886	1.473641
H	-0.398200	-3.586802	1.423327
H	0.878305	-2.848037	2.409661
H	1.033202	-2.910042	0.649959
O	-2.122864	2.685149	1.281853
C	-1.701256	2.393136	-0.885936
H	-1.678482	2.536816	-1.955074
C	-2.269783	3.200934	0.046375
H	-2.805909	4.135487	-0.011510
O	-0.630024	0.075594	-2.138433
Si	0.594132	-0.221375	-3.253117
C	1.532559	1.391060	-3.631695
C	1.965022	2.087103	-2.329994
H	1.103017	2.393395	-1.726170
H	2.602494	1.443012	-1.710971
C	2.783536	1.068175	-4.468549
H	3.298777	1.995328	-4.753988
H	2.535135	0.536618	-5.395809
H	3.500634	0.455174	-3.910562
C	0.624884	2.341125	-4.430979
H	-0.300816	2.570493	-3.889918
H	0.347838	1.917109	-5.403008
H	1.140335	3.292824	-4.620590
H	2.543730	2.992681	-2.557898
C	1.777436	-1.517376	-2.568298
H	2.307553	-1.174301	-1.672229
H	2.534559	-1.777673	-3.316123
H	1.243230	-2.438227	-2.309908
C	-0.285369	-0.864357	-4.776056
H	-1.093102	-0.188129	-5.072586
H	-0.726746	-1.846889	-4.582314
H	0.400716	-0.965503	-5.625468
C	-0.023801	-2.985887	4.902728
O	1.176185	-2.903562	5.003545
C	-0.879276	-4.078091	5.484517
H	-1.555670	-3.648906	6.229022
H	-0.244248	-4.832306	5.947673
H	-1.493447	-4.531271	4.701257
O	-0.788572	-2.105459	4.224471

H	0.920862	-0.992124	3.913589

Conformer 5 (-954879.43 kcal/mol)

Atom	x	y	z
C	0.684308	-0.975454	1.261805
C	-0.016329	-0.604846	2.346352
C	-1.416249	-0.128254	2.447663
C	-2.213555	0.214364	1.292525
C	-1.901052	0.388835	-0.028818
C	-0.554822	0.193841	-0.663173
H	0.085502	1.051369	-0.402152
C	0.119300	-1.077952	-0.140461
H	-0.597224	-1.905540	-0.207332
H	0.934581	-1.314684	-0.831299
C	-1.822280	0.000001	3.742153
C	-0.717575	-0.367508	4.634322
C	0.521471	-0.619521	3.764401
C	-3.137157	0.425646	4.306963
H	-3.928319	-0.280332	4.038284
H	-3.440881	1.408506	3.934041
H	-3.057298	0.461994	5.396302
O	-0.728194	-0.422358	5.845334
C	2.136965	-1.371344	1.342793
H	2.263094	-2.416132	1.031281
H	2.569273	-1.263524	2.337145
H	2.720530	-0.764343	0.639137
O	-3.537025	0.493101	1.485748
C	-3.105439	0.786723	-0.678524
H	-3.235237	0.984638	-1.731596
C	-4.056332	0.828795	0.289825
H	-5.110809	1.058783	0.283237
O	-0.703879	0.096814	-2.062060
Si	-0.146195	1.234161	-3.163038
C	1.719053	1.004844	-3.451778
C	1.995651	-0.440558	-3.896667
H	1.661776	-1.165343	-3.145705
H	1.487435	-0.679847	-4.837973
C	2.187881	1.972532	-4.552762
H	3.263416	1.846485	-4.736917
H	2.025499	3.020595	-4.274366
H	1.671765	1.791538	-5.502826
C	2.513395	1.294616	-2.167044
H	2.239061	0.614471	-1.351824
H	2.364657	2.321395	-1.813786
H	3.588933	1.163863	-2.348016

H	3.071859	-0.593431	-4.056256
C	-1.120910	0.894629	-4.726520
H	-0.959998	-0.128370	-5.081065
H	-0.840282	1.582566	-5.532847
H	-2.192816	1.016552	-4.541639
C	-0.510468	2.965453	-2.521289
H	-1.574807	3.075403	-2.287885
H	-0.255340	3.719090	-3.275091
H	0.051939	3.206262	-1.612480
C	0.571171	-2.939382	4.210225
O	-0.594009	-3.039599	3.920073
C	1.467938	-4.060888	4.659476
H	2.329276	-4.144607	3.990578
H	0.904362	-4.992938	4.663279
H	1.845969	-3.849428	5.663733
O	1.252830	-1.774422	4.168737
H	1.225589	0.202738	3.927775

Conformer 6 (-954879.94 kcal/mol)

Atom	x	y	z
C	-1.198670	-0.517283	1.055826
C	-1.804344	0.618622	1.442138
C	-2.083763	1.851560	0.669595
C	-1.517868	2.100446	-0.635150
C	-0.538940	1.466983	-1.354537
C	0.199667	0.217603	-0.968331
H	0.953440	0.474385	-0.207677
C	-0.753291	-0.817288	-0.360976
H	-1.619425	-0.922585	-1.025602
H	-0.232811	-1.779728	-0.373094
C	-2.860522	2.728959	1.368962
C	-3.153817	2.160418	2.689497
C	-2.393131	0.838079	2.816099
C	-3.383691	4.074836	0.987678
H	-4.055713	4.012926	0.126503
H	-2.574794	4.759613	0.714729
H	-3.934589	4.491279	1.834753
O	-3.869998	2.623150	3.551941
C	-0.949776	-1.641063	2.028773
H	-1.640759	-2.473048	1.841742
H	-1.046429	-1.345318	3.074299
H	0.063545	-2.034832	1.886246
O	-1.939130	3.206594	-1.317493
C	-0.360315	2.227734	-2.545728
H	0.315316	2.004456	-3.357521

C	-1.236772	3.262079	-2.464327
H	-1.477441	4.082642	-3.122783
O	0.816130	-0.332596	-2.109907
Si	2.476456	-0.418943	-2.350917
C	3.194445	-1.904281	-1.406012
C	2.453886	-3.184618	-1.826192
H	1.380143	-3.117728	-1.617185
H	2.571985	-3.389639	-2.896591
C	4.689701	-2.049350	-1.739352
H	5.117492	-2.904277	-1.199588
H	5.263580	-1.161294	-1.449336
H	4.855902	-2.223953	-2.808656
C	3.040366	-1.711186	0.112243
H	1.988776	-1.624126	0.411418
H	3.567249	-0.818760	0.469182
H	3.458105	-2.573429	0.649201
H	2.848771	-4.052306	-1.280050
C	2.675123	-0.630404	-4.200885
H	2.154394	-1.524558	-4.557766
H	3.730407	-0.715639	-4.483437
H	2.256495	0.231164	-4.731457
C	3.277622	1.185440	-1.779631
H	3.180290	1.345412	-0.700204
H	2.822544	2.046311	-2.282023
H	4.347859	1.189481	-2.016667
C	-3.237327	-0.686012	4.423435
O	-2.321723	-0.463229	5.177529
C	-4.415241	-1.574850	4.716054
H	-5.324266	-0.966825	4.742475
H	-4.271073	-2.067277	5.676940
H	-4.536953	-2.317248	3.921960
O	-3.333383	-0.173405	3.179134
H	-1.633734	0.916907	3.600721

Conformer 7 (-954879.32 kcal/mol)

Atom	x	y	z
C	1.387765	-0.798618	1.655267
C	1.074443	0.125089	2.575856
C	0.251624	1.345159	2.419908
C	-0.037056	1.928434	1.128743
C	0.330259	1.602052	-0.151600
C	1.060557	0.374063	-0.623651
H	2.140113	0.574230	-0.596233
C	0.778602	-0.839639	0.269727
H	-0.307743	-0.972443	0.357295

H	1.166074	-1.717309	-0.256448
C	-0.073584	1.897686	3.622642
C	0.544200	1.108586	4.696259
C	1.471458	0.077152	4.037512
C	-0.896043	3.099027	3.953768
H	-1.890516	3.039420	3.502699
H	-0.430055	4.019296	3.588123
H	-1.000412	3.162519	5.039786
O	0.414479	1.253011	5.892375
C	2.315096	-1.950151	1.947280
H	1.760448	-2.898536	1.946014
H	2.830246	-1.863538	2.903932
H	3.066075	-2.025730	1.151080
O	-0.695735	3.126127	1.118815
C	-0.120704	2.671318	-0.981954
H	-0.005275	2.746084	-2.052561
C	-0.738512	3.553280	-0.157080
H	-1.243394	4.492749	-0.321219
O	0.757878	0.110511	-1.975907
Si	-0.621792	-0.565292	-2.657757
C	-0.496129	-0.091546	-4.490208
C	0.705671	-0.805432	-5.132137
H	1.641396	-0.553127	-4.620829
H	0.589824	-1.895449	-5.107910
C	-1.785049	-0.508253	-5.219073
H	-1.714945	-0.259381	-6.286800
H	-2.665380	0.008724	-4.819347
H	-1.967375	-1.587571	-5.149109
C	-0.309671	1.428217	-4.628875
H	0.626069	1.756774	-4.163643
H	-1.136606	1.983846	-4.167922
H	-0.276018	1.714119	-5.689749
H	0.808170	-0.509185	-6.185492
C	-0.596044	-2.438517	-2.462751
H	0.389457	-2.847120	-2.710829
H	-1.329364	-2.907000	-3.130019
H	-0.841642	-2.743174	-1.439805
C	-2.200831	0.110775	-1.890138
H	-2.346204	1.171894	-2.115252
H	-2.202649	0.008162	-0.799655
H	-3.066199	-0.441075	-2.275284
C	0.227294	-1.786946	4.778013
O	-0.788815	-1.307172	4.342080
C	0.347830	-3.108840	5.486523
H	-0.632357	-3.581808	5.539034
H	0.741062	-2.951181	6.494763

H	1.050016	-3.757047	4.954061
O	1.435724	-1.190844	4.681503
H	2.504266	0.413621	4.176181

Conformer 8 (-954879.48 kcal/mol)

Atom	x	y	z
C	1.217699	-0.819026	1.552468
C	0.876349	0.049908	2.516539
C	0.017070	1.251848	2.429000
C	-0.338158	1.862756	1.168417
C	0.021381	1.604946	-0.130093
C	0.822679	0.446215	-0.660117
H	1.888413	0.705816	-0.609874
C	0.604844	-0.825813	0.167990
H	-0.473325	-1.017555	0.244688
H	1.035793	-1.654935	-0.400410
C	-0.267588	1.763348	3.661602
C	0.409901	0.953157	4.682902
C	1.288364	-0.073849	3.964192
C	-1.087271	2.946277	4.059773
H	-2.097222	2.897265	3.645310
H	-0.638162	3.881896	3.709140
H	-1.150960	2.973940	5.151280
O	0.315690	1.052206	5.887637
C	2.208436	-1.927208	1.800976
H	1.698274	-2.895091	1.893601
H	2.815855	-1.772685	2.694134
H	2.888520	-2.007296	0.945062
O	-1.066658	3.019183	1.216268
C	-0.509923	2.673977	-0.910589
H	-0.419514	2.795415	-1.979284
C	-1.161413	3.487818	-0.041882
H	-1.724133	4.400972	-0.162069
O	0.546331	0.239634	-2.026123
Si	-0.772177	-0.492019	-2.767782
C	-0.633884	0.070254	-4.573126
C	0.620265	-0.550121	-5.212074
H	1.528139	-0.270234	-4.665996
H	0.563622	-1.644799	-5.236545
C	-1.881232	-0.379680	-5.353567
H	-1.798888	-0.079677	-6.407180
H	-2.796760	0.072291	-4.954728
H	-2.008586	-1.468798	-5.336446
C	-0.524863	1.602386	-4.640967
H	0.374259	1.958877	-4.126549

H	-1.397315	2.093494	-4.190428
H	-0.468177	1.935106	-5.687014
H	0.732007	-0.203572	-6.248997
C	-0.638208	-2.366457	-2.652252
H	0.378337	-2.706637	-2.875863
H	-1.314940	-2.845564	-3.369597
H	-0.905920	-2.733018	-1.656014
C	-2.406179	0.047703	-2.006613
H	-2.610852	1.109351	-2.175425
H	-2.427627	-0.117914	-0.923866
H	-3.226538	-0.531695	-2.445636
C	1.982643	-1.961377	5.219436
O	3.109689	-1.540021	5.309493
C	1.465796	-3.225199	5.850594
H	0.701217	-2.970844	6.590728
H	2.287112	-3.753834	6.333229
H	0.997157	-3.861261	5.094654
O	1.005475	-1.360579	4.508819
H	2.345157	0.160718	4.129180

Conformer 9 (-954878.51kcal/mol)

Atom	x	y	z
C	0.900517	-0.434956	1.621498
C	0.486142	0.538193	2.447197
C	-0.252663	1.778689	2.123158
C	-0.336036	2.304733	0.778556
C	0.210297	1.909112	-0.416689
C	0.959865	0.639751	-0.720988
H	2.027055	0.808953	-0.524371
C	0.491648	-0.521196	0.166565
H	-0.599567	-0.597398	0.100525
H	0.901934	-1.439391	-0.263138
C	-0.699407	2.412018	3.246356
C	-0.260670	1.653858	4.426755
C	0.654374	0.523419	3.947092
C	-1.484188	3.672208	3.404652
H	-2.398797	3.656391	2.805356
H	-0.905874	4.545277	3.084531
H	-1.747436	3.791848	4.459013
O	-0.547623	1.858572	5.586796
C	1.751382	-1.579446	2.107177
H	1.154244	-2.494075	2.211605
H	2.241152	-1.379002	3.061425
H	2.532092	-1.792703	1.368584
O	-0.939803	3.520887	0.619627

C	-0.066011	2.955286	-1.346595
H	0.232299	2.989219	-2.383233
C	-0.770227	3.892244	-0.662478
H	-1.205874	4.840003	-0.939035
O	0.878422	0.314892	-2.089267
Si	-0.403627	-0.137418	-3.075207
C	0.088974	-1.788653	-3.873030
C	0.512682	-2.796966	-2.793767
H	1.411739	-2.458576	-2.267619
H	-0.276687	-2.960519	-2.048796
C	-1.108809	-2.358536	-4.652244
H	-0.825715	-3.293630	-5.155042
H	-1.463828	-1.668705	-5.427906
H	-1.954298	-2.584638	-3.991565
C	1.266578	-1.564618	-4.836635
H	2.129246	-1.128477	-4.320343
H	0.994387	-0.896983	-5.662529
H	1.588219	-2.518761	-5.277168
H	0.740108	-3.771458	-3.248070
C	-2.029362	-0.302862	-2.140608
H	-2.089376	-1.221211	-1.547161
H	-2.855558	-0.321583	-2.861261
H	-2.195142	0.549106	-1.471998
C	-0.604693	1.177094	-4.405817
H	0.368358	1.530225	-4.763530
H	-1.165518	2.039965	-4.031190
H	-1.152307	0.780049	-5.268877
C	1.025179	-1.276899	5.445143
O	2.156701	-0.919567	5.664628
C	0.322993	-2.423159	6.119407
H	-0.445309	-2.021653	6.787270
H	1.041251	-3.002688	6.697982
H	-0.176740	-3.054858	5.380662
O	0.208468	-0.692150	4.543445
H	1.684839	0.720746	4.261154

Table S5. ^{1}H (700 MHz) and ^{13}C NMR (175 MHz) data (computed and experimental) for *trans*- and *cis*-**14a** ($CDCl_3$)

atom no.	δ_H, mult (J, Hz) *trans*-**14a**	δ_H, mult (J, Hz) *cis*-**14a**	δ_C (computed δ_C), type *trans*-**14a**	δ_C (computed δ_C), type *cis*-**14a**

1	1.92, s	1.94, s	25.18 (26.0), CH_3	25.27 (24.8), CH_3
2	2.19, s	2.20, s	10.12 (10.4), CH_3	10.31 (10.6), CH_3
3	2.16, s	2.14, s	20.97 (19.9), CH_3	20.83 (19.9), CH_3
4	0.95, s	0.94, s	25.93 (25.2), CH_3	25.9 (25.2), CH_3
5	0.95, s	0.94, s	25.93 (25.2), CH_3	25.9 (25.2), CH_3
6	0.95, s	0.94, s	25.93 (25.2), CH_3	25.9 (25.2), CH_3
7	0.12, s	0.11, s	-4.72 (-3.6), CH_3	-4.73 (-4.4), CH_3
8	0.14, s	0.14, s	-4.6 (-3.6), CH_3	-4.64 (-4.3), CH_3
9	6.59, d (2.1)	6.58, d (1.4)	111.86 (112.7), CH	112.07 (112.3), CH
10	7.57–7.56 (m)		144.96 (142.2), CH	144.87 (142.4), CH
11	2.84, dd (15.8, 10.5)<br>2.52, dd (16.4, 2.8)	2.91, dd (14.4, 10.5)<br>2.42, dd (14.7, 2.8)	44.96 (43.5), CH_2	44.6 (45.2), CH_2
12			134.26 (135.2), C	132.95 (132.3), C
13			132.74 (135.1), C	133.05 (135.0), C
14			169.95 (170.5), C	170.03 (170.8), C
15			136.96 (133.3), C	137.07 (137.0), C
16	4.93, dd (10.5, 2.8)	4.89, dd (10.5, 2.8)	65.49 (66.4), CH	65.56 (67.2), CH
17			127.72 (130.5), C	128.27 (132.3), C
18			149.07 (149.4), C	148.83 (148.8), C
19			199.62 (199.7), C	199.47 (199.2), C
20			144.18 (146.8), C	144.51 (143.1), C
21	5.82, s	5.84, s	71.34 (74.8), CH	71.78 (74.0), CH
22			18.36 (18.5), C	18.37 (18.6), C

Comparing yield and selectivity of proline-catalyzed asymmetric aldol reaction of 15 and 16

We noted that aldehyde **16** gave a much higher yield and enantioselectivity in the aldol reaction, compared to 3-furaldehyde **15** (60% vs 19% yield and 60% ee vs 36% ee). We attribute this to the electron withdrawing propynyl group making aldehyde **16** a stronger electrophile. Our computational studies support this finding, as the computed LUMO energy for aldehyde **16** (0.24 eV) is significantly lower than the computed LUMO of 3-furaldehyde (0.66 eV).

LUMO energies and cartesian coordinates of 15 and 16

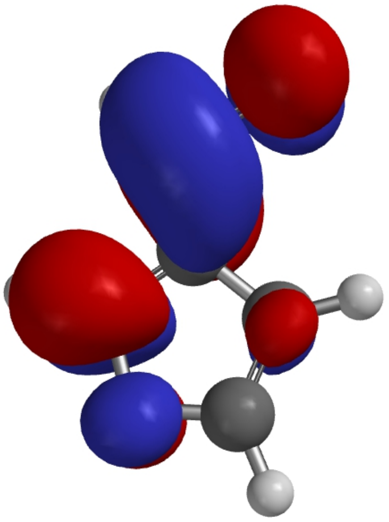

Figure S25. LUMO plot of **15**.

```
Frequency Calculation

Adjusted 1 (out of 48) low frequency modes

Reason for exit: Successful completion

Mechanics CPU Time :       0.0600
Mechanics Wall Time:       0.0452
```

```
SPARTAN'20 Quantum Mechanics Program:  (x86/Darwin)        build  1.1.4
Job type: Geometry optimization.
Method: RWB97X-D
Basis set: 6-31G(D)
Number of basis functions: 162
Number of electrons: 70
Parallel Job: 4 threads
SCF model:
 A restricted hybrid HF-DFT SCF calculation will be
 performed using Pulay DIIS + Geometric Direct Minimization
```

15

```
Conformer 1 (-215380.45 kcal/mol)
LUMO = 0.66 eV
Atom  x           y           z
C       -1.105753      0.000000     -0.583854
C       -0.164992      0.000000      0.404763
C        1.106888      0.000000     -0.263853
C        0.826930      0.000000     -1.585990
H        2.077344      0.000000      0.208646
H        1.431013      0.000000     -2.479307
O       -0.521935      0.000000     -1.792926
C       -0.420991      0.000000      1.845800
H       -1.493265      0.000000      2.134486
O        0.450334      0.000000      2.687804
H       -2.185572      0.000000     -0.575571
```

15

```
Conformer 2 (-215380.00)kcal/mol)
LUMO = 0.60 eV
Atom  x           y           z
C       -1.100470      0.000000     -0.585010
C       -0.161434      0.000000      0.406377
C        1.111430      0.000000     -0.261926
C        0.834425      0.000000     -1.586279
H        2.090500      0.000000      0.195671
H        1.440687      0.000000     -2.478009
O       -0.512507      0.000000     -1.791173
C       -0.436190      0.000000      1.846145
H        0.462930      0.000000      2.497057
O       -1.550409      0.000000      2.324407
H       -2.178962      0.000000     -0.567261
```

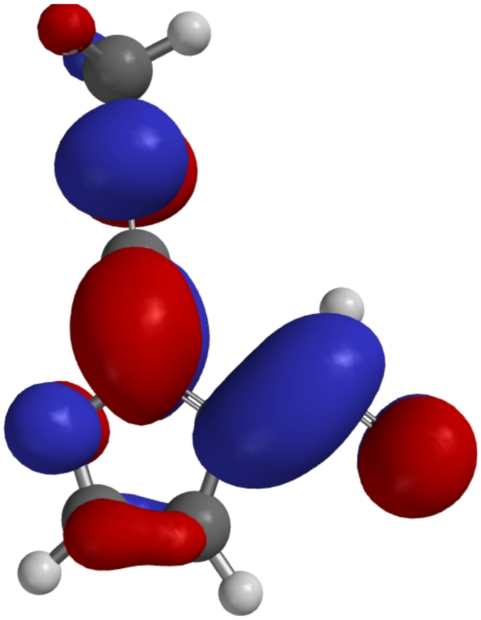

Figure S26. LUMO plot of **16**.

```
SPARTAN'20 MECHANICS PROGRAM:   (x86/Darwin)                           build
1.1.4
Frequency Calculation

  Adjusted 1 (out of 48) low frequency modes

  Reason for exit: Successful completion
  Mechanics CPU Time :       0.0600
  Mechanics Wall Time:       0.0452

SPARTAN'20 Quantum Mechanics Program:  (x86/Darwin)         build  1.1.4

Job type: Geometry optimization.
Method: RWB97X-D
Basis set: 6-31G(D)
Number of basis functions: 162
Number of electrons: 70
Parallel Job: 4 threads
SCF model:
 A restricted hybrid HF-DFT SCF calculation will be
 performed using Pulay DIIS + Geometric Direct Minimization
```

16

```
Conformer 1 (-287813.93 kcal/mol)
LUMO = 0.24 eV
Atom  x           y           z
```

C	-0.557496	0.000000	-0.407193
C	0.229062	0.000000	-1.535207
C	-0.669695	0.000000	-2.649393
C	-1.914121	0.000000	-2.119083
H	-0.392749	0.000000	-3.692644
H	-2.905476	0.000000	-2.543886
O	-1.864785	0.000000	-0.759759
C	-0.233224	0.000000	0.968356
C	0.071027	0.000000	2.137449
C	0.432289	0.000000	3.550081
H	1.520328	0.000000	3.668542
H	0.033980	-0.886878	4.053312
H	0.033980	0.886878	4.053312
C	1.691732	0.000000	-1.565153
H	2.181858	0.000000	-0.570636
O	2.343291	0.000000	-2.588096

16

Conformer 2 (-287812.08 kcal/mol)

LUMO = 0.26 eV

Atom	x	y	z
C	-0.454207	0.000000	-0.497764
C	0.536014	0.000000	-1.456042
C	-0.137715	0.000000	-2.720353
C	-1.461020	0.000000	-2.439446
H	0.318420	0.000000	-3.700174
H	-2.352337	0.000000	-3.046359
O	-1.668378	0.000000	-1.098091
C	-0.415880	0.000000	0.911831
C	-0.369734	0.000000	2.118470
C	-0.288378	0.000000	3.573053
H	0.758088	0.000000	3.893587
H	-0.775810	-0.886714	3.991264
H	-0.775810	0.886714	3.991264
C	1.982495	0.000000	-1.227741
H	2.592506	0.000000	-2.156220
O	2.511748	0.000000	-0.137279

References

(1) M. J. Frisch, G. W. Trucks, H. B. Schlegel, G. E. S.; M. A. Robb, J. R. Cheeseman, G. Scalmani, V. B.; G. A. Petersson, H. Nakatsuji, X. Li, M. Caricato, A. V. M.; J. Bloino, B. G. Janesko, R. Gomperts, B. Mennucci, H. P. H.; J. V. Ortiz, A. F. Izmaylov, J. L. Sonnenberg, D. W.-Y.; F. Ding, F. Lipparini, F. Egidi, J. Goings, B. Peng, A. P.; T. Henderson, D. Ranasinghe, V. G. Zakrzewski, J. Gao, N. R.; G. Zheng, W. Liang, M. Hada, M. Ehara, K. Toyota, R. F.; J. Hasegawa, M. Ishida, T. Nakajima, Y. Honda, O. Kitao, H. N.; T. Vreven, K. Throssell, J. A. Montgomery, Jr., J. E. P.; F. Ogliaro, M. J. Bearpark, J. J. Heyd, E. N. Brothers, K. N. K.; V. N. Staroverov, T. A. Keith, R. Kobayashi, J. N.; K. Raghavachari, A. P. Rendell, J. C. Burant, S. S. I.; J. Tomasi, M. Cossi, J. M. Millam, M. Klene, C. Adamo, R. C.; J. W. Ochterski, R. L. Martin, K. Morokuma, O. F.; J. B. Foresman, D. J. F. Gaussian 16. Gaussian, Inc.: Wallingford, CT 2016.

(2) Becke, A. D. Density-Functional Thermochemistry. III. The Role of Exact Exchange. *J. Chem. Phys* **1993**, *98*, 5648. https://doi.org/10.1063/1.464913.

(3) Lee, C.; Yang, W.; Parr, R. G. Development of the Colle-Salvetti Correlation-Energy Formula into a Functional of the Electron Density. *Phys. Rev. B* **1988**, *37* (2), 785–789. https://doi.org/10.1103/PhysRevB.37.785.

(4) Grimme, S.; Antony, J.; Ehrlich, S.; Krieg, H. A Consistent and Accurate Ab Initio Parametrization of Density Functional Dispersion Correction (DFT-D) for the 94 Elements H-Pu. *J. Chem. Phys.* **2010**, *132* (15). https://doi.org/10.1063/1.3382344.

(5) Chai, J. Da; Head-Gordon, M. Long-Range Corrected Hybrid Density Functionals with Damped Atom-Atom Dispersion Corrections. *Phys. Chem. Chem. Phys.* **2008**, *10* (44), 6615–6620. https://doi.org/10.1039/b810189b.

(6) Marenich, A. V.; Cramer, C. J.; Truhlar, D. G. Universal Solvation Model Based on Solute Electron Density and on a Continuum Model of the Solvent Defined by the Bulk Dielectric Constant and Atomic Surface Tensions. *J. Phys. Chem. B* **2009**, *113* (18), 6378–6396. https://doi.org/10.1021/jp810292n.

(7) Legault, C. Y. CYLview, 1.0b. Université de Sherbrooke 2009. http://www.cylview.org.

(8) Contreras-García, J.; Johnson, E. R.; Keinan, S.; Chaudret, R.; Piquemal, J. P.; Beratan, D. N.; Yang, W. NCIPLOT: A Program for Plotting Noncovalent Interaction Regions. *J. Chem. Theory Comput.* **2011**, *7* (3), 625–632. https://doi.org/10.1021/ct100641a.

(9) The PyMOL Molecular Graphics System v. 2.5.0. Schrödinger, LLC.

(10) Deihl, E.; Jesikiewicz, L.; Newman, L.; Liu, P.; Brummond, K. Rh(I)-Catalyzed Allenic Pauson–Khand Reaction to Access the Thapsigargin Core: Influence of Furan and Allenyl Chloroacetate Groups on Enantioselectivity. *Org. Lett.* **2022**, *24* (4), 995–999. https://doi.org/10.1021/acs.orglett.1c03951.

(11) Burrows, L. C.; Jesikiewicz, L. T.; Lu, G.; Geib, S. J.; Liu, P.; Brummond, K. M. Computationally Guided Catalyst Design in the Type i Dynamic Kinetic Asymmetric Pauson-Khand Reaction of Allenyl Acetates. *J. Am. Chem. Soc.* **2017**, *139* (42), 15022–15032. https://doi.org/10.1021/jacs.7b07121.

(12) Frisch, M. J.; Trucks, G. W.; Schlegel, H. B.; Scuseria, G. E.; Robb, M. A.; Cheeseman, J. R.; Scalmani, G.; Barone, V.; Mennucci, B.; Petersson, G. A.; Nakatsuji, H.; Caricato, M.; Li, X.; Hratchian, H. P.; Izmaylov, A. F.; Bloino, J.; Zheng, G.; Sonnenberg, J. L.; Hada, M.; Ehara, M.; Toyota, K.; Fukuda, R.; Hasegawa, J.; Ishida, S94 M.; Nakajima, T.; Honda, Y.; Kitao, O.; Nakai, H.; Vreven, T.; Montgomery, J. A., Jr.; Peralta, J. E.; Ogliaro, F.; Bearpark, M.; Heyd, J. J.; Brothers, E.; Kudin, K. N.; Staroverov, V. N.; Kobayashi, R.; Normand, J.; Raghavachari, K.; Rendell, A.; Burant, J. C.; Iyengar, S. S.; Tomasi, J.; Cossi, M.; Rega, N.; Millam, N. J.; Klene, M.; Knox, J. E.; Cross, J. B.; Bakken, V.; Adamo, C.; Jaramillo, J.; Gomperts, R.; Stratmann, R. E.; Yazyev, O.; Austin, A. J.; Cammi, R.; Pomelli, C.; Ochterski, J. W.; Martin, R. L.; Morokuma, K.; Zakrzewski, V. G.; Voth, G. A.; Salvador, P.; Dannenberg, J. J.; Dapprich, S.; Daniels, A. D.; Farkas, Ö.; Foresman, J. B.; Ortiz, J. V.; Cioslowski, J.; Fox, D. J. Gaussian 09, Revision D.01; Gaussian, Inc.: Wallingford, CT, 2009
